# Repurposing Dihydroartemisinin to Combat Oral Squamous Cell Carcinoma, Associated with Mitochondrial Dysfunction and Oxidative Stress

**DOI:** 10.1155/2023/9595201

**Published:** 2023-02-16

**Authors:** Shanwei Shi, Huigen Luo, Yuna Ji, Huiya Ouyang, Zheng Wang, Xinchen Wang, Renjie Hu, Lihong Wang, Yun Wang, Juan Xia, Bin Cheng, Baicheng Bao, Xin Li, Guiqing Liao, Baoshan Xu

**Affiliations:** ^1^Hospital of Stomatology, Guangdong Provincial Key Laboratory of Stomatology, Guanghua School of Stomatology, Institute of Stomatological Research, Sun Yat-sen University, Guangzhou, Guangdong Province, China; ^2^Hospital of Stomatology, Department of Oral and Maxillofacial Surgery, Guangdong Provincial Key Laboratory of Stomatology, Guanghua School of Stomatology, Sun Yat-sen University, Guangzhou, Guangdong Province, China; ^3^National Engineering Research Center of JUNCAO Technology, Fujian Agriculture and Forestry University, Fuzhou, Fujian, China

## Abstract

Oral squamous cell carcinoma (OSCC), with aggressive locoregional invasion, has a high rate of early recurrences and poor prognosis. Dihydroartemisinin (DHA), as a derivative of artemisinin, has been found to exert potent antitumor activity. Recent studies reported that DHA suppresses OSCC cell growth and viability through the regulation of reactive oxygen species (ROS) production and mitochondrial calcium uniporter. However, the mechanism underlying the action of DHA on OSCCs remains elusive. In the study, we observed that 159 genes were remarkably misregulated in primary OSCC tumors associated with DHA-inhibited pathways, supporting that OSCCs are susceptible to DHA treatment. Herein, our study showed that DHA exhibited promising effects to suppress OSCC cell growth and survival, and single-cell colony formation. Interestingly, the combination of DHA and cisplatin (CDDP) significantly reduced the toxicity of CDDP treatment alone on human normal oral cells (NOK). Moreover, DHA remarkably impaired mitochondrial structure and function, and triggered DNA damage and ROS generation, and activation of mitophagy. In addition, DHA induced leakage of cytochrome C and apoptosis-inducing factor (AIF) from mitochondria, elevated Bax/cleaved-caspase 3 expression levels and compromised Bcl2 protein expression. In the OSCC tumor-xenograft mice model, DHA remarkably suppressed tumor growth and induced apoptosis of OSCCs *in vivo*. Intriguingly, a selective mitophagy inhibitor Mdivi-1 could significantly reinforce the anticancer activity of DHA treatment. DHA and Mdivi-1 can synergistically suppress OSCC cell proliferation and survival. These data uncover a previously unappreciated contribution of the mitochondria-associated pathway to the antitumor activity of DHA on OSCCs. Our study shed light on a new aspect of a DHA-based therapeutic strategy to combat OSCC tumors.

## 1. Introduction

Oral squamous cell carcinoma (OSCC) is a common tumor type in the oral cavity, with about 300,000 new cases occurring each year [1]. OSCC is a challenging disease to treat and cure, and it has been characterized by frequent malignant metastasis and early recurrences [2]. Cisplatin (CDDP) chemotherapy has been extensively recognized as a standard treatment for patients in the management of unresectable OSCC tumors [3]. Although CDDP chemotherapy improves outcomes, its adverse effects, increased toxicity and drug resistance lead to a substantial diminution of the patient's life quality, challenging its treatment paradigm [4]. Aberrant epidermal growth factor receptor (EGFR) pathway activation is highly associated with OSCC development and progression [5]. The EGFR-targeted drug of OSCC is cetuximab, a chimeric human-murine IgG1 mAb that inhibits EGFR [6]. However, neither cetuximab nor other EGFR tyrosine kinase inhibitors (e.g., Gefitinib and Erlotinib) have been demonstrated to be more effective than the standard cisplatin treatment in OSCC clinical stage [7, 8]. Despite cancer immunotherapy being actively explored in the past few decades, the overall response rates of these immuno-oncology therapies with PD-L1 and PD-1 antibodies in OSCC diseases are very modest at only ~20% [9–11]. Therefore, it is an urgent demand to develop novel effective therapeutic schemes for OSCC treatment.

Dihydroartemisinin (DHA) is a derivative of artemisinin which is originally isolated from the plant *Artemisia annua* in 1972 [12]. DHA is an active metabolite of all kinds of artemisinin compounds (artemisinin, artesunate, artemether, etc.) and is ~5 times more effective than artemisinin against malaria [12–14]. DHA is recently reported to have some antineoplastic activity against various cancers [12]. Sun et al. firstly reported that artemisinin inhibited cell growth and viability of murine leukemia cell line P388, human hepatoma cell line SMMC-7721, and human gastric cancer cell line SGC-7901 [15]. Some studies have demonstrated that DHA acts as an antineoplastic agent by a variety of mechanisms, depending on the cancer type. For instance, DHA inhibits cell proliferation and induces apoptosis of rhabdomyosarcoma tumors by suppressing mTORC1 pathway, via triggering AMPK signaling [16, 17]. Chen et al. reported that DHA can sensitize a panel of cancer cells to ferroptosis by iron-dependent reactive oxygen species (ROS) generation [18]. Lin et al. indicated that DHA induces ferroptosis and apoptosis of head and neck carcinoma cells [19]. DHA inhibits the proliferation and migration of bladder cancer cells by compromising the expression of histone demethylase KDM3A and inducing p21 expression [20]. DHA can also serve as a potent inhibitor of translationally controlled tumor protein (TCTP) to impede gallbladder cancer metastasis, associated with cell division control protein 42 homolog (Cdc42) activity [21]. In addition, DHA triggers cell death of glioma cells through PERK/ATF4/HSPA5 signaling [22]. Moreover, DHA suppresses esophageal cancer cell growth by downregulation of pyruvate kinase M2 and glycolysis [23]. Most recently, DHA treatment has been reported to enhance the drug sensitivity of some tumor cells to conventional chemotherapy [24]. DHA dimer increases the cytotoxicity of a combination of folinic acid (Fol), 5-fluorouracil (5-FU), and oxaliplatin (OX) in colon cancer cells, which is a standard of care treatment for colorectal cancer [25]. Chen et al. demonstrated that DHA combined with dexamethasone can conquer dexamethasone resistance and strengthen its effect in multiple myeloma through ROS generation and cytochrome C translocation from the mitochondria to the cytoplasm [26]. Furthermore, a couple of recent studies report that DHA inhibits OSCC cell growth and survival by the regulation of reactive oxygen species (ROS) generation and mitochondrial calcium uniporter [27, 28]. Although the previous studies indicated that DHA can target each of some signaling pathways in a cancer type-dependent manner, the underlying mechanism of DHA treatment against OSCC is still elusive.

Autophagy is an evolutionarily conserved pathway by degrading a wide variety of cellular components to meet the metabolic needs of the cell and the renewal of certain organelles (mitochondria, peroxisomes, endoplasmic reticulum, etc.). Mitochondria is an important organelle of both autophagy and apoptosis. One of the triggers for mitochondrial dysfunction is intracellular ROS. Mitophagy, which is a selective type of autophagy, is a major pathway for the degradation of dysfunctional mitochondria to prevent the deleterious effects of damaged mitochondria [29]. Although a few studies have reported that DHA could affect mitochondrial function and autophagy to induce cell apoptosis and ferroptosis [30–33], whether mitophagy is actively involved in DHA-induced tumor cell death is still unclear. Herein we hypothesized that DHA works as an antitumor agent primarily by targeting mitochondrial-associated signaling pathways.

In our study, we investigated the anticancer activity of DHA on OSCC cell growth and survival, linking to alterations in mitochondrial function. We found that DHA remarkably hindered cell growth and proliferation, and induced cell apoptosis of OSCCs. Subsequently, we identified that DHA treatment altered mitochondrial permeability, which in turn released cytochrome C and apoptosis-inducing factor (AIF). Moreover, *in vivo* treatment of DHA remarkably suppressed tumor growth and survival in the OSCC-xenografted mice model. Of interest, DHA remarkably triggered the mitophagy pathway, and a mitochondrial division/mitophagy inhibitor Mdivi-1 could significantly reinforce the antineoplastic activity of DHA on OSCC cells. Collectively, our findings support that a DHA-based treatment strategy may be a novel avenue to combat oral cancer.

## 2. Results

### 2.1. Primary OSCC Tumors Exhibit a Remarkable Misregulation of Many Genes, Associated with DHA-Affected Pathways

**Figure 1:**
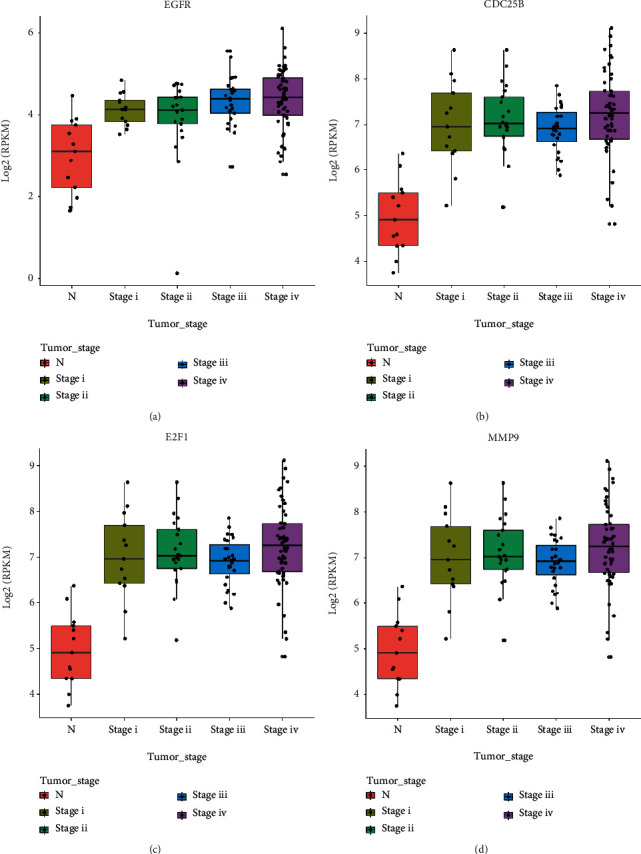
OSCC tumors exhibit a remarkable misregulation of gene expression associated with the DHA-inhibited signaling pathways. (a–d). Gene expression of EGFR (a), CDC25B (b), E2F1 (c), and MMP9 (d) was upregulated in all stages of OSCC tumor development compared to the matched normal tissues. Gene transcriptome data and the clinical information of 127 primary OSCC tumors and 13 adjacent normal tissues were downloaded from the TCGA public dataset GSE62944 (Table S1-2).

DHA has been reported to prevent cell growth of OSCC [34, 35]. To search for correlative genes of DHA-associated signaling pathways on OSCC progression and prognosis, we downloaded gene transcriptome data and the clinical information of 127 primary OSCC tumors and 13 adjacent normal tissues from the TCGA public dataset GSE62944 (Table S1-2). We kept genes with the gene expression value (FPKM) larger than 1 for further analysis. By bioinformatic analysis, the data have shown that transcription of 159 genes in the mitochondrial structure and function, cell cycle, cell survival, glycolytic metabolism, ferroptosis, and PI3K-Akt-mTOR pathway, are remarkably misregulated in the OSCC tumors, compared to the adjacent normal tissues (Figure S1 and Table S3). Interestingly, many of these genes (HIF1, MMP9, CDC25B, E2F1, TGF-*β*, FOXM1, CCNB1, ETS1, EGFR, ITGB1, and VEGF-C, etc.) are significantly upregulated at each stage of OSCC tumor development, associated with DHA-inhibited signaling pathways (Figure 1 and Figure S2) [36–43]. The data suggested that OSCC tumors are preferentially susceptible to DHA treatment.

### 2.2. DHA Significantly Inhibited Cell Growth and Proliferation of OSCCs

**Figure 2:**
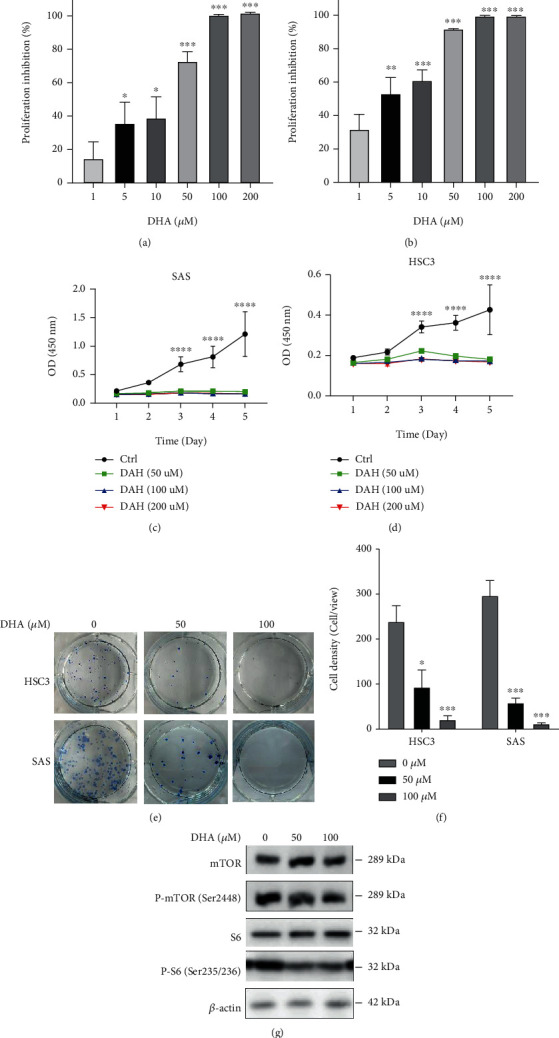
DHA significantly inhibited cell growth and proliferation of the OSCC tumor cells. (a, b). SAS (a) and HSC3 (b) OSCC tumor cells were treated by DHA with various concentrations (1, 5, 10, 50, 100, and 200 *μ*M) for 72 hours. The inhibition ratio of cell proliferation was measured by CCK-8 assay, and the percentages of proliferation inhibition were normalized by DMSO vesicle control groups. The experiments were conducted by three biological replicates (*t*-test, ^∗^*p* < 0.05, ^∗∗^*p* < 0.01, ^∗∗∗^*p* < 0.001; means ± SE, *n* = 3). (c, d). SAS (c) and HSC3 (d) cells were exposed to the gradient concentrations of DHA (0, 50, 100, 200 *μ*M) from 1 to 5 days. Cell growth was assessed by OD value at 450 nm (*t*-test, ^∗^*p* < 0.05, ^∗∗^*p* < 0.01; means ± SE, *n* = 3). (e). The single-cell colony formation capacity was assessed in HSC3 and SAS cells with the DHA treatments (0, 50, 100 *μ*M). (f). The percentages of single-cell colony formation were quantified in HSC3 and SAS cells with the indicated treatments in (e) (*t*-test, ^∗^*p* < 0.05, ^∗∗∗^*p* < 0.001; means ± SE, *n* = 3). (g). The OSCC cells were treated with DHA in a dosage-dependent manner for 12 hours. Protein expression levels of mTOR signaling pathways were assessed by Western blotting analysis.

Given the gene expression analysis, we study the anticancer activity of DHA on OSCC tumor cells. CCK-8 assay was carried out to assess cell proliferation and viability of OSCC tumor cells. HSC3 and SAS cells were treated with diverse concentrations of DHA (0, 1, 5, 10, 50, 100, 200 *μ*M) for 72 hours. As shown in Figures 2(a) and 2(b), DHA suppressed the proliferation and growth of the OSCC tumor cells in a dosage-dependent manner (1 *μ*M: 14.72 ± 5.35% for SAS cells, 32.27 ± 4.23% for HSC3 cells), (5 *μ*M: 36.27 ± 6.67% for SAS cells, *p* = 0.0451; 53.42 ± 4.58% for HSC3 cells, *p* = 0.0098), (50 *μ*M: 73.36 ± 5.34% for SAS cells, *p* < 0.0001; 92.46 ± 1.37% for HSC3 cells, *p* < 0.0001). Also, HSC3 and SAS cells were treated with the gradient concentrations of DHA (0, 50, 100, 200 *μ*M) from 1 to 5 days. The data suggest that DHA markedly hindered the growth of SAS and HSC3 cells in a time-dependent manner (Figures 2(c) and 2(d)). Furthermore, DHA remarkably inhibited single-cell colony formation of OSCC cells in a dosage-dependent manner, as shown in Figures 2(e) and 2(f) (0 *μ*M: 237.70 ± 21.87 for HSC3 cells, 297.00 ± 20.60 for SAS cells), (50 *μ*M: 93.00 ± 23.52 for HSC3 cells, *p* = 0.0108; 57.00 ± 7.234 for SAS cells, *p* = 0.0004), (100 *μ*M: 21.00 ± 6.24 for HSC3 cells, *p* < 0.001; 9.33 ± 2.66 for SAS cells, *p* < 0.001). Increasing studies reported that DHA inhibits cell growth of several cancer types through inhibition of mTOR signaling pathway [16, 23]. Here our data verified that DHA inhibited phosphorylation of mTOR (Ser2448) and S6 ribosomal protein (Ser235/236) of SAS OSCC cells with 12 h treatment (Figure 2(g) and Figure S3).

### 2.3. DHA Induced Cell Apoptosis of OSCCs in a Caspase-3-Dependent Manner

**Figure 3:**
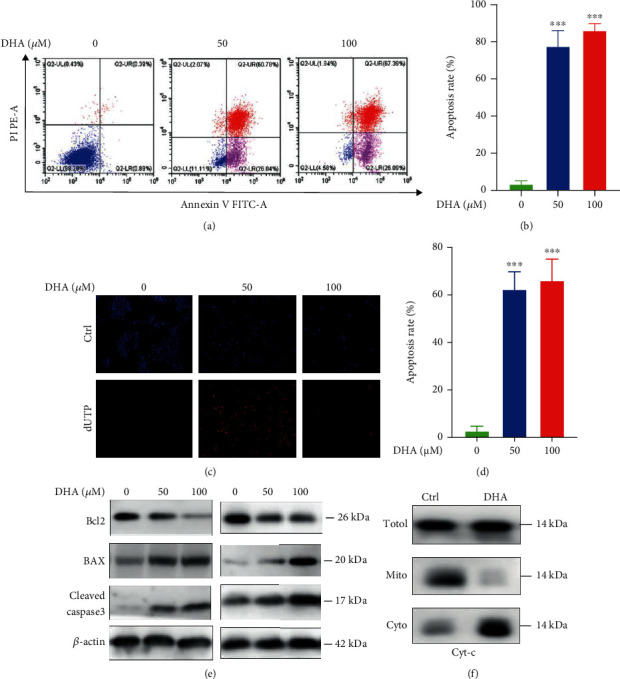
DHA remarkably induced cell apoptosis of the OSCC tumor cells. (a). Annexin V and PI double-staining was used to measure cell apoptosis levels in the OSCC SAS cells with the indicated treatments for 72 hours. (b). Qualification of the cell apoptosis was analyzed in (a) (^∗∗∗^*p* < 0.001; means ± SE, ns, not significant, *n* = 3). (c). dUTP staining was used to measure cell apoptosis levels in the OSCC HSC3 cells with the indicated treatments for 72 hours. (d). Qualification of the cell apoptosis was analyzed in (c) (^∗∗∗^*p* < 0.001; means ± SE, ns, not significant, *n* = 3). The experiments were conducted by three biological replicates. (e). Western blot analysis was carried out in the OSCC cells with the indicated treatments for 24 hours. (f). The OSCC cells were incubated with or without DHA. Then mitochondria and cytoplasm parts were separated by a Beyoeimekit (#C3601). The protein expression of cytochrome C (Cyt-c) was, respectively, detected in the total cell lysates, mitochondria part, and cytoplasm part.

To further study the action of DHA on cellular survival of OSCCs, we have conducted flow cytometry analysis to elucidate cell apoptosis levels of SAS cells with DHA treatment for 48 h, using Annexin V/PI dual staining. The data indicated that DHA significantly induced cell apoptosis (50 *μ*M: 78.47 ± 4.86%, *p* = 0.0001; 100 *μ*M: 87.00 ± 2.08%, *p* < 0.0001) dosage-dependently, compared with the control group (0 *μ*M: 3.46 ± 0.95%) (Figures 3(a) and 3(b)). Also, we have conducted a TUNEL assay to test cell apoptosis of OSCCs with 48 h treatment of DHA. As shown in Figures 3(c) and 3(d), treatment of DHA induced a remarkable increase of apoptotic cells (red fluorescence light). To dissect the underlying mechanism of DHA-induced apoptosis, we performed Western blotting analysis to assess some apoptosis signaling pathway with 24 h treatment of DHA (100 *μ*M). DHA displayed an obvious inhibition on the protein expression levels of antiapoptosis factor Bcl2, activated the proapoptosis factor Bax and cleaved caspase-3 (C-caspase-3) of HSC3 and SAS cells dosage-dependently (Figure 3(e) and Figure S4(a)). In addition, we have separated mitochondrial protein and cytoplasmic protein, respectively, to find that DHA markedly induced mitochondrial c-type cytochrome (Cyt-c) movement to the cytoplasm via immunoblotting analysis (Figure 3(f) and Figure S4(b)). Since mitochondrial Cyt-c triggers cytoplasmic caspase-3 signaling, our data have revealed that DHA induced apoptosis through a caspase-3-dependent manner.

### 2.4. DHA Preferentially Suppressed Cell Proliferation and Survival of OSCC Cells Rather than NOK Cells, Compared to Cisplatin

**Figure 4:**
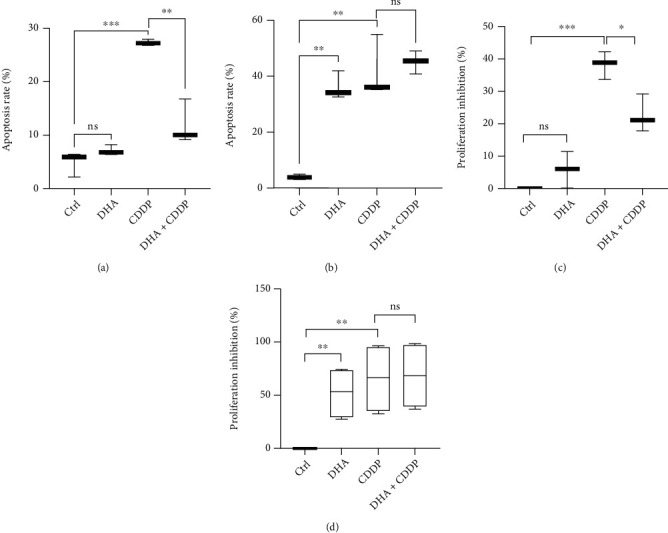
DHA preferentially suppressed proliferation and survival of OSCC tumor cells rather than NOK cells, compared to cisplatin. (a). NOK cells were treated with DHA, CDDP, and the combination (DHA + CDDP) for 48 h. Cell apoptosis was measured with Annexin V-FITC/PI dual staining by flow cytometry analysis (*t*-test, ^∗∗^*p* < 0.01, ^∗∗∗^*p* < 0.001; means ± SE, ns, not significant, *n* = 3). (b). HSC3 OSCC tumor cells were treated with DHA, CDDP, and the combination (DHA + CDDP) for 48 hours. Cell apoptosis was measured with flow cytometry analysis (*t*-test, ^∗∗^*p* < 0.01, ^∗∗∗^*p* < 0.001; means ± SE, ns, not significant, *n* = 3). (c). NOK cells were treated with DHA, CDDP, and the combination (DHA + CDDP) for 48 hours. The inhibition ratio of cell proliferation in NOK cells was measured by CCK-8 assay (*t*-test, ^∗^*p* < 0.05, ^∗∗∗^*p* < 0.001; means ± SE, ns, not significant, *n* = 3). (d). HSC3 cells were treated with DHA, CDDP, and the combination (DHA + CDDP) for 48 hours. The inhibition ratio of cell proliferation in HSC3 cells was measured by CCK-8 assay (*t*-test, ^∗∗^*p* < 0.01; means ± SE, ns, not significant, *n* = 3).

Since cisplatin (CDDP) treatment is a chemotherapy paradigm for OSCC tumors, we compared toxicity between DHA and CDDP on human normal squamous cells (NOK) and OSCC cells (HSC3) by Annexin V-FITC/propidium iodide (PI) dual staining (Figures 4(a) and 4(b) and Figure S5). DHA treatment did not have a cytotoxic activity for NOK cells compared to that in CDDP. Interestingly, the combination treatment of DHA and CDDP significantly reduced the toxicity of single CDDP treatment alone on NOK cells (Figure 4(a)). Furthermore, DHA treatment displayed similar cytotoxicity compared with CDDP on HSC3 cells, but the combined treatment of DHA and CDDP did not significantly induce more apoptosis of the OSCC cells (Figure 4(b)). In addition, we also used the CCK-8 assay to detect cellular toxicity with DHA and CDDP treatments on the NOK and OSCC cells. The results have indicated that OSCC tumor cells are more sensitive to the antiproliferative effects of DHA and CDDP treatments than NOK cells, which are well consistent with the Annexin V/PI dual staining (Figures 4(c) and 4(d)). Collectively, these results have indicated that DHA significantly impeded cell growth and proliferation, and single-cell colony formation of OSCC tumor cells, with few toxic effect on human normal oral cells.

### 2.5. DHA Induced Cellular ROS Generation to Trigger Cell Apoptosis

**Figure 5:**
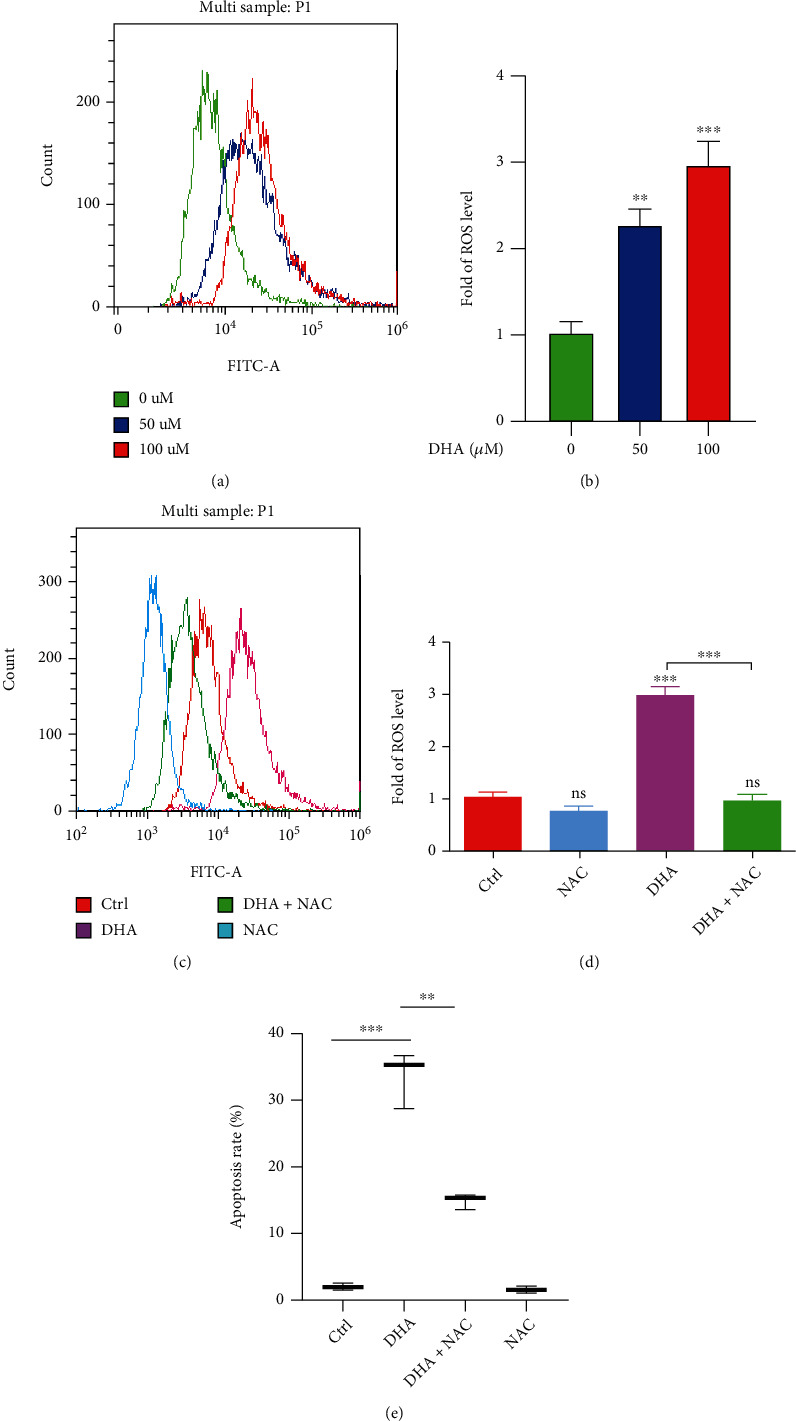
DHA induced ROS generation of the OSCC cells. (a). Intracellular ROS generation was measured using an oxidation-sensitive fluorescent probe (DHE) by flow cytometry analysis in the OSCC tumor cells with the indicated treatments of DHA (0, 50, 100 *μ*M) for 24 h. (b). Qualification of cellular ROS generation was analyzed in (a) (*t*-test, ^∗∗^*p* < 0.01, ^∗∗∗^*p* < 0.001; means ± SE, ns, not significant, *n* = 3). (c). Intracellular ROS generation was measured using an oxidation-sensitive fluorescent probe by flow cytometry analysis in the tumor cells with the DHA treatment in the presence or absence of NAC for 24 h. (d). Qualification of cellular ROS generation was analyzed in (c) (*t*-test, ^∗∗∗^*p* < 0.001; means ± SE, ns, not significant, *n* = 3). (e). OSCC HSC3 tumor cells were treated by DHA for 48 h with or without preincubation of NAC. Cell apoptosis was measured with Annexin V-FITC/PI dual staining by flow cytometry analysis (*t*-test, ^∗∗^*p* < 0.01, ^∗∗∗^*p* < 0.001; means ± SE, ns, not significant, *n* = 3).

Mitochondria is the main organelle of intracellular ROS generation, and also it is sensitive to oxidative stress. Since DHA induced mitochondrial Cyt-c releasing and inhibited mitochondria movement, we wondered whether DHA caused cellular oxidative stress in the OSCCs. With DCFH-DA staining, DHA remarkably induced intracellular ROS generation in a dosage-dependent manner (Figures 5(a) and 5(b)). Moreover, we have performed the rescue experiment about Mito-ROS scavenger N-acetyl-l-cysteine (NAC) on the OSCC tumor cells. The results validated that preincubation of NAC significantly blunted DHA-induced cellular ROS generation and cytotoxicity in the OSCC cells (Figures 5(c)–5(e) and Figure S6).

### 2.6. Treatment of DHA Impaired Mitochondrial Function

**Figure 6:**
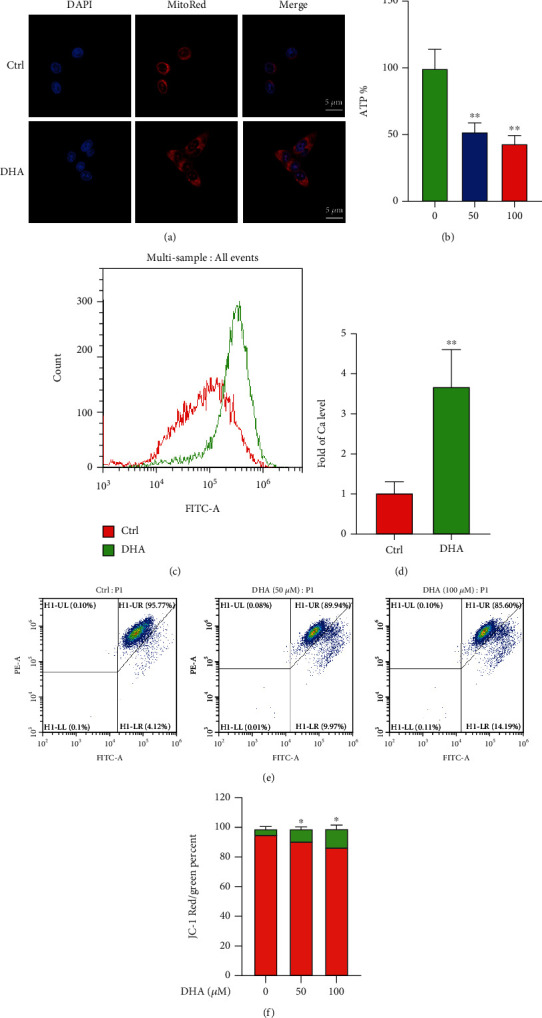
DHA impaired the mitochondrial structure and function of the OSCC cells. (a). MitoRed fluorescence staining was conducted in the OSCC cells with or without DHA treatment. (b). Cellular ATP biosynthesis was measured in the tumor cells for the indicated treatments of DHA (0, 50, 100 *μ*M). (c). Mitochondrial membrane integrity was assessed in the OSCC cells with or without DHA treatment through mitochondrial Ca^2+^ releasing. (d). Qualification of mitochondrial Ca^2+^ releasing was analyzed in (c) (*t*-test, ^∗∗^*p* < 0.01; means ± SE, ns, not significant, *n* = 3). (e). Mitochondrial membrane depolarization was assessed using the fluorescent dye JC-1 staining. (f). Qualification of mitochondrial Ca^2+^ releasing was analyzed in (e) (*t*-test, ^∗^*p* < 0.05; means ± SE, ns, not significant, *n* = 3).

Subsequently, we performed MitoRed fluorescence staining to find that DHA induced a striking leakage of mitochondrial contents, which were highly enriched in the perinuclear and nucleus of OSCC cells (Figure 6(a)). Excessive ROS generation impairs ATP biosynthesis and triggers mitochondrial Ca^2+^ releasing, leading to the opening of mitochondrial membrane permeability transport pores (MPT) and the decrease of the mitochondrial membrane potential (Δ*ψm*) [44]. MPT opening and Δ*ψm* decreasing further mitochondrial cause Ca^2+^ rising. The forming circuit can promote apoptosis in a mitochondria-dependent pathway [45]. Consistently, our data validated that DHA significantly reduced cellular ATP synthesis and elevated cytosolic Ca^2+^ levels in the OSCCs for 12 h administration (Figures 6(b)–6(d)). To further illustrate the effect of DHA on mitochondria, we conducted a MitoPT JC-1 assay to detect mitochondrial membrane depolarization using the fluorescent dye JC-1 staining. OSCC cells were treated with various concentrations of DHA (0, 50, 100 *μ*M) for 12 h. With flow cytometry analysis, these data have reflected that DHA induced a partial loss of mitochondrial membrane (Figures 6(e) and 6(f)), which indicates that DHA caused mitochondrial defects. Hence, our study strongly indicated that DHA-induced mitochondrial damages contributed to the antitumor activity of DHA on OSCCs.

### 2.7. DHA Triggered DNA Damage and Mitochondrial AIF Moving into the Nucleus

**Figure 7:**
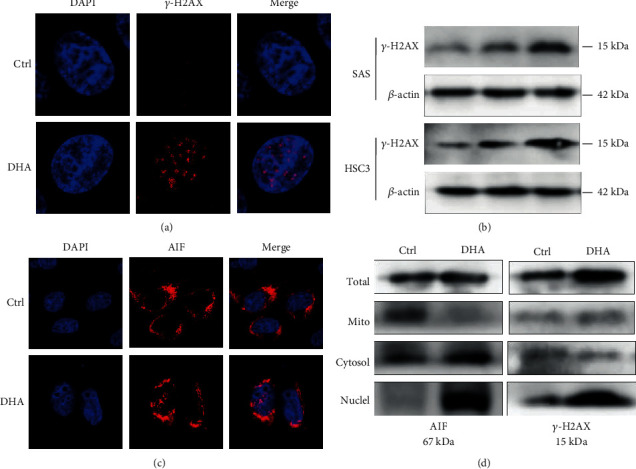
DHA triggered DNA damage and mitochondrial AIF moving into the nucleus. (a). The formation of *γ*-H2AX foci was monitored upon the indicated treatments of DHA in the OSCC cells for 24 h. Scale bar: 5 *μ*m. (b). Western blot analysis was carried out with antibodies against *γ*-H2AX in SAS and HSC3 cells with the indicated treatments of DHA for 24 h. (c). Cellular expression and localization of AIF protein were monitored upon the indicated treatments of DHA in the OSCC cells for 24 h. Scale bar: 5 *μ*m. (d). Protein expression of AIF and *γ*-H2AX were, respectively, assessed in the total cell lysates, mitochondria part, cytoplasm part, and cell nucleus.

Previous studies reported that DHA was able to induce DNA damage of several tumor types. Since our data illuminated that DHA caused mitochondrial contents to be released into the nucleus, we asked whether DHA-induced mitochondria defects could contribute to genomic DNA damage. Herein we verified that DHA remarkably triggered DNA double-strand breaks with *γ*-H2AX IF staining, after 24 h treatment of DHA in SAS cells (Figure 7(a) and Figure S7(a)). Additionally, we conducted Western blotting analysis to confirm that DHA induced *γ*-H2AX increasing SAS and HSC3 cells in a dosage-dependent manner (Figure 7(b) and Figure S7(b)). Mitochondrial apoptosis-inducing factor (AIF) can maintain the oxidative homeostasis of mitochondria niche, and nucleus-localized AIF causes chromatin agglutination and DNA rupture [46]. As expected, we found that AIF signaling was localized around outside of the nucleus in the control group without DHA treatment, while AIF was transported into the nucleus with 24 h treatment of DHA (100 *μ*M) (Figure 7(c) and Figure S7(c)). To validate the IF staining, we separated mitochondria, cytoplasm, and nucleus fragments of the OSCC cells with or without 24 h treatment of DHA (100 *μ*M) for Western blotting analysis. The purity of mitochondria part has been validated by the mitochondrial loading control COX IV immunoblotting in the OSCC tumor cells (Figure S7(d) and (e)). The data further confirmed that DHA strikingly induced mitochondrial AIF transporting into the nucleus and the cytosolic fractions, and activated nucleus *γ*-H2AX increasing (Figure 7(d) and Figure S7(f) and (g)). Given mitochondrial AIF protein is a caspase 3-independent death effector [47], the results indicated that DHA could disrupt mitochondrial oxidation homeostasis, and also trigger a caspase 3-independent pathway through mitochondrial AIF movement into the nucleus and cytosol.

### 2.8. Treatment of DHA Remarkably Inhibits OSCC Tumor Growth and Survival In Vivo

**Figure 8:**
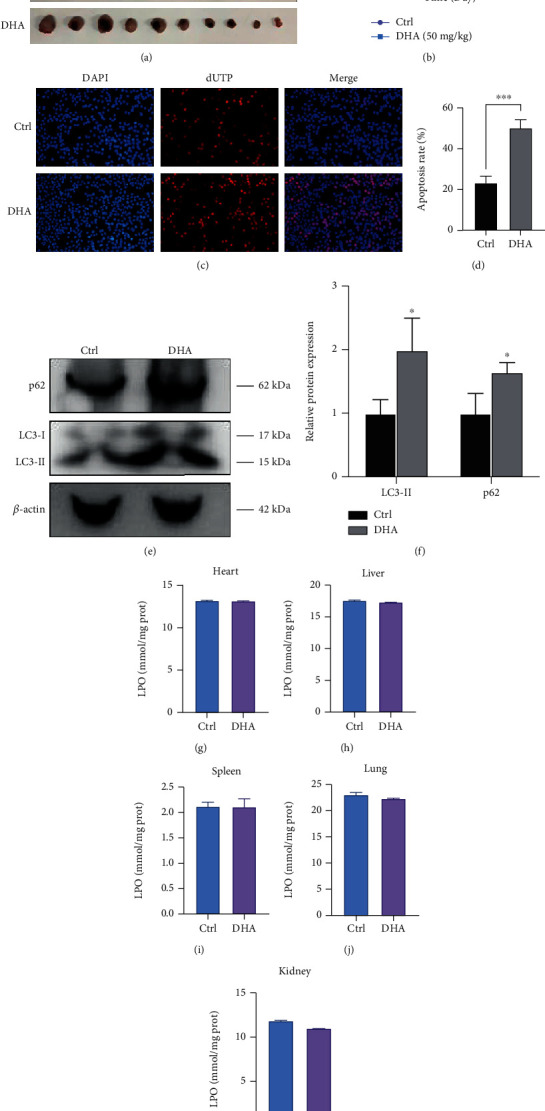
Treatment of DHA remarkably inhibits OSCC tumor growth and survival *in vivo*. (a). The tumor sizes of the tumors were imaged in the OSCC-xenograft mice with or without DHA treatment (*n* = 10). (b). Growth curves of the xenograft tumors were measured in the group with the indicated treatments (*t*-test, ^∗∗∗^*p* < 0.001; means ± SE, ns, not significant, *n* = 3). (c). The apoptotic cells in the tumors were stained by dUTP Immunofluorescence (IF) solution in the tumors with the indicated treatments. DAPI was used for nuclear staining. Scale bar: 20 *μ*m. (d). The percentages of dUTP IF staining in (c) were conducted in the quantitative analysis. The IF data was conducted quantitative analysis by the ImageJ software (*t*-test, ^∗∗^*p* < 0.01; means ± SE, ns, not significant, *n* = 3). (e). Expression of LC3-II and p62 were detected by Western blotting in the tumors with or without DHA treatment *in vivo*. (f). Relative protein level was determind after normalization to *β*-actin (*t*-test, ^∗^*p* < 0.5; means ± SE, ns, not significant, *n* = 3). (g–k). Lipid hydroperoxide (LPO) levels were measured in the heart, liver, spleen, lung, and kidney tissues of tumor-bearing mice with or without DHA treatment *in vivo*. The experiments were conducted by three biological replicates.

In the study, we validated the *in vivo* therapeutic effects of DHA in an OSCC tumor-xenograft mouse model. OSCC tumor xenografts were obtained by subcutaneous injection of SAS cells into nude mice. After six days these tumor-bearing mice were randomly divided into two groups (*n* = 10 mice in each group) and then injected with the vehicle control and DHA for one month. As indicated in Figures 8(a) and 8(b), treatment of DHA robustly inhibited the growth of OSCC tumors compared with the control group (average tumor size, *p* < 0.05). In addition, DHA administration dramatically increased apoptosis levels of the tumors (apoptosis: 27.01 ± −3.297, *p* < 0.05), which was well agreed with the *in vitro* results (Figures 8(c) and 8(d)). Moreover, we detected the autophagy levels of OSCC tumor samples for *in vivo* assay. The results have shown that DHA administration increased the expression levels of LC3-II and p62 protein to induce autophagy in the OSCC cells, which is consistent with the in vitro experiment (Figures 8(e) and 8(f)). Subsequently, because DHA induces cellular ferroptosis coupled with increasing lipid hydroperoxide (LPO) levels [48], we assessed the *in vivo* toxicity and oxidative damage of DHA administration in the heart, liver, spleen, lung, and kidney tissues of tumor-bearing mice using a sensitive and reliable LPO assay kit. Notably, we did not observe any changes in LPO levels in the DHA-treated mice group for various tissues (Figures 8(g)–8(k)), indicating that treatment of DHA shows few adverse effects on various normal organs and tissues *in vivo*.

### 2.9. DHA Markedly Activated Mitophagy of OSCC Tumor Cells

**Figure 9:**
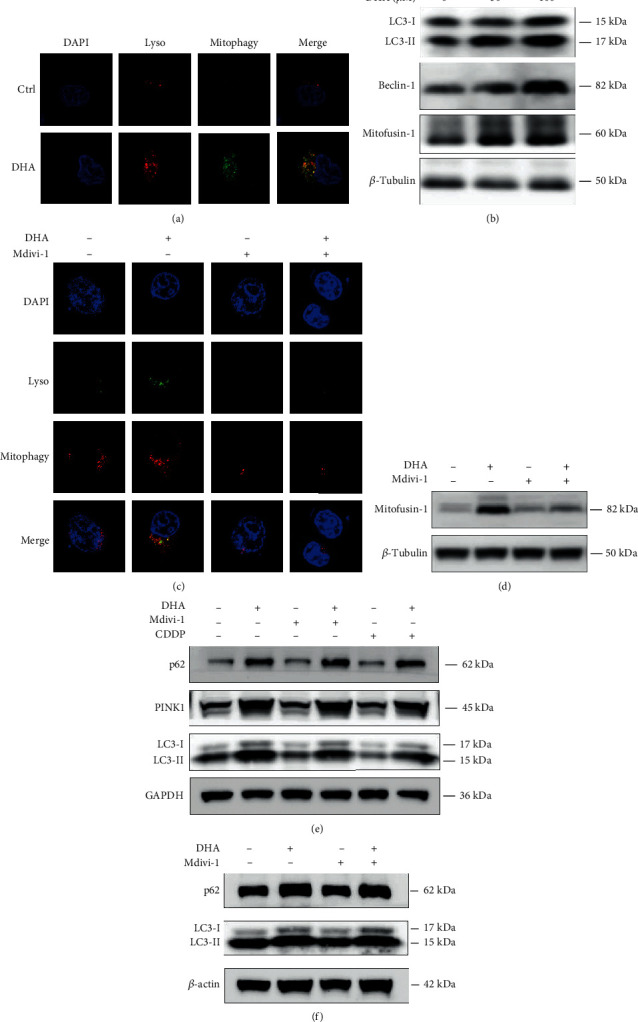
DHA activated mitophagy of the OSCC tumor cells. (a). Mitophagy and lysosome double-staining was used to measure mitochondrial structure and location within 24 hours in the OSCC cells with the indicated treatments for 24 hours. Scale bar: 5 *μ*m. (b). Western blot analysis was carried out with antibodies against LC3I/II, Beclin-1, and Mitofusin-1 in the OSCC cells with the indicated treatments for 24 hours. (c). MitoRed and lysosome double-staining was used to measure mitochondrial structure and location in the OSCC cells with or without DHA treatment in the presence or absence of Mdivi-1 preincubation for 24 h. Scale bar: 5 *μ*m. (d). Western blot analysis was carried out with antibody against Mitofusin-1 protein in the OSCC tumor cells with or without DHA and Mdivi-1 cotreatment for 24 h. (e). HSC3 cells were treated by DHA with or without Mdivi-1 or CDDP for 24 h. Western blot analysis was carried out with antibodies against p62, PINK1, and LC3-I/II. (f). NOK cells were treated by DHA with or without Mdivi-1 for 24 h. Western blot analysis was carried out with antibodies against p62 and LC3-I/II.

Mitophagy is a selective autophagic process for mitochondria [49]. Mitochondrial permeability transition (MPT) has been reported to be responsible for the mitophagy of depolarized mitochondria in mammalian cells [50]. The main function of mitophagy is to remove damaged mitochondria for maintaining cellular survival and growth. Since we have found that treatment of DHA triggered mitochondria injury and dysfunction, we subsequently investigated whether mitophagy could be involved in the process. Here we performed double staining on the OSCC tumor cells in the presence or absence of DHA treatment using mitophagy dye (Ex561 nm, Em650 nm) and lysosome dye (Ex488 nm, Em502-554 nm). Remarkably, treatment of DHA activated the mitophagy pathway (upregulation of lysosome and mitophagy signalings) compared with the DMSO control group (Figure 9(a)). Beclin 1 is a vital protein to promote autophagosome membrane formation. Mitofusin-1 protein serves as an important biomarker of mitophagy in mammalian cells [51]. Consistently, the protein expression levels of LC3-II, Beclin-1, and Mitofusin-1 were upregulated by DHA treatment (0, 50, 100 *μ*M) as shown in Figure 9(b) and Figure S8(a). To further evaluate the role of mitophagy signaling on the effect of DHA, SAS cells were treated by DHA with or without preincubation of a mitophagy inhibitor Mdivi-1 (a selective and cell-permeable mitochondrial fission inhibitor). By IF staining, DHA-triggered mitophagy activation was remarkably blunted by Mdivi-1 pretreatment (Figure 9(c)). Consistently, pretreatment of Mdivi-1 partially reverse DHA-induced mitofusin-1 protein increase (Figure 9(d) and Figure S8(b)). In mammalian cells, PINK1 protein plays a vital role in labeling damaged mitochondria and mediating mitophagy for recycling and disposal [52]. In the study, we monitored the protein expression of PINK1 in the OSCC cells. The results displayed that treatment of DHA significantly upregulated the expression of PINK1 protein, which further validated that DHA induced upregulation of mitophagy of the OSCC cells (Figure 9(e) and Figure S8(c)). Also, DHA upregulated the expression levels of p62 (SQSTM1, the autophagy substrate) and LC3-II protein in the OSCC cells. We observed that Mdivi-1 can partially alleviate DHA-upregulated LC3-II expression, but not p62 and PINK1. We also compared the DHA effect on autophagy between the OSCC and NOK cells by Western blotting analysis (Figure 9(f) and Figure S8(d)). DHA treatment appeared to induce much more autophagy levels (more expression of p62 and LC3-II protein) in the OSCC cells than those in NOK cells. The data suggested that DHA induced much more apoptosis events in the OSCC cells than those in NOK cells, associated with more upregulation of autophagy levels in the OSCC cells. Hence these data suggest that activation of mitophagy might partially protect OSCCs from DHA-induced cell death. Additionally, we used 3-Methyladenine (3-MA, inhibitor of autophagosome formation) to preincubate for 2 h, and then treat the OSCC tumor cells with or without DHA for 24 h (Figures S8(e)–(g)). As shown in the Western blotting analysis, 3-MA did not affect basal autophagy level, and 3-MA partially blunted DHA-induced upregulation of LC3-II and p62 protein in the OSCC tumor cells. For CCK8 assay analysis, 3-MA can mildly inhibit cell proliferation and induce cell death of the OSCC cells. But we observed that 3-MA partially reverses the antitumor effects of DHA on cell proliferation and viability in the OSCC cells, compared to DHA treatment alone (Figure S8(g)). These work support that autophagy plays a complicated dual role in the OSCC cells.

### 2.10. Combined Treatment of DHA and Mdivi-1 Can Synergistically Combat OSCCs

**Figure 10:**
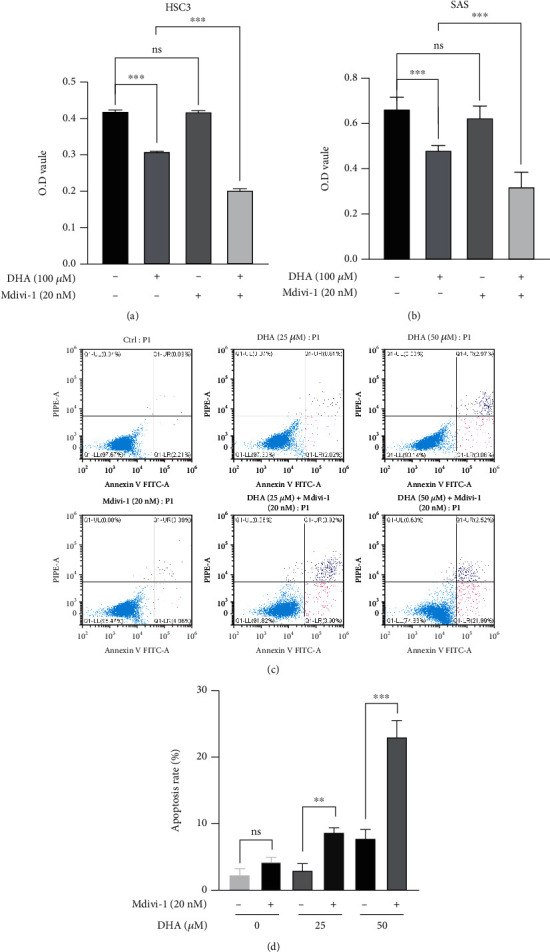
Combined treatment of DHA and Mdivi-1 can synergistically combat OSCCs.(a, b). Growth inhibition of HSC3 (a) and SAS (b) cells were measured with the indicated treatments for 48 h (*t*-test, ^∗∗∗^*p* < 0.001; means ± SE, ns, not significant, *n* = 3). (c, d). Cell apoptosis levels of the OSCC tumor cells were assessed with the indicated treatments for 24 hours, by flow cytometry analysis using Annexin V-FITC/PI dual staining (*t*-test, ^∗∗^*p* < 0.01, ^∗∗∗^*p* < 0.001; means ± SE, ns, not significant, *n* = 3).

To elucidate the role of mitophagy in the antitumor effect of DHA, our data indicated that mitophagy inhibitor Mdivi-1 could markedly aggravate DHA-induced cell death of the OSCCs through inhibition of the mitophagy pathway. CCK8 assay was carried out to detect cell viability and growth in the OSCC tumor cells. HSC3 and SAS cells were cotreated by DHA (100 *μ*M) and Mdivi-1 (20 nM) for 48 h. As indicated in Figures 10(a) and 10(b), the results have shown that DHA and Mdivi-1 combined treatment suppressed the proliferation and growth of the OSCC cells more than DHA treatment alone (DHA and Mdivi-1 combined treatment: 0.167 ± 0.05, *p* = 0.0296 for SAS cells, and 0.106 ± 0.001, *p* < 0.0001 for HSC3 cells). Furthermore, we have carried out flow cytometry analysis to evaluate cell apoptosis levels of SAS cells with single and combined treatment of DHA and Mdivi-1 for 24 h, using Annexin V-FITC/PI dual staining. The data indicated that cotreatment of DHA and Mdivi-1 synergistically induced more cell apoptosis events than DHA (Figures 10(c) and 10(d)). The data strongly support that mitophagy can protect OSCC tumor cells from DHA-induced mitochondrial stress, and also mitophagy inhibitor can reinforce the antitumor activity of DHA on OSCC cell proliferation and survival.

## 3. Discussion

OSCC patients, with tumor metastasis and recurrence, have a poor prognosis in general. Therefore, a significant renovation of therapeutic interventions is an urgent demand for better treatment outcomes for OSCC tumors. Our recent studies have reported that squamous cell carcinomas have a partial loss of ribosomal DNA copy number frequently, and are sensitive to DNA damage-inducing agents [53–55]. In our study, we explored the anticancer activity and underlying mechanism of DHA on OSCCs. The results illuminated that DHA remarkably suppressed OSCC tumor cell growth and proliferation, and single-cell colony formation. In addition, DHA induced cell apoptosis in the caspase 3-dependent and independent manner, associated with impairment of mitochondrial structure and function. Also, treatment of DHA released mitochondrial AIF into the nucleus for inducing DNA damage. Moreover, DHA administration robustly inhibited OSCC tumor growth and survival *in vivo* of tumor-bearing mice associated with few adverse effect. Furthermore, DHA remarkably induced mitochondrial injury and activation of mitophagy signaling. Suppression of mitophagy significantly reinforced the inhibitory effects of DHA to OSCC tumor cell proliferation and survival.

DHA has been successfully applied for the clinical treatment of malaria for over 30 years. Orally administered DHA can be rapidly absorbed in the gastrointestinal tract, with Cmax reached at roughly 1–2 h after administration. Hence DHA has some strengths as an antitumor agent including well biosafety and toleration, less adverse effect on normal tissues, low cross-drug resistance, and synergistic effects with resection or chemotherapy and/or radiation [56–58]. Increasing studies have reported that DHA could exert its antitumor activity through various intracellular pathways such as ROS generation, induction of cell apoptosis, DNA damage and repair, ferroptosis, suppression of tumor metastasis, angiogenesis, and inhibition of prooncogenic signalings [22, 59]. For instance, Qin et al. reported that DHA can induce the decrease of mitochondrial membrane potential, cytochrome c release, caspase activation, and phosphatidylserine externalization in hepatocellular carcinoma cells [60]. And a few studies also demonstrated that DHA can modulate several signaling pathways to trigger caspase-dependent and -independent apoptosis [61–63]. In line with these previous studies, our data have revealed that DHA triggered the protein expression of cleaved caspase-3 in the OSCC tumor cells dosage-dependently. Also, DHA markedly induced mitochondrial c-type cytochrome releasing into the cytoplasm, resulting in activation of cytoplasmic caspase-3. The data have indicated that DHA induced OSCC cell apoptosis in a caspase 3-dependent manner. In addition, we observed that treatment of DHA remarkably upregulated mitochondrial AIF protein expression, and DHA strikingly induced mitochondrial AIF transporting into the nucleus and the cytosolic fractions for activation of nucleus *γ*-H2AX. AIF has been reported to serve as a caspase 3-independent death effector derived from mitochondria [64, 65]. These data revealed that DHA induced OSCC tumor cell death in the caspase 3-dependent and -independent manner. Collectively, we found that DHA remarkably impaired the mitochondrial membrane and its metabolic function, released cytochrome c and AIF, and induced nucleus DNA damage and mitophagy. Some of the factors can trigger the mitophagy pathways, such as nutrient deprivation, oncogene activation, hypoxia, mitochondrial damage, and neurodegenerative conditions [66, 67]. This study firstly demonstrated that the DHA-activated mitophagy pathway can protect the tumor cells from DHA-inhibited cell proliferation and survival.

Currently, Cetuximab, a chimeric human-murine IgG1 mAb that inhibits EGFR, is the only targeted therapy for OSCC tumors [5, 6]. However, the success rate of monotherapy of Cetuximab is very mild, ranging from 10 to 13% [6]. Several oral EGFR tyrosine kinase inhibitors, including Gefitinib and Erlotinib, which are clinically used to treat nonsmall cell lung cancer, have been evaluated in clinical trials in OSCC diseases. But the drug is not more effective than the present chemotherapeutic drugs so far [6]. Cisplatin treatment is still the most common therapeutic regimen for OSCC tumors. In our work, we have found that DHA has not any toxic effect on NOK, but cisplatin is highly toxic to normal oral cells. It should be noted that DHA could reduce the toxic effect of cisplatin in NOK cells with cotreatment of DHA and cisplatin. In the OSCC tumor cells, both DHA and cisplatin induced a similar apoptotic rate. The cytotoxic effects of DHA and cisplatin treatments on the NOK and OSCC cells were assessed by CCK-8 assay. The results have indicated that OSCC tumor cells are much more sensitive to the antiproliferative effects of DHA and cisplatin treatments than NOK cells, which are well consistent with the Annexin V/PI dual staining. A recent study has reported that DHA could significantly reduce the effective concentrations of cisplatin to optimize its antitumor activity. Moreover, DHA could not only reduce the tumor growth but also partly alleviated the toxicity of cisplatin in the tumor-xenograft mice model [68]. In addition, a recent study also reported that DHA-based therapeutics exhibit a preferential antitumor effect on human melanoma and pancreatic cancer cells significantly, rather than normal human fibroblasts [69]. The level of intracellular free iron can serve as a criterion to determine which type of cancer is more sensitive to ferroptosis [70]. Yang et al. previously reported that mutant RAS signaling controls iron metabolism genes to promote the cellular iron reservoir [71]. Increasing studies documented that OSCC tumors have frequent gene mutations of RAS oncogenic pathways [72–74], and OSCCs may belong to iron-rich tumors. Given this evidence, OSCC may be more susceptible to the combined treatment of DHA and cisplatin-promoted ferroptosis preferentially than normal cells. Thus, DHA may be used in combination with cisplatin chemotherapy for primary OSCC tumors, associated with fewer adverse effect. To be noted, although our study indicated that DHA inhibits cell proliferation and survival of OSCCs, it is strongly recommended to treat OSCC tumors at the early cancer stages. Especially, using DHA to treat OSCC patients before the tumor metastasis may give more benefits to suppress further tumor spreading or rapid recurrence.

Many chemotherapy-resistant tumors including specific OSCC have demonstrated autophagy to escape cell death. In other tumors, autophagy impedes tumorigenesis by inhibiting cancer cells' survival and inducing cell death, but it also facilitates tumorigenesis by improving tumor growth. In this study, we used 3-MA to preincubate for 2 h, and then treat the OSCC tumor cells with DHA for 24 h (Figures S8(e)–(g)). 3-MA did not affect basal autophagy level, and 3-MA partially blunted DHA-induced upregulation of LC3-II and p62 protein in the OSCC tumor cells. For CCK8 assay (Figure S8(g)), 3-MA can mildly inhibit cell proliferation and viability of the OSCC. Also, we observed that 3-MA partially alleviated the effect of DHA on cell viability in the OSCC cells, compared to DHA treatment alone. Herein we used 3-MA to determine the level of basal autophagy flux up/downregulations in OSCC tumor cells. The effect of 3-MA is quite different from Midiv-1. Although 3-MA can mildly impair the OSCC cell proliferation and viability, 3-MA partly blunted the antitumor effect of DHA on the OSCC cells associated with its inhibition of autophagosome formation. Moreover, Midiv-1 displayed little cytotoxicity for the OSCC cells, but cotreatment of DHA and Midiv-1 could synergistically trigger OSCC cell death. The autophagy/lysosomal inhibitor are reported to impede the growth and survival of OSCC tumors by some previous studies [75–79]. Taken together, our study supported that autophagy possesses complicated dual effects in the cell death and apoptosis of the OSCC cells. Furthermore, DHA-induced mitophagy can compromise the antitumor effect of DHA in the OSCC cells.

In addition, we have analyzed various therapeutic effects of DHA on OSCC tumors, including inhibiting cell proliferation, promoting cell apoptosis and autophagy, and inducing DNA damage and mitochondrial impairments. This study is aimed at exploring the potential role of DHA in clinical treatment of OSCC tumors. Our data support that multiple pathways are involved in the antitumor effects of DHA on OSCC. Interestingly, DHA remarkably activates the mitophagy pathway of OSCC, and we found that the activation of mitophagy pathway could protect OSCC from the antitumor effects of DHA. The results may shed light on the drug resistance of OSCC in DHA-based treatments.

The high expression of dynamin-related protein (Drp1) in many tumors might be a potential target for tumor therapy. Mdivi-1 is an effective yeast mitochondrial division inhibitor. A study reports that Mdivi-1 impairs cell proliferation by restricting oxidative metabolism instead of glycolysis [80]. The effect of Mdivi-1 on oxidative metabolism is not related to Drp1 inhibition or mitochondrial fusion induction. However, the molecular connection between DHA and Drp1 remains unclear. In our work, we investigated the role of Mdivi-1, a small molecule inhibitor of Drp1, in combination with DHA in the mitochondrial autophagy. The results display that Mdivi-1 blunts DHA-induced mitophagy in the OSCCs. Furthermore, combined treatment of DHA and Mdivi-1 synergistically suppress cell proliferation and triggers cell death of OSCC tumor cells. Overall, these data reveal that inhibition of Drp1 with Mdivi-1 can reinforce some anticancer effects of DHA by eliminating DHA-induced activation of mitophagy, and aggravating DHA-impaired mitochondrial structure and function.

In conclusion, we provided evidence that DHA significantly suppressed cell proliferation and triggered cell death *in vitro* and *in vivo* of OSCC through targeting mitochondria-associated pathways, with few adverse effects on normal tissues/oral cells. The anticancer effects of DHA may be neutralized by the mitophagy pathway upon mitochondria dysfunction. Therefore, inhibition of the subsequent mitophagy pathway with Mdivi-1 can enhance the anticancer activities of DHA on OSCC tumor cells. Our study strongly supports that a DHA-based treatment strategy may be used as a new therapeutic option for OSCC disorder.

## 4. Materials and Methods

### 4.1. Cell Culture and Reagents

Human oral normal and cancer cell lines, including normal oral keratinocyte (NOK), SAS, and HSC3, were acquired from the American Type Culture Collection (ATCC) and Guangdong Provincial Key Laboratory of Stomatology, Guanghua School of Stomatology, Hospital of Stomatology at Sun Yat-sen University. The cells were cultured in DMEM medium (HyClone, Logan, UT, USA) with 10% fetal bovine serum (FBS) (HyClone, Logan, UT, USA), containing 100 U of penicillin G/mL and 100 *μ*g of streptomycin/mL (Sigma-Aldrich, USA) at 37°C in a humidified atmosphere of 5% CO_2_.

Dihydroartemisinin (DHA, Hy-N0176), and Mdivi-1 (Hy-15886) compounds were purchased from Med Chem Express company (San Diego, CA, USA). N-acetyl-l-cysteine (NAC) was purchased from the Beyotime Institute of Biotechnology. 3-Methyladenine (3-MA, S2767), and cisplatin (CDDP, #S1166) compounds were purchased from Selleck company. DHA and 3-MA were stored as 10 mM stock solutions in DMSO (vehicle) for *in vitro* assay. CDDP and NAC were dissolved in 10 mM PBS buffer. DHA was administered every 3 days at 5 mg/kg in PBS buffer for the *in vivo* study.

### 4.2. Cell Proliferation Assay

Proliferation inhibition of NOK and OSCC tumor cells were evaluated by the CCK-8 assay. The cancer cells were seeded into 96-well plates. After 24 hours, the medium was added by the vehicle or compounds. We incubated the cells at 37°C for 48 and 72 hours. Subsequently, we added 100 *μ*L prepared CCK-8 working solution (Dojindo Laboratories, Kumamoto, Japan) into each well. And then, we measured the luminescence by a microplate reader at 450 nm after 1 hour incubation.

### 4.3. Cell Apoptosis Assay

Flow cytometry analysis was conducted to gauge cell apoptosis levels. We cultured the OSCC and NOK cells in the six-well plates. One day later, the cells were incubated with the indicated treatment for 48 hours. Subsequently, we collected the cells in PBS buffer. Then, the dual staining of annexin V-FITC and propidium iodide (PI) was performed according to the manufacturer's protocol (BD Biosciences, Franklin Lakes, NJ, USA). We analyzed the samples using flow cytometry within 1 hour.

### 4.4. Cell Cycle Analysis

The OSCC cells were washed with PBS and centrifuged to collect, then cells were fixed with ice-cold 70% ethanol for overnight incubation at 4°C. After one day, we incubated the cell pellets with 1 mL PI (BD Biosciences, Franklin Lakes, NJ, USA) solution (50 *μ*g/mL PI and 100 *μ*g/mL RNaseA) for 30 min at 37°C. The data were assessed by flow cytometry within an hour and analyzed by FlowJo V10 software to measure the cell cycle phases.

### 4.5. Single-Cell Colony Formation Assay

The OSCC cells were plated into six-well plates at a density of 100 cells per well. After 24 h culture, the cells were incubated with the indicated dosages of DHA in the complete medium for 3 days. 10 days later, we fixed and stained the single-cell colonies with 2 mg/mL methylene blue dissolved in methanol (0.5%). The result reflected the sensitivity of the OSCC cells for the DHA treatments.

### 4.6. Immunofluorescence Staining

The OSCC cells were plated into confocal dishes. After fixation and permeation, the OSCC cells were blocked in 5% nonfat milk and washed with PBS for three times. Then the cells were incubated with antibody overnight at 4°C. The cells were washed and incubated with the secondary antibody the next day. Sequentially, OSCC cells were washed and counter-stained with 4′6-diamidino-2-phenylindole (DAPI) staining. The data were obtained using the laser scanning confocal microscopy. To be noted, *γ*-H2AX, an early cellular response to the induction of DNA double-strand breaks [81], is phosphorylation of the Ser-139 residue of the histone variant H2AX. Thus, the antibody of phospho-Histone H2AX (Ser139) is the antibody of *γ*-H2AX as well (Table S5).

### 4.7. Quantification of Intracellular ROS Generation

The OSCC cells were treated with various concentrations of DHA 1 mM dihydroethidium (DHC) bromide (MCE company, HY-D0079) for 12 h. Then the cells were incubated with a 10 *μ*M DCFH-DA (Beyotime Institute of Biotechnology, Nanjing, China, #S0033S) working solution for 20 min in the darkness at 37°C. Sequentially, the cells were collected, washed, and resuspended in serum-free DMEM. The intracellular ROS production was tested by flow cytometry.

### 4.8. Western Blotting Analysis

For each western blotting analysis, the OSCC and NOK cells were lysed in precooled RIPA buffer containing a phosphatase inhibitor cocktail and a 1% proteinase inhibitor. The supernatants were collected after the lysed samples were centrifuged at 15000 rpm at 4°C for 30 min. After determining the protein concentration with the Pierce BCA Protein Assay Kit (Bio-Rad, Richmond, CA, USA), the mixture containing the sample and 4 × loading buffer was heated to 100°C for 5 min. The lysates were separated by SDS-PAGE and transferred to polyvinylidene fluoride (PVDF) membranes (Bio-Rad, Hercules, CA), which were subsequently blocked with 5% milk for one hour at room temperature, and then incubated overnight at 4°C with suitably diluted the primary antibodies indicated. The PVDF membranes were incubated with HRP-labeled secondary antibodies at room temperature for one hour. Subsequently, the ECL Plus Western Blotting Detection System (Amersham Biosciences, Piscataway, NJ, USA) was used for chemiluminescent visualization. The information of all antibodies were provided in Table S4. The antibody validation statements/sources were provided in Table S5.

### 4.9. Determination of Δ*ψm* Using JC-1 Staining

Changes in mitochondrial membrane potential (Δ*ψm*) of OSCC cells were gauged using a JC-1 (5,5′,6,6′-tetrachloro-1,1′,3,3′-tetraethylbenzymidazolyl carbocyanine iodide) kit (Beyotime Institute of Biotechnology, Jiangsu, China, #C2006). After treatment with 30 *μ*M DHA for 48 h, 2 × 10^6^ cells were harvested and incubated with JC-1 at 37°C for 20 min and then washed and resuspended in PBS. 10,000 events were acquired with the flow cytometer to analyze samples. JC-1 exists in the form of monomers in low Δ*ψm* (Ex = 549 nm, Em = 590 nm), while in the form of protomeric in high Δ*ψm* (Ex = 488 nm, Em = 530 nm). The loss of Δ*ψm* was rejected by increased green fluorescence from the JC-1 monomers.

### 4.10. Quantification of Intracellular ATP Biosynthesis

The SAS cells were incubated in a complete DMEM medium without or with DHA for 24 h. 100 *μ*L ATP detection buffer (Beyotime Institute of Biotechnology, Nanjing, China, #S0026) was added to samples, and the relative light unit (RLU) of the supernatant was gauged using a microplate reader. A standard curve was established with an ATP standard solution in a 96-well plate according to the guideline.

### 4.11. Quantification of Mitochondrial Ca^2+^ Levels

The level of intracellular calcium was evaluated by using the fluorescent dye Fluo-3AM (Beyotime Institute of Biotechnology, Nanjing, China, #S1056). Briefly, the OSCC tumor cells were seeded and treated with various concentrations of DHA for 12 h. After collection and washing, cells were resuspended in Fluo-3 AM (50 *μ*M) for 60 min in darkness at 37°C. After washing twice with PBS, the fluorescent signals reflecting the Ca^2+^ level were detected by flow cytometry.

### 4.12. Mito-Red Staining

The OSCC tumor cells were seeded in laser confocal dishes (20 mm in diameter) at 1 × 10^4^ cells per dish. After indicated treatment with DHA, the cells were washed twice and incubated in the medium containing 100 nM mitotracker Red (ABP Biosciences, #C042) for mitochondrial staining and Hoechst 33342 (Beyotime Institute of Biotechnology, Jiangsu, China #C1027) for nucleus staining for 1 hour at 37°C. After washing with medium, the cells were observed using laser confocal microscopy.

### 4.13. Mitophagy Assay

The cells of the appropriate density were washed with serum-free DMEM twice and incubated in 100 nM Mitophagy Dye working solution (ABP Biosciences, #MD01) at 37°C for 30 min. Subsequently, the cells were washed and cultured in 1 *μ*M Lyso Dye working solution at 37°C for 30 min. After washing, the cells were incubated with nuclear dye for 10 min. Then the fluorescence images were obtained by confocal microscopy.

### 4.14. Isolation of Mitochondria, Cytoplasm, and Nucleus Fragments of the OSCC Cells

The OSCC cells were scraped and centrifuged to collect 1 × 10^6^ cells. The protein of nuclear and cytoplasmic fragments were prepared using the Nucleus and Cytoplasmic Protein Extraction Kit (Beyotime Institute of Biotechnology, Jiangsu, China, #P0027). The cells were lysed in 200 *μ*L cytoplasmic protein extraction buffer A containing 10 *μ*L PMSF. Following a violent vortex, the obtained lysates were incubated for 15 min on ice and added with 10 *μ*L cytoplasmic protein extraction buffer B. After the 5 sec vortex, the lysates were centrifuged at 12000 × g for 5 min at 4°C. The supernatant, consisting of the cytoplasmic fraction, was aliquoted for analysis. The resulting precipitation was resuspended in 50 *μ*L nucleus extraction buffer on ice and the lysates swirled every 2 min for 30 min. The supernatant was used as nucleic fractions following centrifuge at 12000 × g for 10 min.

### 4.15. Lipid Hydroperoxide (LPO) Assay

According to the ratio of tissue mass to extract liquid volume (mL) of 1 : 5 ~ 10, the ice bath homogenization was carried out. The supernatant was centrifuged at 8000 × g, 4°C for 10 min and put on ice. The supernatant and the solution were mixed according to the assay kit (Nanjing Jiancheng, #A106-1), and were added into a new tube. After vortex and incubation at 95°C for 40 min, the sample was centrifuged at 3000 × g for 10 min. Subsequently, the 200 *μ*L of the supernatant was added to a 96-well plate and the optical density (OD) value was determined at a wavelength of 586 nm. Then the LPO content is calculated by the formula.

### 4.16. Bioinformatic Analysis of OSCC Transcriptomics from TCGA Database

We downloaded OSCC RNA-seq datasets from the Cancer Genome Atlas (TCGA) public database (http://portal.gdc.cancer.gov/). There are 140 samples including 127 OSCC tumor samples and 13 adjacent normal tissues from GSE62944. Based on these RNA-Seq datasets, differential gene expression analysis between OSCC samples and the adjacent normal tissues was performed using the Bioconductor package DESeq2. The *P* values were adjusted using Benjamini and Hochberg's method to control the false discovery rate. Genes with an adjusted *P* value <0.05 found by DESeq2 were specified as differential expressions. Gene ontology (GO) functional enrichment analysis was performed using a cluster Profile, in which gene length bias was corrected. GO terms with corrected *P* value less than 0.05 were considered significantly enriched by differential expressed genes. Gene expression data was downloaded from GSE62944. We kept genes with the gene expression value (FPKM) larger than 1 in at least five samples for further analysis. 159 genes were found to be significantly differentially expressed between the OSCC tumor and the adjacent normal samples using the R package edgeR with FDR < = 0.01, fold change > = 2, and the average gene expression > = 2. Herein we focus on gene expression analysis of mitochondrial structure and function associated with OSCC development and progression.

### 4.17. Animal Experimentation Ethics

In the study, all of the animal experiments were conducted based on the protocols approved by the Sun Yat-sen University Animal Ethics Committee, Sun Yat-sen University (Guangzhou, Guangdong Province, China).

### 4.18. *In Vivo* Animal Study

A total of 20 BALB/c nude mice (four-week-old) were purchased from Zhejiang Vital River Laboratory Animal Technology Co., Ltd., and raised under the specific pathogen-free (SPF) conditions. The OSCC tumor-xenograft mice model was established by subcutaneously injection of 1 × 10^6^ SAS cells (suspended in 100 *μ*L of PBS), and one week later, the tumor-bearing mice were randomly divided into two groups. The control group was injected with PBS buffer, another group was injected with DHA within PBS buffer every 3 days. In the study, we used the subcutaneous injection of DHA or the matched vesicle (PBS buffer) into the OSCC tumor-xenograft mice model. After 27 days of the administration, all tumor specimens were collected. The OSCC tumor growth was monitored using a caliper, and the tumor volume was calculated according to the formula *V* = *π* × maximal diameter × perpendicular diameter 2/6. The OSCC tumor growth curves and tumor volume were established.

### 4.19. Tissue Immunostaining

After dehydration and embedding of OSCC tumor tissues, it was cut into a thickness of 4 *μ*m using a pathological slicer (LEICA RM2145, Germany). After the sections have dried, we performed the terminal deoxynucleotidyl transferase-mediated dUTP nick end-labeling (TUNEL) fluorescence staining and imaged the slices using a fluorescence inversion microscope system (Zeiss, Germany).

### 4.20. Statistical Analysis

In the study, the IBM SPSS 20.0 statistical software was used for further statistical analysis. We conducted *t*-test to analyze the differences between the drug treated experimental group and the blank control group. *In vitro* experiments were conducted in three independent times, and presented as a summary of the mean of replicates with SD, unless otherwise noted. For *in vivo* study, histological quantitation of animal tumor tissues was conducted by an Aperio ScanScope digital scanner and viewed by ImageScope software (Leica Biosystems). All values are presented as the mean ± standard deviation (SD). *s* < 0.05 was considered to be statistically significant. The quantitative data represented the mean of three biological replicates.

## Data Availability

All original data in the study are available from the corresponding author upon a reasonable request.

## Supplementary Materials

Supplementary 1 Supplementary Figure 1: the heat map data showed that 159 genes were found to be significantly differentially expressed between tumor and normal samples using the R package edgeR with FDR < = 0.01, fold change > = 2, and the average gene expression > = 2.

Supplementary 2 Supplementary Figure 2: the boxplots showed the genes are significantly upregulated at each stage of OSCC tumor progression.

Supplementary 3 Supplementary Figure 3: expression and phosphorylation of mTOR and S6 were detected by western blot and normalized to mTOR and S6 (*t*-test, ^∗∗^*P* < 0.01; means ± SE, ns, not significant, *n* = 3).

Supplementary 4 Supplementary Figure 4: (a) quantification of Bcl2, Bax, and Cleaved Caspase-3 protein expression in HSC3 of the indicated treatment (*t*-test, ^∗^*P* < 0.5, ^∗∗^*P* < 0.01; means ± SE, ns, not significant, *n* = 3). (b) The level of cytochrome C (Cyt-c) was assessed in mitochondrial fractions and cytosolic fractions (*t*-test, ^∗^*P* < 0.5, ^∗∗^*P* < 0.01; means ± SE, ns, not significant, *n* = 3).

Supplementary 5 Supplementary Figure 5: (a) NOK cells were treated with DHA, CDDP, and the combination (DHA + CDDP) for 48 hours. Cell apoptosis was measured with Annexin V-FITC/PI dual staining by flow cytometry analysis. (b) HSC3 OSCC tumor cells were treated with DHA, CDDP, and the combination (DHA + CDDP) for 48 hours. Cell apoptosis was measured with Annexin V-FITC/PI dual staining by flow cytometry analysis.

Supplementary 6 Supplementary Figure 6: OSCC HSC3 tumor cells were treated by DHA for 48 h with or without pretreatment of NAC. Cell apoptosis was measured with Annexin V-FITC/PI dual staining by flow cytometry analysis.

Supplementary 7 Supplementary Figure 7: DHA induced DNA damage. (a) Average fluorescence dot per nucleus of *γ*-H2AX was analyzed in the OSCC cells (*t*-test, ^∗∗∗∗^*P* < 0.0001; means ± SE, ns, not significant, *n* = 3). (b) Protein expression level of *γ*-H2AX was determind (*t*-test, ^∗^*P* < 0.5, ^∗∗^*P* < 0.01; means ± SE, ns, not significant, *n* = 3). (c) Statistical analysis of AIF average fluorescence area in nucleus (*t*-test, ^∗∗^*P* < 0.01; means ± SE, ns, not significant, *n* = 3). (d) The mitochondrial loading control COX IV was used to validate the purity of mitochondria part by Western blotting analysis. (e) The expression of COX IV was quantified in mitochondrial and cytoplasm (*t*-test, ^∗∗∗∗^*P* < 0.0001; means ± SE, ns, not significant, *n* = 3). (f) Quantification of the level of AIF in mitochondrial,cytoplasm, and nucleus (*t*-test, ^∗^*P* < 0.5, ^∗∗^*P* < 0.01; means ± SE, ns, not significant, *n* = 3). (g) Relative protein expression of *γ*-H2AX was, respectively, assessed mitochondria part, cytoplasm part, and cell nucleus (*t*-test, ^∗^*P* < 0.5, ^∗∗^*P* < 0.01; means ± SE, ns, not significant, *n* = 3).

Supplementary 8 Supplementary Figure 8: the effect of 3-MA on DHA action in the OSCC cells. (a) Relative protein expression of LC3-II, Beclin1, and Mitofusion-1 were determined to assess mitophagy (*t*-test, ^∗^*P* < 0.5, ^∗∗^*P* < 0.01; means ± SE, ns, not significant, *n* = 3). (b) The level of Mitofusion-1 was analyzed to evaluate the mitophagy of OSCC cells with treatment of DHA and Mdivi-1 (*t*-test, ^∗^*P* < 0.5; means ± SE, ns, not significant, *n* = 3). (c) The protein level of LC3-II, PINK1, and p62 was assessed and nomalized to *β*-Tubulin (*t*-test, ^∗^*P* < 0.5, ^∗∗^*P* < 0.01; means ± SE, ns, not significant, *n* = 3). (d) Autophage asscociated protein expression of NOK cells was detected (*t*-test, means ± SE, ns, not significant, *n* = 3). (e) HSC3 cells were preincubated with 3-MA to preincubate for 2 h and then treated with or without DHA for 24 h for Western blot analysis. (f) HSC3 cells were preincubated with 3-MA to preincubate for 2 h and then treated with or without DHA for 48 h for Western blotting. (g) Statistical analysis of proliferation inhibition of cells was assessed with the indicated treatmen (*t*-test, ^∗^*P* < 0.5, ^∗∗^*P* < 0.01; means ± SE, ns, not significant, *n* = 3).

Supplementary 9 Supplementary Table 1: clinical OSCC sample_sheet from TCGA public database.

Supplementary 10 Supplementary Table 2: clinical information of the OSCC patient from TCGA public database.

Supplementary 11 Supplementary Table 3: list of differential gene expression in OSCC tumor samples compared to the adjacent normal tissue samples.

Supplementary 12 Supplementary Table 4: list of all antibodies in the study.

Supplementary 13 Supplementary Table 5: antibody validation statements and sources in the study.

## References

[1] Durr M. L., Li D., Wang S. J. (2013). Oral cavity squamous cell carcinoma in never smokers: analysis of clinicopathologic characteristics and survival. *American Journal of Otolaryngology*.

[2] Vigneswaran N., Williams M. D. (2014). Epidemiologic trends in head and neck cancer and aids in diagnosis. *Oral and Maxillofacial Surgery Clinics of North America*.

[3] Adelstein D. J., Li Y., Adams G. L. (2003). An intergroup phase III comparison of standard radiation therapy and two schedules of concurrent chemoradiotherapy in patients with unresectable squamous cell head and neck cancer. *Journal of Clinical Oncology*.

[4] Posner M. R., Haddad R. I., Wirth L. (2004). Induction chemotherapy in locally advanced squamous cell cancer of the head and neck: evolution of the sequential treatment approach. *Seminars in Oncology*.

[5] Cohen R. B. (2014). Current challenges and clinical investigations of epidermal growth factor receptor (EGFR)- and ErbB family-targeted agents in the treatment of head and neck squamous cell carcinoma (HNSCC). *Cancer Treatment Reviews*.

[6] Sacco A. G., Worden F. P. (2016). Molecularly targeted therapy for the treatment of head and neck cancer: a review of the ErbB family inhibitors. *Oncotargets and Therapy*.

[7] Rodriguez C. P., Adelstein D. J., Rybicki L. A. (2012). Single-arm phase II study of multiagent concurrent chemoradiotherapy and gefitinib in locoregionally advanced squamous cell carcinoma of the head and neck. *Head & Neck*.

[8] Martins R. G., Parvathaneni U., Bauman J. E. (2013). Cisplatin and radiotherapy with or without erlotinib in locally advanced squamous cell carcinoma of the head and neck: a randomized phase II trial. *Journal of Clinical Oncology*.

[9] Ferris R. L. (2015). Immunology and immunotherapy of head and neck cancer. *Journal of Clinical Oncology*.

[10] Ferris R. L., Blumenschein G., Fayette J. (2016). Nivolumab for recurrent squamous-cell carcinoma of the head and neck. *The New England Journal of Medicine*.

[11] Economopoulou P., Perisanidis C., Giotakis E. I., Psyrri A. (2016). The emerging role of immunotherapy in head and neck squamous cell carcinoma (HNSCC): anti-tumor immunity and clinical applications. *Ann Transl Med*.

[12] Li Y. (2012). Qinghaosu (artemisinin): chemistry and pharmacology. *Acta Pharmacologica Sinica*.

[13] Fairhurst R. M., Nayyar G. M. L., Breman J. G. (2012). Artemisinin-resistant malaria: research challenges, opportunities, and public health implications. *The American Journal of Tropical Medicine and Hygiene*.

[14] Li Q. G., Peggins J. O., Fleckenstein L. L., Masonic K., Heiffer M. H., Brewer T. G. (1998). The pharmacokinetics and bioavailability of dihydroartemisinin, arteether, artemether, artesunic acid and artelinic acid in rats. *The Journal of Pharmacy and Pharmacology*.

[15] Sun W. C., Han J. X., Yang W. Y., Deng D. A., Yue X. F. (1992). Antitumor activities of 4 derivatives of artemisic acid and artemisinin B in vitro. *Zhongguo Yao Li Xue Bao*.

[16] Odaka Y., Xu B., Luo Y. (2014). Dihydroartemisinin inhibits the mammalian target of rapamycin-mediated signaling pathways in tumor cells. *Carcinogenesis*.

[17] Luo J., Odaka Y., Huang Z. (2021). Dihydroartemisinin inhibits mTORC1 signaling by activating the AMPK pathway in rhabdomyosarcoma tumor cells. *Cell*.

[18] Chen G. Q., Benthani F. A., Wu J., Liang D., Bian Z. X., Jiang X. (2020). Artemisinin compounds sensitize cancer cells to ferroptosis by regulating iron homeostasis. *Cell Death and Differentiation*.

[19] Lin R., Zhang Z., Chen L. (2016). Dihydroartemisinin (DHA) induces ferroptosis and causes cell cycle arrest in head and neck carcinoma cells. *Cancer Letters*.

[20] Wang T., Luo R., Li W. (2020). Dihydroartemisinin suppresses bladder cancer cell invasion and migration by regulating KDM3A and p21. *Journal of Cancer*.

[21] Zhang F., Ma Q., Xu Z. (2017). Dihydroartemisinin inhibits TCTP-dependent metastasis in gallbladder cancer. *Journal of Experimental & Clinical Cancer Research*.

[22] Chen Y., Mi Y., Zhang X. (2019). Dihydroartemisinin-induced unfolded protein response feedback attenuates ferroptosis via PERK/ATF4/HSPA5 pathway in glioma cells. *Journal of Experimental & Clinical Cancer Research*.

[23] Li S., Huang P., Gan J. (2019). Dihydroartemisinin represses esophageal cancer glycolysis by down-regulating pyruvate kinase M2. *European Journal of Pharmacology*.

[24] Crespo-Ortiz M. P., Wei M. Q. (2012). Antitumor activity of artemisinin and its derivatives: from a well-known antimalarial agent to a potential anticancer drug. *Journal of Biomedicine & Biotechnology*.

[25] Elhassanny A. E. M., Soliman E., Marie M. (2020). Heme-dependent ER stress apoptosis: a mechanism for the selective toxicity of the dihydroartemisinin, NSC735847, in colorectal cancer cells. *Frontiers in Oncology*.

[26] Chen Y., Li R., Zhu Y. (2020). Dihydroartemisinin induces growth arrest and overcomes dexamethasone resistance in multiple myeloma. *Frontiers in Oncology*.

[27] Zhou Q., Ye F., Qiu J. (2022). Dihydroartemisinin induces ER stress-mediated apoptosis in human tongue squamous carcinoma by regulating ROS production. *Anti-Cancer Agents in Medicinal Chemistry*.

[28] Zheng S., Wu R., Deng Y., Zhang Q. (2022). Dihydroartemisinin represses oral squamous cell carcinoma progression through downregulating mitochondrial calcium uniporter. *Bioengineered*.

[29] Liu L., Liao X., Wu H., Li Y., Zhu Y., Chen Q. (2020). Mitophagy and its contribution to metabolic and aging-associated disorders. *Antioxidants & Redox Signaling*.

[30] Laleve A., Panozzo C., Kühl I. (2020). Artemisinin and its derivatives target mitochondrial *c* -type cytochromes in yeast and human cells. *Biochimica et Biophysica Acta (BBA)-Molecular Cell Research*.

[31] Du J., Wang T., Li Y. (2019). DHA inhibits proliferation and induces ferroptosis of leukemia cells through autophagy dependent degradation of ferritin. *Free Radical Biology & Medicine*.

[32] Poupel F., Aghaei M., Movahedian A., Jafari S. M., Shahrestanaki M. K. (2017). Dihydroartemisinin induces apoptosis in human bladder cancer cell lines through reactive oxygen species, mitochondrial membrane potential, and cytochrome C pathway. *International Journal of Preventive Medicine*.

[33] Dou C., Ding N., Xing J. (2016). Dihydroartemisinin attenuates lipopolysaccharide-induced osteoclastogenesis and bone loss via the mitochondria-dependent apoptosis pathway. *Cell Death & Disease*.

[34] Chaib I., Cai X., Llige D. (2019). Osimertinib and dihydroartemisinin: a novel drug combination targeting head and neck squamous cell carcinoma. *Ann Transl Med*.

[35] Nam W., Tak J., Ryu J. K. (2007). Effects of artemisinin and its derivatives on growth inhibition and apoptosis of oral cancer cells. *Head & Neck*.

[36] Hou J., Wang D., Zhang R., Wang H. (2008). Experimental therapy of hepatoma with artemisinin and its derivatives: in vitro and in vivo activity, chemosensitization, and mechanisms of action. *Clinical Cancer Research*.

[37] Wang X., Song W., Zhang F., Huang R. (2021). Dihydroartemisinin inhibits TGF-*β*-induced fibrosis in human tenon fibroblasts via inducing autophagy. *Drug Design, Development and Therapy*.

[38] Yi Y. C., Liang R., Chen X. Y. (2021). Dihydroartemisinin suppresses the tumorigenesis and cycle progression of colorectal cancer by targeting CDK1/CCNB1/PLK1 signaling. *Frontiers in Oncology*.

[39] Zhu W., Li Y., Zhao D. (2019). Dihydroartemisinin suppresses glycolysis of LNCaP cells by inhibiting PI3K/AKT pathway and downregulating HIF-1*α* expression. *Life Sciences*.

[40] Ji Y., Zhang Y. C., Pei L. B., Shi L. L., Yan J. L., Ma X. H. (2011). Anti-tumor effects of dihydroartemisinin on human osteosarcoma. *Molecular and Cellular Biochemistry*.

[41] Liang R., Chen W., Chen X. Y., Fan H. N., Zhang J., Zhu J. S. (2021). Dihydroartemisinin inhibits the tumorigenesis and invasion of gastric cancer by regulating STAT1/KDR/MMP9 and P53/BCL2L1/CASP3/7 pathways. *Pathology, Research and Practice*.

[42] Cai X., Miao J., Sun R. (2021). Dihydroartemisinin overcomes the resistance to osimertinib in EGFR-mutant non- small-cell lung cancer. *Pharmacological Research*.

[43] Wang J., Zhang B., Guo Y. (2008). Artemisinin inhibits tumor lymphangiogenesis by suppression of vascular endothelial growth factor C. *Pharmacology*.

[44] Zhou B., Tian R. (2018). Mitochondrial dysfunction in pathophysiology of heart failure. *The Journal of Clinical Investigation*.

[45] Sinha K., Das J., Pal P. B., Sil P. C. (2013). Oxidative stress: the mitochondria-dependent and mitochondria-independent pathways of apoptosis. *Archives of Toxicology*.

[46] Bano D., Prehn J. H. M. (2018). Apoptosis-inducing factor (AIF) in physiology and disease: the tale of a repented natural born killer. *eBioMedicine*.

[47] Delavallée L., Cabon L., Galán-Malo P., Lorenzo H. K., Susin S. A. (2011). AIF-mediated caspase-independent necroptosis: a new chance for targeted therapeutics. *IUBMB Life*.

[48] Fei W., Chen D., Tang H. (2020). Targeted GSH-exhausting and hydroxyl radical self-producing manganese-silica nanomissiles for MRI guided ferroptotic cancer therapy. *Nanoscale*.

[49] Rodriguez-Enriquez S., Kim I., Currin R. T., Lemasters J. J. (2006). Tracker dyes to probe mitochondrial autophagy (mitophagy) in rat hepatocytes. *Autophagy*.

[50] Lemasters J. J., Qian T., He L. (2002). Role of mitochondrial inner membrane permeabilization in necrotic cell death, apoptosis, and autophagy. *Antioxidants & Redox Signaling*.

[51] Gegg M. E., Cooper J. M., Chau K. Y., Rojo M., Schapira A. H., Taanman J. W. (2010). Mitofusin 1 and mitofusin 2 are ubiquitinated in a PINK1/Parkin-dependent manner upon induction of mitophagy. *Human Molecular Genetics*.

[52] Eiyama A., Okamoto K. (2015). PINK1/Parkin-mediated mitophagy in mammalian cells. *Current Opinion in Cell Biology*.

[53] Shi S., Luo H., Wang L. (2021). Combined inhibition of RNA polymerase I and mTORC1/2 synergize to combat oral squamous cell carcinoma. *Biomedicine & Pharmacotherapy*.

[54] Xu B., Li H., Perry J. M. (2017). Ribosomal DNA copy number loss and sequence variation in cancer. *PLoS Genetics*.

[55] de Lima L. G., Howe E., Singh V. P. (2021). PCR amplicons identify widespread copy number variation in human centromeric arrays and instability in cancer. *Cell Genomics*.

[56] Gong Y., Gallis B. M., Goodlett D. R. (2013). Effects of transferrin conjugates of artemisinin and artemisinin dimer on breast cancer cell lines. *Anticancer Research*.

[57] Efferth T. (2006). Molecular pharmacology and pharmacogenomics of artemisinin and its derivatives in cancer cells. *Current Drug Targets*.

[58] Feng X., Li L., Jiang H., Jiang K., Jin Y., Zheng J. (2014). Dihydroartemisinin potentiates the anticancer effect of cisplatin via mTOR inhibition in cisplatin-resistant ovarian cancer cells: involvement of apoptosis and autophagy. *Biochemical and Biophysical Research Communications*.

[59] Efferth T. (2017). From ancient herb to modern drug: _Artemisia annua_ and artemisinin for cancer therapy. *Seminars in Cancer Biology*.

[60] Qin G., Zhao C., Zhang L. (2015). Dihydroartemisinin induces apoptosis preferentially via a Bim-mediated intrinsic pathway in hepatocarcinoma cells. *Apoptosis*.

[61] Handrick R., Ontikatze T., Bauer K. D. (2010). Dihydroartemisinin induces apoptosis by a Bak-dependent intrinsic pathway. *Molecular Cancer Therapeutics*.

[62] Zhao X., Zhong H., Wang R. (2015). Dihydroartemisinin and its derivative induce apoptosis in acute myeloid leukemia through Noxa-mediated pathway requiring iron and endoperoxide moiety. *Oncotarget*.

[63] Dixon S. J., Patel D. N., Welsch M. (2014). Pharmacological inhibition of cystine-glutamate exchange induces endoplasmic reticulum stress and ferroptosis. *eLife*.

[64] Cregan S. P., Dawson V. L., Slack R. S. (2004). Role of AIF in caspase-dependent and caspase-independent cell death. *Oncogene*.

[65] Candé C., Cohen I., Daugas E. (2002). Apoptosis-inducing factor (AIF): a novel caspase-independent death effector released from mitochondria. *Biochimie*.

[66] Morth J. P., Pedersen B. P., Buch-Pedersen M. J. (2011). A structural overview of the plasma membrane Na^+^,K^+^-ATPase and H^+^-ATPase ion pumps. *Nature Reviews. Molecular Cell Biology*.

[67] Wang L., Lu G., Shen H. M. (2020). The long and the short of PTEN in the regulation of mitophagy. *Frontiers in Cell and Development Biology*.

[68] Du J., Wang X., Li Y. (2021). DHA exhibits synergistic therapeutic efficacy with cisplatin to induce ferroptosis in pancreatic ductal adenocarcinoma via modulation of iron metabolism. *Cell Death & Disease*.

[69] Varmazyad M., Modi M. M., Kalen A. L. (2021). N-alkyl triphenylvinylpyridinium conjugated dihydroartemisinin perturbs mitochondrial functions resulting in enhanced cancer versus normal cell toxicity. *Free Radical Biology & Medicine*.

[70] Tang D., Chen X., Kang R., Kroemer G. (2021). Ferroptosis: molecular mechanisms and health implications. *Cell Research*.

[71] Yang W. S., Stockwell B. R. (2008). Synthetic lethal screening identifies compounds activating iron-dependent, nonapoptotic cell death in oncogenic-RAS-harboring cancer cells. *Chemistry & Biology*.

[72] Ruicci K. M., Pinto N., Khan M. I. (2018). ERK-TSC2 signalling in constitutively-active HRAS mutant HNSCC cells promotes resistance to PI3K inhibition. *Oral Oncology*.

[73] Gao P., Liu S., Yoshida R. (2019). Ral GTPase activation by downregulation of RalGAP enhances oral squamous cell carcinoma progression. *Journal of Dental Research*.

[74] Ho A. L., Brana I., Haddad R. (2021). Tipifarnib in head and neck squamous cell carcinoma with HRAS mutations. *Journal of Clinical Oncology*.

[75] Jia L., Wang J., Wu T., Wu J., Ling J., Cheng B. (2017). In vitro and in vivo antitumor effects of chloroquine on oral squamous cell carcinoma. *Molecular Medicine Reports*.

[76] Li Q., Liu X., Yan W., Chen Y. (2020). Antitumor effect of poly lactic acid nanoparticles loaded with cisplatin and chloroquine on the oral squamous cell carcinoma. *Aging*.

[77] Quan H. Y., Quan H. Y., Zhou L. J., Li A. D., Zhang Z. B. (2015). Mechanism of chloroquine in promoting sensitivity of chemotherapeutics in oral squamous cell carcinoma CAL-27 cell line to cisplatin. *Shanghai Kou Qiang Yi Xue*.

[78] Li J., Yang D., Wang W. (2015). Inhibition of autophagy by 3-MA enhances IL-24-induced apoptosis in human oral squamous cell carcinoma cells. *Journal of Experimental & Clinical Cancer Research*.

[79] Zhang J., Mao W., Liu Y. (2021). 3-MA enhanced chemosensitivity in cisplatin resistant hypopharyngeal squamous carcinoma cells via inhibiting Beclin-1 mediated autophagy. *Current Pharmaceutical Design*.

[80] Dai W., Wang G., Chwa J. (2020). Mitochondrial division inhibitor (mdivi-1) decreases oxidative metabolism in cancer. *British Journal of Cancer*.

[81] Mah L. J., El-Osta A., Karagiannis T. C. (2010). *γ*H2AX: a sensitive molecular marker of DNA damage and repair. *Leukemia*.

