# Supplementary figures and images for: Repurposing Dihydroartemisinin to Combat Oral Squamous Cell Carcinoma, Associated with Mitochondrial Dysfunction and Oxidative Stress
Source: Oxid Med Cell Longev. 2023 Feb 16;2023:9595201. doi: 10.1155/2023/9595201 (PMC10239307; doi:10.1155/2023/9595201)

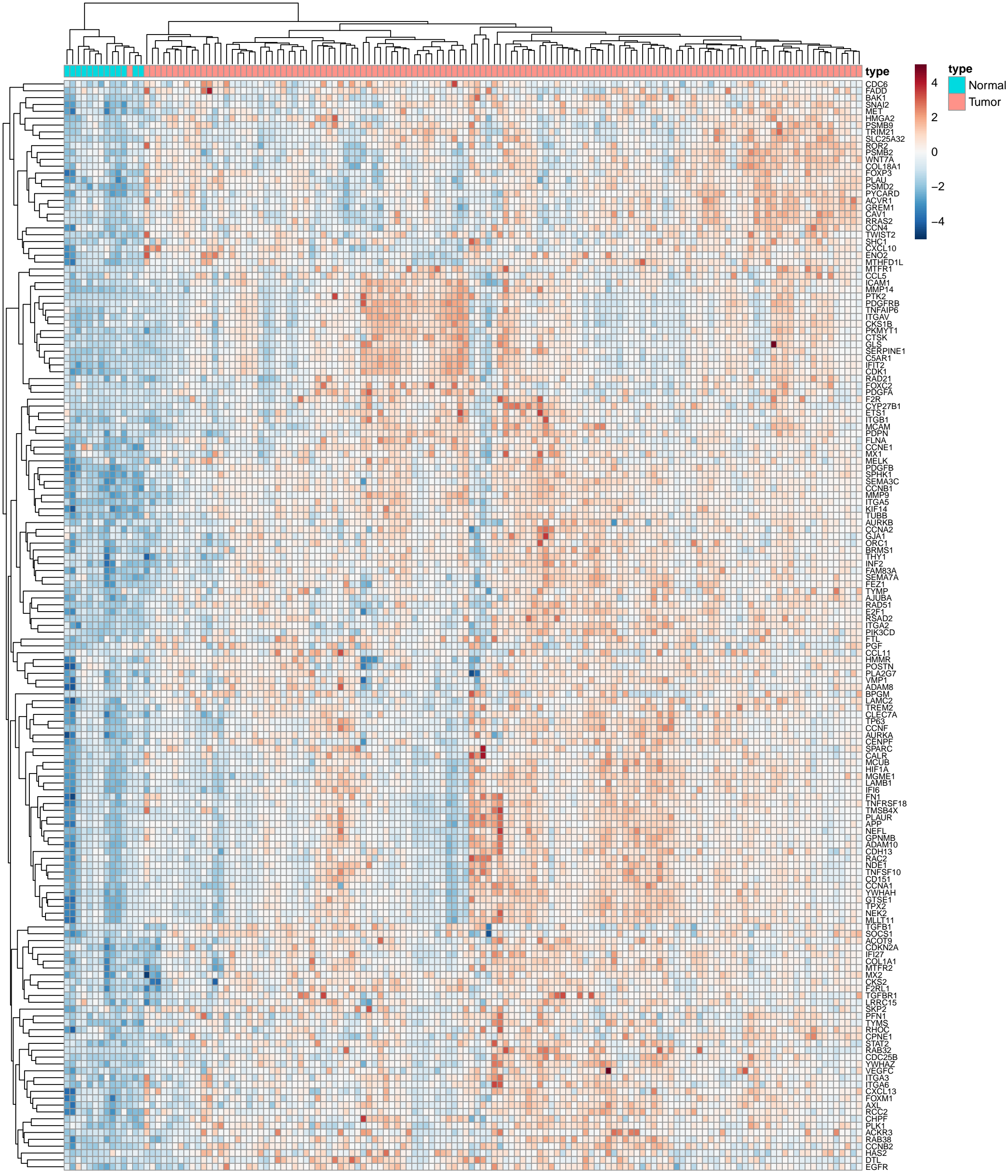

Supplement: Supplementary 1 — Supplementary Figure 1: the heat map data showed that 159 genes were found to be significantly differentially expressed between tumor and normal samples using the R package edgeR with FDR < = 0.01, fold change > = 2, and the average gene expression > = 2. [file 9595201.f1.pdf]

## Slide 1
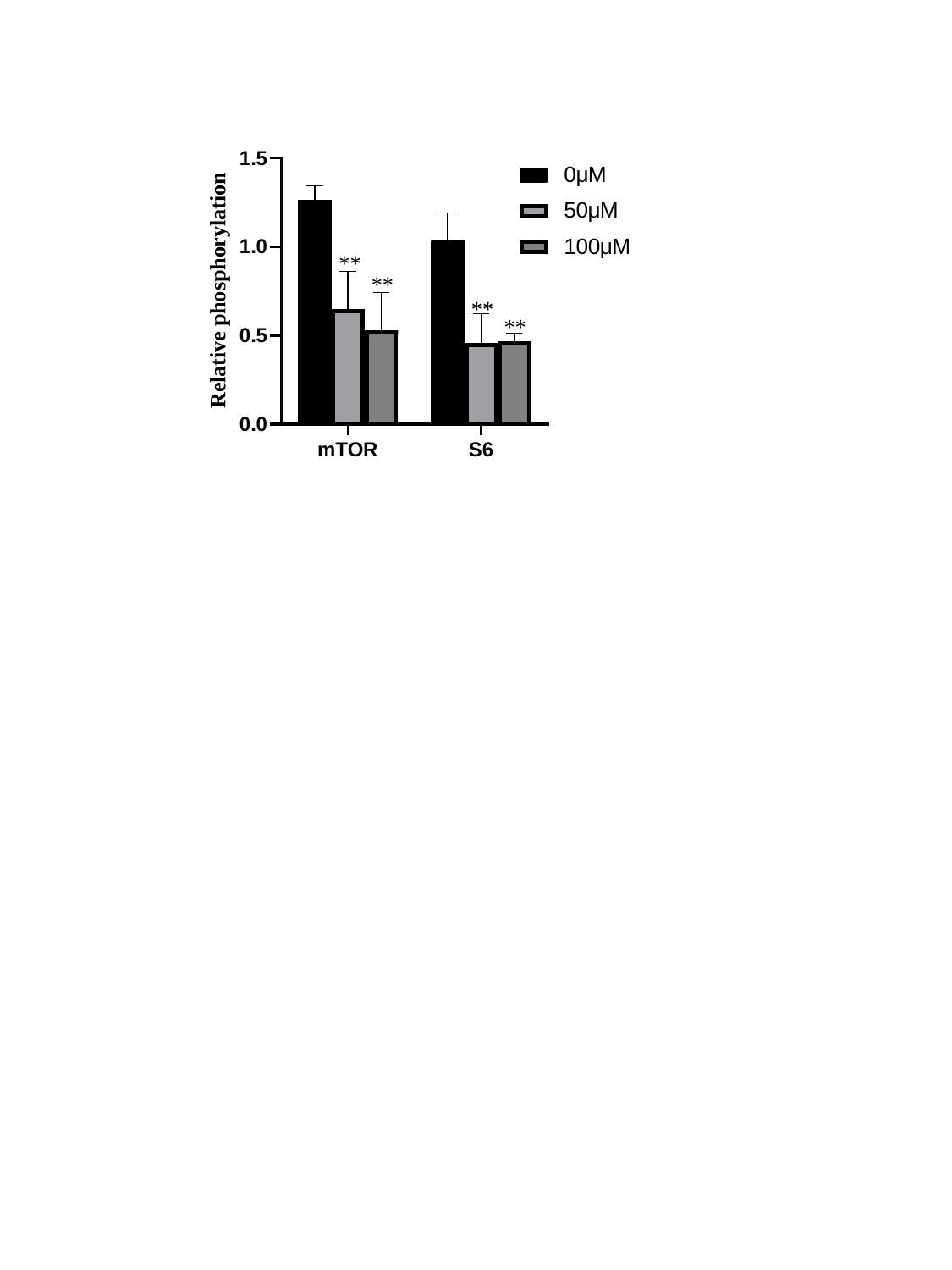

Supplement: Supplementary 3 — Supplementary Figure 3: expression and phosphorylation of mTOR and S6 were detected by western blot and normalized to mTOR and S6 (t-test, ∗∗P < 0.01; means ± SE, ns, not significant, n = 3). [file 9595201.f3.pptx]

## Slide 1
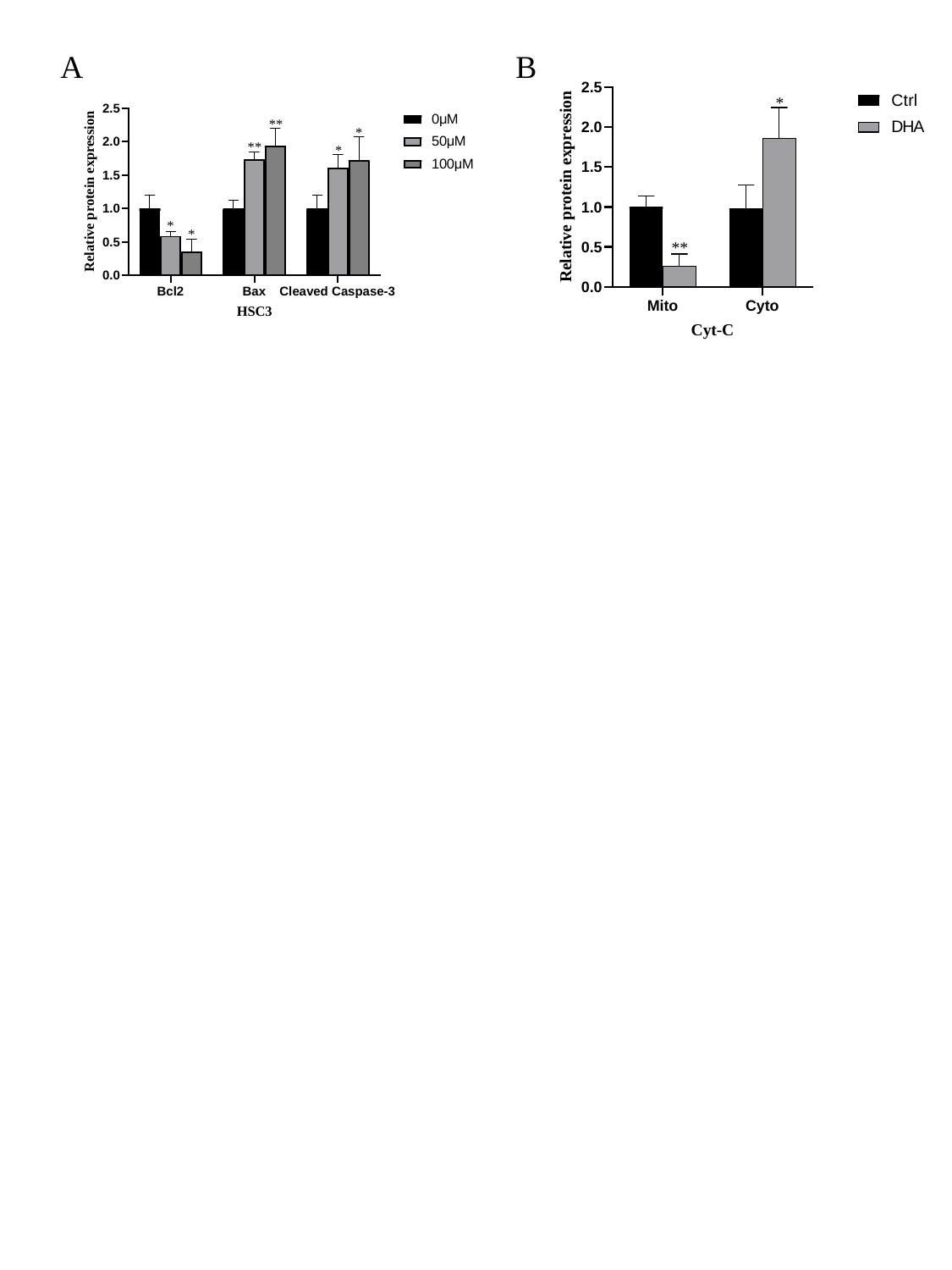

A
B

Supplement: Supplementary 4 — Supplementary Figure 4: (a) quantification of Bcl2, Bax, and Cleaved Caspase-3 protein expression in HSC3 of the indicated treatment (t-test, ∗P < 0.5, ∗∗P < 0.01; means ± SE, ns, not significant, n = 3). (b) The level of cytochrome C (Cyt-c) was assessed in mitochondrial fractions and cytosolic fractions (t-test, ∗P < 0.5, ∗∗P < 0.01; means ± SE, ns, not significant, n = 3). [file 9595201.f4.pptx]

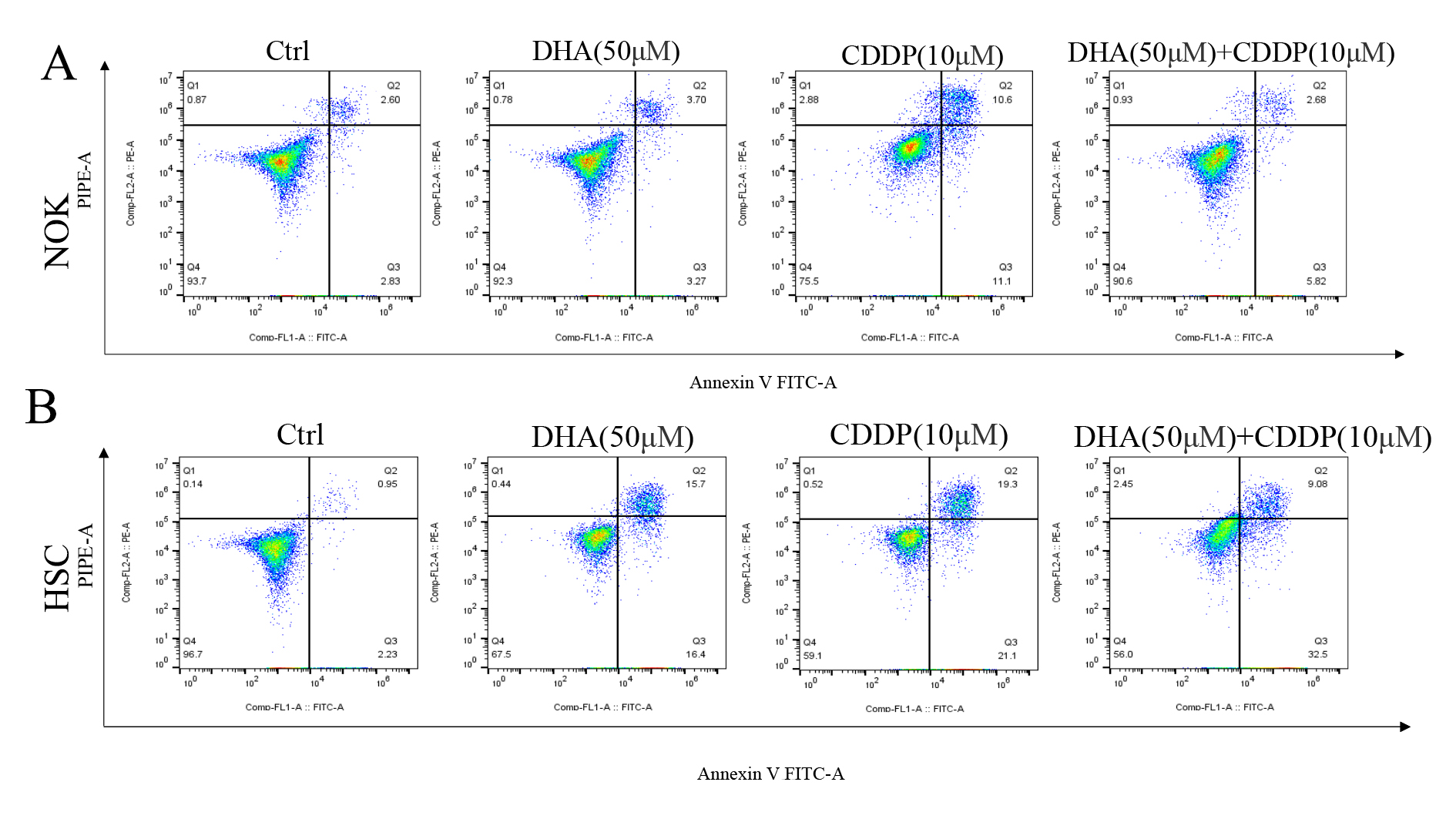

Supplement: Supplementary 5 — Supplementary Figure 5: (a) NOK cells were treated with DHA, CDDP, and the combination (DHA + CDDP) for 48 hours. Cell apoptosis was measured with Annexin V-FITC/PI dual staining by flow cytometry analysis. (b) HSC3 OSCC tumor cells were treated with DHA, CDDP, and the combination (DHA + CDDP) for 48 hours. Cell apoptosis was measured with Annexin V-FITC/PI dual staining by flow cytometry analysis. [file 9595201.f5.jpg]

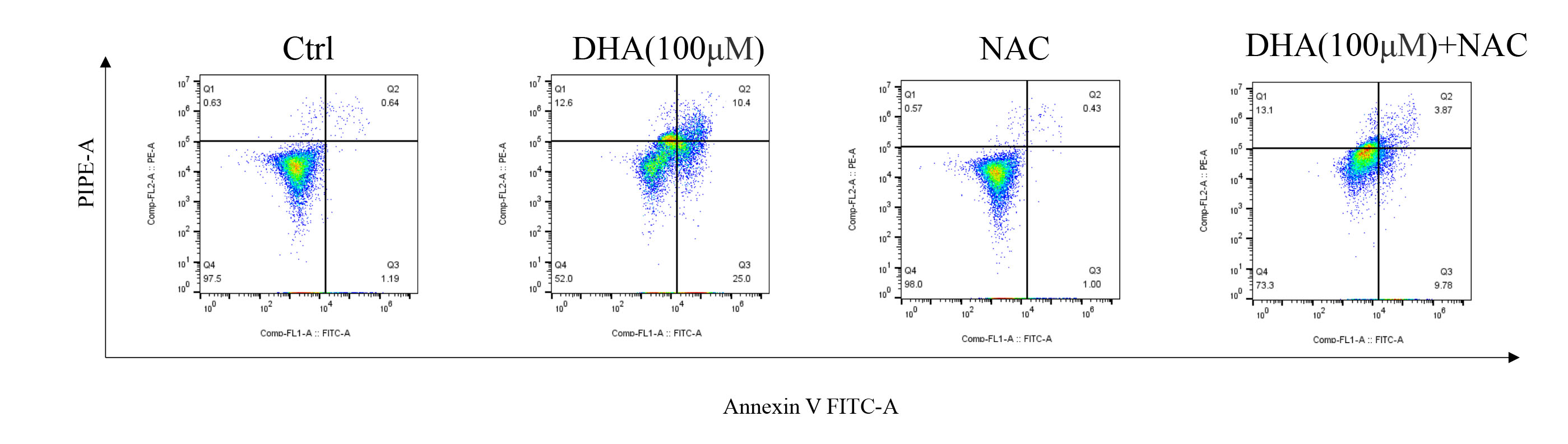

Supplement: Supplementary 6 — Supplementary Figure 6: OSCC HSC3 tumor cells were treated by DHA for 48 h with or without pretreatment of NAC. Cell apoptosis was measured with Annexin V-FITC/PI dual staining by flow cytometry analysis. [file 9595201.f6.jpg]

## Slide 1
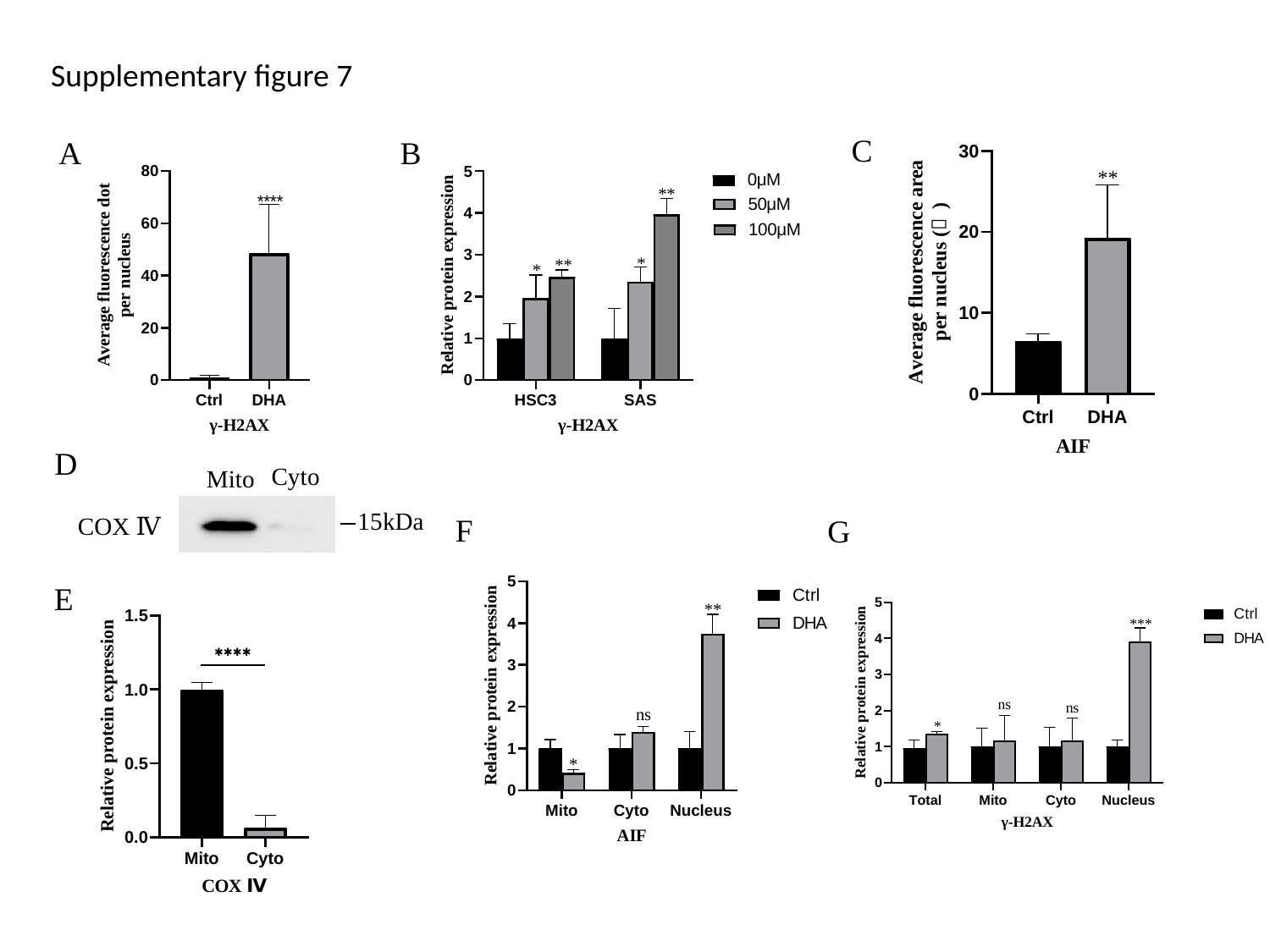

Supplementary figure 7
C
A
B
D
Cyto
Mito
15kDa
COX Ⅳ
F
G
E

Supplement: Supplementary 7 — Supplementary Figure 7: DHA induced DNA damage. (a) Average fluorescence dot per nucleus of γ-H2AX was analyzed in the OSCC cells (t-test, ∗∗∗∗P < 0.0001; means ± SE, ns, not significant, n = 3). (b) Protein expression level of γ-H2AX was determind (t-test, ∗P < 0.5, ∗∗P < 0.01; means ± SE, ns, not significant, n = 3). (c) Statistical analysis of AIF average fluorescence area in nucleus (t-test, ∗∗P < 0.01; means ± SE, ns, not significant, n = 3). (d) The mitochondrial loading control COX IV was used to validate the purity of mitochondria part by Western blotting analysis. (e) The expression of COX IV was quantified in mitochondrial and cytoplasm (t-test, ∗∗∗∗P < 0.0001; means ± SE, ns, not significant, n = 3). (f) Quantification of the level of AIF in mitochondrial,cytoplasm, and nucleus (t-test, ∗P < 0.5, ∗∗P < 0.01; means ± SE, ns, not significant, n = 3). (g) Relative protein expression of γ-H2AX was, respectively, assessed mitochondria part, cytoplasm part, and cell nucleus (t-test, ∗P < 0.5, ∗∗P < 0.01; means ± SE, ns, not significant, n = 3). [file 9595201.f7.pptx]

## Slide 1
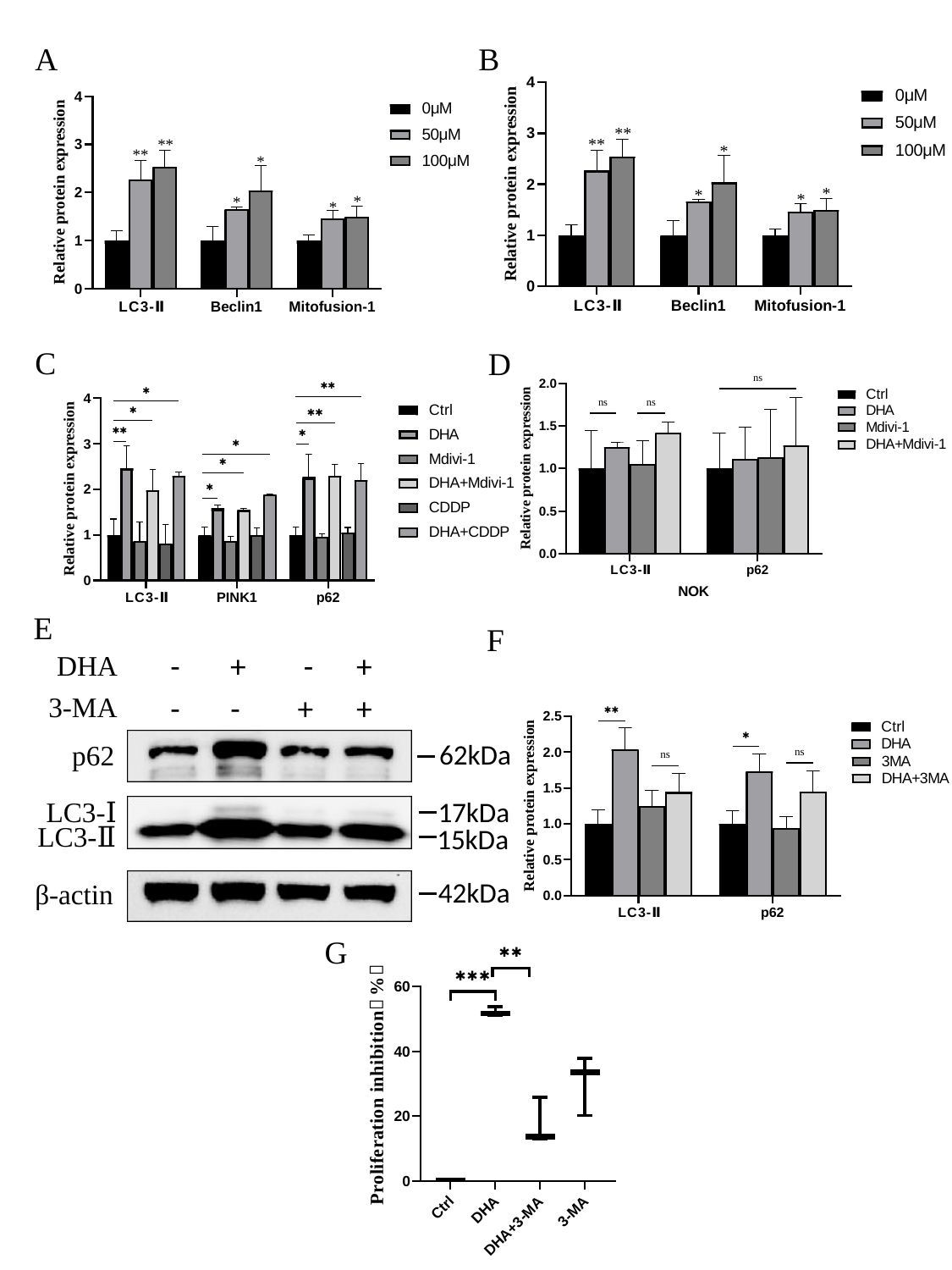

B
A
C
D
E
F
- + - +
DHA
 - - + +
3-MA
62kDa
p62
LC3-Ⅰ
17kDa
LC3-Ⅱ
15kDa
42kDa
β-actin
G

Supplement: Supplementary 8 — Supplementary Figure 8: the effect of 3-MA on DHA action in the OSCC cells. (a) Relative protein expression of LC3-II, Beclin1, and Mitofusion-1 were determined to assess mitophagy (t-test, ∗P < 0.5, ∗∗P < 0.01; means ± SE, ns, not significant, n = 3). (b) The level of Mitofusion-1 was analyzed to evaluate the mitophagy of OSCC cells with treatment of DHA and Mdivi-1 (t-test, ∗P < 0.5; means ± SE, ns, not significant, n = 3). (c) The protein level of LC3-II, PINK1, and p62 was assessed and nomalized to β-Tubulin (t-test, ∗P < 0.5, ∗∗P < 0.01; means ± SE, ns, not significant, n = 3). (d) Autophage asscociated protein expression of NOK cells was detected (t-test, means ± SE, ns, not significant, n = 3). (e) HSC3 cells were preincubated with 3-MA to preincubate for 2 h and then treated with or without DHA for 24 h for Western blot analysis. (f) HSC3 cells were preincubated with 3-MA to preincubate for 2 h and then treated with or without DHA for 48 h for Western blotting. (g) Statistical analysis of proliferation inhibition of cells was assessed with the indicated treatmen (t-test, ∗P < 0.5, ∗∗P < 0.01; means ± SE, ns, not significant, n = 3). [file 9595201.f8.pptx]
