# Supplementary material for: Repurposing Dihydroartemisinin to Combat Oral Squamous Cell Carcinoma, Associated with Mitochondrial Dysfunction and Oxidative Stress
Source: Oxid Med Cell Longev. 2023 Feb 16;2023:9595201. doi: 10.1155/2023/9595201 (PMC10239307; doi:10.1155/2023/9595201)
Supplement: Supplementary 2 — Supplementary Figure 2: the boxplots showed the genes are significantly upregulated at each stage of OSCC tumor progression. [file 9595201.f2.pdf]

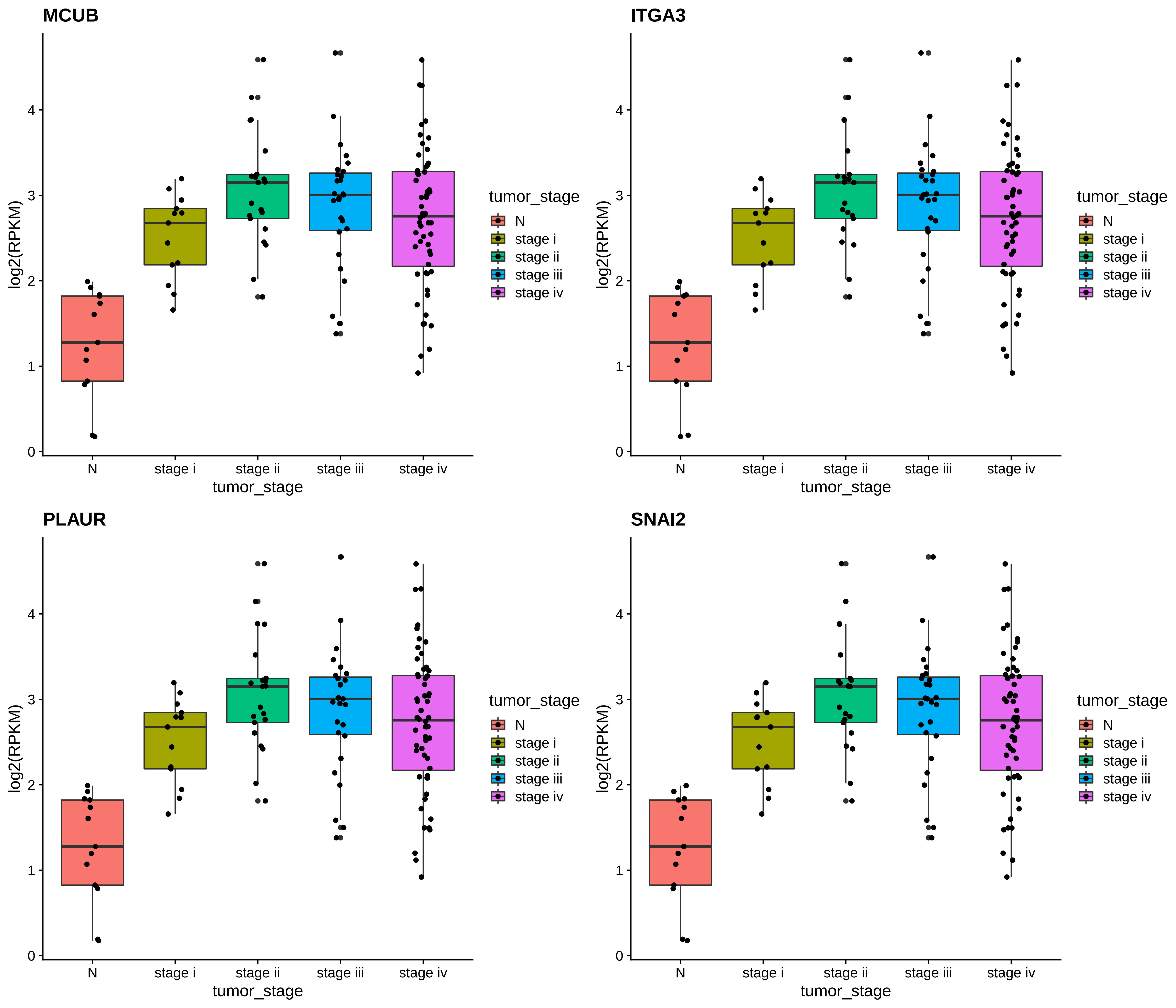
MCUB
ITGA3
PLAUR
SNAI2
log2(RPKM)
tumor_stage
N
stage i
stage ii
stage iii
stage iv
0
1
2
3
4

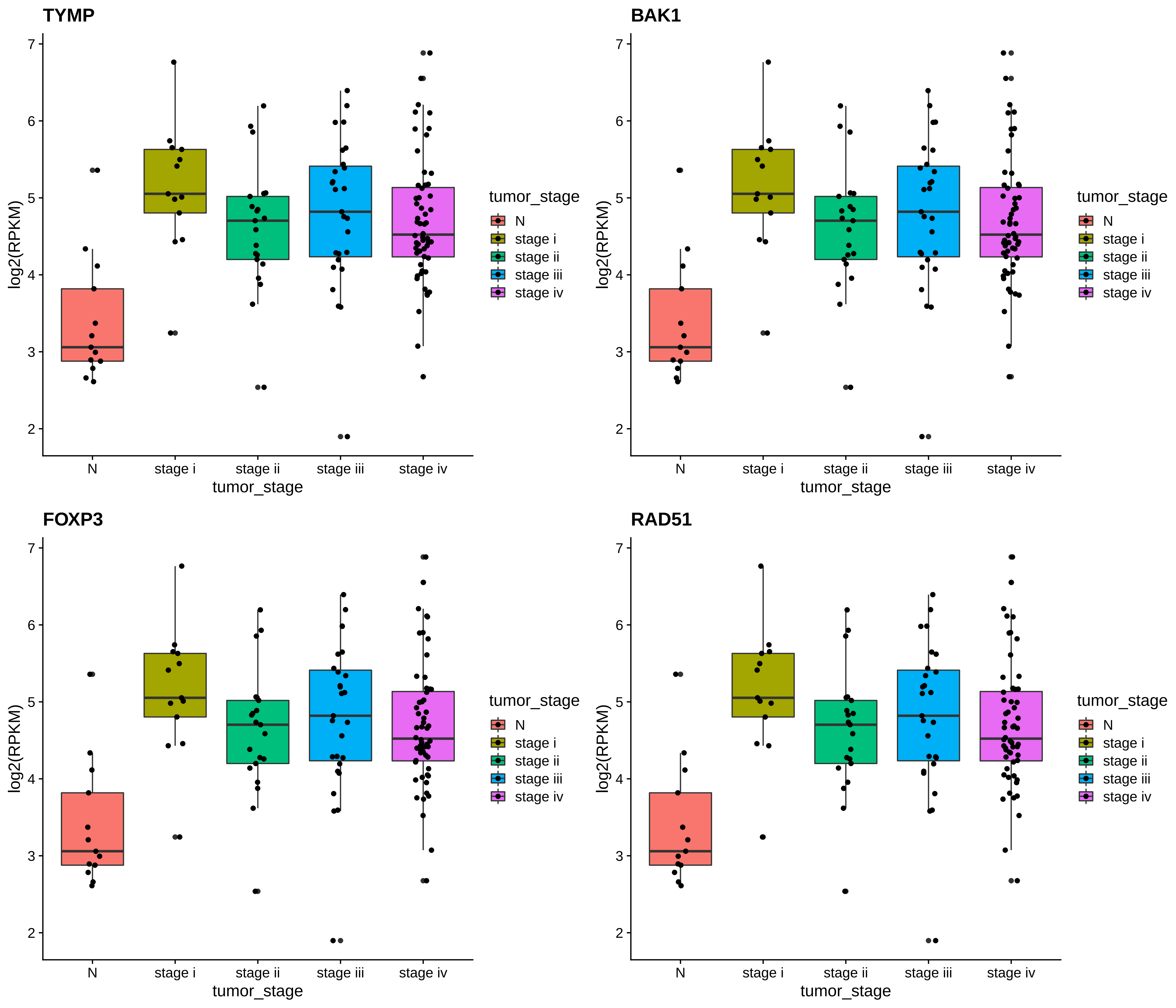
TYMP
BAK1
FOXP3
RAD51
log2(RPKM)
tumor_stage
N
stage i
stage ii
stage iii
stage iv
2
3
4
5
6
7

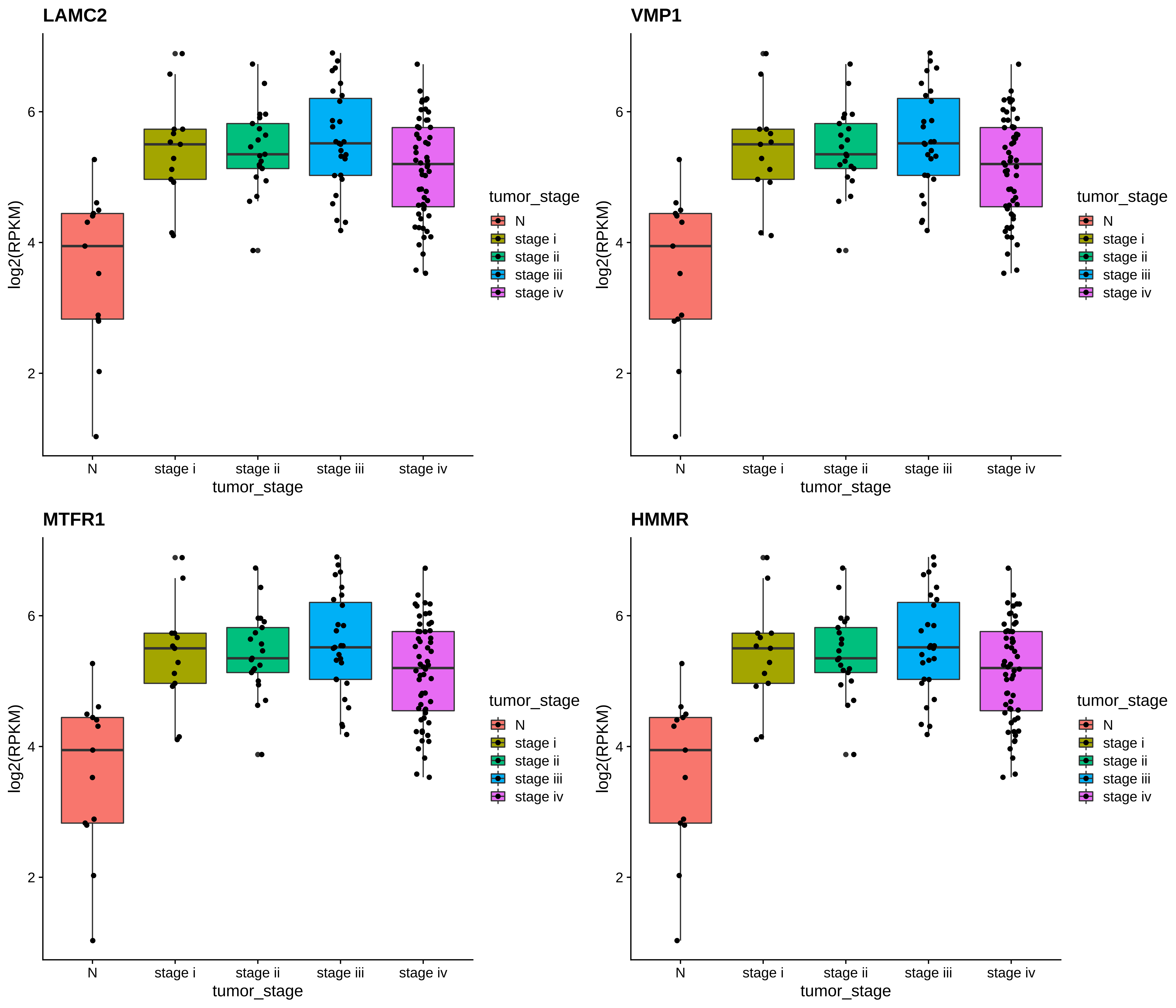

LAMC2
VMP1
MTFR1
HMMR
log2(RPKM)
tumor_stage
N
stage i
stage ii
stage iii
stage iv
2
4
6

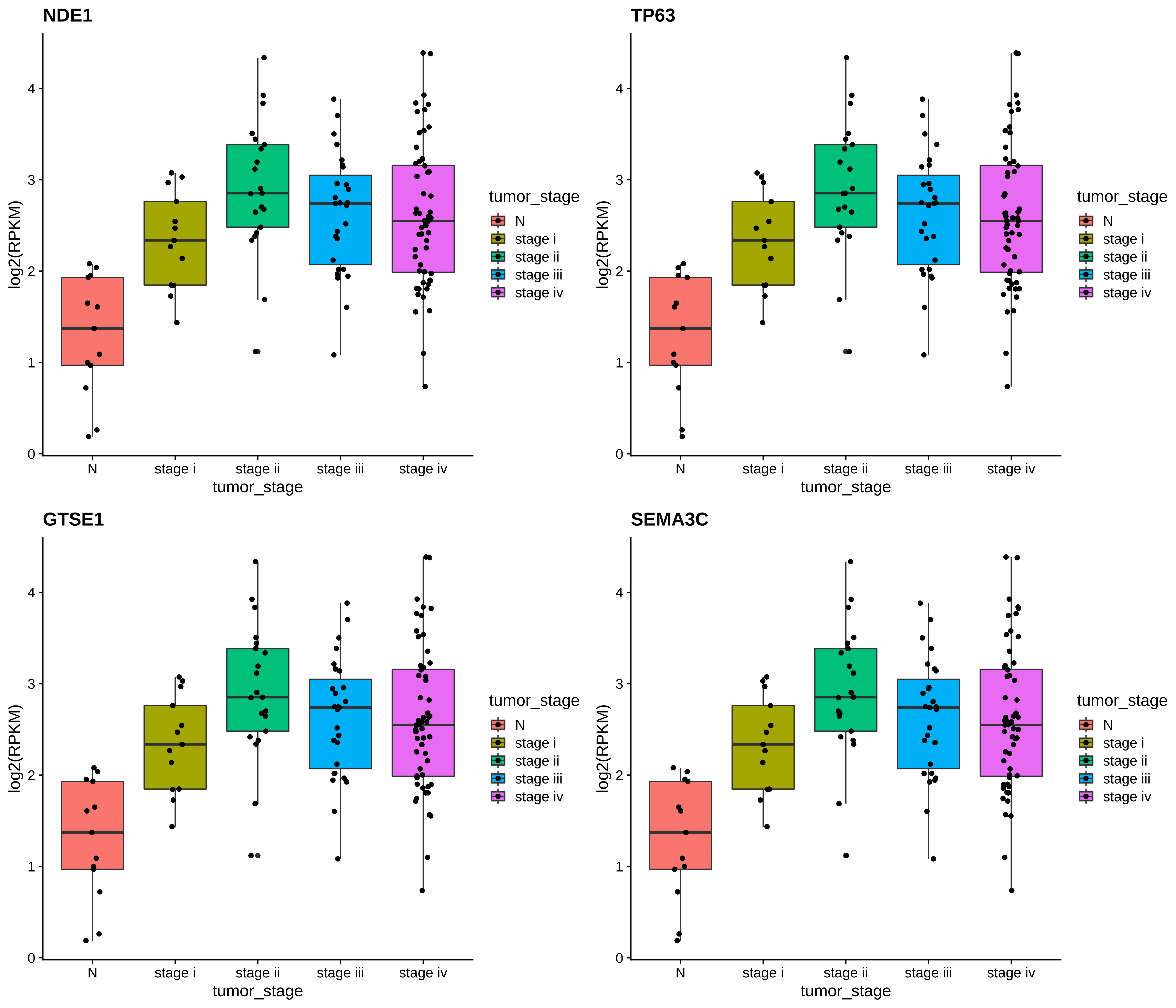
NDE1
TP63
GTSE1
SEMA3C
log2(RPKM)
tumor_stage
N
stage i
stage ii
stage iii
stage iv
0
1
2
3
4

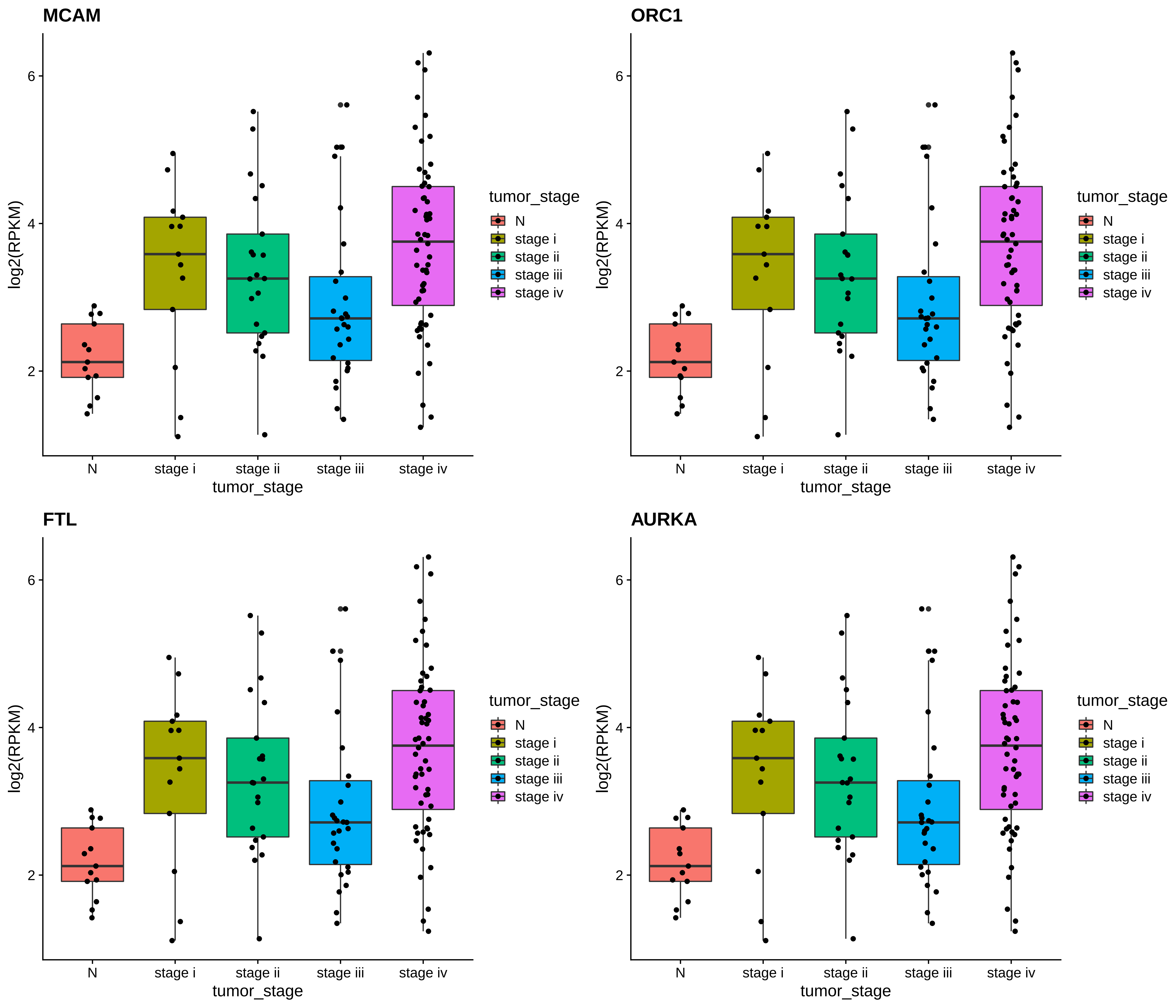
MCAM
ORC1
FTL
AURKA
log2(RPKM)
2
4
6
N
stage i
stage ii
stage iii
stage iv
tumor_stage

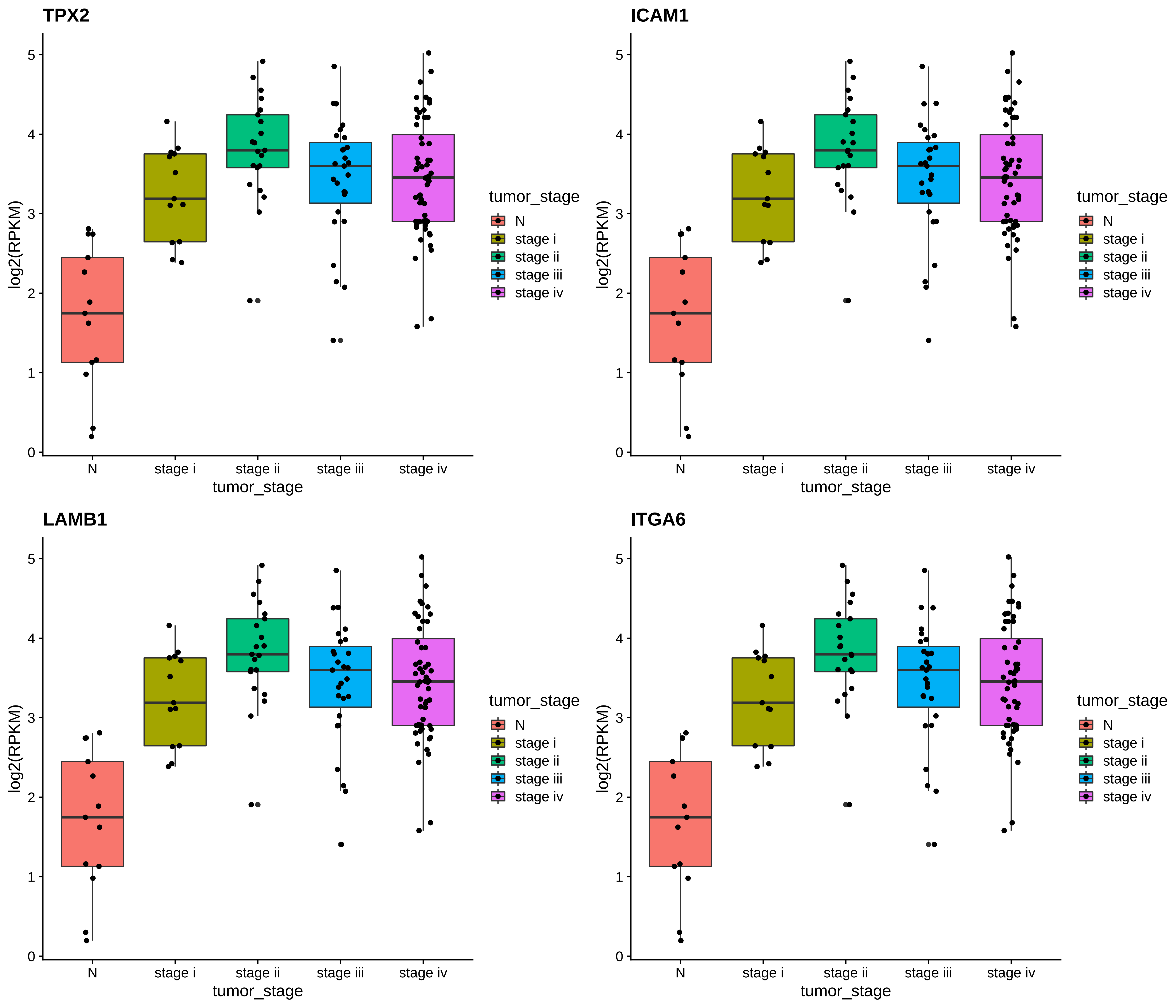
TPX2
ICAM1
LAMB1
ITGA6
log2(RPKM)
tumor_stage
N
stage i
stage ii
stage iii
stage iv
0
1
2
3
4
5

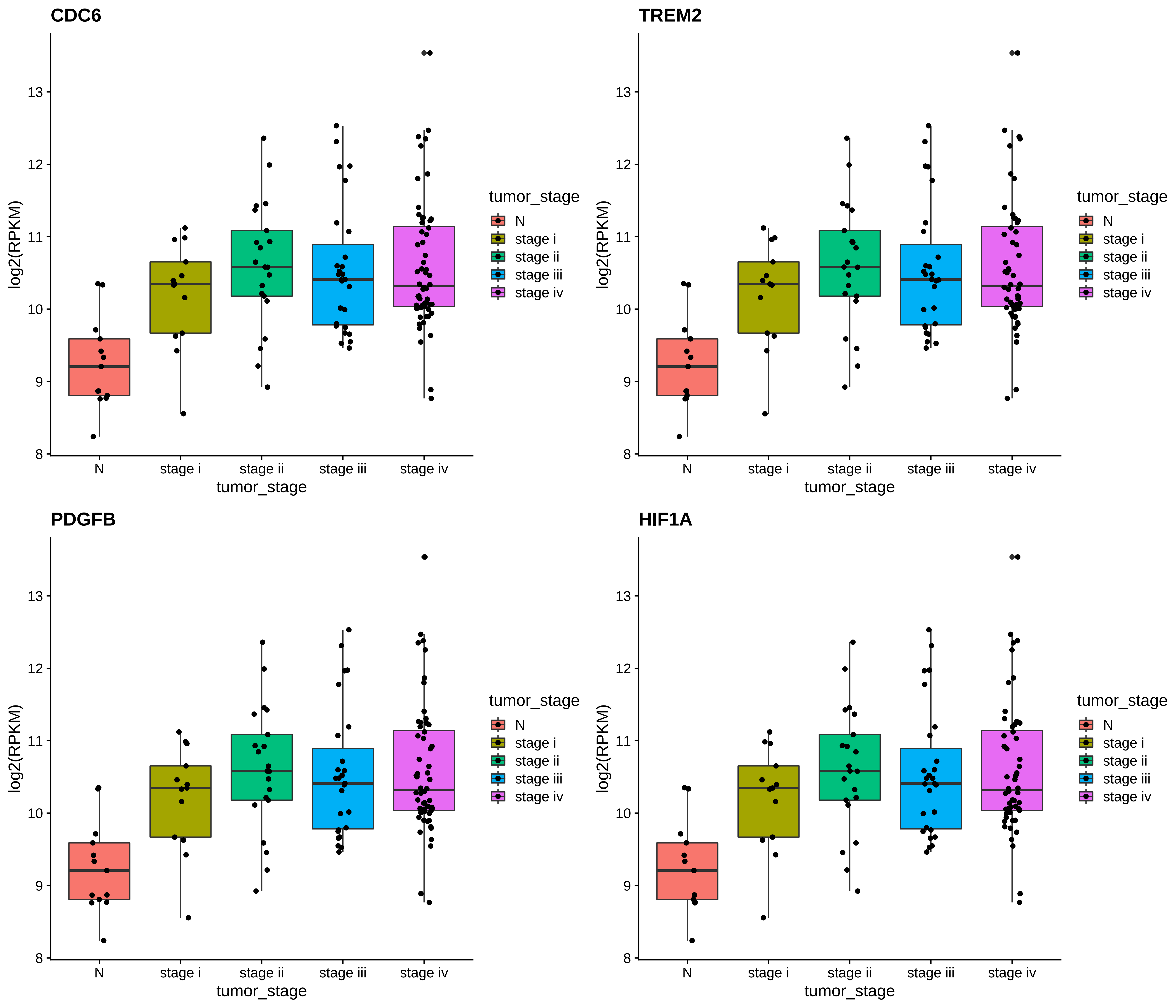
CDC6
TREM2
PDGFB
HIF1A
log2(RPKM)
tumor_stage
N
stage i
stage ii
stage iii
stage iv
8
9
10
11
12
13

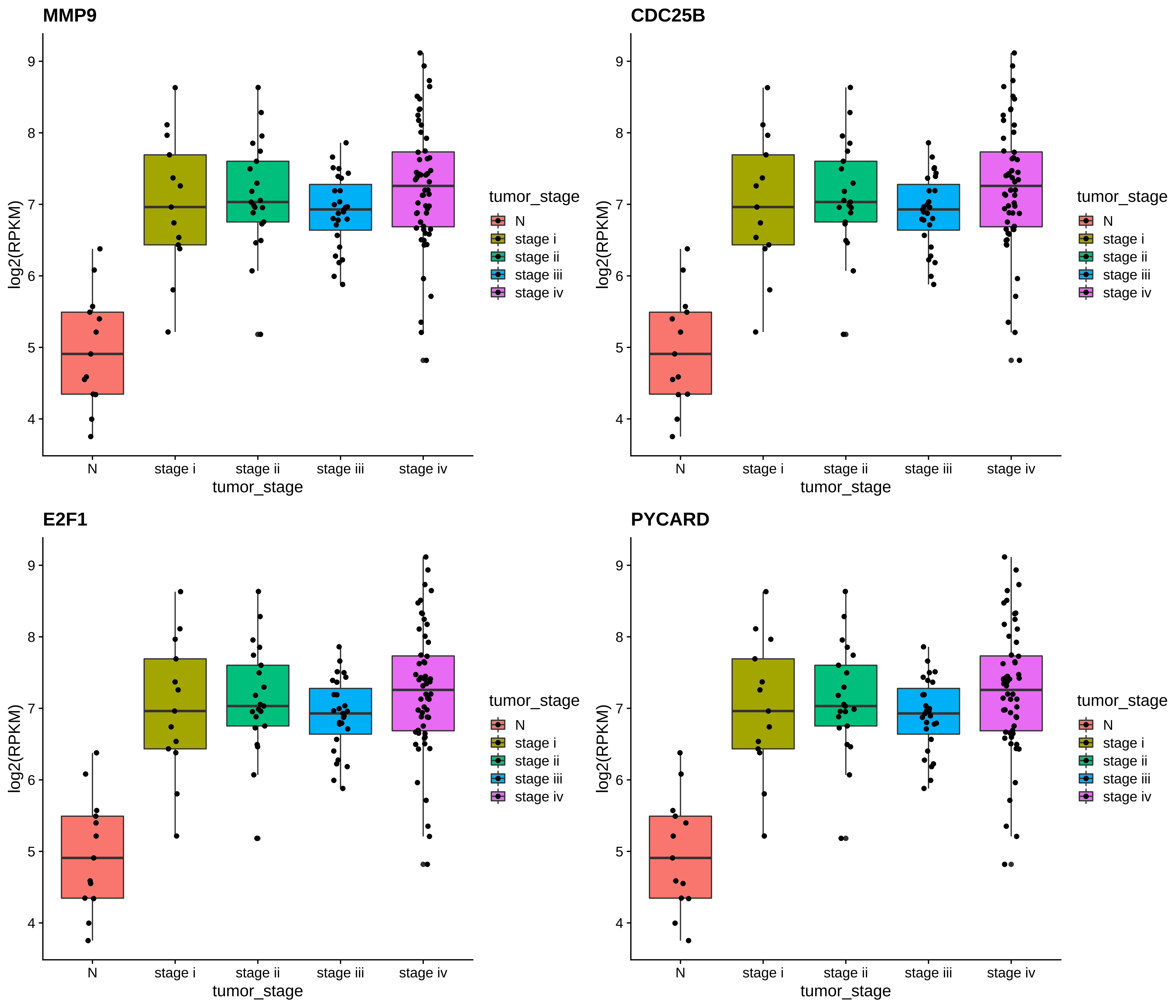

MMP9
CDC25B
E2F1
PYCARD
log2(RPKM)
tumor_stage
N
stage i
stage ii
stage iii
stage iv

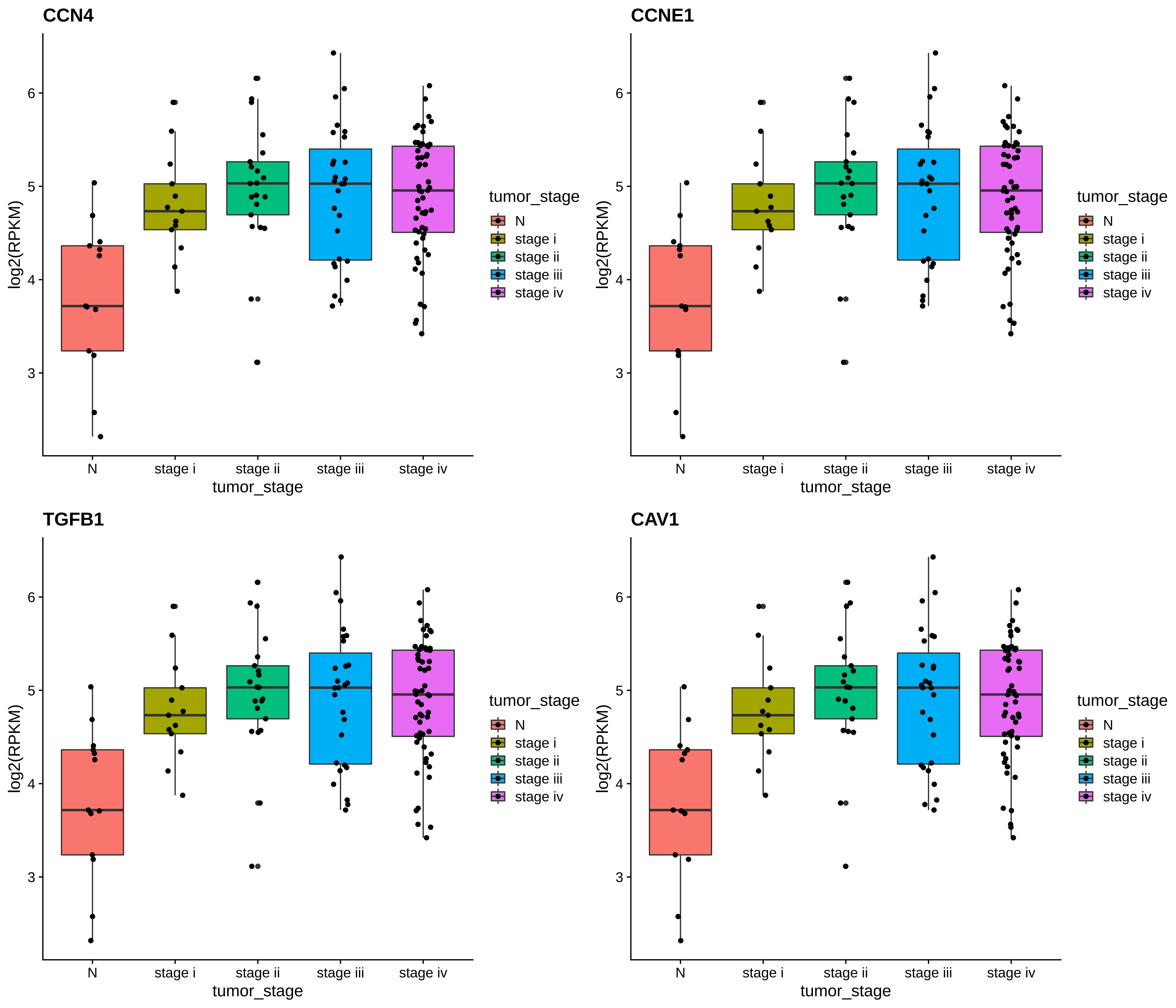
CCN4
CCNE1
TGFB1
CAV1
log2(RPKM)
tumor_stage
N
stage i
stage ii
stage iii
stage iv
3
4
5
6

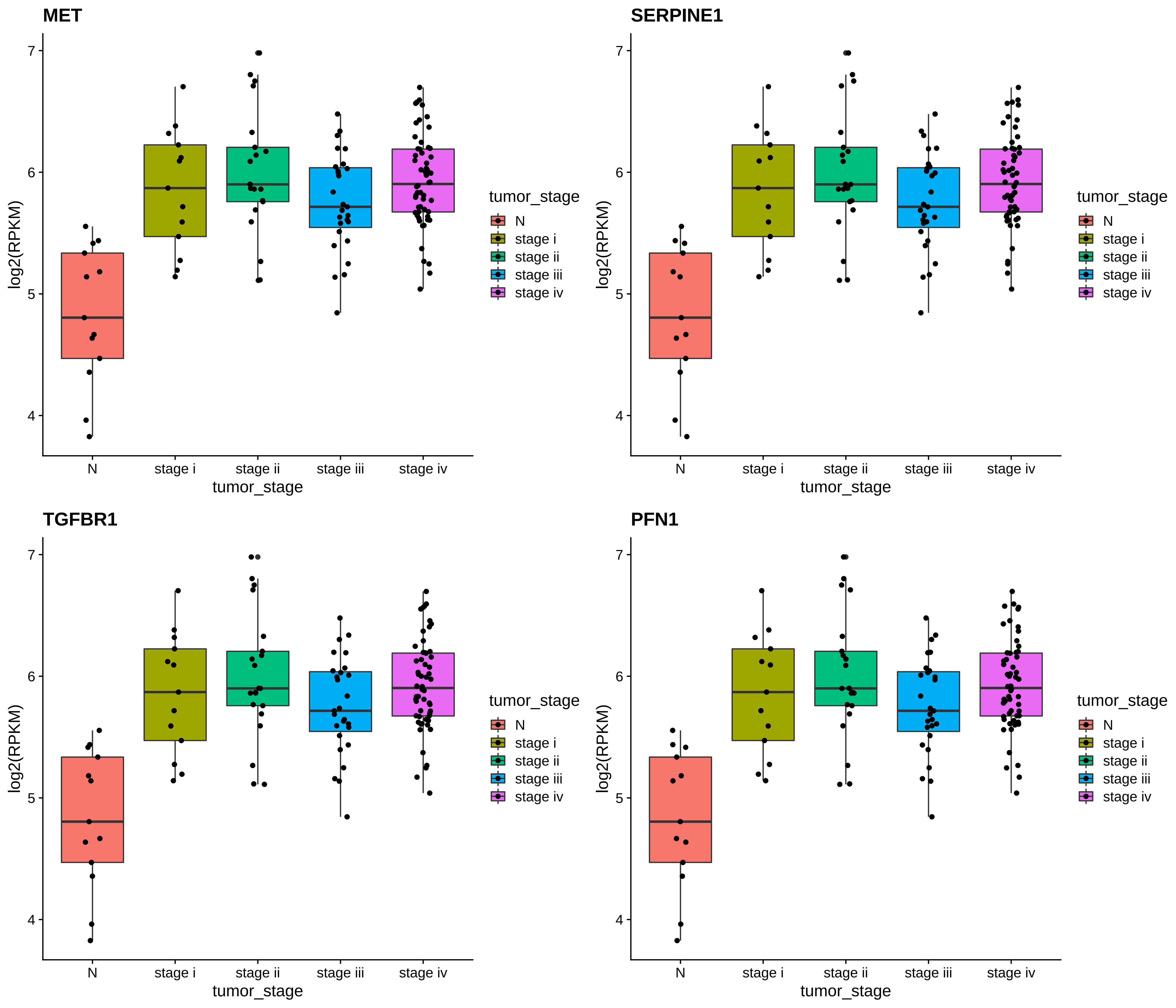
MET
SERPINE1
TGFBR1
PFN1
log2(RPKM)
4
5
6
7
N
stage i
stage ii
stage iii
stage iv
tumor_stage
tumor_stage
N
stage i
stage ii
stage iii
stage iv

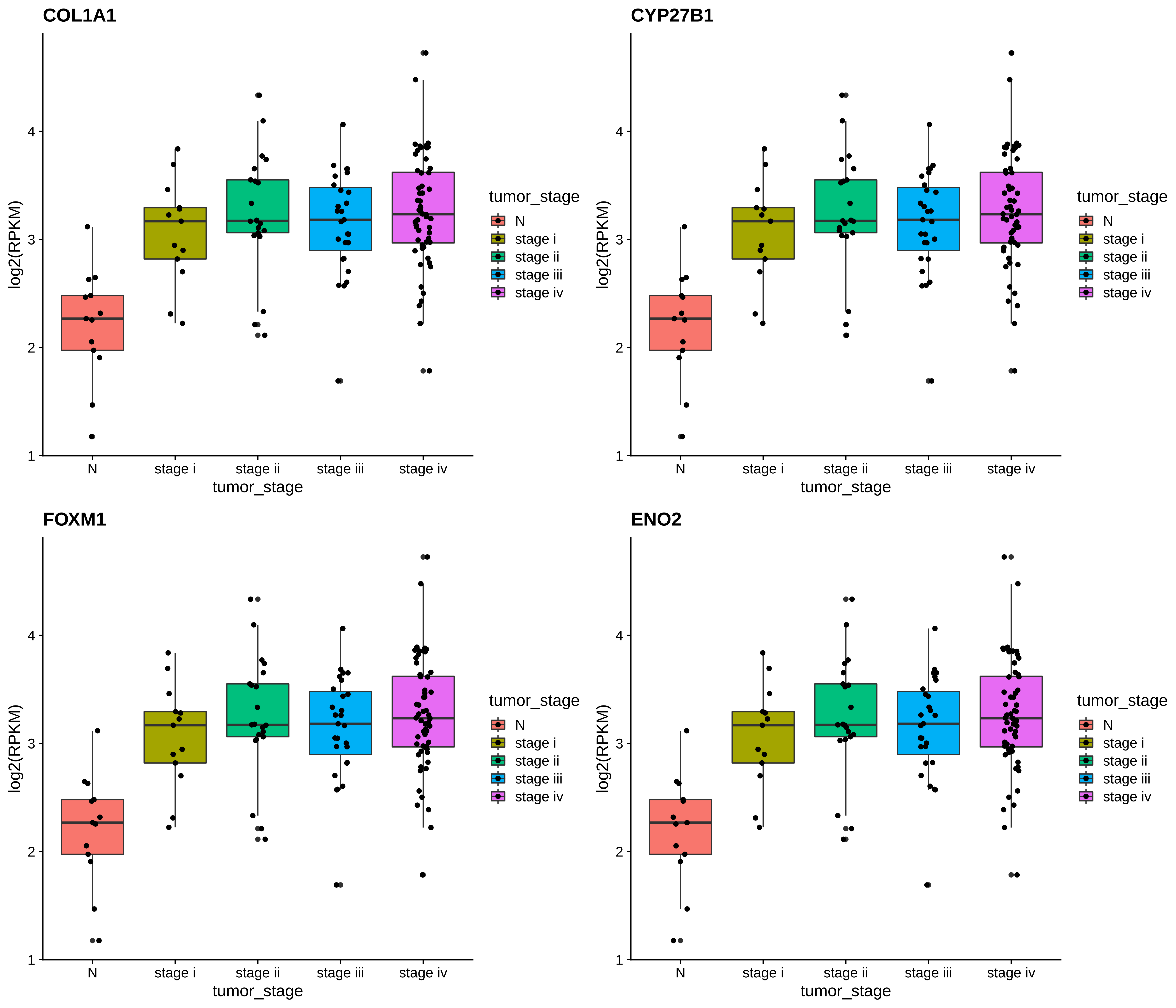
COL1A1
CYP27B1
FOXM1
ENO2
log2(RPKM)
tumor_stage
N
stage i
stage ii
stage iii
stage iv
1
2
3
4

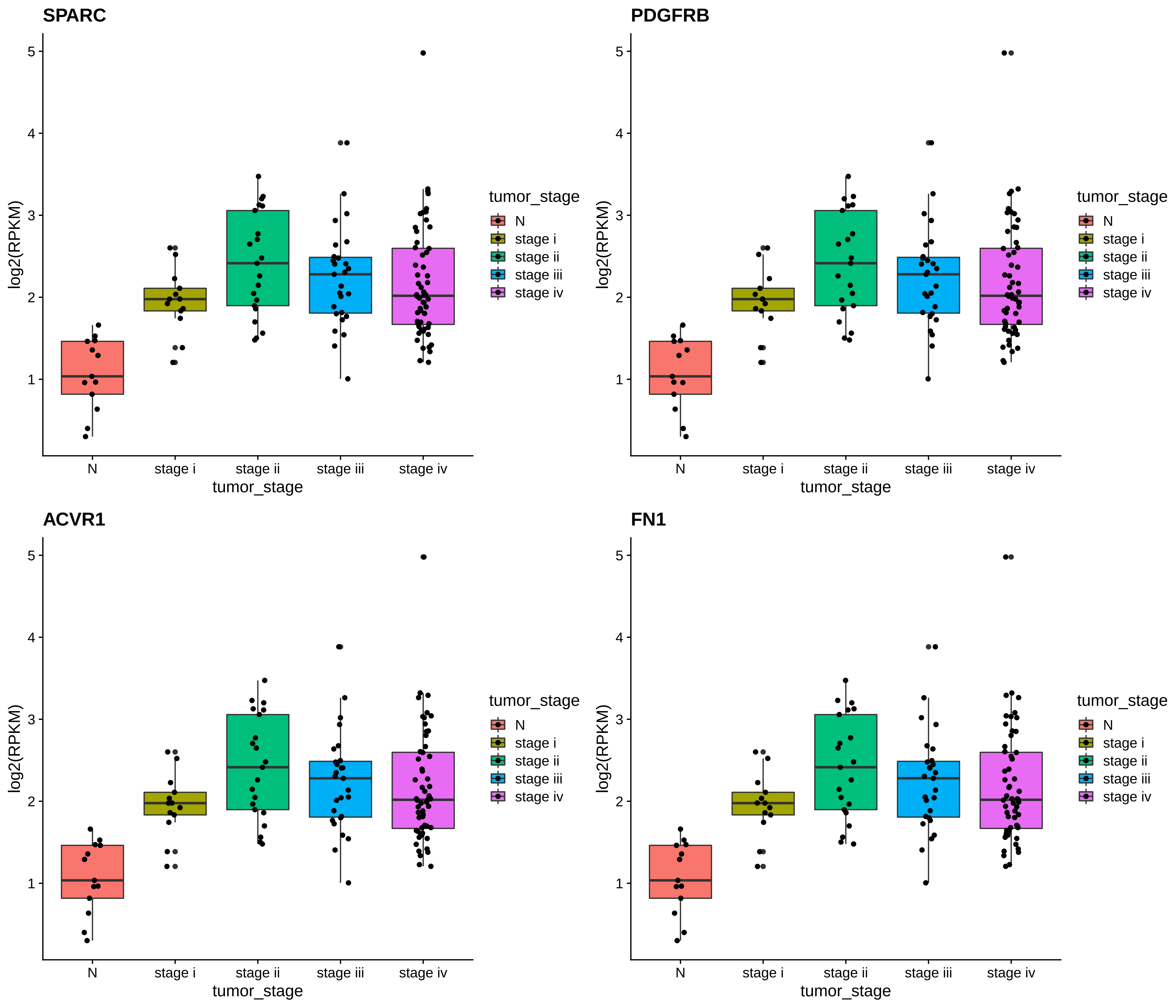
SPARC
PDGFRB
ACVR1
FN1
log2(RPKM)
tumor_stage
N
stage i
stage ii
stage iii
stage iv
1
2
3
4
5

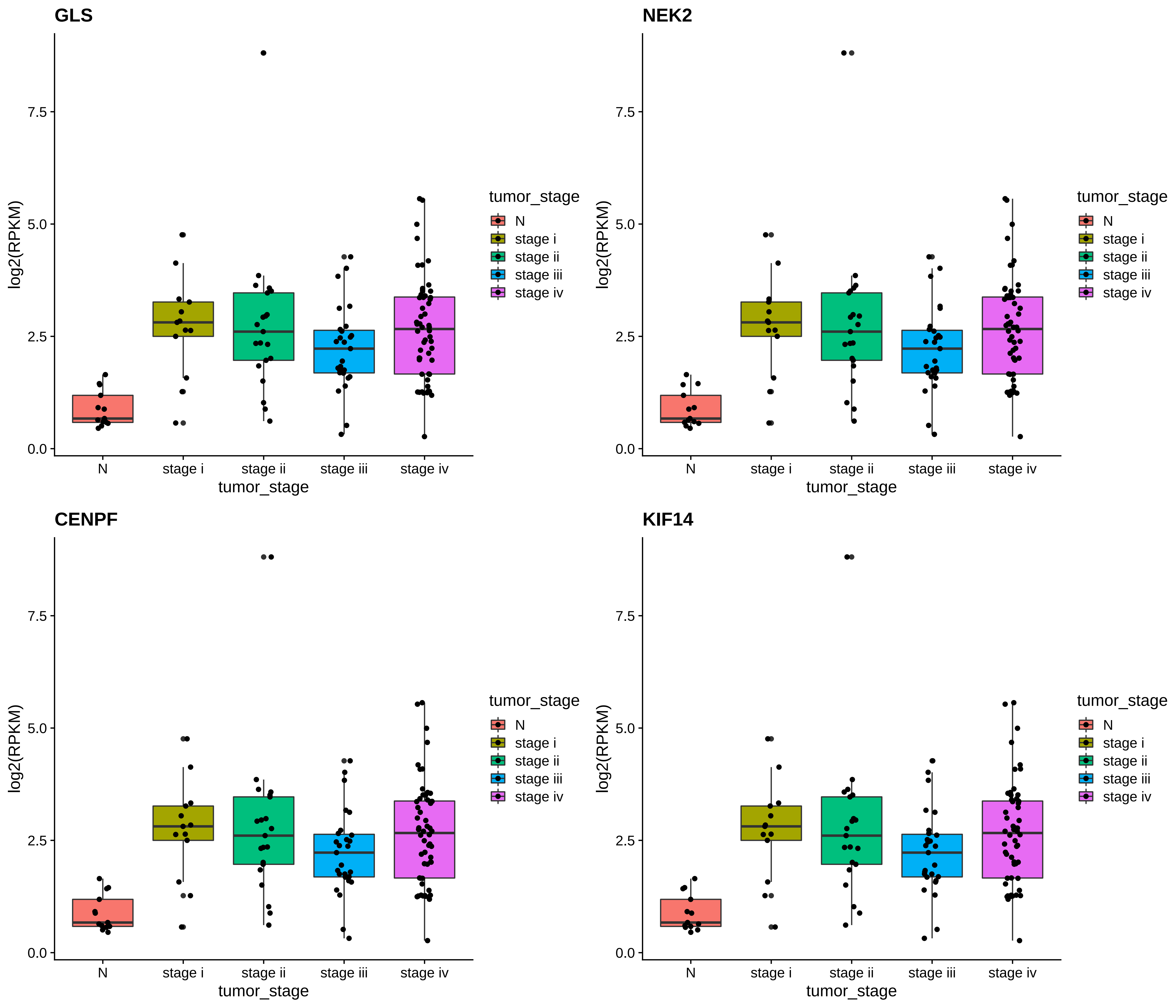
GLS
NEK2
CENPF
KIF14
log2(RPKM)
tumor_stage
N
stage i
stage ii
stage iii
stage iv
0.0
2.5
5.0
7.5

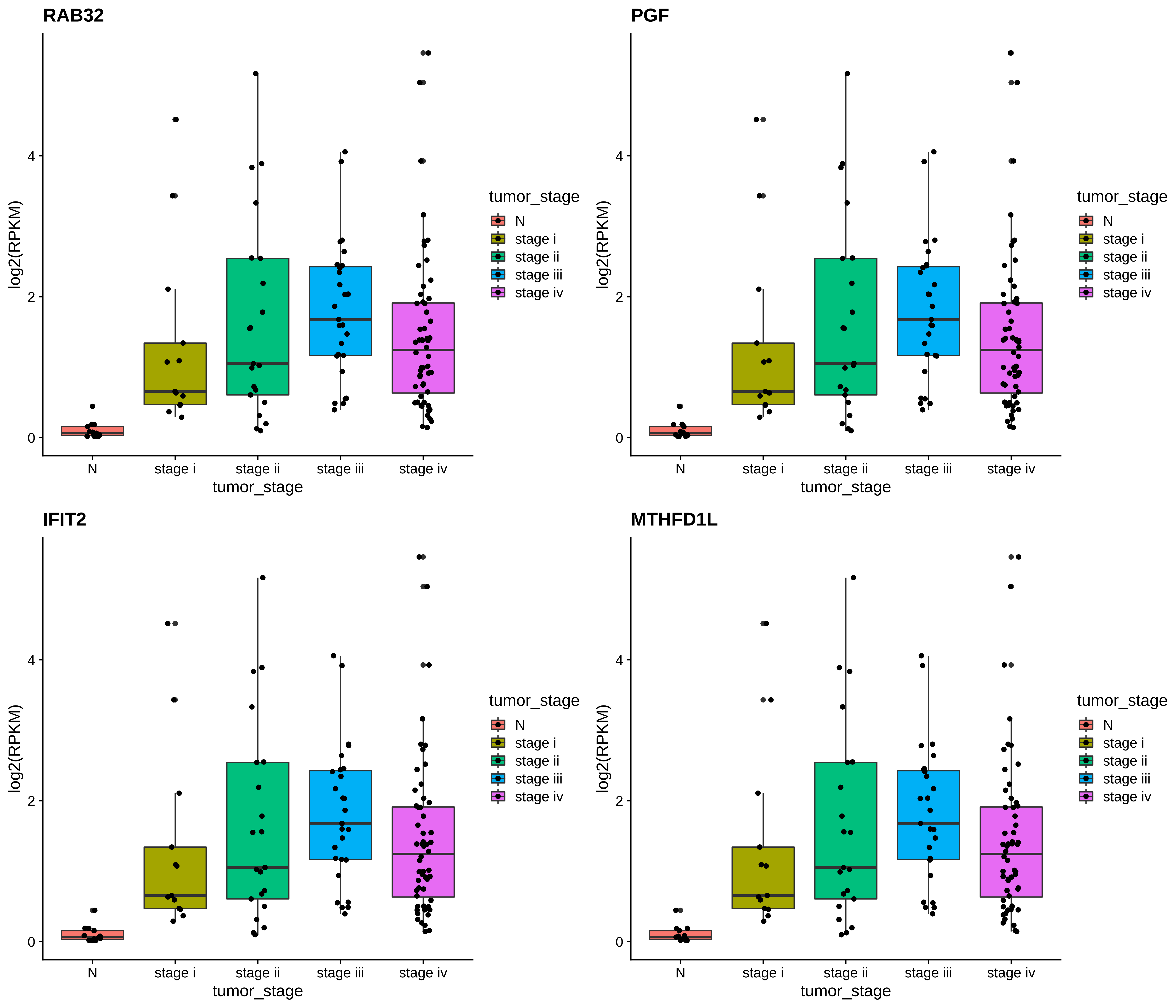
RAB32
PGF
IFIT2
MTHFD1L
log2(RPKM)
0
2
4
N
stage i
stage ii
stage iii
stage iv
tumor_stage

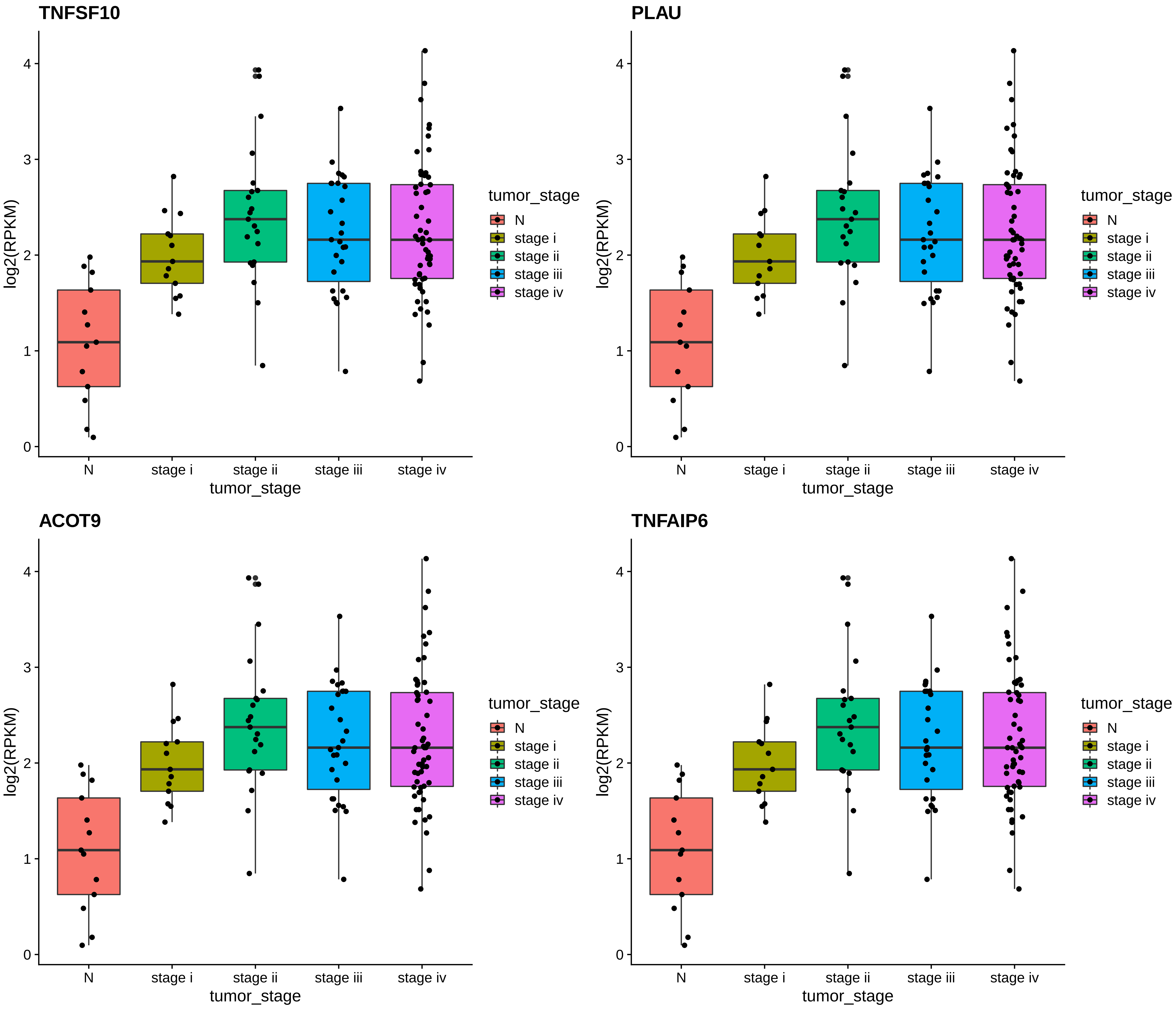
TNFSF10
PLAU
ACOT9
TNFAIP6
log2(RPKM)
0
1
2
3
4
N
stage i
stage ii
stage iii
stage iv
tumor_stage
tumor_stage
N
stage i
stage ii
stage iii
stage iv

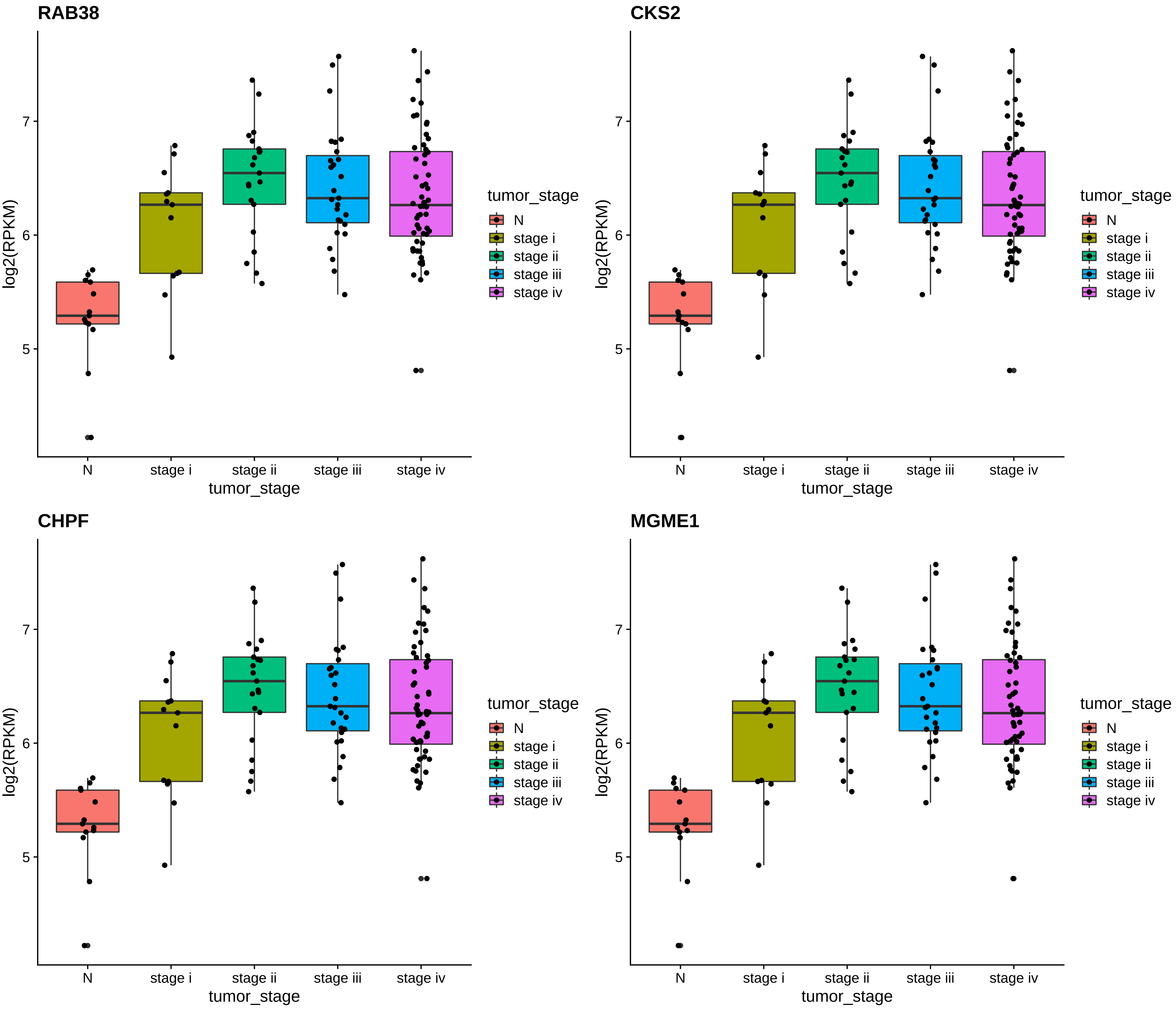
RAB38
CKS2
CHPF
MGME1
log2(RPKM)
7
6
5
N
stage i
stage ii
stage iii
stage iv
tumor_stage

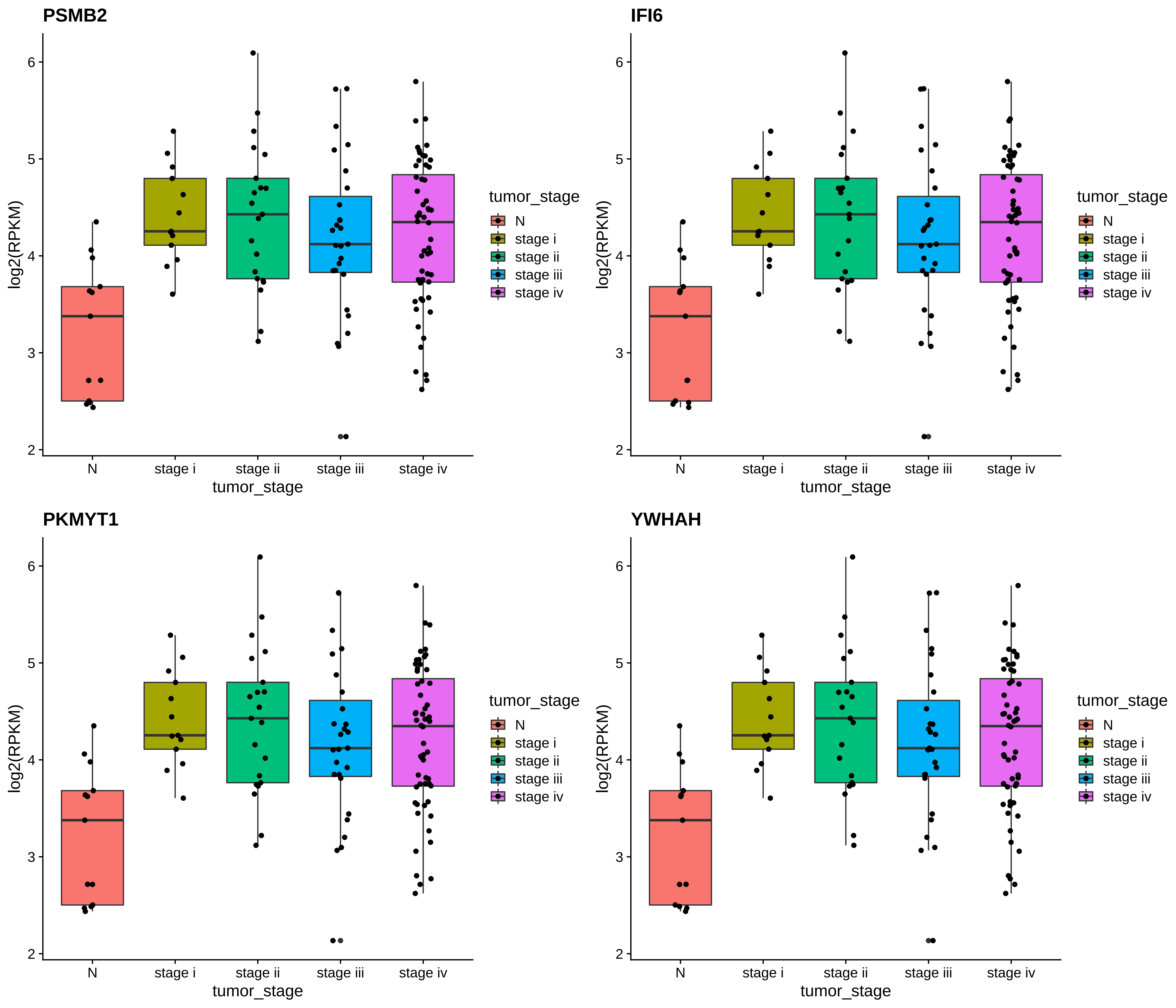

PSMB2
IFI6
PKMYT1
YWHAH
log2(RPKM)
tumor_stage
N
stage i
stage ii
stage iii
stage iv
2
3
4
5
6

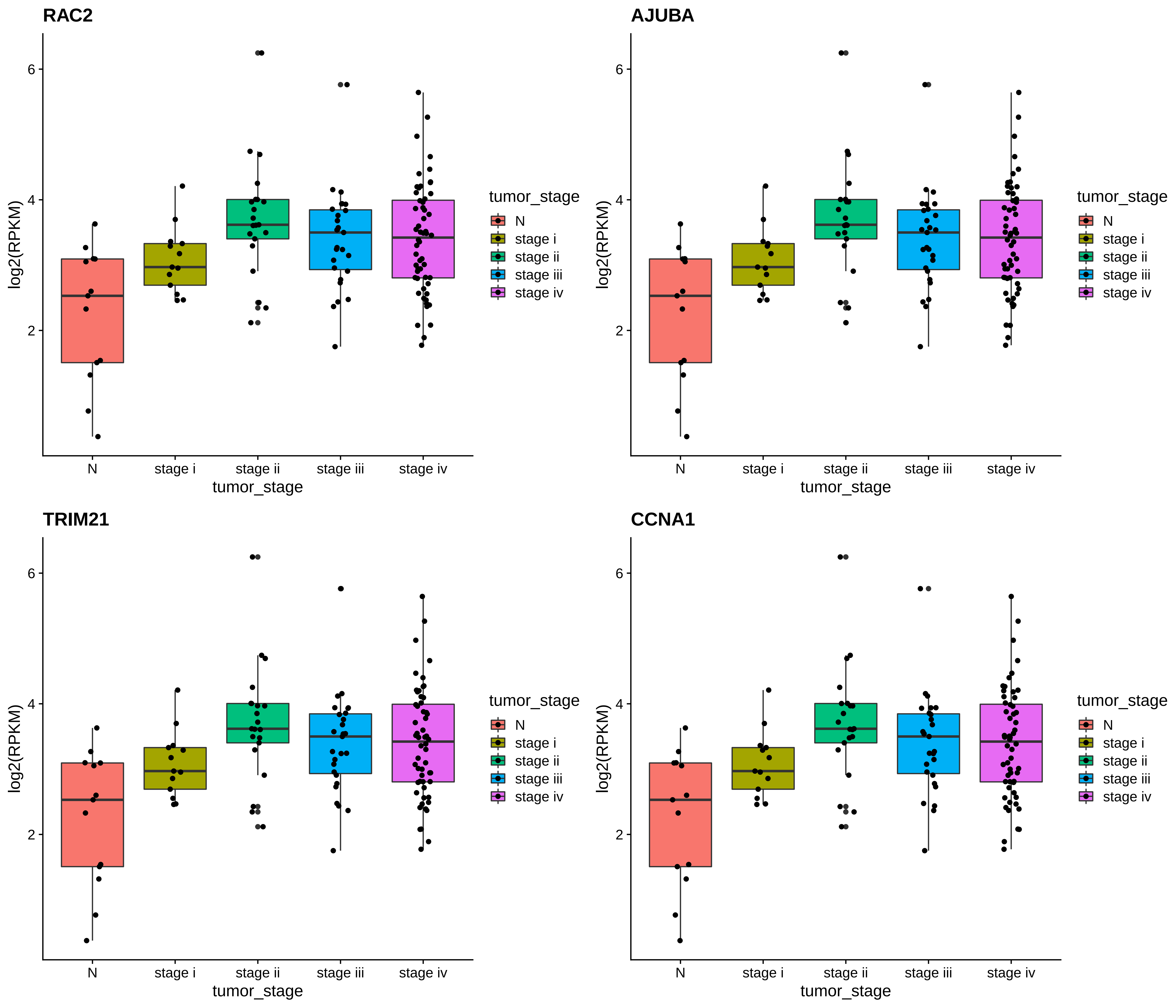

RAC2
AJUBA
TRIM21
CCNA1
log2(RPKM)
tumor_stage
N
stage i
stage ii
stage iii
stage iv

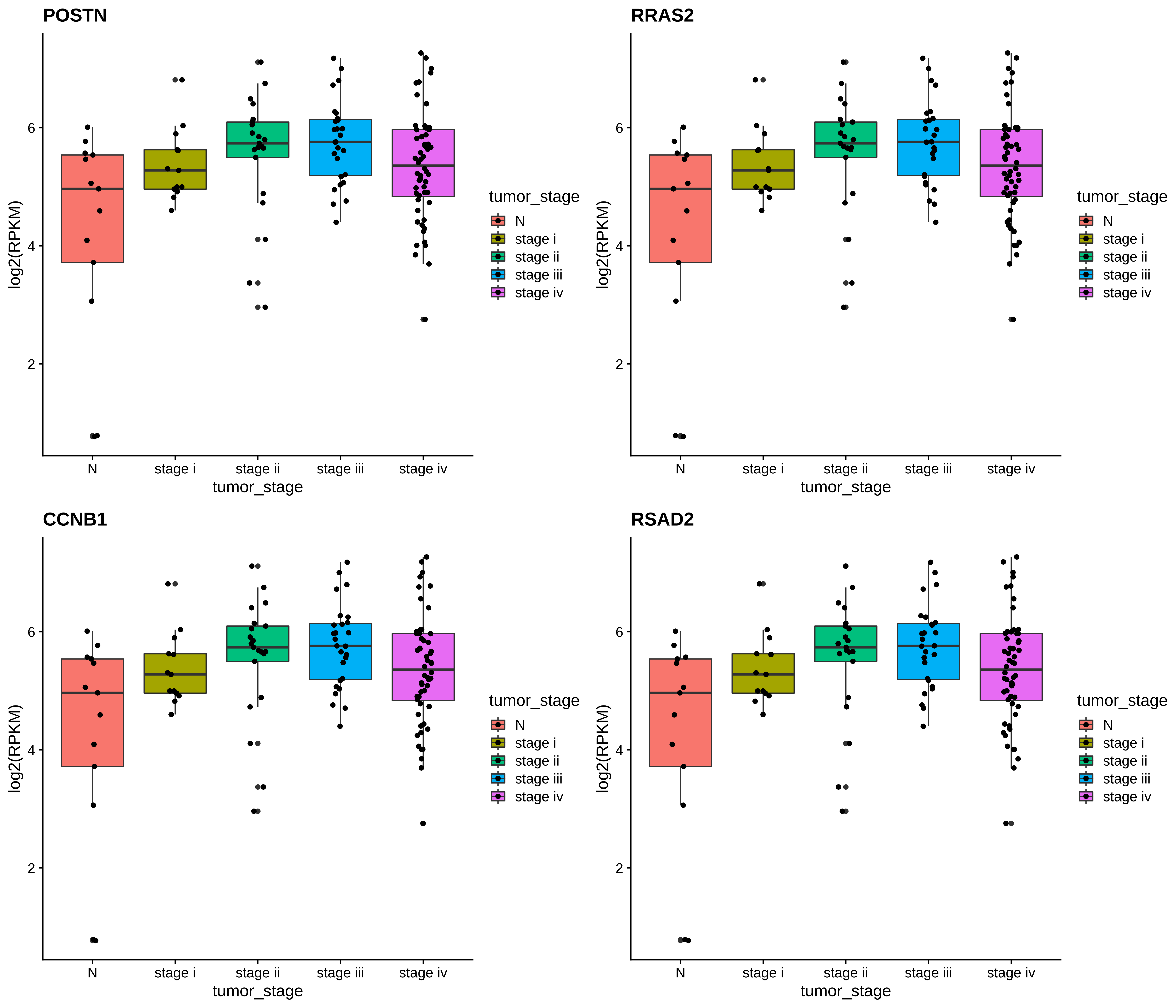
POSTN
RRAS2
CCNB1
RSAD2
log2(RPKM)
tumor_stage
N
stage i
stage ii
stage iii
stage iv
2
4
6

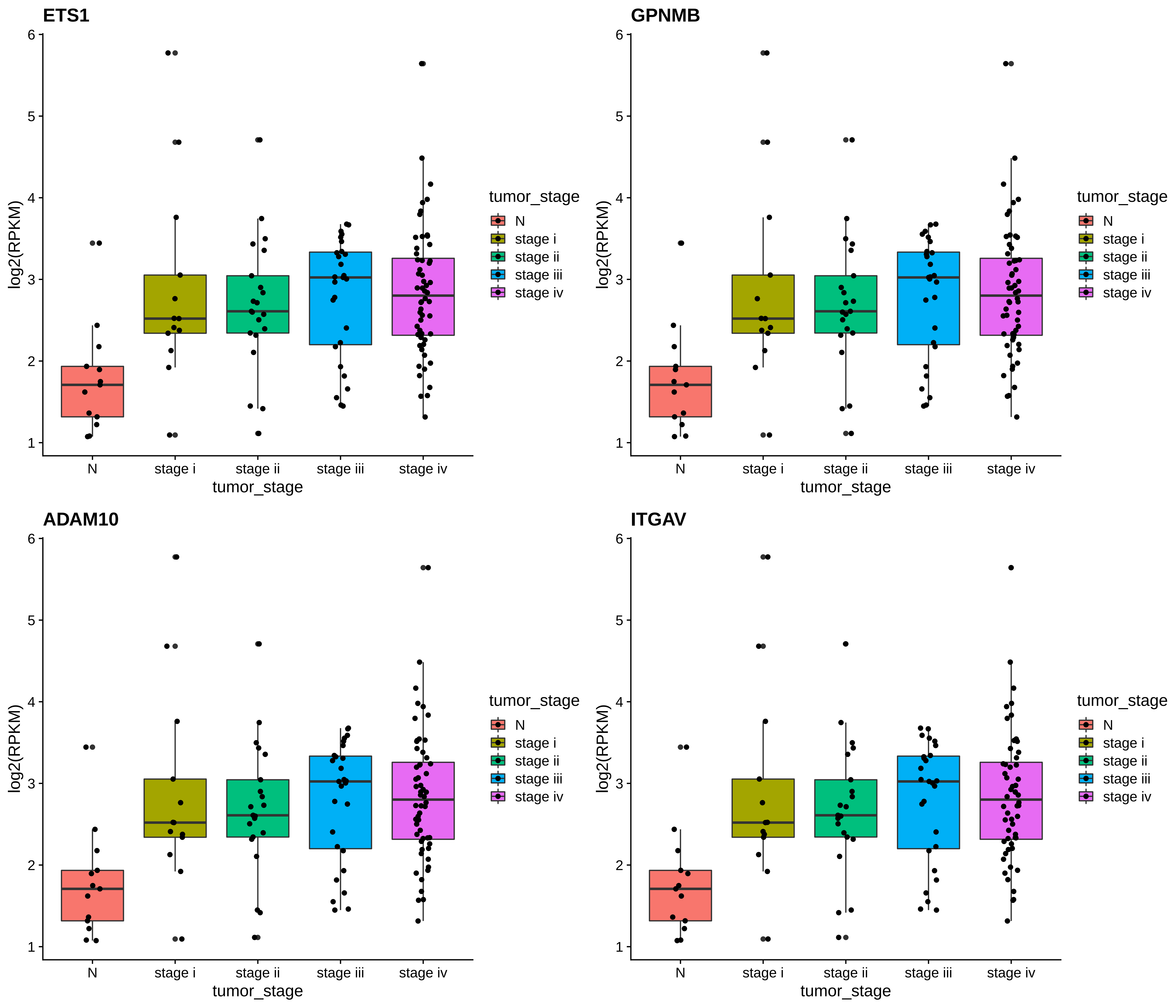
ETS1
GPNMB
ADAM10
ITGAV
log2(RPKM)
1
2
3
4
5
6
N
stage i
stage ii
stage iii
stage iv
tumor_stage

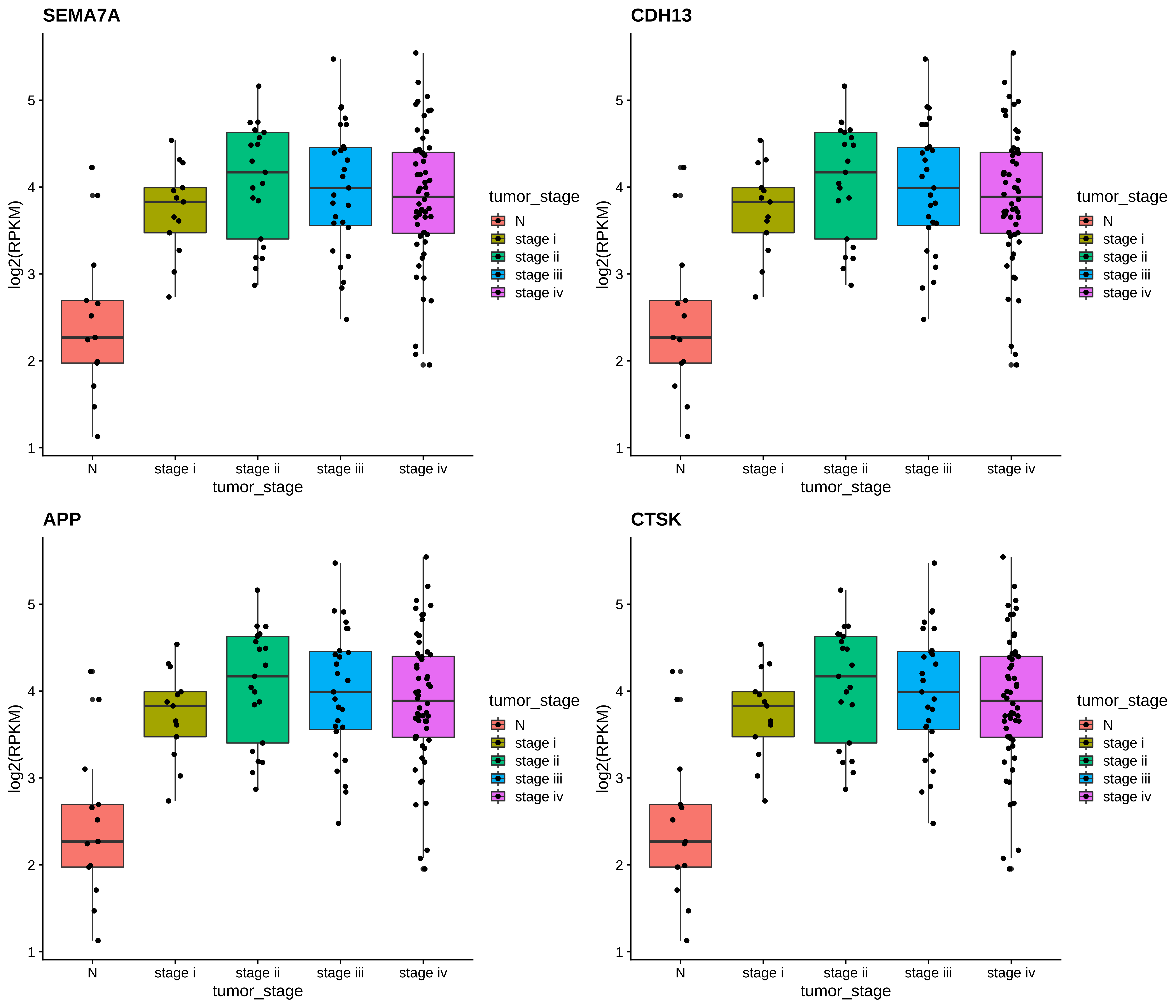
SEMA7A
CDH13
APP
CTSK
log2(RPKM)
tumor_stage
N
stage i
stage ii
stage iii
stage iv
1
2
3
4
5

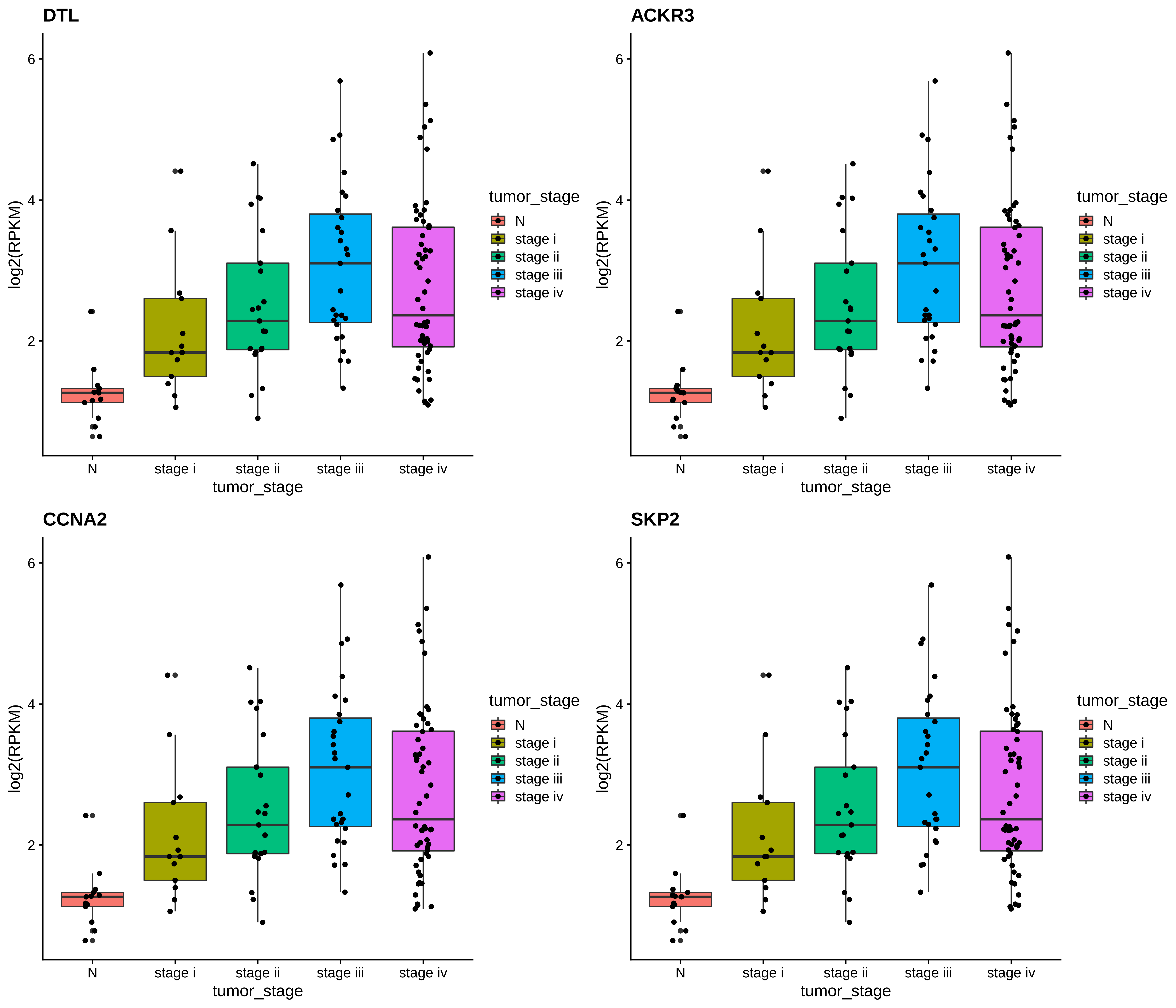
DTL
ACKR3
CCNA2
SKP2
log2(RPKM)
tumor_stage
N
stage i
stage ii
stage iii
stage iv
2
4
6

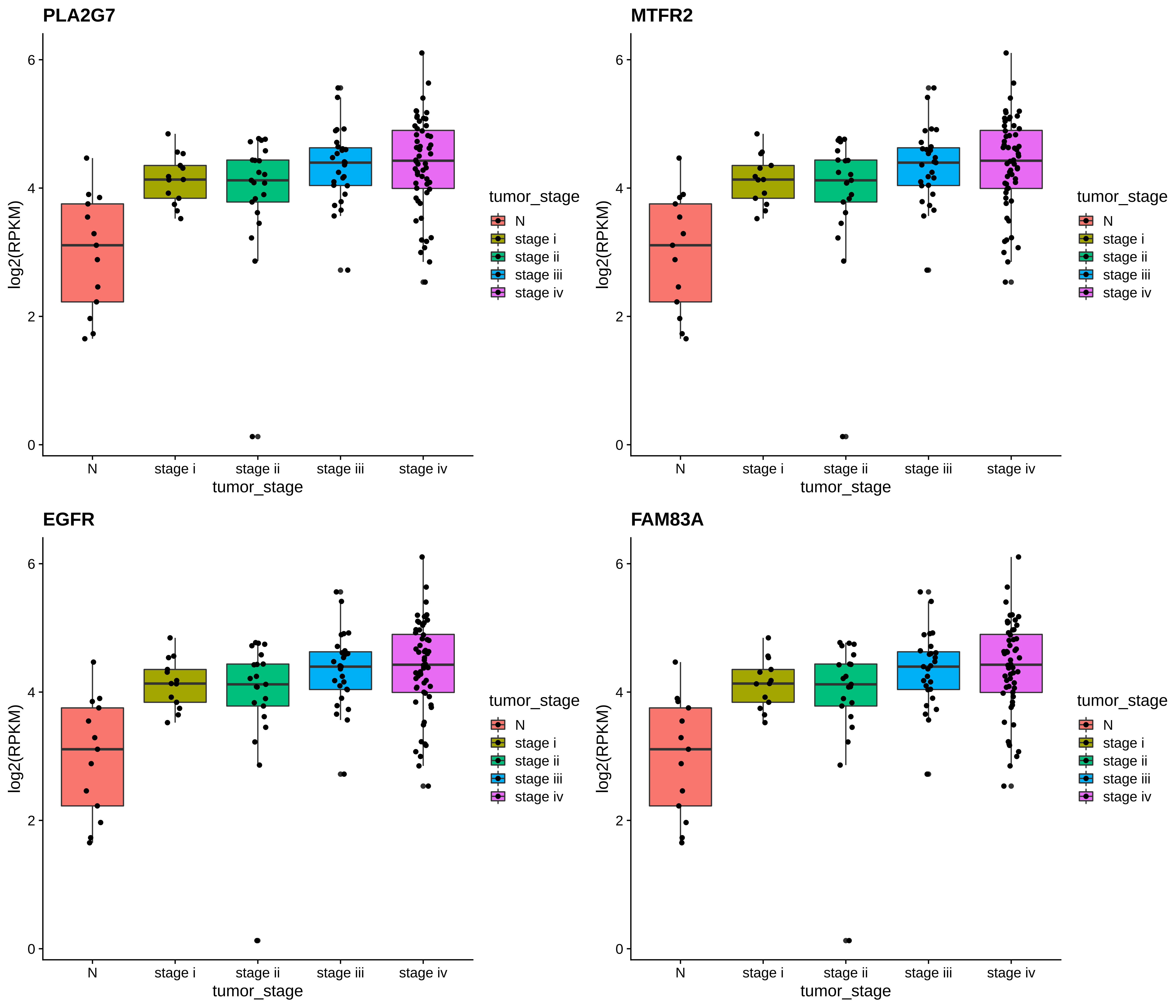
PLA2G7
MTFR2
EGFR
FAM83A
log2(RPKM)
tumor_stage
N
stage i
stage ii
stage iii
stage iv
0
2
4
6

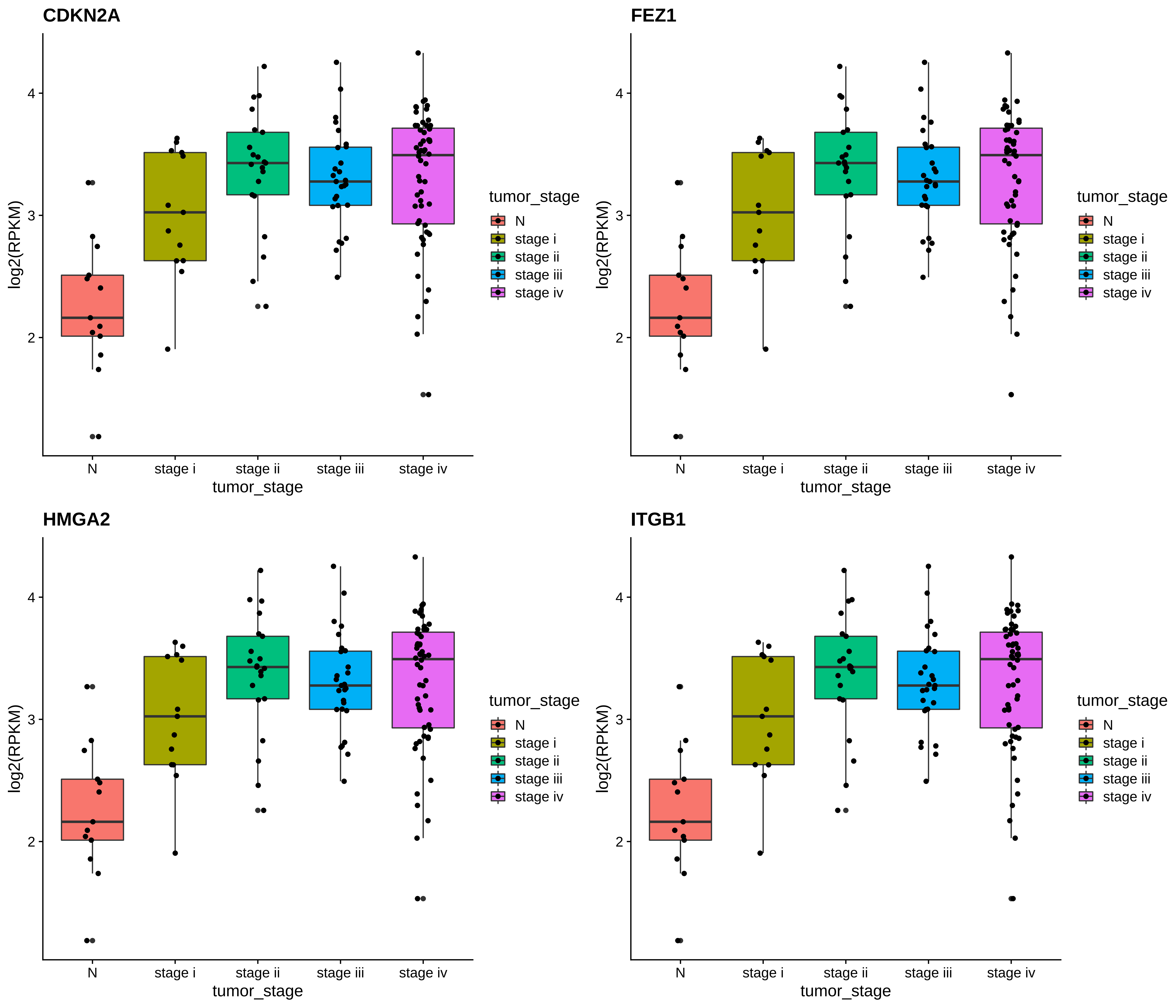
CDKN2A
FEZ1
HMGA2
ITGB1
log2(RPKM)
tumor_stage
N
stage i
stage ii
stage iii
stage iv
2
3
4

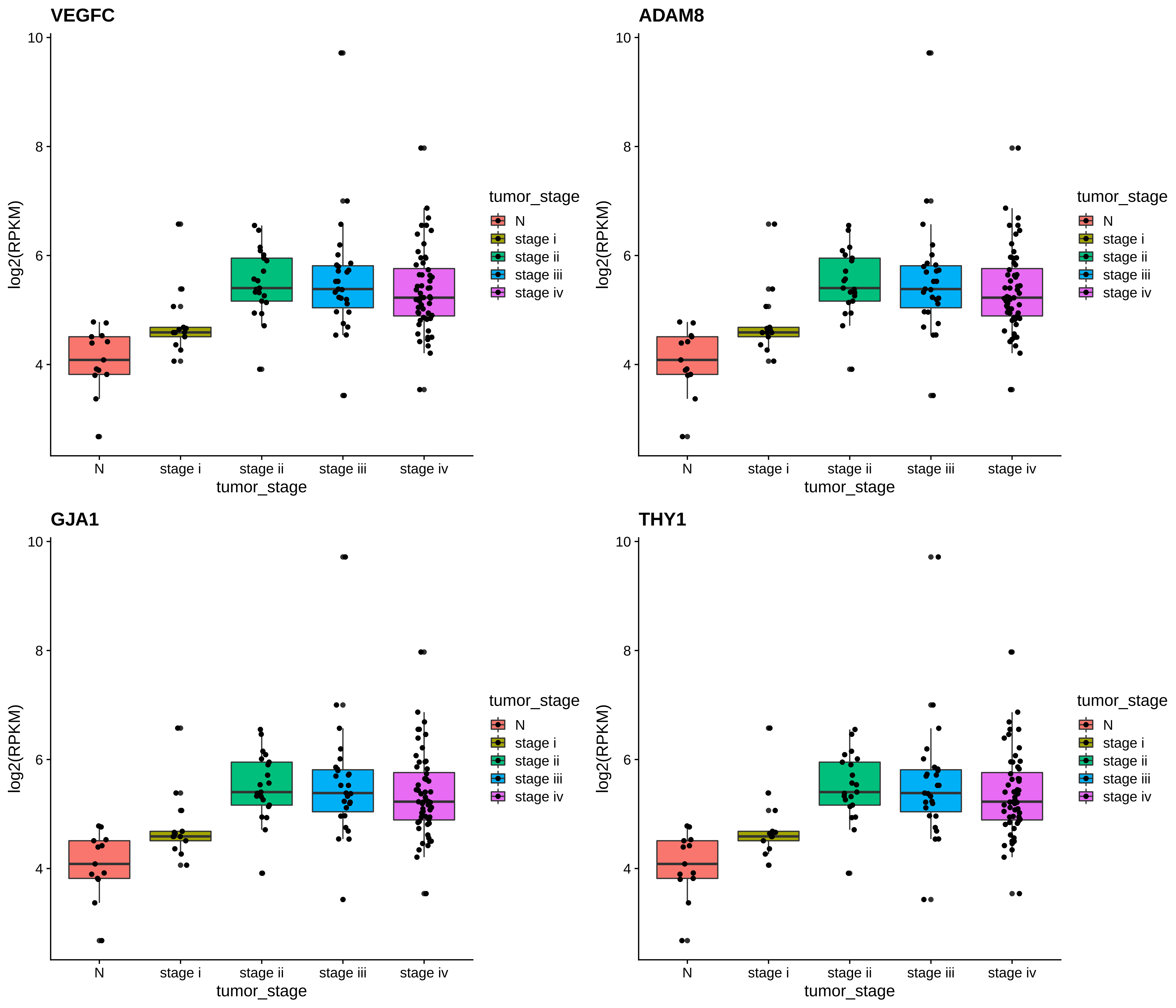
VEGFC
ADAM8
GJA1
THY1
log2(RPKM)
tumor_stage
N
stage i
stage ii
stage iii
stage iv
10
8
6
4

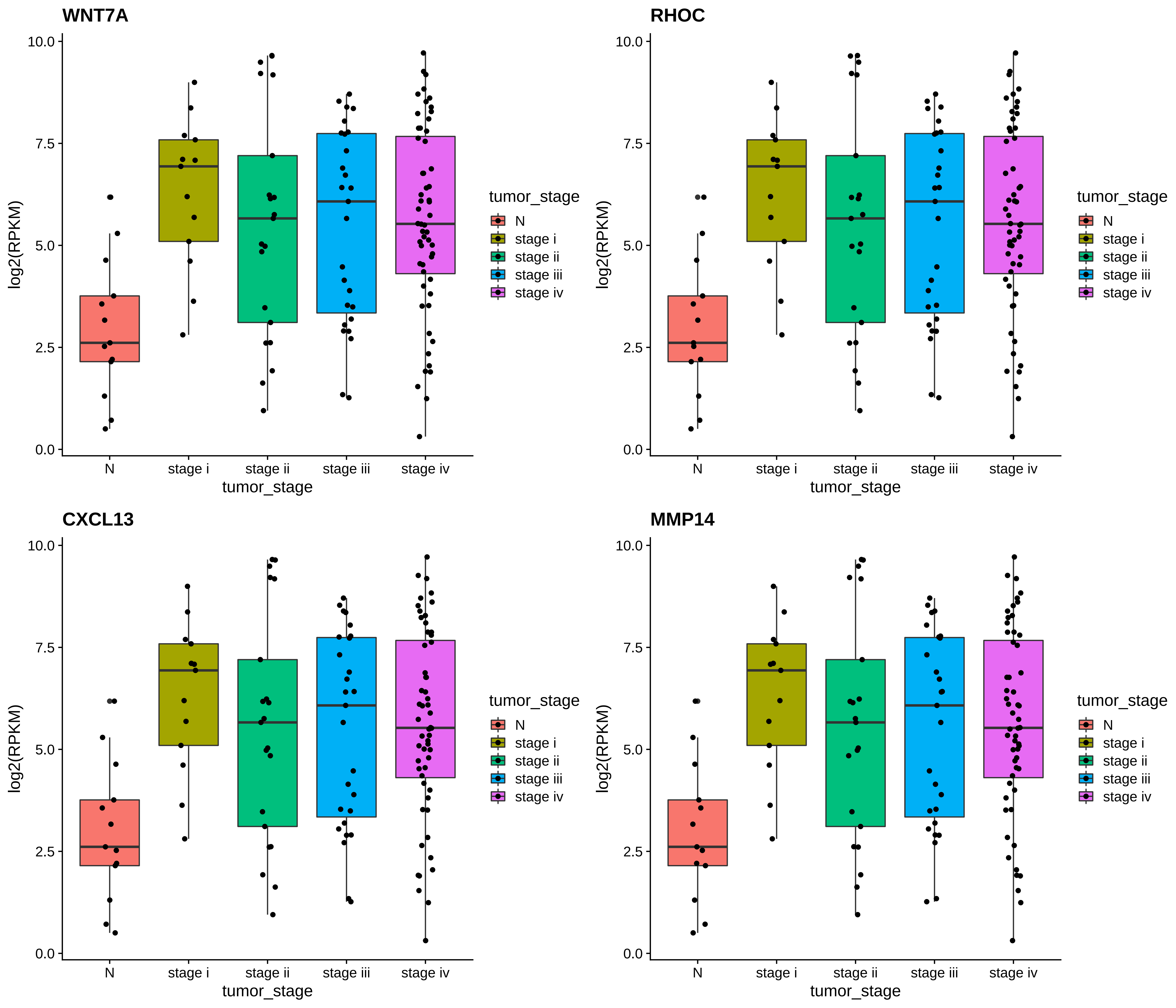

WNT7A
RHOC
CXCL13
MMP14
log2(RPKM)
0.0
2.5
5.0
7.5
10.0
N
stage i
stage ii
stage iii
stage iv
tumor_stage

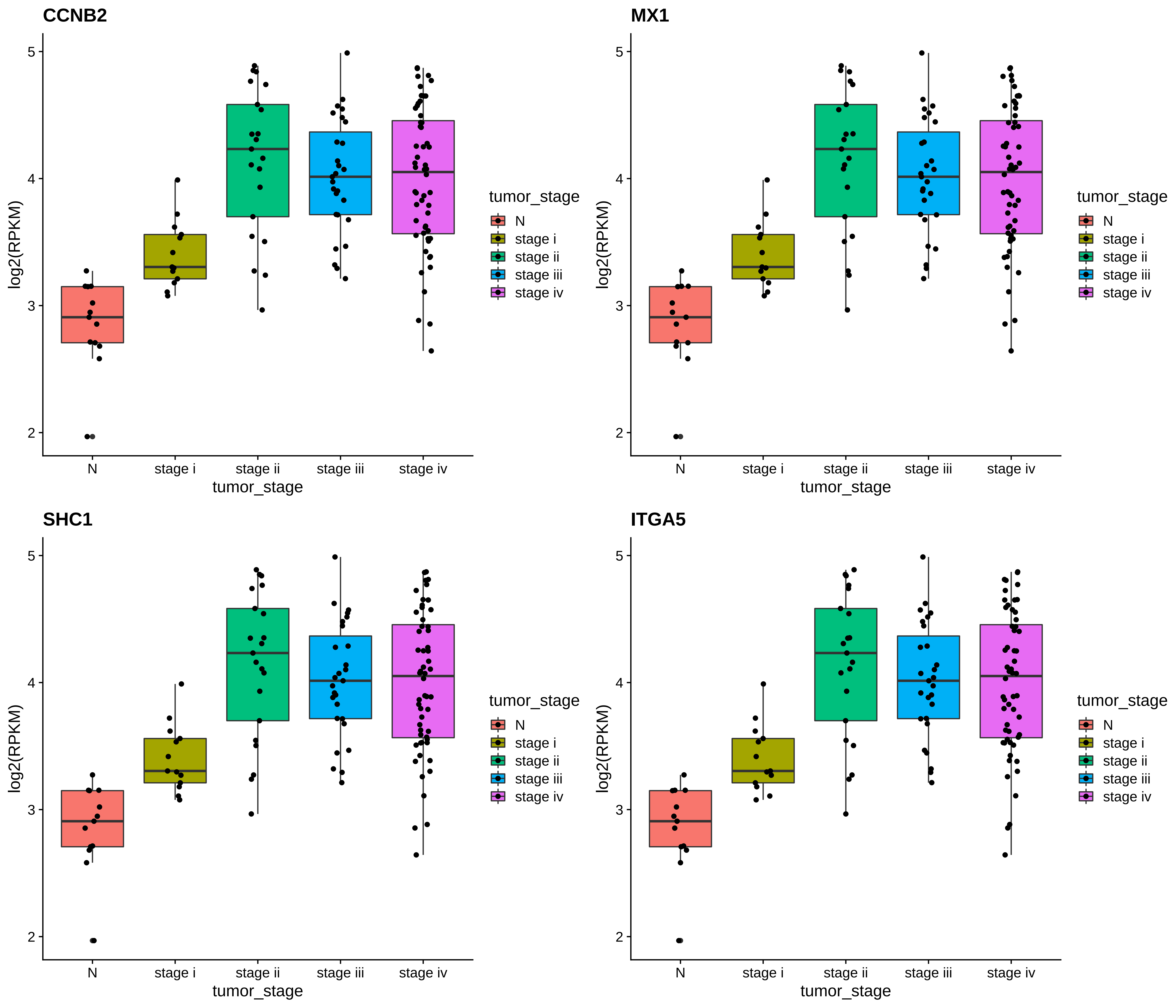

CCNB2
MX1
SHC1
ITGA5
log2(RPKM)
tumor_stage
N
stage i
stage ii
stage iii
stage iv
2
3
4
5

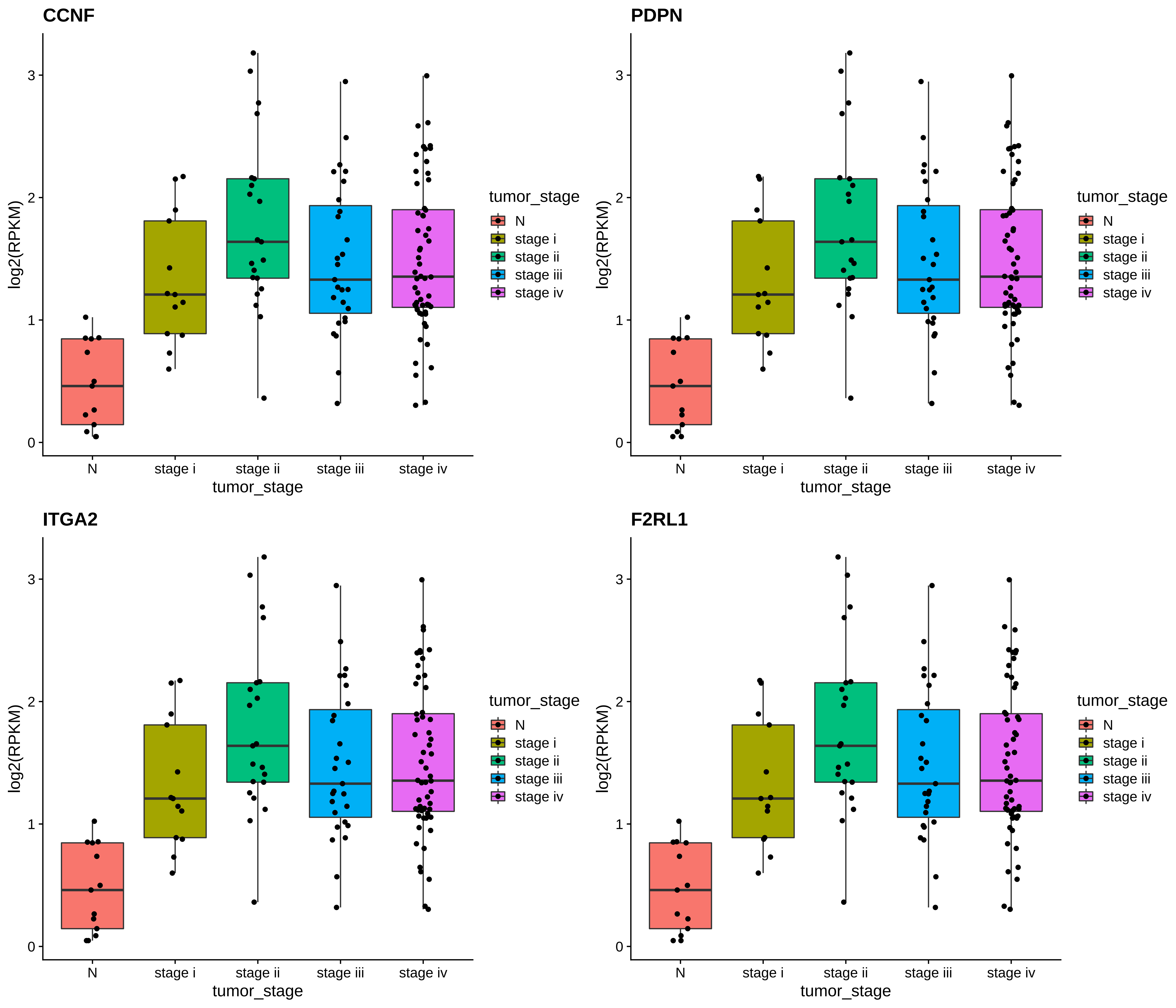
CCNF
PDPN
ITGA2
F2RL1
log2(RPKM)
0
1
2
3
N
stage i
stage ii
stage iii
stage iv
tumor_stage

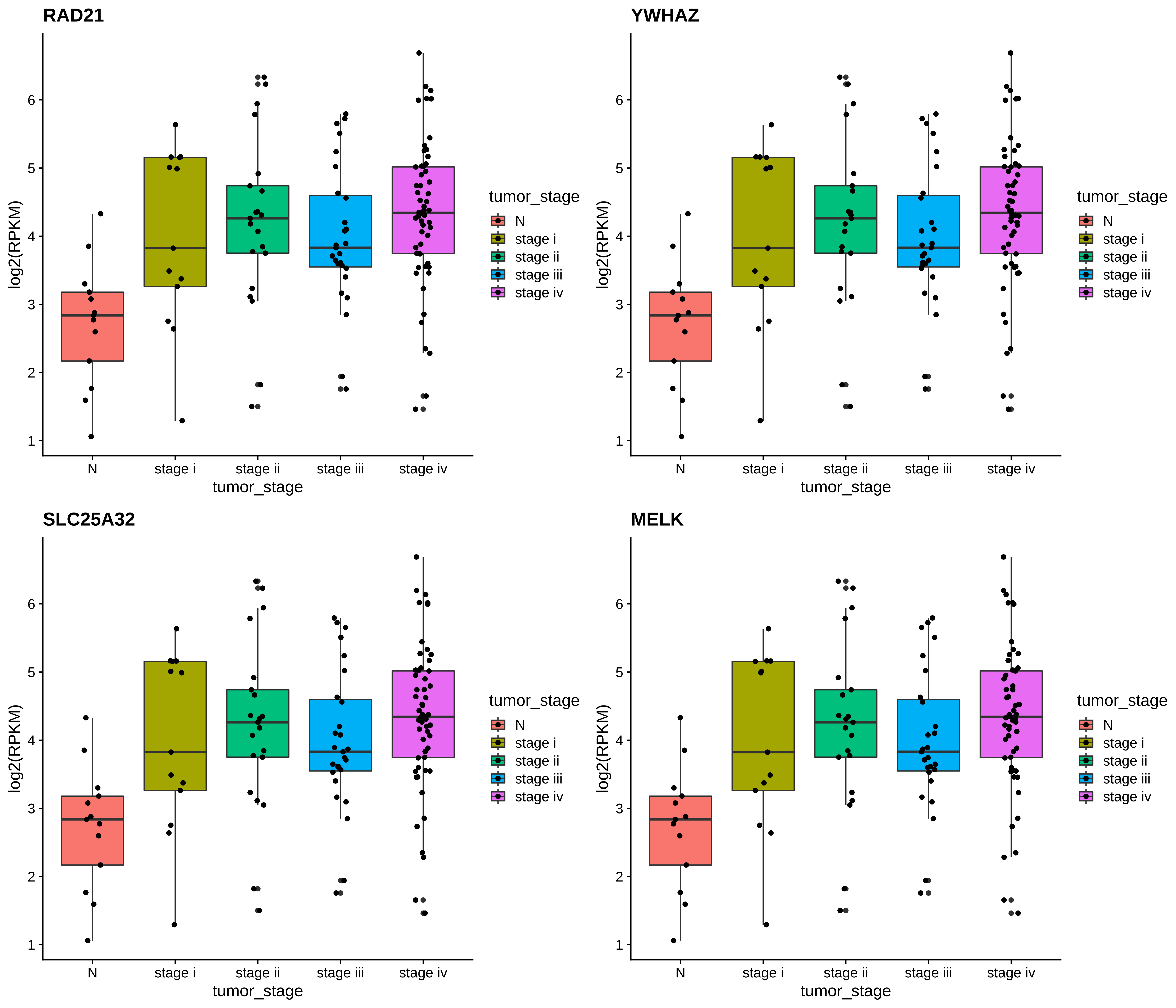
RAD21
YWHAZ
SLC25A32
MELK
log2(RPKM)
tumor_stage
N
stage i
stage ii
stage iii
stage iv
1
2
3
4
5
6

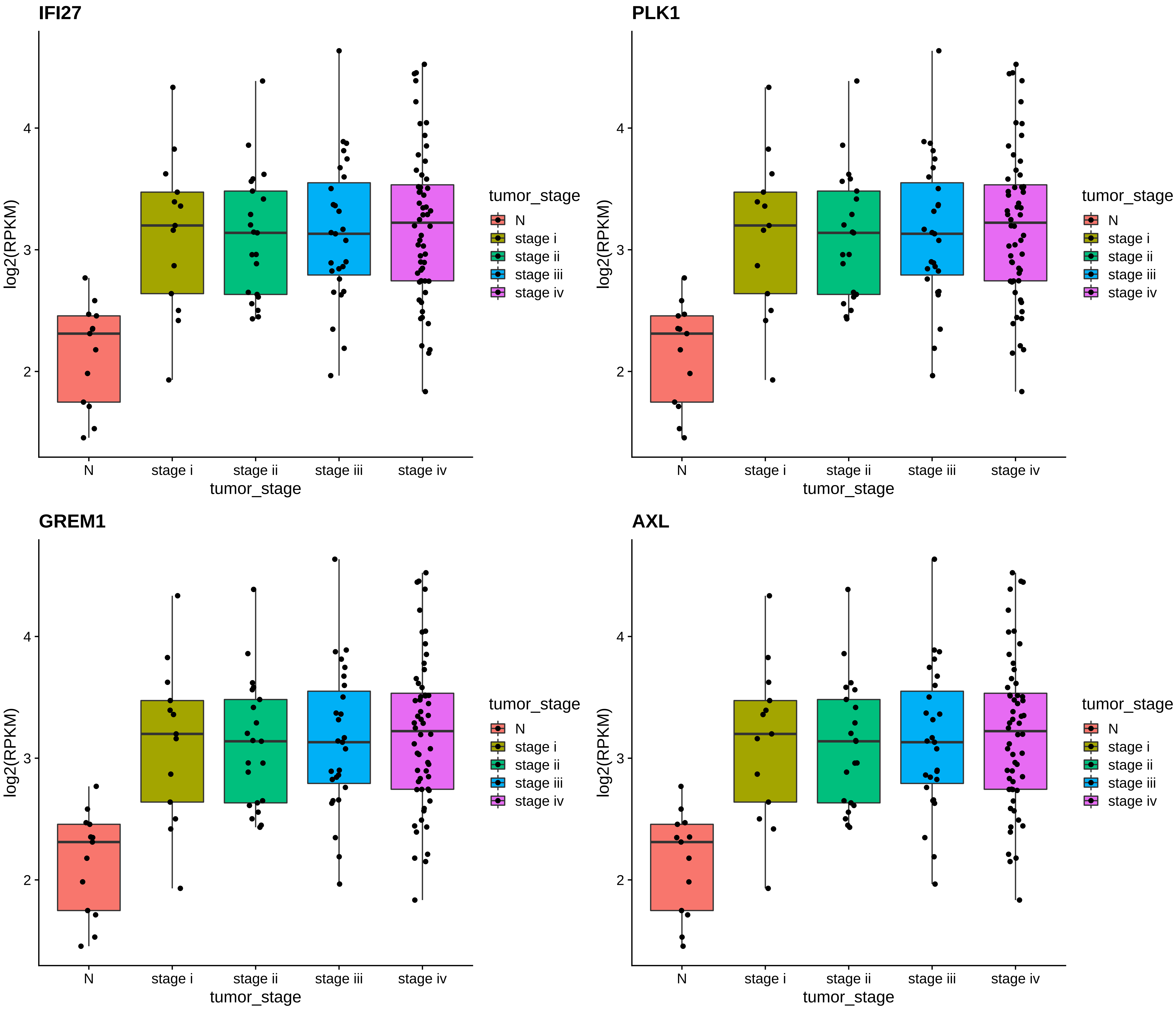
IFI27
PLK1
GREM1
AXL
log2(RPKM)
2
3
4
N
stage i
stage ii
stage iii
stage iv
tumor_stage
tumor_stage
N
stage i
stage ii
stage iii
stage iv

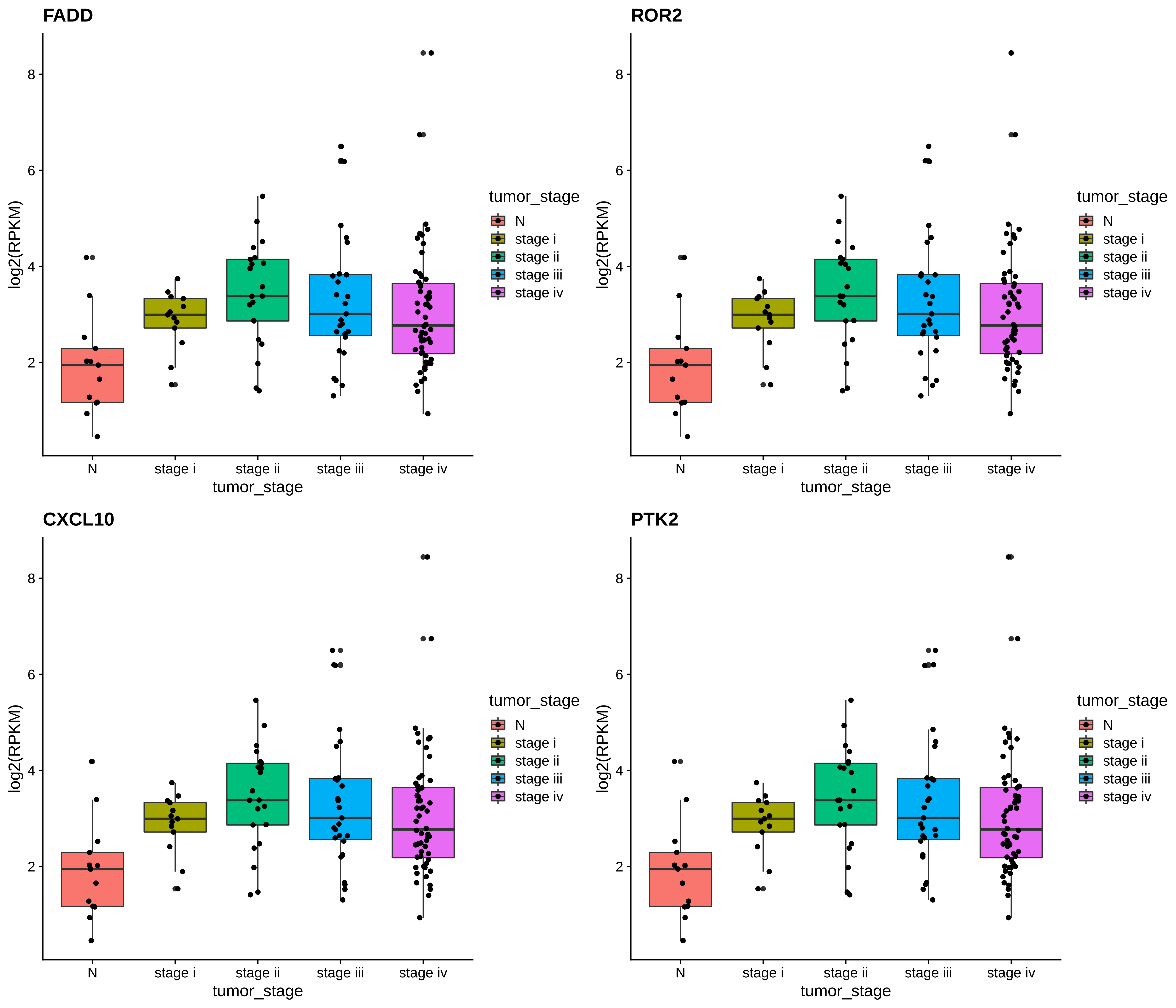
FADD
ROR2
CXCL10
PTK2
log2(RPKM)
tumor_stage
N
stage i
stage ii
stage iii
stage iv
2
4
6
8

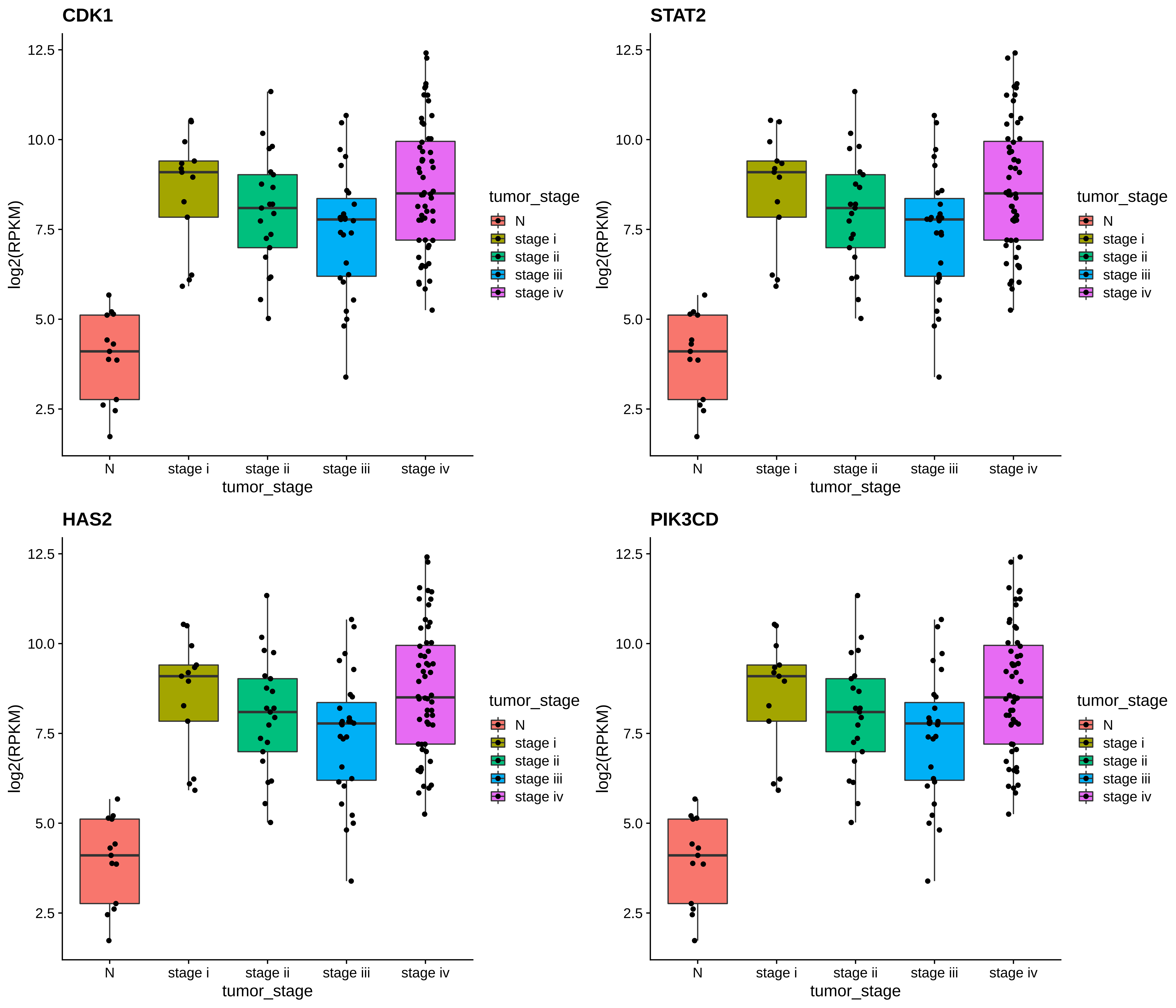
CDK1
STAT2
HAS2
PIK3CD
log2(RPKM)
12.5
10.0
7.5
5.0
2.5
N
stage i
stage ii
stage iii
stage iv
tumor_stage

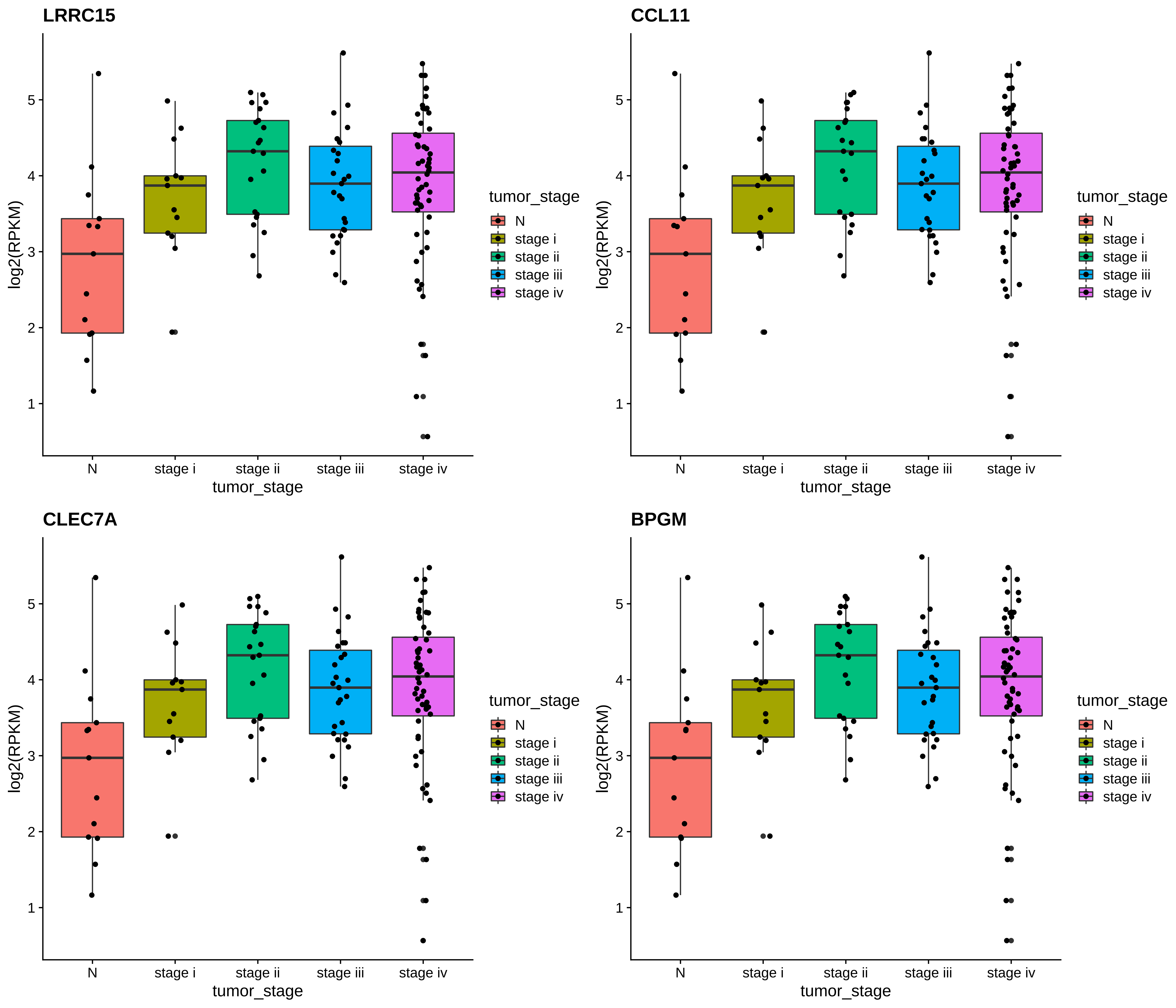

LRRC15
CCL11
CLEC7A
BPGM
log2(RPKM)
1
2
3
4
5
N
stage i
stage ii
stage iii
stage iv
tumor_stage

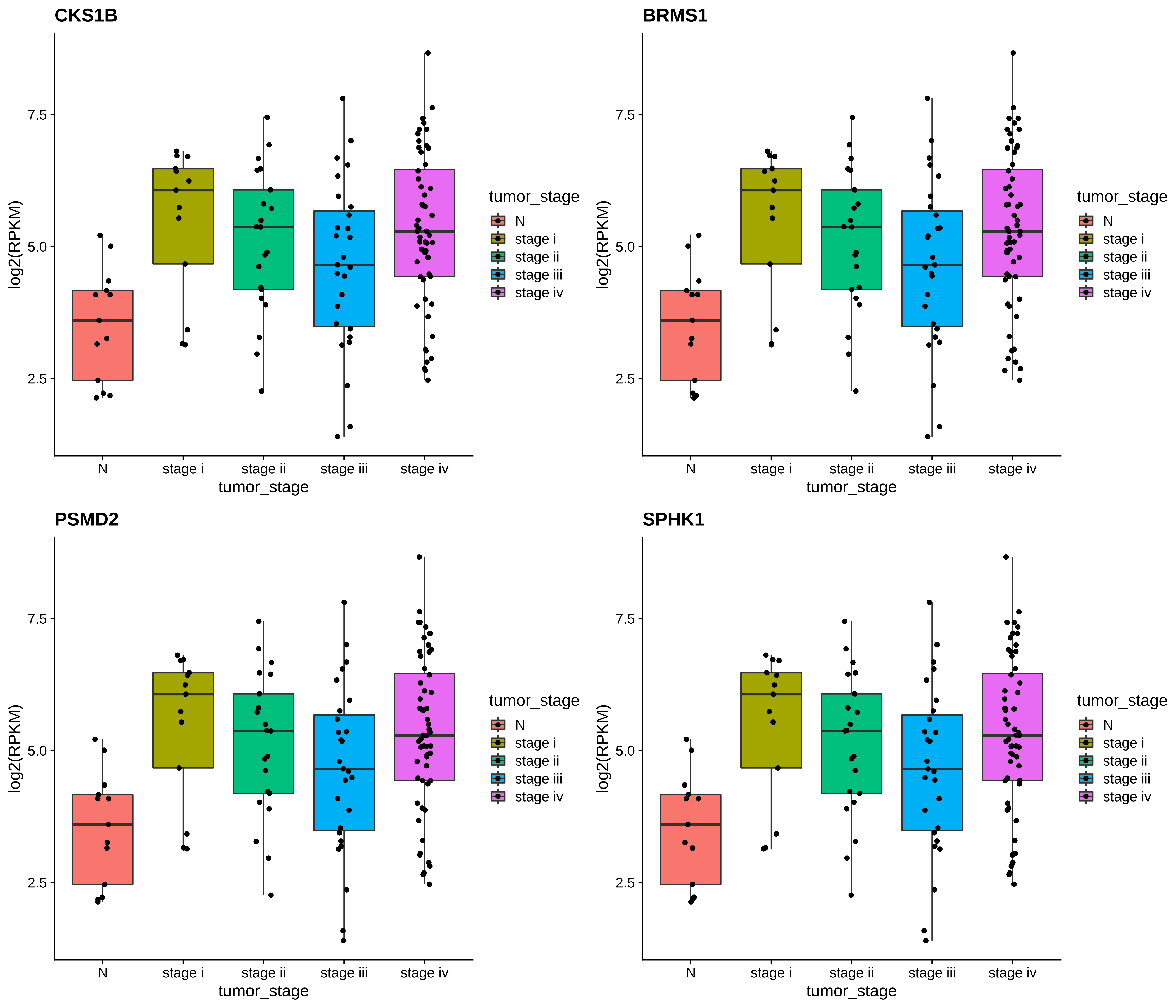

CKS1B
BRMS1
PSMD2
SPHK1
log2(RPKM)
2.5
5.0
7.5
N
stage i
stage ii
stage iii
stage iv
tumor_stage

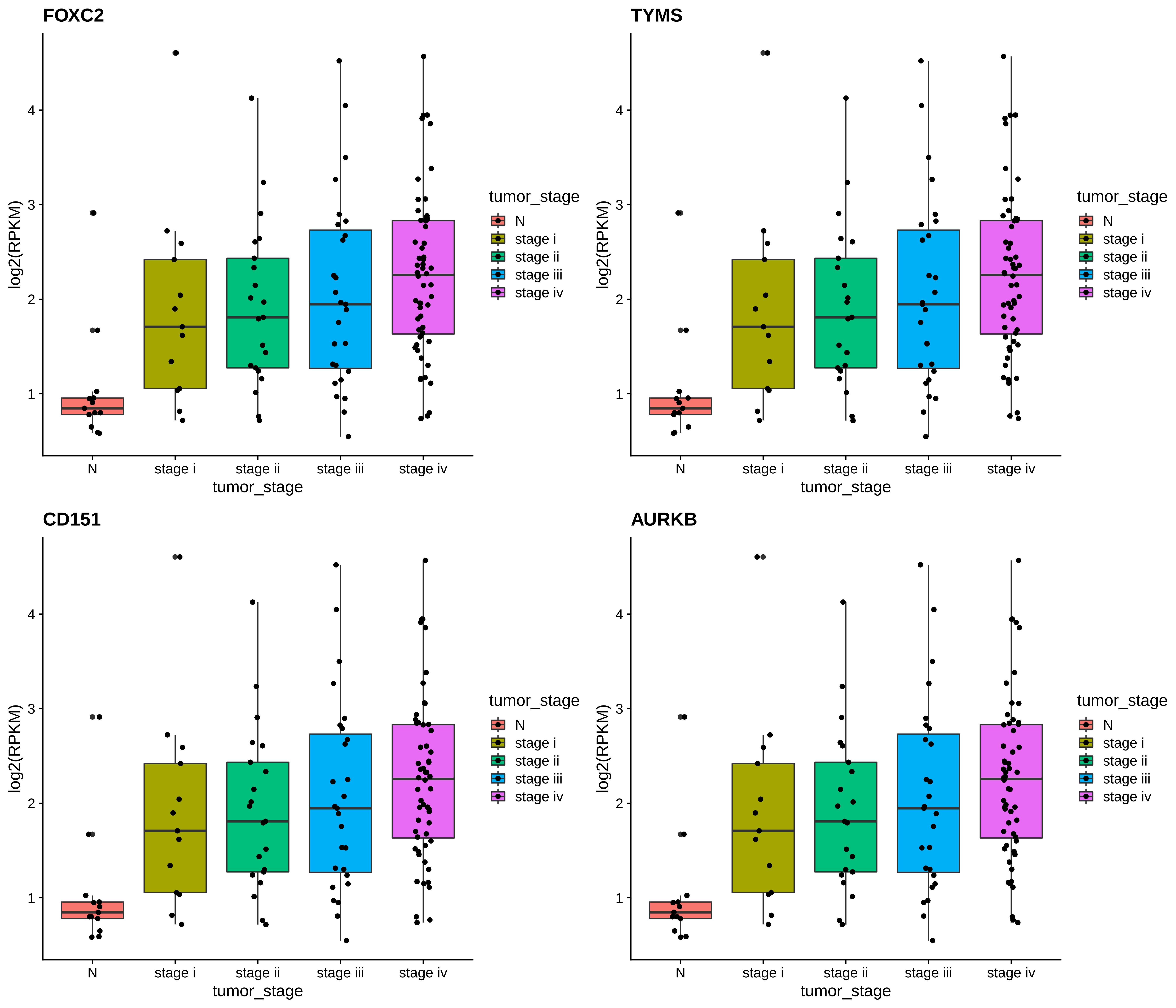
FOXC2
TYMS
CD151
AURKB
log2(RPKM)
tumor_stage
N
stage i
stage ii
stage iii
stage iv
1
2
3
4

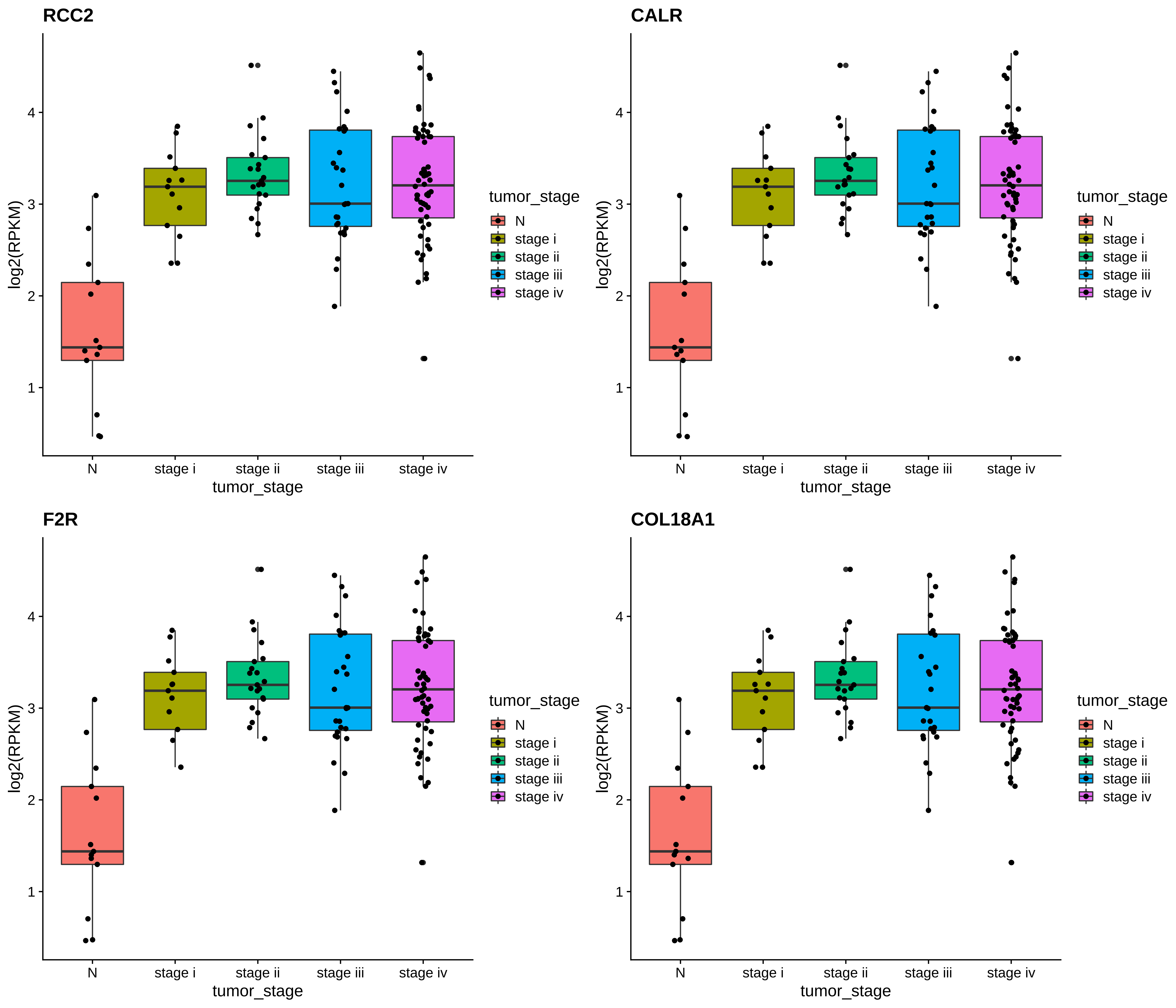
RCC2
CALR
F2R
COL18A1
log2(RPKM)
tumor_stage
N
stage i
stage ii
stage iii
stage iv
1
2
3
4

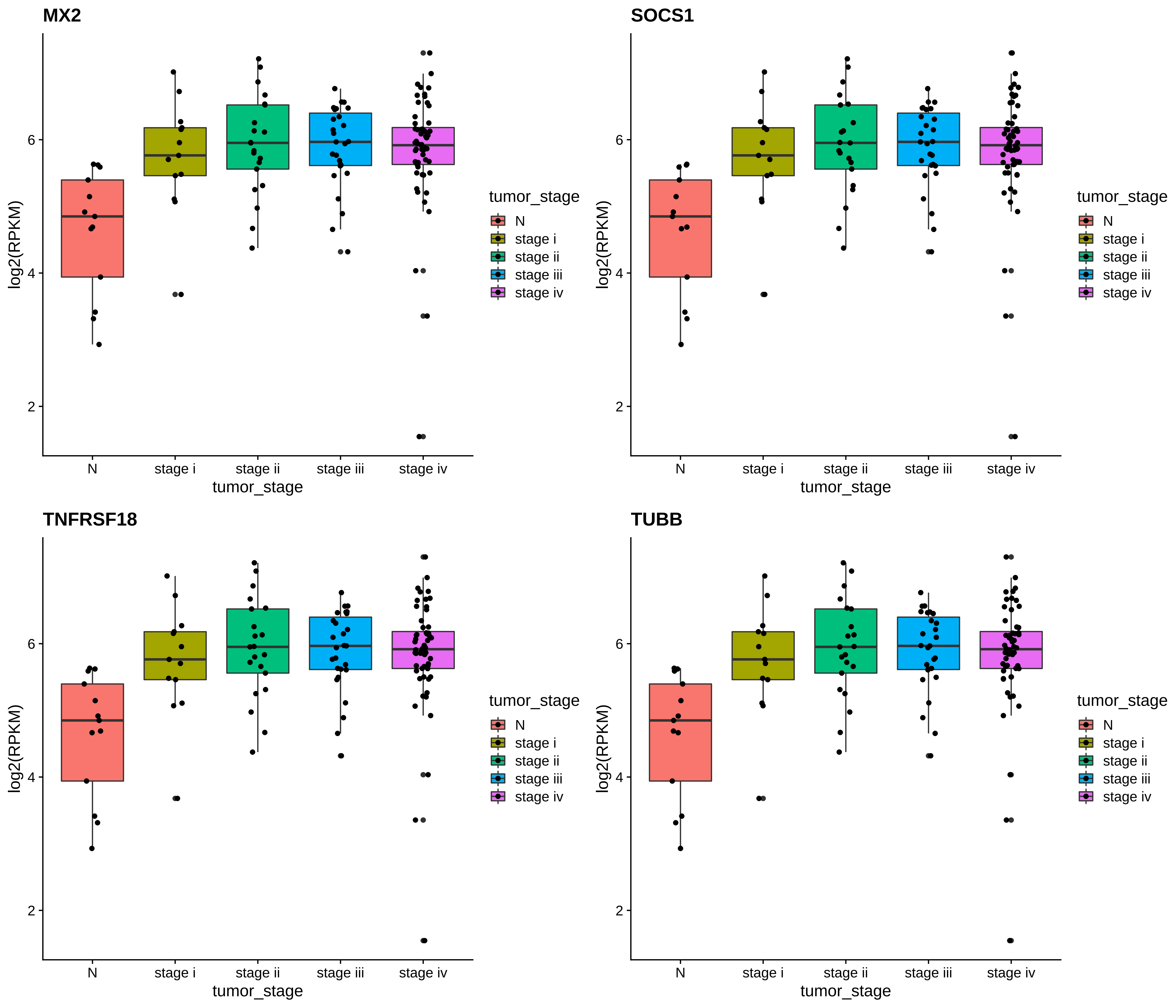
MX2
SOCS1
TNFRSF18
TUBB
log2(RPKM)
tumor_stage
N
stage i
stage ii
stage iii
stage iv
2
4
6

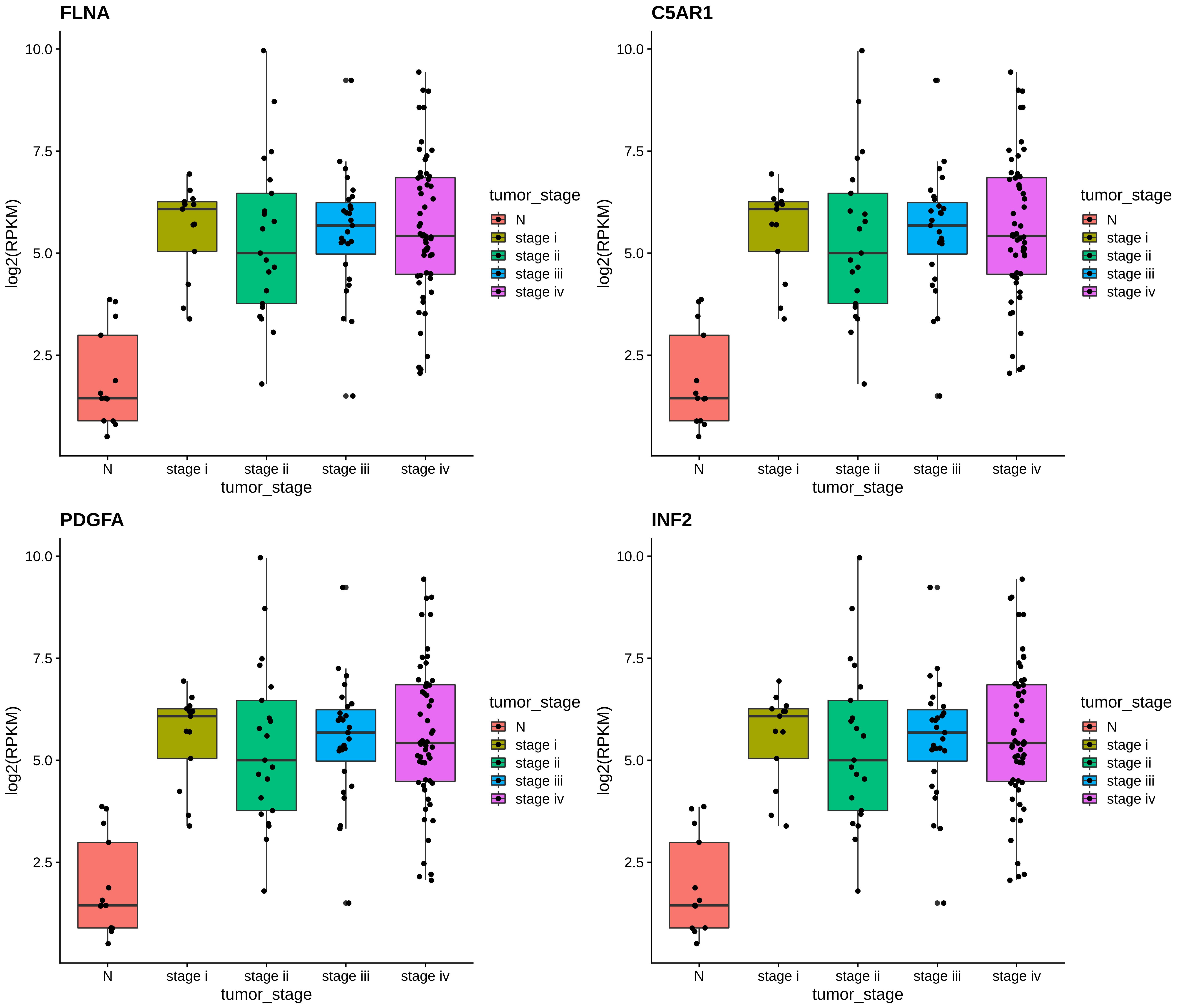
FLNA
C5AR1
PDGFA
INF2
log2(RPKM)
10.0
7.5
5.0
2.5
N
stage i
stage ii
stage iii
stage iv
tumor_stage

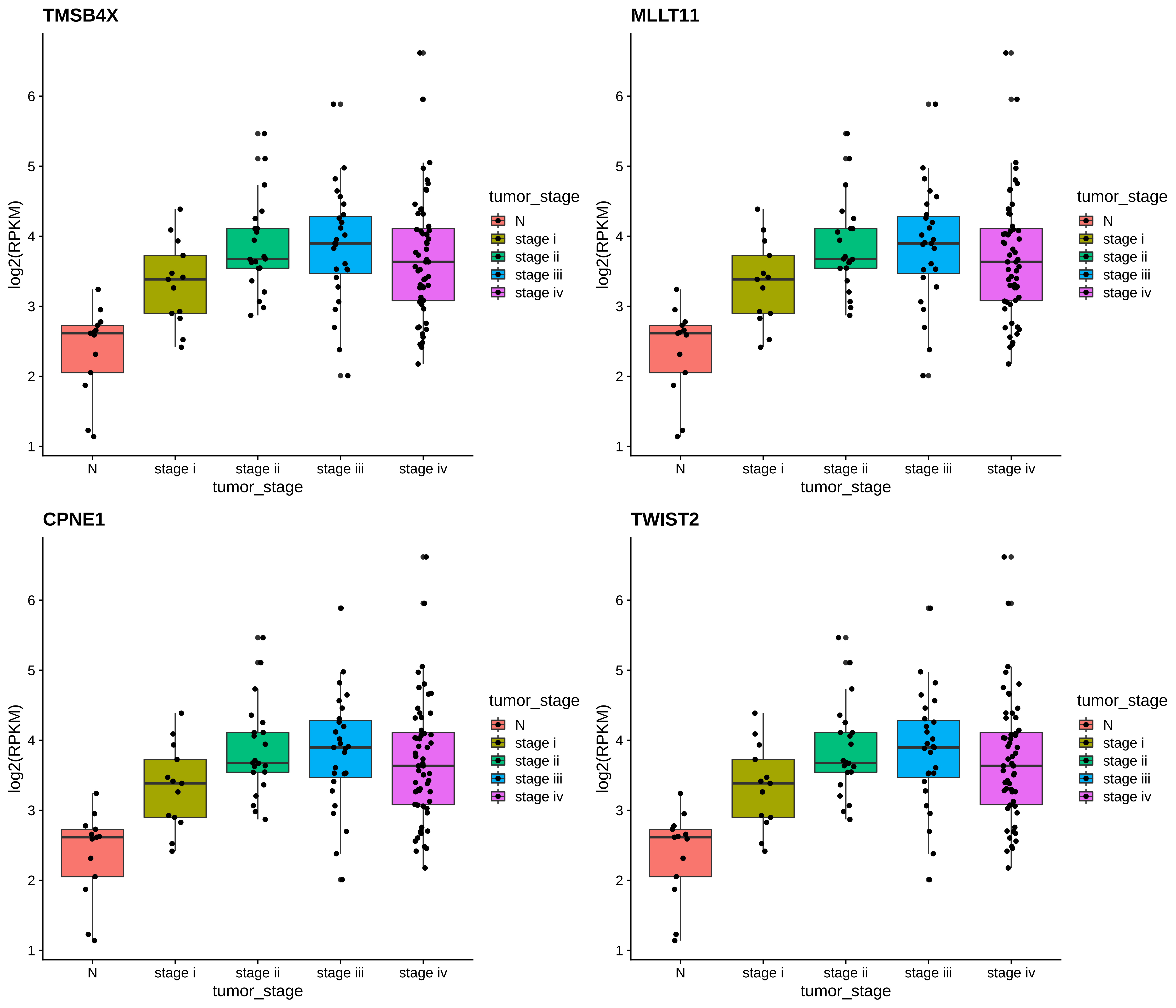
TMSB4X
MLLT11
CPNE1
TWIST2
log2(RPKM)
1
2
3
4
5
6
tumor_stage
N
stage i
stage ii
stage iii
stage iv

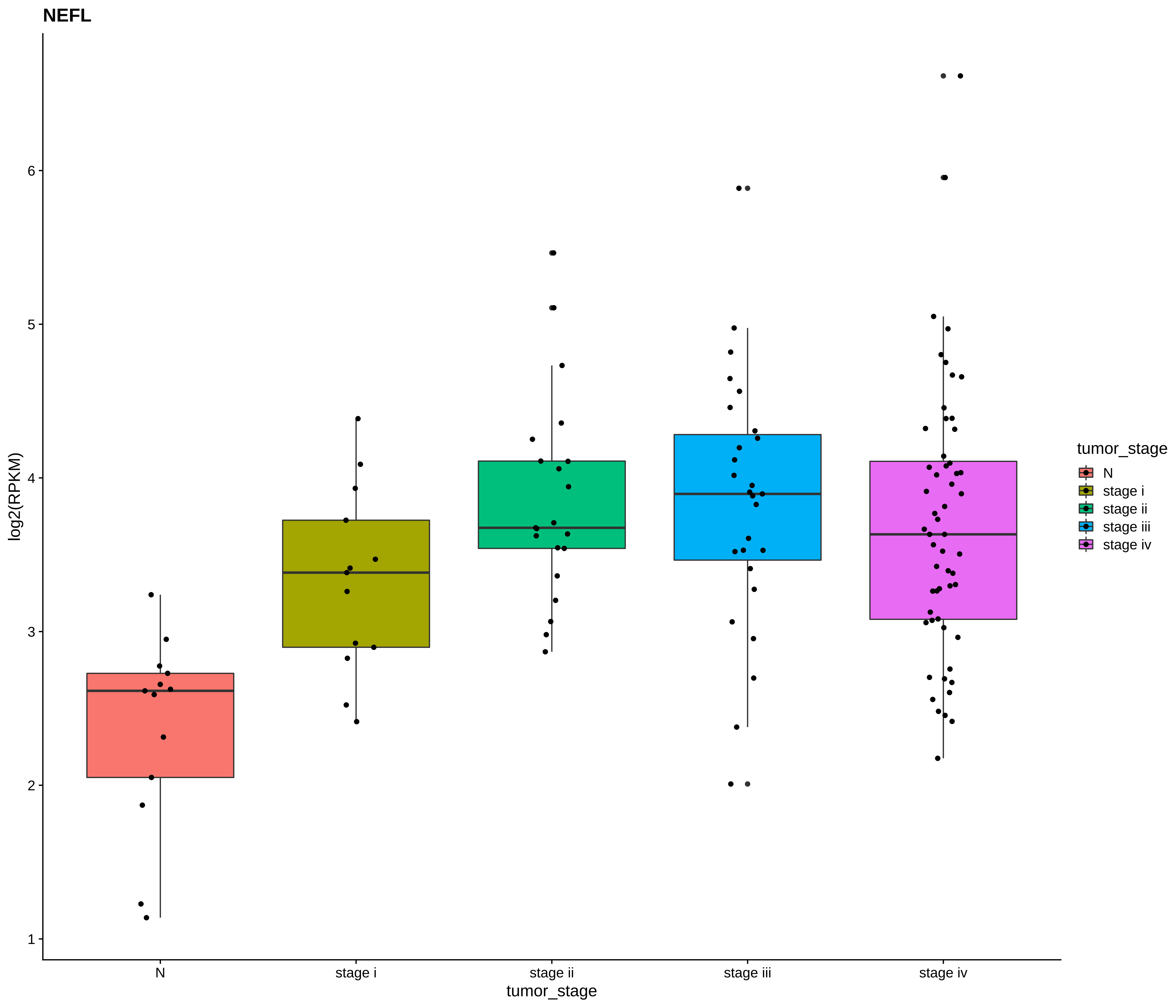

NEFL
log2(RPKM)
1
2
3
4
5
6
N
stage i
stage ii
stage iii
stage iv
tumor_stage
tumor_stage
N
stage i
stage ii
stage iii
stage iv
