# Supplementary material for: Repurposing Dihydroartemisinin to Combat Oral Squamous Cell Carcinoma, Associated with Mitochondrial Dysfunction and Oxidative Stress
Source: Oxid Med Cell Longev. 2023 Feb 16;2023:9595201. doi: 10.1155/2023/9595201 (PMC10239307; doi:10.1155/2023/9595201)
Supplement: Supplementary 9 — Supplementary Table 1: clinical OSCC sample_sheet from TCGA public database. [file 9595201.f9.pdf]

File.ID	File.Name	Data.Category	Data.Type	Project.ID	Case.ID	Sample.ID	Sample.Type	sample	type
55d710a9-a177-4f8b-89e4-68fe7faf0700	12a40f0f-e59b-4fd7-9e11-76cb27caa528.FPKM.txt.gz	Transcriptome Profiling	Gene Expression Quantification	TCGA-HNSC	TCGA-CN-6016	TCGA-CN-6016-01A	Primary Tumor	TCGA.CN.6016.01A	Tumor
392e66b7-26a2-49d6-9ecb-b46a49178128	ed0f20d3-f5e6-4b69-99a2-50d281e6e86a.FPKM.txt.gz	Transcriptome Profiling	Gene Expression Quantification	TCGA-HNSC	TCGA-CN-6024	TCGA-CN-6024-01A	Primary Tumor	TCGA.CN.6024.01A	Tumor
e14d7782-23dd-472f-91a7-80dacf1a1661	8502070e-8dde-4cd4-94e7-4eb06ec37d2f.FPKM.txt.gz	Transcriptome Profiling	Gene Expression Quantification	TCGA-HNSC	TCGA-BB-4224	TCGA-BB-4224-01A	Primary Tumor	TCGA.BB.4224.01A	Tumor
58a95752-31eb-4d57-80f6-3e115cacb3e9	b9da241a-3715-4c95-9d22-d43c7f4d7552.FPKM.txt.gz	Transcriptome Profiling	Gene Expression Quantification	TCGA-HNSC	TCGA-BA-4075	TCGA-BA-4075-01A	Primary Tumor	TCGA.BA.4075.01A	Tumor
960fa069-b423-4ddf-9c97-3d9cd61cf58d	37ba0f80-6041-4e6d-bfa5-c177ac6094b0.FPKM.txt.gz	Transcriptome Profiling	Gene Expression Quantification	TCGA-HNSC	TCGA-KU-A6H8	TCGA-KU-A6H8-01A	Primary Tumor	TCGA.KU.A6H8.01A	Tumor
9e285124-26ff-4b79-bd1c-87400fe51d92	5081bdea-794f-4547-8a5e-507496e2a052.FPKM.txt.gz	Transcriptome Profiling	Gene Expression Quantification	TCGA-HNSC	TCGA-CV-6939	TCGA-CV-6939-11A	Solid Tissue Normal	TCGA.CV.6939.11A	Normal
5cd64f4a-5816-4634-918b-20feb5e6dcaf	61199357-d9a5-4f09-976c-2ccb0fe241f0.FPKM.txt.gz	Transcriptome Profiling	Gene Expression Quantification	TCGA-HNSC	TCGA-P3-A5QA	TCGA-P3-A5QA-01A	Primary Tumor	TCGA.P3.A5QA.01A	Tumor
1bc91054-1654-45d6-aaf6-8f5520bdf0b3	d46bb04d-aca3-468a-ba80-3bc12e2b3919.FPKM.txt.gz	Transcriptome Profiling	Gene Expression Quantification	TCGA-HNSC	TCGA-CQ-6218	TCGA-CQ-6218-01A	Primary Tumor	TCGA.CQ.6218.01A	Tumor
15fd3816-bb93-4e96-a8d2-e71ab251c316	c67a9442-495e-4523-a9c7-b3cbbeecc1ce.FPKM.txt.gz	Transcriptome Profiling	Gene Expression Quantification	TCGA-HNSC	TCGA-CR-6488	TCGA-CR-6488-01A	Primary Tumor	TCGA.CR.6488.01A	Tumor
b0d13c07-97f1-4d80-b196-da33ccdf4eff	b7081519-274c-4610-bd53-c4beaa0a53e6.FPKM.txt.gz	Transcriptome Profiling	Gene Expression Quantification	TCGA-HNSC	TCGA-CN-5370	TCGA-CN-5370-01A	Primary Tumor	TCGA.CN.5370.01A	Tumor
ce8a68b9-e648-4482-8f34-e8caf85b66a3	06dc3b74-4514-4f97-b6a0-28b0ecca2262.FPKM.txt.gz	Transcriptome Profiling	Gene Expression Quantification	TCGA-HNSC	TCGA-IQ-A61J	TCGA-IQ-A61J-01A	Primary Tumor	TCGA.IQ.A61J.01A	Tumor
a5d64efd-3aed-40ab-b9d7-e28456ca5ec5	a56fd480-01e5-4d43-a38d-4bb89cc559b5.FPKM.txt.gz	Transcriptome Profiling	Gene Expression Quantification	TCGA-HNSC	TCGA-H7-A6C4	TCGA-H7-A6C4-11A	Solid Tissue Normal	TCGA.H7.A6C4.11A	Normal
5df9759b-47cc-44a2-bd25-0cd8ea29340c	4596bf99-aedb-40cf-8e98-ec3b953897f0.FPKM.txt.gz	Transcriptome Profiling	Gene Expression Quantification	TCGA-HNSC	TCGA-CQ-5325	TCGA-CQ-5325-01A	Primary Tumor	TCGA.CQ.5325.01A	Tumor
82918ef2-59ce-41ce-a3db-454ed4c792d3	74882059-8730-4b3f-8dd2-69704dda3aa4.FPKM.txt.gz	Transcriptome Profiling	Gene Expression Quantification	TCGA-HNSC	TCGA-CV-6959	TCGA-CV-6959-01A	Primary Tumor	TCGA.CV.6959.01A	Tumor
ef264309-227d-47a4-947f-8235b408cf4d	24535c31-1ee2-4af9-b908-b52bbfa3ee5f.FPKM.txt.gz	Transcriptome Profiling	Gene Expression Quantification	TCGA-HNSC	TCGA-CV-6939	TCGA-CV-6939-01A	Primary Tumor	TCGA.CV.6939.01A	Tumor
8aef7b5e-ea0c-420c-8e0f-c9dcdc98a868	ae2a857b-c219-4361-8d55-23047f8355fe.FPKM.txt.gz	Transcriptome Profiling	Gene Expression Quantification	TCGA-HNSC	TCGA-CV-6003	TCGA-CV-6003-01A	Primary Tumor	TCGA.CV.6003.01A	Tumor
cf411064-8466-49ef-946a-99ea49a7cd03	57fb015b-ca45-4615-87fc-66e4975efde9.FPKM.txt.gz	Transcriptome Profiling	Gene Expression Quantification	TCGA-HNSC	TCGA-CN-4736	TCGA-CN-4736-01A	Primary Tumor	TCGA.CN.4736.01A	Tumor
943889fb-4a00-4ba9-9cbb-74d69066f174	d19f374b-fde4-4b46-830f-56975e437fdc.FPKM.txt.gz	Transcriptome Profiling	Gene Expression Quantification	TCGA-HNSC	TCGA-CV-7104	TCGA-CV-7104-01A	Primary Tumor	TCGA.CV.7104.01A	Tumor
ecd1e738-b504-4f8d-9a88-af322c058d83	2e648bea-efbd-4700-86f8-300d26e153ee.FPKM.txt.gz	Transcriptome Profiling	Gene Expression Quantification	TCGA-HNSC	TCGA-CR-7394	TCGA-CR-7394-01A	Primary Tumor	TCGA.CR.7394.01A	Tumor
da56e17e-2519-4df9-9599-4c5a12e5c998	080d17ee-f898-4e67-bd6f-7019834171ac.FPKM.txt.gz	Transcriptome Profiling	Gene Expression Quantification	TCGA-HNSC	TCGA-QK-A652	TCGA-QK-A652-01A	Primary Tumor	TCGA.QK.A652.01A	Tumor
e8b716fd-5f15-446b-9ef2-52745ad6825e	07a6cb77-dbf5-40cc-8391-0d60f3483c81.FPKM.txt.gz	Transcriptome Profiling	Gene Expression Quantification	TCGA-HNSC	TCGA-CV-A6JO	TCGA-CV-A6JO-01B	Primary Tumor	TCGA.CV.A6JO.01B	Tumor
90d853fe-24e3-4851-b63c-61f2b5bfc6f5	301fe08e-bffe-4f01-9e80-12f61f830cac.FPKM.txt.gz	Transcriptome Profiling	Gene Expression Quantification	TCGA-HNSC	TCGA-CV-A45P	TCGA-CV-A45P-01A	Primary Tumor	TCGA.CV.A45P.01A	Tumor
81484ed6-9c06-4be0-8c84-63680b45c0f7	537d05c7-a7d0-48ab-aae0-90fde055a277.FPKM.txt.gz	Transcriptome Profiling	Gene Expression Quantification	TCGA-HNSC	TCGA-CV-5970	TCGA-CV-5970-01A	Primary Tumor	TCGA.CV.5970.01A	Tumor
2edf326d-bc68-46aa-b219-91072b49641e	5082fc15-781a-4d4c-b68a-c9de0edc77ba.FPKM.txt.gz	Transcriptome Profiling	Gene Expression Quantification	TCGA-HNSC	TCGA-CV-7103	TCGA-CV-7103-01A	Primary Tumor	TCGA.CV.7103.01A	Tumor
20011c2e-2a56-47aa-9ee0-2ec2a283901f	1e08c82a-70bc-4a09-bdb0-f9248b581dbc.FPKM.txt.gz	Transcriptome Profiling	Gene Expression Quantification	TCGA-HNSC	TCGA-D6-8569	TCGA-D6-8569-01A	Primary Tumor	TCGA.D6.8569.01A	Tumor
db8a3587-afef-46fb-afd5-cdb9f65b7786	e698cf41-8ad9-4765-a3a8-80b7f4f6b417.FPKM.txt.gz	Transcriptome Profiling	Gene Expression Quantification	TCGA-HNSC	TCGA-CR-7392	TCGA-CR-7392-01A	Primary Tumor	TCGA.CR.7392.01A	Tumor
8bcb074b-2e3c-4ea4-a5a7-d3037c3c7d92	e5d2d3c9-5d34-4c98-b810-97795cd1c9b1.FPKM.txt.gz	Transcriptome Profiling	Gene Expression Quantification	TCGA-HNSC	TCGA-CV-7438	TCGA-CV-7438-11A	Solid Tissue Normal	TCGA.CV.7438.11A	Normal
199fa374-acc9-441d-bee2-00014a45a088	0c8fee2b-71b6-4eb8-b983-8b75521c2a37.FPKM.txt.gz	Transcriptome Profiling	Gene Expression Quantification	TCGA-HNSC	TCGA-DQ-5625	TCGA-DQ-5625-01A	Primary Tumor	TCGA.DQ.5625.01A	Tumor
4d4f2889-3213-4c79-a06f-c9c0afb92752	86fee03b-f9f3-41cf-8969-6860e0b2a5e7.FPKM.txt.gz	Transcriptome Profiling	Gene Expression Quantification	TCGA-HNSC	TCGA-CV-5973	TCGA-CV-5973-01A	Primary Tumor	TCGA.CV.5973.01A	Tumor
0f09ec13-70ca-493c-a42c-7c75742c8d25	afb668fa-aa77-4b25-886a-6afc513d8376.FPKM.txt.gz	Transcriptome Profiling	Gene Expression Quantification	TCGA-HNSC	TCGA-CV-6933	TCGA-CV-6933-01A	Primary Tumor	TCGA.CV.6933.01A	Tumor
a5161c8f-3ffe-4a3b-b1a3-0116f938bcc3	d7e93acc-cec6-4a70-9b90-37bc080ee5c9.FPKM.txt.gz	Transcriptome Profiling	Gene Expression Quantification	TCGA-HNSC	TCGA-CV-7236	TCGA-CV-7236-01A	Primary Tumor	TCGA.CV.7236.01A	Tumor
19650e03-1102-46a4-b493-6e2e0fdd54f8	8f756223-6229-49bf-b96f-22d9a4b622bc.FPKM.txt.gz	Transcriptome Profiling	Gene Expression Quantification	TCGA-HNSC	TCGA-CV-6959	TCGA-CV-6959-11A	Solid Tissue Normal	TCGA.CV.6959.11A	Normal
e9c622cf-2748-4bfd-8cc3-b97dcf88201f	c1317d94-8e44-4426-9185-c0af48d12447.FPKM.txt.gz	Transcriptome Profiling	Gene Expression Quantification	TCGA-HNSC	TCGA-CV-7180	TCGA-CV-7180-01A	Primary Tumor	TCGA.CV.7180.01A	Tumor
88ab5d11-8ebf-4778-97ad-eca52a1b03f3	7d93c086-f38d-4a18-b906-c607647d8e54.FPKM.txt.gz	Transcriptome Profiling	Gene Expression Quantification	TCGA-HNSC	TCGA-CV-A465	TCGA-CV-A465-01A	Primary Tumor	TCGA.CV.A465.01A	Tumor
f31f0bf2-1381-4b7e-836f-3257a522b45b	54e100ab-a87e-49b4-a272-af7f546791ff.FPKM.txt.gz	Transcriptome Profiling	Gene Expression Quantification	TCGA-HNSC	TCGA-CV-7446	TCGA-CV-7446-01A	Primary Tumor	TCGA.CV.7446.01A	Tumor
996b9650-7f41-4aab-972a-d3d80f3e90a6	e623911e-3b9b-42df-9b1f-46b435081c47.FPKM.txt.gz	Transcriptome Profiling	Gene Expression Quantification	TCGA-HNSC	TCGA-HD-7831	TCGA-HD-7831-01A	Primary Tumor	TCGA.HD.7831.01A	Tumor
af158c7c-f391-40b5-a345-2aab5d5062a5	b6479904-c5ce-439c-9d56-990b921bac30.FPKM.txt.gz	Transcriptome Profiling	Gene Expression Quantification	TCGA-HNSC	TCGA-CV-5979	TCGA-CV-5979-01A	Primary Tumor	TCGA.CV.5979.01A	Tumor
a7d36c99-079e-412b-89d5-d4e41f16cba6	51988c9c-bf24-4706-ac30-c29986819d82.FPKM.txt.gz	Transcriptome Profiling	Gene Expression Quantification	TCGA-HNSC	TCGA-CQ-A4CA	TCGA-CQ-A4CA-01A	Primary Tumor	TCGA.CQ.A4CA.01A	Tumor
008cfb58-052a-4249-ac72-c3fb857de845	7444bf59-9dfd-4037-b405-c9e45d78b445.FPKM.txt.gz	Transcriptome Profiling	Gene Expression Quantification	TCGA-HNSC	TCGA-IQ-A6SH	TCGA-IQ-A6SH-01A	Primary Tumor	TCGA.IQ.A6SH.01A	Tumor
9421e5f6-bf8f-4a71-96c9-a583ae933b84	33a2e244-9f45-45e2-b2f3-ad4c52388490.FPKM.txt.gz	Transcriptome Profiling	Gene Expression Quantification	TCGA-HNSC	TCGA-BA-A6DE	TCGA-BA-A6DE-01A	Primary Tumor	TCGA.BA.A6DE.01A	Tumor
ff7c8af5-aa5a-44ed-aa89-ddbae8753f59	5a6262b0-7930-40c5-bfa6-40fcd4ba7b4c.FPKM.txt.gz	Transcriptome Profiling	Gene Expression Quantification	TCGA-HNSC	TCGA-CV-6433	TCGA-CV-6433-01A	Primary Tumor	TCGA.CV.6433.01A	Tumor
86e93274-4821-4568-9b4f-bb29d102dffe	cd486a20-1d8c-46d7-b49e-28f6e0e86ea6.FPKM.txt.gz	Transcriptome Profiling	Gene Expression Quantification	TCGA-HNSC	TCGA-CV-6933	TCGA-CV-6933-11A	Solid Tissue Normal	TCGA.CV.6933.11A	Normal
7eba114a-6cf8-4e3f-bc19-4c1605065a72	a4e355f9-b44d-43ab-ba1e-d76e0538f8fb.FPKM.txt.gz	Transcriptome Profiling	Gene Expression Quantification	TCGA-HNSC	TCGA-CV-6941	TCGA-CV-6941-01A	Primary Tumor	TCGA.CV.6941.01A	Tumor
2c2f9581-a890-4f01-90ab-5d71f3497913	1004e3e8-6bec-472b-bfce-5042d1f6912b.FPKM.txt.gz	Transcriptome Profiling	Gene Expression Quantification	TCGA-HNSC	TCGA-CQ-6222	TCGA-CQ-6222-01A	Primary Tumor	TCGA.CQ.6222.01A	Tumor
9f51055a-24cd-43bb-a606-6a0a9857e669	49fc6d64-3d80-4555-9645-a7d91fd59d33.FPKM.txt.gz	Transcriptome Profiling	Gene Expression Quantification	TCGA-HNSC	TCGA-CV-6441	TCGA-CV-6441-01A	Primary Tumor	TCGA.CV.6441.01A	Tumor
485c4a06-c1ec-4b34-9f87-c54b12e4a173	9616e47a-f554-445b-95af-aeb2a402d630.FPKM.txt.gz	Transcriptome Profiling	Gene Expression Quantification	TCGA-HNSC	TCGA-CN-4742	TCGA-CN-4742-01A	Primary Tumor	TCGA.CN.4742.01A	Tumor
c9551ef7-ef0d-46d7-9672-48e3803c292f	c0af0359-15f8-47cb-8071-b5701a35ecd2.FPKM.txt.gz	Transcriptome Profiling	Gene Expression Quantification	TCGA-HNSC	TCGA-CV-A6JT	TCGA-CV-A6JT-01A	Primary Tumor	TCGA.CV.A6JT.01A	Tumor
8e05498d-e45b-4f64-a37d-6e9d31b9fd67	7f0f64a8-9610-4663-acdd-a769573e9f4b.FPKM.txt.gz	Transcriptome Profiling	Gene Expression Quantification	TCGA-HNSC	TCGA-BA-4074	TCGA-BA-4074-01A	Primary Tumor	TCGA.BA.4074.01A	Tumor
fee35984-fcbc-4db2-ac98-48daf44d125c	f1aca685-306a-415d-b8b1-e3c5226e3b7d.FPKM.txt.gz	Transcriptome Profiling	Gene Expression Quantification	TCGA-HNSC	TCGA-DQ-5631	TCGA-DQ-5631-01A	Primary Tumor	TCGA.DQ.5631.01A	Tumor
472a988d-e3b5-486c-8148-e07a16018419	53b04dae-845b-4fc0-b98e-49ab5f81663f.FPKM.txt.gz	Transcriptome Profiling	Gene Expression Quantification	TCGA-HNSC	TCGA-CV-6952	TCGA-CV-6952-01A	Primary Tumor	TCGA.CV.6952.01A	Tumor
cd744fd5-3909-4d3d-bfbc-f6354ba3d48d	4ea57542-c760-4311-9f5e-dcd9d9dc33b5.FPKM.txt.gz	Transcriptome Profiling	Gene Expression Quantification	TCGA-HNSC	TCGA-BA-7269	TCGA-BA-7269-01A	Primary Tumor	TCGA.BA.7269.01A	Tumor
941e3b13-acbd-4f9e-a4e4-4f9ce6accee4	6150399d-74bb-4065-9724-7e90ade0a9ec.FPKM.txt.gz	Transcriptome Profiling	Gene Expression Quantification	TCGA-HNSC	TCGA-CQ-5327	TCGA-CQ-5327-01A	Primary Tumor	TCGA.CQ.5327.01A	Tumor
1a333bdb-9161-48bb-8075-9fe90a361865	2f069559-91be-47bf-bdfc-7257603f41c3.FPKM.txt.gz	Transcriptome Profiling	Gene Expression Quantification	TCGA-HNSC	TCGA-CR-7393	TCGA-CR-7393-01A	Primary Tumor	TCGA.CR.7393.01A	Tumor
dc5de3da-4e78-425b-a6c6-4a5ff74dbbd4	e263f4f6-f6ed-444e-8d91-4aed04ca2bf3.FPKM.txt.gz	Transcriptome Profiling	Gene Expression Quantification	TCGA-HNSC	TCGA-CN-4725	TCGA-CN-4725-01A	Primary Tumor	TCGA.CN.4725.01A	Tumor
28559236-580d-43c2-8d00-6bf07237392c	ea729fa0-1b54-485d-bc77-09660d3ad822.FPKM.txt.gz	Transcriptome Profiling	Gene Expression Quantification	TCGA-HNSC	TCGA-C9-A47Z	TCGA-C9-A47Z-01A	Primary Tumor	TCGA.C9.A47Z.01A	Tumor
53d2d7fb-3d4e-4cf3-a29d-a49c85f14c36	c59e3211-e578-48af-95a9-84e1a8f4d28b.FPKM.txt.gz	Transcriptome Profiling	Gene Expression Quantification	TCGA-HNSC	TCGA-IQ-A61E	TCGA-IQ-A61E-01A	Primary Tumor	TCGA.IQ.A61E.01A	Tumor
450bd706-6f0e-4195-b159-95501fadd871	db6cc551-0cd6-49b4-8167-fff8dd55d59e.FPKM.txt.gz	Transcriptome Profiling	Gene Expression Quantification	TCGA-HNSC	TCGA-HD-8635	TCGA-HD-8635-11A	Solid Tissue Normal	TCGA.HD.8635.11A	Normal
f3537151-1cb7-45e9-84c8-b8e7c537e865	da50f41d-e644-4481-94b8-62c31538e4b8.FPKM.txt.gz	Transcriptome Profiling	Gene Expression Quantification	TCGA-HNSC	TCGA-MT-A51X	TCGA-MT-A51X-01A	Primary Tumor	TCGA.MT.A51X.01A	Tumor
3d2ff227-81e2-4308-a6f2-365720002afd	113a6464-b2b9-4566-b501-9060291b54b3.FPKM.txt.gz	Transcriptome Profiling	Gene Expression Quantification	TCGA-HNSC	TCGA-CV-6954	TCGA-CV-6954-01A	Primary Tumor	TCGA.CV.6954.01A	Tumor
51040db6-b4f1-4e1a-8667-f9669504b5ff	5a16cb58-82c0-49ee-8f71-5cf738e0beb2.FPKM.txt.gz	Transcriptome Profiling	Gene Expression Quantification	TCGA-HNSC	TCGA-CQ-A4CH	TCGA-CQ-A4CH-01A	Primary Tumor	TCGA.CQ.A4CH.01A	Tumor
b765e887-dddd-4a66-80b1-a58ba74b3bf4	e3997ec9-6b95-4faf-a7a6-f041c9fe094f.FPKM.txt.gz	Transcriptome Profiling	Gene Expression Quantification	TCGA-HNSC	TCGA-CV-6956	TCGA-CV-6956-01A	Primary Tumor	TCGA.CV.6956.01A	Tumor
668ebd50-efe3-43b7-8372-1a9223596183	59c9c406-1687-42ed-8be8-4590c9577094.FPKM.txt.gz	Transcriptome Profiling	Gene Expression Quantification	TCGA-HNSC	TCGA-CQ-5333	TCGA-CQ-5333-01A	Primary Tumor	TCGA.CQ.5333.01A	Tumor

cac2e8aa-0afa-459c-a3b6-15424f00ddc8	59d1b758-2bea-40ff-a147-6c116a1bae9c.FPKM.txt.gz	Transcriptome Profiling	Gene Expression Quantification	TCGA-HNSC	TCGA-CQ-7072	TCGA-CQ-7072-01A	Primary Tumor	TCGA.CQ.7072.01A	Tumor
2edd6c30-2d5d-42c9-b6ee-1b69b8f2117e	e055bccc-cdd1-4cf4-8fb8-fbdbd0d3a773.FPKM.txt.gz	Transcriptome Profiling	Gene Expression Quantification	TCGA-HNSC	TCGA-F7-A50J	TCGA-F7-A50J-01A	Primary Tumor	TCGA.F7.A50J.01A	Tumor
b50ea6a1-2298-4f61-94ef-91c5a6e3e75c	92329d1b-1249-45e2-8adc-d94cfcfacb2e.FPKM.txt.gz	Transcriptome Profiling	Gene Expression Quantification	TCGA-HNSC	TCGA-CV-A45T	TCGA-CV-A45T-01A	Primary Tumor	TCGA.CV.A45T.01A	Tumor
5471499e-ec94-4f55-bad5-fe76a7b2cab4	2be3f783-b3cb-4074-b565-d941158f0ff6.FPKM.txt.gz	Transcriptome Profiling	Gene Expression Quantification	TCGA-HNSC	TCGA-D6-6823	TCGA-D6-6823-01A	Primary Tumor	TCGA.D6.6823.01A	Tumor
cad320e6-80cf-4dbd-8546-7822972fb235	7c634205-6de0-48fe-800e-88a4ff43e498.FPKM.txt.gz	Transcriptome Profiling	Gene Expression Quantification	TCGA-HNSC	TCGA-CV-A6JU	TCGA-CV-A6JU-01A	Primary Tumor	TCGA.CV.A6JU.01A	Tumor
83401bf0-0c18-43ab-a0e6-bb57311499c4	34c3a27a-a4bf-4c6c-b34d-67154354933b.FPKM.txt.gz	Transcriptome Profiling	Gene Expression Quantification	TCGA-HNSC	TCGA-CN-6019	TCGA-CN-6019-01A	Primary Tumor	TCGA.CN.6019.01A	Tumor
b12a843b-f0a5-45a1-b2e6-10bb483520fa	31ed3ca5-bfa9-41ce-819f-18ace1a62099.FPKM.txt.gz	Transcriptome Profiling	Gene Expression Quantification	TCGA-HNSC	TCGA-CV-7238	TCGA-CV-7238-01A	Primary Tumor	TCGA.CV.7238.01A	Tumor
db1e047f-8a18-4a9b-bac4-bb4d099246eb	ef4c294b-b29c-4c00-996c-26195febad00.FPKM.txt.gz	Transcriptome Profiling	Gene Expression Quantification	TCGA-HNSC	TCGA-F7-A50G	TCGA-F7-A50G-01A	Primary Tumor	TCGA.F7.A50G.01A	Tumor
2ec4fce5-b1ad-4d1d-ba2d-6c0a4098e8a7	a32b6a6b-c776-473d-925a-c82421b1c5f1.FPKM.txt.gz	Transcriptome Profiling	Gene Expression Quantification	TCGA-HNSC	TCGA-CN-6017	TCGA-CN-6017-01A	Primary Tumor	TCGA.CN.6017.01A	Tumor
0077f87b-f1b3-40cd-81a3-97c254108a11	09001b78-7752-4939-847f-f1674503e4a4.FPKM.txt.gz	Transcriptome Profiling	Gene Expression Quantification	TCGA-HNSC	TCGA-CV-6945	TCGA-CV-6945-01A	Primary Tumor	TCGA.CV.6945.01A	Tumor
2b344e0f-36d8-42d5-b0c9-ad66f8a9de6e	a4aaba48-26ef-4400-b3a0-9d6922ef76e7.FPKM.txt.gz	Transcriptome Profiling	Gene Expression Quantification	TCGA-HNSC	TCGA-UF-A7JS	TCGA-UF-A7JS-01A	Primary Tumor	TCGA.UF.A7JS.01A	Tumor
e93bcd62-5dc0-4792-8415-7399ba97f4db	ee00edb2-f47a-4d1b-a2e7-3441cfdb29f6.FPKM.txt.gz	Transcriptome Profiling	Gene Expression Quantification	TCGA-HNSC	TCGA-CQ-6219	TCGA-CQ-6219-01A	Primary Tumor	TCGA.CQ.6219.01A	Tumor
1cd466e0-ecd2-4adf-8377-57aa0d28b27c	39b59f6d-b1ff-4c27-989f-c30e539b8a40.FPKM.txt.gz	Transcriptome Profiling	Gene Expression Quantification	TCGA-HNSC	TCGA-BA-A6DG	TCGA-BA-A6DG-01A	Primary Tumor	TCGA.BA.A6DG.01A	Tumor
52719581-7d1f-4fc3-8792-e3e1ce9fb16a	35d3f8a8-6364-43b2-bbd3-c237986f3ee9.FPKM.txt.gz	Transcriptome Profiling	Gene Expression Quantification	TCGA-HNSC	TCGA-BA-6873	TCGA-BA-6873-01A	Primary Tumor	TCGA.BA.6873.01A	Tumor
8bf82d0b-82ff-4740-aae0-4364616e6a7d	d939e0e3-4b94-48d3-a558-fa212f84fc8c.FPKM.txt.gz	Transcriptome Profiling	Gene Expression Quantification	TCGA-HNSC	TCGA-IQ-A61H	TCGA-IQ-A61H-01A	Primary Tumor	TCGA.IQ.A61H.01A	Tumor
1295381d-92d1-477f-adb0-a300acfa4daf	26b7907d-dec3-4517-b98f-27189c54e3c3.FPKM.txt.gz	Transcriptome Profiling	Gene Expression Quantification	TCGA-HNSC	TCGA-CV-6951	TCGA-CV-6951-01A	Primary Tumor	TCGA.CV.6951.01A	Tumor
93e19a20-1f61-4b04-9607-48ab20f50594	f5cdc287-7046-4dfe-af4c-ae7363aa84c6.FPKM.txt.gz	Transcriptome Profiling	Gene Expression Quantification	TCGA-HNSC	TCGA-CN-4733	TCGA-CN-4733-01A	Primary Tumor	TCGA.CN.4733.01A	Tumor
64461e60-229e-46a2-ac6e-ff1429c6e380	0d5105ae-0b7e-4477-a822-cc20ba9423fa.FPKM.txt.gz	Transcriptome Profiling	Gene Expression Quantification	TCGA-HNSC	TCGA-F7-A61W	TCGA-F7-A61W-01A	Primary Tumor	TCGA.F7.A61W.01A	Tumor
dcfad7ea-0547-442b-a980-d6efd3f663fd	8483eeab-fb92-4597-992a-20a4dabc3712.FPKM.txt.gz	Transcriptome Profiling	Gene Expression Quantification	TCGA-HNSC	TCGA-HD-A6HZ	TCGA-HD-A6HZ-11A	Solid Tissue Normal	TCGA.HD.A6HZ.11A	Normal
29a5da8f-9414-4b35-9308-15580ba5a6f0	ae1545f7-1b6f-4a7f-897b-ae6882454afe.FPKM.txt.gz	Transcriptome Profiling	Gene Expression Quantification	TCGA-HNSC	TCGA-BB-A6UO	TCGA-BB-A6UO-01A	Primary Tumor	TCGA.BB.A6UO.01A	Tumor
7f892bee-649c-4147-8563-e82981638062	4919d5f0-8f43-4875-a2b1-d145fce8e362.FPKM.txt.gz	Transcriptome Profiling	Gene Expression Quantification	TCGA-HNSC	TCGA-CR-7390	TCGA-CR-7390-01A	Primary Tumor	TCGA.CR.7390.01A	Tumor
f1fe468e-074d-4169-adad-3d93e3f66c41	6be2aa36-5cc9-4832-af98-47e04d8424e0.FPKM.txt.gz	Transcriptome Profiling	Gene Expression Quantification	TCGA-HNSC	TCGA-CQ-6229	TCGA-CQ-6229-01A	Primary Tumor	TCGA.CQ.6229.01A	Tumor
063d611d-1510-4953-b655-14e44f6e9740	3684c680-68bc-4a84-b8f9-13142c6276e6.FPKM.txt.gz	Transcriptome Profiling	Gene Expression Quantification	TCGA-HNSC	TCGA-CV-7103	TCGA-CV-7103-11A	Solid Tissue Normal	TCGA.CV.7103.11A	Normal
9fc3977e-e8a0-4f2e-9f43-97b844332102	4b705795-afeb-4cab-be3c-2206af44fa84.FPKM.txt.gz	Transcriptome Profiling	Gene Expression Quantification	TCGA-HNSC	TCGA-BA-4077	TCGA-BA-4077-01B	Primary Tumor	TCGA.BA.4077.01B	Tumor
5484cdfd-9179-40dd-99a8-2f329de33d54	211f4551-8720-4f0d-9c59-88108fcc037f.FPKM.txt.gz	Transcriptome Profiling	Gene Expression Quantification	TCGA-HNSC	TCGA-HD-8635	TCGA-HD-8635-01A	Primary Tumor	TCGA.HD.8635.01A	Tumor
3ebedf46-9787-4625-bf89-857d8b07d4b8	5f2b6920-36df-43f2-b245-57d3352bf3a6.FPKM.txt.gz	Transcriptome Profiling	Gene Expression Quantification	TCGA-HNSC	TCGA-UP-A6WW	TCGA-UP-A6WW-01A	Primary Tumor	TCGA.UP.A6WW.01A	Tumor
c469cdf1-4ff3-493a-bf45-1a0075b928f5	23e18feb-ec4e-40f4-97bb-7567af02c1e4.FPKM.txt.gz	Transcriptome Profiling	Gene Expression Quantification	TCGA-HNSC	TCGA-CR-7391	TCGA-CR-7391-01A	Primary Tumor	TCGA.CR.7391.01A	Tumor
8e6411b1-8f9b-4ee5-a320-922be52a2748	4dab062b-5119-4e8a-a9bf-f2cfa2e6a6d6.FPKM.txt.gz	Transcriptome Profiling	Gene Expression Quantification	TCGA-HNSC	TCGA-CQ-5329	TCGA-CQ-5329-01A	Primary Tumor	TCGA.CQ.5329.01A	Tumor
581c46bb-2b8b-4b0f-aaf9-792e696757cf	c246ad49-e7fe-4e6c-8506-899bd081ea66.FPKM.txt.gz	Transcriptome Profiling	Gene Expression Quantification	TCGA-HNSC	TCGA-CQ-6221	TCGA-CQ-6221-01A	Primary Tumor	TCGA.CQ.6221.01A	Tumor
991c996a-7de1-4971-99dd-476e62a3b2f5	9767968a-11d5-474a-900f-5a91367e7418.FPKM.txt.gz	Transcriptome Profiling	Gene Expression Quantification	TCGA-HNSC	TCGA-DQ-5630	TCGA-DQ-5630-01A	Primary Tumor	TCGA.DQ.5630.01A	Tumor
59a53532-7ddb-4f17-a295-64b31cd81c94	47b9e3ef-3cdc-4ad4-8d6f-c658bc862f7e.FPKM.txt.gz	Transcriptome Profiling	Gene Expression Quantification	TCGA-HNSC	TCGA-CV-6934	TCGA-CV-6934-01A	Primary Tumor	TCGA.CV.6934.01A	Tumor
86fa23c9-e600-46ab-8208-8a0206934fbe	c5cd7905-ee91-4d7c-89b9-cf012338f402.FPKM.txt.gz	Transcriptome Profiling	Gene Expression Quantification	TCGA-HNSC	TCGA-CV-7255	TCGA-CV-7255-11A	Solid Tissue Normal	TCGA.CV.7255.11A	Normal
56b503d2-9092-47de-ac52-022c4621cb8c	bfc13d26-a053-4e72-bce9-29655bb7788f.FPKM.txt.gz	Transcriptome Profiling	Gene Expression Quantification	TCGA-HNSC	TCGA-CR-7401	TCGA-CR-7401-01A	Primary Tumor	TCGA.CR.7401.01A	Tumor
440f25dd-290e-4250-b711-7d963f1d39a4	04900b0d-c100-41f7-b58a-ca1529d472b6.FPKM.txt.gz	Transcriptome Profiling	Gene Expression Quantification	TCGA-HNSC	TCGA-DQ-7592	TCGA-DQ-7592-01A	Primary Tumor	TCGA.DQ.7592.01A	Tumor
e4e8535c-8857-4c77-8b2f-bcfe6f0dbdd0	0ea4cfc7-e096-4c03-96a0-792eedf79f56.FPKM.txt.gz	Transcriptome Profiling	Gene Expression Quantification	TCGA-HNSC	TCGA-4P-AA8J	TCGA-4P-AA8J-01A	Primary Tumor	TCGA.4P.AA8J.01A	Tumor
86702dd2-1d1f-4a7e-848d-cfb6964a2955	032c007c-a7c8-42bd-a7af-3134bcc8c4a1.FPKM.txt.gz	Transcriptome Profiling	Gene Expression Quantification	TCGA-HNSC	TCGA-IQ-A6SG	TCGA-IQ-A6SG-01A	Primary Tumor	TCGA.IQ.A6SG.01A	Tumor
45894483-0c10-4949-abc8-0c3a1729e9b1	26019321-345c-44e3-87be-7a49f2ec04d1.FPKM.txt.gz	Transcriptome Profiling	Gene Expression Quantification	TCGA-HNSC	TCGA-CN-4737	TCGA-CN-4737-01A	Primary Tumor	TCGA.CN.4737.01A	Tumor
dd52b0ea-7215-4c1e-9c11-7c9aad49232c	50a77663-3a8d-46b7-b3c6-89926478eb6d.FPKM.txt.gz	Transcriptome Profiling	Gene Expression Quantification	TCGA-HNSC	TCGA-CV-7238	TCGA-CV-7238-11A	Solid Tissue Normal	TCGA.CV.7238.11A	Normal
04b92be0-6a20-4d04-99c8-f3eecd3986e5	cea213f2-1dca-482a-bc29-d346a6682085.FPKM.txt.gz	Transcriptome Profiling	Gene Expression Quantification	TCGA-HNSC	TCGA-CN-A498	TCGA-CN-A498-01A	Primary Tumor	TCGA.CN.A498.01A	Tumor
7c9ba317-c40d-47e1-a6ac-bdc20290b3c0	955f400d-8b2f-49a3-9e07-d3a2b286d093.FPKM.txt.gz	Transcriptome Profiling	Gene Expression Quantification	TCGA-HNSC	TCGA-HD-8634	TCGA-HD-8634-01A	Primary Tumor	TCGA.HD.8634.01A	Tumor
0a7ebd2d-e990-40da-b43a-0f516cc39cf6	4eabde76-a065-4dff-8480-722a9a5f0c5c.FPKM.txt.gz	Transcriptome Profiling	Gene Expression Quantification	TCGA-HNSC	TCGA-CV-6934	TCGA-CV-6934-11A	Solid Tissue Normal	TCGA.CV.6934.11A	Normal
6aeb1a9a-13aa-467e-af40-51b8bd759e28	da68f717-ab42-4534-a518-16d40c844ad1.FPKM.txt.gz	Transcriptome Profiling	Gene Expression Quantification	TCGA-HNSC	TCGA-HD-A6HZ	TCGA-HD-A6HZ-01A	Primary Tumor	TCGA.HD.A6HZ.01A	Tumor
1d586525-c3f5-4a5f-b1bd-4b82430d7a4a	55533823-eb81-4c6b-b6ab-163dccf7e39b.FPKM.txt.gz	Transcriptome Profiling	Gene Expression Quantification	TCGA-HNSC	TCGA-CR-7382	TCGA-CR-7382-01A	Primary Tumor	TCGA.CR.7382.01A	Tumor
6b13b8c7-087d-432d-a6d6-1ac7b17e3d3b	5959921c-0711-473c-85ea-6cf4ed0fb77f.FPKM.txt.gz	Transcriptome Profiling	Gene Expression Quantification	TCGA-HNSC	TCGA-F7-A61S	TCGA-F7-A61S-01A	Primary Tumor	TCGA.F7.A61S.01A	Tumor
3f110886-b222-4cd8-8686-6faddfe56269	e6321970-44a6-46b9-83b5-0928ae3b7786.FPKM.txt.gz	Transcriptome Profiling	Gene Expression Quantification	TCGA-HNSC	TCGA-CN-A642	TCGA-CN-A642-01A	Primary Tumor	TCGA.CN.A642.01A	Tumor
a906b2b2-4c3b-4857-9977-99ccc873050d	0b6141e2-00ef-4a7d-8859-f986aad44d06.FPKM.txt.gz	Transcriptome Profiling	Gene Expression Quantification	TCGA-HNSC	TCGA-CN-6996	TCGA-CN-6996-01A	Primary Tumor	TCGA.CN.6996.01A	Tumor
81a00301-49a3-4784-a951-85bcfc09e527	0968dd71-882c-4727-9b75-6e572d3b161e.FPKM.txt.gz	Transcriptome Profiling	Gene Expression Quantification	TCGA-HNSC	TCGA-CV-A45R	TCGA-CV-A45R-01A	Primary Tumor	TCGA.CV.A45R.01A	Tumor
dacb52c7-ef36-42ad-9d7f-b94931c8e5b4	ec13a6c1-ecca-4f5c-bbd2-f0310df9db46.FPKM.txt.gz	Transcriptome Profiling	Gene Expression Quantification	TCGA-HNSC	TCGA-H7-A6C4	TCGA-H7-A6C4-01A	Primary Tumor	TCGA.H7.A6C4.01A	Tumor
9333ebe0-936a-45b3-b5f2-03e5441f8183	418dd9f0-a87c-402e-8101-2c769e383cc5.FPKM.txt.gz	Transcriptome Profiling	Gene Expression Quantification	TCGA-HNSC	TCGA-D6-6515	TCGA-D6-6515-01A	Primary Tumor	TCGA.D6.6515.01A	Tumor
235ab83b-7434-4d2a-96a8-dc954a8ea768	571fc52e-e962-4614-b213-8529ae7bfa99.FPKM.txt.gz	Transcriptome Profiling	Gene Expression Quantification	TCGA-HNSC	TCGA-CV-5976	TCGA-CV-5976-01A	Primary Tumor	TCGA.CV.5976.01A	Tumor
5002201c-9a9f-49bb-a22a-8404d64d40df	1bc56e7f-f29d-4776-984a-2cf3dbf7efc4.FPKM.txt.gz	Transcriptome Profiling	Gene Expression Quantification	TCGA-HNSC	TCGA-CV-6956	TCGA-CV-6956-11A	Solid Tissue Normal	TCGA.CV.6956.11A	Normal
a2c41bc4-d1b1-4547-8370-d82eea6d758d	09a0944b-285f-4115-9715-3b1b4fd66f89.FPKM.txt.gz	Transcriptome Profiling	Gene Expression Quantification	TCGA-HNSC	TCGA-CV-7255	TCGA-CV-7255-01A	Primary Tumor	TCGA.CV.7255.01A	Tumor
b95c2f7e-c8c7-4827-a47c-8188e9362b9b	43f16f16-ef71-43ea-bf3f-5f594bdb293d.FPKM.txt.gz	Transcriptome Profiling	Gene Expression Quantification	TCGA-HNSC	TCGA-CX-7085	TCGA-CX-7085-01A	Primary Tumor	TCGA.CX.7085.01A	Tumor
caf1cae1-998e-4e5f-b9c1-e6f354f55343	d72c9ded-5e78-4d07-ba07-a52ea1032816.FPKM.txt.gz	Transcriptome Profiling	Gene Expression Quantification	TCGA-HNSC	TCGA-CN-5367	TCGA-CN-5367-01A	Primary Tumor	TCGA.CN.5367.01A	Tumor
e0d18e30-d67a-4423-b6a2-80977e5e0b58	3811c6a6-dd9f-4f76-92cc-d487bd443ed5.FPKM.txt.gz	Transcriptome Profiling	Gene Expression Quantification	TCGA-HNSC	TCGA-CV-5971	TCGA-CV-5971-01A	Primary Tumor	TCGA.CV.5971.01A	Tumor
9d64245e-eeeb-45bf-855b-1a7d3e885324	9b874579-0d12-425f-ba47-a0b52f752cb0.FPKM.txt.gz	Transcriptome Profiling	Gene Expression Quantification	TCGA-HNSC	TCGA-CV-5977	TCGA-CV-5977-01A	Primary Tumor	TCGA.CV.5977.01A	Tumor
dd892b18-19a4-4fd9-a82e-fa798ae8a578	84fbe27a-a8b8-4610-afdd-7ebf05d2ef76.FPKM.txt.gz	Transcriptome Profiling	Gene Expression Quantification	TCGA-HNSC	TCGA-MT-A67A	TCGA-MT-A67A-01A	Primary Tumor	TCGA.MT.A67A.01A	Tumor
5dfd7026-459b-40d2-a4b3-087922925137	b8f951b7-1e0c-4333-a763-1c81bdc938b8.FPKM.txt.gz	Transcriptome Profiling	Gene Expression Quantification	TCGA-HNSC	TCGA-C9-A480	TCGA-C9-A480-01A	Primary Tumor	TCGA.C9.A480.01A	Tumor
4bf25153-802f-49b1-8cab-801cecadc7ce	9dc3417f-c1d9-4935-ae0a-0686cf934615.FPKM.txt.gz	Transcriptome Profiling	Gene Expression Quantification	TCGA-HNSC	TCGA-CQ-A4CE	TCGA-CQ-A4CE-01A	Primary Tumor	TCGA.CQ.A4CE.01A	Tumor
9dc17731-0c4a-4997-afa9-b88d2c70d554	163d5aad-7488-4da9-9a09-e46fcb161d3e.FPKM.txt.gz	Transcriptome Profiling	Gene Expression Quantification	TCGA-HNSC	TCGA-CV-6961	TCGA-CV-6961-01A	Primary Tumor	TCGA.CV.6961.01A	Tumor
d34860b8-e351-4d9d-a407-6d685398f285	2a328b48-a908-4bcf-8add-955f317d6fac.FPKM.txt.gz	Transcriptome Profiling	Gene Expression Quantification	TCGA-HNSC	TCGA-CV-7438	TCGA-CV-7438-01A	Primary Tumor	TCGA.CV.7438.01A	Tumor
7bbb3e88-859e-4d62-bf1d-17fed7a6c006	200c86df-4c7d-4f53-aad4-8745dd2537a7.FPKM.txt.gz	Transcriptome Profiling	Gene Expression Quantification	TCGA-HNSC	TCGA-CN-6998	TCGA-CN-6998-01A	Primary Tumor	TCGA.CN.6998.01A	Tumor
955abd1e-c7ce-4a24-94cd-73c2cef97ef8	817d8462-672b-4373-a453-79cdd7498725.FPKM.txt.gz	Transcriptome Profiling	Gene Expression Quantification	TCGA-HNSC	TCGA-CR-6493	TCGA-CR-6493-01A	Primary Tumor	TCGA.CR.6493.01A	Tumor

63f2b2ee-3cf8-4d68-9dca-4a737b18880d	bae4bda2-14b0-4450-b96d-1b7496bab0dd.FPKM.txt.gz	Transcriptome Profiling	Gene Expression Quantification	TCGA-HNSC	TCGA-WA-A7H4	TCGA-WA-A7H4-01A	Primary Tumor	TCGA.WA.A7H4.01A	Tumor
0ccb1240-7a13-4ad6-9791-2c6db4373e44	b1bac168-47cc-4117-b43c-9fc886560b0b.FPKM.txt.gz	Transcriptome Profiling	Gene Expression Quantification	TCGA-HNSC	TCGA-D6-A4Z9	TCGA-D6-A4Z9-01A	Primary Tumor	TCGA.D6.A4Z9.01A	Tumor
2c3f62c5-5e5b-4f4c-9de8-6ef3646922e3	9be23df6-f5e3-4309-824a-52c7c4750380.FPKM.txt.gz	Transcriptome Profiling	Gene Expression Quantification	TCGA-HNSC	TCGA-D6-6825	TCGA-D6-6825-01A	Primary Tumor	TCGA.D6.6825.01A	Tumor
5bce7ca7-c7ad-433b-b329-1e1c23727436	e2c687dd-e061-4896-a362-7832f6ab30b2.FPKM.txt.gz	Transcriptome Profiling	Gene Expression Quantification	TCGA-HNSC	TCGA-DQ-5624	TCGA-DQ-5624-01A	Primary Tumor	TCGA.DQ.5624.01A	Tumor
11ba8ef6-f9e9-4ed3-8bef-8963bac90e65	abb7069d-2ead-4970-80e7-178fa8b4bb88.FPKM.txt.gz	Transcriptome Profiling	Gene Expression Quantification	TCGA-HNSC	TCGA-CQ-6224	TCGA-CQ-6224-01A	Primary Tumor	TCGA.CQ.6224.01A	Tumor
2bb39384-1cac-443f-8a78-1fd197798868	a7208747-f725-4d83-9fb0-e8e53b497938.FPKM.txt.gz	Transcriptome Profiling	Gene Expression Quantification	TCGA-HNSC	TCGA-CV-A6K0	TCGA-CV-A6K0-01B	Primary Tumor	TCGA.CV.A6K0.01B	Tumor
43921385-00de-4de2-95ca-3738d99cb889	0aa2ad55-702a-4241-9f36-58f1fe7d1302.FPKM.txt.gz	Transcriptome Profiling	Gene Expression Quantification	TCGA-HNSC	TCGA-CQ-7065	TCGA-CQ-7065-01A	Primary Tumor	TCGA.CQ.7065.01A	Tumor
60116c36-d1e9-4f98-b80a-5d1305592fef	5c6604b8-7256-4957-9193-5b42a162226e.FPKM.txt.gz	Transcriptome Profiling	Gene Expression Quantification	TCGA-HNSC	TCGA-BA-A6DB	TCGA-BA-A6DB-01A	Primary Tumor	TCGA.BA.A6DB.01A	Tumor
01402385-9e5d-48f6-b16d-f29651c7f479	21bf82e4-3223-481c-a89b-f41e42628764.FPKM.txt.gz	Transcriptome Profiling	Gene Expression Quantification	TCGA-HNSC	TCGA-CR-7397	TCGA-CR-7397-01A	Primary Tumor	TCGA.CR.7397.01A	Tumor
4cf93b7e-99a2-43ea-8cc2-016b2ea3f8a4	9dcc4f82-49ff-43d5-af4b-e1fd6480b863.FPKM.txt.gz	Transcriptome Profiling	Gene Expression Quantification	TCGA-HNSC	TCGA-CQ-5330	TCGA-CQ-5330-01A	Primary Tumor	TCGA.CQ.5330.01A	Tumor
c3ae2486-71c6-4304-9a63-84e8a0f86ed2	bef39330-3d03-4355-85f2-74c454d32850.FPKM.txt.gz	Transcriptome Profiling	Gene Expression Quantification	TCGA-HNSC	TCGA-QK-AA3K	TCGA-QK-AA3K-01A	Primary Tumor	TCGA.QK.AA3K.01A	Tumor
ddf6041c-68ae-4350-977d-01a5e1ddddcb	60eeee3f-55f1-468c-b6eb-bd4d8cddc6d1.FPKM.txt.gz	Transcriptome Profiling	Gene Expression Quantification	TCGA-HNSC	TCGA-CV-6436	TCGA-CV-6436-01A	Primary Tumor	TCGA.CV.6436.01A	Tumor
973909d5-d936-4272-86be-a862c6a7dae0	3241cae3-5e31-4126-b0f3-553f748b2be1.FPKM.txt.gz	Transcriptome Profiling	Gene Expression Quantification	TCGA-HNSC	TCGA-CR-7372	TCGA-CR-7372-01A	Primary Tumor	TCGA.CR.7372.01A	Tumor
dc356273-8361-4ca6-8966-0589b49703a5	b4a4cc9a-5053-4968-a54e-be08e53ce2e1.FPKM.txt.gz	Transcriptome Profiling	Gene Expression Quantification	TCGA-HNSC	TCGA-D6-A4ZB	TCGA-D6-A4ZB-01A	Primary Tumor	TCGA.D6.A4ZB.01A	Tumor
36d8eca8-a59f-41bb-be44-f1720949bf45	1f781542-09e0-48ff-8363-3a6cef4ebc83.FPKM.txt.gz	Transcriptome Profiling	Gene Expression Quantification	TCGA-HNSC	TCGA-CV-6961	TCGA-CV-6961-11A	Solid Tissue Normal	TCGA.CV.6961.11A	Normal
