# Supplementary material for: Repurposing Dihydroartemisinin to Combat Oral Squamous Cell Carcinoma, Associated with Mitochondrial Dysfunction and Oxidative Stress
Source: Oxid Med Cell Longev. 2023 Feb 16;2023:9595201. doi: 10.1155/2023/9595201 (PMC10239307; doi:10.1155/2023/9595201)
Supplement: Supplementary 10 — Supplementary Table 2: clinical information of the OSCC patient from TCGA public database. [file 9595201.f10.pdf]

case_id	case_subm	project_i	age_at_ir	age_is_ob	cause_of_	cause_of_	country_c	days_to_b	days_to_c	ethnicity	gender	occupatic	premature	race	vital_sta
ae90972d-	TCGA-CQ-A	TCGA-HNSC	58	'--	'--	'--	'--	-21455	379	not hispa	male	'--	'--	asian	Dead
ae90972d-	TCGA-CQ-A	TCGA-HNSC	58	'--	'--	'--	'--	-21455	379	not hispa	male	'--	'--	asian	Dead
03aca47b-	TCGA-CR-7	TCGA-HNSC	67	'--	'--	'--	'--	-24650	'--	not hispa	female	'--	'--	white	Alive
03aca47b-	TCGA-CR-7	TCGA-HNSC	67	'--	'--	'--	'--	-24650	'--	not hispa	female	'--	'--	white	Alive
42bc2336-	TCGA-D6-6	TCGA-HNSC	73	'--	'--	'--	'--	-26976	'--	not hispa	male	'--	'--	white	Alive
42bc2336-	TCGA-D6-6	TCGA-HNSC	73	'--	'--	'--	'--	-26976	'--	not hispa	male	'--	'--	white	Alive
f030e410-	TCGA-CR-7	TCGA-HNSC	49	'--	'--	'--	'--	-18078	'--	not hispa	male	'--	'--	white	Alive
f030e410-	TCGA-CR-7	TCGA-HNSC	49	'--	'--	'--	'--	-18078	'--	not hispa	male	'--	'--	white	Alive
90d21b9b-	TCGA-IQ-A	TCGA-HNSC	76	'--	'--	'--	'--	-27990	'--	hispanic	male	'--	'--	white	Alive
90d21b9b-	TCGA-IQ-A	TCGA-HNSC	76	'--	'--	'--	'--	-27990	'--	hispanic	male	'--	'--	white	Alive
b4a41682-	TCGA-DQ-5	TCGA-HNSC	43	'--	'--	'--	'--	-15867	'--	not hispa	female	'--	'--	white	Alive
b4a41682-	TCGA-DQ-5	TCGA-HNSC	43	'--	'--	'--	'--	-15867	'--	not hispa	female	'--	'--	white	Alive
64a195f6-	TCGA-C9-A	TCGA-HNSC	72	'--	'--	'--	'--	-26592	191	not hispa	female	'--	'--	asian	Dead
64a195f6-	TCGA-C9-A	TCGA-HNSC	72	'--	'--	'--	'--	-26592	191	not hispa	female	'--	'--	asian	Dead
ef3ce0e5-	TCGA-CV-6	TCGA-HNSC	48	'--	'--	'--	'--	-17679	256	not hispa	male	'--	'--	white	Dead
ef3ce0e5-	TCGA-CV-6	TCGA-HNSC	48	'--	'--	'--	'--	-17679	256	not hispa	male	'--	'--	white	Dead
4075717b-	TCGA-CV-6	TCGA-HNSC	61	'--	'--	'--	'--	-22532	76	not hispa	male	'--	'--	white	Dead
4075717b-	TCGA-CV-6	TCGA-HNSC	61	'--	'--	'--	'--	-22532	76	not hispa	male	'--	'--	white	Dead
839b660c-	TCGA-CQ-6	TCGA-HNSC	79	'--	'--	'--	'--	-29026	'--	not hispa	male	'--	'--	white	Alive
839b660c-	TCGA-CQ-6	TCGA-HNSC	79	'--	'--	'--	'--	-29026	'--	not hispa	male	'--	'--	white	Alive
b7ac2f06-	TCGA-BA-A	TCGA-HNSC	49	'--	'--	'--	'--	-18052	69	not hispa	male	'--	'--	white	Dead
b7ac2f06-	TCGA-BA-A	TCGA-HNSC	49	'--	'--	'--	'--	-18052	69	not hispa	male	'--	'--	white	Dead
53d9a130-	TCGA-DQ-5	TCGA-HNSC	52	'--	'--	'--	'--	-19211	1133	not hispa	female	'--	'--	white	Dead
53d9a130-	TCGA-DQ-5	TCGA-HNSC	52	'--	'--	'--	'--	-19211	1133	not hispa	female	'--	'--	white	Dead
34aad752-	TCGA-CN-A	TCGA-HNSC	61	'--	'--	'--	'--	-22330	773	not hispa	female	'--	'--	white	Dead
34aad752-	TCGA-CN-A	TCGA-HNSC	61	'--	'--	'--	'--	-22330	773	not hispa	female	'--	'--	white	Dead
15e43ee8-	TCGA-F7-A	TCGA-HNSC	67	'--	'--	'--	'--	-24758	'--	not hispa	female	'--	'--	white	Alive
15e43ee8-	TCGA-F7-A	TCGA-HNSC	67	'--	'--	'--	'--	-24758	'--	not hispa	female	'--	'--	white	Alive
757c56d0-	TCGA-4P-A	TCGA-HNSC	66	'--	'--	'--	'--	-24222	'--	not hispa	male	'--	'--	black or	Alive
757c56d0-	TCGA-4P-A	TCGA-HNSC	66	'--	'--	'--	'--	-24222	'--	not hispa	male	'--	'--	black or	Alive
207c8a26-	TCGA-BA-A	TCGA-HNSC	24	'--	'--	'--	'--	-8967	'--	not hispa	female	'--	'--	white	Alive
207c8a26-	TCGA-BA-A	TCGA-HNSC	24	'--	'--	'--	'--	-8967	'--	not hispa	female	'--	'--	white	Alive
91a712b5-	TCGA-DQ-5	TCGA-HNSC	73	'--	'--	'--	'--	-26855	'--	not hispa	male	'--	'--	white	Alive
91a712b5-	TCGA-DQ-5	TCGA-HNSC	73	'--	'--	'--	'--	-26855	'--	not hispa	male	'--	'--	white	Alive

6a82a217-	TCGA-CV-A	TCGA-HNSC	65	'--	'--	'--	'--	-23806	'--	not hispa	male	'--	'--	white	Alive
6a82a217-	TCGA-CV-A	TCGA-HNSC	65	'--	'--	'--	'--	-23806	'--	not hispa	male	'--	'--	white	Alive
e1527704-	TCGA-CQ-A	TCGA-HNSC	76	'--	'--	'--	'--	-27793	'--	not repor	female	'--	'--	not repor	Alive
e1527704-	TCGA-CQ-A	TCGA-HNSC	76	'--	'--	'--	'--	-27793	'--	not repor	female	'--	'--	not repor	Alive
e7d1f0dd-	TCGA-CV-6	TCGA-HNSC	60	'--	'--	'--	'--	-21947	666	not repor	male	'--	'--	white	Dead
e7d1f0dd-	TCGA-CV-6	TCGA-HNSC	60	'--	'--	'--	'--	-21947	666	not repor	male	'--	'--	white	Dead
ebfb8c1b-	TCGA-H7-A	TCGA-HNSC	35	'--	'--	'--	'--	-13137	'--	not hispa	female	'--	'--	white	Dead
ebfb8c1b-	TCGA-H7-A	TCGA-HNSC	35	'--	'--	'--	'--	-13137	'--	not hispa	female	'--	'--	white	Dead
865c624a-	TCGA-CV-A	TCGA-HNSC	69	'--	'--	'--	'--	-25377	197	not hispa	male	'--	'--	white	Dead
865c624a-	TCGA-CV-A	TCGA-HNSC	69	'--	'--	'--	'--	-25377	197	not hispa	male	'--	'--	white	Dead
837bf19d-	TCGA-CV-A	TCGA-HNSC	64	'--	'--	'--	'--	-23452	4856	not hispa	female	'--	'--	white	Dead
837bf19d-	TCGA-CV-A	TCGA-HNSC	64	'--	'--	'--	'--	-23452	4856	not hispa	female	'--	'--	white	Dead
d86fd392-	TCGA-CQ-6	TCGA-HNSC	52	'--	'--	'--	'--	-19051	'--	not hispa	male	'--	'--	white	Alive
d86fd392-	TCGA-CQ-6	TCGA-HNSC	52	'--	'--	'--	'--	-19051	'--	not hispa	male	'--	'--	white	Alive
aa34f900-	TCGA-CV-7	TCGA-HNSC	61	'--	'--	'--	'--	-22349	393	not hispa	female	'--	'--	white	Dead
aa34f900-	TCGA-CV-7	TCGA-HNSC	61	'--	'--	'--	'--	-22349	393	not hispa	female	'--	'--	white	Dead
91728cd6-	TCGA-CV-6	TCGA-HNSC	57	'--	'--	'--	'--	-21166	915	not hispa	male	'--	'--	white	Dead
91728cd6-	TCGA-CV-6	TCGA-HNSC	57	'--	'--	'--	'--	-21166	915	not hispa	male	'--	'--	white	Dead
45c3cd39-	TCGA-CR-7	TCGA-HNSC	45	'--	'--	'--	'--	-16610	'--	not hispa	male	'--	'--	white	Alive
45c3cd39-	TCGA-CR-7	TCGA-HNSC	45	'--	'--	'--	'--	-16610	'--	not hispa	male	'--	'--	white	Alive
a40bb4bc-	TCGA-CR-7	TCGA-HNSC	44	'--	'--	'--	'--	-16351	'--	not hispa	male	'--	'--	white	Alive
a40bb4bc-	TCGA-CR-7	TCGA-HNSC	44	'--	'--	'--	'--	-16351	'--	not hispa	male	'--	'--	white	Alive
83aa326a-	TCGA-D6-8	TCGA-HNSC	52	'--	'--	'--	'--	-19212	'--	not hispa	male	'--	'--	white	Alive
83aa326a-	TCGA-D6-8	TCGA-HNSC	52	'--	'--	'--	'--	-19212	'--	not hispa	male	'--	'--	white	Alive
8f6c64c9-	TCGA-F7-A	TCGA-HNSC	51	'--	'--	'--	'--	-18879	'--	not hispa	male	'--	'--	asian	Alive
8f6c64c9-	TCGA-F7-A	TCGA-HNSC	51	'--	'--	'--	'--	-18879	'--	not hispa	male	'--	'--	asian	Alive
64260b33-	TCGA-CQ-6	TCGA-HNSC	52	'--	'--	'--	'--	-19096	'--	not hispa	female	'--	'--	white	Alive
64260b33-	TCGA-CQ-6	TCGA-HNSC	52	'--	'--	'--	'--	-19096	'--	not hispa	female	'--	'--	white	Alive
50239d1d-	TCGA-CV-5	TCGA-HNSC	66	'--	'--	'--	'--	-24115	'--	not hispa	male	'--	'--	white	Alive
50239d1d-	TCGA-CV-5	TCGA-HNSC	66	'--	'--	'--	'--	-24115	'--	not hispa	male	'--	'--	white	Alive
20ece2ca-	TCGA-CQ-7	TCGA-HNSC	40	'--	'--	'--	'--	-14676	'--	not hispa	male	'--	'--	asian	Alive
20ece2ca-	TCGA-CQ-7	TCGA-HNSC	40	'--	'--	'--	'--	-14676	'--	not hispa	male	'--	'--	asian	Alive
3be2ace5-	TCGA-CN-4	TCGA-HNSC	60	'--	'--	'--	'--	-22171	'--	not hispa	male	'--	'--	white	Alive
3be2ace5-	TCGA-CN-4	TCGA-HNSC	60	'--	'--	'--	'--	-22171	'--	not hispa	male	'--	'--	white	Alive
d69e29ca-	TCGA-IQ-A	TCGA-HNSC	54	'--	'--	'--	'--	-19913	'--	not hispa	male	'--	'--	white	Alive

d69e29ca-	TCGA-IQ-A	TCGA-HNSC	54	'--	'--	'--	'--	-19913	'--	not hispa	male	'--	'--	white	Alive
15714e69-	TCGA-CV-6	TCGA-HNSC	41	'--	'--	'--	'--	-15158	366	not hispa	male	'--	'--	white	Dead
15714e69-	TCGA-CV-6	TCGA-HNSC	41	'--	'--	'--	'--	-15158	366	not hispa	male	'--	'--	white	Dead
f88ec55c-	TCGA-UP-A	TCGA-HNSC	58	'--	'--	'--	'--	-21427	'--	hispanic	male	'--	'--	white	Alive
f88ec55c-	TCGA-UP-A	TCGA-HNSC	58	'--	'--	'--	'--	-21427	'--	hispanic	male	'--	'--	white	Alive
116ad004-	TCGA-BA-A	TCGA-HNSC	70	'--	'--	'--	'--	-25874	'--	not hispa	female	'--	'--	white	Alive
116ad004-	TCGA-BA-A	TCGA-HNSC	70	'--	'--	'--	'--	-25874	'--	not hispa	female	'--	'--	white	Alive
8c238d30-	TCGA-CV-6	TCGA-HNSC	67	'--	'--	'--	'--	-24720	217	hispanic	male	'--	'--	white	Dead
8c238d30-	TCGA-CV-6	TCGA-HNSC	67	'--	'--	'--	'--	-24720	217	hispanic	male	'--	'--	white	Dead
b1b3983d-	TCGA-CV-A	TCGA-HNSC	61	'--	'--	'--	'--	-22591	'--	not hispa	female	'--	'--	white	Alive
b1b3983d-	TCGA-CV-A	TCGA-HNSC	61	'--	'--	'--	'--	-22591	'--	not hispa	female	'--	'--	white	Alive
61d0709c-	TCGA-CN-5	TCGA-HNSC	60	'--	'--	'--	'--	-22246	352	not hispa	female	'--	'--	white	Dead
61d0709c-	TCGA-CN-5	TCGA-HNSC	60	'--	'--	'--	'--	-22246	352	not hispa	female	'--	'--	white	Dead
5a03e2b6-	TCGA-CQ-5	TCGA-HNSC	61	'--	'--	'--	'--	-22307	'--	not hispa	female	'--	'--	asian	Alive
5a03e2b6-	TCGA-CQ-5	TCGA-HNSC	61	'--	'--	'--	'--	-22307	'--	not hispa	female	'--	'--	asian	Alive
0f718e17-	TCGA-HD-7	TCGA-HNSC	74	'--	'--	'--	'--	-27334	'--	not hispa	male	'--	'--	white	Alive
0f718e17-	TCGA-HD-7	TCGA-HNSC	74	'--	'--	'--	'--	-27334	'--	not hispa	male	'--	'--	white	Alive
534f85f2-	TCGA-CV-7	TCGA-HNSC	32	'--	'--	'--	'--	-11931	64	not hispa	female	'--	'--	white	Dead
534f85f2-	TCGA-CV-7	TCGA-HNSC	32	'--	'--	'--	'--	-11931	64	not hispa	female	'--	'--	white	Dead
23062172-	TCGA-CV-5	TCGA-HNSC	50	'--	'--	'--	'--	-18267	'--	not hispa	male	'--	'--	white	Alive
23062172-	TCGA-CV-5	TCGA-HNSC	50	'--	'--	'--	'--	-18267	'--	not hispa	male	'--	'--	white	Alive
1465b7ec-	TCGA-CV-5	TCGA-HNSC	60	'--	'--	'--	'--	-22279	'--	not hispa	male	'--	'--	white	Alive
1465b7ec-	TCGA-CV-5	TCGA-HNSC	60	'--	'--	'--	'--	-22279	'--	not hispa	male	'--	'--	white	Alive
10b3f56a-	TCGA-DQ-5	TCGA-HNSC	52	'--	'--	'--	'--	-19172	548	not hispa	male	'--	'--	white	Dead
10b3f56a-	TCGA-DQ-5	TCGA-HNSC	52	'--	'--	'--	'--	-19172	548	not hispa	male	'--	'--	white	Dead
ccd672df-	TCGA-BB-A	TCGA-HNSC	61	'--	'--	'--	'--	-22506	268	not hispa	female	'--	'--	white	Dead
ccd672df-	TCGA-BB-A	TCGA-HNSC	61	'--	'--	'--	'--	-22506	268	not hispa	female	'--	'--	white	Dead
ea93555b-	TCGA-CV-6	TCGA-HNSC	51	'--	'--	'--	'--	-18812	342	not hispa	male	'--	'--	white	Dead
ea93555b-	TCGA-CV-6	TCGA-HNSC	51	'--	'--	'--	'--	-18812	342	not hispa	male	'--	'--	white	Dead
039788ec-	TCGA-CN-6	TCGA-HNSC	53	'--	'--	'--	'--	-19447	357	not hispa	male	'--	'--	white	Dead
039788ec-	TCGA-CN-6	TCGA-HNSC	53	'--	'--	'--	'--	-19447	357	not hispa	male	'--	'--	white	Dead
9180e195-	TCGA-CV-A	TCGA-HNSC	46	'--	'--	'--	'--	-17125	'--	hispanic	male	'--	'--	white	Alive
9180e195-	TCGA-CV-A	TCGA-HNSC	46	'--	'--	'--	'--	-17125	'--	hispanic	male	'--	'--	white	Alive
85ae3b0f-	TCGA-F7-A	TCGA-HNSC	66	'--	'--	'--	'--	-24285	'--	not hispa	male	'--	'--	white	Alive
85ae3b0f-	TCGA-F7-A	TCGA-HNSC	66	'--	'--	'--	'--	-24285	'--	not hispa	male	'--	'--	white	Alive

a63ca97d-	TCGA-BA-4	TCGA-HNSC	69	'--	'--	'--	'--	-25282	462	not hispa	male	'--	'--	white	Dead
a63ca97d-	TCGA-BA-4	TCGA-HNSC	69	'--	'--	'--	'--	-25282	462	not hispa	male	'--	'--	white	Dead
d622bcab-	TCGA-CQ-7	TCGA-HNSC	51	'--	'--	'--	'--	-18841	'--	not hispa	male	'--	'--	white	Alive
d622bcab-	TCGA-CQ-7	TCGA-HNSC	51	'--	'--	'--	'--	-18841	'--	not hispa	male	'--	'--	white	Alive
1f744ada-	TCGA-CN-4	TCGA-HNSC	19	'--	'--	'--	'--	-7301	'--	not hispa	male	'--	'--	white	Alive
1f744ada-	TCGA-CN-4	TCGA-HNSC	19	'--	'--	'--	'--	-7301	'--	not hispa	male	'--	'--	white	Alive
0fb3e5ff-	TCGA-CN-4	TCGA-HNSC	70	'--	'--	'--	'--	-25683	395	not hispa	female	'--	'--	white	Dead
0fb3e5ff-	TCGA-CN-4	TCGA-HNSC	70	'--	'--	'--	'--	-25683	395	not hispa	female	'--	'--	white	Dead
df37eed0-	TCGA-CQ-A	TCGA-HNSC	'--	'--	'--	'--	'--	'--	'--	not repor	male	'--	'--	not repor	Alive
df37eed0-	TCGA-CQ-A	TCGA-HNSC	'--	'--	'--	'--	'--	'--	'--	not repor	male	'--	'--	not repor	Alive
d5eedfb5-	TCGA-CV-6	TCGA-HNSC	57	'--	'--	'--	'--	-20855	'--	not hispa	male	'--	'--	white	Alive
d5eedfb5-	TCGA-CV-6	TCGA-HNSC	57	'--	'--	'--	'--	-20855	'--	not hispa	male	'--	'--	white	Alive
7ed006e8-	TCGA-CV-6	TCGA-HNSC	65	'--	'--	'--	'--	-23892	185	not hispa	female	'--	'--	white	Dead
7ed006e8-	TCGA-CV-6	TCGA-HNSC	65	'--	'--	'--	'--	-23892	185	not hispa	female	'--	'--	white	Dead
e8f0cd72-	TCGA-C9-A	TCGA-HNSC	45	'--	'--	'--	'--	-16567	'--	not hispa	female	'--	'--	asian	Alive
e8f0cd72-	TCGA-C9-A	TCGA-HNSC	45	'--	'--	'--	'--	-16567	'--	not hispa	female	'--	'--	asian	Alive
e2e64c5d-	TCGA-CR-7	TCGA-HNSC	64	'--	'--	'--	'--	-23420	'--	not hispa	male	'--	'--	white	Alive
e2e64c5d-	TCGA-CR-7	TCGA-HNSC	64	'--	'--	'--	'--	-23420	'--	not hispa	male	'--	'--	white	Alive
08a45833-	TCGA-CV-7	TCGA-HNSC	87	'--	'--	'--	'--	-32021	194	not hispa	female	'--	'--	white	Dead
08a45833-	TCGA-CV-7	TCGA-HNSC	87	'--	'--	'--	'--	-32021	194	not hispa	female	'--	'--	white	Dead
408c5d54-	TCGA-CQ-5	TCGA-HNSC	65	'--	'--	'--	'--	-23833	654	not hispa	male	'--	'--	white	Dead
408c5d54-	TCGA-CQ-5	TCGA-HNSC	65	'--	'--	'--	'--	-23833	654	not hispa	male	'--	'--	white	Dead
a1ded1e8-	TCGA-CN-A	TCGA-HNSC	57	'--	'--	'--	'--	-21011	82	not hispa	male	'--	'--	white	Dead
a1ded1e8-	TCGA-CN-A	TCGA-HNSC	57	'--	'--	'--	'--	-21011	82	not hispa	male	'--	'--	white	Dead
bf6b931e-	TCGA-BA-6	TCGA-HNSC	28	'--	'--	'--	'--	-10512	'--	hispanic	male	'--	'--	white	Alive
bf6b931e-	TCGA-BA-6	TCGA-HNSC	28	'--	'--	'--	'--	-10512	'--	hispanic	male	'--	'--	white	Alive
7a211ce6-	TCGA-F7-A	TCGA-HNSC	62	'--	'--	'--	'--	-22655	'--	not hispa	male	'--	'--	white	Alive
7a211ce6-	TCGA-F7-A	TCGA-HNSC	62	'--	'--	'--	'--	-22655	'--	not hispa	male	'--	'--	white	Alive
9bf40eeb-	TCGA-KU-A	TCGA-HNSC	41	'--	'--	'--	'--	-15066	327	not hispa	male	'--	'--	white	Dead
9bf40eeb-	TCGA-KU-A	TCGA-HNSC	41	'--	'--	'--	'--	-15066	327	not hispa	male	'--	'--	white	Dead
09f6c3ab-	TCGA-QK-A	TCGA-HNSC	60	'--	'--	'--	'--	-22093	'--	not hispa	male	'--	'--	black or	Alive
09f6c3ab-	TCGA-QK-A	TCGA-HNSC	60	'--	'--	'--	'--	-22093	'--	not hispa	male	'--	'--	black or	Alive
d3f786a6-	TCGA-IQ-A	TCGA-HNSC	55	'--	'--	'--	'--	-20417	'--	not hispa	female	'--	'--	white	Alive
d3f786a6-	TCGA-IQ-A	TCGA-HNSC	55	'--	'--	'--	'--	-20417	'--	not hispa	female	'--	'--	white	Alive
59398d50-	TCGA-CV-6	TCGA-HNSC	60	'--	'--	'--	'--	-22025	292	hispanic	male	'--	'--	white	Dead

59398d50-	TCGA-CV-6	TCGA-HNSC	60	'--	'--	'--	'--	-22025	292	hispanic	male	'--	'--	white	Dead
d5e136fd-	TCGA-CV-A	TCGA-HNSC	58	'--	'--	'--	'--	-21488	'--	not hispa	male	'--	'--	white	Alive
d5e136fd-	TCGA-CV-A	TCGA-HNSC	58	'--	'--	'--	'--	-21488	'--	not hispa	male	'--	'--	white	Alive
8e0e456e-	TCGA-D6-6	TCGA-HNSC	82	'--	'--	'--	'--	-30099	403	not hispa	female	'--	'--	white	Dead
8e0e456e-	TCGA-D6-6	TCGA-HNSC	82	'--	'--	'--	'--	-30099	403	not hispa	female	'--	'--	white	Dead
0f4bb968-	TCGA-CR-7	TCGA-HNSC	26	'--	'--	'--	'--	-9750	'--	not hispa	male	'--	'--	white	Alive
0f4bb968-	TCGA-CR-7	TCGA-HNSC	26	'--	'--	'--	'--	-9750	'--	not hispa	male	'--	'--	white	Alive
78fcc813-	TCGA-CV-7	TCGA-HNSC	69	'--	'--	'--	'--	-25471	'--	not hispa	female	'--	'--	white	Alive
78fcc813-	TCGA-CV-7	TCGA-HNSC	69	'--	'--	'--	'--	-25471	'--	not hispa	female	'--	'--	white	Alive
2fc48edb-	TCGA-CN-6	TCGA-HNSC	66	'--	'--	'--	'--	-24259	337	not hispa	male	'--	'--	white	Dead
2fc48edb-	TCGA-CN-6	TCGA-HNSC	66	'--	'--	'--	'--	-24259	337	not hispa	male	'--	'--	white	Dead
444e5bfc-	TCGA-CN-4	TCGA-HNSC	48	'--	'--	'--	'--	-17793	397	not hispa	female	'--	'--	white	Dead
444e5bfc-	TCGA-CN-4	TCGA-HNSC	48	'--	'--	'--	'--	-17793	397	not hispa	female	'--	'--	white	Dead
67f2ac9b-	TCGA-CR-7	TCGA-HNSC	67	'--	'--	'--	'--	-24575	'--	not hispa	male	'--	'--	white	Alive
67f2ac9b-	TCGA-CR-7	TCGA-HNSC	67	'--	'--	'--	'--	-24575	'--	not hispa	male	'--	'--	white	Alive
80d51546-	TCGA-P3-A	TCGA-HNSC	41	'--	'--	'--	'--	-15125	'--	not repor	male	'--	'--	not repor	Alive
80d51546-	TCGA-P3-A	TCGA-HNSC	41	'--	'--	'--	'--	-15125	'--	not repor	male	'--	'--	not repor	Alive
1bc03593-	TCGA-UF-A	TCGA-HNSC	59	'--	'--	'--	'--	-21676	680	not repor	male	'--	'--	white	Dead
1bc03593-	TCGA-UF-A	TCGA-HNSC	59	'--	'--	'--	'--	-21676	680	not repor	male	'--	'--	white	Dead
6f8b9c64-	TCGA-CV-A	TCGA-HNSC	24	'--	'--	'--	'--	-9015	215	hispanic	male	'--	'--	white	Dead
6f8b9c64-	TCGA-CV-A	TCGA-HNSC	24	'--	'--	'--	'--	-9015	215	hispanic	male	'--	'--	white	Dead
eb611a76-	TCGA-BA-7	TCGA-HNSC	61	'--	'--	'--	'--	-22609	'--	not hispa	male	'--	'--	white	Alive
eb611a76-	TCGA-BA-7	TCGA-HNSC	61	'--	'--	'--	'--	-22609	'--	not hispa	male	'--	'--	white	Alive
a091822c-	TCGA-CQ-6	TCGA-HNSC	61	'--	'--	'--	'--	-22541	'--	not hispa	male	'--	'--	white	Alive
a091822c-	TCGA-CQ-6	TCGA-HNSC	61	'--	'--	'--	'--	-22541	'--	not hispa	male	'--	'--	white	Alive
d82e6c52-	TCGA-DQ-7	TCGA-HNSC	57	'--	'--	'--	'--	-21041	'--	not hispa	male	'--	'--	american	Alive
d82e6c52-	TCGA-DQ-7	TCGA-HNSC	57	'--	'--	'--	'--	-21041	'--	not hispa	male	'--	'--	american	Alive
49eb77f3-	TCGA-QK-A	TCGA-HNSC	60	'--	'--	'--	'--	-22023	'--	not hispa	male	'--	'--	white	Alive
49eb77f3-	TCGA-QK-A	TCGA-HNSC	60	'--	'--	'--	'--	-22023	'--	not hispa	male	'--	'--	white	Alive
23c4e2eb-	TCGA-CN-6	TCGA-HNSC	55	'--	'--	'--	'--	-20358	853	not hispa	male	'--	'--	white	Dead
23c4e2eb-	TCGA-CN-6	TCGA-HNSC	55	'--	'--	'--	'--	-20358	853	not hispa	male	'--	'--	white	Dead
55a9e8ae-	TCGA-D6-A	TCGA-HNSC	59	'--	'--	'--	'--	-21659	'--	not hispa	male	'--	'--	white	Alive
55a9e8ae-	TCGA-D6-A	TCGA-HNSC	59	'--	'--	'--	'--	-21659	'--	not hispa	male	'--	'--	white	Alive
e08e1d99-	TCGA-D6-A	TCGA-HNSC	61	'--	'--	'--	'--	-22455	'--	not hispa	male	'--	'--	white	Alive
e08e1d99-	TCGA-D6-A	TCGA-HNSC	61	'--	'--	'--	'--	-22455	'--	not hispa	male	'--	'--	white	Alive

dedd71b4-	TCGA-CN-4	TCGA-HNSC	61	'--	'--	'--	'--	-22561	'--	not hispa	male	'--	'--	white	Alive
dedd71b4-	TCGA-CN-4	TCGA-HNSC	61	'--	'--	'--	'--	-22561	'--	not hispa	male	'--	'--	white	Alive
2a749317-	TCGA-CV-6	TCGA-HNSC	53	'--	'--	'--	'--	-19518	2741	not hispa	male	'--	'--	white	Dead
2a749317-	TCGA-CV-6	TCGA-HNSC	53	'--	'--	'--	'--	-19518	2741	not hispa	male	'--	'--	white	Dead
d2ca9a4d-	TCGA-CV-7	TCGA-HNSC	34	'--	'--	'--	'--	-12523	327	not hispa	male	'--	'--	white	Dead
d2ca9a4d-	TCGA-CV-7	TCGA-HNSC	34	'--	'--	'--	'--	-12523	327	not hispa	male	'--	'--	white	Dead
4199c571-	TCGA-WA-A	TCGA-HNSC	69	'--	'--	'--	'--	-25363	'--	not hispa	male	'--	'--	white	Alive
4199c571-	TCGA-WA-A	TCGA-HNSC	69	'--	'--	'--	'--	-25363	'--	not hispa	male	'--	'--	white	Alive
02dcc11f-	TCGA-CQ-5	TCGA-HNSC	46	'--	'--	'--	'--	-17028	'--	not hispa	female	'--	'--	white	Alive
02dcc11f-	TCGA-CQ-5	TCGA-HNSC	46	'--	'--	'--	'--	-17028	'--	not hispa	female	'--	'--	white	Alive
38cfe5af-	TCGA-CV-7	TCGA-HNSC	77	'--	'--	'--	'--	-28250	144	not hispa	female	'--	'--	white	Dead
38cfe5af-	TCGA-CV-7	TCGA-HNSC	77	'--	'--	'--	'--	-28250	144	not hispa	female	'--	'--	white	Dead
f458d21d-	TCGA-CR-6	TCGA-HNSC	68	'--	'--	'--	'--	-25176	'--	not hispa	female	'--	'--	white	Alive
f458d21d-	TCGA-CR-6	TCGA-HNSC	68	'--	'--	'--	'--	-25176	'--	not hispa	female	'--	'--	white	Alive
4bfbce2b-	TCGA-BA-4	TCGA-HNSC	45	'--	'--	'--	'--	-16536	1134	not hispa	female	'--	'--	white	Dead
4bfbce2b-	TCGA-BA-4	TCGA-HNSC	45	'--	'--	'--	'--	-16536	1134	not hispa	female	'--	'--	white	Dead
06a802b9-	TCGA-CQ-5	TCGA-HNSC	74	'--	'--	'--	'--	-27110	341	not hispa	male	'--	'--	white	Dead
06a802b9-	TCGA-CQ-5	TCGA-HNSC	74	'--	'--	'--	'--	-27110	341	not hispa	male	'--	'--	white	Dead
c2f1b2d5-	TCGA-IQ-A	TCGA-HNSC	61	'--	'--	'--	'--	-22529	'--	hispanic	female	'--	'--	white	Alive
c2f1b2d5-	TCGA-IQ-A	TCGA-HNSC	61	'--	'--	'--	'--	-22529	'--	hispanic	female	'--	'--	white	Alive
4f5bc340-	TCGA-CV-6	TCGA-HNSC	66	'--	'--	'--	'--	-24180	65	not hispa	female	'--	'--	white	Dead
4f5bc340-	TCGA-CV-6	TCGA-HNSC	66	'--	'--	'--	'--	-24180	65	not hispa	female	'--	'--	white	Dead
2a7b4613-	TCGA-CX-7	TCGA-HNSC	77	'--	'--	'--	'--	-28221	'--	not hispa	female	'--	'--	white	Alive
2a7b4613-	TCGA-CX-7	TCGA-HNSC	77	'--	'--	'--	'--	-28221	'--	not hispa	female	'--	'--	white	Alive
0ca3d25a-	TCGA-CQ-6	TCGA-HNSC	50	'--	'--	'--	'--	-18578	479	not hispa	female	'--	'--	white	Dead
0ca3d25a-	TCGA-CQ-6	TCGA-HNSC	50	'--	'--	'--	'--	-18578	479	not hispa	female	'--	'--	white	Dead
6fa62461-	TCGA-BA-4	TCGA-HNSC	49	'--	'--	'--	'--	-17951	283	not hispa	male	'--	'--	black or	Dead
6fa62461-	TCGA-BA-4	TCGA-HNSC	49	'--	'--	'--	'--	-17951	283	not hispa	male	'--	'--	black or	Dead
49b94421-	TCGA-CN-6	TCGA-HNSC	58	'--	'--	'--	'--	-21447	530	not hispa	female	'--	'--	white	Dead
49b94421-	TCGA-CN-6	TCGA-HNSC	58	'--	'--	'--	'--	-21447	530	not hispa	female	'--	'--	white	Dead
b843df7e-	TCGA-HD-8	TCGA-HNSC	61	'--	'--	'--	'--	-22340	'--	not hispa	female	'--	'--	white	Alive
b843df7e-	TCGA-HD-8	TCGA-HNSC	61	'--	'--	'--	'--	-22340	'--	not hispa	female	'--	'--	white	Alive
417b357e-	TCGA-CV-6	TCGA-HNSC	50	'--	'--	'--	'--	-18600	'--	not hispa	female	'--	'--	white	Alive
417b357e-	TCGA-CV-6	TCGA-HNSC	50	'--	'--	'--	'--	-18600	'--	not hispa	female	'--	'--	white	Alive
1e05fdbd-	TCGA-CV-5	TCGA-HNSC	26	'--	'--	'--	'--	-9567	'--	not hispa	male	'--	'--	white	Alive

1e05fdbd-	TCGA-CV-5	TCGA-HNSC	26	'--	'--	'--	'--	-9567	'--	not hispa	male	'--	'--	white	Alive
402fd96c-	TCGA-CV-6	TCGA-HNSC	59	'--	'--	'--	'--	-21557	2002	not repor	male	'--	'--	black or	Dead
402fd96c-	TCGA-CV-6	TCGA-HNSC	59	'--	'--	'--	'--	-21557	2002	not repor	male	'--	'--	black or	Dead
573e3713-	TCGA-IQ-A	TCGA-HNSC	55	'--	'--	'--	'--	-20109	'--	not hispa	male	'--	'--	black or	Alive
573e3713-	TCGA-IQ-A	TCGA-HNSC	55	'--	'--	'--	'--	-20109	'--	not hispa	male	'--	'--	black or	Alive
ffcf851d-	TCGA-CN-6	TCGA-HNSC	64	'--	'--	'--	'--	-23664	'--	not hispa	male	'--	'--	white	Alive
ffcf851d-	TCGA-CN-6	TCGA-HNSC	64	'--	'--	'--	'--	-23664	'--	not hispa	male	'--	'--	white	Alive
9ffa79fa-	TCGA-CQ-5	TCGA-HNSC	69	'--	'--	'--	'--	-25255	'--	not hispa	female	'--	'--	white	Alive
9ffa79fa-	TCGA-CQ-5	TCGA-HNSC	69	'--	'--	'--	'--	-25255	'--	not hispa	female	'--	'--	white	Alive
8fc1f1be-	TCGA-CV-5	TCGA-HNSC	62	'--	'--	'--	'--	-22771	'--	not hispa	female	'--	'--	white	Alive
8fc1f1be-	TCGA-CV-5	TCGA-HNSC	62	'--	'--	'--	'--	-22771	'--	not hispa	female	'--	'--	white	Alive
e1670628-	TCGA-CQ-6	TCGA-HNSC	63	'--	'--	'--	'--	-23326	'--	not hispa	male	'--	'--	white	Alive
e1670628-	TCGA-CQ-6	TCGA-HNSC	63	'--	'--	'--	'--	-23326	'--	not hispa	male	'--	'--	white	Alive
1c509ba7-	TCGA-CV-A	TCGA-HNSC	82	'--	'--	'--	'--	-30297	'--	not hispa	female	'--	'--	white	Alive
1c509ba7-	TCGA-CV-A	TCGA-HNSC	82	'--	'--	'--	'--	-30297	'--	not hispa	female	'--	'--	white	Alive
54dab158-	TCGA-CN-5	TCGA-HNSC	78	'--	'--	'--	'--	-28549	259	not hispa	male	'--	'--	white	Dead
54dab158-	TCGA-CN-5	TCGA-HNSC	78	'--	'--	'--	'--	-28549	259	not hispa	male	'--	'--	white	Dead
ee3061ec-	TCGA-CN-6	TCGA-HNSC	61	'--	'--	'--	'--	-22485	'--	not hispa	male	'--	'--	white	Alive
ee3061ec-	TCGA-CN-6	TCGA-HNSC	61	'--	'--	'--	'--	-22485	'--	not hispa	male	'--	'--	white	Alive
b43b8aed-	TCGA-CR-6	TCGA-HNSC	69	'--	'--	'--	'--	-25432	282	not hispa	male	'--	'--	black or	Dead
b43b8aed-	TCGA-CR-6	TCGA-HNSC	69	'--	'--	'--	'--	-25432	282	not hispa	male	'--	'--	black or	Dead
1f6167d9-	TCGA-CV-5	TCGA-HNSC	59	'--	'--	'--	'--	-21890	406	hispanic	male	'--	'--	white	Dead
1f6167d9-	TCGA-CV-5	TCGA-HNSC	59	'--	'--	'--	'--	-21890	406	hispanic	male	'--	'--	white	Dead
d296cdb5-	TCGA-CR-7	TCGA-HNSC	70	'--	'--	'--	'--	-25581	'--	not hispa	male	'--	'--	white	Alive
d296cdb5-	TCGA-CR-7	TCGA-HNSC	70	'--	'--	'--	'--	-25581	'--	not hispa	male	'--	'--	white	Alive
c4ad0479-	TCGA-CV-7	TCGA-HNSC	66	'--	'--	'--	'--	-24170	1093	not hispa	male	'--	'--	white	Dead
c4ad0479-	TCGA-CV-7	TCGA-HNSC	66	'--	'--	'--	'--	-24170	1093	not hispa	male	'--	'--	white	Dead
9205dc07-	TCGA-HD-A	TCGA-HNSC	79	'--	'--	'--	'--	-29045	'--	not hispa	female	'--	'--	white	Alive
9205dc07-	TCGA-HD-A	TCGA-HNSC	79	'--	'--	'--	'--	-29045	'--	not hispa	female	'--	'--	white	Alive
58574e35-	TCGA-BB-4	TCGA-HNSC	52	'--	'--	'--	'--	-19119	'--	not hispa	male	'--	'--	white	Alive
58574e35-	TCGA-BB-4	TCGA-HNSC	52	'--	'--	'--	'--	-19119	'--	not hispa	male	'--	'--	white	Alive
832316ee-	TCGA-CR-7	TCGA-HNSC	36	'--	'--	'--	'--	-13508	'--	not hispa	female	'--	'--	white	Alive
832316ee-	TCGA-CR-7	TCGA-HNSC	36	'--	'--	'--	'--	-13508	'--	not hispa	female	'--	'--	white	Alive
22933e67-	TCGA-HD-8	TCGA-HNSC	51	'--	'--	'--	'--	-18718	385	not hispa	female	'--	'--	white	Dead
22933e67-	TCGA-HD-8	TCGA-HNSC	51	'--	'--	'--	'--	-18718	385	not hispa	female	'--	'--	white	Dead

84fd72d1-	TCGA-CV-6	TCGA-HNSC	62	'--	'--	'--	'--	-22885	'--	not hispa	male	'--	'--	white	Alive
84fd72d1-	TCGA-CV-6	TCGA-HNSC	62	'--	'--	'--	'--	-22885	'--	not hispa	male	'--	'--	white	Alive
fbdab01c-	TCGA-D6-6	TCGA-HNSC	50	'--	'--	'--	'--	-18611	'--	not hispa	male	'--	'--	white	Alive
fbdab01c-	TCGA-D6-6	TCGA-HNSC	50	'--	'--	'--	'--	-18611	'--	not hispa	male	'--	'--	white	Alive
1dd23cd6-	TCGA-CV-7	TCGA-HNSC	49	'--	'--	'--	'--	-18219	1591	not hispa	male	'--	'--	white	Dead
1dd23cd6-	TCGA-CV-7	TCGA-HNSC	49	'--	'--	'--	'--	-18219	1591	not hispa	male	'--	'--	white	Dead
375e4b96-	TCGA-MT-A	TCGA-HNSC	30	'--	'--	'--	'--	-10976	'--	not hispa	male	'--	'--	white	Alive
375e4b96-	TCGA-MT-A	TCGA-HNSC	30	'--	'--	'--	'--	-10976	'--	not hispa	male	'--	'--	white	Alive
eda7df86-	TCGA-MT-A	TCGA-HNSC	85	'--	'--	'--	'--	-31161	'--	not hispa	female	'--	'--	white	Alive
eda7df86-	TCGA-MT-A	TCGA-HNSC	85	'--	'--	'--	'--	-31161	'--	not hispa	female	'--	'--	white	Alive

weeks_ges	year_of_b	year_of_d	age_at_di	ajcc_clin	ajcc_clin	ajcc_clin	ajcc_clin	ajcc_path	ajcc_path	ajcc_path	ajcc_path	ajcc_stag	anaplasia	anaplasia	ann_arbor
'--	1954	'--	21455	M0	N2c	Stage IVA	T4a	M0	N0	Stage II	T2	7th	'--	'--	'--
'--	1954	'--	21455	M0	N2c	Stage IVA	T4a	M0	N0	Stage II	T2	7th	'--	'--	'--
'--	1942	'--	24650	M0	N2b	Stage IVA	T3	M0	N2b	Stage IVA	T3	6th	'--	'--	'--
'--	1942	'--	24650	M0	N2b	Stage IVA	T3	M0	N2b	Stage IVA	T3	6th	'--	'--	'--
'--	1938	'--	26976	M0	N0	Stage III	T3	'--	N0	Stage I	T1	7th	'--	'--	'--
'--	1938	'--	26976	M0	N0	Stage III	T3	'--	N0	Stage I	T1	7th	'--	'--	'--
'--	1959	'--	18078	M0	N2c	Stage IVA	T2	M0	N2c	Stage IVA	T2	6th	'--	'--	'--
'--	1959	'--	18078	M0	N2c	Stage IVA	T2	M0	N2c	Stage IVA	T2	6th	'--	'--	'--
'--	1936	'--	27990	MX	N0	Stage II	T2	MX	N0	Stage II	T2	7th	'--	'--	'--
'--	1936	'--	27990	MX	N0	Stage II	T2	MX	N0	Stage II	T2	7th	'--	'--	'--
'--	1964	'--	15867	M0	N1	Stage IVA	T4a	'--	NX	'--	TX	7th	'--	'--	'--
'--	1964	'--	15867	M0	N1	Stage IVA	T4a	'--	NX	'--	TX	7th	'--	'--	'--
'--	1939	'--	26592	M0	N1	Stage III	T2	M0	N1	Stage III	T2	7th	'--	'--	'--
'--	1939	'--	26592	M0	N1	Stage III	T2	M0	N1	Stage III	T2	7th	'--	'--	'--
'--	1951	1999	17679	M0	N1	Stage III	T3	'--	N0	Stage III	T3	7th	'--	'--	'--
'--	1951	1999	17679	M0	N1	Stage III	T3	'--	N0	Stage III	T3	7th	'--	'--	'--
'--	1938	1999	22532	M0	N0	Stage II	T2	'--	N0	Stage II	T2	7th	'--	'--	'--
'--	1938	1999	22532	M0	N0	Stage II	T2	'--	N0	Stage II	T2	7th	'--	'--	'--
'--	1929	'--	29026	M0	N0	Stage II	T2	'--	NX	'--	T2	6th	'--	'--	'--
'--	1929	'--	29026	M0	N0	Stage II	T2	'--	NX	'--	T2	6th	'--	'--	'--
'--	1963	2012	18052	M0	N2b	Stage IVA	T4a	MX	NX	'--	TX	7th	'--	'--	'--
'--	1963	2012	18052	M0	N2b	Stage IVA	T4a	MX	NX	'--	TX	7th	'--	'--	'--
'--	1956	'--	19211	M0	N0	Stage II	T2	'--	NX	'--	TX	6th	'--	'--	'--
'--	1956	'--	19211	M0	N0	Stage II	T2	'--	NX	'--	TX	6th	'--	'--	'--
'--	1950	'--	22330	M0	N0	Stage II	T2	MX	N0	Stage III	T3	7th	'--	'--	'--
'--	1950	'--	22330	M0	N0	Stage II	T2	MX	N0	Stage III	T3	7th	'--	'--	'--
'--	1944	'--	24758	'--	'--	'--	'--	M0	N0	Stage III	T3	7th	'--	'--	'--
'--	1944	'--	24758	'--	'--	'--	'--	M0	N0	Stage III	T3	7th	'--	'--	'--
'--	1947	'--	24222	M0	N2a	Stage IVA	T4a	MX	N2c	Stage IVA	T2	7th	'--	'--	'--
'--	1947	'--	24222	M0	N2a	Stage IVA	T4a	MX	N2c	Stage IVA	T2	7th	'--	'--	'--
'--	1988	'--	8967	M0	N0	Stage II	T2	M0	N0	Stage I	T1	7th	'--	'--	'--
'--	1988	'--	8967	M0	N0	Stage II	T2	M0	N0	Stage I	T1	7th	'--	'--	'--
'--	1936	'--	26855	M0	N1	Stage III	T3	'--	NX	'--	TX	6th	'--	'--	'--
'--	1936	'--	26855	M0	N1	Stage III	T3	'--	NX	'--	TX	6th	'--	'--	'--

'--	1946	'--	23806	M0	N0	Stage II	T2	M0	N0	Stage II	T2	7th	'--	'--	'--
'--	1946	'--	23806	M0	N0	Stage II	T2	M0	N0	Stage II	T2	7th	'--	'--	'--
'--	1935	'--	27793	M0	N0	Stage II	T2	M0	N0	Stage II	T2	7th	'--	'--	'--
'--	1935	'--	27793	M0	N0	Stage II	T2	M0	N0	Stage II	T2	7th	'--	'--	'--
'--	1934	1995	21947	M0	N1	Stage IVA	T4a	'--	N2b	Stage IVA	T4a	7th	'--	'--	'--
'--	1934	1995	21947	M0	N1	Stage IVA	T4a	'--	N2b	Stage IVA	T4a	7th	'--	'--	'--
'--	1977	'--	13137	M0	N1	Stage III	T3	M0	N2b	Stage IVA	T2	7th	'--	'--	'--
'--	1977	'--	13137	M0	N1	Stage III	T3	M0	N2b	Stage IVA	T2	7th	'--	'--	'--
'--	1942	2011	25377	M0	N2c	Stage IVA	T4	M0	N2c	Stage IVA	T3	7th	'--	'--	'--
'--	1942	2011	25377	M0	N2c	Stage IVA	T4	M0	N2c	Stage IVA	T3	7th	'--	'--	'--
'--	1934	2011	23452	M0	N0	Stage II	T2	M0	N0	'--	T1	5th	'--	'--	'--
'--	1934	2011	23452	M0	N0	Stage II	T2	M0	N0	'--	T1	5th	'--	'--	'--
'--	1957	'--	19051	M0	N0	Stage II	T2	'--	N2b	Stage IVA	T2	6th	'--	'--	'--
'--	1957	'--	19051	M0	N0	Stage II	T2	'--	N2b	Stage IVA	T2	6th	'--	'--	'--
'--	1939	2001	22349	M0	N2	Stage IVA	T2	'--	N2b	Stage IVA	T2	7th	'--	'--	'--
'--	1939	2001	22349	M0	N2	Stage IVA	T2	'--	N2b	Stage IVA	T2	7th	'--	'--	'--
'--	1939	1998	21166	M0	N2c	Stage IVA	T4a	'--	N2c	Stage IVA	T4a	7th	'--	'--	'--
'--	1939	1998	21166	M0	N2c	Stage IVA	T4a	'--	N2c	Stage IVA	T4a	7th	'--	'--	'--
'--	1964	'--	16610	M0	N0	Stage II	T2	M0	N0	Stage I	T1	6th	'--	'--	'--
'--	1964	'--	16610	M0	N0	Stage II	T2	M0	N0	Stage I	T1	6th	'--	'--	'--
'--	1967	'--	16351	M0	N2b	Stage IVA	T3	M0	N2b	Stage IVA	T3	7th	'--	'--	'--
'--	1967	'--	16351	M0	N2b	Stage IVA	T3	M0	N2b	Stage IVA	T3	7th	'--	'--	'--
'--	1960	'--	19212	M0	N0	Stage II	T2	M0	N0	Stage II	T2	7th	'--	'--	'--
'--	1960	'--	19212	M0	N0	Stage II	T2	M0	N0	Stage II	T2	7th	'--	'--	'--
'--	1961	'--	18879	MX	NX	'--	TX	M0	N2b	Stage IVA	T2	7th	'--	'--	'--
'--	1961	'--	18879	MX	NX	'--	TX	M0	N2b	Stage IVA	T2	7th	'--	'--	'--
'--	1957	'--	19096	M0	N0	Stage III	T3	'--	N2b	Stage IVA	T3	6th	'--	'--	'--
'--	1957	'--	19096	M0	N0	Stage III	T3	'--	N2b	Stage IVA	T3	6th	'--	'--	'--
'--	1941	'--	24115	M0	N1	Stage III	T3	'--	N2b	Stage IVA	T3	7th	'--	'--	'--
'--	1941	'--	24115	M0	N1	Stage III	T3	'--	N2b	Stage IVA	T3	7th	'--	'--	'--
'--	1969	'--	14676	M0	N0	Stage II	T2	'--	N0	Stage II	T2	6th	'--	'--	'--
'--	1969	'--	14676	M0	N0	Stage II	T2	'--	N0	Stage II	T2	6th	'--	'--	'--
'--	1950	'--	22171	M0	N0	Stage II	T2	'--	N0	Stage II	T2	7th	'--	'--	'--
'--	1950	'--	22171	M0	N0	Stage II	T2	'--	N0	Stage II	T2	7th	'--	'--	'--
'--	1958	'--	19913	M0	N2b	Stage IVA	T2	M0	N2b	Stage IVA	T2	7th	'--	'--	'--

'--	1958	'--	19913	M0	N2b	Stage IVA	T2	M0	N2b	Stage IVA	T2	7th	'--	'--	'--
'--	1952	1994	15158	M0	N2b	Stage IVA	T4a	'--	N2	Stage IVA	T4a	7th	'--	'--	'--
'--	1952	1994	15158	M0	N2b	Stage IVA	T4a	'--	N2	Stage IVA	T4a	7th	'--	'--	'--
'--	1955	'--	21427	MX	N2c	Stage IVA	T2	'--	'--	'--	'--	7th	'--	'--	'--
'--	1955	'--	21427	MX	N2c	Stage IVA	T2	'--	'--	'--	'--	7th	'--	'--	'--
'--	1943	'--	25874	M0	N0	Stage III	T3	M0	N0	Stage II	T2	7th	'--	'--	'--
'--	1943	'--	25874	M0	N0	Stage III	T3	M0	N0	Stage II	T2	7th	'--	'--	'--
'--	1926	1993	24720	M0	N2c	Stage IVA	T4a	'--	N1	Stage III	T3	7th	'--	'--	'--
'--	1926	1993	24720	M0	N2c	Stage IVA	T4a	'--	N1	Stage III	T3	7th	'--	'--	'--
'--	1950	'--	22591	M0	N0	Stage IVA	T4a	M0	N0	Stage IVB	T4b	7th	'--	'--	'--
'--	1950	'--	22591	M0	N0	Stage IVA	T4a	M0	N0	Stage IVB	T4b	7th	'--	'--	'--
'--	1946	2006	22246	M0	N0	Stage IVA	T4a	M0	N2b	Stage IVA	T4a	6th	'--	'--	'--
'--	1946	2006	22246	M0	N0	Stage IVA	T4a	M0	N2b	Stage IVA	T4a	6th	'--	'--	'--
'--	1947	'--	22307	M0	N2c	Stage IVA	T3	'--	N2c	Stage IVA	T3	6th	'--	'--	'--
'--	1947	'--	22307	M0	N2c	Stage IVA	T3	'--	N2c	Stage IVA	T3	6th	'--	'--	'--
'--	1937	'--	27334	M0	N0	Stage III	T3	'--	N2	Stage IVA	T2	7th	'--	'--	'--
'--	1937	'--	27334	M0	N0	Stage III	T3	'--	N2	Stage IVA	T2	7th	'--	'--	'--
'--	1963	1995	11931	M0	N0	Stage II	T2	'--	N0	Stage IVA	T4a	7th	'--	'--	'--
'--	1963	1995	11931	M0	N0	Stage II	T2	'--	N0	Stage IVA	T4a	7th	'--	'--	'--
'--	1956	'--	18267	M0	N1	Stage III	T3	'--	N2b	Stage IVA	T4a	7th	'--	'--	'--
'--	1956	'--	18267	M0	N1	Stage III	T3	'--	N2b	Stage IVA	T4a	7th	'--	'--	'--
'--	1949	'--	22279	M0	N2b	Stage IVA	T2	'--	N2a	Stage IVA	T4a	7th	'--	'--	'--
'--	1949	'--	22279	M0	N2b	Stage IVA	T2	'--	N2a	Stage IVA	T4a	7th	'--	'--	'--
'--	1957	2010	19172	M0	N2b	Stage IVA	T3	'--	NX	'--	TX	6th	'--	'--	'--
'--	1957	2010	19172	M0	N2b	Stage IVA	T3	'--	NX	'--	TX	6th	'--	'--	'--
'--	1951	2012	22506	MX	N2	Stage IVA	T3	MX	N2b	Stage IVA	T4a	7th	'--	'--	'--
'--	1951	2012	22506	MX	N2	Stage IVA	T3	MX	N2b	Stage IVA	T4a	7th	'--	'--	'--
'--	1948	1999	18812	M0	N0	Stage III	T3	'--	N0	Stage III	T3	7th	'--	'--	'--
'--	1948	1999	18812	M0	N0	Stage III	T3	'--	N0	Stage III	T3	7th	'--	'--	'--
'--	1958	'--	19447	M0	N1	Stage IVA	T4a	'--	N2b	Stage IVA	T3	7th	'--	'--	'--
'--	1958	'--	19447	M0	N1	Stage IVA	T4a	'--	N2b	Stage IVA	T3	7th	'--	'--	'--
'--	1951	'--	17125	M0	N1	Stage III	T3	M0	N1	Stage III	T2	4th	'--	'--	'--
'--	1951	'--	17125	M0	N1	Stage III	T3	M0	N1	Stage III	T2	4th	'--	'--	'--
'--	1946	'--	24285	'--	'--	'--	'--	M0	N1	Stage III	T3	7th	'--	'--	'--
'--	1946	'--	24285	'--	'--	'--	'--	M0	N1	Stage III	T3	7th	'--	'--	'--

'--	1934	2004	25282	M0	N2c	Stage IVA	T3	M0	N2c	Stage IVA	T2	6th	'--	'--	'--
'--	1934	2004	25282	M0	N2c	Stage IVA	T3	M0	N2c	Stage IVA	T2	6th	'--	'--	'--
'--	1956	'--	18841	M0	N0	Stage IVA	T4a	M0	N0	Stage II	T2	6th	'--	'--	'--
'--	1956	'--	18841	M0	N0	Stage IVA	T4a	M0	N0	Stage II	T2	6th	'--	'--	'--
'--	1991	'--	7301	M0	N0	Stage II	T2	'--	N2b	Stage IVA	T2	7th	'--	'--	'--
'--	1991	'--	7301	M0	N0	Stage II	T2	'--	N2b	Stage IVA	T2	7th	'--	'--	'--
'--	1940	2011	25683	M0	N0	Stage II	T2	'--	NX	'--	T1	7th	'--	'--	'--
'--	1940	2011	25683	M0	N0	Stage II	T2	'--	NX	'--	T1	7th	'--	'--	'--
'--	'--	'--	'--	M0	N0	Stage II	T2	M0	N0	Stage II	T2	7th	'--	'--	'--
'--	'--	'--	'--	M0	N0	Stage II	T2	M0	N0	Stage II	T2	7th	'--	'--	'--
'--	1953	'--	20855	M0	N0	Stage II	T2	'--	N0	Stage II	T2	7th	'--	'--	'--
'--	1953	'--	20855	M0	N0	Stage II	T2	'--	N0	Stage II	T2	7th	'--	'--	'--
'--	1931	1996	23892	M0	N0	Stage III	T3	'--	N2b	Stage IVA	T3	7th	'--	'--	'--
'--	1931	1996	23892	M0	N0	Stage III	T3	'--	N2b	Stage IVA	T3	7th	'--	'--	'--
'--	1966	'--	16567	M0	N0	Stage III	T3	M0	N0	Stage III	T3	7th	'--	'--	'--
'--	1966	'--	16567	M0	N0	Stage III	T3	M0	N0	Stage III	T3	7th	'--	'--	'--
'--	1944	'--	23420	M0	N0	Stage I	T1	M0	N0	Stage I	T1	6th	'--	'--	'--
'--	1944	'--	23420	M0	N0	Stage I	T1	M0	N0	Stage I	T1	6th	'--	'--	'--
'--	1913	2000	32021	M0	N0	Stage II	T2	'--	NX	Stage I	T1	7th	'--	'--	'--
'--	1913	2000	32021	M0	N0	Stage II	T2	'--	NX	Stage I	T1	7th	'--	'--	'--
'--	1942	2008	23833	M0	N0	Stage I	T1	'--	N0	Stage I	T1	6th	'--	'--	'--
'--	1942	2008	23833	M0	N0	Stage I	T1	'--	N0	Stage I	T1	6th	'--	'--	'--
'--	1955	2012	21011	M0	N2c	Stage IVA	T3	M0	N3	Stage IVB	T4a	7th	'--	'--	'--
'--	1955	2012	21011	M0	N2c	Stage IVA	T3	M0	N3	Stage IVB	T4a	7th	'--	'--	'--
'--	1983	'--	10512	M0	N2b	Stage IVA	T2	'--	N2b	Stage IVA	T4a	7th	'--	'--	'--
'--	1983	'--	10512	M0	N2b	Stage IVA	T2	'--	N2b	Stage IVA	T4a	7th	'--	'--	'--
'--	1950	'--	22655	MX	NX	'--	TX	M0	N0	Stage III	T3	7th	'--	'--	'--
'--	1950	'--	22655	MX	NX	'--	TX	M0	N0	Stage III	T3	7th	'--	'--	'--
'--	1972	'--	15066	M0	N0	Stage I	T1	M0	N2b	Stage IVA	T2	7th	'--	'--	'--
'--	1972	'--	15066	M0	N0	Stage I	T1	M0	N2b	Stage IVA	T2	7th	'--	'--	'--
'--	1953	'--	22093	MX	N2b	Stage IVA	T3	MX	N2b	Stage IVA	T3	7th	'--	'--	'--
'--	1953	'--	22093	MX	N2b	Stage IVA	T3	MX	N2b	Stage IVA	T3	7th	'--	'--	'--
'--	1956	'--	20417	MX	N0	Stage III	T3	MX	N0	Stage III	T3	7th	'--	'--	'--
'--	1956	'--	20417	MX	N0	Stage III	T3	MX	N0	Stage III	T3	7th	'--	'--	'--
'--	1950	2010	22025	M0	N0	Stage III	T3	'--	N0	Stage III	T3	7th	'--	'--	'--

'--	1950	2010	22025	M0	N0	Stage III	T3	'--	N0	Stage III	T3	7th	'--	'--	'--
'--	1953	'--	21488	M0	N0	Stage II	T2	M0	N0	Stage I	T1	7th	'--	'--	'--
'--	1953	'--	21488	M0	N0	Stage II	T2	M0	N0	Stage I	T1	7th	'--	'--	'--
'--	1928	2011	30099	M0	N0	Stage II	T2	'--	N0	Stage II	T2	7th	'--	'--	'--
'--	1928	2011	30099	M0	N0	Stage II	T2	'--	N0	Stage II	T2	7th	'--	'--	'--
'--	1983	'--	9750	M0	N0	Stage I	T1	M0	N1	Stage III	T1	6th	'--	'--	'--
'--	1983	'--	9750	M0	N0	Stage I	T1	M0	N1	Stage III	T1	6th	'--	'--	'--
'--	1938	'--	25471	M0	N0	Stage II	T2	'--	N0	Stage II	T2	7th	'--	'--	'--
'--	1938	'--	25471	M0	N0	Stage II	T2	'--	N0	Stage II	T2	7th	'--	'--	'--
'--	1944	'--	24259	M0	N2	Stage IVA	T4a	M0	N2c	Stage IVA	T4a	6th	'--	'--	'--
'--	1944	'--	24259	M0	N2	Stage IVA	T4a	M0	N2c	Stage IVA	T4a	6th	'--	'--	'--
'--	1960	2009	17793	M0	N1	Stage IVA	T4a	M0	N2b	Stage IVA	T4a	6th	'--	'--	'--
'--	1960	2009	17793	M0	N1	Stage IVA	T4a	M0	N2b	Stage IVA	T4a	6th	'--	'--	'--
'--	1942	'--	24575	M0	N0	Stage III	T3	M0	N0	Stage IVA	T4a	6th	'--	'--	'--
'--	1942	'--	24575	M0	N0	Stage III	T3	M0	N0	Stage IVA	T4a	6th	'--	'--	'--
'--	1967	'--	15125	M0	N0	Stage II	T2	MX	NX	Stage I	T1	6th	'--	'--	'--
'--	1967	'--	15125	M0	N0	Stage II	T2	MX	NX	Stage I	T1	6th	'--	'--	'--
'--	1953	'--	21676	M0	N2b	Stage IVA	T3	M0	N2b	Stage IVA	T4a	7th	'--	'--	'--
'--	1953	'--	21676	M0	N2b	Stage IVA	T3	M0	N2b	Stage IVA	T4a	7th	'--	'--	'--
'--	1984	2008	9015	M0	N0	Stage II	T2	M0	N0	Stage III	T3	6th	'--	'--	'--
'--	1984	2008	9015	M0	N0	Stage II	T2	M0	N0	Stage III	T3	6th	'--	'--	'--
'--	1949	'--	22609	M0	N1	Stage III	T2	'--	N1	Stage III	T2	7th	'--	'--	'--
'--	1949	'--	22609	M0	N1	Stage III	T2	'--	N1	Stage III	T2	7th	'--	'--	'--
'--	1948	'--	22541	M0	N1	Stage III	T2	'--	N0	Stage II	T2	6th	'--	'--	'--
'--	1948	'--	22541	M0	N1	Stage III	T2	'--	N0	Stage II	T2	6th	'--	'--	'--
'--	1954	'--	21041	M0	N2b	Stage IVA	T4a	'--	NX	'--	TX	7th	'--	'--	'--
'--	1954	'--	21041	M0	N2b	Stage IVA	T4a	'--	NX	'--	TX	7th	'--	'--	'--
'--	1953	'--	22023	M0	N0	Stage II	T2	MX	N1	Stage III	T1	7th	'--	'--	'--
'--	1953	'--	22023	M0	N0	Stage II	T2	MX	N1	Stage III	T1	7th	'--	'--	'--
'--	1955	'--	20358	M0	N1	Stage III	T2	'--	N2b	Stage IVA	T3	7th	'--	'--	'--
'--	1955	'--	20358	M0	N1	Stage III	T2	'--	N2b	Stage IVA	T3	7th	'--	'--	'--
'--	1953	'--	21659	M0	N2	Stage IVA	T2	M0	N2	Stage IVA	T2	7th	'--	'--	'--
'--	1953	'--	21659	M0	N2	Stage IVA	T2	M0	N2	Stage IVA	T2	7th	'--	'--	'--
'--	1951	'--	22455	M0	N0	Stage III	T3	M0	N0	Stage III	T3	7th	'--	'--	'--
'--	1951	'--	22455	M0	N0	Stage III	T3	M0	N0	Stage III	T3	7th	'--	'--	'--

'--	1949	'--	22561	M0	N0	Stage I	T1	'--	N1	Stage III	T1	7th	'--	'--	'--
'--	1949	'--	22561	M0	N0	Stage I	T1	'--	N1	Stage III	T1	7th	'--	'--	'--
'--	1945	2005	19518	M0	N0	Stage IVA	T4a	'--	N1	Stage III	T3	7th	'--	'--	'--
'--	1945	2005	19518	M0	N0	Stage IVA	T4a	'--	N1	Stage III	T3	7th	'--	'--	'--
'--	1962	1996	12523	M0	N0	Stage II	T2	'--	NX	Stage II	T2	7th	'--	'--	'--
'--	1962	1996	12523	M0	N0	Stage II	T2	'--	NX	Stage II	T2	7th	'--	'--	'--
'--	1944	'--	25363	M0	N0	Stage II	T2	'--	N0	Stage II	T2	7th	'--	'--	'--
'--	1944	'--	25363	M0	N0	Stage II	T2	'--	N0	Stage II	T2	7th	'--	'--	'--
'--	1962	'--	17028	M0	N1	Stage III	T3	'--	N0	Stage II	T2	6th	'--	'--	'--
'--	1962	'--	17028	M0	N1	Stage III	T3	'--	N0	Stage II	T2	6th	'--	'--	'--
'--	1930	2007	28250	M0	N2c	Stage IVA	T2	'--	N2c	Stage IVA	T3	7th	'--	'--	'--
'--	1930	2007	28250	M0	N2c	Stage IVA	T2	'--	N2c	Stage IVA	T3	7th	'--	'--	'--
'--	1942	'--	25176	M0	N0	Stage II	T2	'--	N0	Stage II	T2	7th	'--	'--	'--
'--	1942	'--	25176	M0	N0	Stage II	T2	'--	N0	Stage II	T2	7th	'--	'--	'--
'--	1958	2006	16536	M0	N3	Stage IVB	T4b	M0	N0	Stage IVA	T4a	6th	'--	'--	'--
'--	1958	2006	16536	M0	N3	Stage IVB	T4b	M0	N0	Stage IVA	T4a	6th	'--	'--	'--
'--	1934	2008	27110	M0	N0	Stage I	T1	'--	N0	Stage II	T2	6th	'--	'--	'--
'--	1934	2008	27110	M0	N0	Stage I	T1	'--	N0	Stage II	T2	6th	'--	'--	'--
'--	1952	'--	22529	M0	N0	Stage III	T3	M0	N0	Stage III	T3	7th	'--	'--	'--
'--	1952	'--	22529	M0	N0	Stage III	T3	M0	N0	Stage III	T3	7th	'--	'--	'--
'--	1932	1998	24180	M0	N2b	Stage IVA	T3	'--	N2b	Stage IVA	T3	7th	'--	'--	'--
'--	1932	1998	24180	M0	N2b	Stage IVA	T3	'--	N2b	Stage IVA	T3	7th	'--	'--	'--
'--	1933	'--	28221	M0	N0	Stage III	T3	'--	N0	Stage I	T1	7th	'--	'--	'--
'--	1933	'--	28221	M0	N0	Stage III	T3	'--	N0	Stage I	T1	7th	'--	'--	'--
'--	1957	2008	18578	M0	N2b	Stage IVA	T3	'--	N2a	Stage IVA	T3	6th	'--	'--	'--
'--	1957	2008	18578	M0	N2b	Stage IVA	T3	'--	N2a	Stage IVA	T3	6th	'--	'--	'--
'--	1955	2004	17951	M0	N1	Stage IVA	T4a	M0	N0	Stage III	T3	6th	'--	'--	'--
'--	1955	2004	17951	M0	N1	Stage IVA	T4a	M0	N0	Stage III	T3	6th	'--	'--	'--
'--	1953	'--	21447	M0	N2b	Stage IVA	T3	'--	N2b	Stage IVA	T3	7th	'--	'--	'--
'--	1953	'--	21447	M0	N2b	Stage IVA	T3	'--	N2b	Stage IVA	T3	7th	'--	'--	'--
'--	1951	'--	22340	M0	N1	Stage III	T1	MX	N1	Stage III	T1	7th	'--	'--	'--
'--	1951	'--	22340	M0	N1	Stage III	T1	MX	N1	Stage III	T1	7th	'--	'--	'--
'--	1957	'--	18600	M0	N0	Stage II	T2	'--	N1	Stage III	T2	7th	'--	'--	'--
'--	1957	'--	18600	M0	N0	Stage II	T2	'--	N1	Stage III	T2	7th	'--	'--	'--
'--	1981	'--	9567	M0	N1	Stage III	T3	'--	N2b	Stage IVA	T2	7th	'--	'--	'--

'--	1981	'--	9567	M0	N1	Stage III	T3	'--	N2b	Stage IV/	T2	7th	'--	'--	'--
'--	1939	2003	21557	M0	N1	Stage IV/	T4a	'--	NX	Stage IV/	T4a	7th	'--	'--	'--
'--	1939	2003	21557	M0	N1	Stage IV/	T4a	'--	NX	Stage IV/	T4a	7th	'--	'--	'--
'--	1958	'--	20109	M1	N1	Stage IV(	T2	M0	N1	Stage III	T2	7th	'--	'--	'--
'--	1958	'--	20109	M1	N1	Stage IV(	T2	M0	N1	Stage III	T2	7th	'--	'--	'--
'--	1946	'--	23664	M0	N1	Stage IV/	T4a	'--	N1	Stage IV/	T4a	7th	'--	'--	'--
'--	1946	'--	23664	M0	N1	Stage IV/	T4a	'--	N1	Stage IV/	T4a	7th	'--	'--	'--
'--	1939	'--	25255	M0	N1	Stage III	T3	'--	N2b	Stage IV/	T3	6th	'--	'--	'--
'--	1939	'--	25255	M0	N1	Stage III	T3	'--	N2b	Stage IV/	T3	6th	'--	'--	'--
'--	1943	'--	22771	M0	N0	Stage III	T3	'--	N1	Stage III	T3	7th	'--	'--	'--
'--	1943	'--	22771	M0	N0	Stage III	T3	'--	N1	Stage III	T3	7th	'--	'--	'--
'--	1945	'--	23326	M0	N2b	Stage IV/	T3	'--	N2b	Stage IV/	T2	6th	'--	'--	'--
'--	1945	'--	23326	M0	N2b	Stage IV/	T3	'--	N2b	Stage IV/	T2	6th	'--	'--	'--
'--	1916	'--	30297	M0	N0	Stage II	T2	M0	N0	Stage I	T1	5th	'--	'--	'--
'--	1916	'--	30297	M0	N0	Stage II	T2	M0	N0	Stage I	T1	5th	'--	'--	'--
'--	1928	2006	28549	M0	N0	Stage II	T2	M0	N1	Stage III	T3	6th	'--	'--	'--
'--	1928	2006	28549	M0	N0	Stage II	T2	M0	N1	Stage III	T3	6th	'--	'--	'--
'--	1950	'--	22485	M0	N0	Stage IV/	T4a	'--	N0	Stage IV/	T4a	7th	'--	'--	'--
'--	1950	'--	22485	M0	N0	Stage IV/	T4a	'--	N0	Stage IV/	T4a	7th	'--	'--	'--
'--	1941	2010	25432	M0	N2b	Stage IV/	T3	'--	N2b	Stage IV/	T3	7th	'--	'--	'--
'--	1941	2010	25432	M0	N2b	Stage IV/	T3	'--	N2b	Stage IV/	T3	7th	'--	'--	'--
'--	1950	2010	21890	M0	N2b	Stage IV/	T4a	'--	N2b	Stage IV/	T4a	7th	'--	'--	'--
'--	1950	2010	21890	M0	N2b	Stage IV/	T4a	'--	N2b	Stage IV/	T4a	7th	'--	'--	'--
'--	1939	'--	25581	M0	N2c	Stage IV/	T3	M0	N0	Stage IV/	T4a	6th	'--	'--	'--
'--	1939	'--	25581	M0	N2c	Stage IV/	T3	M0	N0	Stage IV/	T4a	6th	'--	'--	'--
'--	1932	2000	24170	M0	N0	Stage II	T2	'--	N2b	Stage IV/	T2	7th	'--	'--	'--
'--	1932	2000	24170	M0	N0	Stage II	T2	'--	N2b	Stage IV/	T2	7th	'--	'--	'--
'--	1933	'--	29045	M0	N0	Stage II	T2	MX	N1	Stage III	T2	7th	'--	'--	'--
'--	1933	'--	29045	M0	N0	Stage II	T2	MX	N1	Stage III	T2	7th	'--	'--	'--
'--	1952	'--	19119	M0	N0	Stage III	T3	'--	N2b	Stage IV/	T2	7th	'--	'--	'--
'--	1952	'--	19119	M0	N0	Stage III	T3	'--	N2b	Stage IV/	T2	7th	'--	'--	'--
'--	1973	'--	13508	M0	N0	Stage I	T1	M0	N0	Stage I	T1	6th	'--	'--	'--
'--	1973	'--	13508	M0	N0	Stage I	T1	M0	N0	Stage I	T1	6th	'--	'--	'--
'--	1961	'--	18718	M0	NX	Stage I	T1	MX	N0	Stage I	T1	7th	'--	'--	'--
'--	1961	'--	18718	M0	NX	Stage I	T1	MX	N0	Stage I	T1	7th	'--	'--	'--

'--	1945	'--	22885	M0	N0	Stage III	T3	'--	N0	Stage IVA	T4a	7th	'--	'--	'--
'--	1945	'--	22885	M0	N0	Stage III	T3	'--	N0	Stage IVA	T4a	7th	'--	'--	'--
'--	1960	'--	18611	M0	N1	Stage III	T3	'--	N0	Stage II	T2	7th	'--	'--	'--
'--	1960	'--	18611	M0	N1	Stage III	T3	'--	N0	Stage II	T2	7th	'--	'--	'--
'--	1951	2004	18219	M0	N0	Stage II	T2	'--	N2b	Stage IVA	T2	7th	'--	'--	'--
'--	1951	2004	18219	M0	N0	Stage II	T2	'--	N2b	Stage IVA	T2	7th	'--	'--	'--
'--	1982	'--	10976	M0	N2b	Stage IVA	T1	M0	N2b	Stage IVA	T1	7th	'--	'--	'--
'--	1982	'--	10976	M0	N2b	Stage IVA	T1	M0	N2b	Stage IVA	T1	7th	'--	'--	'--
'--	1926	'--	31161	M0	N0	Stage I	T1	MX	N0	Stage I	T1	7th	'--	'--	'--
'--	1926	'--	31161	M0	N0	Stage I	T1	MX	N0	Stage I	T1	7th	'--	'--	'--

ann_arbor	ann_arbor	ann_arbor	best_over	breslow_t	burkitt_l	child_pug	circumfer	classific	cog_liver	cog_neuro	cog_renal	cog_rhabd	days_to_b	days_to_c	days_to_l
'--	'--	'--	'--	'--	'--	'--	'--	not repor	'--	'--	'--	'--	'--	0	371
'--	'--	'--	'--	'--	'--	'--	'--	not repor	'--	'--	'--	'--	'--	0	371
'--	'--	'--	'--	'--	'--	'--	'--	not repor	'--	'--	'--	'--	'--	0	1425
'--	'--	'--	'--	'--	'--	'--	'--	not repor	'--	'--	'--	'--	'--	0	1425
'--	'--	'--	'--	'--	'--	'--	'--	not repor	'--	'--	'--	'--	'--	0	491
'--	'--	'--	'--	'--	'--	'--	'--	not repor	'--	'--	'--	'--	'--	0	491
'--	'--	'--	'--	'--	'--	'--	'--	not repor	'--	'--	'--	'--	'--	0	796
'--	'--	'--	'--	'--	'--	'--	'--	not repor	'--	'--	'--	'--	'--	0	796
'--	'--	'--	'--	'--	'--	'--	'--	not repor	'--	'--	'--	'--	'--	0	1138
'--	'--	'--	'--	'--	'--	'--	'--	not repor	'--	'--	'--	'--	'--	0	1138
'--	'--	'--	'--	'--	'--	'--	'--	not repor	'--	'--	'--	'--	'--	0	1778
'--	'--	'--	'--	'--	'--	'--	'--	not repor	'--	'--	'--	'--	'--	0	1778
'--	'--	'--	'--	'--	'--	'--	'--	not repor	'--	'--	'--	'--	'--	0	5
'--	'--	'--	'--	'--	'--	'--	'--	not repor	'--	'--	'--	'--	'--	0	5
'--	'--	'--	'--	'--	'--	'--	'--	not repor	'--	'--	'--	'--	'--	0	'--
'--	'--	'--	'--	'--	'--	'--	'--	not repor	'--	'--	'--	'--	'--	0	'--
'--	'--	'--	'--	'--	'--	'--	'--	not repor	'--	'--	'--	'--	'--	0	'--
'--	'--	'--	'--	'--	'--	'--	'--	not repor	'--	'--	'--	'--	'--	0	'--
'--	'--	'--	'--	'--	'--	'--	'--	not repor	'--	'--	'--	'--	'--	0	1000
'--	'--	'--	'--	'--	'--	'--	'--	not repor	'--	'--	'--	'--	'--	0	1000
'--	'--	'--	'--	'--	'--	'--	'--	not repor	'--	'--	'--	'--	'--	0	'--
'--	'--	'--	'--	'--	'--	'--	'--	not repor	'--	'--	'--	'--	'--	0	'--
'--	'--	'--	'--	'--	'--	'--	'--	not repor	'--	'--	'--	'--	'--	0	1081
'--	'--	'--	'--	'--	'--	'--	'--	not repor	'--	'--	'--	'--	'--	0	1081
'--	'--	'--	'--	'--	'--	'--	'--	not repor	'--	'--	'--	'--	'--	0	443
'--	'--	'--	'--	'--	'--	'--	'--	not repor	'--	'--	'--	'--	'--	0	443
'--	'--	'--	'--	'--	'--	'--	'--	not repor	'--	'--	'--	'--	'--	0	947
'--	'--	'--	'--	'--	'--	'--	'--	not repor	'--	'--	'--	'--	'--	0	947
'--	'--	'--	'--	'--	'--	'--	'--	not repor	'--	'--	'--	'--	'--	0	102
'--	'--	'--	'--	'--	'--	'--	'--	not repor	'--	'--	'--	'--	'--	0	102
'--	'--	'--	'--	'--	'--	'--	'--	not repor	'--	'--	'--	'--	'--	0	216
'--	'--	'--	'--	'--	'--	'--	'--	not repor	'--	'--	'--	'--	'--	0	216
'--	'--	'--	'--	'--	'--	'--	'--	not repor	'--	'--	'--	'--	'--	0	1030
'--	'--	'--	'--	'--	'--	'--	'--	not repor	'--	'--	'--	'--	'--	0	1030

'--	'--	'--	'--	'--	'--	'--	'--	not repor	'--	'--	'--	'--	'--	0	852
'--	'--	'--	'--	'--	'--	'--	'--	not repor	'--	'--	'--	'--	'--	0	852
'--	'--	'--	'--	'--	'--	'--	'--	not repor	'--	'--	'--	'--	'--	0	897
'--	'--	'--	'--	'--	'--	'--	'--	not repor	'--	'--	'--	'--	'--	0	897
'--	'--	'--	'--	'--	'--	'--	'--	not repor	'--	'--	'--	'--	'--	0	'--
'--	'--	'--	'--	'--	'--	'--	'--	not repor	'--	'--	'--	'--	'--	0	'--
'--	'--	'--	'--	'--	'--	'--	'--	not repor	'--	'--	'--	'--	'--	0	414
'--	'--	'--	'--	'--	'--	'--	'--	not repor	'--	'--	'--	'--	'--	0	414
'--	'--	'--	'--	'--	'--	'--	'--	not repor	'--	'--	'--	'--	'--	0	'--
'--	'--	'--	'--	'--	'--	'--	'--	not repor	'--	'--	'--	'--	'--	0	'--
'--	'--	'--	'--	'--	'--	'--	'--	not repor	'--	'--	'--	'--	'--	0	'--
'--	'--	'--	'--	'--	'--	'--	'--	not repor	'--	'--	'--	'--	'--	0	'--
'--	'--	1721	'--	'--	'--	'--	'--	not repor	'--	'--	'--	'--	'--	0	1721
'--	'--	1721	'--	'--	'--	'--	'--	not repor	'--	'--	'--	'--	'--	0	1721
'--	'--	'--	'--	'--	'--	'--	'--	not repor	'--	'--	'--	'--	'--	0	'--
'--	'--	'--	'--	'--	'--	'--	'--	not repor	'--	'--	'--	'--	'--	0	'--
'--	'--	'--	'--	'--	'--	'--	'--	not repor	'--	'--	'--	'--	'--	0	'--
'--	'--	'--	'--	'--	'--	'--	'--	not repor	'--	'--	'--	'--	'--	0	'--
'--	'--	759	'--	'--	'--	'--	'--	not repor	'--	'--	'--	'--	'--	0	759
'--	'--	759	'--	'--	'--	'--	'--	not repor	'--	'--	'--	'--	'--	0	759
'--	'--	754	'--	'--	'--	'--	'--	not repor	'--	'--	'--	'--	'--	0	754
'--	'--	754	'--	'--	'--	'--	'--	not repor	'--	'--	'--	'--	'--	0	754
'--	'--	770	'--	'--	'--	'--	'--	not repor	'--	'--	'--	'--	'--	0	770
'--	'--	770	'--	'--	'--	'--	'--	not repor	'--	'--	'--	'--	'--	0	770
'--	'--	14	'--	'--	'--	'--	'--	not repor	'--	'--	'--	'--	'--	0	14
'--	'--	14	'--	'--	'--	'--	'--	not repor	'--	'--	'--	'--	'--	0	14
'--	'--	1253	'--	'--	'--	'--	'--	not repor	'--	'--	'--	'--	'--	0	1253
'--	'--	1253	'--	'--	'--	'--	'--	not repor	'--	'--	'--	'--	'--	0	1253
'--	'--	1840	'--	'--	'--	'--	'--	not repor	'--	'--	'--	'--	'--	0	1840
'--	'--	1840	'--	'--	'--	'--	'--	not repor	'--	'--	'--	'--	'--	0	1840
'--	'--	1628	'--	'--	'--	'--	'--	not repor	'--	'--	'--	'--	'--	0	1628
'--	'--	1628	'--	'--	'--	'--	'--	not repor	'--	'--	'--	'--	'--	0	1628
'--	'--	1157	'--	'--	'--	'--	'--	not repor	'--	'--	'--	'--	'--	0	1157
'--	'--	1157	'--	'--	'--	'--	'--	not repor	'--	'--	'--	'--	'--	0	1157
'--	'--	'--	'--	'--	'--	'--	'--	not repor	'--	'--	'--	'--	'--	0	1021

'--	'--	'--	'--	'--	'--	'--	'--	not repor	'--	'--	'--	'--	'--	0	1021
'--	'--	'--	'--	'--	'--	'--	'--	not repor	'--	'--	'--	'--	'--	0	'--
'--	'--	'--	'--	'--	'--	'--	'--	not repor	'--	'--	'--	'--	'--	0	'--
'--	'--	'--	'--	'--	'--	'--	'--	not repor	'--	'--	'--	'--	'--	0	518
'--	'--	'--	'--	'--	'--	'--	'--	not repor	'--	'--	'--	'--	'--	0	518
'--	'--	'--	'--	'--	'--	'--	'--	not repor	'--	'--	'--	'--	'--	0	440
'--	'--	'--	'--	'--	'--	'--	'--	not repor	'--	'--	'--	'--	'--	0	440
'--	'--	'--	'--	'--	'--	'--	'--	not repor	'--	'--	'--	'--	'--	0	'--
'--	'--	'--	'--	'--	'--	'--	'--	not repor	'--	'--	'--	'--	'--	0	'--
'--	'--	'--	'--	'--	'--	'--	'--	not repor	'--	'--	'--	'--	'--	0	110
'--	'--	'--	'--	'--	'--	'--	'--	not repor	'--	'--	'--	'--	'--	0	110
'--	'--	'--	'--	'--	'--	'--	'--	not repor	'--	'--	'--	'--	'--	0	'--
'--	'--	'--	'--	'--	'--	'--	'--	not repor	'--	'--	'--	'--	'--	0	'--
'--	'--	'--	'--	'--	'--	'--	'--	not repor	'--	'--	'--	'--	'--	0	1660
'--	'--	'--	'--	'--	'--	'--	'--	not repor	'--	'--	'--	'--	'--	0	1660
'--	'--	'--	'--	'--	'--	'--	'--	not repor	'--	'--	'--	'--	'--	0	667
'--	'--	'--	'--	'--	'--	'--	'--	not repor	'--	'--	'--	'--	'--	0	667
'--	'--	'--	'--	'--	'--	'--	'--	not repor	'--	'--	'--	'--	'--	0	'--
'--	'--	'--	'--	'--	'--	'--	'--	not repor	'--	'--	'--	'--	'--	0	'--
'--	'--	1478	'--	'--	'--	'--	'--	not repor	'--	'--	'--	'--	'--	0	1478
'--	'--	1478	'--	'--	'--	'--	'--	not repor	'--	'--	'--	'--	'--	0	1478
'--	'--	701	'--	'--	'--	'--	'--	not repor	'--	'--	'--	'--	'--	0	701
'--	'--	701	'--	'--	'--	'--	'--	not repor	'--	'--	'--	'--	'--	0	701
'--	'--	548	'--	'--	'--	'--	'--	not repor	'--	'--	'--	'--	'--	0	548
'--	'--	548	'--	'--	'--	'--	'--	not repor	'--	'--	'--	'--	'--	0	548
'--	'--	'--	'--	'--	'--	'--	'--	not repor	'--	'--	'--	'--	'--	0	'--
'--	'--	'--	'--	'--	'--	'--	'--	not repor	'--	'--	'--	'--	'--	0	'--
'--	'--	'--	'--	'--	'--	'--	'--	not repor	'--	'--	'--	'--	'--	0	'--
'--	'--	'--	'--	'--	'--	'--	'--	not repor	'--	'--	'--	'--	'--	0	'--
'--	'--	'--	'--	'--	'--	'--	'--	not repor	'--	'--	'--	'--	'--	0	46
'--	'--	'--	'--	'--	'--	'--	'--	not repor	'--	'--	'--	'--	'--	0	46
'--	'--	'--	'--	'--	'--	'--	'--	not repor	'--	'--	'--	'--	'--	0	5480
'--	'--	'--	'--	'--	'--	'--	'--	not repor	'--	'--	'--	'--	'--	0	5480
'--	'--	'--	'--	'--	'--	'--	'--	not repor	'--	'--	'--	'--	'--	0	616
'--	'--	'--	'--	'--	'--	'--	'--	not repor	'--	'--	'--	'--	'--	0	616

'--	'--	'--	'--	'--	'--	'--	'--	not repor	'--	'--	'--	'--	'--	0	'--
'--	'--	'--	'--	'--	'--	'--	'--	not repor	'--	'--	'--	'--	'--	0	'--
'--	'--	'--	'--	'--	'--	'--	'--	not repor	'--	'--	'--	'--	'--	0	2359
'--	'--	'--	'--	'--	'--	'--	'--	not repor	'--	'--	'--	'--	'--	0	2359
'--	'--	'--	'--	'--	'--	'--	'--	not repor	'--	'--	'--	'--	'--	0	625
'--	'--	'--	'--	'--	'--	'--	'--	not repor	'--	'--	'--	'--	'--	0	625
'--	'--	'--	'--	'--	'--	'--	'--	not repor	'--	'--	'--	'--	'--	0	'--
'--	'--	'--	'--	'--	'--	'--	'--	not repor	'--	'--	'--	'--	'--	0	'--
'--	'--	'--	'--	'--	'--	'--	'--	not repor	'--	'--	'--	'--	'--	'--	'--
'--	'--	'--	'--	'--	'--	'--	'--	not repor	'--	'--	'--	'--	'--	'--	'--
'--	'--	'--	'--	'--	'--	'--	'--	not repor	'--	'--	'--	'--	'--	0	641
'--	'--	'--	'--	'--	'--	'--	'--	not repor	'--	'--	'--	'--	'--	0	641
'--	'--	'--	'--	'--	'--	'--	'--	not repor	'--	'--	'--	'--	'--	0	'--
'--	'--	'--	'--	'--	'--	'--	'--	not repor	'--	'--	'--	'--	'--	0	'--
'--	'--	'--	'--	'--	'--	'--	'--	not repor	'--	'--	'--	'--	'--	0	386
'--	'--	'--	'--	'--	'--	'--	'--	not repor	'--	'--	'--	'--	'--	0	386
'--	'--	1077	'--	'--	'--	'--	'--	not repor	'--	'--	'--	'--	'--	0	1077
'--	'--	1077	'--	'--	'--	'--	'--	not repor	'--	'--	'--	'--	'--	0	1077
'--	'--	'--	'--	'--	'--	'--	'--	not repor	'--	'--	'--	'--	'--	0	'--
'--	'--	'--	'--	'--	'--	'--	'--	not repor	'--	'--	'--	'--	'--	0	'--
'--	'--	'--	'--	'--	'--	'--	'--	not repor	'--	'--	'--	'--	'--	0	'--
'--	'--	'--	'--	'--	'--	'--	'--	not repor	'--	'--	'--	'--	'--	0	'--
'--	'--	'--	'--	'--	'--	'--	'--	not repor	'--	'--	'--	'--	'--	0	'--
'--	'--	'--	'--	'--	'--	'--	'--	not repor	'--	'--	'--	'--	'--	0	'--
'--	'--	'--	'--	'--	'--	'--	'--	not repor	'--	'--	'--	'--	'--	0	122
'--	'--	'--	'--	'--	'--	'--	'--	not repor	'--	'--	'--	'--	'--	0	122
'--	'--	'--	'--	'--	'--	'--	'--	not repor	'--	'--	'--	'--	'--	0	576
'--	'--	'--	'--	'--	'--	'--	'--	not repor	'--	'--	'--	'--	'--	0	576
'--	'--	'--	'--	'--	'--	'--	'--	not repor	'--	'--	'--	'--	'--	0	168
'--	'--	'--	'--	'--	'--	'--	'--	not repor	'--	'--	'--	'--	'--	0	168
'--	'--	'--	'--	'--	'--	'--	'--	not repor	'--	'--	'--	'--	'--	0	253
'--	'--	'--	'--	'--	'--	'--	'--	not repor	'--	'--	'--	'--	'--	0	253
'--	'--	1147	'--	'--	'--	'--	'--	not repor	'--	'--	'--	'--	'--	0	1147
'--	'--	1147	'--	'--	'--	'--	'--	not repor	'--	'--	'--	'--	'--	0	1147
'--	'--	'--	'--	'--	'--	'--	'--	not repor	'--	'--	'--	'--	'--	0	'--

'--	'--	'--	'--	'--	'--	'--	'--	not repor	'--	'--	'--	'--	'--	0	'--
'--	'--	'--	'--	'--	'--	'--	'--	not repor	'--	'--	'--	'--	'--	0	606
'--	'--	'--	'--	'--	'--	'--	'--	not repor	'--	'--	'--	'--	'--	0	606
'--	'--	'--	'--	'--	'--	'--	'--	not repor	'--	'--	'--	'--	'--	0	'--
'--	'--	'--	'--	'--	'--	'--	'--	not repor	'--	'--	'--	'--	'--	0	'--
'--	'--	'--	'--	'--	'--	'--	'--	not repor	'--	'--	'--	'--	'--	0	993
'--	'--	'--	'--	'--	'--	'--	'--	not repor	'--	'--	'--	'--	'--	0	993
'--	'--	'--	'--	'--	'--	'--	'--	not repor	'--	'--	'--	'--	'--	0	2727
'--	'--	'--	'--	'--	'--	'--	'--	not repor	'--	'--	'--	'--	'--	0	2727
'--	'--	'--	'--	'--	'--	'--	'--	not repor	'--	'--	'--	'--	'--	0	224
'--	'--	'--	'--	'--	'--	'--	'--	not repor	'--	'--	'--	'--	'--	0	224
'--	'--	'--	'--	'--	'--	'--	'--	not repor	'--	'--	'--	'--	'--	0	'--
'--	'--	'--	'--	'--	'--	'--	'--	not repor	'--	'--	'--	'--	'--	0	'--
'--	'--	'--	'--	'--	'--	'--	'--	not repor	'--	'--	'--	'--	'--	0	1508
'--	'--	'--	'--	'--	'--	'--	'--	not repor	'--	'--	'--	'--	'--	0	1508
'--	'--	'--	'--	'--	'--	'--	'--	not repor	'--	'--	'--	'--	'--	0	2182
'--	'--	'--	'--	'--	'--	'--	'--	not repor	'--	'--	'--	'--	'--	0	2182
'--	'--	'--	'--	'--	'--	'--	'--	not repor	'--	'--	'--	'--	'--	0	413
'--	'--	'--	'--	'--	'--	'--	'--	not repor	'--	'--	'--	'--	'--	0	413
'--	'--	'--	'--	'--	'--	'--	'--	not repor	'--	'--	'--	'--	'--	0	'--
'--	'--	'--	'--	'--	'--	'--	'--	not repor	'--	'--	'--	'--	'--	0	'--
'--	'--	'--	'--	'--	'--	'--	'--	not repor	'--	'--	'--	'--	'--	0	1273
'--	'--	'--	'--	'--	'--	'--	'--	not repor	'--	'--	'--	'--	'--	0	1273
'--	'--	'--	'--	'--	'--	'--	'--	not repor	'--	'--	'--	'--	'--	0	1179
'--	'--	'--	'--	'--	'--	'--	'--	not repor	'--	'--	'--	'--	'--	0	1179
'--	'--	'--	'--	'--	'--	'--	'--	not repor	'--	'--	'--	'--	'--	0	1143
'--	'--	'--	'--	'--	'--	'--	'--	not repor	'--	'--	'--	'--	'--	0	1143
'--	'--	'--	'--	'--	'--	'--	'--	not repor	'--	'--	'--	'--	'--	0	645
'--	'--	'--	'--	'--	'--	'--	'--	not repor	'--	'--	'--	'--	'--	0	645
'--	'--	'--	'--	'--	'--	'--	'--	not repor	'--	'--	'--	'--	'--	0	629
'--	'--	'--	'--	'--	'--	'--	'--	not repor	'--	'--	'--	'--	'--	0	629
'--	'--	'--	'--	'--	'--	'--	'--	not repor	'--	'--	'--	'--	'--	0	539
'--	'--	'--	'--	'--	'--	'--	'--	not repor	'--	'--	'--	'--	'--	0	539
'--	'--	'--	'--	'--	'--	'--	'--	not repor	'--	'--	'--	'--	'--	0	376
'--	'--	'--	'--	'--	'--	'--	'--	not repor	'--	'--	'--	'--	'--	0	376

'--	'--	'--	'--	'--	'--	'--	'--	not repor	'--	'--	'--	'--	'--	0	1586
'--	'--	'--	'--	'--	'--	'--	'--	not repor	'--	'--	'--	'--	'--	0	1586
'--	'--	'--	'--	'--	'--	'--	'--	not repor	'--	'--	'--	'--	'--	0	'--
'--	'--	'--	'--	'--	'--	'--	'--	not repor	'--	'--	'--	'--	'--	0	'--
'--	'--	'--	'--	'--	'--	'--	'--	not repor	'--	'--	'--	'--	'--	0	'--
'--	'--	'--	'--	'--	'--	'--	'--	not repor	'--	'--	'--	'--	'--	0	'--
'--	'--	'--	'--	'--	'--	'--	'--	not repor	'--	'--	'--	'--	'--	0	443
'--	'--	'--	'--	'--	'--	'--	'--	not repor	'--	'--	'--	'--	'--	0	443
'--	'--	'--	'--	'--	'--	'--	'--	not repor	'--	'--	'--	'--	'--	0	2143
'--	'--	'--	'--	'--	'--	'--	'--	not repor	'--	'--	'--	'--	'--	0	2143
'--	'--	'--	'--	'--	'--	'--	'--	not repor	'--	'--	'--	'--	'--	0	'--
'--	'--	'--	'--	'--	'--	'--	'--	not repor	'--	'--	'--	'--	'--	0	'--
'--	'--	'--	'--	'--	'--	'--	'--	not repor	'--	'--	'--	'--	'--	0	379
'--	'--	'--	'--	'--	'--	'--	'--	not repor	'--	'--	'--	'--	'--	0	379
'--	'--	'--	'--	'--	'--	'--	'--	not repor	'--	'--	'--	'--	'--	0	'--
'--	'--	'--	'--	'--	'--	'--	'--	not repor	'--	'--	'--	'--	'--	0	'--
'--	'--	'--	'--	'--	'--	'--	'--	not repor	'--	'--	'--	'--	'--	0	'--
'--	'--	'--	'--	'--	'--	'--	'--	not repor	'--	'--	'--	'--	'--	0	'--
'--	'--	'--	'--	'--	'--	'--	'--	not repor	'--	'--	'--	'--	'--	0	579
'--	'--	'--	'--	'--	'--	'--	'--	not repor	'--	'--	'--	'--	'--	0	579
'--	'--	'--	'--	'--	'--	'--	'--	not repor	'--	'--	'--	'--	'--	0	'--
'--	'--	'--	'--	'--	'--	'--	'--	not repor	'--	'--	'--	'--	'--	0	'--
'--	'--	'--	'--	'--	'--	'--	'--	not repor	'--	'--	'--	'--	'--	0	321
'--	'--	'--	'--	'--	'--	'--	'--	not repor	'--	'--	'--	'--	'--	0	321
'--	'--	'--	'--	'--	'--	'--	'--	not repor	'--	'--	'--	'--	'--	0	'--
'--	'--	'--	'--	'--	'--	'--	'--	not repor	'--	'--	'--	'--	'--	0	'--
'--	'--	'--	'--	'--	'--	'--	'--	not repor	'--	'--	'--	'--	'--	0	'--
'--	'--	'--	'--	'--	'--	'--	'--	not repor	'--	'--	'--	'--	'--	0	'--
'--	'--	'--	'--	'--	'--	'--	'--	not repor	'--	'--	'--	'--	'--	0	432
'--	'--	'--	'--	'--	'--	'--	'--	not repor	'--	'--	'--	'--	'--	0	432
'--	'--	'--	'--	'--	'--	'--	'--	not repor	'--	'--	'--	'--	'--	0	695
'--	'--	'--	'--	'--	'--	'--	'--	not repor	'--	'--	'--	'--	'--	0	695
'--	'--	'--	'--	'--	'--	'--	'--	not repor	'--	'--	'--	'--	'--	0	1665
'--	'--	'--	'--	'--	'--	'--	'--	not repor	'--	'--	'--	'--	'--	0	1665
'--	'--	'--	'--	'--	'--	'--	'--	not repor	'--	'--	'--	'--	'--	0	1315

'--	'--	'--	'--	'--	'--	'--	'--	not repor	'--	'--	'--	'--	'--	0	1315
'--	'--	'--	'--	'--	'--	'--	'--	not repor	'--	'--	'--	'--	'--	0	'--
'--	'--	'--	'--	'--	'--	'--	'--	not repor	'--	'--	'--	'--	'--	0	'--
'--	'--	'--	'--	'--	'--	'--	'--	not repor	'--	'--	'--	'--	'--	0	471
'--	'--	'--	'--	'--	'--	'--	'--	not repor	'--	'--	'--	'--	'--	0	471
'--	'--	'--	'--	'--	'--	'--	'--	not repor	'--	'--	'--	'--	'--	0	1443
'--	'--	'--	'--	'--	'--	'--	'--	not repor	'--	'--	'--	'--	'--	0	1443
'--	'--	'--	'--	'--	'--	'--	'--	not repor	'--	'--	'--	'--	'--	0	1897
'--	'--	'--	'--	'--	'--	'--	'--	not repor	'--	'--	'--	'--	'--	0	1897
'--	'--	'--	'--	'--	'--	'--	'--	not repor	'--	'--	'--	'--	'--	0	2641
'--	'--	'--	'--	'--	'--	'--	'--	not repor	'--	'--	'--	'--	'--	0	2641
'--	'--	'--	'--	'--	'--	'--	'--	not repor	'--	'--	'--	'--	'--	0	2016
'--	'--	'--	'--	'--	'--	'--	'--	not repor	'--	'--	'--	'--	'--	0	2016
'--	'--	'--	'--	'--	'--	'--	'--	not repor	'--	'--	'--	'--	'--	0	639
'--	'--	'--	'--	'--	'--	'--	'--	not repor	'--	'--	'--	'--	'--	0	639
'--	'--	'--	'--	'--	'--	'--	'--	not repor	'--	'--	'--	'--	'--	0	'--
'--	'--	'--	'--	'--	'--	'--	'--	not repor	'--	'--	'--	'--	'--	0	'--
'--	'--	'--	'--	'--	'--	'--	'--	not repor	'--	'--	'--	'--	'--	0	1038
'--	'--	'--	'--	'--	'--	'--	'--	not repor	'--	'--	'--	'--	'--	0	1038
'--	'--	'--	'--	'--	'--	'--	'--	not repor	'--	'--	'--	'--	'--	0	'--
'--	'--	'--	'--	'--	'--	'--	'--	not repor	'--	'--	'--	'--	'--	0	'--
'--	'--	'--	'--	'--	'--	'--	'--	not repor	'--	'--	'--	'--	'--	0	406
'--	'--	'--	'--	'--	'--	'--	'--	not repor	'--	'--	'--	'--	'--	0	406
'--	'--	'--	'--	'--	'--	'--	'--	not repor	'--	'--	'--	'--	'--	0	1346
'--	'--	'--	'--	'--	'--	'--	'--	not repor	'--	'--	'--	'--	'--	0	1346
'--	'--	'--	'--	'--	'--	'--	'--	not repor	'--	'--	'--	'--	'--	0	'--
'--	'--	'--	'--	'--	'--	'--	'--	not repor	'--	'--	'--	'--	'--	0	'--
'--	'--	'--	'--	'--	'--	'--	'--	not repor	'--	'--	'--	'--	'--	0	111
'--	'--	'--	'--	'--	'--	'--	'--	not repor	'--	'--	'--	'--	'--	0	111
'--	'--	'--	'--	'--	'--	'--	'--	not repor	'--	'--	'--	'--	'--	0	278
'--	'--	'--	'--	'--	'--	'--	'--	not repor	'--	'--	'--	'--	'--	0	278
'--	'--	'--	'--	'--	'--	'--	'--	not repor	'--	'--	'--	'--	'--	0	913
'--	'--	'--	'--	'--	'--	'--	'--	not repor	'--	'--	'--	'--	'--	0	913
'--	'--	'--	'--	'--	'--	'--	'--	not repor	'--	'--	'--	'--	'--	0	24
'--	'--	'--	'--	'--	'--	'--	'--	not repor	'--	'--	'--	'--	'--	0	24

'--	'--	'--	'--	'--	'--	'--	'--	not repor	'--	'--	'--	'--	'--	0	1899
'--	'--	'--	'--	'--	'--	'--	'--	not repor	'--	'--	'--	'--	'--	0	1899
'--	'--	'--	'--	'--	'--	'--	'--	not repor	'--	'--	'--	'--	'--	0	701
'--	'--	'--	'--	'--	'--	'--	'--	not repor	'--	'--	'--	'--	'--	0	701
'--	'--	'--	'--	'--	'--	'--	'--	not repor	'--	'--	'--	'--	'--	0	'--
'--	'--	'--	'--	'--	'--	'--	'--	not repor	'--	'--	'--	'--	'--	0	'--
'--	'--	'--	'--	'--	'--	'--	'--	not repor	'--	'--	'--	'--	'--	0	242
'--	'--	'--	'--	'--	'--	'--	'--	not repor	'--	'--	'--	'--	'--	0	242
'--	'--	'--	'--	'--	'--	'--	'--	not repor	'--	'--	'--	'--	'--	0	914
'--	'--	'--	'--	'--	'--	'--	'--	not repor	'--	'--	'--	'--	'--	0	914

days_to_l	days_to_r	eln_risk_	enneking_	enneking_	enneking_	enneking_	esophagea	esophagea	figo_stag	figo_stag	first_sym	gastric_e	gleason_g	gleason_g	gleason_p
'__	'__	'__	'__	'__	'__	'__	'__	'__	'__	'__	'__	'__	'__	'__	'__
'__	'__	'__	'__	'__	'__	'__	'__	'__	'__	'__	'__	'__	'__	'__	'__
'__	'__	'__	'__	'__	'__	'__	'__	'__	'__	'__	'__	'__	'__	'__	'__
'__	'__	'__	'__	'__	'__	'__	'__	'__	'__	'__	'__	'__	'__	'__	'__
'__	'__	'__	'__	'__	'__	'__	'__	'__	'__	'__	'__	'__	'__	'__	'__
'__	'__	'__	'__	'__	'__	'__	'__	'__	'__	'__	'__	'__	'__	'__	'__
'__	'__	'__	'__	'__	'__	'__	'__	'__	'__	'__	'__	'__	'__	'__	'__
'__	'__	'__	'__	'__	'__	'__	'__	'__	'__	'__	'__	'__	'__	'__	'__
'__	'__	'__	'__	'__	'__	'__	'__	'__	'__	'__	'__	'__	'__	'__	'__
'__	'__	'__	'__	'__	'__	'__	'__	'__	'__	'__	'__	'__	'__	'__	'__
'__	'__	'__	'__	'__	'__	'__	'__	'__	'__	'__	'__	'__	'__	'__	'__
'__	'__	'__	'__	'__	'__	'__	'__	'__	'__	'__	'__	'__	'__	'__	'__
'__	'__	'__	'__	'__	'__	'__	'__	'__	'__	'__	'__	'__	'__	'__	'__
'__	'__	'__	'__	'__	'__	'__	'__	'__	'__	'__	'__	'__	'__	'__	'__
'__	'__	'__	'__	'__	'__	'__	'__	'__	'__	'__	'__	'__	'__	'__	'__
'__	'__	'__	'__	'__	'__	'__	'__	'__	'__	'__	'__	'__	'__	'__	'__
'__	'__	'__	'__	'__	'__	'__	'__	'__	'__	'__	'__	'__	'__	'__	'__
'__	'__	'__	'__	'__	'__	'__	'__	'__	'__	'__	'__	'__	'__	'__	'__
'__	'__	'__	'__	'__	'__	'__	'__	'__	'__	'__	'__	'__	'__	'__	'__
'__	'__	'__	'__	'__	'__	'__	'__	'__	'__	'__	'__	'__	'__	'__	'__
'__	'__	'__	'__	'__	'__	'__	'__	'__	'__	'__	'__	'__	'__	'__	'__
'__	'__	'__	'__	'__	'__	'__	'__	'__	'__	'__	'__	'__	'__	'__	'__
'__	'__	'__	'__	'__	'__	'__	'__	'__	'__	'__	'__	'__	'__	'__	'__
'__	'__	'__	'__	'__	'__	'__	'__	'__	'__	'__	'__	'__	'__	'__	'__
'__	'__	'__	'__	'__	'__	'__	'__	'__	'__	'__	'__	'__	'__	'__	'__
'__	'__	'__	'__	'__	'__	'__	'__	'__	'__	'__	'__	'__	'__	'__	'__
'__	'__	'__	'__	'__	'__	'__	'__	'__	'__	'__	'__	'__	'__	'__	'__
'__	'__	'__	'__	'__	'__	'__	'__	'__	'__	'__	'__	'__	'__	'__	'__
'__	'__	'__	'__	'__	'__	'__	'__	'__	'__	'__	'__	'__	'__	'__	'__
'__	'__	'__	'__	'__	'__	'__	'__	'__	'__	'__	'__	'__	'__	'__	'__
'__	'__	'__	'__	'__	'__	'__	'__	'__	'__	'__	'__	'__	'__	'__	'__
'__	'__	'__	'__	'__	'__	'__	'__	'__	'__	'__	'__	'__	'__	'__	'__
'__	'__	'__	'__	'__	'__	'__	'__	'__	'__	'__	'__	'__	'__	'__	'__
'__	'__	'__	'__	'__	'__	'__	'__	'__	'__	'__	'__	'__	'__	'__	'__

, __ , __ , __ , __ , __ , __ , __ , __ , __ , __ , __ , __ , __ , __ , __ , __
, __ , __ , __ , __ , __ , __ , __ , __ , __ , __ , __ , __ , __ , __ , __ , __
, __ , __ , __ , __ , __ , __ , __ , __ , __ , __ , __ , __ , __ , __ , __ , __
, __ , __ , __ , __ , __ , __ , __ , __ , __ , __ , __ , __ , __ , __ , __ , __
, __ , __ , __ , __ , __ , __ , __ , __ , __ , __ , __ , __ , __ , __ , __ , __
, __ , __ , __ , __ , __ , __ , __ , __ , __ , __ , __ , __ , __ , __ , __ , __
, __ , __ , __ , __ , __ , __ , __ , __ , __ , __ , __ , __ , __ , __ , __ , __
, __ , __ , __ , __ , __ , __ , __ , __ , __ , __ , __ , __ , __ , __ , __ , __
, __ , __ , __ , __ , __ , __ , __ , __ , __ , __ , __ , __ , __ , __ , __ , __
, __ , __ , __ , __ , __ , __ , __ , __ , __ , __ , __ , __ , __ , __ , __ , __
, __ , __ , __ , __ , __ , __ , __ , __ , __ , __ , __ , __ , __ , __ , __ , __
, __ , __ , __ , __ , __ , __ , __ , __ , __ , __ , __ , __ , __ , __ , __ , __
, __ , __ , __ , __ , __ , __ , __ , __ , __ , __ , __ , __ , __ , __ , __ , __
, __ , __ , __ , __ , __ , __ , __ , __ , __ , __ , __ , __ , __ , __ , __ , __
, __ , __ , __ , __ , __ , __ , __ , __ , __ , __ , __ , __ , __ , __ , __ , __
, __ , __ , __ , __ , __ , __ , __ , __ , __ , __ , __ , __ , __ , __ , __ , __
, __ , __ , __ , __ , __ , __ , __ , __ , __ , __ , __ , __ , __ , __ , __ , __
, __ , __ , __ , __ , __ , __ , __ , __ , __ , __ , __ , __ , __ , __ , __ , __
, __ , __ , __ , __ , __ , __ , __ , __ , __ , __ , __ , __ , __ , __ , __ , __
, __ , __ , __ , __ , __ , __ , __ , __ , __ , __ , __ , __ , __ , __ , __ , __
, __ , __ , __ , __ , __ , __ , __ , __ , __ , __ , __ , __ , __ , __ , __ , __
, __ , __ , __ , __ , __ , __ , __ , __ , __ , __ , __ , __ , __ , __ , __ , __
, __ , __ , __ , __ , __ , __ , __ , __ , __ , __ , __ , __ , __ , __ , __ , __
, __ , __ , __ , __ , __ , __ , __ , __ , __ , __ , __ , __ , __ , __ , __ , __
, __ , __ , __ , __ , __ , __ , __ , __ , __ , __ , __ , __ , __ , __ , __ , __
, __ , __ , __ , __ , __ , __ , __ , __ , __ , __ , __ , __ , __ , __ , __ , __
, __ , __ , __ , __ , __ , __ , __ , __ , __ , __ , __ , __ , __ , __ , __ , __
, __ , __ , __ , __ , __ , __ , __ , __ , __ , __ , __ , __ , __ , __ , __ , __
, __ , __ , __ , __ , __ , __ , __ , __ , __ , __ , __ , __ , __ , __ , __ , __
, __ , __ , __ , __ , __ , __ , __ , __ , __ , __ , __ , __ , __ , __ , __ , __
, __ , __ , __ , __ , __ , __ , __ , __ , __ , __ , __ , __ , __ , __ , __ , __
, __ , __ , __ , __ , __ , __ , __ , __ , __ , __ , __ , __ , __ , __ , __ , __
, __ , __ , __ , __ , __ , __ , __ , __ , __ , __ , __ , __ , __ , __ , __ , __
, __ , __ , __ , __ , __ , __ , __ , __ , __ , __ , __ , __ , __ , __ , __ , __
, __ , __ , __ , __ , __ , __ , __ , __ , __ , __ , __ , __ , __ , __ , __ , __

, __	, __	, __	, __	, __	, __	, __	, __	, __	, __	, __	, __	, __	, __	, __	, __
, __	, __	, __	, __	, __	, __	, __	, __	, __	, __	, __	, __	, __	, __	, __	, __
, __	, __	, __	, __	, __	, __	, __	, __	, __	, __	, __	, __	, __	, __	, __	, __
, __	, __	, __	, __	, __	, __	, __	, __	, __	, __	, __	, __	, __	, __	, __	, __
, __	, __	, __	, __	, __	, __	, __	, __	, __	, __	, __	, __	, __	, __	, __	, __
, __	, __	, __	, __	, __	, __	, __	, __	, __	, __	, __	, __	, __	, __	, __	, __
, __	, __	, __	, __	, __	, __	, __	, __	, __	, __	, __	, __	, __	, __	, __	, __
, __	, __	, __	, __	, __	, __	, __	, __	, __	, __	, __	, __	, __	, __	, __	, __
, __	, __	, __	, __	, __	, __	, __	, __	, __	, __	, __	, __	, __	, __	, __	, __
, __	, __	, __	, __	, __	, __	, __	, __	, __	, __	, __	, __	, __	, __	, __	, __
, __	, __	, __	, __	, __	, __	, __	, __	, __	, __	, __	, __	, __	, __	, __	, __
, __	, __	, __	, __	, __	, __	, __	, __	, __	, __	, __	, __	, __	, __	, __	, __
, __	, __	, __	, __	, __	, __	, __	, __	, __	, __	, __	, __	, __	, __	, __	, __
, __	, __	, __	, __	, __	, __	, __	, __	, __	, __	, __	, __	, __	, __	, __	, __
, __	, __	, __	, __	, __	, __	, __	, __	, __	, __	, __	, __	, __	, __	, __	, __
, __	, __	, __	, __	, __	, __	, __	, __	, __	, __	, __	, __	, __	, __	, __	, __
, __	, __	, __	, __	, __	, __	, __	, __	, __	, __	, __	, __	, __	, __	, __	, __
, __	, __	, __	, __	, __	, __	, __	, __	, __	, __	, __	, __	, __	, __	, __	, __
, __	, __	, __	, __	, __	, __	, __	, __	, __	, __	, __	, __	, __	, __	, __	, __
, __	, __	, __	, __	, __	, __	, __	, __	, __	, __	, __	, __	, __	, __	, __	, __
, __	, __	, __	, __	, __	, __	, __	, __	, __	, __	, __	, __	, __	, __	, __	, __
, __	, __	, __	, __	, __	, __	, __	, __	, __	, __	, __	, __	, __	, __	, __	, __
, __	, __	, __	, __	, __	, __	, __	, __	, __	, __	, __	, __	, __	, __	, __	, __
, __	, __	, __	, __	, __	, __	, __	, __	, __	, __	, __	, __	, __	, __	, __	, __
, __	, __	, __	, __	, __	, __	, __	, __	, __	, __	, __	, __	, __	, __	, __	, __
, __	, __	, __	, __	, __	, __	, __	, __	, __	, __	, __	, __	, __	, __	, __	, __
, __	, __	, __	, __	, __	, __	, __	, __	, __	, __	, __	, __	, __	, __	, __	, __
, __	, __	, __	, __	, __	, __	, __	, __	, __	, __	, __	, __	, __	, __	, __	, __
, __	, __	, __	, __	, __	, __	, __	, __	, __	, __	, __	, __	, __	, __	, __	, __
, __	, __	, __	, __	, __	, __	, __	, __	, __	, __	, __	, __	, __	, __	, __	, __
, __	, __	, __	, __	, __	, __	, __	, __	, __	, __	, __	, __	, __	, __	, __	, __
, __	, __	, __	, __	, __	, __	, __	, __	, __	, __	, __	, __	, __	, __	, __	, __
, __	, __	, __	, __	, __	, __	, __	, __	, __	, __	, __	, __	, __	, __	, __	, __
, __	, __	, __	, __	, __	, __	, __	, __	, __	, __	, __	, __	, __	, __	, __	, __
, __	, __	, __	, __	, __	, __	, __	, __	, __	, __	, __	, __	, __	, __	, __	, __

, __	, __	, __	, __	, __	, __	, __	, __	, __	, __	, __	, __	, __	, __	, __	, __
, __	, __	, __	, __	, __	, __	, __	, __	, __	, __	, __	, __	, __	, __	, __	, __
, __	, __	, __	, __	, __	, __	, __	, __	, __	, __	, __	, __	, __	, __	, __	, __
, __	, __	, __	, __	, __	, __	, __	, __	, __	, __	, __	, __	, __	, __	, __	, __
, __	, __	, __	, __	, __	, __	, __	, __	, __	, __	, __	, __	, __	, __	, __	, __
, __	, __	, __	, __	, __	, __	, __	, __	, __	, __	, __	, __	, __	, __	, __	, __
, __	, __	, __	, __	, __	, __	, __	, __	, __	, __	, __	, __	, __	, __	, __	, __
, __	, __	, __	, __	, __	, __	, __	, __	, __	, __	, __	, __	, __	, __	, __	, __
, __	, __	, __	, __	, __	, __	, __	, __	, __	, __	, __	, __	, __	, __	, __	, __
, __	, __	, __	, __	, __	, __	, __	, __	, __	, __	, __	, __	, __	, __	, __	, __
, __	, __	, __	, __	, __	, __	, __	, __	, __	, __	, __	, __	, __	, __	, __	, __
, __	, __	, __	, __	, __	, __	, __	, __	, __	, __	, __	, __	, __	, __	, __	, __
, __	, __	, __	, __	, __	, __	, __	, __	, __	, __	, __	, __	, __	, __	, __	, __
, __	, __	, __	, __	, __	, __	, __	, __	, __	, __	, __	, __	, __	, __	, __	, __
, __	, __	, __	, __	, __	, __	, __	, __	, __	, __	, __	, __	, __	, __	, __	, __
, __	, __	, __	, __	, __	, __	, __	, __	, __	, __	, __	, __	, __	, __	, __	, __
, __	, __	, __	, __	, __	, __	, __	, __	, __	, __	, __	, __	, __	, __	, __	, __
, __	, __	, __	, __	, __	, __	, __	, __	, __	, __	, __	, __	, __	, __	, __	, __
, __	, __	, __	, __	, __	, __	, __	, __	, __	, __	, __	, __	, __	, __	, __	, __
, __	, __	, __	, __	, __	, __	, __	, __	, __	, __	, __	, __	, __	, __	, __	, __
, __	, __	, __	, __	, __	, __	, __	, __	, __	, __	, __	, __	, __	, __	, __	, __
, __	, __	, __	, __	, __	, __	, __	, __	, __	, __	, __	, __	, __	, __	, __	, __
, __	, __	, __	, __	, __	, __	, __	, __	, __	, __	, __	, __	, __	, __	, __	, __
, __	, __	, __	, __	, __	, __	, __	, __	, __	, __	, __	, __	, __	, __	, __	, __
, __	, __	, __	, __	, __	, __	, __	, __	, __	, __	, __	, __	, __	, __	, __	, __
, __	, __	, __	, __	, __	, __	, __	, __	, __	, __	, __	, __	, __	, __	, __	, __
, __	, __	, __	, __	, __	, __	, __	, __	, __	, __	, __	, __	, __	, __	, __	, __
, __	, __	, __	, __	, __	, __	, __	, __	, __	, __	, __	, __	, __	, __	, __	, __
, __	, __	, __	, __	, __	, __	, __	, __	, __	, __	, __	, __	, __	, __	, __	, __
, __	, __	, __	, __	, __	, __	, __	, __	, __	, __	, __	, __	, __	, __	, __	, __
, __	, __	, __	, __	, __	, __	, __	, __	, __	, __	, __	, __	, __	, __	, __	, __
, __	, __	, __	, __	, __	, __	, __	, __	, __	, __	, __	, __	, __	, __	, __	, __
, __	, __	, __	, __	, __	, __	, __	, __	, __	, __	, __	, __	, __	, __	, __	, __
, __	, __	, __	, __	, __	, __	, __	, __	, __	, __	, __	, __	, __	, __	, __	, __
, __	, __	, __	, __	, __	, __	, __	, __	, __	, __	, __	, __	, __	, __	, __	, __

’ __ ’ __ ’ __ ’ __ ’ __ ’ __ ’ __ ’ __ ’ __ ’ __ ’ __ ’ __ ’ __ ’ __ ’ __ ’ __
’ __ ’ __ ’ __ ’ __ ’ __ ’ __ ’ __ ’ __ ’ __ ’ __ ’ __ ’ __ ’ __ ’ __ ’ __ ’ __
’ __ ’ __ ’ __ ’ __ ’ __ ’ __ ’ __ ’ __ ’ __ ’ __ ’ __ ’ __ ’ __ ’ __ ’ __ ’ __
’ __ ’ __ ’ __ ’ __ ’ __ ’ __ ’ __ ’ __ ’ __ ’ __ ’ __ ’ __ ’ __ ’ __ ’ __ ’ __
’ __ ’ __ ’ __ ’ __ ’ __ ’ __ ’ __ ’ __ ’ __ ’ __ ’ __ ’ __ ’ __ ’ __ ’ __ ’ __
’ __ ’ __ ’ __ ’ __ ’ __ ’ __ ’ __ ’ __ ’ __ ’ __ ’ __ ’ __ ’ __ ’ __ ’ __ ’ __
’ __ ’ __ ’ __ ’ __ ’ __ ’ __ ’ __ ’ __ ’ __ ’ __ ’ __ ’ __ ’ __ ’ __ ’ __ ’ __
’ __ ’ __ ’ __ ’ __ ’ __ ’ __ ’ __ ’ __ ’ __ ’ __ ’ __ ’ __ ’ __ ’ __ ’ __ ’ __
’ __ ’ __ ’ __ ’ __ ’ __ ’ __ ’ __ ’ __ ’ __ ’ __ ’ __ ’ __ ’ __ ’ __ ’ __ ’ __
’ __ ’ __ ’ __ ’ __ ’ __ ’ __ ’ __ ’ __ ’ __ ’ __ ’ __ ’ __ ’ __ ’ __ ’ __ ’ __
’ __ ’ __ ’ __ ’ __ ’ __ ’ __ ’ __ ’ __ ’ __ ’ __ ’ __ ’ __ ’ __ ’ __ ’ __ ’ __
’ __ ’ __ ’ __ ’ __ ’ __ ’ __ ’ __ ’ __ ’ __ ’ __ ’ __ ’ __ ’ __ ’ __ ’ __ ’ __
’ __ ’ __ ’ __ ’ __ ’ __ ’ __ ’ __ ’ __ ’ __ ’ __ ’ __ ’ __ ’ __ ’ __ ’ __ ’ __
’ __ ’ __ ’ __ ’ __ ’ __ ’ __ ’ __ ’ __ ’ __ ’ __ ’ __ ’ __ ’ __ ’ __ ’ __ ’ __
’ __ ’ __ ’ __ ’ __ ’ __ ’ __ ’ __ ’ __ ’ __ ’ __ ’ __ ’ __ ’ __ ’ __ ’ __ ’ __
’ __ ’ __ ’ __ ’ __ ’ __ ’ __ ’ __ ’ __ ’ __ ’ __ ’ __ ’ __ ’ __ ’ __ ’ __ ’ __
’ __ ’ __ ’ __ ’ __ ’ __ ’ __ ’ __ ’ __ ’ __ ’ __ ’ __ ’ __ ’ __ ’ __ ’ __ ’ __
’ __ ’ __ ’ __ ’ __ ’ __ ’ __ ’ __ ’ __ ’ __ ’ __ ’ __ ’ __ ’ __ ’ __ ’ __ ’ __
’ __ ’ __ ’ __ ’ __ ’ __ ’ __ ’ __ ’ __ ’ __ ’ __ ’ __ ’ __ ’ __ ’ __ ’ __ ’ __
’ __ ’ __ ’ __ ’ __ ’ __ ’ __ ’ __ ’ __ ’ __ ’ __ ’ __ ’ __ ’ __ ’ __ ’ __ ’ __
’ __ ’ __ ’ __ ’ __ ’ __ ’ __ ’ __ ’ __ ’ __ ’ __ ’ __ ’ __ ’ __ ’ __ ’ __ ’ __
’ __ ’ __ ’ __ ’ __ ’ __ ’ __ ’ __ ’ __ ’ __ ’ __ ’ __ ’ __ ’ __ ’ __ ’ __ ’ __
’ __ ’ __ ’ __ ’ __ ’ __ ’ __ ’ __ ’ __ ’ __ ’ __ ’ __ ’ __ ’ __ ’ __ ’ __ ’ __
’ __ ’ __ ’ __ ’ __ ’ __ ’ __ ’ __ ’ __ ’ __ ’ __ ’ __ ’ __ ’ __ ’ __ ’ __ ’ __
’ __ ’ __ ’ __ ’ __ ’ __ ’ __ ’ __ ’ __ ’ __ ’ __ ’ __ ’ __ ’ __ ’ __ ’ __ ’ __
’ __ ’ __ ’ __ ’ __ ’ __ ’ __ ’ __ ’ __ ’ __ ’ __ ’ __ ’ __ ’ __ ’ __ ’ __ ’ __
’ __ ’ __ ’ __ ’ __ ’ __ ’ __ ’ __ ’ __ ’ __ ’ __ ’ __ ’ __ ’ __ ’ __ ’ __ ’ __
’ __ ’ __ ’ __ ’ __ ’ __ ’ __ ’ __ ’ __ ’ __ ’ __ ’ __ ’ __ ’ __ ’ __ ’ __ ’ __
’ __ ’ __ ’ __ ’ __ ’ __ ’ __ ’ __ ’ __ ’ __ ’ __ ’ __ ’ __ ’ __ ’ __ ’ __ ’ __
’ __ ’ __ ’ __ ’ __ ’ __ ’ __ ’ __ ’ __ ’ __ ’ __ ’ __ ’ __ ’ __ ’ __ ’ __ ’ __
’ __ ’ __ ’ __ ’ __ ’ __ ’ __ ’ __ ’ __ ’ __ ’ __ ’ __ ’ __ ’ __ ’ __ ’ __ ’ __
’ __ ’ __ ’ __ ’ __ ’ __ ’ __ ’ __ ’ __ ’ __ ’ __ ’ __ ’ __ ’ __ ’ __ ’ __ ’ __
’ __ ’ __ ’ __ ’ __ ’ __ ’ __ ’ __ ’ __ ’ __ ’ __ ’ __ ’ __ ’ __ ’ __ ’ __ ’ __
’ __ ’ __ ’ __ ’ __ ’ __ ’ __ ’ __ ’ __ ’ __ ’ __ ’ __ ’ __ ’ __ ’ __ ’ __ ’ __
’ __ ’ __ ’ __ ’ __ ’ __ ’ __ ’ __ ’ __ ’ __ ’ __ ’ __ ’ __ ’ __ ’ __ ’ __ ’ __

’ __	’ __	’ __	’ __	’ __	’ __	’ __	’ __	’ __	’ __	’ __	’ __	’ __	’ __	’ __	’ __
’ __	’ __	’ __	’ __	’ __	’ __	’ __	’ __	’ __	’ __	’ __	’ __	’ __	’ __	’ __	’ __
’ __	’ __	’ __	’ __	’ __	’ __	’ __	’ __	’ __	’ __	’ __	’ __	’ __	’ __	’ __	’ __
’ __	’ __	’ __	’ __	’ __	’ __	’ __	’ __	’ __	’ __	’ __	’ __	’ __	’ __	’ __	’ __
’ __	’ __	’ __	’ __	’ __	’ __	’ __	’ __	’ __	’ __	’ __	’ __	’ __	’ __	’ __	’ __
’ __	’ __	’ __	’ __	’ __	’ __	’ __	’ __	’ __	’ __	’ __	’ __	’ __	’ __	’ __	’ __
’ __	’ __	’ __	’ __	’ __	’ __	’ __	’ __	’ __	’ __	’ __	’ __	’ __	’ __	’ __	’ __
’ __	’ __	’ __	’ __	’ __	’ __	’ __	’ __	’ __	’ __	’ __	’ __	’ __	’ __	’ __	’ __
’ __	’ __	’ __	’ __	’ __	’ __	’ __	’ __	’ __	’ __	’ __	’ __	’ __	’ __	’ __	’ __
’ __	’ __	’ __	’ __	’ __	’ __	’ __	’ __	’ __	’ __	’ __	’ __	’ __	’ __	’ __	’ __
’ __	’ __	’ __	’ __	’ __	’ __	’ __	’ __	’ __	’ __	’ __	’ __	’ __	’ __	’ __	’ __
’ __	’ __	’ __	’ __	’ __	’ __	’ __	’ __	’ __	’ __	’ __	’ __	’ __	’ __	’ __	’ __
’ __	’ __	’ __	’ __	’ __	’ __	’ __	’ __	’ __	’ __	’ __	’ __	’ __	’ __	’ __	’ __
’ __	’ __	’ __	’ __	’ __	’ __	’ __	’ __	’ __	’ __	’ __	’ __	’ __	’ __	’ __	’ __
’ __	’ __	’ __	’ __	’ __	’ __	’ __	’ __	’ __	’ __	’ __	’ __	’ __	’ __	’ __	’ __
’ __	’ __	’ __	’ __	’ __	’ __	’ __	’ __	’ __	’ __	’ __	’ __	’ __	’ __	’ __	’ __
’ __	’ __	’ __	’ __	’ __	’ __	’ __	’ __	’ __	’ __	’ __	’ __	’ __	’ __	’ __	’ __
’ __	’ __	’ __	’ __	’ __	’ __	’ __	’ __	’ __	’ __	’ __	’ __	’ __	’ __	’ __	’ __
’ __	’ __	’ __	’ __	’ __	’ __	’ __	’ __	’ __	’ __	’ __	’ __	’ __	’ __	’ __	’ __
’ __	’ __	’ __	’ __	’ __	’ __	’ __	’ __	’ __	’ __	’ __	’ __	’ __	’ __	’ __	’ __
’ __	’ __	’ __	’ __	’ __	’ __	’ __	’ __	’ __	’ __	’ __	’ __	’ __	’ __	’ __	’ __
’ __	’ __	’ __	’ __	’ __	’ __	’ __	’ __	’ __	’ __	’ __	’ __	’ __	’ __	’ __	’ __
’ __	’ __	’ __	’ __	’ __	’ __	’ __	’ __	’ __	’ __	’ __	’ __	’ __	’ __	’ __	’ __
’ __	’ __	’ __	’ __	’ __	’ __	’ __	’ __	’ __	’ __	’ __	’ __	’ __	’ __	’ __	’ __
’ __	’ __	’ __	’ __	’ __	’ __	’ __	’ __	’ __	’ __	’ __	’ __	’ __	’ __	’ __	’ __
’ __	’ __	’ __	’ __	’ __	’ __	’ __	’ __	’ __	’ __	’ __	’ __	’ __	’ __	’ __	’ __
’ __	’ __	’ __	’ __	’ __	’ __	’ __	’ __	’ __	’ __	’ __	’ __	’ __	’ __	’ __	’ __
’ __	’ __	’ __	’ __	’ __	’ __	’ __	’ __	’ __	’ __	’ __	’ __	’ __	’ __	’ __	’ __
’ __	’ __	’ __	’ __	’ __	’ __	’ __	’ __	’ __	’ __	’ __	’ __	’ __	’ __	’ __	’ __
’ __	’ __	’ __	’ __	’ __	’ __	’ __	’ __	’ __	’ __	’ __	’ __	’ __	’ __	’ __	’ __
’ __	’ __	’ __	’ __	’ __	’ __	’ __	’ __	’ __	’ __	’ __	’ __	’ __	’ __	’ __	’ __
’ __	’ __	’ __	’ __	’ __	’ __	’ __	’ __	’ __	’ __	’ __	’ __	’ __	’ __	’ __	’ __
’ __	’ __	’ __	’ __	’ __	’ __	’ __	’ __	’ __	’ __	’ __	’ __	’ __	’ __	’ __	’ __
’ __	’ __	’ __	’ __	’ __	’ __	’ __	’ __	’ __	’ __	’ __	’ __	’ __	’ __	’ __	’ __
’ __	’ __	’ __	’ __	’ __	’ __	’ __	’ __	’ __	’ __	’ __	’ __	’ __	’ __	’ __	’ __

’ __	’ __	’ __	’ __	’ __	’ __	’ __	’ __	’ __	’ __	’ __	’ __	’ __	’ __	’ __	’ __
’ __	’ __	’ __	’ __	’ __	’ __	’ __	’ __	’ __	’ __	’ __	’ __	’ __	’ __	’ __	’ __
’ __	’ __	’ __	’ __	’ __	’ __	’ __	’ __	’ __	’ __	’ __	’ __	’ __	’ __	’ __	’ __
’ __	’ __	’ __	’ __	’ __	’ __	’ __	’ __	’ __	’ __	’ __	’ __	’ __	’ __	’ __	’ __
’ __	’ __	’ __	’ __	’ __	’ __	’ __	’ __	’ __	’ __	’ __	’ __	’ __	’ __	’ __	’ __
’ __	’ __	’ __	’ __	’ __	’ __	’ __	’ __	’ __	’ __	’ __	’ __	’ __	’ __	’ __	’ __
’ __	’ __	’ __	’ __	’ __	’ __	’ __	’ __	’ __	’ __	’ __	’ __	’ __	’ __	’ __	’ __
’ __	’ __	’ __	’ __	’ __	’ __	’ __	’ __	’ __	’ __	’ __	’ __	’ __	’ __	’ __	’ __
’ __	’ __	’ __	’ __	’ __	’ __	’ __	’ __	’ __	’ __	’ __	’ __	’ __	’ __	’ __	’ __
’ __	’ __	’ __	’ __	’ __	’ __	’ __	’ __	’ __	’ __	’ __	’ __	’ __	’ __	’ __	’ __
’ __	’ __	’ __	’ __	’ __	’ __	’ __	’ __	’ __	’ __	’ __	’ __	’ __	’ __	’ __	’ __
’ __	’ __	’ __	’ __	’ __	’ __	’ __	’ __	’ __	’ __	’ __	’ __	’ __	’ __	’ __	’ __
’ __	’ __	’ __	’ __	’ __	’ __	’ __	’ __	’ __	’ __	’ __	’ __	’ __	’ __	’ __	’ __
’ __	’ __	’ __	’ __	’ __	’ __	’ __	’ __	’ __	’ __	’ __	’ __	’ __	’ __	’ __	’ __
’ __	’ __	’ __	’ __	’ __	’ __	’ __	’ __	’ __	’ __	’ __	’ __	’ __	’ __	’ __	’ __
’ __	’ __	’ __	’ __	’ __	’ __	’ __	’ __	’ __	’ __	’ __	’ __	’ __	’ __	’ __	’ __
’ __	’ __	’ __	’ __	’ __	’ __	’ __	’ __	’ __	’ __	’ __	’ __	’ __	’ __	’ __	’ __
’ __	’ __	’ __	’ __	’ __	’ __	’ __	’ __	’ __	’ __	’ __	’ __	’ __	’ __	’ __	’ __
’ __	’ __	’ __	’ __	’ __	’ __	’ __	’ __	’ __	’ __	’ __	’ __	’ __	’ __	’ __	’ __
’ __	’ __	’ __	’ __	’ __	’ __	’ __	’ __	’ __	’ __	’ __	’ __	’ __	’ __	’ __	’ __
’ __	’ __	’ __	’ __	’ __	’ __	’ __	’ __	’ __	’ __	’ __	’ __	’ __	’ __	’ __	’ __
’ __	’ __	’ __	’ __	’ __	’ __	’ __	’ __	’ __	’ __	’ __	’ __	’ __	’ __	’ __	’ __
’ __	’ __	’ __	’ __	’ __	’ __	’ __	’ __	’ __	’ __	’ __	’ __	’ __	’ __	’ __	’ __
’ __	’ __	’ __	’ __	’ __	’ __	’ __	’ __	’ __	’ __	’ __	’ __	’ __	’ __	’ __	’ __
’ __	’ __	’ __	’ __	’ __	’ __	’ __	’ __	’ __	’ __	’ __	’ __	’ __	’ __	’ __	’ __
’ __	’ __	’ __	’ __	’ __	’ __	’ __	’ __	’ __	’ __	’ __	’ __	’ __	’ __	’ __	’ __
’ __	’ __	’ __	’ __	’ __	’ __	’ __	’ __	’ __	’ __	’ __	’ __	’ __	’ __	’ __	’ __
’ __	’ __	’ __	’ __	’ __	’ __	’ __	’ __	’ __	’ __	’ __	’ __	’ __	’ __	’ __	’ __
’ __	’ __	’ __	’ __	’ __	’ __	’ __	’ __	’ __	’ __	’ __	’ __	’ __	’ __	’ __	’ __
’ __	’ __	’ __	’ __	’ __	’ __	’ __	’ __	’ __	’ __	’ __	’ __	’ __	’ __	’ __	’ __
’ __	’ __	’ __	’ __	’ __	’ __	’ __	’ __	’ __	’ __	’ __	’ __	’ __	’ __	’ __	’ __
’ __	’ __	’ __	’ __	’ __	’ __	’ __	’ __	’ __	’ __	’ __	’ __	’ __	’ __	’ __	’ __
’ __	’ __	’ __	’ __	’ __	’ __	’ __	’ __	’ __	’ __	’ __	’ __	’ __	’ __	’ __	’ __
’ __	’ __	’ __	’ __	’ __	’ __	’ __	’ __	’ __	’ __	’ __	’ __	’ __	’ __	’ __	’ __
’ __	’ __	’ __	’ __	’ __	’ __	’ __	’ __	’ __	’ __	’ __	’ __	’ __	’ __	’ __	’ __

, __	, __	, __	, __	, __	, __	, __	, __	, __	, __	, __	, __	, __	, __	, __	, __
, __	, __	, __	, __	, __	, __	, __	, __	, __	, __	, __	, __	, __	, __	, __	, __
, __	, __	, __	, __	, __	, __	, __	, __	, __	, __	, __	, __	, __	, __	, __	, __
, __	, __	, __	, __	, __	, __	, __	, __	, __	, __	, __	, __	, __	, __	, __	, __
, __	, __	, __	, __	, __	, __	, __	, __	, __	, __	, __	, __	, __	, __	, __	, __
, __	, __	, __	, __	, __	, __	, __	, __	, __	, __	, __	, __	, __	, __	, __	, __
, __	, __	, __	, __	, __	, __	, __	, __	, __	, __	, __	, __	, __	, __	, __	, __
, __	, __	, __	, __	, __	, __	, __	, __	, __	, __	, __	, __	, __	, __	, __	, __
, __	, __	, __	, __	, __	, __	, __	, __	, __	, __	, __	, __	, __	, __	, __	, __
, __	, __	, __	, __	, __	, __	, __	, __	, __	, __	, __	, __	, __	, __	, __	, __

goblet_ce	greatest_	gross_tum	icd_10_cc	igcccg_st	inpc_grac	inpc_hist	inrg_stag	inss_stag	internati	irs_group	irs_stage	ishak_fib	iss_stage	largest_e	last_know
'--	'--	'--	C02.1	'--	'--	'--	'--	'--	'--	'--	'--	'--	'--	'--	not repor
'--	'--	'--	C02.1	'--	'--	'--	'--	'--	'--	'--	'--	'--	'--	'--	not repor
'--	'--	'--	C02.9	'--	'--	'--	'--	'--	'--	'--	'--	'--	'--	'--	not repor
'--	'--	'--	C02.9	'--	'--	'--	'--	'--	'--	'--	'--	'--	'--	'--	not repor
'--	'--	'--	C02.9	'--	'--	'--	'--	'--	'--	'--	'--	'--	'--	'--	not repor
'--	'--	'--	C02.9	'--	'--	'--	'--	'--	'--	'--	'--	'--	'--	'--	not repor
'--	'--	'--	C02.9	'--	'--	'--	'--	'--	'--	'--	'--	'--	'--	'--	not repor
'--	'--	'--	C02.9	'--	'--	'--	'--	'--	'--	'--	'--	'--	'--	'--	not repor
'--	'--	'--	C02.9	'--	'--	'--	'--	'--	'--	'--	'--	'--	'--	'--	not repor
'--	'--	'--	C02.9	'--	'--	'--	'--	'--	'--	'--	'--	'--	'--	'--	not repor
'--	'--	'--	C02.9	'--	'--	'--	'--	'--	'--	'--	'--	'--	'--	'--	not repor
'--	'--	'--	C02.9	'--	'--	'--	'--	'--	'--	'--	'--	'--	'--	'--	not repor
'--	'--	'--	C02.9	'--	'--	'--	'--	'--	'--	'--	'--	'--	'--	'--	not repor
'--	'--	'--	C02.9	'--	'--	'--	'--	'--	'--	'--	'--	'--	'--	'--	not repor
'--	'--	'--	C02.9	'--	'--	'--	'--	'--	'--	'--	'--	'--	'--	'--	not repor
'--	'--	'--	C02.9	'--	'--	'--	'--	'--	'--	'--	'--	'--	'--	'--	not repor
'--	'--	'--	C02.9	'--	'--	'--	'--	'--	'--	'--	'--	'--	'--	'--	not repor
'--	'--	'--	C02.9	'--	'--	'--	'--	'--	'--	'--	'--	'--	'--	'--	not repor
'--	'--	'--	C02.9	'--	'--	'--	'--	'--	'--	'--	'--	'--	'--	'--	not repor
'--	'--	'--	C02.9	'--	'--	'--	'--	'--	'--	'--	'--	'--	'--	'--	not repor
'--	'--	'--	C02.9	'--	'--	'--	'--	'--	'--	'--	'--	'--	'--	'--	not repor
'--	'--	'--	C02.9	'--	'--	'--	'--	'--	'--	'--	'--	'--	'--	'--	not repor
'--	'--	'--	C02.9	'--	'--	'--	'--	'--	'--	'--	'--	'--	'--	'--	not repor
'--	'--	'--	C02.9	'--	'--	'--	'--	'--	'--	'--	'--	'--	'--	'--	not repor
'--	'--	'--	C02.9	'--	'--	'--	'--	'--	'--	'--	'--	'--	'--	'--	not repor
'--	'--	'--	C02.9	'--	'--	'--	'--	'--	'--	'--	'--	'--	'--	'--	not repor
'--	'--	'--	C02.9	'--	'--	'--	'--	'--	'--	'--	'--	'--	'--	'--	not repor
'--	'--	'--	C02.9	'--	'--	'--	'--	'--	'--	'--	'--	'--	'--	'--	not repor
'--	'--	'--	C02.9	'--	'--	'--	'--	'--	'--	'--	'--	'--	'--	'--	not repor
'--	'--	'--	C02.9	'--	'--	'--	'--	'--	'--	'--	'--	'--	'--	'--	not repor
'--	'--	'--	C02.9	'--	'--	'--	'--	'--	'--	'--	'--	'--	'--	'--	not repor
'--	'--	'--	C02.9	'--	'--	'--	'--	'--	'--	'--	'--	'--	'--	'--	not repor
'--	'--	'--	C02.9	'--	'--	'--	'--	'--	'--	'--	'--	'--	'--	'--	not repor
'--	'--	'--	C02.9	'--	'--	'--	'--	'--	'--	'--	'--	'--	'--	'--	not repor

'--	'--	'--	C02.9	'--	'--	'--	'--	'--	'--	'--	'--	'--	'--	'--	not repor
'--	'--	'--	C02.9	'--	'--	'--	'--	'--	'--	'--	'--	'--	'--	'--	not repor
'--	'--	'--	C02.9	'--	'--	'--	'--	'--	'--	'--	'--	'--	'--	'--	not repor
'--	'--	'--	C02.9	'--	'--	'--	'--	'--	'--	'--	'--	'--	'--	'--	not repor
'--	'--	'--	C02.9	'--	'--	'--	'--	'--	'--	'--	'--	'--	'--	'--	not repor
'--	'--	'--	C02.9	'--	'--	'--	'--	'--	'--	'--	'--	'--	'--	'--	not repor
'--	'--	'--	C02.9	'--	'--	'--	'--	'--	'--	'--	'--	'--	'--	'--	not repor
'--	'--	'--	C02.9	'--	'--	'--	'--	'--	'--	'--	'--	'--	'--	'--	not repor
'--	'--	'--	C02.9	'--	'--	'--	'--	'--	'--	'--	'--	'--	'--	'--	not repor
'--	'--	'--	C02.9	'--	'--	'--	'--	'--	'--	'--	'--	'--	'--	'--	not repor
'--	'--	'--	C02.9	'--	'--	'--	'--	'--	'--	'--	'--	'--	'--	'--	not repor
'--	'--	'--	C02.9	'--	'--	'--	'--	'--	'--	'--	'--	'--	'--	'--	not repor
'--	'--	'--	C02.9	'--	'--	'--	'--	'--	'--	'--	'--	'--	'--	'--	not repor
'--	'--	'--	C02.9	'--	'--	'--	'--	'--	'--	'--	'--	'--	'--	'--	not repor
'--	'--	'--	C02.9	'--	'--	'--	'--	'--	'--	'--	'--	'--	'--	'--	not repor
'--	'--	'--	C02.9	'--	'--	'--	'--	'--	'--	'--	'--	'--	'--	'--	not repor
'--	'--	'--	C02.9	'--	'--	'--	'--	'--	'--	'--	'--	'--	'--	'--	not repor
'--	'--	'--	C02.9	'--	'--	'--	'--	'--	'--	'--	'--	'--	'--	'--	not repor
'--	'--	'--	C02.9	'--	'--	'--	'--	'--	'--	'--	'--	'--	'--	'--	not repor
'--	'--	'--	C02.9	'--	'--	'--	'--	'--	'--	'--	'--	'--	'--	'--	not repor
'--	'--	'--	C02.9	'--	'--	'--	'--	'--	'--	'--	'--	'--	'--	'--	not repor
'--	'--	'--	C02.9	'--	'--	'--	'--	'--	'--	'--	'--	'--	'--	'--	not repor
'--	'--	'--	C02.9	'--	'--	'--	'--	'--	'--	'--	'--	'--	'--	'--	not repor
'--	'--	'--	C02.9	'--	'--	'--	'--	'--	'--	'--	'--	'--	'--	'--	not repor
'--	'--	'--	C02.9	'--	'--	'--	'--	'--	'--	'--	'--	'--	'--	'--	not repor
'--	'--	'--	C02.9	'--	'--	'--	'--	'--	'--	'--	'--	'--	'--	'--	not repor
'--	'--	'--	C02.9	'--	'--	'--	'--	'--	'--	'--	'--	'--	'--	'--	not repor
'--	'--	'--	C02.9	'--	'--	'--	'--	'--	'--	'--	'--	'--	'--	'--	not repor
'--	'--	'--	C02.9	'--	'--	'--	'--	'--	'--	'--	'--	'--	'--	'--	not repor
'--	'--	'--	C02.9	'--	'--	'--	'--	'--	'--	'--	'--	'--	'--	'--	not repor
'--	'--	'--	C02.9	'--	'--	'--	'--	'--	'--	'--	'--	'--	'--	'--	not repor
'--	'--	'--	C02.9	'--	'--	'--	'--	'--	'--	'--	'--	'--	'--	'--	not repor
'--	'--	'--	C02.9	'--	'--	'--	'--	'--	'--	'--	'--	'--	'--	'--	not repor
'--	'--	'--	C02.9	'--	'--	'--	'--	'--	'--	'--	'--	'--	'--	'--	not repor
'--	'--	'--	C02.9	'--	'--	'--	'--	'--	'--	'--	'--	'--	'--	'--	not repor

'--	'--	'--	C02.9	'--	'--	'--	'--	'--	'--	'--	'--	'--	'--	'--	not repor
'--	'--	'--	C02.9	'--	'--	'--	'--	'--	'--	'--	'--	'--	'--	'--	not repor
'--	'--	'--	C02.9	'--	'--	'--	'--	'--	'--	'--	'--	'--	'--	'--	not repor
'--	'--	'--	C02.9	'--	'--	'--	'--	'--	'--	'--	'--	'--	'--	'--	not repor
'--	'--	'--	C02.9	'--	'--	'--	'--	'--	'--	'--	'--	'--	'--	'--	not repor
'--	'--	'--	C02.9	'--	'--	'--	'--	'--	'--	'--	'--	'--	'--	'--	not repor
'--	'--	'--	C02.9	'--	'--	'--	'--	'--	'--	'--	'--	'--	'--	'--	not repor
'--	'--	'--	C02.9	'--	'--	'--	'--	'--	'--	'--	'--	'--	'--	'--	not repor
'--	'--	'--	C02.9	'--	'--	'--	'--	'--	'--	'--	'--	'--	'--	'--	not repor
'--	'--	'--	C02.9	'--	'--	'--	'--	'--	'--	'--	'--	'--	'--	'--	not repor
'--	'--	'--	C02.9	'--	'--	'--	'--	'--	'--	'--	'--	'--	'--	'--	not repor
'--	'--	'--	C02.9	'--	'--	'--	'--	'--	'--	'--	'--	'--	'--	'--	not repor
'--	'--	'--	C02.9	'--	'--	'--	'--	'--	'--	'--	'--	'--	'--	'--	not repor
'--	'--	'--	C02.9	'--	'--	'--	'--	'--	'--	'--	'--	'--	'--	'--	not repor
'--	'--	'--	C02.9	'--	'--	'--	'--	'--	'--	'--	'--	'--	'--	'--	not repor
'--	'--	'--	C02.9	'--	'--	'--	'--	'--	'--	'--	'--	'--	'--	'--	not repor
'--	'--	'--	C02.9	'--	'--	'--	'--	'--	'--	'--	'--	'--	'--	'--	not repor
'--	'--	'--	C02.9	'--	'--	'--	'--	'--	'--	'--	'--	'--	'--	'--	not repor
'--	'--	'--	C02.9	'--	'--	'--	'--	'--	'--	'--	'--	'--	'--	'--	not repor
'--	'--	'--	C02.9	'--	'--	'--	'--	'--	'--	'--	'--	'--	'--	'--	not repor
'--	'--	'--	C02.9	'--	'--	'--	'--	'--	'--	'--	'--	'--	'--	'--	not repor
'--	'--	'--	C02.9	'--	'--	'--	'--	'--	'--	'--	'--	'--	'--	'--	not repor
'--	'--	'--	C02.9	'--	'--	'--	'--	'--	'--	'--	'--	'--	'--	'--	not repor
'--	'--	'--	C02.9	'--	'--	'--	'--	'--	'--	'--	'--	'--	'--	'--	not repor
'--	'--	'--	C02.9	'--	'--	'--	'--	'--	'--	'--	'--	'--	'--	'--	not repor
'--	'--	'--	C02.9	'--	'--	'--	'--	'--	'--	'--	'--	'--	'--	'--	not repor
'--	'--	'--	C02.9	'--	'--	'--	'--	'--	'--	'--	'--	'--	'--	'--	not repor
'--	'--	'--	C02.9	'--	'--	'--	'--	'--	'--	'--	'--	'--	'--	'--	not repor
'--	'--	'--	C02.9	'--	'--	'--	'--	'--	'--	'--	'--	'--	'--	'--	not repor
'--	'--	'--	C02.9	'--	'--	'--	'--	'--	'--	'--	'--	'--	'--	'--	not repor
'--	'--	'--	C02.9	'--	'--	'--	'--	'--	'--	'--	'--	'--	'--	'--	not repor
'--	'--	'--	C02.9	'--	'--	'--	'--	'--	'--	'--	'--	'--	'--	'--	not repor
'--	'--	'--	C02.9	'--	'--	'--	'--	'--	'--	'--	'--	'--	'--	'--	not repor
'--	'--	'--	C02.9	'--	'--	'--	'--	'--	'--	'--	'--	'--	'--	'--	not repor
'--	'--	'--	C02.9	'--	'--	'--	'--	'--	'--	'--	'--	'--	'--	'--	not repor

'--	'--	'--	C02.9	'--	'--	'--	'--	'--	'--	'--	'--	'--	'--	'--	not repor
'--	'--	'--	C02.9	'--	'--	'--	'--	'--	'--	'--	'--	'--	'--	'--	not repor
'--	'--	'--	C02.9	'--	'--	'--	'--	'--	'--	'--	'--	'--	'--	'--	not repor
'--	'--	'--	C02.9	'--	'--	'--	'--	'--	'--	'--	'--	'--	'--	'--	not repor
'--	'--	'--	C02.9	'--	'--	'--	'--	'--	'--	'--	'--	'--	'--	'--	not repor
'--	'--	'--	C02.9	'--	'--	'--	'--	'--	'--	'--	'--	'--	'--	'--	not repor
'--	'--	'--	C02.9	'--	'--	'--	'--	'--	'--	'--	'--	'--	'--	'--	not repor
'--	'--	'--	C02.9	'--	'--	'--	'--	'--	'--	'--	'--	'--	'--	'--	not repor
'--	'--	'--	C02.2	'--	'--	'--	'--	'--	'--	'--	'--	'--	'--	'--	not repor
'--	'--	'--	C02.2	'--	'--	'--	'--	'--	'--	'--	'--	'--	'--	'--	not repor
'--	'--	'--	C02.9	'--	'--	'--	'--	'--	'--	'--	'--	'--	'--	'--	not repor
'--	'--	'--	C02.9	'--	'--	'--	'--	'--	'--	'--	'--	'--	'--	'--	not repor
'--	'--	'--	C02.9	'--	'--	'--	'--	'--	'--	'--	'--	'--	'--	'--	not repor
'--	'--	'--	C02.9	'--	'--	'--	'--	'--	'--	'--	'--	'--	'--	'--	not repor
'--	'--	'--	C02.9	'--	'--	'--	'--	'--	'--	'--	'--	'--	'--	'--	not repor
'--	'--	'--	C02.9	'--	'--	'--	'--	'--	'--	'--	'--	'--	'--	'--	not repor
'--	'--	'--	C02.9	'--	'--	'--	'--	'--	'--	'--	'--	'--	'--	'--	not repor
'--	'--	'--	C02.9	'--	'--	'--	'--	'--	'--	'--	'--	'--	'--	'--	not repor
'--	'--	'--	C02.9	'--	'--	'--	'--	'--	'--	'--	'--	'--	'--	'--	not repor
'--	'--	'--	C02.9	'--	'--	'--	'--	'--	'--	'--	'--	'--	'--	'--	not repor
'--	'--	'--	C02.9	'--	'--	'--	'--	'--	'--	'--	'--	'--	'--	'--	not repor
'--	'--	'--	C02.9	'--	'--	'--	'--	'--	'--	'--	'--	'--	'--	'--	not repor
'--	'--	'--	C02.9	'--	'--	'--	'--	'--	'--	'--	'--	'--	'--	'--	not repor
'--	'--	'--	C02.9	'--	'--	'--	'--	'--	'--	'--	'--	'--	'--	'--	not repor
'--	'--	'--	C02.9	'--	'--	'--	'--	'--	'--	'--	'--	'--	'--	'--	not repor
'--	'--	'--	C02.9	'--	'--	'--	'--	'--	'--	'--	'--	'--	'--	'--	not repor
'--	'--	'--	C02.9	'--	'--	'--	'--	'--	'--	'--	'--	'--	'--	'--	not repor
'--	'--	'--	C02.9	'--	'--	'--	'--	'--	'--	'--	'--	'--	'--	'--	not repor
'--	'--	'--	C02.9	'--	'--	'--	'--	'--	'--	'--	'--	'--	'--	'--	not repor
'--	'--	'--	C02.9	'--	'--	'--	'--	'--	'--	'--	'--	'--	'--	'--	not repor
'--	'--	'--	C02.9	'--	'--	'--	'--	'--	'--	'--	'--	'--	'--	'--	not repor
'--	'--	'--	C02.9	'--	'--	'--	'--	'--	'--	'--	'--	'--	'--	'--	not repor
'--	'--	'--	C02.9	'--	'--	'--	'--	'--	'--	'--	'--	'--	'--	'--	not repor
'--	'--	'--	C02.9	'--	'--	'--	'--	'--	'--	'--	'--	'--	'--	'--	not repor
'--	'--	'--	C02.9	'--	'--	'--	'--	'--	'--	'--	'--	'--	'--	'--	not repor

'--	'--	'--	C02.9	'--	'--	'--	'--	'--	'--	'--	'--	'--	'--	'--	not repor
'--	'--	'--	C02.9	'--	'--	'--	'--	'--	'--	'--	'--	'--	'--	'--	not repor
'--	'--	'--	C02.9	'--	'--	'--	'--	'--	'--	'--	'--	'--	'--	'--	not repor
'--	'--	'--	C02.9	'--	'--	'--	'--	'--	'--	'--	'--	'--	'--	'--	not repor
'--	'--	'--	C02.9	'--	'--	'--	'--	'--	'--	'--	'--	'--	'--	'--	not repor
'--	'--	'--	C02.9	'--	'--	'--	'--	'--	'--	'--	'--	'--	'--	'--	not repor
'--	'--	'--	C02.9	'--	'--	'--	'--	'--	'--	'--	'--	'--	'--	'--	not repor
'--	'--	'--	C02.9	'--	'--	'--	'--	'--	'--	'--	'--	'--	'--	'--	not repor
'--	'--	'--	C02.9	'--	'--	'--	'--	'--	'--	'--	'--	'--	'--	'--	not repor
'--	'--	'--	C02.9	'--	'--	'--	'--	'--	'--	'--	'--	'--	'--	'--	not repor
'--	'--	'--	C02.9	'--	'--	'--	'--	'--	'--	'--	'--	'--	'--	'--	not repor
'--	'--	'--	C02.9	'--	'--	'--	'--	'--	'--	'--	'--	'--	'--	'--	not repor
'--	'--	'--	C02.9	'--	'--	'--	'--	'--	'--	'--	'--	'--	'--	'--	not repor
'--	'--	'--	C02.9	'--	'--	'--	'--	'--	'--	'--	'--	'--	'--	'--	not repor
'--	'--	'--	C02.9	'--	'--	'--	'--	'--	'--	'--	'--	'--	'--	'--	not repor
'--	'--	'--	C02.9	'--	'--	'--	'--	'--	'--	'--	'--	'--	'--	'--	not repor
'--	'--	'--	C02.9	'--	'--	'--	'--	'--	'--	'--	'--	'--	'--	'--	not repor
'--	'--	'--	C02.9	'--	'--	'--	'--	'--	'--	'--	'--	'--	'--	'--	not repor
'--	'--	'--	C02.9	'--	'--	'--	'--	'--	'--	'--	'--	'--	'--	'--	not repor
'--	'--	'--	C02.9	'--	'--	'--	'--	'--	'--	'--	'--	'--	'--	'--	not repor
'--	'--	'--	C02.9	'--	'--	'--	'--	'--	'--	'--	'--	'--	'--	'--	not repor
'--	'--	'--	C02.9	'--	'--	'--	'--	'--	'--	'--	'--	'--	'--	'--	not repor
'--	'--	'--	C02.9	'--	'--	'--	'--	'--	'--	'--	'--	'--	'--	'--	not repor
'--	'--	'--	C02.9	'--	'--	'--	'--	'--	'--	'--	'--	'--	'--	'--	not repor
'--	'--	'--	C02.9	'--	'--	'--	'--	'--	'--	'--	'--	'--	'--	'--	not repor
'--	'--	'--	C02.9	'--	'--	'--	'--	'--	'--	'--	'--	'--	'--	'--	not repor
'--	'--	'--	C02.9	'--	'--	'--	'--	'--	'--	'--	'--	'--	'--	'--	not repor
'--	'--	'--	C02.9	'--	'--	'--	'--	'--	'--	'--	'--	'--	'--	'--	not repor
'--	'--	'--	C02.9	'--	'--	'--	'--	'--	'--	'--	'--	'--	'--	'--	not repor
'--	'--	'--	C02.9	'--	'--	'--	'--	'--	'--	'--	'--	'--	'--	'--	not repor
'--	'--	'--	C02.9	'--	'--	'--	'--	'--	'--	'--	'--	'--	'--	'--	not repor
'--	'--	'--	C02.9	'--	'--	'--	'--	'--	'--	'--	'--	'--	'--	'--	not repor
'--	'--	'--	C02.9	'--	'--	'--	'--	'--	'--	'--	'--	'--	'--	'--	not repor
'--	'--	'--	C02.9	'--	'--	'--	'--	'--	'--	'--	'--	'--	'--	'--	not repor
'--	'--	'--	C02.9	'--	'--	'--	'--	'--	'--	'--	'--	'--	'--	'--	not repor

'--	'--	'--	C02.9	'--	'--	'--	'--	'--	'--	'--	'--	'--	'--	'--	not repor
'--	'--	'--	C02.9	'--	'--	'--	'--	'--	'--	'--	'--	'--	'--	'--	not repor
'--	'--	'--	C02.9	'--	'--	'--	'--	'--	'--	'--	'--	'--	'--	'--	not repor
'--	'--	'--	C02.9	'--	'--	'--	'--	'--	'--	'--	'--	'--	'--	'--	not repor
'--	'--	'--	C02.9	'--	'--	'--	'--	'--	'--	'--	'--	'--	'--	'--	not repor
'--	'--	'--	C02.9	'--	'--	'--	'--	'--	'--	'--	'--	'--	'--	'--	not repor
'--	'--	'--	C02.9	'--	'--	'--	'--	'--	'--	'--	'--	'--	'--	'--	not repor
'--	'--	'--	C02.9	'--	'--	'--	'--	'--	'--	'--	'--	'--	'--	'--	not repor
'--	'--	'--	C02.9	'--	'--	'--	'--	'--	'--	'--	'--	'--	'--	'--	not repor
'--	'--	'--	C02.9	'--	'--	'--	'--	'--	'--	'--	'--	'--	'--	'--	not repor
'--	'--	'--	C02.9	'--	'--	'--	'--	'--	'--	'--	'--	'--	'--	'--	not repor
'--	'--	'--	C02.9	'--	'--	'--	'--	'--	'--	'--	'--	'--	'--	'--	not repor
'--	'--	'--	C02.9	'--	'--	'--	'--	'--	'--	'--	'--	'--	'--	'--	not repor
'--	'--	'--	C02.9	'--	'--	'--	'--	'--	'--	'--	'--	'--	'--	'--	not repor
'--	'--	'--	C02.9	'--	'--	'--	'--	'--	'--	'--	'--	'--	'--	'--	not repor
'--	'--	'--	C02.9	'--	'--	'--	'--	'--	'--	'--	'--	'--	'--	'--	not repor
'--	'--	'--	C02.9	'--	'--	'--	'--	'--	'--	'--	'--	'--	'--	'--	not repor
'--	'--	'--	C02.9	'--	'--	'--	'--	'--	'--	'--	'--	'--	'--	'--	not repor
'--	'--	'--	C02.9	'--	'--	'--	'--	'--	'--	'--	'--	'--	'--	'--	not repor
'--	'--	'--	C02.9	'--	'--	'--	'--	'--	'--	'--	'--	'--	'--	'--	not repor
'--	'--	'--	C02.9	'--	'--	'--	'--	'--	'--	'--	'--	'--	'--	'--	not repor
'--	'--	'--	C02.9	'--	'--	'--	'--	'--	'--	'--	'--	'--	'--	'--	not repor
'--	'--	'--	C02.9	'--	'--	'--	'--	'--	'--	'--	'--	'--	'--	'--	not repor
'--	'--	'--	C02.9	'--	'--	'--	'--	'--	'--	'--	'--	'--	'--	'--	not repor
'--	'--	'--	C02.9	'--	'--	'--	'--	'--	'--	'--	'--	'--	'--	'--	not repor
'--	'--	'--	C02.9	'--	'--	'--	'--	'--	'--	'--	'--	'--	'--	'--	not repor
'--	'--	'--	C02.9	'--	'--	'--	'--	'--	'--	'--	'--	'--	'--	'--	not repor
'--	'--	'--	C02.9	'--	'--	'--	'--	'--	'--	'--	'--	'--	'--	'--	not repor
'--	'--	'--	C02.9	'--	'--	'--	'--	'--	'--	'--	'--	'--	'--	'--	not repor
'--	'--	'--	C02.9	'--	'--	'--	'--	'--	'--	'--	'--	'--	'--	'--	not repor
'--	'--	'--	C02.9	'--	'--	'--	'--	'--	'--	'--	'--	'--	'--	'--	not repor
'--	'--	'--	C02.9	'--	'--	'--	'--	'--	'--	'--	'--	'--	'--	'--	not repor
'--	'--	'--	C02.9	'--	'--	'--	'--	'--	'--	'--	'--	'--	'--	'--	not repor
'--	'--	'--	C02.9	'--	'--	'--	'--	'--	'--	'--	'--	'--	'--	'--	not repor
'--	'--	'--	C02.9	'--	'--	'--	'--	'--	'--	'--	'--	'--	'--	'--	not repor

'--	'--	'--	C02.9	'--	'--	'--	'--	'--	'--	'--	'--	'--	'--	'--	not repor
'--	'--	'--	C02.9	'--	'--	'--	'--	'--	'--	'--	'--	'--	'--	'--	not repor
'--	'--	'--	C02.9	'--	'--	'--	'--	'--	'--	'--	'--	'--	'--	'--	not repor
'--	'--	'--	C02.9	'--	'--	'--	'--	'--	'--	'--	'--	'--	'--	'--	not repor
'--	'--	'--	C02.9	'--	'--	'--	'--	'--	'--	'--	'--	'--	'--	'--	not repor
'--	'--	'--	C02.9	'--	'--	'--	'--	'--	'--	'--	'--	'--	'--	'--	not repor
'--	'--	'--	C02.9	'--	'--	'--	'--	'--	'--	'--	'--	'--	'--	'--	not repor
'--	'--	'--	C02.9	'--	'--	'--	'--	'--	'--	'--	'--	'--	'--	'--	not repor
'--	'--	'--	C02.9	'--	'--	'--	'--	'--	'--	'--	'--	'--	'--	'--	not repor
'--	'--	'--	C02.9	'--	'--	'--	'--	'--	'--	'--	'--	'--	'--	'--	not repor
'--	'--	'--	C02.9	'--	'--	'--	'--	'--	'--	'--	'--	'--	'--	'--	not repor
'--	'--	'--	C02.9	'--	'--	'--	'--	'--	'--	'--	'--	'--	'--	'--	not repor
'--	'--	'--	C02.9	'--	'--	'--	'--	'--	'--	'--	'--	'--	'--	'--	not repor
'--	'--	'--	C02.9	'--	'--	'--	'--	'--	'--	'--	'--	'--	'--	'--	not repor
'--	'--	'--	C02.9	'--	'--	'--	'--	'--	'--	'--	'--	'--	'--	'--	not repor
'--	'--	'--	C02.9	'--	'--	'--	'--	'--	'--	'--	'--	'--	'--	'--	not repor
'--	'--	'--	C02.9	'--	'--	'--	'--	'--	'--	'--	'--	'--	'--	'--	not repor
'--	'--	'--	C02.9	'--	'--	'--	'--	'--	'--	'--	'--	'--	'--	'--	not repor
'--	'--	'--	C02.9	'--	'--	'--	'--	'--	'--	'--	'--	'--	'--	'--	not repor
'--	'--	'--	C02.9	'--	'--	'--	'--	'--	'--	'--	'--	'--	'--	'--	not repor
'--	'--	'--	C02.9	'--	'--	'--	'--	'--	'--	'--	'--	'--	'--	'--	not repor
'--	'--	'--	C02.9	'--	'--	'--	'--	'--	'--	'--	'--	'--	'--	'--	not repor
'--	'--	'--	C02.9	'--	'--	'--	'--	'--	'--	'--	'--	'--	'--	'--	not repor
'--	'--	'--	C02.9	'--	'--	'--	'--	'--	'--	'--	'--	'--	'--	'--	not repor
'--	'--	'--	C02.9	'--	'--	'--	'--	'--	'--	'--	'--	'--	'--	'--	not repor
'--	'--	'--	C02.9	'--	'--	'--	'--	'--	'--	'--	'--	'--	'--	'--	not repor
'--	'--	'--	C02.9	'--	'--	'--	'--	'--	'--	'--	'--	'--	'--	'--	not repor
'--	'--	'--	C02.9	'--	'--	'--	'--	'--	'--	'--	'--	'--	'--	'--	not repor
'--	'--	'--	C02.9	'--	'--	'--	'--	'--	'--	'--	'--	'--	'--	'--	not repor
'--	'--	'--	C02.9	'--	'--	'--	'--	'--	'--	'--	'--	'--	'--	'--	not repor
'--	'--	'--	C02.9	'--	'--	'--	'--	'--	'--	'--	'--	'--	'--	'--	not repor
'--	'--	'--	C02.9	'--	'--	'--	'--	'--	'--	'--	'--	'--	'--	'--	not repor
'--	'--	'--	C02.9	'--	'--	'--	'--	'--	'--	'--	'--	'--	'--	'--	not repor
'--	'--	'--	C02.9	'--	'--	'--	'--	'--	'--	'--	'--	'--	'--	'--	not repor
'--	'--	'--	C02.9	'--	'--	'--	'--	'--	'--	'--	'--	'--	'--	'--	not repor

'--	'--	'--	C02.9	'--	'--	'--	'--	'--	'--	'--	'--	'--	'--	'--	not repor
'--	'--	'--	C02.9	'--	'--	'--	'--	'--	'--	'--	'--	'--	'--	'--	not repor
'--	'--	'--	C02.9	'--	'--	'--	'--	'--	'--	'--	'--	'--	'--	'--	not repor
'--	'--	'--	C02.9	'--	'--	'--	'--	'--	'--	'--	'--	'--	'--	'--	not repor
'--	'--	'--	C02.9	'--	'--	'--	'--	'--	'--	'--	'--	'--	'--	'--	not repor
'--	'--	'--	C02.9	'--	'--	'--	'--	'--	'--	'--	'--	'--	'--	'--	not repor
'--	'--	'--	C02.9	'--	'--	'--	'--	'--	'--	'--	'--	'--	'--	'--	not repor
'--	'--	'--	C02.9	'--	'--	'--	'--	'--	'--	'--	'--	'--	'--	'--	not repor
'--	'--	'--	C02.9	'--	'--	'--	'--	'--	'--	'--	'--	'--	'--	'--	not repor
'--	'--	'--	C02.9	'--	'--	'--	'--	'--	'--	'--	'--	'--	'--	'--	not repor

lateralit	lymph_noc	lymph_noc	lymph_noc	lymphatic	margin_di	margins_i	masaoka_s	medullobl	metastasi	metastasi	method_of	micropapi	mitosis_k	mitotic_c	morpholog
'--	'--	'--	'--	'--	'--	'--	'--	'--	'--	'--	'--	'--	'--	'--	Mar-70
'--	'--	'--	'--	'--	'--	'--	'--	'--	'--	'--	'--	'--	'--	'--	Mar-70
'--	'--	'--	'--	'--	'--	'--	'--	'--	'--	'--	'--	'--	'--	'--	Mar-70
'--	'--	'--	'--	'--	'--	'--	'--	'--	'--	'--	'--	'--	'--	'--	Mar-70
'--	'--	'--	'--	'--	'--	'--	'--	'--	'--	'--	'--	'--	'--	'--	Mar-70
'--	'--	'--	'--	'--	'--	'--	'--	'--	'--	'--	'--	'--	'--	'--	Mar-70
'--	'--	'--	'--	'--	'--	'--	'--	'--	'--	'--	'--	'--	'--	'--	Mar-70
'--	'--	'--	'--	'--	'--	'--	'--	'--	'--	'--	'--	'--	'--	'--	Mar-70
'--	'--	'--	'--	'--	'--	'--	'--	'--	'--	'--	'--	'--	'--	'--	Mar-71
'--	'--	'--	'--	'--	'--	'--	'--	'--	'--	'--	'--	'--	'--	'--	Mar-71
'--	'--	'--	'--	'--	'--	'--	'--	'--	'--	'--	'--	'--	'--	'--	Mar-70
'--	'--	'--	'--	'--	'--	'--	'--	'--	'--	'--	'--	'--	'--	'--	Mar-70
'--	'--	'--	'--	'--	'--	'--	'--	'--	'--	'--	'--	'--	'--	'--	Mar-70
'--	'--	'--	'--	'--	'--	'--	'--	'--	'--	'--	'--	'--	'--	'--	Mar-70
'--	'--	'--	'--	'--	'--	'--	'--	'--	'--	'--	'--	'--	'--	'--	Mar-70
'--	'--	'--	'--	'--	'--	'--	'--	'--	'--	'--	'--	'--	'--	'--	Mar-70
'--	'--	'--	'--	'--	'--	'--	'--	'--	'--	'--	'--	'--	'--	'--	Mar-70
'--	'--	'--	'--	'--	'--	'--	'--	'--	'--	'--	'--	'--	'--	'--	Mar-70
'--	'--	'--	'--	'--	'--	'--	'--	'--	'--	'--	'--	'--	'--	'--	Mar-70
'--	'--	'--	'--	'--	'--	'--	'--	'--	'--	'--	'--	'--	'--	'--	Mar-70
'--	'--	'--	'--	'--	'--	'--	'--	'--	'--	'--	'--	'--	'--	'--	Mar-70
'--	'--	'--	'--	'--	'--	'--	'--	'--	'--	'--	'--	'--	'--	'--	Mar-70
'--	'--	'--	'--	'--	'--	'--	'--	'--	'--	'--	'--	'--	'--	'--	Mar-70
'--	'--	'--	'--	'--	'--	'--	'--	'--	'--	'--	'--	'--	'--	'--	Mar-70
'--	'--	'--	'--	'--	'--	'--	'--	'--	'--	'--	'--	'--	'--	'--	Mar-71
'--	'--	'--	'--	'--	'--	'--	'--	'--	'--	'--	'--	'--	'--	'--	Mar-71
'--	'--	'--	'--	'--	'--	'--	'--	'--	'--	'--	'--	'--	'--	'--	Mar-71
'--	'--	'--	'--	'--	'--	'--	'--	'--	'--	'--	'--	'--	'--	'--	Mar-71
'--	'--	'--	'--	'--	'--	'--	'--	'--	'--	'--	'--	'--	'--	'--	Mar-71
'--	'--	'--	'--	'--	'--	'--	'--	'--	'--	'--	'--	'--	'--	'--	Mar-71
'--	'--	'--	'--	'--	'--	'--	'--	'--	'--	'--	'--	'--	'--	'--	Mar-71
'--	'--	'--	'--	'--	'--	'--	'--	'--	'--	'--	'--	'--	'--	'--	Mar-71
'--	'--	'--	'--	'--	'--	'--	'--	'--	'--	'--	'--	'--	'--	'--	Mar-70
'--	'--	'--	'--	'--	'--	'--	'--	'--	'--	'--	'--	'--	'--	'--	Mar-70

’__	’__	’__	’__	’__	’__	’__	’__	’__	’__	’__	’__	’__	’__	’__	Mar-70
’__	’__	’__	’__	’__	’__	’__	’__	’__	’__	’__	’__	’__	’__	’__	Mar-70
’__	’__	’__	’__	’__	’__	’__	’__	’__	’__	’__	’__	’__	’__	’__	Mar-71
’__	’__	’__	’__	’__	’__	’__	’__	’__	’__	’__	’__	’__	’__	’__	Mar-71
’__	’__	’__	’__	’__	’__	’__	’__	’__	’__	’__	’__	’__	’__	’__	Mar-70
’__	’__	’__	’__	’__	’__	’__	’__	’__	’__	’__	’__	’__	’__	’__	Mar-70
’__	’__	’__	’__	’__	’__	’__	’__	’__	’__	’__	’__	’__	’__	’__	Mar-71
’__	’__	’__	’__	’__	’__	’__	’__	’__	’__	’__	’__	’__	’__	’__	Mar-71
’__	’__	’__	’__	’__	’__	’__	’__	’__	’__	’__	’__	’__	’__	’__	Mar-70
’__	’__	’__	’__	’__	’__	’__	’__	’__	’__	’__	’__	’__	’__	’__	Mar-70
’__	’__	’__	’__	’__	’__	’__	’__	’__	’__	’__	’__	’__	’__	’__	Mar-70
’__	’__	’__	’__	’__	’__	’__	’__	’__	’__	’__	’__	’__	’__	’__	Mar-70
’__	’__	’__	’__	’__	’__	’__	’__	’__	’__	’__	’__	’__	’__	’__	Mar-70
’__	’__	’__	’__	’__	’__	’__	’__	’__	’__	’__	’__	’__	’__	’__	Mar-70
’__	’__	’__	’__	’__	’__	’__	’__	’__	’__	’__	’__	’__	’__	’__	Mar-71
’__	’__	’__	’__	’__	’__	’__	’__	’__	’__	’__	’__	’__	’__	’__	Mar-71
’__	’__	’__	’__	’__	’__	’__	’__	’__	’__	’__	’__	’__	’__	’__	Mar-70
’__	’__	’__	’__	’__	’__	’__	’__	’__	’__	’__	’__	’__	’__	’__	Mar-70
’__	’__	’__	’__	’__	’__	’__	’__	’__	’__	’__	’__	’__	’__	’__	Mar-70
’__	’__	’__	’__	’__	’__	’__	’__	’__	’__	’__	’__	’__	’__	’__	Mar-70
’__	’__	’__	’__	’__	’__	’__	’__	’__	’__	’__	’__	’__	’__	’__	Mar-70
’__	’__	’__	’__	’__	’__	’__	’__	’__	’__	’__	’__	’__	’__	’__	Mar-70
’__	’__	’__	’__	’__	’__	’__	’__	’__	’__	’__	’__	’__	’__	’__	Mar-71
’__	’__	’__	’__	’__	’__	’__	’__	’__	’__	’__	’__	’__	’__	’__	Mar-71
’__	’__	’__	’__	’__	’__	’__	’__	’__	’__	’__	’__	’__	’__	’__	Mar-71
’__	’__	’__	’__	’__	’__	’__	’__	’__	’__	’__	’__	’__	’__	’__	Mar-71
’__	’__	’__	’__	’__	’__	’__	’__	’__	’__	’__	’__	’__	’__	’__	Mar-70
’__	’__	’__	’__	’__	’__	’__	’__	’__	’__	’__	’__	’__	’__	’__	Mar-70
’__	’__	’__	’__	’__	’__	’__	’__	’__	’__	’__	’__	’__	’__	’__	Mar-70
’__	’__	’__	’__	’__	’__	’__	’__	’__	’__	’__	’__	’__	’__	’__	Mar-70
’__	’__	’__	’__	’__	’__	’__	’__	’__	’__	’__	’__	’__	’__	’__	Mar-70
’__	’__	’__	’__	’__	’__	’__	’__	’__	’__	’__	’__	’__	’__	’__	Mar-70
’__	’__	’__	’__	’__	’__	’__	’__	’__	’__	’__	’__	’__	’__	’__	Mar-70
’__	’__	’__	’__	’__	’__	’__	’__	’__	’__	’__	’__	’__	’__	’__	Mar-70
’__	’__	’__	’__	’__	’__	’__	’__	’__	’__	’__	’__	’__	’__	’__	Mar-71

'--	'--	'--	'--	'--	'--	'--	'--	'--	'--	'--	'--	'--	'--	'--	Mar-71
'--	'--	'--	'--	'--	'--	'--	'--	'--	'--	'--	'--	'--	'--	'--	Mar-70
'--	'--	'--	'--	'--	'--	'--	'--	'--	'--	'--	'--	'--	'--	'--	Mar-70
'--	'--	'--	'--	'--	'--	'--	'--	'--	'--	'--	'--	'--	'--	'--	Mar-70
'--	'--	'--	'--	'--	'--	'--	'--	'--	'--	'--	'--	'--	'--	'--	Mar-70
'--	'--	'--	'--	'--	'--	'--	'--	'--	'--	'--	'--	'--	'--	'--	Mar-71
'--	'--	'--	'--	'--	'--	'--	'--	'--	'--	'--	'--	'--	'--	'--	Mar-71
'--	'--	'--	'--	'--	'--	'--	'--	'--	'--	'--	'--	'--	'--	'--	Mar-70
'--	'--	'--	'--	'--	'--	'--	'--	'--	'--	'--	'--	'--	'--	'--	Mar-70
'--	'--	'--	'--	'--	'--	'--	'--	'--	'--	'--	'--	'--	'--	'--	Mar-70
'--	'--	'--	'--	'--	'--	'--	'--	'--	'--	'--	'--	'--	'--	'--	Mar-70
'--	'--	'--	'--	'--	'--	'--	'--	'--	'--	'--	'--	'--	'--	'--	Mar-70
'--	'--	'--	'--	'--	'--	'--	'--	'--	'--	'--	'--	'--	'--	'--	Mar-70
'--	'--	'--	'--	'--	'--	'--	'--	'--	'--	'--	'--	'--	'--	'--	Mar-70
'--	'--	'--	'--	'--	'--	'--	'--	'--	'--	'--	'--	'--	'--	'--	Mar-70
'--	'--	'--	'--	'--	'--	'--	'--	'--	'--	'--	'--	'--	'--	'--	Mar-70
'--	'--	'--	'--	'--	'--	'--	'--	'--	'--	'--	'--	'--	'--	'--	Mar-70
'--	'--	'--	'--	'--	'--	'--	'--	'--	'--	'--	'--	'--	'--	'--	Mar-70
'--	'--	'--	'--	'--	'--	'--	'--	'--	'--	'--	'--	'--	'--	'--	Mar-70
'--	'--	'--	'--	'--	'--	'--	'--	'--	'--	'--	'--	'--	'--	'--	Mar-70
'--	'--	'--	'--	'--	'--	'--	'--	'--	'--	'--	'--	'--	'--	'--	Mar-70
'--	'--	'--	'--	'--	'--	'--	'--	'--	'--	'--	'--	'--	'--	'--	Mar-70
'--	'--	'--	'--	'--	'--	'--	'--	'--	'--	'--	'--	'--	'--	'--	Mar-70
'--	'--	'--	'--	'--	'--	'--	'--	'--	'--	'--	'--	'--	'--	'--	Mar-70
'--	'--	'--	'--	'--	'--	'--	'--	'--	'--	'--	'--	'--	'--	'--	Mar-70
'--	'--	'--	'--	'--	'--	'--	'--	'--	'--	'--	'--	'--	'--	'--	Mar-70
'--	'--	'--	'--	'--	'--	'--	'--	'--	'--	'--	'--	'--	'--	'--	Mar-70
'--	'--	'--	'--	'--	'--	'--	'--	'--	'--	'--	'--	'--	'--	'--	Mar-70
'--	'--	'--	'--	'--	'--	'--	'--	'--	'--	'--	'--	'--	'--	'--	Mar-70
'--	'--	'--	'--	'--	'--	'--	'--	'--	'--	'--	'--	'--	'--	'--	Mar-70
'--	'--	'--	'--	'--	'--	'--	'--	'--	'--	'--	'--	'--	'--	'--	Mar-70
'--	'--	'--	'--	'--	'--	'--	'--	'--	'--	'--	'--	'--	'--	'--	Mar-71
'--	'--	'--	'--	'--	'--	'--	'--	'--	'--	'--	'--	'--	'--	'--	Mar-71
'--	'--	'--	'--	'--	'--	'--	'--	'--	'--	'--	'--	'--	'--	'--	Mar-70
'--	'--	'--	'--	'--	'--	'--	'--	'--	'--	'--	'--	'--	'--	'--	Mar-70

'__	'__	'__	'__	'__	'__	'__	'__	'__	'__	'__	'__	'__	'__	'__	Mar-70
'__	'__	'__	'__	'__	'__	'__	'__	'__	'__	'__	'__	'__	'__	'__	Mar-70
'__	'__	'__	'__	'__	'__	'__	'__	'__	'__	'__	'__	'__	'__	'__	Mar-70
'__	'__	'__	'__	'__	'__	'__	'__	'__	'__	'__	'__	'__	'__	'__	Mar-70
'__	'__	'__	'__	'__	'__	'__	'__	'__	'__	'__	'__	'__	'__	'__	Mar-70
'__	'__	'__	'__	'__	'__	'__	'__	'__	'__	'__	'__	'__	'__	'__	Mar-70
'__	'__	'__	'__	'__	'__	'__	'__	'__	'__	'__	'__	'__	'__	'__	Mar-70
'__	'__	'__	'__	'__	'__	'__	'__	'__	'__	'__	'__	'__	'__	'__	Mar-70
'__	'__	'__	'__	'__	'__	'__	'__	'__	'__	'__	'__	'__	'__	'__	Mar-70
'__	'__	'__	'__	'__	'__	'__	'__	'__	'__	'__	'__	'__	'__	'__	Mar-70
'__	'__	'__	'__	'__	'__	'__	'__	'__	'__	'__	'__	'__	'__	'__	Mar-70
'__	'__	'__	'__	'__	'__	'__	'__	'__	'__	'__	'__	'__	'__	'__	Mar-70
'__	'__	'__	'__	'__	'__	'__	'__	'__	'__	'__	'__	'__	'__	'__	Mar-70
'__	'__	'__	'__	'__	'__	'__	'__	'__	'__	'__	'__	'__	'__	'__	Mar-70
'__	'__	'__	'__	'__	'__	'__	'__	'__	'__	'__	'__	'__	'__	'__	Mar-70
'__	'__	'__	'__	'__	'__	'__	'__	'__	'__	'__	'__	'__	'__	'__	Mar-70
'__	'__	'__	'__	'__	'__	'__	'__	'__	'__	'__	'__	'__	'__	'__	Mar-70
'__	'__	'__	'__	'__	'__	'__	'__	'__	'__	'__	'__	'__	'__	'__	Mar-70
'__	'__	'__	'__	'__	'__	'__	'__	'__	'__	'__	'__	'__	'__	'__	Mar-70
'__	'__	'__	'__	'__	'__	'__	'__	'__	'__	'__	'__	'__	'__	'__	Mar-70
'__	'__	'__	'__	'__	'__	'__	'__	'__	'__	'__	'__	'__	'__	'__	Mar-70
'__	'__	'__	'__	'__	'__	'__	'__	'__	'__	'__	'__	'__	'__	'__	Mar-70
'__	'__	'__	'__	'__	'__	'__	'__	'__	'__	'__	'__	'__	'__	'__	Mar-70
'__	'__	'__	'__	'__	'__	'__	'__	'__	'__	'__	'__	'__	'__	'__	Mar-70
'__	'__	'__	'__	'__	'__	'__	'__	'__	'__	'__	'__	'__	'__	'__	Mar-70
'__	'__	'__	'__	'__	'__	'__	'__	'__	'__	'__	'__	'__	'__	'__	Mar-70
'__	'__	'__	'__	'__	'__	'__	'__	'__	'__	'__	'__	'__	'__	'__	Mar-71
'__	'__	'__	'__	'__	'__	'__	'__	'__	'__	'__	'__	'__	'__	'__	Mar-71
'__	'__	'__	'__	'__	'__	'__	'__	'__	'__	'__	'__	'__	'__	'__	Mar-70
'__	'__	'__	'__	'__	'__	'__	'__	'__	'__	'__	'__	'__	'__	'__	Mar-70
'__	'__	'__	'__	'__	'__	'__	'__	'__	'__	'__	'__	'__	'__	'__	Mar-70
'__	'__	'__	'__	'__	'__	'__	'__	'__	'__	'__	'__	'__	'__	'__	Mar-70
'__	'__	'__	'__	'__	'__	'__	'__	'__	'__	'__	'__	'__	'__	'__	Mar-70
'__	'__	'__	'__	'__	'__	'__	'__	'__	'__	'__	'__	'__	'__	'__	Mar-70
'__	'__	'__	'__	'__	'__	'__	'__	'__	'__	'__	'__	'__	'__	'__	Mar-70

'__	'__	'__	'__	'__	'__	'__	'__	'__	'__	'__	'__	'__	'__	'__	Mar-70
'__	'__	'__	'__	'__	'__	'__	'__	'__	'__	'__	'__	'__	'__	'__	Mar-70
'__	'__	'__	'__	'__	'__	'__	'__	'__	'__	'__	'__	'__	'__	'__	Mar-70
'__	'__	'__	'__	'__	'__	'__	'__	'__	'__	'__	'__	'__	'__	'__	Mar-70
'__	'__	'__	'__	'__	'__	'__	'__	'__	'__	'__	'__	'__	'__	'__	Mar-70
'__	'__	'__	'__	'__	'__	'__	'__	'__	'__	'__	'__	'__	'__	'__	Mar-70
'__	'__	'__	'__	'__	'__	'__	'__	'__	'__	'__	'__	'__	'__	'__	Mar-70
'__	'__	'__	'__	'__	'__	'__	'__	'__	'__	'__	'__	'__	'__	'__	Mar-70
'__	'__	'__	'__	'__	'__	'__	'__	'__	'__	'__	'__	'__	'__	'__	Mar-70
'__	'__	'__	'__	'__	'__	'__	'__	'__	'__	'__	'__	'__	'__	'__	Mar-70
'__	'__	'__	'__	'__	'__	'__	'__	'__	'__	'__	'__	'__	'__	'__	Mar-70
'__	'__	'__	'__	'__	'__	'__	'__	'__	'__	'__	'__	'__	'__	'__	Mar-70
'__	'__	'__	'__	'__	'__	'__	'__	'__	'__	'__	'__	'__	'__	'__	Mar-70
'__	'__	'__	'__	'__	'__	'__	'__	'__	'__	'__	'__	'__	'__	'__	Mar-70
'__	'__	'__	'__	'__	'__	'__	'__	'__	'__	'__	'__	'__	'__	'__	Mar-70
'__	'__	'__	'__	'__	'__	'__	'__	'__	'__	'__	'__	'__	'__	'__	Mar-70
'__	'__	'__	'__	'__	'__	'__	'__	'__	'__	'__	'__	'__	'__	'__	Mar-70
'__	'__	'__	'__	'__	'__	'__	'__	'__	'__	'__	'__	'__	'__	'__	Mar-70
'__	'__	'__	'__	'__	'__	'__	'__	'__	'__	'__	'__	'__	'__	'__	Mar-70
'__	'__	'__	'__	'__	'__	'__	'__	'__	'__	'__	'__	'__	'__	'__	Mar-70
'__	'__	'__	'__	'__	'__	'__	'__	'__	'__	'__	'__	'__	'__	'__	Mar-70
'__	'__	'__	'__	'__	'__	'__	'__	'__	'__	'__	'__	'__	'__	'__	Mar-70
'__	'__	'__	'__	'__	'__	'__	'__	'__	'__	'__	'__	'__	'__	'__	Mar-70
'__	'__	'__	'__	'__	'__	'__	'__	'__	'__	'__	'__	'__	'__	'__	Mar-70
'__	'__	'__	'__	'__	'__	'__	'__	'__	'__	'__	'__	'__	'__	'__	Mar-70
'__	'__	'__	'__	'__	'__	'__	'__	'__	'__	'__	'__	'__	'__	'__	Mar-70
'__	'__	'__	'__	'__	'__	'__	'__	'__	'__	'__	'__	'__	'__	'__	Mar-70
'__	'__	'__	'__	'__	'__	'__	'__	'__	'__	'__	'__	'__	'__	'__	Mar-70
'__	'__	'__	'__	'__	'__	'__	'__	'__	'__	'__	'__	'__	'__	'__	Mar-70
'__	'__	'__	'__	'__	'__	'__	'__	'__	'__	'__	'__	'__	'__	'__	Mar-70
'__	'__	'__	'__	'__	'__	'__	'__	'__	'__	'__	'__	'__	'__	'__	Mar-70
'__	'__	'__	'__	'__	'__	'__	'__	'__	'__	'__	'__	'__	'__	'__	Mar-71
'__	'__	'__	'__	'__	'__	'__	'__	'__	'__	'__	'__	'__	'__	'__	Mar-71
'__	'__	'__	'__	'__	'__	'__	'__	'__	'__	'__	'__	'__	'__	'__	Mar-71
'__	'__	'__	'__	'__	'__	'__	'__	'__	'__	'__	'__	'__	'__	'__	Mar-71

'__	'__	'__	'__	'__	'__	'__	'__	'__	'__	'__	'__	'__	'__	'__	Mar-70
'__	'__	'__	'__	'__	'__	'__	'__	'__	'__	'__	'__	'__	'__	'__	Mar-70
'__	'__	'__	'__	'__	'__	'__	'__	'__	'__	'__	'__	'__	'__	'__	Mar-70
'__	'__	'__	'__	'__	'__	'__	'__	'__	'__	'__	'__	'__	'__	'__	Mar-70
'__	'__	'__	'__	'__	'__	'__	'__	'__	'__	'__	'__	'__	'__	'__	Mar-70
'__	'__	'__	'__	'__	'__	'__	'__	'__	'__	'__	'__	'__	'__	'__	Mar-70
'__	'__	'__	'__	'__	'__	'__	'__	'__	'__	'__	'__	'__	'__	'__	Mar-71
'__	'__	'__	'__	'__	'__	'__	'__	'__	'__	'__	'__	'__	'__	'__	Mar-71
'__	'__	'__	'__	'__	'__	'__	'__	'__	'__	'__	'__	'__	'__	'__	Mar-70
'__	'__	'__	'__	'__	'__	'__	'__	'__	'__	'__	'__	'__	'__	'__	Mar-70
'__	'__	'__	'__	'__	'__	'__	'__	'__	'__	'__	'__	'__	'__	'__	Mar-70
'__	'__	'__	'__	'__	'__	'__	'__	'__	'__	'__	'__	'__	'__	'__	Mar-70
'__	'__	'__	'__	'__	'__	'__	'__	'__	'__	'__	'__	'__	'__	'__	Mar-70
'__	'__	'__	'__	'__	'__	'__	'__	'__	'__	'__	'__	'__	'__	'__	Mar-70
'__	'__	'__	'__	'__	'__	'__	'__	'__	'__	'__	'__	'__	'__	'__	Mar-70
'__	'__	'__	'__	'__	'__	'__	'__	'__	'__	'__	'__	'__	'__	'__	Mar-70
'__	'__	'__	'__	'__	'__	'__	'__	'__	'__	'__	'__	'__	'__	'__	Mar-70
'__	'__	'__	'__	'__	'__	'__	'__	'__	'__	'__	'__	'__	'__	'__	Mar-70
'__	'__	'__	'__	'__	'__	'__	'__	'__	'__	'__	'__	'__	'__	'__	Mar-71
'__	'__	'__	'__	'__	'__	'__	'__	'__	'__	'__	'__	'__	'__	'__	Mar-71
'__	'__	'__	'__	'__	'__	'__	'__	'__	'__	'__	'__	'__	'__	'__	Mar-70
'__	'__	'__	'__	'__	'__	'__	'__	'__	'__	'__	'__	'__	'__	'__	Mar-70
'__	'__	'__	'__	'__	'__	'__	'__	'__	'__	'__	'__	'__	'__	'__	Mar-70
'__	'__	'__	'__	'__	'__	'__	'__	'__	'__	'__	'__	'__	'__	'__	Mar-70
'__	'__	'__	'__	'__	'__	'__	'__	'__	'__	'__	'__	'__	'__	'__	Mar-70
'__	'__	'__	'__	'__	'__	'__	'__	'__	'__	'__	'__	'__	'__	'__	Mar-70
'__	'__	'__	'__	'__	'__	'__	'__	'__	'__	'__	'__	'__	'__	'__	Mar-70
'__	'__	'__	'__	'__	'__	'__	'__	'__	'__	'__	'__	'__	'__	'__	Mar-70
'__	'__	'__	'__	'__	'__	'__	'__	'__	'__	'__	'__	'__	'__	'__	Mar-70
'__	'__	'__	'__	'__	'__	'__	'__	'__	'__	'__	'__	'__	'__	'__	Mar-70
'__	'__	'__	'__	'__	'__	'__	'__	'__	'__	'__	'__	'__	'__	'__	Mar-70
'__	'__	'__	'__	'__	'__	'__	'__	'__	'__	'__	'__	'__	'__	'__	Mar-70
'__	'__	'__	'__	'__	'__	'__	'__	'__	'__	'__	'__	'__	'__	'__	Mar-70
'__	'__	'__	'__	'__	'__	'__	'__	'__	'__	'__	'__	'__	'__	'__	Mar-70
'__	'__	'__	'__	'__	'__	'__	'__	'__	'__	'__	'__	'__	'__	'__	Mar-70

’__	’__	’__	’__	’__	’__	’__	’__	’__	’__	’__	’__	’__	’__	’__	Mar-70
’__	’__	’__	’__	’__	’__	’__	’__	’__	’__	’__	’__	’__	’__	’__	Mar-70
’__	’__	’__	’__	’__	’__	’__	’__	’__	’__	’__	’__	’__	’__	’__	Mar-70
’__	’__	’__	’__	’__	’__	’__	’__	’__	’__	’__	’__	’__	’__	’__	Mar-70
’__	’__	’__	’__	’__	’__	’__	’__	’__	’__	’__	’__	’__	’__	’__	Mar-70
’__	’__	’__	’__	’__	’__	’__	’__	’__	’__	’__	’__	’__	’__	’__	Mar-70
’__	’__	’__	’__	’__	’__	’__	’__	’__	’__	’__	’__	’__	’__	’__	Mar-70
’__	’__	’__	’__	’__	’__	’__	’__	’__	’__	’__	’__	’__	’__	’__	Mar-70
’__	’__	’__	’__	’__	’__	’__	’__	’__	’__	’__	’__	’__	’__	’__	Mar-70
’__	’__	’__	’__	’__	’__	’__	’__	’__	’__	’__	’__	’__	’__	’__	Mar-70
’__	’__	’__	’__	’__	’__	’__	’__	’__	’__	’__	’__	’__	’__	’__	Mar-70
’__	’__	’__	’__	’__	’__	’__	’__	’__	’__	’__	’__	’__	’__	’__	Mar-70
’__	’__	’__	’__	’__	’__	’__	’__	’__	’__	’__	’__	’__	’__	’__	Mar-70
’__	’__	’__	’__	’__	’__	’__	’__	’__	’__	’__	’__	’__	’__	’__	Mar-70
’__	’__	’__	’__	’__	’__	’__	’__	’__	’__	’__	’__	’__	’__	’__	Mar-70
’__	’__	’__	’__	’__	’__	’__	’__	’__	’__	’__	’__	’__	’__	’__	Mar-70
’__	’__	’__	’__	’__	’__	’__	’__	’__	’__	’__	’__	’__	’__	’__	Mar-70
’__	’__	’__	’__	’__	’__	’__	’__	’__	’__	’__	’__	’__	’__	’__	Mar-70
’__	’__	’__	’__	’__	’__	’__	’__	’__	’__	’__	’__	’__	’__	’__	Mar-70
’__	’__	’__	’__	’__	’__	’__	’__	’__	’__	’__	’__	’__	’__	’__	Mar-70
’__	’__	’__	’__	’__	’__	’__	’__	’__	’__	’__	’__	’__	’__	’__	Mar-70
’__	’__	’__	’__	’__	’__	’__	’__	’__	’__	’__	’__	’__	’__	’__	Mar-70
’__	’__	’__	’__	’__	’__	’__	’__	’__	’__	’__	’__	’__	’__	’__	Mar-70
’__	’__	’__	’__	’__	’__	’__	’__	’__	’__	’__	’__	’__	’__	’__	Mar-70
’__	’__	’__	’__	’__	’__	’__	’__	’__	’__	’__	’__	’__	’__	’__	Mar-70
’__	’__	’__	’__	’__	’__	’__	’__	’__	’__	’__	’__	’__	’__	’__	Mar-70
’__	’__	’__	’__	’__	’__	’__	’__	’__	’__	’__	’__	’__	’__	’__	Mar-70
’__	’__	’__	’__	’__	’__	’__	’__	’__	’__	’__	’__	’__	’__	’__	Mar-70
’__	’__	’__	’__	’__	’__	’__	’__	’__	’__	’__	’__	’__	’__	’__	Mar-70
’__	’__	’__	’__	’__	’__	’__	’__	’__	’__	’__	’__	’__	’__	’__	Mar-70
’__	’__	’__	’__	’__	’__	’__	’__	’__	’__	’__	’__	’__	’__	’__	Mar-70
’__	’__	’__	’__	’__	’__	’__	’__	’__	’__	’__	’__	’__	’__	’__	Mar-70
’__	’__	’__	’__	’__	’__	’__	’__	’__	’__	’__	’__	’__	’__	’__	Mar-70
’__	’__	’__	’__	’__	’__	’__	’__	’__	’__	’__	’__	’__	’__	’__	Mar-70
’__	’__	’__	’__	’__	’__	’__	’__	’__	’__	’__	’__	’__	’__	’__	Mar-70

'--	'--	'--	'--	'--	'--	'--	'--	'--	'--	'--	'--	'--	'--	'--	Mar-70
'--	'--	'--	'--	'--	'--	'--	'--	'--	'--	'--	'--	'--	'--	'--	Mar-70
'--	'--	'--	'--	'--	'--	'--	'--	'--	'--	'--	'--	'--	'--	'--	Mar-70
'--	'--	'--	'--	'--	'--	'--	'--	'--	'--	'--	'--	'--	'--	'--	Mar-70
'--	'--	'--	'--	'--	'--	'--	'--	'--	'--	'--	'--	'--	'--	'--	Mar-70
'--	'--	'--	'--	'--	'--	'--	'--	'--	'--	'--	'--	'--	'--	'--	Mar-70
'--	'--	'--	'--	'--	'--	'--	'--	'--	'--	'--	'--	'--	'--	'--	Mar-71
'--	'--	'--	'--	'--	'--	'--	'--	'--	'--	'--	'--	'--	'--	'--	Mar-71
'--	'--	'--	'--	'--	'--	'--	'--	'--	'--	'--	'--	'--	'--	'--	Mar-70
'--	'--	'--	'--	'--	'--	'--	'--	'--	'--	'--	'--	'--	'--	'--	Mar-70

non_nodal	non_nodal	ovarian_s	ovarian_s	papillary	percent_t	perineura	peripancr	peripancr	peritonea	pregnant_	primary_c	primary_g	prior_mal	prior_tre	progressi
'--	'--	'--	'--	'--	'--	'--	'--	'--	'--	'--	Squamous	'--	no	No	not repor
'--	'--	'--	'--	'--	'--	'--	'--	'--	'--	'--	Squamous	'--	no	No	not repor
'--	'--	'--	'--	'--	'--	'--	'--	'--	'--	'--	Squamous	'--	no	No	not repor
'--	'--	'--	'--	'--	'--	'--	'--	'--	'--	'--	Squamous	'--	no	No	not repor
'--	'--	'--	'--	'--	'--	'--	'--	'--	'--	'--	Squamous	'--	no	No	not repor
'--	'--	'--	'--	'--	'--	'--	'--	'--	'--	'--	Squamous	'--	no	No	not repor
'--	'--	'--	'--	'--	'--	'--	'--	'--	'--	'--	Squamous	'--	no	No	not repor
'--	'--	'--	'--	'--	'--	'--	'--	'--	'--	'--	Squamous	'--	no	No	not repor
'--	'--	'--	'--	'--	'--	'--	'--	'--	'--	'--	Squamous	'--	no	No	not repor
'--	'--	'--	'--	'--	'--	'--	'--	'--	'--	'--	Squamous	'--	no	No	not repor
'--	'--	'--	'--	'--	'--	'--	'--	'--	'--	'--	Squamous	'--	no	No	not repor
'--	'--	'--	'--	'--	'--	'--	'--	'--	'--	'--	Squamous	'--	no	No	not repor
'--	'--	'--	'--	'--	'--	'--	'--	'--	'--	'--	Squamous	'--	no	No	not repor
'--	'--	'--	'--	'--	'--	'--	'--	'--	'--	'--	Squamous	'--	no	No	not repor
'--	'--	'--	'--	'--	'--	'--	'--	'--	'--	'--	Squamous	'--	no	No	not repor
'--	'--	'--	'--	'--	'--	'--	'--	'--	'--	'--	Squamous	'--	no	No	not repor
'--	'--	'--	'--	'--	'--	'--	'--	'--	'--	'--	Squamous	'--	no	No	not repor
'--	'--	'--	'--	'--	'--	'--	'--	'--	'--	'--	Squamous	'--	no	No	not repor
'--	'--	'--	'--	'--	'--	'--	'--	'--	'--	'--	Squamous	'--	yes	No	not repor
'--	'--	'--	'--	'--	'--	'--	'--	'--	'--	'--	Squamous	'--	yes	No	not repor
'--	'--	'--	'--	'--	'--	'--	'--	'--	'--	'--	Squamous	'--	no	No	not repor
'--	'--	'--	'--	'--	'--	'--	'--	'--	'--	'--	Squamous	'--	no	No	not repor
'--	'--	'--	'--	'--	'--	'--	'--	'--	'--	'--	Squamous	'--	no	No	not repor
'--	'--	'--	'--	'--	'--	'--	'--	'--	'--	'--	Squamous	'--	no	No	not repor
'--	'--	'--	'--	'--	'--	'--	'--	'--	'--	'--	Squamous	'--	no	No	not repor
'--	'--	'--	'--	'--	'--	'--	'--	'--	'--	'--	Squamous	'--	no	No	not repor
'--	'--	'--	'--	'--	'--	'--	'--	'--	'--	'--	Squamous	'--	no	No	not repor
'--	'--	'--	'--	'--	'--	'--	'--	'--	'--	'--	Squamous	'--	no	No	not repor
'--	'--	'--	'--	'--	'--	'--	'--	'--	'--	'--	Squamous	'--	no	No	not repor
'--	'--	'--	'--	'--	'--	'--	'--	'--	'--	'--	Squamous	'--	no	No	not repor
'--	'--	'--	'--	'--	'--	'--	'--	'--	'--	'--	Squamous	'--	no	No	not repor
'--	'--	'--	'--	'--	'--	'--	'--	'--	'--	'--	Squamous	'--	no	No	not repor
'--	'--	'--	'--	'--	'--	'--	'--	'--	'--	'--	Squamous	'--	no	No	not repor
'--	'--	'--	'--	'--	'--	'--	'--	'--	'--	'--	Squamous	'--	no	No	not repor

'--	'--	'--	'--	'--	'--	'--	'--	'--	'--	'--	Squamous	'--	no	No	not repor
'--	'--	'--	'--	'--	'--	'--	'--	'--	'--	'--	Squamous	'--	no	No	not repor
'--	'--	'--	'--	'--	'--	'--	'--	'--	'--	'--	Squamous	'--	no	No	not repor
'--	'--	'--	'--	'--	'--	'--	'--	'--	'--	'--	Squamous	'--	no	No	not repor
'--	'--	'--	'--	'--	'--	'--	'--	'--	'--	'--	Squamous	'--	no	No	not repor
'--	'--	'--	'--	'--	'--	'--	'--	'--	'--	'--	Squamous	'--	no	No	not repor
'--	'--	'--	'--	'--	'--	'--	'--	'--	'--	'--	Squamous	'--	yes	No	not repor
'--	'--	'--	'--	'--	'--	'--	'--	'--	'--	'--	Squamous	'--	yes	No	not repor
'--	'--	'--	'--	'--	'--	'--	'--	'--	'--	'--	Squamous	'--	no	No	not repor
'--	'--	'--	'--	'--	'--	'--	'--	'--	'--	'--	Squamous	'--	no	No	not repor
'--	'--	'--	'--	'--	'--	'--	'--	'--	'--	'--	Squamous	'--	no	No	not repor
'--	'--	'--	'--	'--	'--	'--	'--	'--	'--	'--	Squamous	'--	no	No	not repor
'--	'--	'--	'--	'--	'--	'--	'--	'--	'--	'--	Squamous	'--	no	No	not repor
'--	'--	'--	'--	'--	'--	'--	'--	'--	'--	'--	Squamous	'--	no	No	not repor
'--	'--	'--	'--	'--	'--	'--	'--	'--	'--	'--	Squamous	'--	no	No	not repor
'--	'--	'--	'--	'--	'--	'--	'--	'--	'--	'--	Squamous	'--	no	No	not repor
'--	'--	'--	'--	'--	'--	'--	'--	'--	'--	'--	Squamous	'--	no	No	not repor
'--	'--	'--	'--	'--	'--	'--	'--	'--	'--	'--	Squamous	'--	no	No	not repor
'--	'--	'--	'--	'--	'--	'--	'--	'--	'--	'--	Squamous	'--	no	No	not repor
'--	'--	'--	'--	'--	'--	'--	'--	'--	'--	'--	Squamous	'--	no	No	not repor
'--	'--	'--	'--	'--	'--	'--	'--	'--	'--	'--	Squamous	'--	no	No	not repor
'--	'--	'--	'--	'--	'--	'--	'--	'--	'--	'--	Squamous	'--	no	No	not repor
'--	'--	'--	'--	'--	'--	'--	'--	'--	'--	'--	Squamous	'--	no	No	not repor
'--	'--	'--	'--	'--	'--	'--	'--	'--	'--	'--	Squamous	'--	no	No	not repor
'--	'--	'--	'--	'--	'--	'--	'--	'--	'--	'--	Squamous	'--	no	No	not repor
'--	'--	'--	'--	'--	'--	'--	'--	'--	'--	'--	Squamous	'--	no	No	not repor
'--	'--	'--	'--	'--	'--	'--	'--	'--	'--	'--	Squamous	'--	no	No	not repor
'--	'--	'--	'--	'--	'--	'--	'--	'--	'--	'--	Squamous	'--	no	No	not repor
'--	'--	'--	'--	'--	'--	'--	'--	'--	'--	'--	Squamous	'--	no	No	not repor
'--	'--	'--	'--	'--	'--	'--	'--	'--	'--	'--	Squamous	'--	no	No	not repor
'--	'--	'--	'--	'--	'--	'--	'--	'--	'--	'--	Squamous	'--	no	No	not repor
'--	'--	'--	'--	'--	'--	'--	'--	'--	'--	'--	Squamous	'--	no	No	not repor
'--	'--	'--	'--	'--	'--	'--	'--	'--	'--	'--	Squamous	'--	no	No	not repor
'--	'--	'--	'--	'--	'--	'--	'--	'--	'--	'--	Squamous	'--	no	No	not repor
'--	'--	'--	'--	'--	'--	'--	'--	'--	'--	'--	Squamous	'--	no	No	not repor

'--	'--	'--	'--	'--	'--	'--	'--	'--	'--	'--	Squamous	'--	no	No	not repor
'--	'--	'--	'--	'--	'--	'--	'--	'--	'--	'--	Squamous	'--	no	No	not repor
'--	'--	'--	'--	'--	'--	'--	'--	'--	'--	'--	Squamous	'--	no	No	not repor
'--	'--	'--	'--	'--	'--	'--	'--	'--	'--	'--	Squamous	'--	no	No	not repor
'--	'--	'--	'--	'--	'--	'--	'--	'--	'--	'--	Squamous	'--	no	No	not repor
'--	'--	'--	'--	'--	'--	'--	'--	'--	'--	'--	Squamous	'--	no	No	not repor
'--	'--	'--	'--	'--	'--	'--	'--	'--	'--	'--	Squamous	'--	no	No	not repor
'--	'--	'--	'--	'--	'--	'--	'--	'--	'--	'--	Squamous	'--	no	No	not repor
'--	'--	'--	'--	'--	'--	'--	'--	'--	'--	'--	Squamous	'--	no	No	not repor
'--	'--	'--	'--	'--	'--	'--	'--	'--	'--	'--	Squamous	'--	no	No	not repor
'--	'--	'--	'--	'--	'--	'--	'--	'--	'--	'--	Squamous	'--	no	No	not repor
'--	'--	'--	'--	'--	'--	'--	'--	'--	'--	'--	Squamous	'--	no	No	not repor
'--	'--	'--	'--	'--	'--	'--	'--	'--	'--	'--	Squamous	'--	no	No	not repor
'--	'--	'--	'--	'--	'--	'--	'--	'--	'--	'--	Squamous	'--	no	No	not repor
'--	'--	'--	'--	'--	'--	'--	'--	'--	'--	'--	Squamous	'--	no	No	not repor
'--	'--	'--	'--	'--	'--	'--	'--	'--	'--	'--	Squamous	'--	no	No	not repor
'--	'--	'--	'--	'--	'--	'--	'--	'--	'--	'--	Squamous	'--	no	No	not repor
'--	'--	'--	'--	'--	'--	'--	'--	'--	'--	'--	Squamous	'--	no	No	not repor
'--	'--	'--	'--	'--	'--	'--	'--	'--	'--	'--	Squamous	'--	no	No	not repor
'--	'--	'--	'--	'--	'--	'--	'--	'--	'--	'--	Squamous	'--	no	No	not repor
'--	'--	'--	'--	'--	'--	'--	'--	'--	'--	'--	Squamous	'--	no	No	not repor
'--	'--	'--	'--	'--	'--	'--	'--	'--	'--	'--	Squamous	'--	no	No	not repor
'--	'--	'--	'--	'--	'--	'--	'--	'--	'--	'--	Squamous	'--	no	No	not repor
'--	'--	'--	'--	'--	'--	'--	'--	'--	'--	'--	Squamous	'--	no	No	not repor
'--	'--	'--	'--	'--	'--	'--	'--	'--	'--	'--	Squamous	'--	no	No	not repor
'--	'--	'--	'--	'--	'--	'--	'--	'--	'--	'--	Squamous	'--	no	No	not repor
'--	'--	'--	'--	'--	'--	'--	'--	'--	'--	'--	Squamous	'--	no	No	not repor
'--	'--	'--	'--	'--	'--	'--	'--	'--	'--	'--	Squamous	'--	no	No	not repor
'--	'--	'--	'--	'--	'--	'--	'--	'--	'--	'--	Squamous	'--	no	No	not repor
'--	'--	'--	'--	'--	'--	'--	'--	'--	'--	'--	Squamous	'--	no	No	not repor
'--	'--	'--	'--	'--	'--	'--	'--	'--	'--	'--	Squamous	'--	no	No	not repor
'--	'--	'--	'--	'--	'--	'--	'--	'--	'--	'--	Squamous	'--	no	No	not repor
'--	'--	'--	'--	'--	'--	'--	'--	'--	'--	'--	Squamous	'--	no	No	not repor
'--	'--	'--	'--	'--	'--	'--	'--	'--	'--	'--	Squamous	'--	no	No	not repor
'--	'--	'--	'--	'--	'--	'--	'--	'--	'--	'--	Squamous	'--	no	No	not repor

'--	'--	'--	'--	'--	'--	'--	'--	'--	'--	'--	Squamous	'--	no	No	not repor
'--	'--	'--	'--	'--	'--	'--	'--	'--	'--	'--	Squamous	'--	no	No	not repor
'--	'--	'--	'--	'--	'--	'--	'--	'--	'--	'--	Squamous	'--	no	No	not repor
'--	'--	'--	'--	'--	'--	'--	'--	'--	'--	'--	Squamous	'--	no	No	not repor
'--	'--	'--	'--	'--	'--	'--	'--	'--	'--	'--	Squamous	'--	no	No	not repor
'--	'--	'--	'--	'--	'--	'--	'--	'--	'--	'--	Squamous	'--	no	No	not repor
'--	'--	'--	'--	'--	'--	'--	'--	'--	'--	'--	Squamous	'--	no	No	not repor
'--	'--	'--	'--	'--	'--	'--	'--	'--	'--	'--	Squamous	'--	no	No	not repor
'--	'--	'--	'--	'--	'--	'--	'--	'--	'--	'--	Squamous	'--	no	No	not repor
'--	'--	'--	'--	'--	'--	'--	'--	'--	'--	'--	Squamous	'--	no	No	not repor
'--	'--	'--	'--	'--	'--	'--	'--	'--	'--	'--	Squamous	'--	no	No	not repor
'--	'--	'--	'--	'--	'--	'--	'--	'--	'--	'--	Squamous	'--	no	No	not repor
'--	'--	'--	'--	'--	'--	'--	'--	'--	'--	'--	Squamous	'--	no	No	not repor
'--	'--	'--	'--	'--	'--	'--	'--	'--	'--	'--	Squamous	'--	no	No	not repor
'--	'--	'--	'--	'--	'--	'--	'--	'--	'--	'--	Squamous	'--	no	No	not repor
'--	'--	'--	'--	'--	'--	'--	'--	'--	'--	'--	Squamous	'--	no	No	not repor
'--	'--	'--	'--	'--	'--	'--	'--	'--	'--	'--	Squamous	'--	no	No	not repor
'--	'--	'--	'--	'--	'--	'--	'--	'--	'--	'--	Squamous	'--	no	No	not repor
'--	'--	'--	'--	'--	'--	'--	'--	'--	'--	'--	Squamous	'--	no	No	not repor
'--	'--	'--	'--	'--	'--	'--	'--	'--	'--	'--	Squamous	'--	no	No	not repor
'--	'--	'--	'--	'--	'--	'--	'--	'--	'--	'--	Squamous	'--	no	No	not repor
'--	'--	'--	'--	'--	'--	'--	'--	'--	'--	'--	Squamous	'--	no	No	not repor
'--	'--	'--	'--	'--	'--	'--	'--	'--	'--	'--	Squamous	'--	no	No	not repor
'--	'--	'--	'--	'--	'--	'--	'--	'--	'--	'--	Squamous	'--	no	No	not repor
'--	'--	'--	'--	'--	'--	'--	'--	'--	'--	'--	Squamous	'--	no	No	not repor
'--	'--	'--	'--	'--	'--	'--	'--	'--	'--	'--	Squamous	'--	no	No	not repor
'--	'--	'--	'--	'--	'--	'--	'--	'--	'--	'--	Squamous	'--	no	No	not repor
'--	'--	'--	'--	'--	'--	'--	'--	'--	'--	'--	Squamous	'--	no	No	not repor
'--	'--	'--	'--	'--	'--	'--	'--	'--	'--	'--	Squamous	'--	no	No	not repor
'--	'--	'--	'--	'--	'--	'--	'--	'--	'--	'--	Squamous	'--	no	No	not repor
'--	'--	'--	'--	'--	'--	'--	'--	'--	'--	'--	Squamous	'--	no	No	not repor
'--	'--	'--	'--	'--	'--	'--	'--	'--	'--	'--	Squamous	'--	no	No	not repor
'--	'--	'--	'--	'--	'--	'--	'--	'--	'--	'--	Squamous	'--	no	No	not repor
'--	'--	'--	'--	'--	'--	'--	'--	'--	'--	'--	Squamous	'--	no	No	not repor
'--	'--	'--	'--	'--	'--	'--	'--	'--	'--	'--	Squamous	'--	no	No	not repor

'--	'--	'--	'--	'--	'--	'--	'--	'--	'--	'--	Squamous	'--	no	No	not repor
'--	'--	'--	'--	'--	'--	'--	'--	'--	'--	'--	Squamous	'--	no	No	not repor
'--	'--	'--	'--	'--	'--	'--	'--	'--	'--	'--	Squamous	'--	no	No	not repor
'--	'--	'--	'--	'--	'--	'--	'--	'--	'--	'--	Squamous	'--	no	No	not repor
'--	'--	'--	'--	'--	'--	'--	'--	'--	'--	'--	Squamous	'--	no	No	not repor
'--	'--	'--	'--	'--	'--	'--	'--	'--	'--	'--	Squamous	'--	no	No	not repor
'--	'--	'--	'--	'--	'--	'--	'--	'--	'--	'--	Squamous	'--	no	No	not repor
'--	'--	'--	'--	'--	'--	'--	'--	'--	'--	'--	Squamous	'--	no	No	not repor
'--	'--	'--	'--	'--	'--	'--	'--	'--	'--	'--	Squamous	'--	no	No	not repor
'--	'--	'--	'--	'--	'--	'--	'--	'--	'--	'--	Squamous	'--	no	No	not repor
'--	'--	'--	'--	'--	'--	'--	'--	'--	'--	'--	Squamous	'--	no	No	not repor
'--	'--	'--	'--	'--	'--	'--	'--	'--	'--	'--	Squamous	'--	no	No	not repor
'--	'--	'--	'--	'--	'--	'--	'--	'--	'--	'--	Squamous	'--	no	No	not repor
'--	'--	'--	'--	'--	'--	'--	'--	'--	'--	'--	Squamous	'--	no	No	not repor
'--	'--	'--	'--	'--	'--	'--	'--	'--	'--	'--	Squamous	'--	no	No	not repor
'--	'--	'--	'--	'--	'--	'--	'--	'--	'--	'--	Squamous	'--	no	No	not repor
'--	'--	'--	'--	'--	'--	'--	'--	'--	'--	'--	Squamous	'--	no	No	not repor
'--	'--	'--	'--	'--	'--	'--	'--	'--	'--	'--	Squamous	'--	no	No	not repor
'--	'--	'--	'--	'--	'--	'--	'--	'--	'--	'--	Squamous	'--	no	No	not repor
'--	'--	'--	'--	'--	'--	'--	'--	'--	'--	'--	Squamous	'--	no	No	not repor
'--	'--	'--	'--	'--	'--	'--	'--	'--	'--	'--	Squamous	'--	no	No	not repor
'--	'--	'--	'--	'--	'--	'--	'--	'--	'--	'--	Squamous	'--	no	No	not repor
'--	'--	'--	'--	'--	'--	'--	'--	'--	'--	'--	Squamous	'--	no	No	not repor
'--	'--	'--	'--	'--	'--	'--	'--	'--	'--	'--	Squamous	'--	no	No	not repor
'--	'--	'--	'--	'--	'--	'--	'--	'--	'--	'--	Squamous	'--	no	No	not repor
'--	'--	'--	'--	'--	'--	'--	'--	'--	'--	'--	Squamous	'--	no	No	not repor
'--	'--	'--	'--	'--	'--	'--	'--	'--	'--	'--	Squamous	'--	no	No	not repor
'--	'--	'--	'--	'--	'--	'--	'--	'--	'--	'--	Squamous	'--	no	No	not repor
'--	'--	'--	'--	'--	'--	'--	'--	'--	'--	'--	Squamous	'--	no	No	not repor
'--	'--	'--	'--	'--	'--	'--	'--	'--	'--	'--	Squamous	'--	no	No	not repor
'--	'--	'--	'--	'--	'--	'--	'--	'--	'--	'--	Squamous	'--	no	No	not repor
'--	'--	'--	'--	'--	'--	'--	'--	'--	'--	'--	Squamous	'--	no	No	not repor
'--	'--	'--	'--	'--	'--	'--	'--	'--	'--	'--	Squamous	'--	no	No	not repor
'--	'--	'--	'--	'--	'--	'--	'--	'--	'--	'--	Squamous	'--	no	No	not repor
'--	'--	'--	'--	'--	'--	'--	'--	'--	'--	'--	Squamous	'--	no	No	not repor

'--	'--	'--	'--	'--	'--	'--	'--	'--	'--	'--	Squamous	'--	no	No	not repor
'--	'--	'--	'--	'--	'--	'--	'--	'--	'--	'--	Squamous	'--	no	No	not repor
'--	'--	'--	'--	'--	'--	'--	'--	'--	'--	'--	Squamous	'--	no	No	not repor
'--	'--	'--	'--	'--	'--	'--	'--	'--	'--	'--	Squamous	'--	no	No	not repor
'--	'--	'--	'--	'--	'--	'--	'--	'--	'--	'--	Squamous	'--	no	No	not repor
'--	'--	'--	'--	'--	'--	'--	'--	'--	'--	'--	Squamous	'--	no	No	not repor
'--	'--	'--	'--	'--	'--	'--	'--	'--	'--	'--	Squamous	'--	no	No	not repor
'--	'--	'--	'--	'--	'--	'--	'--	'--	'--	'--	Squamous	'--	no	No	not repor
'--	'--	'--	'--	'--	'--	'--	'--	'--	'--	'--	Squamous	'--	no	No	not repor
'--	'--	'--	'--	'--	'--	'--	'--	'--	'--	'--	Squamous	'--	no	No	not repor
'--	'--	'--	'--	'--	'--	'--	'--	'--	'--	'--	Squamous	'--	no	No	not repor
'--	'--	'--	'--	'--	'--	'--	'--	'--	'--	'--	Squamous	'--	no	No	not repor
'--	'--	'--	'--	'--	'--	'--	'--	'--	'--	'--	Squamous	'--	no	No	not repor
'--	'--	'--	'--	'--	'--	'--	'--	'--	'--	'--	Squamous	'--	no	No	not repor
'--	'--	'--	'--	'--	'--	'--	'--	'--	'--	'--	Squamous	'--	yes	Yes	not repor
'--	'--	'--	'--	'--	'--	'--	'--	'--	'--	'--	Squamous	'--	yes	Yes	not repor
'--	'--	'--	'--	'--	'--	'--	'--	'--	'--	'--	Squamous	'--	no	No	not repor
'--	'--	'--	'--	'--	'--	'--	'--	'--	'--	'--	Squamous	'--	no	No	not repor
'--	'--	'--	'--	'--	'--	'--	'--	'--	'--	'--	Squamous	'--	no	No	not repor
'--	'--	'--	'--	'--	'--	'--	'--	'--	'--	'--	Squamous	'--	no	No	not repor
'--	'--	'--	'--	'--	'--	'--	'--	'--	'--	'--	Squamous	'--	no	No	not repor
'--	'--	'--	'--	'--	'--	'--	'--	'--	'--	'--	Squamous	'--	no	No	not repor
'--	'--	'--	'--	'--	'--	'--	'--	'--	'--	'--	Squamous	'--	no	No	not repor
'--	'--	'--	'--	'--	'--	'--	'--	'--	'--	'--	Squamous	'--	no	No	not repor
'--	'--	'--	'--	'--	'--	'--	'--	'--	'--	'--	Squamous	'--	no	No	not repor
'--	'--	'--	'--	'--	'--	'--	'--	'--	'--	'--	Squamous	'--	no	No	not repor
'--	'--	'--	'--	'--	'--	'--	'--	'--	'--	'--	Squamous	'--	yes	Yes	not repor
'--	'--	'--	'--	'--	'--	'--	'--	'--	'--	'--	Squamous	'--	yes	Yes	not repor
'--	'--	'--	'--	'--	'--	'--	'--	'--	'--	'--	Squamous	'--	no	No	not repor
'--	'--	'--	'--	'--	'--	'--	'--	'--	'--	'--	Squamous	'--	no	No	not repor
'--	'--	'--	'--	'--	'--	'--	'--	'--	'--	'--	Squamous	'--	yes	No	not repor
'--	'--	'--	'--	'--	'--	'--	'--	'--	'--	'--	Squamous	'--	yes	No	not repor
'--	'--	'--	'--	'--	'--	'--	'--	'--	'--	'--	Squamous	'--	no	No	not repor
'--	'--	'--	'--	'--	'--	'--	'--	'--	'--	'--	Squamous	'--	no	No	not repor
'--	'--	'--	'--	'--	'--	'--	'--	'--	'--	'--	Squamous	'--	no	No	not repor

'--	'--	'--	'--	'--	'--	'--	'--	'--	'--	'--	Squamous	'--	no	No	not repor
'--	'--	'--	'--	'--	'--	'--	'--	'--	'--	'--	Squamous	'--	no	No	not repor
'--	'--	'--	'--	'--	'--	'--	'--	'--	'--	'--	Squamous	'--	no	No	not repor
'--	'--	'--	'--	'--	'--	'--	'--	'--	'--	'--	Squamous	'--	no	No	not repor
'--	'--	'--	'--	'--	'--	'--	'--	'--	'--	'--	Squamous	'--	no	No	not repor
'--	'--	'--	'--	'--	'--	'--	'--	'--	'--	'--	Squamous	'--	no	No	not repor
'--	'--	'--	'--	'--	'--	'--	'--	'--	'--	'--	Squamous	'--	no	No	not repor
'--	'--	'--	'--	'--	'--	'--	'--	'--	'--	'--	Squamous	'--	no	No	not repor
'--	'--	'--	'--	'--	'--	'--	'--	'--	'--	'--	Squamous	'--	no	No	not repor
'--	'--	'--	'--	'--	'--	'--	'--	'--	'--	'--	Squamous	'--	no	No	not repor
'--	'--	'--	'--	'--	'--	'--	'--	'--	'--	'--	Squamous	'--	no	No	not repor
'--	'--	'--	'--	'--	'--	'--	'--	'--	'--	'--	Squamous	'--	no	No	not repor
'--	'--	'--	'--	'--	'--	'--	'--	'--	'--	'--	Squamous	'--	no	No	not repor
'--	'--	'--	'--	'--	'--	'--	'--	'--	'--	'--	Squamous	'--	no	No	not repor
'--	'--	'--	'--	'--	'--	'--	'--	'--	'--	'--	Squamous	'--	no	No	not repor
'--	'--	'--	'--	'--	'--	'--	'--	'--	'--	'--	Squamous	'--	no	No	not repor
'--	'--	'--	'--	'--	'--	'--	'--	'--	'--	'--	Squamous	'--	no	No	not repor
'--	'--	'--	'--	'--	'--	'--	'--	'--	'--	'--	Squamous	'--	no	No	not repor
'--	'--	'--	'--	'--	'--	'--	'--	'--	'--	'--	Squamous	'--	no	No	not repor
'--	'--	'--	'--	'--	'--	'--	'--	'--	'--	'--	Squamous	'--	no	No	not repor
'--	'--	'--	'--	'--	'--	'--	'--	'--	'--	'--	Squamous	'--	no	No	not repor
'--	'--	'--	'--	'--	'--	'--	'--	'--	'--	'--	Squamous	'--	no	No	not repor
'--	'--	'--	'--	'--	'--	'--	'--	'--	'--	'--	Squamous	'--	no	No	not repor
'--	'--	'--	'--	'--	'--	'--	'--	'--	'--	'--	Squamous	'--	no	No	not repor
'--	'--	'--	'--	'--	'--	'--	'--	'--	'--	'--	Squamous	'--	no	No	not repor
'--	'--	'--	'--	'--	'--	'--	'--	'--	'--	'--	Squamous	'--	no	No	not repor
'--	'--	'--	'--	'--	'--	'--	'--	'--	'--	'--	Squamous	'--	no	No	not repor
'--	'--	'--	'--	'--	'--	'--	'--	'--	'--	'--	Squamous	'--	no	No	not repor
'--	'--	'--	'--	'--	'--	'--	'--	'--	'--	'--	Squamous	'--	no	No	not repor
'--	'--	'--	'--	'--	'--	'--	'--	'--	'--	'--	Squamous	'--	no	No	not repor
'--	'--	'--	'--	'--	'--	'--	'--	'--	'--	'--	Squamous	'--	no	No	not repor
'--	'--	'--	'--	'--	'--	'--	'--	'--	'--	'--	Squamous	'--	no	No	not repor
'--	'--	'--	'--	'--	'--	'--	'--	'--	'--	'--	Squamous	'--	no	No	not repor
'--	'--	'--	'--	'--	'--	'--	'--	'--	'--	'--	Squamous	'--	yes	No	not repor
'--	'--	'--	'--	'--	'--	'--	'--	'--	'--	'--	Squamous	'--	yes	No	not repor

'--	'--	'--	'--	'--	'--	'--	'--	'--	'--	'--	Squamous	'--	no	No	not repor
'--	'--	'--	'--	'--	'--	'--	'--	'--	'--	'--	Squamous	'--	no	No	not repor
'--	'--	'--	'--	'--	'--	'--	'--	'--	'--	'--	Squamous	'--	no	No	not repor
'--	'--	'--	'--	'--	'--	'--	'--	'--	'--	'--	Squamous	'--	no	No	not repor
'--	'--	'--	'--	'--	'--	'--	'--	'--	'--	'--	Squamous	'--	no	No	not repor
'--	'--	'--	'--	'--	'--	'--	'--	'--	'--	'--	Squamous	'--	no	No	not repor
'--	'--	'--	'--	'--	'--	'--	'--	'--	'--	'--	Squamous	'--	no	No	not repor
'--	'--	'--	'--	'--	'--	'--	'--	'--	'--	'--	Squamous	'--	no	No	not repor
'--	'--	'--	'--	'--	'--	'--	'--	'--	'--	'--	Squamous	'--	no	No	not repor
'--	'--	'--	'--	'--	'--	'--	'--	'--	'--	'--	Squamous	'--	no	No	not repor

residual_	satellite	secondary	site_of_r	sites_of_	supratent	synchrono	tissue_or	transglot	tumor_cor	tumor_dep	tumor_foc	tumor_gra	tumor_lar	tumor_reg	tumor_sta
'--	'--	'--	Border of	'--	'--	No	Border of	'--	'--	'--	'--	not repor	'--	'--	stage ii
'--	'--	'--	Border of	'--	'--	No	Border of	'--	'--	'--	'--	not repor	'--	'--	stage ii
'--	'--	'--	Tongue, M	'--	'--	No	Tongue, M	'--	'--	'--	'--	not repor	'--	'--	stage iva
'--	'--	'--	Tongue, M	'--	'--	No	Tongue, M	'--	'--	'--	'--	not repor	'--	'--	stage iva
'--	'--	'--	Tongue, M	'--	'--	No	Tongue, M	'--	'--	'--	'--	not repor	'--	'--	stage i
'--	'--	'--	Tongue, M	'--	'--	No	Tongue, M	'--	'--	'--	'--	not repor	'--	'--	stage i
'--	'--	'--	Tongue, M	'--	'--	No	Tongue, M	'--	'--	'--	'--	not repor	'--	'--	stage iva
'--	'--	'--	Tongue, M	'--	'--	No	Tongue, M	'--	'--	'--	'--	not repor	'--	'--	stage iva
'--	'--	'--	Tongue, M	'--	'--	No	Tongue, M	'--	'--	'--	'--	not repor	'--	'--	stage ii
'--	'--	'--	Tongue, M	'--	'--	No	Tongue, M	'--	'--	'--	'--	not repor	'--	'--	stage ii
'--	'--	'--	Tongue, M	'--	'--	No	Tongue, M	'--	'--	'--	'--	not repor	'--	'--	not repor
'--	'--	'--	Tongue, M	'--	'--	No	Tongue, M	'--	'--	'--	'--	not repor	'--	'--	not repor
'--	'--	'--	Tongue, M	'--	'--	No	Tongue, M	'--	'--	'--	'--	not repor	'--	'--	stage iii
'--	'--	'--	Tongue, M	'--	'--	No	Tongue, M	'--	'--	'--	'--	not repor	'--	'--	stage iii
'--	'--	'--	Tongue, M	'--	'--	No	Tongue, M	'--	'--	'--	'--	not repor	'--	'--	stage iii
'--	'--	'--	Tongue, M	'--	'--	No	Tongue, M	'--	'--	'--	'--	not repor	'--	'--	stage iii
'--	'--	'--	Tongue, M	'--	'--	No	Tongue, M	'--	'--	'--	'--	not repor	'--	'--	stage ii
'--	'--	'--	Tongue, M	'--	'--	No	Tongue, M	'--	'--	'--	'--	not repor	'--	'--	stage ii
'--	'--	'--	Tongue, M	'--	'--	Not Repor	Tongue, M	'--	'--	'--	'--	not repor	'--	'--	not repor
'--	'--	'--	Tongue, M	'--	'--	Not Repor	Tongue, M	'--	'--	'--	'--	not repor	'--	'--	not repor
'--	'--	'--	Tongue, M	'--	'--	No	Tongue, M	'--	'--	'--	'--	not repor	'--	'--	not repor
'--	'--	'--	Tongue, M	'--	'--	No	Tongue, M	'--	'--	'--	'--	not repor	'--	'--	not repor
'--	'--	'--	Tongue, M	'--	'--	No	Tongue, M	'--	'--	'--	'--	not repor	'--	'--	not repor
'--	'--	'--	Tongue, M	'--	'--	No	Tongue, M	'--	'--	'--	'--	not repor	'--	'--	not repor
'--	'--	'--	Tongue, M	'--	'--	No	Tongue, M	'--	'--	'--	'--	not repor	'--	'--	stage iii
'--	'--	'--	Tongue, M	'--	'--	No	Tongue, M	'--	'--	'--	'--	not repor	'--	'--	stage iii
'--	'--	'--	Tongue, M	'--	'--	No	Tongue, M	'--	'--	'--	'--	not repor	'--	'--	stage iii
'--	'--	'--	Tongue, M	'--	'--	No	Tongue, M	'--	'--	'--	'--	not repor	'--	'--	stage iii
'--	'--	'--	Tongue, M	'--	'--	No	Tongue, M	'--	'--	'--	'--	not repor	'--	'--	stage iva
'--	'--	'--	Tongue, M	'--	'--	No	Tongue, M	'--	'--	'--	'--	not repor	'--	'--	stage iva
'--	'--	'--	Tongue, M	'--	'--	No	Tongue, M	'--	'--	'--	'--	not repor	'--	'--	stage i
'--	'--	'--	Tongue, M	'--	'--	No	Tongue, M	'--	'--	'--	'--	not repor	'--	'--	stage i
'--	'--	'--	Tongue, M	'--	'--	No	Tongue, M	'--	'--	'--	'--	not repor	'--	'--	not repor
'--	'--	'--	Tongue, M	'--	'--	No	Tongue, M	'--	'--	'--	'--	not repor	'--	'--	not repor

'--	'--	'--	Tongue, N	'--	'--	No	Tongue, N	'--	'--	'--	'--	not repor	'--	'--	stage ii
'--	'--	'--	Tongue, N	'--	'--	No	Tongue, N	'--	'--	'--	'--	not repor	'--	'--	stage ii
'--	'--	'--	Tongue, N	'--	'--	No	Tongue, N	'--	'--	'--	'--	not repor	'--	'--	stage ii
'--	'--	'--	Tongue, N	'--	'--	No	Tongue, N	'--	'--	'--	'--	not repor	'--	'--	stage ii
'--	'--	'--	Tongue, N	'--	'--	No	Tongue, N	'--	'--	'--	'--	not repor	'--	'--	stage iva
'--	'--	'--	Tongue, N	'--	'--	No	Tongue, N	'--	'--	'--	'--	not repor	'--	'--	stage iva
'--	'--	'--	Tongue, N	'--	'--	No	Tongue, N	'--	'--	'--	'--	not repor	'--	'--	stage iva
'--	'--	'--	Tongue, N	'--	'--	No	Tongue, N	'--	'--	'--	'--	not repor	'--	'--	stage iva
'--	'--	'--	Tongue, N	'--	'--	No	Tongue, N	'--	'--	'--	'--	not repor	'--	'--	stage iva
'--	'--	'--	Tongue, N	'--	'--	No	Tongue, N	'--	'--	'--	'--	not repor	'--	'--	stage iva
'--	'--	'--	Tongue, N	'--	'--	No	Tongue, N	'--	'--	'--	'--	not repor	'--	'--	not repor
'--	'--	'--	Tongue, N	'--	'--	No	Tongue, N	'--	'--	'--	'--	not repor	'--	'--	not repor
'--	'--	'--	Tongue, N	'--	'--	No	Tongue, N	'--	'--	'--	'--	not repor	'--	'--	stage iva
'--	'--	'--	Tongue, N	'--	'--	No	Tongue, N	'--	'--	'--	'--	not repor	'--	'--	stage iva
'--	'--	'--	Tongue, N	'--	'--	No	Tongue, N	'--	'--	'--	'--	not repor	'--	'--	stage iva
'--	'--	'--	Tongue, N	'--	'--	No	Tongue, N	'--	'--	'--	'--	not repor	'--	'--	stage iva
'--	'--	'--	Tongue, N	'--	'--	No	Tongue, N	'--	'--	'--	'--	not repor	'--	'--	stage iva
'--	'--	'--	Tongue, N	'--	'--	No	Tongue, N	'--	'--	'--	'--	not repor	'--	'--	stage iva
'--	'--	'--	Tongue, N	'--	'--	No	Tongue, N	'--	'--	'--	'--	not repor	'--	'--	stage i
'--	'--	'--	Tongue, N	'--	'--	No	Tongue, N	'--	'--	'--	'--	not repor	'--	'--	stage i
'--	'--	'--	Tongue, N	'--	'--	No	Tongue, N	'--	'--	'--	'--	not repor	'--	'--	stage iva
'--	'--	'--	Tongue, N	'--	'--	No	Tongue, N	'--	'--	'--	'--	not repor	'--	'--	stage iva
'--	'--	'--	Tongue, N	'--	'--	No	Tongue, N	'--	'--	'--	'--	not repor	'--	'--	stage ii
'--	'--	'--	Tongue, N	'--	'--	No	Tongue, N	'--	'--	'--	'--	not repor	'--	'--	stage ii
'--	'--	'--	Tongue, N	'--	'--	No	Tongue, N	'--	'--	'--	'--	not repor	'--	'--	stage iva
'--	'--	'--	Tongue, N	'--	'--	No	Tongue, N	'--	'--	'--	'--	not repor	'--	'--	stage iva
'--	'--	'--	Tongue, N	'--	'--	No	Tongue, N	'--	'--	'--	'--	not repor	'--	'--	stage iva
'--	'--	'--	Tongue, N	'--	'--	No	Tongue, N	'--	'--	'--	'--	not repor	'--	'--	stage iva
'--	'--	'--	Tongue, N	'--	'--	No	Tongue, N	'--	'--	'--	'--	not repor	'--	'--	stage iva
'--	'--	'--	Tongue, N	'--	'--	No	Tongue, N	'--	'--	'--	'--	not repor	'--	'--	stage iva
'--	'--	'--	Tongue, N	'--	'--	No	Tongue, N	'--	'--	'--	'--	not repor	'--	'--	stage ii
'--	'--	'--	Tongue, N	'--	'--	No	Tongue, N	'--	'--	'--	'--	not repor	'--	'--	stage ii
'--	'--	'--	Tongue, N	'--	'--	No	Tongue, N	'--	'--	'--	'--	not repor	'--	'--	stage ii
'--	'--	'--	Tongue, N	'--	'--	No	Tongue, N	'--	'--	'--	'--	not repor	'--	'--	stage ii
'--	'--	'--	Tongue, N	'--	'--	No	Tongue, N	'--	'--	'--	'--	not repor	'--	'--	stage iva

'--	'--	'--	Tongue, N	'--	'--	No	Tongue, N	'--	'--	'--	'--	not repor	'--	'--	stage iva
'--	'--	'--	Tongue, N	'--	'--	No	Tongue, N	'--	'--	'--	'--	not repor	'--	'--	stage iva
'--	'--	'--	Tongue, N	'--	'--	No	Tongue, N	'--	'--	'--	'--	not repor	'--	'--	stage iva
'--	'--	'--	Tongue, N	'--	'--	No	Tongue, N	'--	'--	'--	'--	not repor	'--	'--	not repor
'--	'--	'--	Tongue, N	'--	'--	No	Tongue, N	'--	'--	'--	'--	not repor	'--	'--	not repor
'--	'--	'--	Tongue, N	'--	'--	No	Tongue, N	'--	'--	'--	'--	not repor	'--	'--	stage ii
'--	'--	'--	Tongue, N	'--	'--	No	Tongue, N	'--	'--	'--	'--	not repor	'--	'--	stage ii
'--	'--	'--	Tongue, N	'--	'--	No	Tongue, N	'--	'--	'--	'--	not repor	'--	'--	stage iii
'--	'--	'--	Tongue, N	'--	'--	No	Tongue, N	'--	'--	'--	'--	not repor	'--	'--	stage iii
'--	'--	'--	Tongue, N	'--	'--	No	Tongue, N	'--	'--	'--	'--	not repor	'--	'--	stage ivb
'--	'--	'--	Tongue, N	'--	'--	No	Tongue, N	'--	'--	'--	'--	not repor	'--	'--	stage ivb
'--	'--	'--	Tongue, N	'--	'--	No	Tongue, N	'--	'--	'--	'--	not repor	'--	'--	stage iva
'--	'--	'--	Tongue, N	'--	'--	No	Tongue, N	'--	'--	'--	'--	not repor	'--	'--	stage iva
'--	'--	'--	Tongue, N	'--	'--	No	Tongue, N	'--	'--	'--	'--	not repor	'--	'--	stage iva
'--	'--	'--	Tongue, N	'--	'--	No	Tongue, N	'--	'--	'--	'--	not repor	'--	'--	stage iva
'--	'--	'--	Tongue, N	'--	'--	No	Tongue, N	'--	'--	'--	'--	not repor	'--	'--	stage iva
'--	'--	'--	Tongue, N	'--	'--	No	Tongue, N	'--	'--	'--	'--	not repor	'--	'--	stage iva
'--	'--	'--	Tongue, N	'--	'--	No	Tongue, N	'--	'--	'--	'--	not repor	'--	'--	stage iva
'--	'--	'--	Tongue, N	'--	'--	No	Tongue, N	'--	'--	'--	'--	not repor	'--	'--	stage iva
'--	'--	'--	Tongue, N	'--	'--	No	Tongue, N	'--	'--	'--	'--	not repor	'--	'--	stage iva
'--	'--	'--	Tongue, N	'--	'--	No	Tongue, N	'--	'--	'--	'--	not repor	'--	'--	stage iva
'--	'--	'--	Tongue, N	'--	'--	No	Tongue, N	'--	'--	'--	'--	not repor	'--	'--	stage iva
'--	'--	'--	Tongue, N	'--	'--	No	Tongue, N	'--	'--	'--	'--	not repor	'--	'--	stage iva
'--	'--	'--	Tongue, N	'--	'--	No	Tongue, N	'--	'--	'--	'--	not repor	'--	'--	not repor
'--	'--	'--	Tongue, N	'--	'--	No	Tongue, N	'--	'--	'--	'--	not repor	'--	'--	not repor
'--	'--	'--	Tongue, N	'--	'--	No	Tongue, N	'--	'--	'--	'--	not repor	'--	'--	stage iva
'--	'--	'--	Tongue, N	'--	'--	No	Tongue, N	'--	'--	'--	'--	not repor	'--	'--	stage iva
'--	'--	'--	Tongue, N	'--	'--	No	Tongue, N	'--	'--	'--	'--	not repor	'--	'--	stage iii
'--	'--	'--	Tongue, N	'--	'--	No	Tongue, N	'--	'--	'--	'--	not repor	'--	'--	stage iii
'--	'--	'--	Tongue, N	'--	'--	No	Tongue, N	'--	'--	'--	'--	not repor	'--	'--	stage iva
'--	'--	'--	Tongue, N	'--	'--	No	Tongue, N	'--	'--	'--	'--	not repor	'--	'--	stage iva
'--	'--	'--	Tongue, N	'--	'--	No	Tongue, N	'--	'--	'--	'--	not repor	'--	'--	stage iii
'--	'--	'--	Tongue, N	'--	'--	No	Tongue, N	'--	'--	'--	'--	not repor	'--	'--	stage iii
'--	'--	'--	Tongue, N	'--	'--	No	Tongue, N	'--	'--	'--	'--	not repor	'--	'--	stage iii
'--	'--	'--	Tongue, N	'--	'--	No	Tongue, N	'--	'--	'--	'--	not repor	'--	'--	stage iii

'--	'--	'--	Tongue, N	'--	'--	No	Tongue, N	'--	'--	'--	'--	not repor	'--	'--	stage iva
'--	'--	'--	Tongue, N	'--	'--	No	Tongue, N	'--	'--	'--	'--	not repor	'--	'--	stage iva
'--	'--	'--	Tongue, N	'--	'--	No	Tongue, N	'--	'--	'--	'--	not repor	'--	'--	stage ii
'--	'--	'--	Tongue, N	'--	'--	No	Tongue, N	'--	'--	'--	'--	not repor	'--	'--	stage ii
'--	'--	'--	Tongue, N	'--	'--	No	Tongue, N	'--	'--	'--	'--	not repor	'--	'--	stage iva
'--	'--	'--	Tongue, N	'--	'--	No	Tongue, N	'--	'--	'--	'--	not repor	'--	'--	stage iva
'--	'--	'--	Tongue, N	'--	'--	No	Tongue, N	'--	'--	'--	'--	not repor	'--	'--	not repor
'--	'--	'--	Tongue, N	'--	'--	No	Tongue, N	'--	'--	'--	'--	not repor	'--	'--	not repor
'--	'--	'--	Ventral s	'--	'--	No	Ventral s	'--	'--	'--	'--	not repor	'--	'--	stage ii
'--	'--	'--	Ventral s	'--	'--	No	Ventral s	'--	'--	'--	'--	not repor	'--	'--	stage ii
'--	'--	'--	Tongue, N	'--	'--	No	Tongue, N	'--	'--	'--	'--	not repor	'--	'--	stage ii
'--	'--	'--	Tongue, N	'--	'--	No	Tongue, N	'--	'--	'--	'--	not repor	'--	'--	stage ii
'--	'--	'--	Tongue, N	'--	'--	No	Tongue, N	'--	'--	'--	'--	not repor	'--	'--	stage iva
'--	'--	'--	Tongue, N	'--	'--	No	Tongue, N	'--	'--	'--	'--	not repor	'--	'--	stage iva
'--	'--	'--	Tongue, N	'--	'--	No	Tongue, N	'--	'--	'--	'--	not repor	'--	'--	stage iii
'--	'--	'--	Tongue, N	'--	'--	No	Tongue, N	'--	'--	'--	'--	not repor	'--	'--	stage iii
'--	'--	'--	Tongue, N	'--	'--	No	Tongue, N	'--	'--	'--	'--	not repor	'--	'--	stage i
'--	'--	'--	Tongue, N	'--	'--	No	Tongue, N	'--	'--	'--	'--	not repor	'--	'--	stage i
'--	'--	'--	Tongue, N	'--	'--	No	Tongue, N	'--	'--	'--	'--	not repor	'--	'--	stage i
'--	'--	'--	Tongue, N	'--	'--	No	Tongue, N	'--	'--	'--	'--	not repor	'--	'--	stage i
'--	'--	'--	Tongue, N	'--	'--	No	Tongue, N	'--	'--	'--	'--	not repor	'--	'--	stage i
'--	'--	'--	Tongue, N	'--	'--	No	Tongue, N	'--	'--	'--	'--	not repor	'--	'--	stage i
'--	'--	'--	Tongue, N	'--	'--	No	Tongue, N	'--	'--	'--	'--	not repor	'--	'--	stage ivb
'--	'--	'--	Tongue, N	'--	'--	No	Tongue, N	'--	'--	'--	'--	not repor	'--	'--	stage ivb
'--	'--	'--	Tongue, N	'--	'--	No	Tongue, N	'--	'--	'--	'--	not repor	'--	'--	stage iva
'--	'--	'--	Tongue, N	'--	'--	No	Tongue, N	'--	'--	'--	'--	not repor	'--	'--	stage iva
'--	'--	'--	Tongue, N	'--	'--	No	Tongue, N	'--	'--	'--	'--	not repor	'--	'--	stage iii
'--	'--	'--	Tongue, N	'--	'--	No	Tongue, N	'--	'--	'--	'--	not repor	'--	'--	stage iii
'--	'--	'--	Tongue, N	'--	'--	No	Tongue, N	'--	'--	'--	'--	not repor	'--	'--	stage iva
'--	'--	'--	Tongue, N	'--	'--	No	Tongue, N	'--	'--	'--	'--	not repor	'--	'--	stage iva
'--	'--	'--	Tongue, N	'--	'--	No	Tongue, N	'--	'--	'--	'--	not repor	'--	'--	stage iva
'--	'--	'--	Tongue, N	'--	'--	No	Tongue, N	'--	'--	'--	'--	not repor	'--	'--	stage iva
'--	'--	'--	Tongue, N	'--	'--	No	Tongue, N	'--	'--	'--	'--	not repor	'--	'--	stage iii
'--	'--	'--	Tongue, N	'--	'--	No	Tongue, N	'--	'--	'--	'--	not repor	'--	'--	stage iii
'--	'--	'--	Tongue, N	'--	'--	No	Tongue, N	'--	'--	'--	'--	not repor	'--	'--	stage iii

'--	'--	'--	Tongue, N	'--	'--	No	Tongue, N	'--	'--	'--	'--	not repor	'--	'--	stage iii
'--	'--	'--	Tongue, N	'--	'--	No	Tongue, N	'--	'--	'--	'--	not repor	'--	'--	stage i
'--	'--	'--	Tongue, N	'--	'--	No	Tongue, N	'--	'--	'--	'--	not repor	'--	'--	stage i
'--	'--	'--	Tongue, N	'--	'--	No	Tongue, N	'--	'--	'--	'--	not repor	'--	'--	stage ii
'--	'--	'--	Tongue, N	'--	'--	No	Tongue, N	'--	'--	'--	'--	not repor	'--	'--	stage ii
'--	'--	'--	Tongue, N	'--	'--	No	Tongue, N	'--	'--	'--	'--	not repor	'--	'--	stage iii
'--	'--	'--	Tongue, N	'--	'--	No	Tongue, N	'--	'--	'--	'--	not repor	'--	'--	stage iii
'--	'--	'--	Tongue, N	'--	'--	No	Tongue, N	'--	'--	'--	'--	not repor	'--	'--	stage ii
'--	'--	'--	Tongue, N	'--	'--	No	Tongue, N	'--	'--	'--	'--	not repor	'--	'--	stage ii
'--	'--	'--	Tongue, N	'--	'--	No	Tongue, N	'--	'--	'--	'--	not repor	'--	'--	stage iva
'--	'--	'--	Tongue, N	'--	'--	No	Tongue, N	'--	'--	'--	'--	not repor	'--	'--	stage iva
'--	'--	'--	Tongue, N	'--	'--	No	Tongue, N	'--	'--	'--	'--	not repor	'--	'--	stage iva
'--	'--	'--	Tongue, N	'--	'--	No	Tongue, N	'--	'--	'--	'--	not repor	'--	'--	stage iva
'--	'--	'--	Tongue, N	'--	'--	No	Tongue, N	'--	'--	'--	'--	not repor	'--	'--	stage iva
'--	'--	'--	Tongue, N	'--	'--	No	Tongue, N	'--	'--	'--	'--	not repor	'--	'--	stage iva
'--	'--	'--	Tongue, N	'--	'--	No	Tongue, N	'--	'--	'--	'--	not repor	'--	'--	stage i
'--	'--	'--	Tongue, N	'--	'--	No	Tongue, N	'--	'--	'--	'--	not repor	'--	'--	stage i
'--	'--	'--	Tongue, N	'--	'--	No	Tongue, N	'--	'--	'--	'--	not repor	'--	'--	stage iva
'--	'--	'--	Tongue, N	'--	'--	No	Tongue, N	'--	'--	'--	'--	not repor	'--	'--	stage iva
'--	'--	'--	Tongue, N	'--	'--	No	Tongue, N	'--	'--	'--	'--	not repor	'--	'--	stage iii
'--	'--	'--	Tongue, N	'--	'--	No	Tongue, N	'--	'--	'--	'--	not repor	'--	'--	stage iii
'--	'--	'--	Tongue, N	'--	'--	No	Tongue, N	'--	'--	'--	'--	not repor	'--	'--	stage iii
'--	'--	'--	Tongue, N	'--	'--	No	Tongue, N	'--	'--	'--	'--	not repor	'--	'--	stage iii
'--	'--	'--	Tongue, N	'--	'--	No	Tongue, N	'--	'--	'--	'--	not repor	'--	'--	stage ii
'--	'--	'--	Tongue, N	'--	'--	No	Tongue, N	'--	'--	'--	'--	not repor	'--	'--	stage ii
'--	'--	'--	Tongue, N	'--	'--	No	Tongue, N	'--	'--	'--	'--	not repor	'--	'--	not repor
'--	'--	'--	Tongue, N	'--	'--	No	Tongue, N	'--	'--	'--	'--	not repor	'--	'--	not repor
'--	'--	'--	Tongue, N	'--	'--	No	Tongue, N	'--	'--	'--	'--	not repor	'--	'--	stage iii
'--	'--	'--	Tongue, N	'--	'--	No	Tongue, N	'--	'--	'--	'--	not repor	'--	'--	stage iii
'--	'--	'--	Tongue, N	'--	'--	No	Tongue, N	'--	'--	'--	'--	not repor	'--	'--	stage iva
'--	'--	'--	Tongue, N	'--	'--	No	Tongue, N	'--	'--	'--	'--	not repor	'--	'--	stage iva
'--	'--	'--	Tongue, N	'--	'--	No	Tongue, N	'--	'--	'--	'--	not repor	'--	'--	stage iva
'--	'--	'--	Tongue, N	'--	'--	No	Tongue, N	'--	'--	'--	'--	not repor	'--	'--	stage iva
'--	'--	'--	Tongue, N	'--	'--	No	Tongue, N	'--	'--	'--	'--	not repor	'--	'--	stage iii
'--	'--	'--	Tongue, N	'--	'--	No	Tongue, N	'--	'--	'--	'--	not repor	'--	'--	stage iii

'--	'--	'--	Tongue, N	'--	'--	No	Tongue, N	'--	'--	'--	'--	not repor	'--	'--	stage iii
'--	'--	'--	Tongue, N	'--	'--	No	Tongue, N	'--	'--	'--	'--	not repor	'--	'--	stage iii
'--	'--	'--	Tongue, N	'--	'--	No	Tongue, N	'--	'--	'--	'--	not repor	'--	'--	stage iii
'--	'--	'--	Tongue, N	'--	'--	No	Tongue, N	'--	'--	'--	'--	not repor	'--	'--	stage iii
'--	'--	'--	Tongue, N	'--	'--	No	Tongue, N	'--	'--	'--	'--	not repor	'--	'--	stage ii
'--	'--	'--	Tongue, N	'--	'--	No	Tongue, N	'--	'--	'--	'--	not repor	'--	'--	stage ii
'--	'--	'--	Tongue, N	'--	'--	No	Tongue, N	'--	'--	'--	'--	not repor	'--	'--	stage ii
'--	'--	'--	Tongue, N	'--	'--	No	Tongue, N	'--	'--	'--	'--	not repor	'--	'--	stage ii
'--	'--	'--	Tongue, N	'--	'--	No	Tongue, N	'--	'--	'--	'--	not repor	'--	'--	stage ii
'--	'--	'--	Tongue, N	'--	'--	No	Tongue, N	'--	'--	'--	'--	not repor	'--	'--	stage ii
'--	'--	'--	Tongue, N	'--	'--	No	Tongue, N	'--	'--	'--	'--	not repor	'--	'--	stage iva
'--	'--	'--	Tongue, N	'--	'--	No	Tongue, N	'--	'--	'--	'--	not repor	'--	'--	stage iva
'--	'--	'--	Tongue, N	'--	'--	No	Tongue, N	'--	'--	'--	'--	not repor	'--	'--	stage ii
'--	'--	'--	Tongue, N	'--	'--	No	Tongue, N	'--	'--	'--	'--	not repor	'--	'--	stage ii
'--	'--	'--	Tongue, N	'--	'--	Not Repor	Tongue, N	'--	'--	'--	'--	not repor	'--	'--	stage iva
'--	'--	'--	Tongue, N	'--	'--	Not Repor	Tongue, N	'--	'--	'--	'--	not repor	'--	'--	stage iva
'--	'--	'--	Tongue, N	'--	'--	No	Tongue, N	'--	'--	'--	'--	not repor	'--	'--	stage ii
'--	'--	'--	Tongue, N	'--	'--	No	Tongue, N	'--	'--	'--	'--	not repor	'--	'--	stage ii
'--	'--	'--	Tongue, N	'--	'--	No	Tongue, N	'--	'--	'--	'--	not repor	'--	'--	stage iii
'--	'--	'--	Tongue, N	'--	'--	No	Tongue, N	'--	'--	'--	'--	not repor	'--	'--	stage iii
'--	'--	'--	Tongue, N	'--	'--	No	Tongue, N	'--	'--	'--	'--	not repor	'--	'--	stage iva
'--	'--	'--	Tongue, N	'--	'--	No	Tongue, N	'--	'--	'--	'--	not repor	'--	'--	stage iva
'--	'--	'--	Tongue, N	'--	'--	No	Tongue, N	'--	'--	'--	'--	not repor	'--	'--	stage i
'--	'--	'--	Tongue, N	'--	'--	No	Tongue, N	'--	'--	'--	'--	not repor	'--	'--	stage i
'--	'--	'--	Tongue, N	'--	'--	No	Tongue, N	'--	'--	'--	'--	not repor	'--	'--	stage iva
'--	'--	'--	Tongue, N	'--	'--	No	Tongue, N	'--	'--	'--	'--	not repor	'--	'--	stage iva
'--	'--	'--	Tongue, N	'--	'--	Not Repor	Tongue, N	'--	'--	'--	'--	not repor	'--	'--	stage iii
'--	'--	'--	Tongue, N	'--	'--	Not Repor	Tongue, N	'--	'--	'--	'--	not repor	'--	'--	stage iii
'--	'--	'--	Tongue, N	'--	'--	No	Tongue, N	'--	'--	'--	'--	not repor	'--	'--	stage iva
'--	'--	'--	Tongue, N	'--	'--	No	Tongue, N	'--	'--	'--	'--	not repor	'--	'--	stage iva
'--	'--	'--	Tongue, N	'--	'--	No	Tongue, N	'--	'--	'--	'--	not repor	'--	'--	stage iii
'--	'--	'--	Tongue, N	'--	'--	No	Tongue, N	'--	'--	'--	'--	not repor	'--	'--	stage iii
'--	'--	'--	Tongue, N	'--	'--	No	Tongue, N	'--	'--	'--	'--	not repor	'--	'--	stage iii
'--	'--	'--	Tongue, N	'--	'--	No	Tongue, N	'--	'--	'--	'--	not repor	'--	'--	stage iii
'--	'--	'--	Tongue, N	'--	'--	No	Tongue, N	'--	'--	'--	'--	not repor	'--	'--	stage iva

'--	'--	'--	Tongue, N	'--	'--	No	Tongue, N	'--	'--	'--	'--	not repor	'--	'--	stage iva
'--	'--	'--	Tongue, N	'--	'--	No	Tongue, N	'--	'--	'--	'--	not repor	'--	'--	stage iva
'--	'--	'--	Tongue, N	'--	'--	No	Tongue, N	'--	'--	'--	'--	not repor	'--	'--	stage iva
'--	'--	'--	Tongue, N	'--	'--	No	Tongue, N	'--	'--	'--	'--	not repor	'--	'--	stage iii
'--	'--	'--	Tongue, N	'--	'--	No	Tongue, N	'--	'--	'--	'--	not repor	'--	'--	stage iii
'--	'--	'--	Tongue, N	'--	'--	No	Tongue, N	'--	'--	'--	'--	not repor	'--	'--	stage iva
'--	'--	'--	Tongue, N	'--	'--	No	Tongue, N	'--	'--	'--	'--	not repor	'--	'--	stage iva
'--	'--	'--	Tongue, N	'--	'--	No	Tongue, N	'--	'--	'--	'--	not repor	'--	'--	stage iva
'--	'--	'--	Tongue, N	'--	'--	No	Tongue, N	'--	'--	'--	'--	not repor	'--	'--	stage iva
'--	'--	'--	Tongue, N	'--	'--	No	Tongue, N	'--	'--	'--	'--	not repor	'--	'--	stage iii
'--	'--	'--	Tongue, N	'--	'--	No	Tongue, N	'--	'--	'--	'--	not repor	'--	'--	stage iii
'--	'--	'--	Tongue, N	'--	'--	No	Tongue, N	'--	'--	'--	'--	not repor	'--	'--	stage iva
'--	'--	'--	Tongue, N	'--	'--	No	Tongue, N	'--	'--	'--	'--	not repor	'--	'--	stage iva
'--	'--	'--	Tongue, N	'--	'--	No	Tongue, N	'--	'--	'--	'--	not repor	'--	'--	stage i
'--	'--	'--	Tongue, N	'--	'--	No	Tongue, N	'--	'--	'--	'--	not repor	'--	'--	stage i
'--	'--	'--	Tongue, N	'--	'--	No	Tongue, N	'--	'--	'--	'--	not repor	'--	'--	stage iii
'--	'--	'--	Tongue, N	'--	'--	No	Tongue, N	'--	'--	'--	'--	not repor	'--	'--	stage iii
'--	'--	'--	Tongue, N	'--	'--	No	Tongue, N	'--	'--	'--	'--	not repor	'--	'--	stage iva
'--	'--	'--	Tongue, N	'--	'--	No	Tongue, N	'--	'--	'--	'--	not repor	'--	'--	stage iva
'--	'--	'--	Tongue, N	'--	'--	No	Tongue, N	'--	'--	'--	'--	not repor	'--	'--	stage iva
'--	'--	'--	Tongue, N	'--	'--	No	Tongue, N	'--	'--	'--	'--	not repor	'--	'--	stage iva
'--	'--	'--	Tongue, N	'--	'--	No	Tongue, N	'--	'--	'--	'--	not repor	'--	'--	stage iva
'--	'--	'--	Tongue, N	'--	'--	No	Tongue, N	'--	'--	'--	'--	not repor	'--	'--	stage iva
'--	'--	'--	Tongue, N	'--	'--	No	Tongue, N	'--	'--	'--	'--	not repor	'--	'--	stage iva
'--	'--	'--	Tongue, N	'--	'--	No	Tongue, N	'--	'--	'--	'--	not repor	'--	'--	stage iva
'--	'--	'--	Tongue, N	'--	'--	No	Tongue, N	'--	'--	'--	'--	not repor	'--	'--	stage iva
'--	'--	'--	Tongue, N	'--	'--	No	Tongue, N	'--	'--	'--	'--	not repor	'--	'--	stage iva
'--	'--	'--	Tongue, N	'--	'--	No	Tongue, N	'--	'--	'--	'--	not repor	'--	'--	stage iii
'--	'--	'--	Tongue, N	'--	'--	No	Tongue, N	'--	'--	'--	'--	not repor	'--	'--	stage iii
'--	'--	'--	Tongue, N	'--	'--	No	Tongue, N	'--	'--	'--	'--	not repor	'--	'--	stage iva
'--	'--	'--	Tongue, N	'--	'--	No	Tongue, N	'--	'--	'--	'--	not repor	'--	'--	stage iva
'--	'--	'--	Tongue, N	'--	'--	No	Tongue, N	'--	'--	'--	'--	not repor	'--	'--	stage i
'--	'--	'--	Tongue, N	'--	'--	No	Tongue, N	'--	'--	'--	'--	not repor	'--	'--	stage i
'--	'--	'--	Tongue, N	'--	'--	No	Tongue, N	'--	'--	'--	'--	not repor	'--	'--	stage i
'--	'--	'--	Tongue, N	'--	'--	No	Tongue, N	'--	'--	'--	'--	not repor	'--	'--	stage i

'--	'--	'--	Tongue, N	'--	'--	No	Tongue, N	'--	'--	'--	'--	not repor	'--	'--	stage iva
'--	'--	'--	Tongue, N	'--	'--	No	Tongue, N	'--	'--	'--	'--	not repor	'--	'--	stage iva
'--	'--	'--	Tongue, N	'--	'--	No	Tongue, N	'--	'--	'--	'--	not repor	'--	'--	stage ii
'--	'--	'--	Tongue, N	'--	'--	No	Tongue, N	'--	'--	'--	'--	not repor	'--	'--	stage ii
'--	'--	'--	Tongue, N	'--	'--	No	Tongue, N	'--	'--	'--	'--	not repor	'--	'--	stage iva
'--	'--	'--	Tongue, N	'--	'--	No	Tongue, N	'--	'--	'--	'--	not repor	'--	'--	stage iva
'--	'--	'--	Tongue, N	'--	'--	No	Tongue, N	'--	'--	'--	'--	not repor	'--	'--	stage iva
'--	'--	'--	Tongue, N	'--	'--	No	Tongue, N	'--	'--	'--	'--	not repor	'--	'--	stage iva
'--	'--	'--	Tongue, N	'--	'--	No	Tongue, N	'--	'--	'--	'--	not repor	'--	'--	stage i
'--	'--	'--	Tongue, N	'--	'--	No	Tongue, N	'--	'--	'--	'--	not repor	'--	'--	stage i

vascular_	vascular_	weiss_ass	who_cns_g	who_nte_g	wilms_tun	year_of_c	chemo_cor	days_to_t	days_to_t	initial_c	number_of	reason_tr	regimen_c	therapeut	treatment
'--	'--	'--	'--	'--	'--	2012	'--	'--	'--	'--	'--	'--	'--	'--	'--
'--	'--	'--	'--	'--	'--	2012	'--	'--	'--	'--	'--	'--	'--	'--	'--
'--	'--	'--	'--	'--	'--	2009	'--	'--	'--	'--	'--	'--	'--	'--	'--
'--	'--	'--	'--	'--	'--	2009	'--	'--	'--	'--	'--	'--	'--	'--	'--
'--	'--	'--	'--	'--	'--	2011	'--	'--	'--	'--	'--	'--	'--	'--	'--
'--	'--	'--	'--	'--	'--	2011	'--	'--	'--	'--	'--	'--	'--	'--	'--
'--	'--	'--	'--	'--	'--	2008	'--	'--	'--	'--	'--	'--	'--	'--	'--
'--	'--	'--	'--	'--	'--	2008	'--	'--	'--	'--	'--	'--	'--	'--	'--
'--	'--	'--	'--	'--	'--	2012	'--	'--	'--	'--	'--	'--	'--	'--	'--
'--	'--	'--	'--	'--	'--	2012	'--	'--	'--	'--	'--	'--	'--	'--	'--
'--	'--	'--	'--	'--	'--	2007	'--	'--	'--	'--	'--	'--	'--	'--	'--
'--	'--	'--	'--	'--	'--	2007	'--	'--	'--	'--	'--	'--	'--	'--	'--
'--	'--	'--	'--	'--	'--	2011	'--	'--	'--	'--	'--	'--	'--	'--	'--
'--	'--	'--	'--	'--	'--	2011	'--	'--	'--	'--	'--	'--	'--	'--	'--
'--	'--	'--	'--	'--	'--	1999	'--	'--	'--	'--	'--	'--	'--	'--	'--
'--	'--	'--	'--	'--	'--	1999	'--	'--	'--	'--	'--	'--	'--	'--	'--
'--	'--	'--	'--	'--	'--	1999	'--	'--	'--	'--	'--	'--	'--	'--	'--
'--	'--	'--	'--	'--	'--	1999	'--	'--	'--	'--	'--	'--	'--	'--	'--
'--	'--	'--	'--	'--	'--	2008	'--	'--	'--	'--	'--	'--	'--	'--	'--
'--	'--	'--	'--	'--	'--	2008	'--	'--	'--	'--	'--	'--	'--	'--	'--
'--	'--	'--	'--	'--	'--	2012	'--	'--	'--	'--	'--	'--	'--	'--	'--
'--	'--	'--	'--	'--	'--	2012	'--	'--	'--	'--	'--	'--	'--	'--	'--
'--	'--	'--	'--	'--	'--	2008	'--	'--	'--	'--	'--	'--	'--	'--	'--
'--	'--	'--	'--	'--	'--	2008	'--	'--	'--	'--	'--	'--	'--	'--	'--
'--	'--	'--	'--	'--	'--	2011	'--	'--	'--	'--	'--	'--	'--	'--	'--
'--	'--	'--	'--	'--	'--	2011	'--	'--	'--	'--	'--	'--	'--	'--	'--
'--	'--	'--	'--	'--	'--	2011	'--	'--	'--	'--	'--	'--	'--	'--	'--
'--	'--	'--	'--	'--	'--	2011	'--	'--	'--	'--	'--	'--	'--	'--	'--
'--	'--	'--	'--	'--	'--	2013	'--	'--	'--	'--	'--	'--	'--	'--	'--
'--	'--	'--	'--	'--	'--	2013	'--	'--	'--	'--	'--	'--	'--	'--	'--
'--	'--	'--	'--	'--	'--	2012	'--	'--	'--	'--	'--	'--	'--	'--	'--
'--	'--	'--	'--	'--	'--	2012	'--	'--	'--	'--	'--	'--	'--	'--	'--
'--	'--	'--	'--	'--	'--	2009	'--	'--	'--	'--	'--	'--	'--	'--	'--
'--	'--	'--	'--	'--	'--	2009	'--	'--	'--	'--	'--	'--	'--	'--	'--

’--	’--	’--	’--	’--	’--	2011 ’--	’--	’--	’--	’--	’--	’--	’--	’--
’--	’--	’--	’--	’--	’--	2011 ’--	’--	’--	’--	’--	’--	’--	’--	’--
’--	’--	’--	’--	’--	’--	2011 ’--	’--	’--	’--	’--	’--	’--	’--	’--
’--	’--	’--	’--	’--	’--	2011 ’--	’--	’--	’--	’--	’--	’--	’--	’--
’--	’--	’--	’--	’--	’--	1994 ’--	’--	’--	’--	’--	’--	’--	’--	’--
’--	’--	’--	’--	’--	’--	1994 ’--	’--	’--	’--	’--	’--	’--	’--	’--
’--	’--	’--	’--	’--	’--	2012 ’--	’--	’--	’--	’--	’--	’--	’--	’--
’--	’--	’--	’--	’--	’--	2012 ’--	’--	’--	’--	’--	’--	’--	’--	’--
’--	’--	’--	’--	’--	’--	2011 ’--	’--	’--	’--	’--	’--	’--	’--	’--
’--	’--	’--	’--	’--	’--	2011 ’--	’--	’--	’--	’--	’--	’--	’--	’--
’--	’--	’--	’--	’--	’--	1998 ’--	’--	’--	’--	’--	’--	’--	’--	’--
’--	’--	’--	’--	’--	’--	1998 ’--	’--	’--	’--	’--	’--	’--	’--	’--
’--	’--	’--	’--	’--	’--	2009 ’--	’--	’--	’--	’--	’--	’--	’--	’--
’--	’--	’--	’--	’--	’--	2009 ’--	’--	’--	’--	’--	’--	’--	’--	’--
’--	’--	’--	’--	’--	’--	2000 ’--	’--	’--	’--	’--	’--	’--	’--	’--
’--	’--	’--	’--	’--	’--	2000 ’--	’--	’--	’--	’--	’--	’--	’--	’--
’--	’--	’--	’--	’--	’--	1996 ’--	’--	’--	’--	’--	’--	’--	’--	’--
’--	’--	’--	’--	’--	’--	1996 ’--	’--	’--	’--	’--	’--	’--	’--	’--
’--	’--	’--	’--	’--	’--	2009 ’--	’--	’--	’--	’--	’--	’--	’--	’--
’--	’--	’--	’--	’--	’--	2009 ’--	’--	’--	’--	’--	’--	’--	’--	’--
’--	’--	’--	’--	’--	’--	2011 ’--	’--	’--	’--	’--	’--	’--	’--	’--
’--	’--	’--	’--	’--	’--	2011 ’--	’--	’--	’--	’--	’--	’--	’--	’--
’--	’--	’--	’--	’--	’--	2012 ’--	’--	’--	’--	’--	’--	’--	’--	’--
’--	’--	’--	’--	’--	’--	2012 ’--	’--	’--	’--	’--	’--	’--	’--	’--
’--	’--	’--	’--	’--	’--	2012 ’--	’--	’--	’--	’--	’--	’--	’--	’--
’--	’--	’--	’--	’--	’--	2012 ’--	’--	’--	’--	’--	’--	’--	’--	’--
’--	’--	’--	’--	’--	’--	2009 ’--	’--	’--	’--	’--	’--	’--	’--	’--
’--	’--	’--	’--	’--	’--	2009 ’--	’--	’--	’--	’--	’--	’--	’--	’--
’--	’--	’--	’--	’--	’--	2007 ’--	’--	’--	’--	’--	’--	’--	’--	’--
’--	’--	’--	’--	’--	’--	2007 ’--	’--	’--	’--	’--	’--	’--	’--	’--
’--	’--	’--	’--	’--	’--	2009 ’--	’--	’--	’--	’--	’--	’--	’--	’--
’--	’--	’--	’--	’--	’--	2009 ’--	’--	’--	’--	’--	’--	’--	’--	’--
’--	’--	’--	’--	’--	’--	2010 ’--	’--	’--	’--	’--	’--	’--	’--	’--
’--	’--	’--	’--	’--	’--	2010 ’--	’--	’--	’--	’--	’--	’--	’--	’--
’--	’--	’--	’--	’--	’--	2012 ’--	’--	’--	’--	’--	’--	’--	’--	’--

’--	’--	’--	’--	’--	’--	2012 ’--	’--	’--	’--	’--	’--	’--	’--	’--
’--	’--	’--	’--	’--	’--	1993 ’--	’--	’--	’--	’--	’--	’--	’--	’--
’--	’--	’--	’--	’--	’--	1993 ’--	’--	’--	’--	’--	’--	’--	’--	’--
’--	’--	’--	’--	’--	’--	2013 ’--	’--	’--	’--	’--	’--	’--	’--	’--
’--	’--	’--	’--	’--	’--	2013 ’--	’--	’--	’--	’--	’--	’--	’--	’--
’--	’--	’--	’--	’--	’--	2013 ’--	’--	’--	’--	’--	’--	’--	’--	’--
’--	’--	’--	’--	’--	’--	2013 ’--	’--	’--	’--	’--	’--	’--	’--	’--
’--	’--	’--	’--	’--	’--	1993 ’--	’--	’--	’--	’--	’--	’--	’--	’--
’--	’--	’--	’--	’--	’--	1993 ’--	’--	’--	’--	’--	’--	’--	’--	’--
’--	’--	’--	’--	’--	’--	2011 ’--	’--	’--	’--	’--	’--	’--	’--	’--
’--	’--	’--	’--	’--	’--	2011 ’--	’--	’--	’--	’--	’--	’--	’--	’--
’--	’--	’--	’--	’--	’--	2006 ’--	’--	’--	’--	’--	’--	’--	’--	’--
’--	’--	’--	’--	’--	’--	2006 ’--	’--	’--	’--	’--	’--	’--	’--	’--
’--	’--	’--	’--	’--	’--	2008 ’--	’--	’--	’--	’--	’--	’--	’--	’--
’--	’--	’--	’--	’--	’--	2008 ’--	’--	’--	’--	’--	’--	’--	’--	’--
’--	’--	’--	’--	’--	’--	2011 ’--	’--	’--	’--	’--	’--	’--	’--	’--
’--	’--	’--	’--	’--	’--	2011 ’--	’--	’--	’--	’--	’--	’--	’--	’--
’--	’--	’--	’--	’--	’--	1995 ’--	’--	’--	’--	’--	’--	’--	’--	’--
’--	’--	’--	’--	’--	’--	1995 ’--	’--	’--	’--	’--	’--	’--	’--	’--
’--	’--	’--	’--	’--	’--	2006 ’--	’--	’--	’--	’--	’--	’--	’--	’--
’--	’--	’--	’--	’--	’--	2006 ’--	’--	’--	’--	’--	’--	’--	’--	’--
’--	’--	’--	’--	’--	’--	2009 ’--	’--	’--	’--	’--	’--	’--	’--	’--
’--	’--	’--	’--	’--	’--	2009 ’--	’--	’--	’--	’--	’--	’--	’--	’--
’--	’--	’--	’--	’--	’--	2009 ’--	’--	’--	’--	’--	’--	’--	’--	’--
’--	’--	’--	’--	’--	’--	2009 ’--	’--	’--	’--	’--	’--	’--	’--	’--
’--	’--	’--	’--	’--	’--	2012 ’--	’--	’--	’--	’--	’--	’--	’--	’--
’--	’--	’--	’--	’--	’--	2012 ’--	’--	’--	’--	’--	’--	’--	’--	’--
’--	’--	’--	’--	’--	’--	1999 ’--	’--	’--	’--	’--	’--	’--	’--	’--
’--	’--	’--	’--	’--	’--	1999 ’--	’--	’--	’--	’--	’--	’--	’--	’--
’--	’--	’--	’--	’--	’--	2011 ’--	’--	’--	’--	’--	’--	’--	’--	’--
’--	’--	’--	’--	’--	’--	2011 ’--	’--	’--	’--	’--	’--	’--	’--	’--
’--	’--	’--	’--	’--	’--	1997 ’--	’--	’--	’--	’--	’--	’--	’--	’--
’--	’--	’--	’--	’--	’--	1997 ’--	’--	’--	’--	’--	’--	’--	’--	’--
’--	’--	’--	’--	’--	’--	2012 ’--	’--	’--	’--	’--	’--	’--	’--	’--
’--	’--	’--	’--	’--	’--	2012 ’--	’--	’--	’--	’--	’--	’--	’--	’--

’——	’——	’——	’——	’——	’——	2003 ’——	’——	’——	’——	’——	’——	’——	’——	’——
’——	’——	’——	’——	’——	’——	2003 ’——	’——	’——	’——	’——	’——	’——	’——	’——
’——	’——	’——	’——	’——	’——	2007 ’——	’——	’——	’——	’——	’——	’——	’——	’——
’——	’——	’——	’——	’——	’——	2007 ’——	’——	’——	’——	’——	’——	’——	’——	’——
’——	’——	’——	’——	’——	’——	2010 ’——	’——	’——	’——	’——	’——	’——	’——	’——
’——	’——	’——	’——	’——	’——	2010 ’——	’——	’——	’——	’——	’——	’——	’——	’——
’——	’——	’——	’——	’——	’——	2010 ’——	’——	’——	’——	’——	’——	’——	’——	’——
’——	’——	’——	’——	’——	’——	2010 ’——	’——	’——	’——	’——	’——	’——	’——	’——
’——	’——	’——	’——	’——	’——	2011 ’——	’——	’——	’——	’——	’——	’——	’——	’——
’——	’——	’——	’——	’——	’——	2011 ’——	’——	’——	’——	’——	’——	’——	’——	’——
’——	’——	’——	’——	’——	’——	2010 ’——	’——	’——	’——	’——	’——	’——	’——	’——
’——	’——	’——	’——	’——	’——	2010 ’——	’——	’——	’——	’——	’——	’——	’——	’——
’——	’——	’——	’——	’——	’——	1996 ’——	’——	’——	’——	’——	’——	’——	’——	’——
’——	’——	’——	’——	’——	’——	1996 ’——	’——	’——	’——	’——	’——	’——	’——	’——
’——	’——	’——	’——	’——	’——	2011 ’——	’——	’——	’——	’——	’——	’——	’——	’——
’——	’——	’——	’——	’——	’——	2011 ’——	’——	’——	’——	’——	’——	’——	’——	’——
’——	’——	’——	’——	’——	’——	2008 ’——	’——	’——	’——	’——	’——	’——	’——	’——
’——	’——	’——	’——	’——	’——	2008 ’——	’——	’——	’——	’——	’——	’——	’——	’——
’——	’——	’——	’——	’——	’——	2000 ’——	’——	’——	’——	’——	’——	’——	’——	’——
’——	’——	’——	’——	’——	’——	2000 ’——	’——	’——	’——	’——	’——	’——	’——	’——
’——	’——	’——	’——	’——	’——	2007 ’——	’——	’——	’——	’——	’——	’——	’——	’——
’——	’——	’——	’——	’——	’——	2007 ’——	’——	’——	’——	’——	’——	’——	’——	’——
’——	’——	’——	’——	’——	’——	2012 ’——	’——	’——	’——	’——	’——	’——	’——	’——
’——	’——	’——	’——	’——	’——	2012 ’——	’——	’——	’——	’——	’——	’——	’——	’——
’——	’——	’——	’——	’——	’——	2011 ’——	’——	’——	’——	’——	’——	’——	’——	’——
’——	’——	’——	’——	’——	’——	2011 ’——	’——	’——	’——	’——	’——	’——	’——	’——
’——	’——	’——	’——	’——	’——	2012 ’——	’——	’——	’——	’——	’——	’——	’——	’——
’——	’——	’——	’——	’——	’——	2012 ’——	’——	’——	’——	’——	’——	’——	’——	’——
’——	’——	’——	’——	’——	’——	2013 ’——	’——	’——	’——	’——	’——	’——	’——	’——
’——	’——	’——	’——	’——	’——	2013 ’——	’——	’——	’——	’——	’——	’——	’——	’——
’——	’——	’——	’——	’——	’——	2013 ’——	’——	’——	’——	’——	’——	’——	’——	’——
’——	’——	’——	’——	’——	’——	2013 ’——	’——	’——	’——	’——	’——	’——	’——	’——
’——	’——	’——	’——	’——	’——	2011 ’——	’——	’——	’——	’——	’——	’——	’——	’——
’——	’——	’——	’——	’——	’——	2011 ’——	’——	’——	’——	’——	’——	’——	’——	’——
’——	’——	’——	’——	’——	’——	2010 ’——	’——	’——	’——	’——	’——	’——	’——	’——

’--	’--	’--	’--	’--	’--	2010	’--	’--	’--	’--	’--	’--	’--	’--	’--	’--
’--	’--	’--	’--	’--	’--	2011	’--	’--	’--	’--	’--	’--	’--	’--	’--	’--
’--	’--	’--	’--	’--	’--	2011	’--	’--	’--	’--	’--	’--	’--	’--	’--	’--
’--	’--	’--	’--	’--	’--	2010	’--	’--	’--	’--	’--	’--	’--	’--	’--	’--
’--	’--	’--	’--	’--	’--	2010	’--	’--	’--	’--	’--	’--	’--	’--	’--	’--
’--	’--	’--	’--	’--	’--	2009	’--	’--	’--	’--	’--	’--	’--	’--	’--	’--
’--	’--	’--	’--	’--	’--	2009	’--	’--	’--	’--	’--	’--	’--	’--	’--	’--
’--	’--	’--	’--	’--	’--	2007	’--	’--	’--	’--	’--	’--	’--	’--	’--	’--
’--	’--	’--	’--	’--	’--	2007	’--	’--	’--	’--	’--	’--	’--	’--	’--	’--
’--	’--	’--	’--	’--	’--	2010	’--	’--	’--	’--	’--	’--	’--	’--	’--	’--
’--	’--	’--	’--	’--	’--	2010	’--	’--	’--	’--	’--	’--	’--	’--	’--	’--
’--	’--	’--	’--	’--	’--	2008	’--	’--	’--	’--	’--	’--	’--	’--	’--	’--
’--	’--	’--	’--	’--	’--	2008	’--	’--	’--	’--	’--	’--	’--	’--	’--	’--
’--	’--	’--	’--	’--	’--	2009	’--	’--	’--	’--	’--	’--	’--	’--	’--	’--
’--	’--	’--	’--	’--	’--	2009	’--	’--	’--	’--	’--	’--	’--	’--	’--	’--
’--	’--	’--	’--	’--	’--	2008	’--	’--	’--	’--	’--	’--	’--	’--	’--	’--
’--	’--	’--	’--	’--	’--	2008	’--	’--	’--	’--	’--	’--	’--	’--	’--	’--
’--	’--	’--	’--	’--	’--	2012	’--	’--	’--	’--	’--	’--	’--	’--	’--	’--
’--	’--	’--	’--	’--	’--	2012	’--	’--	’--	’--	’--	’--	’--	’--	’--	’--
’--	’--	’--	’--	’--	’--	2008	’--	’--	’--	’--	’--	’--	’--	’--	’--	’--
’--	’--	’--	’--	’--	’--	2008	’--	’--	’--	’--	’--	’--	’--	’--	’--	’--
’--	’--	’--	’--	’--	’--	2010	’--	’--	’--	’--	’--	’--	’--	’--	’--	’--
’--	’--	’--	’--	’--	’--	2010	’--	’--	’--	’--	’--	’--	’--	’--	’--	’--
’--	’--	’--	’--	’--	’--	2009	’--	’--	’--	’--	’--	’--	’--	’--	’--	’--
’--	’--	’--	’--	’--	’--	2009	’--	’--	’--	’--	’--	’--	’--	’--	’--	’--
’--	’--	’--	’--	’--	’--	2011	’--	’--	’--	’--	’--	’--	’--	’--	’--	’--
’--	’--	’--	’--	’--	’--	2011	’--	’--	’--	’--	’--	’--	’--	’--	’--	’--
’--	’--	’--	’--	’--	’--	2013	’--	’--	’--	’--	’--	’--	’--	’--	’--	’--
’--	’--	’--	’--	’--	’--	2013	’--	’--	’--	’--	’--	’--	’--	’--	’--	’--
’--	’--	’--	’--	’--	’--	2010	’--	’--	’--	’--	’--	’--	’--	’--	’--	’--
’--	’--	’--	’--	’--	’--	2010	’--	’--	’--	’--	’--	’--	’--	’--	’--	’--
’--	’--	’--	’--	’--	’--	2012	’--	’--	’--	’--	’--	’--	’--	’--	’--	’--
’--	’--	’--	’--	’--	’--	2012	’--	’--	’--	’--	’--	’--	’--	’--	’--	’--
’--	’--	’--	’--	’--	’--	2012	’--	’--	’--	’--	’--	’--	’--	’--	’--	’--
’--	’--	’--	’--	’--	’--	2012	’--	’--	’--	’--	’--	’--	’--	’--	’--	’--

’--	’--	’--	’--	’--	’--	2010	’--	’--	’--	’--	’--	’--	’--	’--	’--
’--	’--	’--	’--	’--	’--	2010	’--	’--	’--	’--	’--	’--	’--	’--	’--
’--	’--	’--	’--	’--	’--	1998	’--	’--	’--	’--	’--	’--	’--	’--	’--
’--	’--	’--	’--	’--	’--	1998	’--	’--	’--	’--	’--	’--	’--	’--	’--
’--	’--	’--	’--	’--	’--	1996	’--	’--	’--	’--	’--	’--	’--	’--	’--
’--	’--	’--	’--	’--	’--	1996	’--	’--	’--	’--	’--	’--	’--	’--	’--
’--	’--	’--	’--	’--	’--	2013	’--	’--	’--	’--	’--	’--	’--	’--	’--
’--	’--	’--	’--	’--	’--	2013	’--	’--	’--	’--	’--	’--	’--	’--	’--
’--	’--	’--	’--	’--	’--	2008	’--	’--	’--	’--	’--	’--	’--	’--	’--
’--	’--	’--	’--	’--	’--	2008	’--	’--	’--	’--	’--	’--	’--	’--	’--
’--	’--	’--	’--	’--	’--	2007	’--	’--	’--	’--	’--	’--	’--	’--	’--
’--	’--	’--	’--	’--	’--	2007	’--	’--	’--	’--	’--	’--	’--	’--	’--
’--	’--	’--	’--	’--	’--	2010	’--	’--	’--	’--	’--	’--	’--	’--	’--
’--	’--	’--	’--	’--	’--	2010	’--	’--	’--	’--	’--	’--	’--	’--	’--
’--	’--	’--	’--	’--	’--	2003	’--	’--	’--	’--	’--	’--	’--	’--	’--
’--	’--	’--	’--	’--	’--	2003	’--	’--	’--	’--	’--	’--	’--	’--	’--
’--	’--	’--	’--	’--	’--	2008	’--	’--	’--	’--	’--	’--	’--	’--	’--
’--	’--	’--	’--	’--	’--	2008	’--	’--	’--	’--	’--	’--	’--	’--	’--
’--	’--	’--	’--	’--	’--	2013	’--	’--	’--	’--	’--	’--	’--	’--	’--
’--	’--	’--	’--	’--	’--	2013	’--	’--	’--	’--	’--	’--	’--	’--	’--
’--	’--	’--	’--	’--	’--	1998	’--	’--	’--	’--	’--	’--	’--	’--	’--
’--	’--	’--	’--	’--	’--	1998	’--	’--	’--	’--	’--	’--	’--	’--	’--
’--	’--	’--	’--	’--	’--	2010	’--	’--	’--	’--	’--	’--	’--	’--	’--
’--	’--	’--	’--	’--	’--	2010	’--	’--	’--	’--	’--	’--	’--	’--	’--
’--	’--	’--	’--	’--	’--	2007	’--	’--	’--	’--	’--	’--	’--	’--	’--
’--	’--	’--	’--	’--	’--	2007	’--	’--	’--	’--	’--	’--	’--	’--	’--
’--	’--	’--	’--	’--	’--	2004	’--	’--	’--	’--	’--	’--	’--	’--	’--
’--	’--	’--	’--	’--	’--	2004	’--	’--	’--	’--	’--	’--	’--	’--	’--
’--	’--	’--	’--	’--	’--	2011	’--	’--	’--	’--	’--	’--	’--	’--	’--
’--	’--	’--	’--	’--	’--	2011	’--	’--	’--	’--	’--	’--	’--	’--	’--
’--	’--	’--	’--	’--	’--	2012	’--	’--	’--	’--	’--	’--	’--	’--	’--
’--	’--	’--	’--	’--	’--	2012	’--	’--	’--	’--	’--	’--	’--	’--	’--
’--	’--	’--	’--	’--	’--	2007	’--	’--	’--	’--	’--	’--	’--	’--	’--
’--	’--	’--	’--	’--	’--	2007	’--	’--	’--	’--	’--	’--	’--	’--	’--
’--	’--	’--	’--	’--	’--	2007	’--	’--	’--	’--	’--	’--	’--	’--	’--

’__	’__	’__	’__	’__	’__	2007	’--	’__	’__	’__	’__	’__	’__	’__	’__
’__	’__	’__	’__	’__	’__	1998	’--	’__	’__	’__	’__	’__	’__	’__	’__
’__	’__	’__	’__	’__	’__	1998	’--	’__	’__	’__	’__	’__	’__	’__	’__
’__	’__	’__	’__	’__	’__	2013	’--	’__	’__	’__	’__	’__	’__	’__	’__
’__	’__	’__	’__	’__	’__	2013	’--	’__	’__	’__	’__	’__	’__	’__	’__
’__	’__	’__	’__	’__	’__	2010	’--	’__	’__	’__	’__	’__	’__	’__	’__
’__	’__	’__	’__	’__	’__	2010	’--	’__	’__	’__	’__	’__	’__	’__	’__
’__	’__	’__	’__	’__	’__	2008	’--	’__	’__	’__	’__	’__	’__	’__	’__
’__	’__	’__	’__	’__	’__	2008	’--	’__	’__	’__	’__	’__	’__	’__	’__
’__	’__	’__	’__	’__	’__	2005	’--	’__	’__	’__	’__	’__	’__	’__	’__
’__	’__	’__	’__	’__	’__	2005	’--	’__	’__	’__	’__	’__	’__	’__	’__
’__	’__	’__	’__	’__	’__	2008	’--	’__	’__	’__	’__	’__	’__	’__	’__
’__	’__	’__	’__	’__	’__	2008	’--	’__	’__	’__	’__	’__	’__	’__	’__
’__	’__	’__	’__	’__	’__	1998	’--	’__	’__	’__	’__	’__	’__	’__	’__
’__	’__	’__	’__	’__	’__	1998	’--	’__	’__	’__	’__	’__	’__	’__	’__
’__	’__	’__	’__	’__	’__	2006	’--	’__	’__	’__	’__	’__	’__	’__	’__
’__	’__	’__	’__	’__	’__	2006	’--	’__	’__	’__	’__	’__	’__	’__	’__
’__	’__	’__	’__	’__	’__	2011	’--	’__	’__	’__	’__	’__	’__	’__	’__
’__	’__	’__	’__	’__	’__	2011	’--	’__	’__	’__	’__	’__	’__	’__	’__
’__	’__	’__	’__	’__	’__	2010	’--	’__	’__	’__	’__	’__	’__	’__	’__
’__	’__	’__	’__	’__	’__	2010	’--	’__	’__	’__	’__	’__	’__	’__	’__
’__	’__	’__	’__	’__	’__	2009	’--	’__	’__	’__	’__	’__	’__	’__	’__
’__	’__	’__	’__	’__	’__	2009	’--	’__	’__	’__	’__	’__	’__	’__	’__
’__	’__	’__	’__	’__	’__	2009	’--	’__	’__	’__	’__	’__	’__	’__	’__
’__	’__	’__	’__	’__	’__	2009	’--	’__	’__	’__	’__	’__	’__	’__	’__
’__	’__	’__	’__	’__	’__	1998	’--	’__	’__	’__	’__	’__	’__	’__	’__
’__	’__	’__	’__	’__	’__	1998	’--	’__	’__	’__	’__	’__	’__	’__	’__
’__	’__	’__	’__	’__	’__	2012	’--	’__	’__	’__	’__	’__	’__	’__	’__
’__	’__	’__	’__	’__	’__	2012	’--	’__	’__	’__	’__	’__	’__	’__	’__
’__	’__	’__	’__	’__	’__	2004	’--	’__	’__	’__	’__	’__	’__	’__	’__
’__	’__	’__	’__	’__	’__	2004	’--	’__	’__	’__	’__	’__	’__	’__	’__
’__	’__	’__	’__	’__	’__	2009	’--	’__	’__	’__	’__	’__	’__	’__	’__
’__	’__	’__	’__	’__	’__	2009	’--	’__	’__	’__	’__	’__	’__	’__	’__
’__	’__	’__	’__	’__	’__	2012	’--	’__	’__	’__	’__	’__	’__	’__	’__
’__	’__	’__	’__	’__	’__	2012	’--	’__	’__	’__	’__	’__	’__	’__	’__

’--	’--	’--	’--	’--	’--	2007	’--	’--	’--	’--	’--	’--	’--	’--	’--
’--	’--	’--	’--	’--	’--	2007	’--	’--	’--	’--	’--	’--	’--	’--	’--
’--	’--	’--	’--	’--	’--	2010	’--	’--	’--	’--	’--	’--	’--	’--	’--
’--	’--	’--	’--	’--	’--	2010	’--	’--	’--	’--	’--	’--	’--	’--	’--
’--	’--	’--	’--	’--	’--	2000	’--	’--	’--	’--	’--	’--	’--	’--	’--
’--	’--	’--	’--	’--	’--	2000	’--	’--	’--	’--	’--	’--	’--	’--	’--
’--	’--	’--	’--	’--	’--	2012	’--	’--	’--	’--	’--	’--	’--	’--	’--
’--	’--	’--	’--	’--	’--	2012	’--	’--	’--	’--	’--	’--	’--	’--	’--
’--	’--	’--	’--	’--	’--	2011	’--	’--	’--	’--	’--	’--	’--	’--	’--
’--	’--	’--	’--	’--	’--	2011	’--	’--	’--	’--	’--	’--	’--	’--	’--

treatment	treatment	treatment	treatment	treatment	treatment	treatment	treatment	treatment	treatment_type
'--	'--	'--	'--	'--	'--	'--	yes	'--	Pharmaceutical Therapy, NOS
'--	'--	'--	'--	'--	'--	'--	yes	'--	Radiation Therapy, NOS
'--	'--	'--	'--	'--	'--	'--	no	'--	Pharmaceutical Therapy, NOS
'--	'--	'--	'--	'--	'--	'--	yes	'--	Radiation Therapy, NOS
'--	'--	'--	'--	'--	'--	'--	no	'--	Radiation Therapy, NOS
'--	'--	'--	'--	'--	'--	'--	no	'--	Pharmaceutical Therapy, NOS
'--	'--	'--	'--	'--	'--	'--	yes	'--	Radiation Therapy, NOS
'--	'--	'--	'--	'--	'--	'--	yes	'--	Pharmaceutical Therapy, NOS
'--	'--	'--	'--	'--	'--	'--	no	'--	Pharmaceutical Therapy, NOS
'--	'--	'--	'--	'--	'--	'--	no	'--	Radiation Therapy, NOS
'--	'--	'--	'--	'--	'--	'--	yes	'--	Radiation Therapy, NOS
'--	'--	'--	'--	'--	'--	'--	yes	'--	Pharmaceutical Therapy, NOS
'--	'--	'--	'--	'--	'--	'--	no	'--	Radiation Therapy, NOS
'--	'--	'--	'--	'--	'--	'--	no	'--	Pharmaceutical Therapy, NOS
'--	'--	'--	'--	'--	'--	'--	no	'--	Radiation Therapy, NOS
'--	'--	'--	'--	'--	'--	'--	no	'--	Pharmaceutical Therapy, NOS
'--	'--	'--	'--	'--	'--	'--	no	'--	Radiation Therapy, NOS
'--	'--	'--	'--	'--	'--	'--	no	'--	Pharmaceutical Therapy, NOS
'--	'--	'--	'--	'--	'--	'--	no	'--	Pharmaceutical Therapy, NOS
'--	'--	'--	'--	'--	'--	'--	no	'--	Radiation Therapy, NOS
'--	'--	'--	'--	'--	'--	'--	yes	'--	Radiation Therapy, NOS
'--	'--	'--	'--	'--	'--	'--	no	'--	Pharmaceutical Therapy, NOS
'--	'--	'--	'--	'--	'--	'--	no	'--	Radiation Therapy, NOS
'--	'--	'--	'--	'--	'--	'--	no	'--	Pharmaceutical Therapy, NOS
'--	'--	'--	'--	'--	'--	'--	yes	'--	Pharmaceutical Therapy, NOS
'--	'--	'--	'--	'--	'--	'--	yes	'--	Radiation Therapy, NOS
'--	'--	'--	'--	'--	'--	'--	yes	'--	Radiation Therapy, NOS
'--	'--	'--	'--	'--	'--	'--	yes	'--	Pharmaceutical Therapy, NOS
'--	'--	'--	'--	'--	'--	'--	no	'--	Pharmaceutical Therapy, NOS
'--	'--	'--	'--	'--	'--	'--	no	'--	Radiation Therapy, NOS
'--	'--	'--	'--	'--	'--	'--	no	'--	Radiation Therapy, NOS
'--	'--	'--	'--	'--	'--	'--	no	'--	Pharmaceutical Therapy, NOS
'--	'--	'--	'--	'--	'--	'--	yes	'--	Radiation Therapy, NOS
'--	'--	'--	'--	'--	'--	'--	no	'--	Pharmaceutical Therapy, NOS

'--	'--	'--	'--	'--	'--	'--	no	'--	Pharmaceutical Therapy, NOS
'--	'--	'--	'--	'--	'--	'--	yes	'--	Radiation Therapy, NOS
'--	'--	'--	'--	'--	'--	'--	no	'--	Pharmaceutical Therapy, NOS
'--	'--	'--	'--	'--	'--	'--	no	'--	Radiation Therapy, NOS
'--	'--	'--	'--	'--	'--	'--	not repor	'--	Pharmaceutical Therapy, NOS
'--	'--	'--	'--	'--	'--	'--	not repor	'--	Radiation Therapy, NOS
'--	'--	'--	'--	'--	'--	'--	yes	'--	Radiation Therapy, NOS
'--	'--	'--	'--	'--	'--	'--	yes	'--	Pharmaceutical Therapy, NOS
'--	'--	'--	'--	'--	'--	'--	yes	'--	Radiation Therapy, NOS
'--	'--	'--	'--	'--	'--	'--	no	'--	Pharmaceutical Therapy, NOS
'--	'--	'--	'--	'--	'--	'--	no	'--	Pharmaceutical Therapy, NOS
'--	'--	'--	'--	'--	'--	'--	no	'--	Radiation Therapy, NOS
'--	'--	'--	'--	'--	'--	'--	yes	'--	Radiation Therapy, NOS
'--	'--	'--	'--	'--	'--	'--	yes	'--	Pharmaceutical Therapy, NOS
'--	'--	'--	'--	'--	'--	'--	no	'--	Pharmaceutical Therapy, NOS
'--	'--	'--	'--	'--	'--	'--	no	'--	Radiation Therapy, NOS
'--	'--	'--	'--	'--	'--	'--	not repor	'--	Radiation Therapy, NOS
'--	'--	'--	'--	'--	'--	'--	not repor	'--	Pharmaceutical Therapy, NOS
'--	'--	'--	'--	'--	'--	'--	no	'--	Pharmaceutical Therapy, NOS
'--	'--	'--	'--	'--	'--	'--	no	'--	Radiation Therapy, NOS
'--	'--	'--	'--	'--	'--	'--	yes	'--	Radiation Therapy, NOS
'--	'--	'--	'--	'--	'--	'--	yes	'--	Pharmaceutical Therapy, NOS
'--	'--	'--	'--	'--	'--	'--	no	'--	Pharmaceutical Therapy, NOS
'--	'--	'--	'--	'--	'--	'--	no	'--	Radiation Therapy, NOS
'--	'--	'--	'--	'--	'--	'--	not repor	'--	Radiation Therapy, NOS
'--	'--	'--	'--	'--	'--	'--	not repor	'--	Pharmaceutical Therapy, NOS
'--	'--	'--	'--	'--	'--	'--	yes	'--	Pharmaceutical Therapy, NOS
'--	'--	'--	'--	'--	'--	'--	yes	'--	Radiation Therapy, NOS
'--	'--	'--	'--	'--	'--	'--	no	'--	Pharmaceutical Therapy, NOS
'--	'--	'--	'--	'--	'--	'--	yes	'--	Radiation Therapy, NOS
'--	'--	'--	'--	'--	'--	'--	yes	'--	Radiation Therapy, NOS
'--	'--	'--	'--	'--	'--	'--	yes	'--	Pharmaceutical Therapy, NOS
'--	'--	'--	'--	'--	'--	'--	no	'--	Radiation Therapy, NOS
'--	'--	'--	'--	'--	'--	'--	no	'--	Pharmaceutical Therapy, NOS
'--	'--	'--	'--	'--	'--	'--	yes	'--	Radiation Therapy, NOS

'--	'--	'--	'--	'--	'--	'--	yes	'--	Pharmaceutical Therapy, NOS
'--	'--	'--	'--	'--	'--	'--	not repor	'--	Radiation Therapy, NOS
'--	'--	'--	'--	'--	'--	'--	not repor	'--	Pharmaceutical Therapy, NOS
'--	'--	'--	'--	'--	'--	'--	yes	'--	Radiation Therapy, NOS
'--	'--	'--	'--	'--	'--	'--	no	'--	Pharmaceutical Therapy, NOS
'--	'--	'--	'--	'--	'--	'--	no	'--	Pharmaceutical Therapy, NOS
'--	'--	'--	'--	'--	'--	'--	no	'--	Radiation Therapy, NOS
'--	'--	'--	'--	'--	'--	'--	no	'--	Radiation Therapy, NOS
'--	'--	'--	'--	'--	'--	'--	no	'--	Pharmaceutical Therapy, NOS
'--	'--	'--	'--	'--	'--	'--	yes	'--	Radiation Therapy, NOS
'--	'--	'--	'--	'--	'--	'--	no	'--	Pharmaceutical Therapy, NOS
'--	'--	'--	'--	'--	'--	'--	yes	'--	Pharmaceutical Therapy, NOS
'--	'--	'--	'--	'--	'--	'--	yes	'--	Radiation Therapy, NOS
'--	'--	'--	'--	'--	'--	'--	yes	'--	Radiation Therapy, NOS
'--	'--	'--	'--	'--	'--	'--	yes	'--	Pharmaceutical Therapy, NOS
'--	'--	'--	'--	'--	'--	'--	no	'--	Pharmaceutical Therapy, NOS
'--	'--	'--	'--	'--	'--	'--	yes	'--	Radiation Therapy, NOS
'--	'--	'--	'--	'--	'--	'--	no	'--	Radiation Therapy, NOS
'--	'--	'--	'--	'--	'--	'--	no	'--	Pharmaceutical Therapy, NOS
'--	'--	'--	'--	'--	'--	'--	not repor	'--	Radiation Therapy, NOS
'--	'--	'--	'--	'--	'--	'--	not repor	'--	Pharmaceutical Therapy, NOS
'--	'--	'--	'--	'--	'--	'--	no	'--	Pharmaceutical Therapy, NOS
'--	'--	'--	'--	'--	'--	'--	no	'--	Radiation Therapy, NOS
'--	'--	'--	'--	'--	'--	'--	yes	'--	Radiation Therapy, NOS
'--	'--	'--	'--	'--	'--	'--	yes	'--	Pharmaceutical Therapy, NOS
'--	'--	'--	'--	'--	'--	'--	yes	'--	Radiation Therapy, NOS
'--	'--	'--	'--	'--	'--	'--	no	'--	Pharmaceutical Therapy, NOS
'--	'--	'--	'--	'--	'--	'--	yes	'--	Radiation Therapy, NOS
'--	'--	'--	'--	'--	'--	'--	no	'--	Pharmaceutical Therapy, NOS
'--	'--	'--	'--	'--	'--	'--	yes	'--	Pharmaceutical Therapy, NOS
'--	'--	'--	'--	'--	'--	'--	yes	'--	Radiation Therapy, NOS
'--	'--	'--	'--	'--	'--	'--	no	'--	Pharmaceutical Therapy, NOS
'--	'--	'--	'--	'--	'--	'--	yes	'--	Radiation Therapy, NOS
'--	'--	'--	'--	'--	'--	'--	no	'--	Pharmaceutical Therapy, NOS
'--	'--	'--	'--	'--	'--	'--	yes	'--	Radiation Therapy, NOS

'--	'--	'--	'--	'--	'--	'--	yes	'--	Radiation Therapy, NOS
'--	'--	'--	'--	'--	'--	'--	yes	'--	Pharmaceutical Therapy, NOS
'--	'--	'--	'--	'--	'--	'--	yes	'--	Radiation Therapy, NOS
'--	'--	'--	'--	'--	'--	'--	no	'--	Pharmaceutical Therapy, NOS
'--	'--	'--	'--	'--	'--	'--	yes	'--	Radiation Therapy, NOS
'--	'--	'--	'--	'--	'--	'--	yes	'--	Pharmaceutical Therapy, NOS
'--	'--	'--	'--	'--	'--	'--	yes	'--	Pharmaceutical Therapy, NOS
'--	'--	'--	'--	'--	'--	'--	yes	'--	Radiation Therapy, NOS
'--	'--	'--	'--	'--	'--	'--	no	'--	Radiation Therapy, NOS
'--	'--	'--	'--	'--	'--	'--	no	'--	Pharmaceutical Therapy, NOS
'--	'--	'--	'--	'--	'--	'--	yes	'--	Radiation Therapy, NOS
'--	'--	'--	'--	'--	'--	'--	yes	'--	Pharmaceutical Therapy, NOS
'--	'--	'--	'--	'--	'--	'--	no	'--	Pharmaceutical Therapy, NOS
'--	'--	'--	'--	'--	'--	'--	yes	'--	Radiation Therapy, NOS
'--	'--	'--	'--	'--	'--	'--	yes	'--	Radiation Therapy, NOS
'--	'--	'--	'--	'--	'--	'--	no	'--	Pharmaceutical Therapy, NOS
'--	'--	'--	'--	'--	'--	'--	no	'--	Pharmaceutical Therapy, NOS
'--	'--	'--	'--	'--	'--	'--	no	'--	Radiation Therapy, NOS
'--	'--	'--	'--	'--	'--	'--	no	'--	Radiation Therapy, NOS
'--	'--	'--	'--	'--	'--	'--	no	'--	Pharmaceutical Therapy, NOS
'--	'--	'--	'--	'--	'--	'--	no	'--	Pharmaceutical Therapy, NOS
'--	'--	'--	'--	'--	'--	'--	yes	'--	Radiation Therapy, NOS
'--	'--	'--	'--	'--	'--	'--	no	'--	Pharmaceutical Therapy, NOS
'--	'--	'--	'--	'--	'--	'--	no	'--	Radiation Therapy, NOS
'--	'--	'--	'--	'--	'--	'--	yes	'--	Radiation Therapy, NOS
'--	'--	'--	'--	'--	'--	'--	yes	'--	Pharmaceutical Therapy, NOS
'--	'--	'--	'--	'--	'--	'--	yes	'--	Radiation Therapy, NOS
'--	'--	'--	'--	'--	'--	'--	no	'--	Pharmaceutical Therapy, NOS
'--	'--	'--	'--	'--	'--	'--	yes	'--	Radiation Therapy, NOS
'--	'--	'--	'--	'--	'--	'--	yes	'--	Pharmaceutical Therapy, NOS
'--	'--	'--	'--	'--	'--	'--	yes	'--	Radiation Therapy, NOS
'--	'--	'--	'--	'--	'--	'--	yes	'--	Pharmaceutical Therapy, NOS
'--	'--	'--	'--	'--	'--	'--	yes	'--	Radiation Therapy, NOS
'--	'--	'--	'--	'--	'--	'--	yes	'--	Pharmaceutical Therapy, NOS
'--	'--	'--	'--	'--	'--	'--	no	'--	Pharmaceutical Therapy, NOS

'--	'--	'--	'--	'--	'--	'--	no	'--	Radiation Therapy, NOS
'--	'--	'--	'--	'--	'--	'--	no	'--	Pharmaceutical Therapy, NOS
'--	'--	'--	'--	'--	'--	'--	yes	'--	Radiation Therapy, NOS
'--	'--	'--	'--	'--	'--	'--	no	'--	Pharmaceutical Therapy, NOS
'--	'--	'--	'--	'--	'--	'--	yes	'--	Radiation Therapy, NOS
'--	'--	'--	'--	'--	'--	'--	yes	'--	Radiation Therapy, NOS
'--	'--	'--	'--	'--	'--	'--	yes	'--	Pharmaceutical Therapy, NOS
'--	'--	'--	'--	'--	'--	'--	yes	'--	Radiation Therapy, NOS
'--	'--	'--	'--	'--	'--	'--	no	'--	Pharmaceutical Therapy, NOS
'--	'--	'--	'--	'--	'--	'--	not repor	'--	Radiation Therapy, NOS
'--	'--	'--	'--	'--	'--	'--	not repor	'--	Pharmaceutical Therapy, NOS
'--	'--	'--	'--	'--	'--	'--	yes	'--	Radiation Therapy, NOS
'--	'--	'--	'--	'--	'--	'--	yes	'--	Pharmaceutical Therapy, NOS
'--	'--	'--	'--	'--	'--	'--	no	'--	Pharmaceutical Therapy, NOS
'--	'--	'--	'--	'--	'--	'--	yes	'--	Radiation Therapy, NOS
'--	'--	'--	'--	'--	'--	'--	no	'--	Pharmaceutical Therapy, NOS
'--	'--	'--	'--	'--	'--	'--	yes	'--	Radiation Therapy, NOS
'--	'--	'--	'--	'--	'--	'--	yes	'--	Radiation Therapy, NOS
'--	'--	'--	'--	'--	'--	'--	no	'--	Pharmaceutical Therapy, NOS
'--	'--	'--	'--	'--	'--	'--	no	'--	Pharmaceutical Therapy, NOS
'--	'--	'--	'--	'--	'--	'--	no	'--	Radiation Therapy, NOS
'--	'--	'--	'--	'--	'--	'--	no	'--	Pharmaceutical Therapy, NOS
'--	'--	'--	'--	'--	'--	'--	yes	'--	Radiation Therapy, NOS
'--	'--	'--	'--	'--	'--	'--	no	'--	Radiation Therapy, NOS
'--	'--	'--	'--	'--	'--	'--	no	'--	Pharmaceutical Therapy, NOS
'--	'--	'--	'--	'--	'--	'--	yes	'--	Radiation Therapy, NOS
'--	'--	'--	'--	'--	'--	'--	yes	'--	Pharmaceutical Therapy, NOS
'--	'--	'--	'--	'--	'--	'--	no	'--	Pharmaceutical Therapy, NOS
'--	'--	'--	'--	'--	'--	'--	yes	'--	Radiation Therapy, NOS
'--	'--	'--	'--	'--	'--	'--	yes	'--	Radiation Therapy, NOS
'--	'--	'--	'--	'--	'--	'--	yes	'--	Pharmaceutical Therapy, NOS
'--	'--	'--	'--	'--	'--	'--	yes	'--	Radiation Therapy, NOS
'--	'--	'--	'--	'--	'--	'--	no	'--	Pharmaceutical Therapy, NOS
'--	'--	'--	'--	'--	'--	'--	no	'--	Pharmaceutical Therapy, NOS
'--	'--	'--	'--	'--	'--	'--	yes	'--	Radiation Therapy, NOS

'--	'--	'--	'--	'--	'--	'--	no	'--	Pharmaceutical Therapy, NOS
'--	'--	'--	'--	'--	'--	'--	no	'--	Radiation Therapy, NOS
'--	'--	'--	'--	'--	'--	'--	yes	'--	Radiation Therapy, NOS
'--	'--	'--	'--	'--	'--	'--	no	'--	Pharmaceutical Therapy, NOS
'--	'--	'--	'--	'--	'--	'--	not repor	'--	Radiation Therapy, NOS
'--	'--	'--	'--	'--	'--	'--	not repor	'--	Pharmaceutical Therapy, NOS
'--	'--	'--	'--	'--	'--	'--	no	'--	Pharmaceutical Therapy, NOS
'--	'--	'--	'--	'--	'--	'--	no	'--	Radiation Therapy, NOS
'--	'--	'--	'--	'--	'--	'--	no	'--	Radiation Therapy, NOS
'--	'--	'--	'--	'--	'--	'--	no	'--	Pharmaceutical Therapy, NOS
'--	'--	'--	'--	'--	'--	'--	not repor	'--	Pharmaceutical Therapy, NOS
'--	'--	'--	'--	'--	'--	'--	not repor	'--	Radiation Therapy, NOS
'--	'--	'--	'--	'--	'--	'--	no	'--	Pharmaceutical Therapy, NOS
'--	'--	'--	'--	'--	'--	'--	no	'--	Radiation Therapy, NOS
'--	'--	'--	'--	'--	'--	'--	yes	'--	Pharmaceutical Therapy, NOS
'--	'--	'--	'--	'--	'--	'--	no	'--	Radiation Therapy, NOS
'--	'--	'--	'--	'--	'--	'--	no	'--	Pharmaceutical Therapy, NOS
'--	'--	'--	'--	'--	'--	'--	yes	'--	Radiation Therapy, NOS
'--	'--	'--	'--	'--	'--	'--	yes	'--	Radiation Therapy, NOS
'--	'--	'--	'--	'--	'--	'--	no	'--	Pharmaceutical Therapy, NOS
'--	'--	'--	'--	'--	'--	'--	no	'--	Pharmaceutical Therapy, NOS
'--	'--	'--	'--	'--	'--	'--	no	'--	Radiation Therapy, NOS
'--	'--	'--	'--	'--	'--	'--	no	'--	Radiation Therapy, NOS
'--	'--	'--	'--	'--	'--	'--	no	'--	Pharmaceutical Therapy, NOS
'--	'--	'--	'--	'--	'--	'--	yes	'--	Radiation Therapy, NOS
'--	'--	'--	'--	'--	'--	'--	no	'--	Pharmaceutical Therapy, NOS
'--	'--	'--	'--	'--	'--	'--	yes	'--	Pharmaceutical Therapy, NOS
'--	'--	'--	'--	'--	'--	'--	no	'--	Radiation Therapy, NOS
'--	'--	'--	'--	'--	'--	'--	yes	'--	Pharmaceutical Therapy, NOS
'--	'--	'--	'--	'--	'--	'--	yes	'--	Radiation Therapy, NOS
'--	'--	'--	'--	'--	'--	'--	no	'--	Radiation Therapy, NOS
'--	'--	'--	'--	'--	'--	'--	no	'--	Pharmaceutical Therapy, NOS
'--	'--	'--	'--	'--	'--	'--	no	'--	Radiation Therapy, NOS
'--	'--	'--	'--	'--	'--	'--	no	'--	Pharmaceutical Therapy, NOS
'--	'--	'--	'--	'--	'--	'--	no	'--	Pharmaceutical Therapy, NOS

'--	'--	'--	'--	'--	'--	'--	yes	'--	Radiation Therapy, NOS
'--	'--	'--	'--	'--	'--	'--	yes	'--	Radiation Therapy, NOS
'--	'--	'--	'--	'--	'--	'--	no	'--	Pharmaceutical Therapy, NOS
'--	'--	'--	'--	'--	'--	'--	yes	'--	Radiation Therapy, NOS
'--	'--	'--	'--	'--	'--	'--	no	'--	Pharmaceutical Therapy, NOS
'--	'--	'--	'--	'--	'--	'--	yes	'--	Pharmaceutical Therapy, NOS
'--	'--	'--	'--	'--	'--	'--	yes	'--	Radiation Therapy, NOS
'--	'--	'--	'--	'--	'--	'--	yes	'--	Pharmaceutical Therapy, NOS
'--	'--	'--	'--	'--	'--	'--	yes	'--	Radiation Therapy, NOS
'--	'--	'--	'--	'--	'--	'--	no	'--	Pharmaceutical Therapy, NOS
'--	'--	'--	'--	'--	'--	'--	yes	'--	Radiation Therapy, NOS
'--	'--	'--	'--	'--	'--	'--	yes	'--	Pharmaceutical Therapy, NOS
'--	'--	'--	'--	'--	'--	'--	yes	'--	Radiation Therapy, NOS
'--	'--	'--	'--	'--	'--	'--	yes	'--	Radiation Therapy, NOS
'--	'--	'--	'--	'--	'--	'--	not repor	'--	Pharmaceutical Therapy, NOS
'--	'--	'--	'--	'--	'--	'--	yes	'--	Radiation Therapy, NOS
'--	'--	'--	'--	'--	'--	'--	yes	'--	Pharmaceutical Therapy, NOS
'--	'--	'--	'--	'--	'--	'--	yes	'--	Radiation Therapy, NOS
'--	'--	'--	'--	'--	'--	'--	yes	'--	Pharmaceutical Therapy, NOS
'--	'--	'--	'--	'--	'--	'--	yes	'--	Radiation Therapy, NOS
'--	'--	'--	'--	'--	'--	'--	yes	'--	Pharmaceutical Therapy, NOS
'--	'--	'--	'--	'--	'--	'--	not repor	'--	Pharmaceutical Therapy, NOS
'--	'--	'--	'--	'--	'--	'--	not repor	'--	Radiation Therapy, NOS
'--	'--	'--	'--	'--	'--	'--	no	'--	Pharmaceutical Therapy, NOS
'--	'--	'--	'--	'--	'--	'--	yes	'--	Radiation Therapy, NOS
'--	'--	'--	'--	'--	'--	'--	not repor	'--	Radiation Therapy, NOS
'--	'--	'--	'--	'--	'--	'--	not repor	'--	Pharmaceutical Therapy, NOS
'--	'--	'--	'--	'--	'--	'--	not repor	'--	Radiation Therapy, NOS
'--	'--	'--	'--	'--	'--	'--	not repor	'--	Pharmaceutical Therapy, NOS
'--	'--	'--	'--	'--	'--	'--	yes	'--	Pharmaceutical Therapy, NOS
'--	'--	'--	'--	'--	'--	'--	yes	'--	Radiation Therapy, NOS
'--	'--	'--	'--	'--	'--	'--	no	'--	Pharmaceutical Therapy, NOS
'--	'--	'--	'--	'--	'--	'--	no	'--	Radiation Therapy, NOS
'--	'--	'--	'--	'--	'--	'--	no	'--	Pharmaceutical Therapy, NOS
'--	'--	'--	'--	'--	'--	'--	no	'--	Radiation Therapy, NOS

'--	'--	'--	'--	'--	'--	'--	no	'--	Radiation Therapy, NOS
'--	'--	'--	'--	'--	'--	'--	no	'--	Pharmaceutical Therapy, NOS
'--	'--	'--	'--	'--	'--	'--	no	'--	Pharmaceutical Therapy, NOS
'--	'--	'--	'--	'--	'--	'--	yes	'--	Radiation Therapy, NOS
'--	'--	'--	'--	'--	'--	'--	not repor	'--	Pharmaceutical Therapy, NOS
'--	'--	'--	'--	'--	'--	'--	not repor	'--	Radiation Therapy, NOS
'--	'--	'--	'--	'--	'--	'--	no	'--	Pharmaceutical Therapy, NOS
'--	'--	'--	'--	'--	'--	'--	yes	'--	Radiation Therapy, NOS
'--	'--	'--	'--	'--	'--	'--	no	'--	Radiation Therapy, NOS
'--	'--	'--	'--	'--	'--	'--	no	'--	Pharmaceutical Therapy, NOS
