# Supplementary material for: Repurposing Dihydroartemisinin to Combat Oral Squamous Cell Carcinoma, Associated with Mitochondrial Dysfunction and Oxidative Stress
Source: Oxid Med Cell Longev. 2023 Feb 16;2023:9595201. doi: 10.1155/2023/9595201 (PMC10239307; doi:10.1155/2023/9595201)
Supplement: Supplementary 11 — Supplementary Table 3: list of differential gene expression in OSCC tumor samples compared to the adjacent normal tissue samples. [file 9595201.f11.pdf]

Gene.stable.ID	Gene.name	Gene.type	logFC	AveExpr	t	P.Value	adj.P.Val	B	stable_id	SYMBOL
ENSG00000005059	MCUB	protein_coding	1.02497	3.034487625	6.074630061	1.08E-08	2.29E-07	9.502066258	ENSG00000005059.14	MCUB
ENSG00000005884	ITGA3	protein_coding	2.194987	5.66341637	7.507787142	6.04E-12	3.93E-10	16.76717058	ENSG00000005884.16	ITGA3
ENSG00000011422	PLAUR	protein_coding	1.595646	3.690688075	6.570281163	8.82E-10	2.71E-08	11.92540769	ENSG00000011422.10	PLAUR
ENSG00000019549	SNAI2	protein_coding	2.187292	5.05017756	11.58172824	2.90E-22	5.28E-19	39.94426599	ENSG00000019549.7	SNAI2
ENSG00000025708	TYMP	protein_coding	2.065338	6.734988478	6.617874834	6.90E-10	2.23E-08	12.1634984	ENSG00000025708.11	TYMP
ENSG00000030110	BAK1	protein_coding	1.115501	4.819562798	5.486833679	1.82E-07	2.60E-06	6.775135013	ENSG00000030110.11	BAK1
ENSG00000049768	FOXP3	protein_coding	1.044885	1.798404083	4.2597131	3.70E-05	0.000227325	1.699550774	ENSG00000049768.13	FOXP3
ENSG00000051180	RAD51	protein_coding	1.115332	2.197377554	6.204466512	5.65E-09	1.30E-07	10.12657505	ENSG00000051180.15	RAD51
ENSG00000058085	LAMC2	protein_coding	4.391307	7.187677075	10.8448481	2.41E-20	1.75E-17	35.63204047	ENSG00000058085.13	LAMC2
ENSG00000062716	VMP1	protein_coding	1.048568	4.217544599	5.100205546	1.07E-06	1.17E-05	5.079445626	ENSG00000062716.9	VMP1
ENSG00000066855	MTFR1	protein_coding	1.042155	3.091291413	6.254817015	4.39E-09	1.04E-07	10.37077992	ENSG00000066855.14	MTFR1
ENSG00000072571	HMMR	protein_coding	1.262578	2.136290039	5.910791381	2.41E-08	4.62E-07	8.725049214	ENSG00000072571.18	HMMR
ENSG00000072864	NDE1	protein_coding	1.161985	2.881571351	6.755843453	3.37E-10	1.23E-08	12.85870763	ENSG00000072864.11	NDE1
ENSG00000073282	TP63	protein_coding	1.227257	5.742187803	4.769630857	4.52E-06	3.92E-05	3.697549681	ENSG00000073282.11	TP63
ENSG00000075218	GTSE1	protein_coding	1.176987	2.016681985	6.947412362	1.23E-10	5.23E-09	13.83591772	ENSG00000075218.17	GTSE1
ENSG00000075223	SEMA3C	protein_coding	1.594832	3.896445413	5.605485997	1.04E-07	1.62E-06	7.311684766	ENSG00000075223.12	SEMA3C
ENSG00000076706	MCAM	protein_coding	1.222737	3.652621686	5.685510376	7.14E-08	1.17E-06	7.677654181	ENSG00000076706.13	MCAM
ENSG00000085840	ORC1	protein_coding	1.011457	2.067498538	5.120591303	9.73E-07	1.08E-05	5.166768086	ENSG00000085840.11	ORC1
ENSG00000087086	FTL	protein_coding	1.317603	10.44607262	5.329933672	3.77E-07	4.81E-06	6.077079004	ENSG00000087086.12	FTL
ENSG00000087586	AURKA	protein_coding	1.712198	3.223001874	7.930966026	5.82E-13	5.82E-11	19.04408683	ENSG00000087586.16	AURKA
ENSG00000088325	TPX2	protein_coding	2.071818	4.516157568	8.219392122	1.15E-13	1.48E-11	20.62285434	ENSG00000088325.14	TPX2
ENSG00000090339	ICAM1	protein_coding	1.528762	4.110483538	4.830126704	3.49E-06	3.16E-05	3.945536894	ENSG00000090339.7	ICAM1
ENSG00000091136	LAMB1	protein_coding	1.911219	4.432428077	8.106235418	2.18E-13	2.54E-11	20.00105541	ENSG00000091136.12	LAMB1
ENSG00000091409	ITGA6	protein_coding	2.281058	6.792619075	7.247572153	2.48E-11	1.35E-09	15.39321939	ENSG00000091409.13	ITGA6
ENSG00000094804	CDC6	protein_coding	1.31707	2.491587615	6.255180123	4.38E-09	1.04E-07	10.37254507	ENSG00000094804.8	CDC6
ENSG00000095970	TREM2	protein_coding	2.271733	3.270478594	6.836845114	2.20E-10	8.75E-09	13.27025019	ENSG00000095970.15	TREM2
ENSG00000100311	PDGFB	protein_coding	1.066543	2.738303097	4.46619127	1.61E-05	0.000113504	2.48824711	ENSG00000100311.15	PDGFB
ENSG00000100644	HIF1A	protein_coding	1.219605	5.729419104	5.308929727	4.15E-07	5.21E-06	5.984643632	ENSG00000100644.15	HIF1A
ENSG00000100985	MMP9	protein_coding	3.720084	5.292793008	8.009595711	3.75E-13	4.01E-11	19.47245587	ENSG00000100985.7	MMP9
ENSG00000101224	CDC25B	protein_coding	1.71363	5.137135291	7.416085178	9.95E-12	6.16E-10	16.28057288	ENSG00000101224.16	CDC25B
ENSG00000101412	E2F1	protein_coding	1.407186	2.757106335	6.045603849	1.24E-08	2.57E-07	9.363497109	ENSG00000101412.12	E2F1
ENSG00000103490	PYCARD	protein_coding	1.020777	4.711489656	4.21593591	4.40E-05	0.000262973	1.536012666	ENSG00000103490.13	PYCARD
ENSG00000104415	CCN4	protein_coding	1.757699	1.853111617	5.626149168	9.46E-08	1.50E-06	7.405869487	ENSG00000104415.12	CCN4
ENSG00000105173	CCNE1	protein_coding	1.121567	2.085214973	6.221629563	5.19E-09	1.20E-07	10.20969229	ENSG00000105173.12	CCNE1

ENSG00000105329	TGFB1	protein_coding	1.968386	5.341356432	11.34091368	1.23E-21	1.72E-18	38.53390376	ENSG00000105329.8	TGFB1
ENSG00000105974	CAV1	protein_coding	2.386333	6.403847532	7.384272964	1.18E-11	7.15E-10	16.11237033	ENSG00000105974.10	CAV1
ENSG00000105976	MET	protein_coding	1.475881	3.792053492	6.512906548	1.18E-09	3.49E-08	11.63959349	ENSG00000105976.13	MET
ENSG00000106366	SERPINE1	protein_coding	4.306936	6.21091535	10.85281874	2.30E-20	1.74E-17	35.67860645	ENSG00000106366.8	SERPINE1
ENSG00000106799	TGFBR1	protein_coding	1.112333	3.639830371	6.401355022	2.09E-09	5.57E-08	11.08774657	ENSG00000106799.11	TGFBR1
ENSG00000108518	PFN1	protein_coding	1.095865	8.58146399	6.829737763	2.29E-10	9.03E-09	13.23404197	ENSG00000108518.7	PFN1
ENSG00000108821	COL1A1	protein_coding	4.391736	7.927979969	8.837126205	3.37E-15	6.74E-13	24.06598004	ENSG00000108821.12	COL1A1
ENSG00000111012	CYP27B1	protein_coding	1.637132	1.827594298	6.560667076	9.27E-10	2.80E-08	11.87742221	ENSG00000111012.8	CYP27B1
ENSG00000111206	FOXM1	protein_coding	1.833379	3.495027416	7.044017124	7.37E-11	3.39E-09	14.33374358	ENSG00000111206.11	FOXM1
ENSG00000111674	ENO2	protein_coding	1.501616	2.617797717	4.63413323	8.03E-06	6.33E-05	3.150334112	ENSG00000111674.7	ENO2
ENSG00000113140	SPARC	protein_coding	2.850096	7.591609757	7.462818292	7.72E-12	4.91E-10	16.52823318	ENSG00000113140.9	SPARC
ENSG00000113721	PDGFRB	protein_coding	1.852532	3.755233311	5.250119866	5.42E-07	6.55E-06	5.727123969	ENSG00000113721.12	PDGFRB
ENSG00000115170	ACVR1	protein_coding	1.094397	3.55359692	6.168171336	6.77E-09	1.51E-07	9.951234591	ENSG00000115170.12	ACVR1
ENSG00000115414	FN1	protein_coding	3.687127	5.859589651	6.525393752	1.11E-09	3.30E-08	11.70168579	ENSG00000115414.17	FN1
ENSG00000115419	GLS	protein_coding	1.053487	3.099388662	5.771571796	4.73E-08	8.18E-07	8.074833113	ENSG00000115419.11	GLS
ENSG00000117650	NEK2	protein_coding	1.175191	2.340713861	5.544890093	1.39E-07	2.06E-06	7.036751623	ENSG00000117650.11	NEK2
ENSG00000117724	CENPF	protein_coding	1.228914	2.545251253	5.572375057	1.22E-07	1.86E-06	7.161219731	ENSG00000117724.11	CENPF
ENSG00000118193	KIF14	protein_coding	1.046961	1.41822898	5.908605379	2.44E-08	4.65E-07	8.714767342	ENSG00000118193.10	KIF14
ENSG00000118508	RAB32	protein_coding	1.499012	4.372269612	6.972543107	1.08E-10	4.70E-09	13.96510417	ENSG00000118508.4	RAB32
ENSG00000119630	PGF	protein_coding	1.466482	2.640785013	5.994825001	1.60E-08	3.21E-07	9.122018237	ENSG00000119630.12	PGF
ENSG00000119922	IFIT2	protein_coding	1.713589	3.018123195	4.662550233	7.12E-06	5.73E-05	3.26414539	ENSG00000119922.8	IFIT2
ENSG00000120254	MTHFD1L	protein_coding	1.582218	3.050237018	8.802281032	4.12E-15	7.90E-13	23.86977724	ENSG00000120254.14	MTHFD1L
ENSG00000121858	TNFSF10	protein_coding	1.232562	6.128233405	4.156993715	5.55E-05	0.000320552	1.317894287	ENSG00000121858.9	TNFSF10
ENSG00000122861	PLAU	protein_coding	3.653828	6.360749487	13.75985766	6.39E-28	1.16E-23	52.64029015	ENSG00000122861.14	PLAU
ENSG00000123130	ACOT9	protein_coding	1.224583	2.94244447	8.026314821	3.42E-13	3.72E-11	19.5637413	ENSG00000123130.15	ACOT9
ENSG00000123610	TNFAIP6	protein_coding	1.767917	2.490384724	5.191312077	7.08E-07	8.19E-06	5.471540044	ENSG00000123610.4	TNFAIP6
ENSG00000123892	RAB38	protein_coding	1.193043	5.344398951	3.978230287	0.00011	0.000575108	0.671149942	ENSG00000123892.10	RAB38
ENSG00000123975	CKS2	protein_coding	1.57385	5.473756502	6.088167531	1.01E-08	2.16E-07	9.566825222	ENSG00000123975.4	CKS2
ENSG00000123989	CHPF	protein_coding	1.352917	5.515887908	5.966928028	1.83E-08	3.64E-07	8.98986569	ENSG00000123989.12	CHPF
ENSG00000125871	MGME1	protein_coding	1.088405	3.65412871	6.934895952	1.32E-10	5.52E-09	13.7716605	ENSG00000125871.12	MGME1
ENSG00000126067	PSMB2	protein_coding	1.103105	5.106001987	7.887467538	7.42E-13	7.07E-11	18.80779126	ENSG00000126067.10	PSMB2
ENSG00000126709	IFI6	protein_coding	3.849665	8.412315474	9.042305753	1.03E-15	2.46E-13	25.22552475	ENSG00000126709.13	IFI6
ENSG00000127564	PKMYT1	protein_coding	1.510035	2.633602219	7.042816397	7.42E-11	3.40E-09	14.32753585	ENSG00000127564.15	PKMYT1
ENSG00000128245	YWHAH	protein_coding	1.073349	5.803579025	8.280759921	8.14E-14	1.10E-11	20.9613151	ENSG00000128245.13	YWHAH
ENSG00000128340	RAC2	protein_coding	1.879183	4.422416884	7.742904635	1.66E-12	1.36E-10	18.02608861	ENSG00000128340.13	RAC2

ENSG00000129474	AJUBA	protein_coding	1.47797	3.981042873	5.915960203	2.35E-08	4.52E-07	8.749369864	ENSG00000129474.14	AJUBA
ENSG00000132109	TRIM21	protein_coding	1.026603	4.165166538	4.685634818	6.46E-06	5.30E-05	3.356974324	ENSG00000132109.9	TRIM21
ENSG00000133101	CCNA1	protein_coding	1.198595	1.817800958	3.073667753	0.002535	0.007862777	-2.23880493	ENSG00000133101.8	CCNA1
ENSG00000133110	POSTN	protein_coding	3.409198	4.81724917	6.087784468	1.01E-08	2.16E-07	9.564991619	ENSG00000133110.13	POSTN
ENSG00000133818	RRAS2	protein_coding	1.110442	3.406537868	6.140140794	7.79E-09	1.70E-07	9.81622257	ENSG00000133818.11	RRAS2
ENSG00000134057	CCNB1	protein_coding	1.654385	4.52370206	6.595050265	7.76E-10	2.45E-08	12.04920451	ENSG00000134057.13	CCNB1
ENSG00000134321	RSAD2	protein_coding	2.247085	3.200965457	5.64604017	8.61E-08	1.38E-06	7.496740803	ENSG00000134321.10	RSAD2
ENSG00000134954	ETS1	protein_coding	1.388887	3.719016746	5.864311515	3.02E-08	5.61E-07	8.506923915	ENSG00000134954.13	ETS1
ENSG00000136235	GPNMB	protein_coding	1.402724	6.195745143	3.663226991	0.000351	0.00151558	-0.41269936	ENSG00000136235.14	GPNMB
ENSG00000137845	ADAM10	protein_coding	1.043086	2.954731039	7.527042445	5.43E-12	3.61E-10	16.86966728	ENSG00000137845.13	ADAM10
ENSG00000138448	ITGAV	protein_coding	1.427447	4.688781709	5.082246784	1.15E-06	1.25E-05	5.002717327	ENSG00000138448.10	ITGAV
ENSG00000138623	SEMA7A	protein_coding	1.12354	2.316269605	5.071009353	1.21E-06	1.30E-05	4.954800447	ENSG00000138623.8	SEMA7A
ENSG00000140945	CDH13	protein_coding	1.264007	2.699294897	5.115245326	9.96E-07	1.10E-05	5.143845497	ENSG00000140945.14	CDH13
ENSG00000142192	APP	protein_coding	1.619119	7.123213494	7.922662624	6.10E-13	6.03E-11	18.99894262	ENSG00000142192.19	APP
ENSG00000143387	CTSK	protein_coding	1.680091	5.055932171	4.059486383	8.09E-05	0.000441555	0.962344426	ENSG00000143387.11	CTSK
ENSG00000143476	DTL	protein_coding	1.135614	2.130089857	5.880044713	2.80E-08	5.27E-07	8.580642096	ENSG00000143476.16	DTL
ENSG00000144476	ACKR3	protein_coding	1.79685	4.651295636	5.965123648	1.85E-08	3.67E-07	8.98133062	ENSG00000144476.5	ACKR3
ENSG00000145386	CCNA2	protein_coding	1.467013	3.495783709	6.055237141	1.19E-08	2.47E-07	9.409442861	ENSG00000145386.8	CCNA2
ENSG00000145604	SKP2	protein_coding	1.113458	3.208105087	5.164548879	7.99E-07	9.12E-06	5.355869459	ENSG00000145604.14	SKP2
ENSG00000146070	PLA2G7	protein_coding	1.706559	2.029800711	6.554298298	9.58E-10	2.89E-08	11.84565503	ENSG00000146070.15	PLA2G7
ENSG00000146410	MTFR2	protein_coding	1.009628	1.509723766	6.582541306	8.28E-10	2.57E-08	11.98665372	ENSG00000146410.10	MTFR2
ENSG00000146648	EGFR	protein_coding	1.159434	4.655476666	3.358248867	0.001007	0.003647439	-1.3912966	ENSG00000146648.14	EGFR
ENSG00000147689	FAM83A	protein_coding	2.062363	5.210692137	5.010258626	1.59E-06	1.63E-05	4.697027492	ENSG00000147689.15	FAM83A
ENSG00000147889	CDKN2A	protein_coding	1.480343	2.203188343	3.085391187	0.002444	0.007629066	-2.20517665	ENSG00000147889.15	CDKN2A
ENSG00000149557	FEZ1	protein_coding	1.87989	2.638908192	6.403589485	2.07E-09	5.52E-08	11.09874978	ENSG00000149557.11	FEZ1
ENSG00000149948	HMGA2	protein_coding	1.459753	1.429942572	4.296816763	3.19E-05	0.000201441	1.839176974	ENSG00000149948.12	HMGA2
ENSG00000150093	ITGB1	protein_coding	1.342866	5.699572367	4.566543405	1.06E-05	8.04E-05	2.881692341	ENSG00000150093.17	ITGB1
ENSG00000150630	VEGFC	protein_coding	2.076654	3.194453261	6.156407589	7.18E-09	1.59E-07	9.894530403	ENSG00000150630.3	VEGFC
ENSG00000151651	ADAM8	protein_coding	1.465605	2.770404051	5.606087182	1.04E-07	1.62E-06	7.314421929	ENSG00000151651.14	ADAM8
ENSG00000152661	GJA1	protein_coding	2.013283	7.237213372	6.204269388	5.66E-09	1.30E-07	10.12562117	ENSG00000152661.7	GJA1
ENSG00000154096	THY1	protein_coding	2.255288	3.509518456	6.332596489	2.97E-09	7.46E-08	10.75018767	ENSG00000154096.12	THY1
ENSG00000154764	WNT7A	protein_coding	1.763291	1.77288264	7.233234593	2.68E-11	1.43E-09	15.31814145	ENSG00000154764.5	WNT7A
ENSG00000155366	RHOC	protein_coding	1.218986	5.300862722	6.181958315	6.32E-09	1.43E-07	10.01776991	ENSG00000155366.15	RHOC
ENSG00000156234	CXCL13	protein_coding	2.292777	3.149654455	5.12731898	9.44E-07	1.05E-05	5.19563842	ENSG00000156234.7	CXCL13
ENSG00000157227	MMP14	protein_coding	2.176634	6.945065756	9.340386335	1.81E-16	5.39E-14	26.92192559	ENSG00000157227.11	MMP14

ENSG00000157456	CCNB2	protein_coding	1.581072	3.370543527	7.099178686	5.50E-11	2.62E-09	14.61946585	ENSG00000157456.6	CCNB2
ENSG00000157601	MX1	protein_coding	1.340134	4.612083838	3.636617339	0.000385	0.001638779	-0.50090337	ENSG00000157601.12	MX1
ENSG00000160691	SHC1	protein_coding	1.274053	5.307403405	7.517256794	5.73E-12	3.78E-10	16.81756407	ENSG00000160691.17	SHC1
ENSG00000161638	ITGA5	protein_coding	2.873455	4.510985585	9.997833816	3.75E-18	1.63E-15	30.70389708	ENSG00000161638.9	ITGA5
ENSG00000162063	CCNF	protein_coding	1.084275	2.342128121	6.536626057	1.05E-09	3.14E-08	11.75759215	ENSG00000162063.11	CCNF
ENSG00000162493	PDPN	protein_coding	2.898971	5.134072495	10.75231507	4.19E-20	2.93E-17	35.09163814	ENSG00000162493.15	PDPN
ENSG00000164171	ITGA2	protein_coding	1.014926	3.799005377	3.68555387	0.000324	0.001416847	-0.33828238	ENSG00000164171.9	ITGA2
ENSG00000164251	F2RL1	protein_coding	1.249643	4.121280142	5.387138156	2.89E-07	3.84E-06	6.330048811	ENSG00000164251.4	F2RL1
ENSG00000164754	RAD21	protein_coding	1.006708	5.103782737	5.789628158	4.33E-08	7.60E-07	8.158631014	ENSG00000164754.11	RAD21
ENSG00000164924	YWHAZ	protein_coding	1.013657	7.549848453	4.995467115	1.70E-06	1.72E-05	4.634591904	ENSG00000164924.16	YWHAZ
ENSG00000164933	SLC25A32	protein_coding	1.007866	3.126418476	6.791278959	2.80E-10	1.05E-08	13.03844083	ENSG00000164933.10	SLC25A32
ENSG00000165304	MELK	protein_coding	1.60879	3.033256278	6.33876817	2.88E-09	7.26E-08	10.78040453	ENSG00000165304.6	MELK
ENSG00000165949	IFI27	protein_coding	2.539784	7.223281138	7.371303325	1.27E-11	7.53E-10	16.04388562	ENSG00000165949.11	IFI27
ENSG00000166851	PLK1	protein_coding	1.760484	3.291991707	8.151643528	1.69E-13	2.02E-11	20.25021095	ENSG00000166851.13	PLK1
ENSG00000166923	GREM1	protein_coding	1.878466	2.166910098	5.830247229	3.56E-08	6.47E-07	8.347725026	ENSG00000166923.9	GREM1
ENSG00000167601	AXL	protein_coding	1.325365	3.380091017	4.032557662	8.96E-05	0.000482498	0.865323986	ENSG00000167601.10	AXL
ENSG00000168040	FADD	protein_coding	1.415733	3.437265105	4.978530281	1.83E-06	1.82E-05	4.563258936	ENSG00000168040.4	FADD
ENSG00000169071	ROR2	protein_coding	1.010724	1.483987235	3.75088626	0.000256	0.001162648	-0.11839271	ENSG00000169071.13	ROR2
ENSG00000169245	CXCL10	protein_coding	2.689915	5.410626147	3.942106849	0.000126	0.000644026	0.543201652	ENSG00000169245.5	CXCL10
ENSG00000169398	PTK2	protein_coding	1.037828	3.197530168	6.844032504	2.12E-10	8.45E-09	13.30688521	ENSG00000169398.18	PTK2
ENSG00000170312	CDK1	protein_coding	1.34525	3.32954665	6.233325345	4.89E-09	1.15E-07	10.26640699	ENSG00000170312.14	CDK1
ENSG00000170581	STAT2	protein_coding	1.509349	4.540789879	7.577133345	4.13E-12	2.88E-10	17.13681769	ENSG00000170581.12	STAT2
ENSG00000170961	HAS2	protein_coding	1.33165	1.732789899	4.452757408	1.70E-05	0.000118994	2.436074315	ENSG00000170961.6	HAS2
ENSG00000171608	PIK3CD	protein_coding	1.280079	2.738152376	7.5439552	4.95E-12	3.36E-10	16.95978549	ENSG00000171608.14	PIK3CD
ENSG00000172061	LRRC15	protein_coding	1.625393	2.148502653	3.612799197	0.000419	0.001756075	-0.57940273	ENSG00000172061.8	LRRC15
ENSG00000172156	CCL11	protein_coding	1.507496	1.46106456	4.629017677	8.20E-06	6.46E-05	3.129900303	ENSG00000172156.3	CCL11
ENSG00000172243	CLEC7A	protein_coding	1.282548	2.387200146	5.029964081	1.46E-06	1.52E-05	4.780404334	ENSG00000172243.16	CLEC7A
ENSG00000172331	BPGM	protein_coding	1.39215	4.654324685	5.394608054	2.80E-07	3.73E-06	6.363213222	ENSG00000172331.10	BPGM
ENSG00000173207	CKS1B	protein_coding	1.344473	3.587351396	5.935096353	2.14E-08	4.19E-07	8.839521089	ENSG00000173207.11	CKS1B
ENSG00000174744	BRMS1	protein_coding	1.18629	5.222774013	6.462604744	1.53E-09	4.33E-08	11.39011367	ENSG00000174744.12	BRMS1
ENSG00000175166	PSMD2	protein_coding	1.090391	6.259686819	7.17929904	3.58E-11	1.81E-09	15.03631648	ENSG00000175166.15	PSMD2
ENSG00000176170	SPHK1	protein_coding	1.542191	3.989986265	6.627881524	6.55E-10	2.14E-08	12.21367179	ENSG00000176170.12	SPHK1
ENSG00000176692	FOXC2	protein_coding	1.081552	1.415954163	4.250630166	3.84E-05	0.000234389	1.665512502	ENSG00000176692.5	FOXC2
ENSG00000176890	TYMS	protein_coding	1.183995	3.312973323	4.826627107	3.54E-06	3.19E-05	3.931130226	ENSG00000176890.14	TYMS
ENSG00000177697	CD151	protein_coding	1.229321	6.092816548	6.649847041	5.85E-10	1.93E-08	12.32394464	ENSG00000177697.16	CD151

ENSG00000178999	AURKB	protein_coding	1.715688	3.495038154	7.10165906	5.42E-11	2.59E-09	14.63233811	ENSG00000178999.11	AURKB
ENSG00000179051	RCC2	protein_coding	1.146235	5.818876008	7.483128188	6.91E-12	4.49E-10	16.6360722	ENSG00000179051.12	RCC2
ENSG00000179218	CALR	protein_coding	1.007518	8.595933721	7.214447777	2.96E-11	1.56E-09	15.21986721	ENSG00000179218.12	CALR
ENSG00000181104	F2R	protein_coding	1.534142	3.588612107	5.569148332	1.24E-07	1.88E-06	7.146586908	ENSG00000181104.6	F2R
ENSG00000182871	COL18A1	protein_coding	1.234043	4.564310921	4.755638251	4.80E-06	4.12E-05	3.640510692	ENSG00000182871.13	COL18A1
ENSG00000183486	MX2	protein_coding	1.303365	2.379451496	4.555048584	1.12E-05	8.35E-05	2.836295494	ENSG00000183486.11	MX2
ENSG00000185338	SOCS1	protein_coding	1.442073	2.978971006	6.319056895	3.18E-09	7.87E-08	10.68395419	ENSG00000185338.4	SOCS1
ENSG00000186891	TNFRSF18	protein_coding	1.235543	3.046001833	3.509506344	0.000602	0.002376801	-0.91485887	ENSG00000186891.12	TNFRSF18
ENSG00000196230	TUBB	protein_coding	1.135922	7.930597437	7.227988312	2.75E-11	1.46E-09	15.29068637	ENSG00000196230.11	TUBB
ENSG00000196924	FLNA	protein_coding	1.342278	7.083666464	5.176861696	7.56E-07	8.67E-06	5.409035121	ENSG00000196924.13	FLNA
ENSG00000197405	C5AR1	protein_coding	1.089096	2.023633048	4.283398485	3.37E-05	0.000210534	1.788574728	ENSG00000197405.6	C5AR1
ENSG00000197461	PDGFA	protein_coding	1.305418	2.805820623	6.349021968	2.73E-09	6.95E-08	10.83064359	ENSG00000197461.12	PDGFA
ENSG00000203485	INF2	protein_coding	1.0759	3.830654533	6.967116413	1.11E-10	4.81E-09	13.93718877	ENSG00000203485.11	INF2
ENSG00000205542	TMSB4X	protein_coding	1.002706	8.84036608	5.071883201	1.21E-06	1.30E-05	4.958523952	ENSG00000205542.9	TMSB4X
ENSG00000213190	MLLT11	protein_coding	1.028556	2.271320367	3.787652938	0.000224	0.00103539	0.006741884	ENSG00000213190.3	MLLT11
ENSG00000214078	CPNE1	protein_coding	1.299472	5.252510386	5.613567571	1.00E-07	1.57E-06	7.348495299	ENSG00000214078.10	CPNE1
ENSG00000233608	TWIST2	protein_coding	1.006187	2.160164001	4.422056246	1.93E-05	0.000132318	2.317285101	ENSG00000233608.3	TWIST2
ENSG00000240065	PSMB9	protein_coding	1.415997	4.559261096	4.493394857	1.44E-05	0.000103195	2.594257896	ENSG00000240065.6	PSMB9
ENSG00000271503	CCL5	protein_coding	1.488438	4.885192373	3.45887546	0.000716	0.002747335	-1.07631003	ENSG00000271503.4	CCL5
ENSG00000277586	NEFL	protein_coding	1.543607	3.120964337	3.6749908	0.000336	0.001464377	-0.37353625	ENSG00000277586.1	NEFL
