# Supplementary material for: Repurposing Dihydroartemisinin to Combat Oral Squamous Cell Carcinoma, Associated with Mitochondrial Dysfunction and Oxidative Stress
Source: Oxid Med Cell Longev. 2023 Feb 16;2023:9595201. doi: 10.1155/2023/9595201 (PMC10239307; doi:10.1155/2023/9595201)
Supplement: Supplementary 12 — Supplementary Table 4: list of all antibodies in the study. [file 9595201.f12.DOCX]

**Primary and Secondary Antibodies**

| **Protein** | **Protein Mass (kDa)** | **Primary**  **Antibody Details** | **Primary**  **Antibody**  **Dilution** | **Secondary**  **Antibody**  **Details** |
| --- | --- | --- | --- | --- |
| RPS6 | 32 kDa | Cell Signaling # 2217 | 1:1000 | Rabbit |
| p-rps6 (Ser235/236) | 32 kDa | Cell Signaling # 2211 | 1:1000 | Rabbit |
| P-RPS6 (Ser240/244) | 32 kDa | Cell Signaling # 2215 | 1:1000 | Rabbit |
| p-mTOR (Ser2448) | 289 kDa | Cell Signaling # 5536 | 1:1000 | Rabbit |
| Bcl-2 | 26 kDa | Cell Signaling #15071 | 1:1000 | Mouse |
| Bax | 20 kDa | Cell Signaling #2772 | 1:1000 | Rabbit |
| Cytochrome c | 14 kDa | Cell Signaling #11940 | 1:2000 | Rabbit |
| γ-H2AX (Ser139) | 15 kDa | Cell Signaling # 9718 | 1:1000 | Rabbit |
| γ-H2AX  (Ser139) |  | abcam （ab11175） | 1:200 | DyLight 594  Rabbit |
| Caspase-3 | 17,18,35 kDa | Cell Signaling # 9662 | 1:1000 | Rabbit |
| AIF | 67 kDa | abcam （ab32516） | 1:1000 | Rabbit |
| AIF |  | abcam （ab32516） | 1:500 | DyLight 594  Rabbit |
| LC3A/B | 14,16 kDa | Cell Signaling #12741 | 1:1000 | Rabbit |
| Beclin1 | 60 kDa | Cell Signaling #3495 | 1:1000 | Rabbit |
| Mitofusin-1 | 82 kDa | Cell Signaling #14739 | 1:1000 | Rabbit |
| β-Tubulin | 50 kDa | Cell Signaling #2128 | 1:1000 | Rabbit |
| β-Actin | 42 kDa | Cell Signaling #4970 | 1:1000 | Rabbit |
