# Supplementary material for: Repurposing Dihydroartemisinin to Combat Oral Squamous Cell Carcinoma, Associated with Mitochondrial Dysfunction and Oxidative Stress
Source: Oxid Med Cell Longev. 2023 Feb 16;2023:9595201. doi: 10.1155/2023/9595201 (PMC10239307; doi:10.1155/2023/9595201)
Supplement: Supplementary 13 — Supplementary Table 5: antibody validation statements and sources in the study. [file 9595201.f13.pdf]

Revision 1

Store at -20C

#4970

β-Actin (13E5) Rabbit mAb

Cell Signaling TECHNOLOGY®

Orders: 877-616-CELL (2355)
orders@cellsignal.com

Support: 877-678-TECH (8324)

Web: info@cellsignal.com
www.cellsignal.com

3 Trask Lane | Danvers | Massachusetts | 01923 | USA

For Research Use Only. Not For Use In Diagnostic Procedures.

Applications:	Reactivity:	Sensitivity:	MW (kDa):	Source/Isotype:	UniProt ID:	Entrez-Gene Id:
WB, IHC-P, IF-IC, F	H M R Mk B Pg	Endogenous	45	Rabbit IgG	P60709	60

Product Usage Information

Application	Dilution
Western Blotting	1:1000
Immunohistochemistry (Paraffin)	1:50 - 1:200
Immunofluorescence (Immunocytochemistry)	1:100 - 1:400
Flow Cytometry	1:100 - 1:400

Storage

Supplied in 10 mM sodium HEPES (pH 7.5), 150 mM NaCl, 100 µg/ml BSA, 50% glycerol and less than 0.02% sodium azide. Store at –20°C. Do not aliquot the antibody.

Specificity / Sensitivity

β-Actin (13E5) Rabbit mAb detects endogenous levels of total β-actin protein. Depsite the high sequence identity between the cytoplasmic actin isoforms, β-actin and cytoplasmic γ-actin, β-Actin (13E5) Rabbit mAb #4970 does not cross-react with cytoplasmic γ-actin, or any other actin isoforms.

Species Reactivity:
Human, Mouse, Rat, Monkey, Bovine, Pig

Species predicted to react based on 100% sequence homology:
Hamster, Chicken, Dog, Horse

Source / Purification

Monoclonal antibody is produced by immunizing animals with a synthetic peptide corresponding to residues near the amino-terminus of human β-actin protein.

Background

Actin, a ubiquitous eukaryotic protein, is the major component of the cytoskeleton. At least six isoforms are known in mammals. Nonmuscle β- and γ-actin, also known as cytoplasmic actin, are ubiquitously expressed, controlling cell structure and motility (1). While all actin isoforms are highly homologous, cytoplasmic β- and γ-actin protein sequences differ by only four biochemically similar amino acids (2). For this reason, antibodies raised to β-actin may cross-react with γ-actin, and vice versa. α-cardiac and α-skeletal actin are expressed in striated cardiac and skeletal muscles, respectively; two smooth muscle actins, α- and γ-actin, are found primarily in vascular smooth muscle and enteric smooth muscle, respectively. These actin isoforms regulate the contractile potential of muscle cells (1). Actin exists mainly as a fibrous polymer, F-actin. In response to cytoskeletal reorganizing signals during processes such as cytokinesis, endocytosis, or stress, cofilin promotes fragmentation and depolymerization of F-actin, resulting in an increase in the monomeric globular form, G-actin (3). The ARP2/3 complex stabilizes F-actin fragments and promotes formation of new actin filaments (3). Research studies have shown that actin is hyperphosphorylated in primary breast tumors (4). Cleavage of actin under apoptotic conditions has been observed *in vitro* and in cardiac and skeletal muscle, as shown in research studies (5-7). Actin cleavage by caspase-3 may accelerate ubiquitin/proteasome-dependent muscle proteolysis (7).

1. Herman, I.M. (1993) *Curr. Opin. Cell Biol.* 5, 48-55.
2. Perrin, B.J. and Ervasti, J.M. (2010) *Cytoskeleton (Hoboken)* 67, 630-4.
3. Condeelis, J. (2001) *Trends Cell Biol* 11, 288-93.
4. Lim, Y.P. et al. (2004) *Clin Cancer Res* 10, 3980-7.
5. Kayalar, C. et al. (1996) *Proc Natl Acad Sci U S A* 93, 2234-8.
6. Communal, C. et al. (2002) *Proc Natl Acad Sci U S A* 99, 6252-6.
7. Du, J. et al. (2004) *J Clin Invest* 113, 115-23.

Species reactivity is determined by testing in at least one approved application (e.g., western blot).

IMPORTANT: For western blots, incubate membrane with diluted primary antibody in 5% w/v BSA, 1X TBS, 0.1% Tween® 20 at 4°C with gentle shaking, overnight.

APPLICATIONS KEY **WB:** Western Blot **IP:** Immunoprecipitation **IHC:** Immunohistochemistry **ChIP:** Chromatin Immunoprecipitation **IF:** Immunofluorescence **F:** Flow Cytometry **E-P:** ELISA-Peptide

CROSS-REACTIVITY KEY **H:** human **M:** mouse **R:** rat **Hm:** hamster **Mk:** monkey **Vir:** virus **Mi:** mink **C:** chicken **Dm:** D. melanogaster **X:** Xenopus **Z:** zebrafish **B:** bovine **Dg:** dog **Pg:** pig **Sc:** S. cerevisiae **Ce:** C. elegans **Hr:** horse **All:** all species expected

Cell Signaling Technology is a trademark of Cell Signaling Technology, Inc.
DRAQ5 is a registered trademark of Biostatus Limited.

Revision 1

#4970

β-Actin (13E5) Rabbit mAb

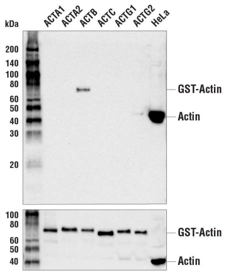

Western blot analysis of recombinant Actin isoforms using β-Actin (13E5) Rabbit mAb (upper) and Pan-Actin Antibody #4968 (lower).

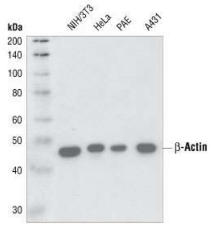

Western blot analysis of cell extracts from various cell lines using beta-Actin (13E5) Rabbit mAb.

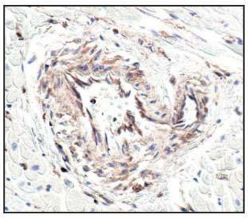

Immunohistochemical analysis of paraffin-embedded human heart using beta-Actin (13E5) Rabbit mAb. Note the lack of staining of cardiac actin.

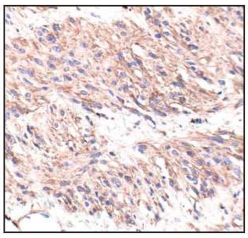

Immunohistochemical analysis of paraffin-embedded human leiomyoma using beta-Actin (13E5) Rabbit mAb.

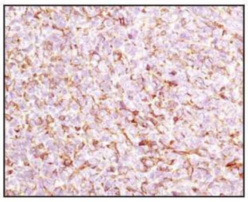

Immunohistochemical analysis of paraffin-embedded 4T1 syngeneic mouse tumor using β-actin (13E5) Rabbit mAb.

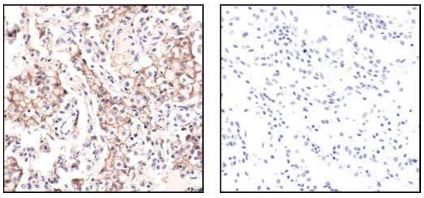

Immunohistochemical analysis of paraffin-embedded human lung carcinoma using beta-Actin (13E5) Rabbit mAb in the presence of control peptide (left) or beta-Actin Blocking Peptide #1025 (right).

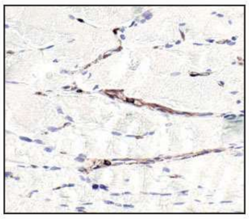

Immunohistochemical analysis of paraffin-embedded human skeletal muscle using beta-Actin (13E5) Rabbit mAb. Note the lack of staining of skeletal muscle actin.

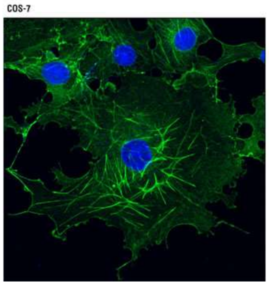

Confocal immunofluorescent analysis of COS-7 cells using β-Actin (13E5) Rabbit mAb (green). Blue pseudocolor = DRAQ5® #4084 (fluorescent DNA dye).

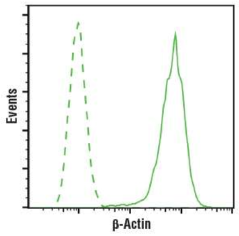

Flow cytometric analysis of HeLa cells using β-Actin (13E5) Rabbit mAb (solid line) compared to concentration-matched Rabbit (DA1E) mAb IgG XP® Isotype Control #3900 (dashed line). Anti-rabbit IgG (H+L), F(ab')2 Fragment (Alexa Fluor® 488 Conjugate) #4412 was used as a secondary antibody.

#4970

β-Actin (13E5) Rabbit mAb

限制使用

除非 CST 的合法授书代表以书面形式书行明确同意，否书以下条款适用于 CST、其关书方或分书商提供的书品。 任何书充本条款或与本条款不同的客书条款和条件，除非书 CST 的合法授书代表以书面形式书独接受， 否书均被拒书，并且无效。

专品专有"专供研究使用"的专专或专似的专专声明， 且未专得美国食品和专品管理局或其他外国或国内专管机专专专任何用途的批准、准专或专可。客专不得将任何专品用于任何专断或治专目的， 或以任何不符合专专声明的方式使用专品。CST 专售或专可的专品提供专作专最专用专的客专，且专用于研专用途。将专品用于专断、专防或治专目的， 或专专售（专独或作专专成）或其他商专目的而专专专品，均需要 CST 的专独专可。客专：(a) 不得专独或与其他材料专合向任何第三方出售、专可、出借、捐专或以其他方式专专或提供任何专品，或使用专品制造任何商专专品，(b) 不得复制、修改、逆向工程、反专专、 反专专专品或以其他方式专专专专专品的基专专专或技专，或使用专品开专任何与 CST 的专品或服专专争的专品或服专， (c) 不得更改或专除专品上的任何商专、商品名称、徽专、专利或版专声明或专专，(d) 只能根据 CST 的专品专售条款和任何适用文档使用专品， (e) 专遵守客专与专品一起使用的任何第三方专品或服专的任何专可、服专条款或专似专专

Orders: 877-616-CELL (2355) • orders@cellsignal.com • Support: 877-678-TECH (8324) • info@cellsignal.com • Web: www.cellsignal.com

abcam

Product datasheet

Anti-AIF antibody [E20] - Mitochondrial Marker ab32516

敲除验证 重组 RabMAb

★★★★☆ 5 Abreviews 59 References 7 图像

概述

**产品名称**	Anti-AIF抗体[E20] - Mitochondrial Marker
**描述**	兔单克隆抗体[E20] to AIF - Mitochondrial Marker
**宿主**	Rabbit
**经测试应用**	**适用于:** Flow Cyt (Intra), ICC/IF, IHC-Fr, WB, IHC-P, IP
**种属反应性**	**与反应:** Mouse, Rat, Human
**免疫原**	Synthetic peptide. This information is proprietary to Abcam and/or its suppliers.
**阳性对照**	WB: HEK-293T and K562 cell lysate. IHC-P: Human cervical carcinoma tissue.
**常规说明**	This product is a recombinant monoclonal antibody, which offers several advantages including:<br>- High batch-to-batch consistency and reproducibility<br>- Improved sensitivity and specificity<br>- Long-term security of supply<br>- Animal-free production<br>For more information see here.<br>Our RabMAb® technology is a patented hybridoma-based technology for making rabbit monoclonal antibodies. For details on our patents, please refer to RabMAb® patents.

性能

**形式**	Liquid
**存放说明**	Shipped at 4°C. Store at +4°C short term (1-2 weeks). Upon delivery aliquot. Store at -20°C. Avoid freeze / thaw cycle.
**存储溶液**	pH: 7.20<br>Preservative: 0.01% Sodium azide<br>Constituents: 49% PBS, 50% Glycerol (glycerin, glycerine), 0.05% BSA
**克隆**	单克隆
**克隆编号**	E20
**同种型**	IgG

应用

[illegible]

The Abpromise guarantee Abpromise™**承诺保证使用**ab32516**于以下的**经测试应用

“应用说明”**部分** 下显示的仅为**推荐的起始稀释度**;实际**最佳的稀释度**/浓度应**由使用者检定**。

应用	Ab评论	说明
**Flow Cyt (Intra)**		1/50.<br>ab172730 - Rabbit monoclonal IgG, is suitable for use as an isotype control with this antibody.
**ICC/IF**		1/500.
**IHC-Fr**	★★★★☆ (1)	Use at an assay dependent concentration. PubMed: 23118224
**WB**	★★★★★ (3)	1/1000. Detects a band of approximately 67 kDa (predicted molecular weight: 67 kDa).
**IHC-P**	★★★★☆ (1)	Use at an assay dependent concentration. Perform heat mediated antigen retrieval with citrate buffer pH 6 before commencing with IHC staining protocol.
**IP**		Use at an assay dependent concentration.

靶标

功能 Probable oxidoreductase that has a dual role in controlling cellular life and death; during apoptosis, it is translocated from the mitochondria to the nucleus to function as a proapoptotic factor in a caspase-independent pathway, while in normal mitochondria, it functions as an antiapoptotic factor via its oxidoreductase activity. The soluble form (AIFsol) found in the nucleus induces 'parthanatos' i.e., caspase-independent fragmentation of chromosomal DNA. Interacts with EIF3G,and thereby inhibits the EIF3 machinery and protein synthesis, and activates casapse-7 to amplify apoptosis. Plays a critical role in caspase-independent, pyknotic cell death in hydrogen peroxide-exposed cells. Binds to DNA in a sequence-independent manner.

疾病相关 Defects in AIFM1 are the cause of combined oxidative phosphorylation deficiency type 6 (COXPD6) [MIM:300816]. It is a mitochondrial disease resulting in a neurodegenerative disorder characterized by psychomotor delay, hypotonia, areflexia, muscle weakness and wasting.

序列相似性 Belongs to the FAD-dependent oxidoreductase family.

翻译后修饰 Under normal conditions, a 54-residue N-terminal segment is first proteolytically removed during or just after translocation into the mitochondrial intermembrane space (IMS) by the mitochondrial processing peptidase (MPP) to form the inner-membrane-anchored mature form (AIFmit). During apoptosis, it is further proteolytically processed at amino-acid position 101 leading to the generation of the mature form, which is confined to the mitochondrial IMS in a soluble form (AIFsol). AIFsol is released to the cytoplasm in response to specific death signals, and translocated to the nucleus, where it induces nuclear apoptosis in a caspase-independent manner.

细胞定位 Mitochondrion intermembrane space. Mitochondrion inner membrane. Cytoplasm. Nucleus. Cytoplasm > perinuclear region. Proteolytic cleavage during or just after translocation into the mitochondrial intermembrane space (IMS) results in the formation of an inner-membrane-anchored mature form (AIFmit). During apoptosis, further proteolytic processing leads to a mature form, which is confined to the mitochondrial IMS in a soluble form (AIFsol). AIFsol is released to

the cytoplasm in response to specific death signals, and translocated to the nucleus, where it induces nuclear apoptosis. Colocalizes with EIF3G in the nucleus and perinuclear region.

图片

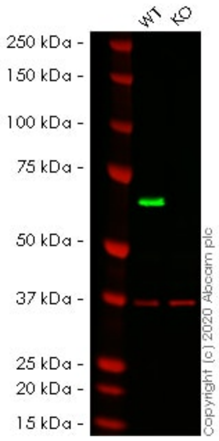

Western blot - Anti-AIF antibody [E20] - Mitochondrial Marker (ab32516)

All lanes : Anti-AIF antibody [E20] - Mitochondrial Marker (ab32516) at 1/1000 dilution

Lane 1 : Wild-type HEK-293T cell lysate
Lane 2 : AIFM1 knockout HEK-293T cell lysate

Lysates/proteins at 20 µg per lane.

Performed under reducing conditions.

Predicted band size: 67 kDa
Observed band size: 67 kDa

Lanes 1- 2: Merged signal (red and green). Green - ab32516 observed at 67 kDa. Red - Anti-GAPDH antibody [6C5] - Loading Control (ab8245) observed at 37 kDa.

ab32516 was shown to react with AIF in wild-type HEK-293T cells in western blot. Loss of signal was observed when knockout cell line ab266347 (knockout cell lysate ab256834) was used. Wild-type HEK-293T and AIFM1 knockout HEK-293T cell lysates were subjected to SDS-PAGE. Membrane was blocked for 1 hour at room temperature in 0.1% TBST with 3% non-fat dried milk. ab32516 and Anti-GAPDH antibody [6C5] - Loading Control (ab8245) overnight at 4°C at a 1 in 1000 dilution and a 1 in 20000 dilution respectively. Blots were developed with Goat anti-Rabbit IgG H&L (IRDye®800CW) preadsorbed (ab216773) and Goat anti-Mouse IgG H&L (IRDye®680RD) preadsorbed (ab216776) secondary antibodies at 1 in 20000 dilution for 1 hour at room temperature before imaging.

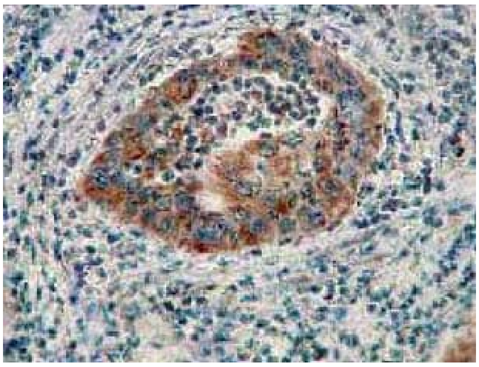

Immunohistochemistry (Formalin/PFA-fixed paraffin-embedded sections) - Anti-AIF antibody [E20] - Mitochondrial Marker (ab32516)

Ab32516, at a 1/500 dilution, staining AIF in paraffin embedded human cervical carcinoma tissue by Immunohistochemistry.

Perform heat mediated antigen retrieval with citrate buffer pH 6 before commencing with IHC staining protocol.

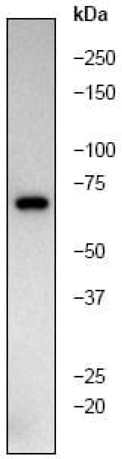

Western blot - Anti-AIF antibody [E20] - Mitochondrial Marker (ab32516)

Anti-AIF antibody [E20] - Mitochondrial Marker (ab32516) at 1/1000 dilution + K562 cell lysate

Predicted band size: 67 kDa

Observed band size: 67 kDa

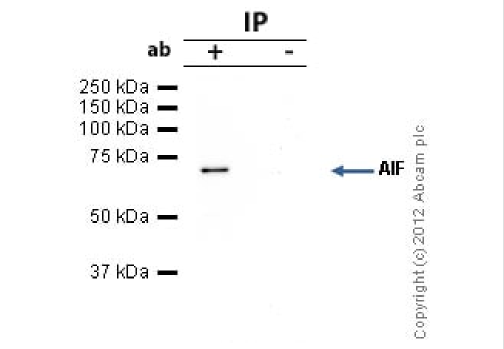

Immunoprecipitation - Anti-AIF antibody [E20] - Mitochondrial Marker (ab32516)

AIF was immunoprecipitated using 0.5mg K562 whole cell extract, 5µg of Rabbit monoclonal to AIF and 50µl of protein G magnetic beads (+). No antibody was added to the control (-).
The antibody was incubated under agitation with Protein G beads for 10min, K562 whole cell extract lysate diluted in RIPA buffer was added to each sample and incubated for a further 10min under agitation.
Proteins were eluted by addition of 40µl SDS loading buffer and incubated for 10min at 70°C; 10µl of each sample was separated on a SDS PAGE gel, transferred to a nitrocellulose membrane, blocked with 5% BSA and probed with ab32516.
Secondary: Mouse monoclonal [SB62a] Secondary Antibody to Rabbit IgG light chain (HRP) (ab99697).
Band: 67kDa: AIF

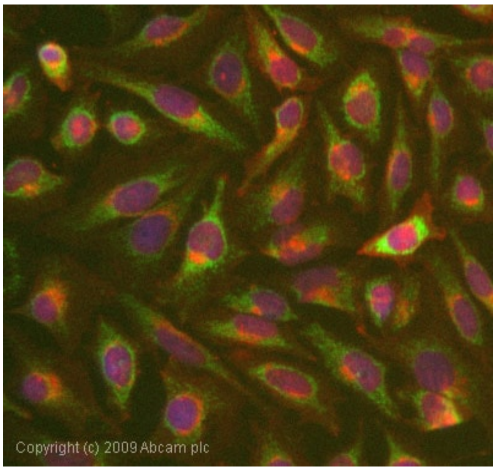

Immunocytochemistry/ Immunofluorescence - Anti-AIF antibody [E20] - Mitochondrial Marker (ab32516)

ICC/IF image of ab32516 stained HeLa cells. The cells were 4% PFA fixed (10 min) and then incubated in 1%BSA / 10% normal goat serum / 0.3M glycine in 0.1% PBS-Tween for 1h to permeabilise the cells and block non-specific protein-protein interactions. The cells were then incubated with the antibody (ab32516, 1µg/ml) overnight at +4°C. The secondary antibody (green) was Alexa Fluor® 488 goat anti-rabbit IgG (H+L) used at a 1/1000 dilution for 1h. Alexa Fluor® 594 WGA was used to label plasma membranes (red) at a 1/200 dilution for 1h. DAPI was used to stain the cell nuclei (blue) at a concentration of 1.43µM.

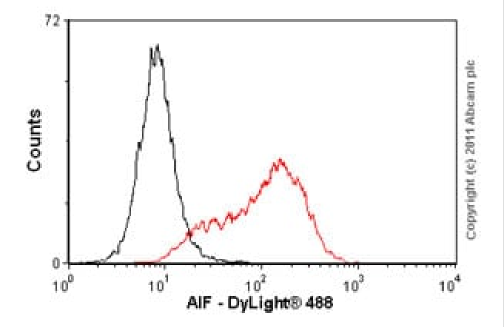

Flow Cytometry (Intracellular) - Anti-AIF antibody [E20] - Mitochondrial Marker (ab32516)

Overlay histogram showing K562 cells stained with ab32516 (red line). The cells were fixed with 4% paraformaldehyde (10 min) and then permeabilized with 0.1% PBS-Tween for 20 min. The cells were then incubated in 1x PBS / 10% normal goat serum / 0.3M glycine to block non-specific protein-protein interactions followed by the antibody (ab32516, 1/50 dilution) for 30 min at 22°C. The secondary antibody used was DyLight® 488 goat anti-rabbit IgG (H+L) (ab96899) at 1/500 dilution for 30 min at 22°C. Isotype control antibody (black line) was rabbit monoclonal IgG (1µg/1x10^6 cells) used under the same conditions. Acquisition of >5,000 events was performed. This antibody gave a positive signal in K562 cells fixed with methanol (5 min)/permeabilized with 0.1% PBS-Tween used under the same conditions.

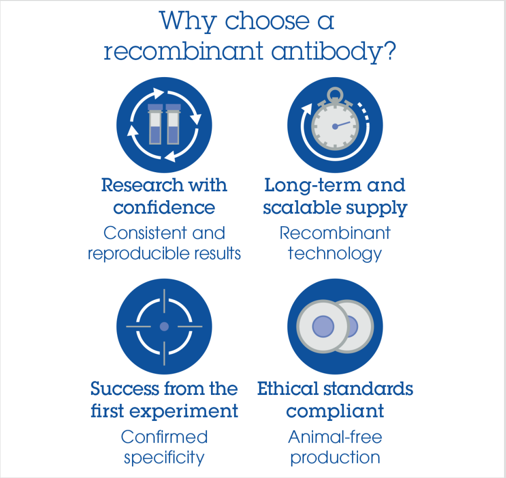

Anti-AIF antibody [E20] - Mitochondrial Marker (ab32516)

Please note: All products are "FOR RESEARCH USE ONLY. NOT FOR USE IN DIAGNOSTIC PROCEDURES"

Our Abpromise to you: Quality guaranteed and expert technical support

- Replacement or refund for products not performing as stated on the datasheet
- Valid for 12 months from date of delivery
- Response to your inquiry within 24 hours

- We provide support in Chinese, English, French, German, Japanese and Spanish
- Extensive multi-media technical resources to help you
- We investigate all quality concerns to ensure our products perform to the highest standards

If the product does not perform as described on this datasheet, we will offer a refund or replacement. For full details of the Abpromise, please visit https://www.abcam.cn/abpromise or contact our technical team.

Terms and conditions

- Guarantee only valid for products bought direct from Abcam or one of our authorized distributors

Revision 1

Store at -20C

#2772

Bax Antibody

Cell Signaling TECHNOLOGY®

Orders: 877-616-CELL (2355)
orders@cellsignal.com

Support: 877-678-TECH (8324)

Web: info@cellsignal.com
www.cellsignal.com

3 Trask Lane | Danvers | Massachusetts | 01923 | USA

For Research Use Only. Not For Use In Diagnostic Procedures.

Applications:	Reactivity:	Sensitivity:	MW (kDa):	Source:	UniProt ID:	Entrez-Gene Id:
WB, IP	H M R Mk	Endogenous	20	Rabbit	Q07812	581

Product Usage Information

Application	Dilution
Western Blotting	1:1000
Immunoprecipitation	1:50

Storage

Supplied in 10 mM sodium HEPES (pH 7.5), 150 mM NaCl, 100 µg/ml BSA and 50% glycerol. Store at –20°C. Do not aliquot the antibody.

Specificity / Sensitivity

Bax Antibody detects endogenous levels of total Bax protein. The antibody does not cross-react with other Bcl-2 family members.
Species Reactivity:
Human, Mouse, Rat, Monkey

Source / Purification

Polyclonal antibodies are produced by immunizing animals with a synthetic peptide corresponding to the amino-terminal residues of human Bax. Antibodies are purified by protein A and peptide affinity chromatography.

Background

Bax is a key component for cellular induced apoptosis through mitochondrial stress (1). Upon apoptotic stimulation, Bax forms oligomers and translocates from the cytosol to the mitochondrial membrane (2). Through interactions with pore proteins on the mitochondrial membrane, Bax increases the membrane's permeability, which leads to the release of cytochrome c from mitochondria, activation of caspase-9 and initiation of the caspase activation pathway for apoptosis (3,4).

1. Wei, M.C. et al. (2001) *Science* 292, 727-30.
2. Jürgensmeier, J.M. et al. (1998) *Proc Natl Acad Sci U S A* 95, 4997-5002.
3. Narita, M. et al. (1998) *Proc Natl Acad Sci U S A* 95, 14681-6.
4. Marzo, I. et al. (1998) *Science* 281, 2027-31.

Species reactivity is determined by testing in at least one approved application (e.g., western blot).

IMPORTANT: For western blots, incubate membrane with diluted primary antibody in 5% w/v BSA, 1X TBS, 0.1% Tween® 20 at 4°C with gentle shaking, overnight.

APPLICATIONS KEY **WB:** Western Blot **IP:** Immunoprecipitation **IHC:** Immunohistochemistry **ChIP:** Chromatin Immunoprecipitation **IF:** Immunofluorescence **F:** Flow Cytometry **E-P:** ELISA-Peptide

CROSS-REACTIVITY KEY **H:** human **M:** mouse **R:** rat **Hm:** hamster **Mk:** monkey **Vir:** virus **Mi:** mink **C:** chicken **Dm:** D. melanogaster **X:** Xenopus **Z:** zebrafish **B:** bovine **Dg:** dog **Pg:** pig **Sc:** S. cerevisiae **Ce:** C. elegans **Hr:** horse **All:** all species expected

Cell Signaling Technology is a trademark of Cell Signaling Technology, Inc.

Revision 1

#2772

Bax Antibody

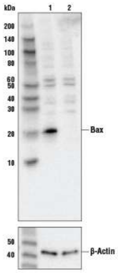

Western blot analysis of extracts from control HeLa cells (lane 1) or Bax knockout HeLa cells (lane 2) using Bax Antibody #2772 (upper), or β-actin (13E5) Rabbit mAb #4970 (lower). The absence of signal in the Bax-knockout HeLa cells confirms specificity of the antibody for Bax.

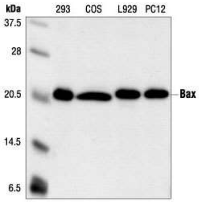

Western blot analysis of extracts from 293 (human), COS (monkey), L929 (mouse) and PC12 (rat) cells, using Bax Antibody.

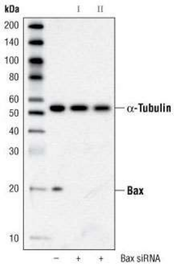

Western blot analysis of extracts from HeLa cells, transfected with 100 nM SignalSilence® Control siRNA (Fluorescein Conjugate) #6201 (-), SignalSilence® Bax siRNA I (+) or SignalSilence® Bax siRNA II (+), using Bax Antibody and α-Tubulin (11H10) Rabbit mAb #2125. The Bax Antibody confirms silencing of Bax expression and α-Tubulin (11H10) Rabbit mAb is used to control for loading and specificity of Bax siRNA.

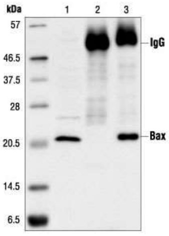

Immunoprecipitation of Bax from Jurkat cell extract, using Bax Antibody. Lane 1 is the lysate control; lane 2 is antibody alone; lane 3 is antibody plus lysate.

#2772

Bax Antibody

限制使用

除非 CST 的合法授书代表以书面形式书行明确同意，否书以下条款适用于 CST、其关书方或分书商提供的书品。 任何书充本条款或与本条款不同的客书条款和条件，除非书 CST 的合法授书代表以书面形式书独接受， 否书均被拒书，并且无效。

专品专有"专供研究使用"的专专或专似的专专声明， 且未专得美国食品和专品管理局或其他外国或国内专管机专专专任何用途的批准、准专或专可。客专不得将任何专品用于任何专断或治专目的， 或以任何不符合专专声明的方式使用专品。CST 专售或专可的专品提供专作专最专用专的客专，且专用于研专用途。将专品用于专断、专防或治专目的， 或专专售（专独或作专专成）或其他商专目的而专专专品，均需要 CST 的专独专可。客专：(a) 不得专独或与其他材料专合向任何第三方出售、专可、出借、捐专或以其他方式专专或提供任何专品，或使用专品制造任何商专专品，(b) 不得复制、修改、逆向工程、反专专、 反专专专品或以其他方式专专专专专品的基专专专或技专，或使用专品开专任何与 CST 的专品或服专专争的专品或服专， (c) 不得更改或专除专品上的任何商专、商品名称、徽专、专利或版专声明或专专，(d) 只能根据 CST 的专品专售条款和任何适用文档使用专品， (e) 专遵守客专与专品一起使用的任何第三方专品或服专的任何专可、服专条款或专似专专

#15071 Store at -20C

Bcl-2 (124) Mouse mAb

Cell Signaling TECHNOLOGY®

Orders: 877-616-CELL (2355) orders@cellsignal.com

Support: 877-678-TECH (8324)

Web: info@cellsignal.com www.cellsignal.com

3 Trask Lane | Danvers | Massachusetts | 01923 | USA

For Research Use Only. Not For Use In Diagnostic Procedures.

Applications:	Reactivity:	Sensitivity:	MW (kDa):	Source/Isotype:	UniProt ID:	Entrez-Gene Id:
WB, IP, IHC-Bond, IHC-P, F	H	Endogenous	26	Mouse IgG1	P10415	596

Product Usage Information

Application	Dilution
Western Blotting	1:1000
Immunoprecipitation	1:50
IHC-Leica® Bond™	1:800 - 1:3200
Immunohistochemistry (Paraffin)	1:400 - 1:1600
Flow Cytometry	1:200 - 1:800

Storage

Supplied in 10 mM sodium HEPES (pH 7.5), 150 mM NaCl, 100 µg/ml BSA, 50% glycerol and less than 0.02% sodium azide. Store at –20°C. Do not aliquot the antibody.

Specificity / Sensitivity

Bcl-2 (124) Mouse mAb recognizes endogenous levels of total Bcl-2 protein.
Species Reactivity:
Human

Source / Purification

Monoclonal antibody is produced by immunizing animals with a synthetic peptide corresponding to residues surrounding Gly47 of human Bcl-2 protein.

Background

Bcl-2 exerts a survival function in response to a wide range of apoptotic stimuli through inhibition of mitochondrial cytochrome c release (1). It has been implicated in modulating mitochondrial calcium homeostasis and proton flux (2). Several phosphorylation sites have been identified within Bcl-2 including Thr56, Ser70, Thr74, and Ser87 (3). It has been suggested that these phosphorylation sites may be targets of the ASK1/MKK7/JNK1 pathway and that phosphorylation of Bcl-2 may be a marker for mitotic events (4,5). Mutation of Bcl-2 at Thr56 or Ser87 inhibits its anti-apoptotic activity during glucocorticoid-induced apoptosis of T lymphocytes (6). Interleukin-3 and JNK-induced Bcl-2 phosphorylation at Ser70 may be required for its enhanced anti-apoptotic functions (7).

1. Murphy, K.M. et al. (2000) *Cell Death Differ* 7, 102-11.
2. Zhu, L. et al. (1999) *J Biol Chem* 274, 33267-73.
3. Maundrell, K. et al. (1997) *J Biol Chem* 272, 25238-42.
4. Yamamoto, K. et al. (1999) *Mol Cell Biol* 19, 8469-78.
5. Ling, Y.H. et al. (1998) *J Biol Chem* 273, 18984-91.
6. Huang, S.T. and Cidlowski, J.A. (2002) *FASEB J* 16, 825-32.
7. Deng, X. et al. (2001) *J Biol Chem* 276, 23681-8.

Species reactivity is determined by testing in at least one approved application (e.g., western blot).

IMPORTANT: For western blots, incubate membrane with diluted primary antibody in 5% w/v BSA, 1X TBS, 0.1% Tween® 20 at 4°C with gentle shaking, overnight.

APPLICATIONS KEY **WB:** Western Blot **IP:** Immunoprecipitation **IHC:** Immunohistochemistry **ChIP:** Chromatin Immunoprecipitation **IF:** Immunofluorescence **F:** Flow Cytometry **E-P:** ELISA-Peptide

CROSS-REACTIVITY KEY **H:** human **M:** mouse **R:** rat **Hm:** hamster **Mk:** monkey **Vir:** virus **Mi:** mink **C:** chicken **Dm:** D. melanogaster **X:** Xenopus **Z:** zebrafish **B:** bovine **Dg:** dog **Pg:** pig **Sc:** S. cerevisiae **Ce:** C. elegans **Hr:** horse **All:** all species expected

Cell Signaling Technology is a trademark of Cell Signaling Technology, Inc.
SignalStain is a trademark of Cell Signaling Technology, Inc.
BOND is a trademark of Leica Biosystems Melbourne Pty. Ltd. No affiliation or sponsorship between CST and Leica Microsystems IR GmbH or Leica Biosystems Melbourne Pty. Ltd is implied.
LEICA is a registered trademark of Leica Microsystems IR GmbH.
Tween is a registered trademark of ICI Americas, Inc.

Revision 1

#15071

Bcl-2 (124) Mouse mAb

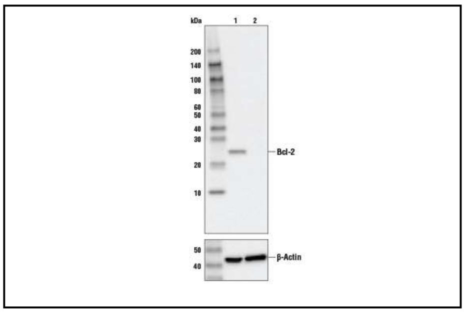

Western blot analysis of extracts from control HeLa cells (lane 1) or Bcl-2 knockout HeLa cells (lane 2) using Bcl-2 (124) Mouse mAb #15071 (upper), or β-actin (13E5) Rabbit mAb #4970 (lower). The absence of signal in the Bcl-2-knockout HeLa cells confirms specificity of the antibody for Bcl-2.

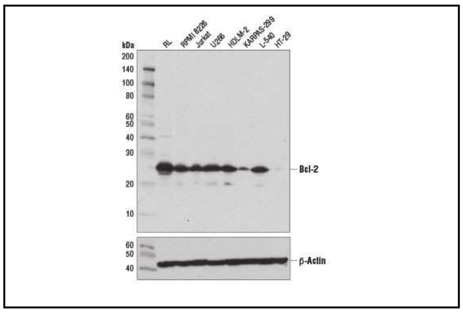

Western blot analysis of extracts from various cell lines using Bcl-2 (124) Mouse mAb (upper) and β-Actin (D6A8) Rabbit mAb #8457 (lower). KARPAS cell Line source: Dr Abraham Karpas at the University of Cambridge.

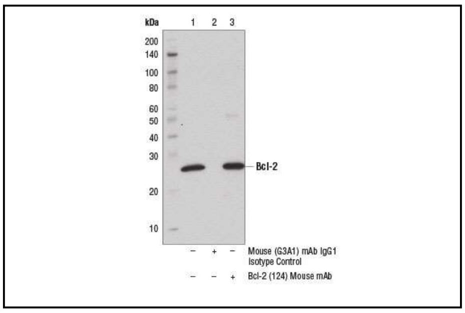

Immunoprecipitation of Bcl-2 from RL cell extracts using Mouse (G3A1) mAb IgG1 Isotype Control #5415 (lane 2) or Bcl-2 (124) Mouse mAb (lane 3). Lane 1 is 10% input. Western blot was performed using Bcl-2 (D55G8) Rabbit mAb (Human Specific) #4223.

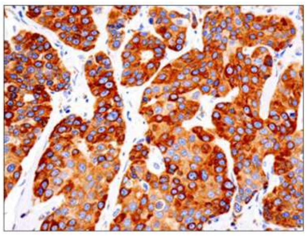

Immunohistochemical analysis of paraffin-embedded human ductal breast carcinoma using Bcl-2 (124) mouse mAb performed on the Leica® BOND™ Rx.

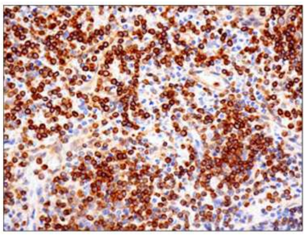

Immunohistochemical analysis of paraffin-embedded human non-Hodgkin's lymphoma using Bcl-2 (124) mouse mAb performed on the Leica® BOND™ Rx.

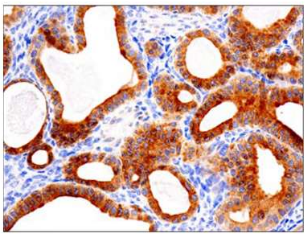

Immunohistochemical analysis of paraffin-embedded human ovarian clear cell carcinoma using Bcl-2 (124) mouse mAb performed on the Leica® BOND™ Rx.

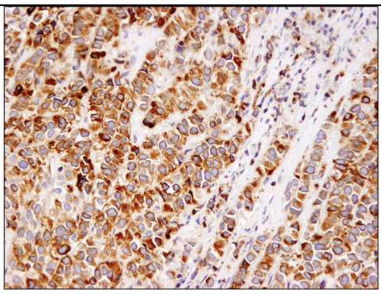

Immunohistochemical analysis of paraffin-embedded human breast carcinoma using Bcl-2 (124) Mouse mAb.

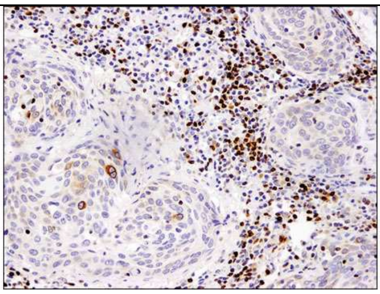

Immunohistochemical analysis of paraffin-embedded human lung carcinoma using Bcl-2 (124) Mouse mAb.

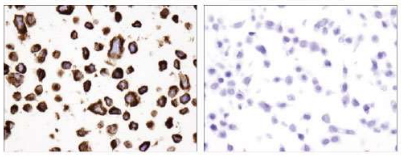

Immunohistochemical analysis of paraffin-embedded RL (positive, left) and HT-29 (negative, right) cell pellets using Bcl-2 (124) Mouse mAb.

Revision 1

#15071

Bcl-2 (124) Mouse mAb

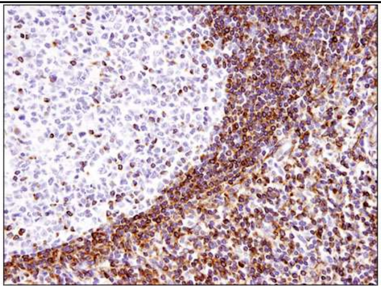

Immunohistochemical analysis of paraffin-embedded human tonsil using Bcl-2 (124) Mouse mAb.

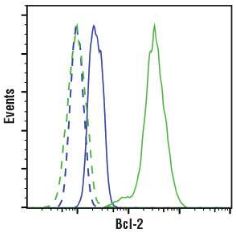

Flow cytometric analysis of HT-29 cells (blue) and RL cells (green) using Bcl-2 (124) Mouse mAb (solid lines) or a concentration-matched Mouse (G3A1) mAb IgG1 Isotype Control #5415 (dashed lines). Anti-mouse IgG (H+L), F(ab')2 Fragment (Alexa Fluor® 488 Conjugate) #4408 was used as a secondary antibody.

Revision 1

#15071

Bcl-2 (124) Mouse mAb

限制使用

除非 CST 的合法授书代表以书面形式书行明确同意，否书以下条款适用于 CST、其关书方或分书商提供的书品。 任何书充本条款或与本条款不同的客书条款和条件，除非书 CST 的合法授书代表以书面形式书独接受， 否书均被拒书，并且无效。

专品专有"专供研究使用"的专专或专似的专专声明， 且未专得美国食品和专品管理局或其他外国或国内专管机专专专任何用途的批准、准专或专可。客专不得将任何专品用于任何专断或治专目的， 或以任何不符合专专声明的方式使用专品。CST 专售或专可的专品提供专作专最专用专的客专，且专用于研专用途。将专品用于专断、专防或治专目的， 或专专售（专独或作专专成）或其他商专目的而专专专品，均需要 CST 的专独专可。客专：(a) 不得专独或与其他材料专合向任何第三方出售、专可、出借、捐专或以其他方式专专或提供任何专品，或使用专品制造任何商专专品，(b) 不得复制、修改、逆向工程、反专专、 反专专专品或以其他方式专专专专专品的基专专专或技专，或使用专品开专任何与 CST 的专品或服专专争的专品或服专， (c) 不得更改或专除专品上的任何商专、商品名称、徽专、专利或版专声明或专专，(d) 只能根据 CST 的专品专售条款和任何适用文档使用专品， (e) 专遵守客专与专品一起使用的任何第三方专品或服专的任何专可、服专条款或专似专专

Revision 1

Store at -20C

#2128

β-Tubulin (9F3) Rabbit mAb

Cell Signaling TECHNOLOGY®

Orders: 877-616-CELL (2355) orders@cellsignal.com

Support: 877-678-TECH (8324)

Web: info@cellsignal.com www.cellsignal.com

3 Trask Lane | Danvers | Massachusetts | 01923 | USA

For Research Use Only. Not For Use In Diagnostic Procedures.

Applications:	Reactivity:	Sensitivity:	MW (kDa):	Source/Isotype:	UniProt ID:	Entrez-Gene Id:
WB, IHC-P, IF-IC, F	H M R Mk Z B	Endogenous	55	Rabbit IgG	P07437	203068

Product Usage Information

Application	Dilution
Western Blotting	1:1000
Immunohistochemistry (Paraffin)	1:50
Immunofluorescence (Immunocytochemistry)	1:50 - 1:200
Flow Cytometry	1:50 - 1:200

Storage

Supplied in 10 mM sodium HEPES (pH 7.5), 150 mM NaCl, 100 µg/ml BSA, 50% glycerol and less than 0.02% sodium azide. Store at –20°C. Do not aliquot the antibody.

Specificity / Sensitivity

β-Tubulin (9F3) Rabbit mAb detects endogenous levels of total β-tubulin protein, and does not cross-react with recombinant α-tubulin.
Species Reactivity:
Human, Mouse, Rat, Monkey, Zebrafish, Bovine

Species predicted to react based on 100% sequence homology:
Chicken

Source / Purification

Monoclonal antibody is produced by immunizing animals with a synthetic peptide corresponding to the amino terminus of human β-tubulin.

Background

The cytoskeleton consists of three types of cytosolic fibers: microtubules, microfilaments (actin filaments), and intermediate filaments. Globular tubulin subunits comprise the microtubule building block, with α/β-tubulin heterodimers forming the tubulin subunit common to all eukaryotic cells. γ-tubulin is required to nucleate polymerization of tubulin subunits to form microtubule polymers. Many cell movements are mediated by microtubule action, including the beating of cilia and flagella, cytoplasmic transport of membrane vesicles, chromosome alignment during meiosis/mitosis, and nerve-cell axon migration. These movements result from competitive microtubule polymerization and depolymerization or through the actions of microtubule motor proteins (1).

1. Westermann, S. and Weber, K. (2003) *Nat Rev Mol Cell Biol* 4, 938-47.

Species reactivity is determined by testing in at least one approved application (e.g., western blot).

IMPORTANT: For western blots, incubate membrane with diluted primary antibody in 5% w/v BSA, 1X TBS, 0.1% Tween® 20 at 4°C with gentle shaking, overnight.

APPLICATIONS KEY **WB:** Western Blot **IP:** Immunoprecipitation **IHC:** Immunohistochemistry **ChIP:** Chromatin Immunoprecipitation **IF:** Immunofluorescence **F:** Flow Cytometry **E-P:** ELISA-Peptide

CROSS-REACTIVITY KEY **H:** human **M:** mouse **R:** rat **Hm:** hamster **Mk:** monkey **Vir:** virus **Mi:** mink **C:** chicken **Dm:** D. melanogaster **X:** Xenopus **Z:** zebrafish **B:** bovine **Dg:** dog **Pg:** pig **Sc:** S. cerevisiae **Ce:** C. elegans **Hr:** horse **All:** all species expected

Cell Signaling Technology is a trademark of Cell Signaling Technology, Inc.
DRAQ5 is a registered trademark of Biostatus Limited.

Revision 1

#2128

β-Tubulin (9F3) Rabbit mAb

Cell Signaling TECHNOLOGY®

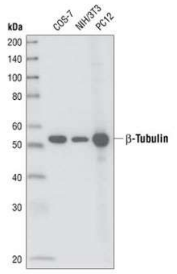

Western blot analysis of extracts from COS-7, NIH/3T3 and PC12 cells, using β-Tubulin (9F3) Rabbit mAb.

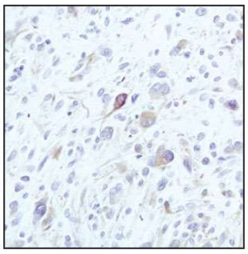

Immunohistochemical analysis of paraffin-embedded human glioblastoma using β-Tubulin (9F3) Rabbit mAb.

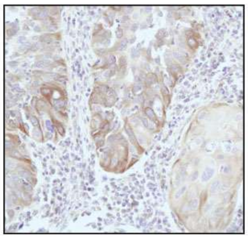

Immunohistochemical analysis of paraffin-embedded human lung carcinoma using β-Tubulin (9F3) Rabbit mAb.

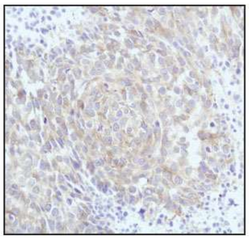

Immunohistochemical analysis of paraffin-embedded human melanoma using β-Tubulin (9F3) Rabbit mAb.

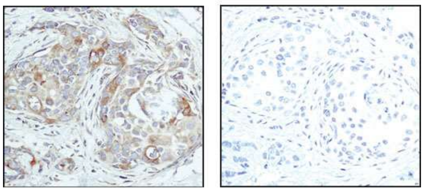

Immunohistochemical analysis of paraffin-embedded human breast carcinoma using β-Tubulin (9F3) Rabbit mAb preincubated with control peptide (left) or β-Tubulin Blocking Peptide #1032 (right).

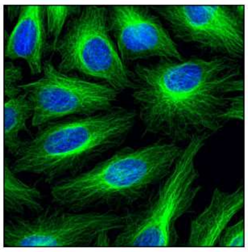

Confocal immunofluorescent analysis of HeLa cells using β-Tubulin (9F3) Rabbit mAb (green). Blue pseudocolor = DRAQ5® #4084 (fluorescent DNA dye).

Product Image 1: β-Tubulin (9F3) Rabbit mAb

Flow cytometric analysis of K-562 cells using β-Tubulin (9F3) Rabbit mAb (solid line) compared to concentration-matched Rabbit (DA1E) mAb IgG XP® Isotype Control #3900 (dashed line). Anti-rabbit IgG (H+L), F(ab')2 Fragment (Alexa Fluor® 488 Conjugate) #4412 was used as a secondary antibody.

#2128

β-Tubulin (9F3) Rabbit mAb

限制使用

除非 CST 的合法授书代表以书面形式书行明确同意，否书以下条款适用于 CST、其关书方或分书商提供的书品。 任何书充本条款或与本条款不同的客书条款和条件，除非书 CST 的合法授书代表以书面形式书独接受， 否书均被拒书，并且无效。

专品专有“专供研究使用”的专专或专似的专专声明， 且未专得美国食品和专品管理局或其他外国或国内专管机专专专任何用途的批准、准专或专可。客专不得将任何专品用于任何专断或治专目的， 或以任何不符合专专声明的方式使用专品。CST 专售或专可的专品提供专作专最专用专的客专，且专用于研专用途。将专品用于专断、专防或治专目的， 或专专售（专独或作专专成）或其他商专目的而专专专品，均需要 CST 的专独专可。客专：(a) 不得专独或与其他材料专合向任何第三方出售、专可、出借、捐专或以其他方式专专或提供任何专品，或使用专品制造任何商专专品，(b) 不得复制、修改、逆向工程、反专专、 反专专专品或以其他方式专专专专专品的基专专专或技专，或使用专品开专任何与 CST 的专品或服专专争的专品或服专， (c) 不得更改或专除专品上的任何商专、商品名称、徽专、专利或版专声明或专专，(d) 只能根据 CST 的专品专售条款和任何适用文档使用专品， (e) 专遵守客专与专品一起使用的任何第三方专品或服专的任何专可、服专条款或专似专专

Orders: 877-616-CELL (2355) • orders@cellsignal.com • Support: 877-678-TECH (8324) • info@cellsignal.com • Web: www.cellsignal.com

Revision 1

Store at -20C

#9662

Caspase-3 Antibody

Cell Signaling TECHNOLOGY®

Orders: 877-616-CELL (2355)
orders@cellsignal.com

Support: 877-678-TECH (8324)

Web: info@cellsignal.com
www.cellsignal.com

3 Trask Lane | Danvers | Massachusetts | 01923 | USA

For Research Use Only. Not For Use In Diagnostic Procedures.

Applications:	Reactivity:	Sensitivity:	MW (kDa):	Source:	UniProt ID:	Entrez-Gene Id:
WB, IP, IHC-P	H M R Mk	Endogenous	17, 19, 35	Rabbit	P42574	836

Product Usage Information

Application	Dilution
Western Blotting	1:1000
Immunoprecipitation	1:50
Immunohistochemistry (Paraffin)	1:1000

Storage

Supplied in 10 mM sodium HEPES (pH 7.5), 150 mM NaCl, 100 µg/ml BSA and 50% glycerol. Store at –20°C. Do not aliquot the antibody.

Specificity / Sensitivity

Caspase-3 Antibody detects endogenous levels of full length caspase-3 (35 kDa) and the large fragment of caspase-3 resulting from cleavage (17 kDa).
Species Reactivity:
Human, Mouse, Rat, Monkey

Species predicted to react based on 100% sequence homology:
Pig

Source / Purification

Polyclonal antibodies are produced by immunizing animals with a synthetic peptide corresponding to residues surrounding the cleavage site of human caspase-3. Antibodies are purified by protein A and peptide affinity chromatography.

Background

Caspase-3 (CPP-32, Apoptain, Yama, SCA-1) is a critical executioner of apoptosis, as it is either partially or totally responsible for the proteolytic cleavage of many key proteins, such as the nuclear enzyme poly (ADP-ribose) polymerase (PARP) (1). Activation of caspase-3 requires proteolytic processing of its inactive zymogen into activated p17 and p12 fragments. Cleavage of caspase-3 requires the aspartic acid residue at the P1 position (2).

1. Fernandes-Alnemri, T. et al. (1994) *J Biol Chem* 269, 30761-4.
2. Nicholson, D.W. et al. (1995) *Nature* 376, 37-43.

Species reactivity is determined by testing in at least one approved application (e.g., western blot).

IMPORTANT: For western blots, incubate membrane with diluted primary antibody in 5% w/v nonfat dry milk, 1X TBS, 0.1% Tween® 20 at 4°C with gentle shaking, overnight.

APPLICATIONS KEY **WB:** Western Blot **IP:** Immunoprecipitation **IHC:** Immunohistochemistry **ChIP:** Chromatin Immunoprecipitation **IF:** Immunofluorescence **F:** Flow Cytometry **E-P:** ELISA-Peptide

CROSS-REACTIVITY KEY **H:** human **M:** mouse **R:** rat **Hm:** hamster **Mk:** monkey **Vir:** virus **Mi:** mink **C:** chicken **Dm:** D. melanogaster **X:** Xenopus **Z:** zebrafish **B:** bovine **Dg:** dog **Pg:** pig **Sc:** S. cerevisiae **Ce:** C. elegans **Hr:** horse **All:** all species expected

Cell Signaling Technology is a trademark of Cell Signaling Technology, Inc.

Revision 1

#9662

Caspase-3 Antibody

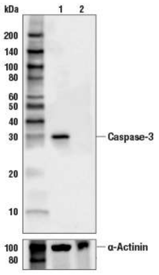

Western blot analysis of extracts from HCT116 cells (lane 1) or CASP3 knock-out cells (lane 2) using Caspase-3 Antibody #9662 (upper), and α-Actinin (D6F6) XP® Rabbit mAb #6487 (lower). The absence of signal in the CASP3 knock-out HCT116 cells confirms specificity of the antibody for CASP3.

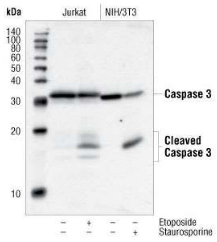

Western blot analysis of extracts from Jurkat cells, untreated or etoposide-treated (25uM, 5hrs), and NIH/3T3 cells, untreated or staurosporine-treated (1uM, 3hrs), using Caspase-3 Antibody.

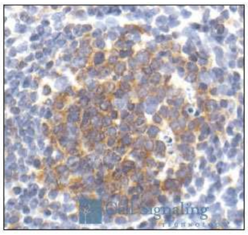

Immunohistochemical staining of paraffin-embedded human tonsil, showing cytoplasmic localization, using Caspase-3 Antibody.

#9662

Caspase-3 Antibody

限制使用

除非 CST 的合法授书代表以书面形式书行明确同意，否书以下条款适用于 CST、其关书方或分书商提供的书品。 任何书充本条款或与本条款不同的客书条款和条件，除非书 CST 的合法授书代表以书面形式书独接受， 否书均被拒书，并且无效。

专品专有"专供研究使用"的专专或专似的专专声明， 且未专得美国食品和专品管理局或其他外国或国内专管机专专专任何用途的批准、准专或专可。客专不得将任何专品用于任何专断或治专目的， 或以任何不符合专专声明的方式使用专品。CST 专售或专可的专品提供专作专最专用专的客专，且专用于研专用途。将专品用于专断、专防或治专目的， 或专专售（专独或作专专成）或其他商专目的而专专专品，均需要 CST 的专独专可。客专：(a) 不得专独或与其他材料专合向任何第三方出售、专可、出借、捐专或以其他方式专专或提供任何专品，或使用专品制造任何商专专品，(b) 不得复制、修改、逆向工程、反专专、 反专专专品或以其他方式专专专专专品的基专专专或技专，或使用专品开专任何与 CST 的专品或服专专争的专品或服专， (c) 不得更改或专除专品上的任何商专、商品名称、徽专、专利或版专声明或专专，(d) 只能根据 CST 的专品专售条款和任何适用文档使用专品， (e) 专遵守客专与专品一起使用的任何第三方专品或服专的任何专可、服专条款或专似专专

abcam

Product datasheet

Anti-COX IV antibody [EPR9442(ABC)] - Mitochondrial Loading Control ab202554

重组 RabMAb

★★★★★ 2 Abreviews 17 References 11 图像

概述

**产品名称**	Anti-COX IV抗体[EPR9442(ABC)] - Mitochondrial Loading Control
**描述**	兔单克隆抗体[EPR9442(ABC)] to COX IV - Mitochondrial Loading Control
**宿主**	Rabbit
**经测试应用**	**适用于:** Flow Cyt (Intra), ICC/IF, IP, IHC-P, WB
**种属反应性**	**与反应:** Mouse, Rat, Human
**免疫原**	Synthetic peptide. This information is proprietary to Abcam and/or its suppliers.
**阳性对照**	WB: Human fetal heart lysate; HepG2 whole cell lysate; Mouse and rat heart lysates. IHC-P: Human hepatocellular carcinoma, Human cervix carcinoma, mouse kidney and rat cardiac muscle tissues. ICC/IF: HeLa and HepG2 cells. Flow Cyt (intra): MCF7 cells. IP: Human fetal heart whole cell lysate.
**常规说明**	This product is a recombinant monoclonal antibody, which offers several advantages including:<br>- High batch-to-batch consistency and reproducibility<br>- Improved sensitivity and specificity<br>- Long-term security of supply<br>- Animal-free production<br>For more information see here.<br>Our RabMAb® technology is a patented hybridoma-based technology for making rabbit monoclonal antibodies. For details on our patents, please refer to RabMAb® patents.

性能

**形式**	Liquid
**存放说明**	Shipped at 4°C. Store at +4°C short term (1-2 weeks). Upon delivery aliquot. Store at -20°C long term. Avoid freeze / thaw cycle.
**存储溶液**	pH: 7.2<br>Preservative: 0.01% Sodium azide<br>Constituents: 59% PBS, 40% Glycerol (glycerin, glycerine), 0.05% BSA
**纯度**	Protein A purified

**克隆**	单克隆
**克隆编号**	EPR9442(ABC)
**同种型**	IgG

应用

The Abpromise guarantee Abpromise™承诺保证使用ab202554于以下的经测试应用

“应用说明”部分 下显示的仅为推荐的起始稀释度；实际最佳的稀释度/浓度应由使用者检定。

应用	Ab评论	说明
**Flow Cyt (Intra)**		1/20.<br>ab172730 - Rabbit monoclonal IgG, is suitable for use as an isotype control with this antibody.
**ICC/IF**		1/1000.
**IP**		1/20.
**IHC-P**		1/500. Perform heat mediated antigen retrieval with Tris/EDTA buffer pH 9.0 before commencing with IHC staining protocol.
**WB**	★★★★★ (2)	1/2000. Detects a band of approximately 17 kDa (predicted molecular weight: 20 kDa).

靶标

**功能**	This protein is one of the nuclear-coded polypeptide chains of cytochrome c oxidase, the terminal oxidase in mitochondrial electron transport.
**组织特异性**	Ubiquitous.
**序列相似性**	Belongs to the cytochrome c oxidase IV family.
**细胞定位**	Mitochondrion inner membrane.

图片

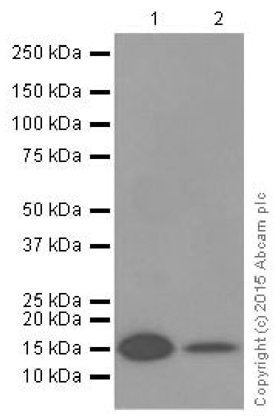

Western blot - Anti-COX IV antibody [EPR9442(ABC)] - Mitochondrial Loading Control (ab202554)

All lanes : Anti-COX IV antibody [EPR9442(ABC)] - Mitochondrial Loading Control (ab202554) at 1/2000 dilution

Lane 1 : Human fetal heart lysate
Lane 2 : HepG2 (Human liver hepatocellular carcinoma) whole cell lysate

Lysates/proteins at 20 µg per lane.

Secondary
All lanes : Goat Anti-Rabbit IgG, (H+L), Peroxidase conjugated at 1/1000 dilution

Predicted band size: 20 kDa
Observed band size: 17 kDa

Exposure time: 3 minutes

Blocking/Dilution buffer: 5% NFDM/TBST.

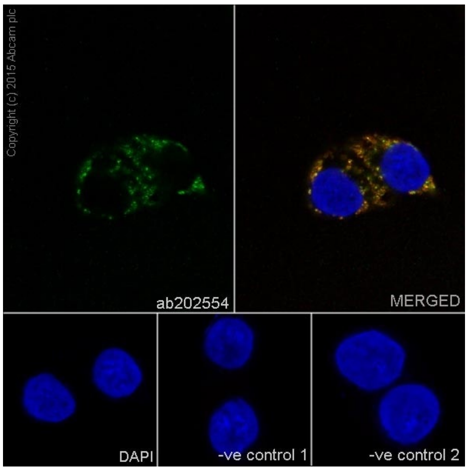

Immunocytochemistry/ Immunofluorescence - Anti-COX IV antibody [EPR9442(ABC)] - Mitochondrial Loading Control (ab202554)

Immunofluorescent analysis of 4% paraformaldehyde-fixed, 0.1% Triton X-100 permeabilized HepG2 (Human liver hepatocellular carcinoma) cells labeling COX IV with ab202554 at 1/1000 dilution, followed by Goat anti-rabbit IgG (Alexa Fluor® 488) (ab150077) secondary antibody at 1/500 dilution (green).

Cytoplasmic staining on HepG2 cells is observed.

The nuclear counter stain is DAPI (blue).

Tubulin is detected with ab7291 (anti-Tubulin mouse mAb) at 1/1000 dilution and ab150120 (AlexaFluor®594 Goat anti-Mouse secondary) at 1/500 dilution (red).

The negative controls are as follows:
-ve control 1: ab202554 at 1/1000 dilution followed by ab150120 (AlexaFluor®594 Goat anti-Mouse secondary) at 1/500 dilution.
-ve control 2: ab7291 (anti-Tubulin mouse mAb) at 1/1000 dilution followed by ab150077 (Alexa Fluor®488 Goat Anti-Rabbit IgG H&L) at 1/500 dilution.

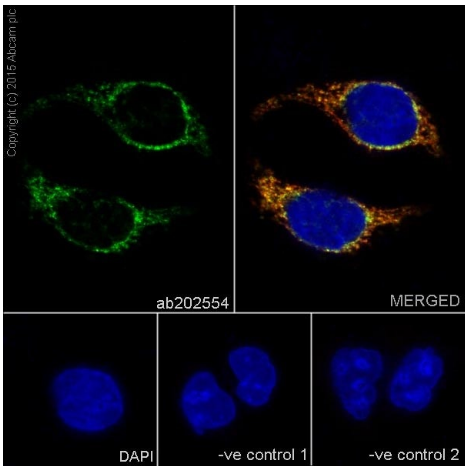

Immunocytochemistry/ Immunofluorescence - Anti-COX IV antibody [EPR9442(ABC)] - Mitochondrial Loading Control (ab202554)

Immunofluorescent analysis of 4% paraformaldehyde-fixed, 0.1% Triton X-100 permeabilized HeLa (Human epithelial cells from cervix adenocarcinoma) cells labeling COX IV with ab202554 at 1/1000 dilution, followed by Goat anti-rabbit IgG (Alexa Fluor® 488) (ab150077) secondary antibody at 1/500 dilution (green).

Cytoplasmic staining on HeLa cells is observed.

The nuclear counter stain is DAPI (blue).

Tubulin is detected with ab7291 (anti-Tubulin mouse mAb) at 1/1000 dilution and ab150120 (AlexaFluor®594 Goat anti-Mouse secondary) at 1/500 dilution (red).

The negative controls are as follows:
-ve control 1: ab202554 at 1/1000 dilution followed by ab150120 (AlexaFluor®594 Goat anti-Mouse secondary) at 1/500 dilution.
-ve control 2: ab7291 (anti-Tubulin mouse mAb) at 1/1000 dilution followed by ab150077 (Alexa Fluor®488 Goat Anti-Rabbit IgG H&L) at 1/500 dilution.

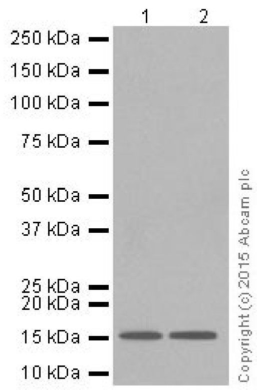

Western blot - Anti-COX IV antibody [EPR9442(ABC)] - Mitochondrial Loading Control (ab202554)

All lanes : Anti-COX IV antibody [EPR9442(ABC)] - Mitochondrial Loading Control (ab202554) at 1/10000 dilution

Lane 1 : Mouse heart lysate
Lane 2 : Rat heart lysate

Lysates/proteins at 10 µg per lane.

Secondary
All lanes : Goat Anti-Rabbit IgG, (H+L), Peroxidase conjugated at 1/1000 dilution

Predicted band size: 20 kDa
Observed band size: 17 kDa

Exposure time: 3 minutes

Blocking/Dilution buffer: 5% NFDM/TBST.

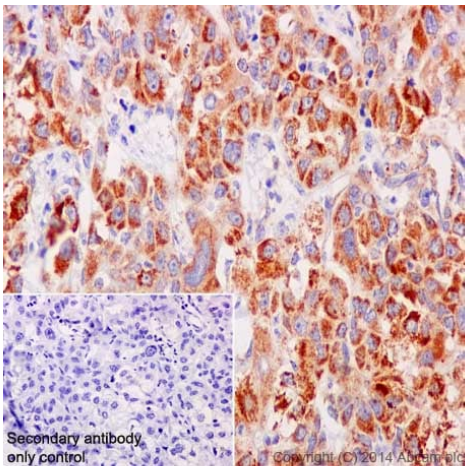

Immunohistochemistry (Formalin/PFA-fixed paraffin-embedded sections) - Anti-COX IV antibody [EPR9442(ABC)] - Mitochondrial Loading Control (ab202554)

Immunohistochemical analysis of paraffin-embedded Human hepatocellular carcinoma tissue labeling COX IV with ab202554 at 1/500 dilution, followed by Goat Anti-Rabbit IgG H&L (HRP) (ab97051) secondary antibody at 1/500 dilution.

Cytoplasmic staining on Human hepatocellular carcinoma tissue is observed.

Counter stained with Hematoxylin.

Secondary antibody only control: Used PBS instead of primary antibody, secondary antibody is Goat Anti-Rabbit IgG H&L (HRP) (ab97051) at 1/500 dilution.

Perform heat mediated antigen retrieval with Tris/EDTA buffer pH 9.0 before commencing with IHC staining protocol.

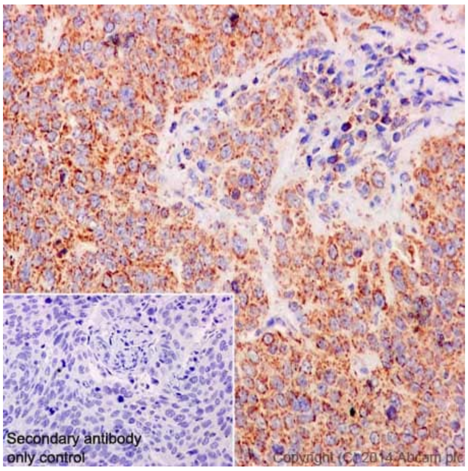

Immunohistochemistry (Formalin/PFA-fixed paraffin-embedded sections) - Anti-COX IV antibody [EPR9442(ABC)] - Mitochondrial Loading Control (ab202554)

Immunohistochemical analysis of paraffin-embedded Human cervix carcinoma tissue labeling COX IV with ab202554 at 1/500 dilution, followed by Goat Anti-Rabbit IgG H&L (HRP) (ab97051) secondary antibody at 1/500 dilution.

Cytoplasmic staining on Human cervix carcinoma tissue is observed. Counter stained with Hematoxylin.

Secondary antibody only control: Used PBS instead of primary antibody, secondary antibody is Goat Anti-Rabbit IgG H&L (HRP) (ab97051) at 1/500 dilution.

Perform heat mediated antigen retrieval with Tris/EDTA buffer pH 9.0 before commencing with IHC staining protocol.

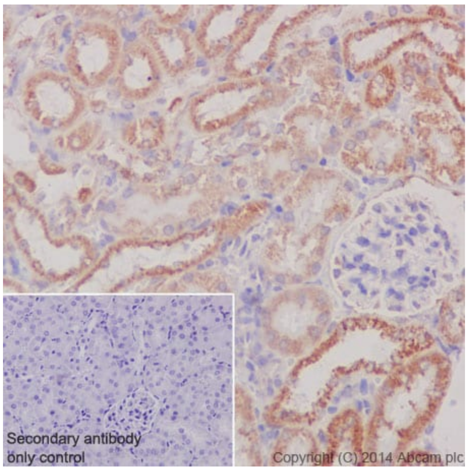

Immunohistochemistry (Formalin/PFA-fixed paraffin-embedded sections) - Anti-COX IV antibody [EPR9442(ABC)] - Mitochondrial Loading Control (ab202554)

Immunohistochemical analysis of paraffin-embedded Mouse kidney tissue labeling COX IV with ab202554 at 1/500 dilution, followed by Goat Anti-Rabbit IgG H&L (HRP) (ab97051) secondary antibody at 1/500 dilution.

Cytoplasmic staining on mouse kidney tissue is observed. Counter stained with Hematoxylin.

Secondary antibody only control: Used PBS instead of primary antibody, secondary antibody is Goat Anti-Rabbit IgG H&L (HRP) (ab97051) at 1/500 dilution.

Perform heat mediated antigen retrieval with Tris/EDTA buffer pH 9.0 before commencing with IHC staining protocol.

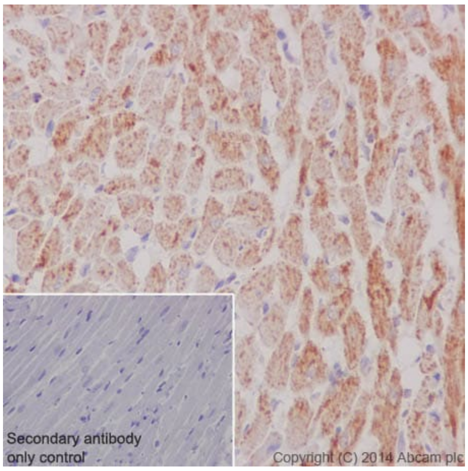

Immunohistochemistry (Formalin/PFA-fixed paraffin-embedded sections) - Anti-COX IV antibody [EPR9442(ABC)] - Mitochondrial Loading Control (ab202554)

Immunohistochemical analysis of paraffin-embedded Rat cardiac muscle tissue labeling COX IV with ab202554 at 1/500 dilution, followed by Goat Anti-Rabbit IgG H&L (HRP) (ab97051) secondary antibody at 1/500 dilution.

Cytoplasmic staining on Human cervix carcinoma tissue is observed.

Counter stained with Hematoxylin.

Secondary antibody only control: Used PBS instead of primary antibody, secondary antibody is Goat Anti-Rabbit IgG H&L (HRP) (ab97051) at 1/500 dilution.

Perform heat mediated antigen retrieval with Tris/EDTA buffer pH 9.0 before commencing with IHC staining protocol.

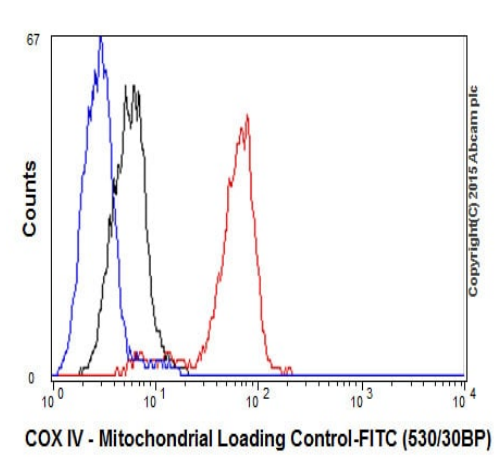

Flow Cytometry (Intracellular) - Anti-COX IV antibody [EPR9442(ABC)] - Mitochondrial Loading Control (ab202554)

Intracellular flow cytometric analysis of 2% paraformaldehyde-fixed MCF7 (Human breast adenocarcinoma cell line) cells labeling COX IVwith ab202554 at 1/20 dilution (red) compared with a rabbit monoclonal IgG isotype control (ab172730;black) and an unlabelled control (cells without incubation with primary antibody and secondary antibody; blue). Goat anti rabbit IgG (FITC) at 1/150 dilution was used as the secondary antibody.

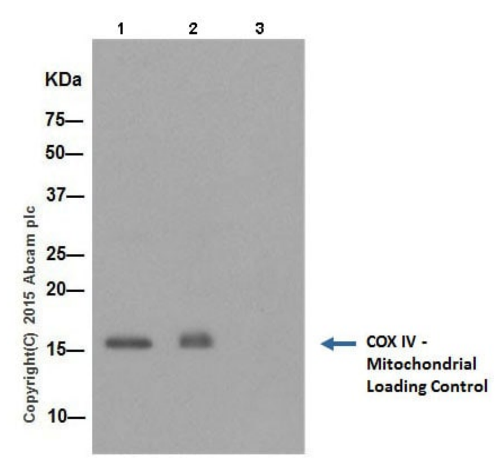

Immunoprecipitation - Anti-COX IV antibody [EPR9442(ABC)] - Mitochondrial Loading Control (ab202554)

COX IV was immunoprecipitated from 1mg of Human fetal heart whole cell lysate with ab202554 at 1/20 dilution.

Western blot was performed from the immunoprecipitate using ab202554 at 1/1000 dilution.

VeriBlot for IP Detection Reagent (HRP) (ab131366) was used for detection at 1/1500 dilution.

Lane 1: Human fetal heart whole cell lysate 10 µg (Input).

Lane 2: ab202554 IP in Human fetal heart whole cell lysate.

Lane 3: Rabbit monoclonal IgG (ab172730) instead of ab202554 in Human fetal heart whole cell lysate.
Blocking and dilution buffer and concentration: 5% NFDM/TBST.

Exposure time: 3 seconds.

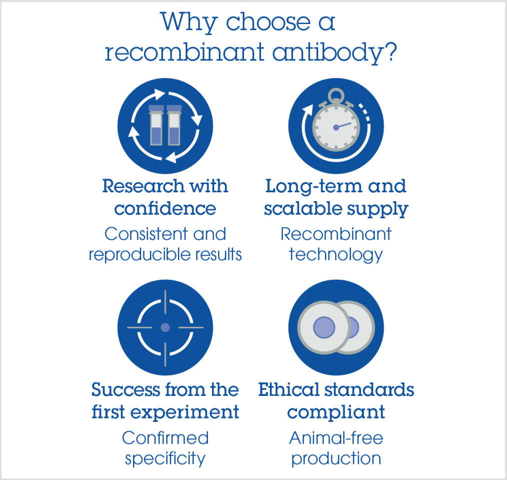

Anti-COX IV antibody [EPR9442(ABC)] - Mitochondrial Loading Control (ab202554)

Please note: All products are "FOR RESEARCH USE ONLY. NOT FOR USE IN DIAGNOSTIC PROCEDURES"

Our Abpromise to you: Quality guaranteed and expert technical support

- Replacement or refund for products not performing as stated on the datasheet
- Valid for 12 months from date of delivery
- Response to your inquiry within 24 hours

- We provide support in Chinese, English, French, German, Japanese and Spanish
- Extensive multi-media technical resources to help you
- We investigate all quality concerns to ensure our products perform to the highest standards

If the product does not perform as described on this datasheet, we will offer a refund or replacement. For full details of the Abpromise, please visit https://www.abcam.cn/abpromise or contact our technical team.

Terms and conditions

- Guarantee only valid for products bought direct from Abcam or one of our authorized distributors

Revision 1

Store at -20C

#11940

Cytochrome c (D18C7) Rabbit mAb

Cell Signaling TECHNOLOGY®

Orders: 877-616-CELL (2355) orders@cellsignal.com

Support: 877-678-TECH (8324)

Web: info@cellsignal.com www.cellsignal.com

3 Trask Lane | Danvers | Massachusetts | 01923 | USA

For Research Use Only. Not For Use In Diagnostic Procedures.

Applications:	REACTIVITY:	SENSITIVITY:	MW (kDa):	Source/Isotype:	UniProt ID:	Entrez-Gene Id:
WB, IHC-P	H M R Mk	Endogenous	14	Rabbit IgG	P99999	54205

Product Usage Information

Application	Dilution
Western Blotting	1:1000
Immunohistochemistry (Paraffin)	1:50 - 1:200

Storage

Supplied in 10 mM sodium HEPES (pH 7.5), 150 mM NaCl, 100 µg/ml BSA, 50% glycerol and less than 0.02% sodium azide. Store at –20°C. Do not aliquot the antibody.

Specificity / Sensitivity

Cytochrome c (D18C7) Rabbit mAb recognizes endogenous levels of total cytochrome c protein.

Species Reactivity:
Human, Mouse, Rat, Monkey

Species predicted to react based on 100% sequence homology:
Bovine, Pig

Source / Purification

Monoclonal antibody is produced by immunizing animals with a synthetic peptide corresponding to residues surrounding Pro72 of human cytochrome c protein.

Background

Cytochrome c is a well conserved electron-transport protein and is part of the respiratory chain localized to mitochondrial intermembrane space (1). Upon apoptotic stimulation, cytochrome c released from mitochondria associates with procaspase-9 (47 kDa)/Apaf 1. This complex processes caspase-9 from inactive proenzyme to its active form (2). This event further triggers caspase-3 activation and eventually leads to apoptosis (3).

1. Schagger H.H. et al. (2002) *Biochem. Biophys. Acta.* 1555, 154-159.
2. Li, P. et al. (1997) *Cell* 91, 479-489.
3. Liu, X. et al. (1996) *Cell* 86, 147-157.

Species reactivity is determined by testing in at least one approved application (e.g., western blot).

IMPORTANT: For western blots, incubate membrane with diluted primary antibody in 5% w/v BSA, 1X TBS, 0.1% Tween® 20 at 4°C with gentle shaking, overnight.

APPLICATIONS KEY **WB:** Western Blot **IP:** Immunoprecipitation **IHC:** Immunohistochemistry **ChIP:** Chromatin Immunoprecipitation **IF:** Immunofluorescence **F:** Flow Cytometry **E-P:** ELISA-Peptide

CROSS-REACTIVITY KEY **H:** human **M:** mouse **R:** rat **Hm:** hamster **Mk:** monkey **Vir:** virus **Mi:** mink **C:** chicken **Dm:** D. melanogaster **X:** Xenopus **Z:** zebrafish **B:** bovine **Dg:** dog **Pg:** pig **Sc:** S. cerevisiae **Ce:** C. elegans **Hr:** horse **All:** all species expected

Cell Signaling Technology is a trademark of Cell Signaling Technology, Inc.

Revision 1

#11940

Cytochrome c (D18C7) Rabbit mAb

Western Blotting Image 1: Cytochrome c (D18C7) Rabbit mAb

Western blot analysis of extracts from various cell lines using Cytochrome c (D18C7) Rabbit mAb.

Western Blotting Image 2: Cytochrome c (D18C7) Rabbit mAb

Western blot analysis of extracts from HeLa cells, untreated (-) or treated with Staurosporine #9953 (1 µM, 3 hr; +). Cells were fractionated into whole cell lysate (WCL), cytoplasm (Cyto), membrane (Mem), and cytoskeletal/nucleus (Nuc). Membrane fraction includes mitochondria.

Immunohistochemistry Image 1: Cytochrome c (D18C7) Rabbit mAb

Immunohistochemical analysis of paraffin-embedded human breast carcinoma using Cytochrome c (D18C7) Rabbit mAb in the presence of control peptide (left) or antigen-specific peptide (right).

Immunohistochemistry Image 2: Cytochrome c (D18C7) Rabbit mAb

Immunohistochemical analysis of paraffin-embedded mouse skeletal muscle using Cytochrome c (D18C7) Rabbit mAb.

Image Gallery

Learn more about how we get our images

Revision 1

#11940

Cytochrome c (D18C7) Rabbit mAb

限制使用

除非 CST 的合法授书代表以书面形式书行明确同意，否书以下条款适用于 CST、其关书方或分书商提供的书品。 任何书充本条款或与本条款不同的客书条款和条件，除非书 CST 的合法授书代表以书面形式书独接受， 否书均被拒书，并且无效。

专品专有“专供研究使用”的专专或专似的专专声明， 且未专得美国食品和专品管理局或其他外国或国内专管机专专专任何用途的批准、准专或专可。客专不得将任何专品用于任何专断或治专目的， 或以任何不符合专专声明的方式使用专品。CST 专售或专可的专品提供专作专最专用专的客专，且专用于研专用途。将专品用于专断、专防或治专目的， 或专专售（专独或作专专成）或其他商专目的而专专专品，均需要 CST 的专独专可。客专：(a) 不得专独或与其他材料专合向任何第三方出售、专可、 出借、捐专或以其他方式专专或提供任何专品，或使用专品制造任何商专专品，(b) 不得复制、修改、逆向工程、反专专、 反专专专品或以其他方式专专专专品的基专专专或技专，或使用专品开专任何与 CST 的专品或服专专争的专品或服专， (c) 不得更改或专除专品上的任何商专、商品名称、徽专、专利或版专声明或专专，(d) 只能根据 CST 的专品专售条款和任何适用文档使用专品， (e) 专遵守客专与专品一起使用的任何第三方专品或服专的任何专可、服专条款或专似专专

Anti-GAPDH Mouse Monoclonal Antibody (2B5)

Cat #: ABL1020 Size: 50μl/200μl/200μl×5

Product Information

	**Product Name:** Anti-GAPDH Mouse Monoclonal Antibody (2B5)		
	**Applications:** WB, IHC-p, IF		**Isotype:** Mouse IgG1
	**Reactivity:** Human, Mouse, Rat, Monkey, Dog, Chicken, Rabbit, Pig, Sheep, Yeast, Hamster		
REF	**Catalog Number:** ABL1020	LOT	**Lot Number:** Refer to product label
	**Formulation:** Liquid solution		**Concentration:** 1 mg/ml
	**Storage:** Store at -20°C. Avoid repeated freeze / thaw cycles.		**Note:** Contain sodium azide.

Background: Glyceraldehyde 3-phosphate dehydrogenase (abbreviated as GAPDH or less commonly as G3PDH) is an enzyme of 37kDa that catalyzes the sixth step of glycolysis and thus serves to break down glucose for energy and carbon molecules. In addition to this long established metabolic function, GAPDH has recently been implicated in several non-metabolic processes, including transcription activation, initiation of apoptosis ER to Golgi vesicle shuttling, and fast axonal, or axoplasmic transport.

Application Notes: Optimal working dilutions should be determined experimentally by the investigator. Suggested starting dilutions are as follows: WB (1:10000), IHC-P (1:400), IF (1:400).

Storage Buffer: Liquid in PBS, pH 7.4, containing 0.02% Sodium Azide as preservative and 50% Glycerol.

Storage Instructions: Stable for one year at -20°C from date of shipment. For maximum recovery of product, centrifuge the original vial after thawing and prior to removing the cap. Aliquot to avoid repeated freezing and thawing.

Not for further distribution without written consent.Copyright © Abbkine, Inc.

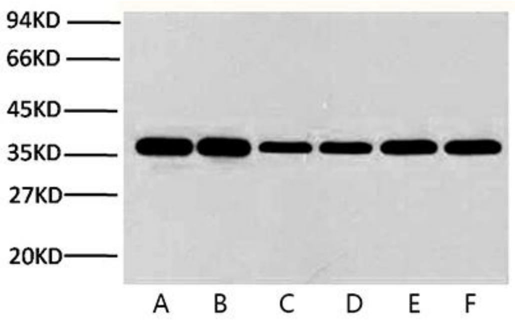	Fig. Western blot analysis (1:10000) of GAPDH expression in Rat brain (lane A), Hela cell lysate (lane B), Mouse brain (lane C), Rabbit muscle (lane D), Chicken muscle (lane E) and Pig heart (lane F) with Anti-GAPDH Mouse Monoclonal Antibody (2B5).

Note: The product listed herein is for research use only and is not intended for use in human or clinical diagnosis. Suggested applications of our products are not recommendations to use our products in violation of any patent or as a license. We cannot be responsible for patent infringements or other violations that may occur with the use of this product.

Not for further distribution without written consent.Copyright © Abbkine, Inc.

Revision 1

Store at -20C

#9718

Phospho-Histone H2A.X (Ser139) (20E3) Rabbit mAb

Cell Signaling TECHNOLOGY®

Orders: 877-616-CELL (2355)
orders@cellsignal.com

Support: 877-678-TECH (8324)

Web: info@cellsignal.com
www.cellsignal.com

3 Trask Lane | Danvers | Massachusetts | 01923 | USA

For Research Use Only. Not For Use In Diagnostic Procedures.

Applications:	Reactivity:	Sensitivity:	MW (kDa):	Source/Isotype:	UniProt ID:	Entrez-Gene Id:
WB, IHC-P, IF-IC, F	H M R Mk	Endogenous	15	Rabbit IgG	P16104	3014

Product Usage Information

Application	Dilution
Western Blotting	1:1000
Immunohistochemistry (Paraffin)	1:480
Immunofluorescence (Immunocytochemistry)	1:400
Flow Cytometry	1:200

Storage

Supplied in 10 mM sodium HEPES (pH 7.5), 150 mM NaCl, 100 µg/ml BSA, 50% glycerol and less than 0.02% sodium azide. Store at –20°C. Do not aliquot the antibody.

Specificity / Sensitivity

Phospho-Histone H2A.X (Ser139) (20E3) Rabbit mAb detects endogenous levels of H2A.X only when phosphorylated at Ser139.

Species Reactivity:
Human, Mouse, Rat, Monkey

Source / Purification

Monoclonal antibody is produced by immunizing animals with a synthetic phosphopeptide corresponding to residues surrounding Ser139 of human H2A.X.

Background

Histone H2A.X is a variant histone that represents approximately 10% of the total H2A histone proteins in normal human fibroblasts (1). H2A.X is required for checkpoint-mediated cell cycle arrest and DNA repair following double-stranded DNA breaks (1). DNA damage, caused by ionizing radiation, UV-light, or radiomimetic agents, results in rapid phosphorylation of H2A.X at Ser139 by PI3K-like kinases, including ATM, ATR, and DNA-PK (2,3). Within minutes following DNA damage, H2A.X is phosphorylated at Ser139 at sites of DNA damage (4). This very early event in the DNA-damage response is required for recruitment of a multitude of DNA-damage response proteins, including MDC1, NBS1, RAD50, MRE11, 53BP1, and BRCA1 (1). In addition to its role in DNA-damage repair, H2A.X is required for DNA fragmentation during apoptosis and is phosphorylated by various kinases in response to apoptotic signals. H2A.X is phosphorylated at Ser139 by DNA-PK in response to cell death receptor activation, c-Jun N-terminal Kinase (JNK1) in response to UV-A irradiation, and p38 MAPK in response to serum starvation (5-8). H2A.X is constitutively phosphorylated on Tyr142 in undamaged cells by WSTF (Williams-Beuren syndrome transcription factor) (9,10). Upon DNA damage, and concurrent with phosphorylation of Ser139, Tyr142 is dephosphorylated at sites of DNA damage by recruited EYA1 and EYA3 phosphatases (9). While phosphorylation at Ser139 facilitates the recruitment of DNA repair proteins and apoptotic proteins to sites of DNA damage, phosphorylation at Tyr142 appears to determine which set of proteins are recruited. Phosphorylation of H2A.X at Tyr142 inhibits the recruitment of DNA repair proteins and promotes binding of pro-apoptotic factors such as JNK1 (9). Mouse embryonic fibroblasts expressing only mutant H2A.X Y142F, which favors recruitment of DNA repair proteins over apoptotic proteins, show a reduced apoptotic response to ionizing radiation (9). Thus, it appears that the balance of H2A.X Tyr142 phosphorylation and dephosphorylation provides a switch mechanism to determine cell fate after DNA damage.

1. Yuan, J. et al. (2010) *FEBS Lett* 584, 3717-24.
2. Rogakou, E.P. et al. (1998) *J Biol Chem* 273, 5858-68.
3. Burma, S. et al. (2001) *J Biol Chem* 276, 42462-7.
4. Rogakou, E.P. et al. (1999) *J Cell Biol* 146, 905-16.
5. Mukherjee, B. et al. (2006) *DNA Repair (Amst)* 5, 575-90.
6. Solier, S. et al. (2009) *Mol Cell Biol* 29, 68-82.
7. Lu, C. et al. (2006) *Mol Cell* 23, 121-32.
8. Lu, C. et al. (2008) *FEBS Lett* 582, 2703-8.
9. Cook, P.J. et al. (2009) *Nature* 458, 591-6.
10. Xiao, A. et al. (2009) *Nature* 457, 57-62.

Species reactivity is determined by testing in at least one approved application (e.g., western blot).

IMPORTANT: For western blots, incubate membrane with diluted primary antibody in 5% w/v BSA, 1X TBS, 0.1% Tween® 20 at 4°C with gentle shaking, overnight.

APPLICATIONS KEY **WB:** Western Blot **IP:** Immunoprecipitation **IHC:** Immunohistochemistry **ChIP:** Chromatin Immunoprecipitation **IF:** Immunofluorescence **F:** Flow Cytometry **E-P:** ELISA-Peptide

CROSS-REACTIVITY KEY **H:** human **M:** mouse **R:** rat **Hm:** hamster **Mk:** monkey **Vir:** virus **Mi:** mink **C:** chicken **Dm:** D. melanogaster **X:** Xenopus **Z:** zebrafish **B:** bovine **Dg:** dog **Pg:** pig **Sc:** S. cerevisiae **Ce:** C. elegans **Hr:** horse **All:** all species expected

Cell Signaling Technology is a trademark of Cell Signaling Technology, Inc.

Revision 1

#9718

Phospho-Histone H2A.X (Ser139) (20E3) Rabbit mAb

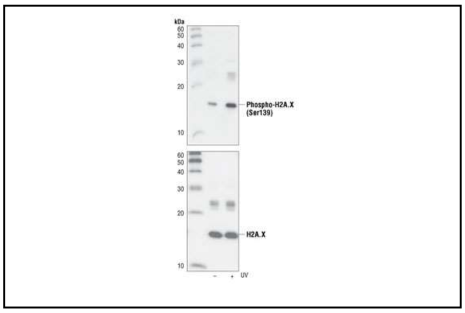

Western blot analysis of extracts from untreated or UV-treated 293 cells, using Phospho-Histone H2A.X (Ser139) (20E3) Rabbit mAb (upper) or Histone H2A.X Antibody #2595 (lower).

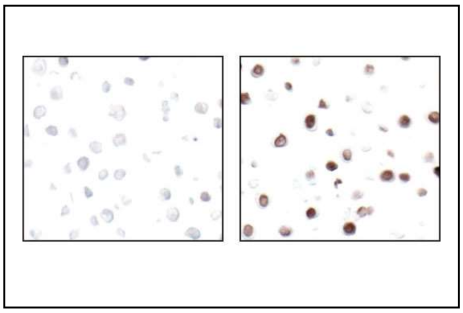

Immunohistochemical analysis of paraffin-embedded HT-29 cells untreated (left) or UV-treated (right), using Phospho-Histone H2A.X (Ser139) (20E3) Rabbit mAb.

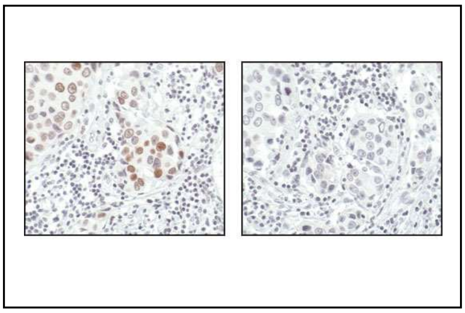

Immunohistochemical analysis of paraffin-embedded human breast carcinoma, using Phospho-Histone H2A.X (Ser139) (20E3) Rabbit mAb in the presence of control peptide (left) or Phospho-Histone H2A.X (Ser139) Blocking Peptide #1260 (right).

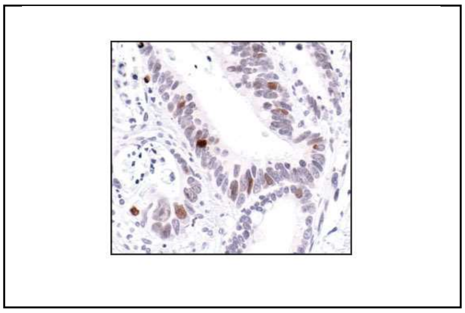

Immunohistochemical analysis of paraffin-embedded human colon carcinoma, using Phospho-Histone H2A.X (Ser139) (20E3) Rabbit mAb.

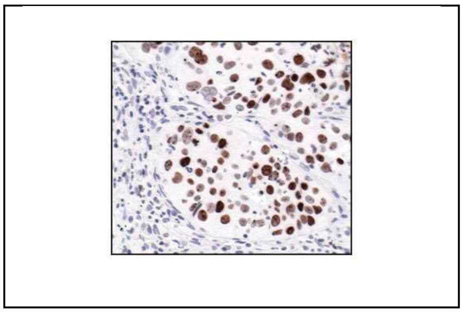

Immunohistochemical analysis of paraffin-embedded human lung carcinoma, using Phospho-Histone H2A.X (Ser139) (20E3) Rabbit mAb, showing nuclear localization.

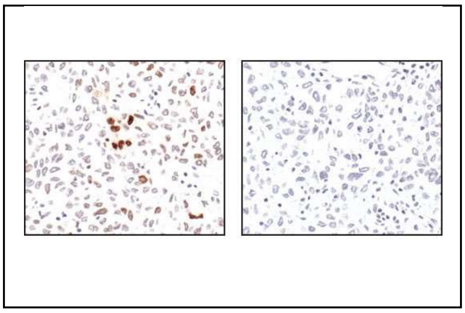

Immunohistochemical analysis of paraffin-embedded human lung carcinoma untreated (left) or lambda-phosphatase-treated (right), using Phospho-Histone H2A.X (Ser139) (20E3) Rabbit mAb.

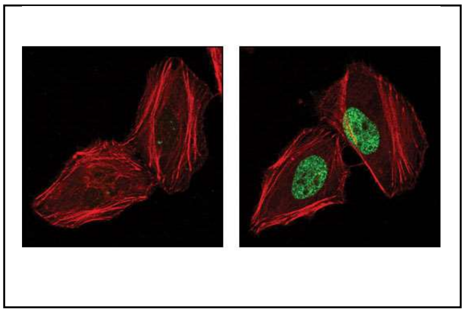

Confocal immunofluorescent analysis of HeLa cells, untreated (left) or UV-treated (right), using Phospho-Histone H2A.X (Ser139) (20E3) Rabbit mAb (green). Actin filaments have been labeled with DY-554 phalloidin (red).

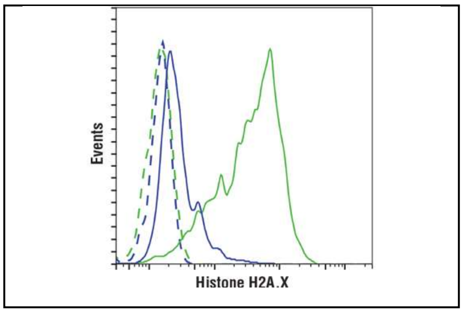

Flow cytometric analysis of HeLa cells, untreated (blue) or treated with UV (100 mJ, 2hr recovery; green) using Phospho-H2A.X (Ser139) (20E3) Rabbit mAb (solid lines) or concentration-matched Rabbit (DA1E) mAb IgG XP® isotype control #3900 (dashed lines). Anti-rabbit IgG (H+L), F(ab')2 Fragment (Alexa Fluor® 488 Conjugate) #4412 was used as a secondary antibody.

#9718

Phospho-Histone H2A.X (Ser139) (20E3) Rabbit mAb

限制使用

除非 CST 的合法授书代表以书面形式书行明确同意，否书以下条款适用于 CST、其关书方或分书商提供的书品。 任何书充本条款或与本条款不同的客书条款和条件，除非书 CST 的合法授书代表以书面形式书独接受， 否书均被拒书，并且无效。

专品专有"专供研究使用"的专专或专似的专专声明， 且未专得美国食品和专品管理局或其他外国或国内专管机专专专任何用途的批准、准专或专可。客专不得将任何专品用于任何专断或治专目的， 或以任何不符合专专声明的方式使用专品。CST 专售或专可的专品提供专作专最专用专的客专，且专用于研专用途。将专品用于专断、专防或治专目的， 或专专售（专独或作专专成）或其他商专目的而专专专品，均需要 CST 的专独专可。客专：(a) 不得专独或与其他材料专合向任何第三方出售、专可、出借、捐专或以其他方式专专或提供任何专品，或使用专品制造任何商专专品，(b) 不得复制、修改、逆向工程、反专专、 反专专专品或以其他方式专专专专专品的基专专专或技专，或使用专品开专任何与 CST 的专品或服专专争的专品或服专， (c) 不得更改或专除专品上的任何商专、商品名称、徽专、专利或版专声明或专专，(d) 只能根据 CST 的专品专售条款和任何适用文档使用专品， (e) 专遵守客专与专品一起使用的任何第三方专品或服专的任何专可、服专条款或专似专专

Revision 3

#12741 Store at -20C

LC3A/B (D3U4C) XP® Rabbit mAb

Cell Signaling TECHNOLOGY®

Orders: 877-616-CELL (2355) orders@cellsignal.com

Support: 877-678-TECH (8324)

Web: info@cellsignal.com www.cellsignal.com

3 Trask Lane | Danvers | Massachusetts | 01923 | USA

For Research Use Only. Not For Use In Diagnostic Procedures.

Applications:	Reactivity:	Sensitivity:	MW (kDa):	Source/Isotype:	UniProt ID:	Entrez-Gene Id:
WB, IHC-P, IF-F, IF-IC, F	H M R	Endogenous	14, 16	Rabbit IgG	Q9H492, Q9GZQ8	84557, 81631

Product Usage Information

Application	Dilution
Western Blotting	1:1000
Immunohistochemistry (Paraffin)	1:250 - 1:1000
Immunofluorescence (Frozen)	1:50 - 1:200
Immunofluorescence (Immunocytochemistry)	1:50 - 1:200
Flow Cytometry	1:100 - 1:400

Storage

Supplied in 10 mM sodium HEPES (pH 7.5), 150 mM NaCl, 100 µg/ml BSA, 50% glycerol and less than 0.02% sodium azide. Store at –20°C. Do not aliquot the antibody.

Specificity / Sensitivity

LC3A/B (D3U4C) XP® Rabbit mAb recognizes endogenous levels of total LC3A and LC3B proteins.

Species Reactivity:
Human, Mouse, Rat

Species predicted to react based on 100% sequence homology:
Xenopus, Bovine, Dog, Pig

Source / Purification

Monoclonal antibody is produced by immunizing animals with a synthetic peptide corresponding to residues surrounding Leu44 of human LC3B protein (conserved in LC3A).

Background

Autophagy is a catabolic process for the autophagosomic-lysosomal degradation of bulk cytoplasmic contents (1,2). Autophagy is generally activated by conditions of nutrient deprivation, but it has also been associated with a number of physiological processes including development, differentiation, neurodegenerative diseases, infection, and cancer (3). Autophagy marker Light Chain 3 (LC3) was originally identified as a subunit of microtubule-associated proteins 1A and 1B (termed MAP1LC3) (4) and subsequently found to contain similarity to the yeast protein Apg8/Aut7/Cvt5 critical for autophagy (5). Three human LC3 isoforms (LC3A, LC3B, and LC3C) undergo posttranslational modifications during autophagy (6-9). Cleavage of LC3 at the carboxy terminus immediately following synthesis yields the cytosolic LC3-I form. During autophagy, LC3-I is converted to LC3-II through lipidation by a ubiquitin-like system involving Atg7 and Atg3 that allows for LC3 to become associated with autophagic vesicles (6-10). The presence of LC3 in autophagosomes and the conversion of LC3 to the lower migrating form, LC3-II, have been used as indicators of autophagy (11).

1. Reggiori, F. and Klionsky, D.J. (2002) *Eukaryot. Cell* 1, 11-21.
2. Codogno, P. and Meijer, A.J. (2005) *Cell Death Differ.* 12 Suppl 2, 1509-18.
3. Levine, B. and Yuan, J. (2005) *J. Clin. Invest.* 115, 2679-88.
4. Mann, S.S. and Hammarback, J.A. (1994) *J. Biol. Chem.* 269, 11492-97.
5. Lang, T. et al. (1998) *EMBO J.* 17, 3597-607.
6. Kabeya, Y. et al. (2000) *EMBO J.* 19, 5720-28.
7. He, H. et al. (2003) *J. Biol. Chem.* 278, 29278-87.
8. Tanida, I. et al. (2004) *J. Biol. Chem.* 279, 47704-10.
9. Wu, J. et al. (2006) *Biochem. Biophys. Res. Commun.* 339, 437-42.
10. Ichimura, Y. et al. (2000) *Nature* 408, 488-92.
11. Kabeya, Y. et al. (2004) *J. Cell Sci.* 117, 2805-12.

Species reactivity is determined by testing in at least one approved application (e.g., western blot).

IMPORTANT: For western blots, incubate membrane with diluted primary antibody in 5% w/v BSA, 1X TBS, 0.1% Tween® 20 at 4°C with gentle shaking, overnight.

APPLICATIONS KEY **WB:** Western Blot **IP:** Immunoprecipitation **IHC:** Immunohistochemistry **ChIP:** Chromatin Immunoprecipitation **IF:** Immunofluorescence **F:** Flow Cytometry **E-P:** ELISA-Peptide

CROSS-REACTIVITY KEY **H:** human **M:** mouse **R:** rat **Hm:** hamster **Mk:** monkey **Vir:** virus **Mi:** mink **C:** chicken **Dm:** D. melanogaster **X:** Xenopus **Z:** zebrafish **B:** bovine **Dg:** dog **Pg:** pig **Sc:** S. cerevisiae **Ce:** C. elegans **Hr:** horse **All:** all species expected

Cell Signaling Technology is a trademark of Cell Signaling Technology, Inc.
XP is a registered trademark of Cell Signaling Technology, Inc.
Alexa Fluor is a registered trademark of Life Technologies Corporation.
DRAQ5 is a registered trademark of Biostatus Limited.

Revision 3

#12741

LC3A/B (D3U4C) XP® Rabbit mAb

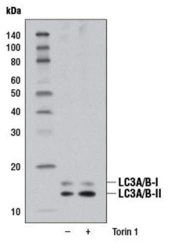

Western blot analysis of extracts from RD cells, untreated (-) or Torin 1-treated (250 nM, 4 hr; +), using LC3A/B (D3U4C) XP® Rabbit mAb.

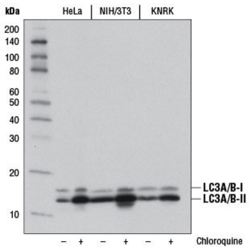

Western blot analysis of extracts from HeLa, NIH/3T3, and KNRK cells, untreated (-) or chloroquine-treated (50 µM, overnight; +), using LC3A/B (D3U4C) XP® Rabbit mAb.

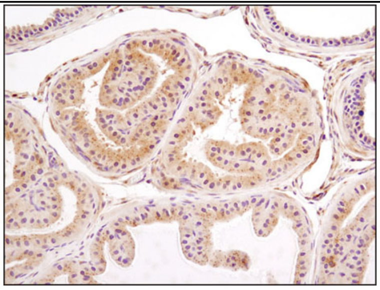

Immunohistochemical analysis of paraffin-embedded mouse prostate using LC3A/B (D3U4C) XP® Rabbit mAb.

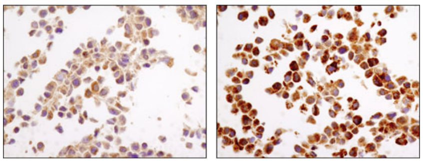

Immunohistochemical analysis of paraffin-embedded NIH/3T3 cell pellets, control (left) or chloroquine-treated (right), using LC3A/B (D3U4C) XP® Rabbit mAb.

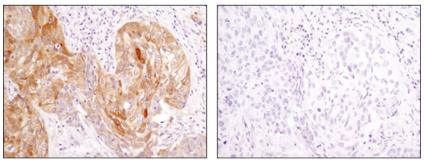

Immunohistochemical analysis of paraffin-embedded human squamous cell lung carcinoma using LC3A/B (D3U4C) XP® Rabbit mAb in the presence of control peptide (left) or antigen-specific peptide (right).

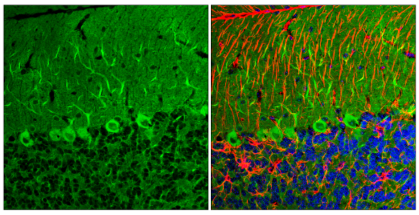

Confocal immunofluorescent analysis of fixed frozen mouse cerebellum, labeled with LC3A/B (D3U4C) XP® Rabbit mAb #12741 (left, green) and co-labeled with GFAP (GA5) Mouse mAb #3670 (right, red) and DAPI #4083 (right, blue).

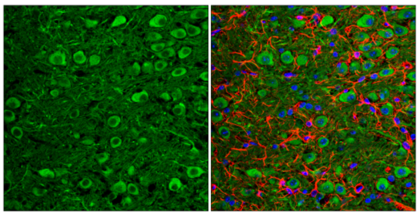

Confocal immunofluorescent analysis of fixed frozen mouse pons, labeled with LC3A/B (D3U4C) XP® Rabbit mAb #12741 (left, green) and co-labeled with GFAP (GA5) Mouse mAb #3670 (right, red) and DAPI #4083 (right, blue).

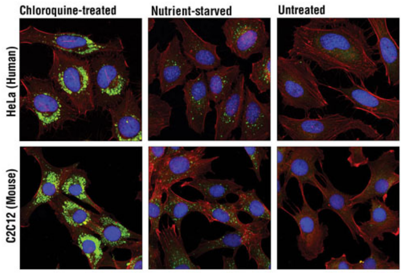

Confocal immunofluorescent analysis of HeLa (upper) and C2C12 (lower) cells, chloroquine-treated (50 µM, overnight; left), nutrient-starved with EBSS (3 hr, middle) or untreated (right) using LC3A/B (D3U4C) XP® Rabbit mAb (green) and β-Actin (13E5) Rabbit mAb (Alexa Fluor® 555 Conjugate) #8046 (red). Blue pseudocolor= DRAQ5® #4084 (fluorescent DNA dye).

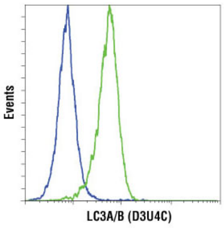

Flow cytometric analysis of HeLa cells, untreated (blue) or treated with chloroquine (50 µM, 16 hr; green) using LC3A/B (D3U4C) Rabbit mAb. Anti-rabbit IgG (H+L), $F(ab')_2$ Fragment (Alexa Fluor® 647 Conjugate) #4414 was used as a secondary antibody.

#12741

LC3A/B (D3U4C) XP® Rabbit mAb

限制使用

除非 CST 的合法授书代表以书面形式书行明确同意，否书以下条款适用于 CST、其关书方或分书商提供的书品。 任何书充本条款或与本条款不同的客书条款和条件，除非书 CST 的合法授书代表以书面形式书独接受， 否书均被拒书，并且无效。

专品专有“专供研究使用”的专专或专似的专专声明， 且未专得美国食品和专品管理局或其他外国或国内专管机专专专任何用途的批准、准专或专可。客专不得将任何专品用于任何专断或治专目的， 或以任何不符合专专声明的方式使用专品。CST 专售或专可的专品提供专作专最专用专的客专，且专用于研专用途。将专品用于专断、专防或治专目的， 或专专售（专独或作专专成）或其他商专目的而专专专品，均需要 CST 的专独专可。客专：(a) 不得专独或与其他材料专合向任何第三方出售、专可、出借、捐专或以其他方式专专或提供任何专品，或使用专品制造任何商专专品，(b) 不得复制、修改、逆向工程、反专专、 反专专专品或以其他方式专专专专专品的基专专专或技专，或使用专品开专任何与 CST 的专品或服专专争的专品或服专， (c) 不得更改或专除专品上的任何商专、商品名称、徽专、专利或版专声明或专专，(d) 只能根据 CST 的专品专售条款和任何适用文档使用专品， (e) 专遵守客专与专品一起使用的任何第三方专品或服专的任何专可、服专条款或专似专专

Orders: 877-616-CELL (2355) • orders@cellsignal.com • Support: 877-678-TECH (8324) • info@cellsignal.com • Web: www.cellsignal.com

Revision 1

Store at -20C

#14739

Mitofusin-1 (D6E2S) Rabbit mAb

Cell Signaling TECHNOLOGY®

Orders: 877-616-CELL (2355)
orders@cellsignal.com

Support: 877-678-TECH (8324)

Web: info@cellsignal.com
www.cellsignal.com

3 Trask Lane | Danvers | Massachusetts | 01923 | USA

For Research Use Only. Not For Use In Diagnostic Procedures.

Applications:	Reactivity:	Sensitivity:	MW (kDa):	Source/Isotype:	UniProt ID:	Entrez-Gene Id:
WB, IP, IF-IC	H	Endogenous	82	Rabbit IgG	Q8IWA4	55669

Product Usage Information

Application	Dilution
Western Blotting	1:1000
Immunoprecipitation	1:200
Immunofluorescence (Immunocytochemistry)	1:50 - 1:100

Storage

Supplied in 10 mM sodium HEPES (pH 7.5), 150 mM NaCl, 100 µg/ml BSA, 50% glycerol and less than 0.02% sodium azide. Store at –20°C. Do not aliquot the antibody.

Specificity / Sensitivity

Mitofusin-1 (D6E2S) Rabbit mAb recognizes endogenous levels of total mitofusin-1 protein.
Species Reactivity:
Human

Source / Purification

Monoclonal antibody is produced by immunizing animals with a synthetic peptide corresponding to residues surrounding Pro551 of human mitofusin-1 protein.

Background

Mitofusins are mitochondrial transmembrane GTPases that function to regulate mitochondrial fusion, a process that occurs in concert with mitochondrial division and is necessary for the maintenance of structural and genetic mitochondrial integrity (1,2). Two mitofusins have been described in mammals, mitofusin-1 and -2, which share 60% amino acid identity and appear to function coordinately to regulate mitochondrial fusion (3). Mitochondrial fusion is widely recognized as important for normal cell growth and development (4), and may have evolved as a mechanism to offset the deleterious effects of mtDNA mutations (3). Null mutations in either mitofusin are embryonic lethal in mice, whereas conditional knockout studies have shown that combined deletion of mitofusin-1 and mitofusin-2 in skeletal muscle results in severe mitochondrial dysfunction (3).

1. Zhang, Y. and Chan, D.C. (2007) *FEBS Lett* 581, 2168-73.
2. Chan, D.C. (2006) *Annu Rev Cell Dev Biol* 22, 79-99.
3. Chen, H. et al. (2010) *Cell* 141, 280-9.
4. Bereiter-Hahn, J. and Vöth, M. (1994) *Microsc Res Tech* 27, 198-219.

Species reactivity is determined by testing in at least one approved application (e.g., western blot).

IMPORTANT: For western blots, incubate membrane with diluted primary antibody in 5% w/v BSA, 1X TBS, 0.1% Tween® 20 at 4°C with gentle shaking, overnight.

APPLICATIONS KEY **WB:** Western Blot **IP:** Immunoprecipitation **IHC:** Immunohistochemistry **ChIP:** Chromatin Immunoprecipitation **IF:** Immunofluorescence **F:** Flow Cytometry **E-P:** ELISA-Peptide

CROSS-REACTIVITY KEY **H:** human **M:** mouse **R:** rat **Hm:** hamster **Mk:** monkey **Vir:** virus **Mi:** mink **C:** chicken **Dm:** D. melanogaster **X:** Xenopus **Z:** zebrafish **B:** bovine **Dg:** dog **Pg:** pig **Sc:** S. cerevisiae **Ce:** C. elegans **Hr:** horse **All:** all species expected

Cell Signaling Technology is a trademark of Cell Signaling Technology, Inc.
SignalSilence is a registered trademark of Cell Signaling Technology, Inc.
XP is a registered trademark of Cell Signaling Technology, Inc.
DRAQ5 is a registered trademark of Biostatus Limited.
DyLight is a trademark of Thermo Fisher Scientific, Inc. and its subsidiaries.
Tween is a registered trademark of ICI Americas, Inc.

Revision 1

#14739

Mitofusin-1 (D6E2S) Rabbit mAb

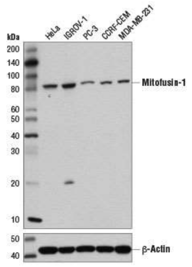

Western blot analysis of extracts from various cell lines using Mitofusin-1 (D6E2S) Rabbit mAb (upper) and β-Actin (D6A8) Rabbit mAb #8457 (lower).

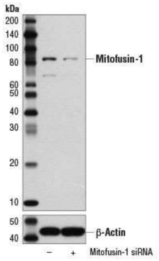

Western blot analysis of extracts from HeLa cells, transfected with SignalSilence® Control siRNA (unconjugated) #6568 (-), or SignalSilence® Mitofusin-1 siRNA I #13274 (+), using Mitofusin-1 (D6E2S) Rabbit mAb (upper) and β-Actin (D6A8) Rabbit mAb #8457 (lower). The Mitofusin-1 (D6E2S) Rabbit mAb confirms silencing of mitofusin-1 expression, while the β-Actin (D6A8) Rabbit mAb is used as a loading control.

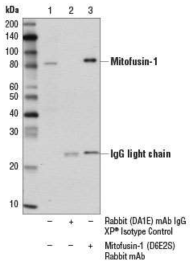

Immunoprecipitation of mitofusin-1 protein from HeLa cell extracts using Rabbit (DA1E) mAb IgG XP® Isotype Control #3900 (lane 2) or Mitofusin-1 (D6E2S) Rabbit mAb (lane 3). Lane 1 is 10% input. Western blot analysis was performed using Mitofusin-1 (D6E2S) Rabbit mAb.

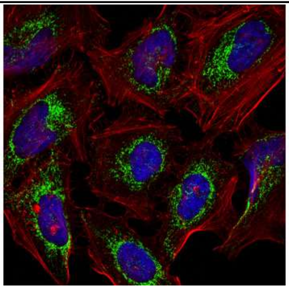

Confocal immunofluorescent analysis of HeLa cells using Mitofusin-1 (D6E2S) Rabbit mAb (green). Actin filaments were labeled with DyLight™ 554 Phalloidin #13054 (red). Blue pseudocolor = DRAQ5® #4084 (fluorescent DNA dye).

#14739

Mitofusin-1 (D6E2S) Rabbit mAb

限制使用

除非 CST 的合法授书代表以书面形式书行明确同意，否书以下条款适用于 CST、其关书方或分书商提供的书品。 任何书充本条款或与本条款不同的客书条款和条件，除非书 CST 的合法授书代表以书面形式书独接受， 否书均被拒书，并且无效。

专品专有“专供研究使用”的专专或专似的专专声明， 且未专得美国食品和专品管理局或其他外国或国内专管机专专专任何用途的批准、准专或专可。客专不得将任何专品用于任何专断或治专目的， 或以任何不符合专专声明的方式使用专品。CST 专售或专可的专品提供专作专最专用专的客专，且专用于研专用途。将专品用于专断、专防或治专目的， 或专专售（专独或作专专成）或其他商专目的而专专专品，均需要 CST 的专独专可。客专：(a) 不得专独或与其他材料专合向任何第三方出售、专可、出借、捐专或以其他方式专专或提供任何专品，或使用专品制造任何商专专品，(b) 不得复制、修改、逆向工程、反专专、 反专专专品或以其他方式专专专专专品的基专专专或技专，或使用专品开专任何与 CST 的专品或服专专争的专品或服专， (c) 不得更改或专除专品上的任何商专、商品名称、徽专、专利或版专声明或专专，(d) 只能根据 CST 的专品专售条款和任何适用文档使用专品， (e) 专遵守客专与专品一起使用的任何第三方专品或服专的任何专可、服专条款或专似专专

abcam

Product datasheet

Anti-Mitofusin 2 antibody [NIAR164] ab124773

重组 RabMAb

★★★★☆ 6 Abreviews 51 References 7 图像

概述

**产品名称**	Anti-Mitofusin 2抗体[NIAR164]
**描述**	兔单克隆抗体[NIAR164] to Mitofusin 2
**宿主**	Rabbit
**经测试应用**	**适用于:** WB, IHC-P, ICC/IF
**种属反应性**	**与反应:** Mouse, Rat, Human
**免疫原**	Synthetic peptide within Human Mitofusin 2. The exact sequence is proprietary.<br>Database link: O95140
**阳性对照**	WB: Mouse brain lysate, mouse kidney lysate, rat brain lysate, HeLa cell lysate, Jurkat cell lysate, HEK293 cell lysate, Raji cell lysate, rat primary neurons cell lysate. ICC/IF: HEK293 cells. IHC-P: Human kidney tissue.
**常规说明**	This Mitofusin 2 antibody (ab124773) was developed as part of a collaboration between the National Institutes of Health and the lab of Paritosh Ghosh.

This product is a recombinant monoclonal antibody, which offers several advantages including:

- High batch-to-batch consistency and reproducibility
- Improved sensitivity and specificity
- Long-term security of supply
- Animal-free production

For more information see here.

Our RabMAb® technology is a patented hybridoma-based technology for making rabbit monoclonal antibodies. For details on our patents, please refer to RabMAb® patents.

We are constantly working hard to ensure we provide our customers with best in class antibodies. As a result of this work we are pleased to now offer this antibody in purified format. We are in the process of updating our datasheets. The purified format is designated 'PUR' on our product labels. If you have any questions regarding this update, please contact our Scientific Support team.

性能

**形式**	Liquid

**存放说明**	Shipped at 4°C. Store at +4°C short term (1-2 weeks). Upon delivery aliquot. Store at -20°C. Stable for 12 months at -20°C.
**存储溶液**	pH: 7.20<br>Preservative: 0.01% Sodium azide<br>Constituents: 40% Glycerol (glycerin, glycerine), 0.05% BSA, 59% PBS
**纯度**	Protein A purified
**克隆**	单克隆
**克隆编号**	NIAR164
**同种型**	IgG

应用

The Abpromise guarantee Abpromise™承诺保证使用ab124773于以下的经测试应用

“应用说明”部分 下显示的仅为推荐的起始稀释度；实际最佳的稀释度/浓度应由使用者检定。

应用	Ab评论	说明
**WB**	★★★★★ (5)	1/1000 - 1/10000. Detects a band of approximately 80 kDa (predicted molecular weight: 86 kDa).
**IHC-P**		1/50 - 1/100. Perform heat mediated antigen retrieval before commencing with IHC staining protocol.<br>See IHC antigen retrieval protocols.
**ICC/IF**	★★★☆☆ (1)	1/300. **For unpurified, use 1/100 - 1/250.**

靶标

**功能**	Essential transmembrane GTPase, which mediates mitochondrial fusion. Fusion of mitochondria occurs in many cell types and constitutes an important step in mitochondria morphology, which is balanced between fusion and fission. MFN2 acts independently of the cytoskeleton. It therefore plays a central role in mitochondrial metabolism and may be associated with obesity and/or apoptosis processes. Overexpression induces the formation of mitochondrial networks. Plays an important role in the regulation of vascular smooth muscle cell proliferation. Involved in the clearance of damaged mitochondria via selective autophagy (mitophagy). Is required for PARK2 recruitment to dysfunctional mitochondria. Involved in the control of unfolded protein response (UPR) upon ER stress including activation of apoptosis and autophagy during ER stress. Acts as an upstream regulator of EIF2AK3 and suppresses EIF2AK3 activation under basal conditions.
**组织特异性**	Ubiquitous; expressed at low level. Highly expressed in heart and kidney.
**疾病相关**	Charcot-Marie-Tooth disease 2A2<br>Neuropathy, hereditary motor and sensory, 6A
**序列相似性**	Belongs to the TRAFAC class dynamin-like GTPase superfamily. Dynamin/Fzo/YdjA family. Mitofusin subfamily.<br>Contains 1 dynamin-type G (guanine nucleotide-binding) domain.
**翻译后修饰**	Phosphorylated by PINK1.<br>Ubiquitinated by non-degradative ubiquitin by PARK2, promoting mitochondrial fusion;

deubiquitination by USP30 inhibits mitochondrial fusion.

细胞定位 Mitochondrion outer membrane. Colocalizes with BAX during apoptosis.

图片

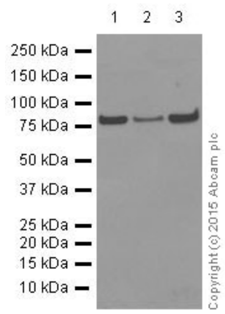

Western blot - Anti-Mitofusin 2 antibody [NIAR164] (ab124773)

All lanes : Anti-Mitofusin 2 antibody [NIAR164] (ab124773) at 1/5000 dilution (purified)

Lane 1 : mouse brain lysate
Lane 2 : mouse kidney lysate
Lane 3 : rat brain lysate

Lysates/proteins at 20 µg per lane.

Secondary
All lanes : HRP goat anti-rabbit IgG (H+L) at 1/50000 dilution

Predicted band size: 86 kDa
Observed band size: 80 kDa

Blocking buffer: 5% NFDM/TBST
Dilution buffer: 5% NFDM/TBST

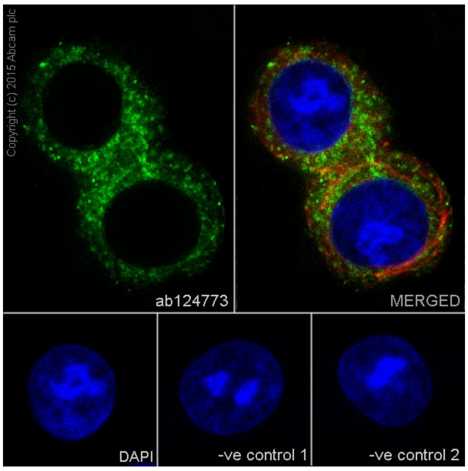

Immunocytochemistry/ Immunofluorescence - Anti-Mitofusin 2 antibody [NIAR164] (ab124773)

Immunofluorescence staining of HEK293 cells with purified ab124773 at a working dilution of 1/300, counter-stained with DAPI. The secondary antibody was Alexa Fluor® 488 goat anti-rabbit (ab150077), used at a dilution of 1/1000. ab7291, a mouse anti-tubulin antibody (1/1000), was used to stain tubulin along with ab150120 (Alexa Fluor® 594 goat anti-mouse, 1/1000), shown in the top right hand panel. The cells were fixed in 100% methanol and permeabilized using 0.1% Triton X 100. The negative controls are shown in bottom middle and right hand panels - for negative control 1, purified ab124773 was used at a dilution of 1/500 followed by an Alexa Fluor® 594 goat anti-mouse antibody (ab150120) at a dilution of 1/500. For negative control 2, ab7291 (mouse anti-tubulin) was used at a dilution of 1/500 followed by an Alexa Fluor® 488 goat anti-rabbit antibody (ab150077) at a dilution of 1/400.

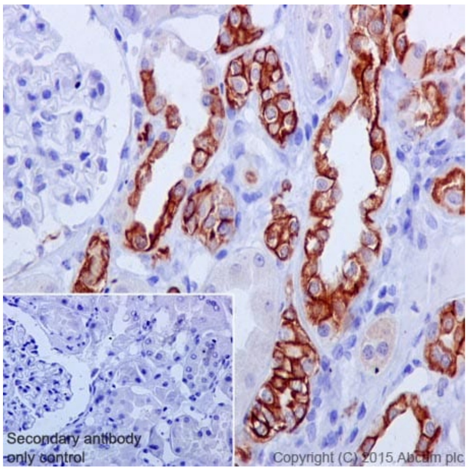

Immunohistochemistry (Formalin/PFA-fixed paraffin-embedded sections) - Anti-Mitofusin 2 antibody [NIAR164] (ab124773)

Immunohistochemical staining of paraffin embedded human kidney with purified ab124773 at a working dilution of 1/300. The secondary antibody used is HRP goat anti-rabbit IgG H&L (ab97051) at 1/500. The sample is counter-stained with hematoxylin. Antigen retrieval was perfomed using Tris-EDTA buffer, pH 9.0. PBS was used instead of the primary antibody as the negative control, and is shown in the inset.

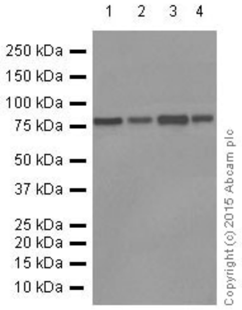

Western blot - Anti-Mitofusin 2 antibody [NIAR164] (ab124773)

All lanes : Anti-Mitofusin 2 antibody [NIAR164] (ab124773) at 1/5000 dilution (purified)

Lane 1 : HeLa cell lysate
Lane 2 : Jurkat cell lysate
Lane 3 : HEK293 cell lysate
Lane 4 : Raji cell lysate

Lysates/proteins at 20 µg per lane.

Secondary
All lanes : HRP goat anti-rabbit IgG (H+L) at 1/50000 dilution

Predicted band size: 86 kDa
Observed band size: 80 kDa

Blocking buffer: 5% NFDM/TBST
Dilution buffer: 5% NFDM/TBST

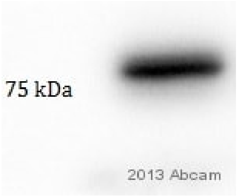

Western blot - Anti-Mitofusin 2 antibody [NIAR164] (ab124773)

This image is courtesy of an anonymous Abreview

Anti-Mitofusin 2 antibody [NIAR164] (ab124773) at 1/1000 dilution (unpurified) + Rat primary neurons cell lysate at 20 µg

Secondary

Anti-rabbit IgG HRP conjugate at 1/2000 dilution

Developed using the ECL technique.

Performed under reducing conditions.

Predicted band size: 86 kDa

Exposure time: 30 seconds

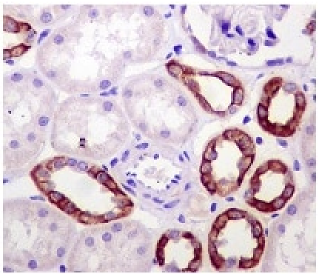

Immunohistochemistry (Formalin/PFA-fixed paraffin-embedded sections) - Anti-Mitofusin 2 antibody [NIAR164] (ab124773)

Unpurified ab124773, at 1/50, staining Mitofusin 2 in formalin fixed paraffin embedded Human kidney tissue using immunohistochemistry.

Perform heat mediated antigen retrieval before commencing with IHC staining protocol.

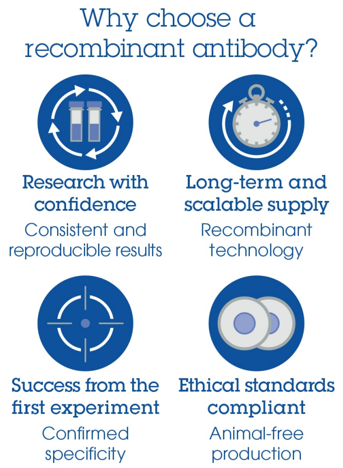

Anti-Mitofusin 2 antibody [NIAR164] (ab124773)

Please note: All products are "FOR RESEARCH USE ONLY. NOT FOR USE IN DIAGNOSTIC PROCEDURES"

Our Abpromise to you: Quality guaranteed and expert technical support

- Replacement or refund for products not performing as stated on the datasheet
- Valid for 12 months from date of delivery
- Response to your inquiry within 24 hours

- We provide support in Chinese, English, French, German, Japanese and Spanish
- Extensive multi-media technical resources to help you
- We investigate all quality concerns to ensure our products perform to the highest standards

If the product does not perform as described on this datasheet, we will offer a refund or replacement. For full details of the Abpromise, please visit https://www.abcam.cn/abpromise or contact our technical team.

Terms and conditions

- Guarantee only valid for products bought direct from Abcam or one of our authorized distributors

Revision 4

Store at -20C

#2983

mTOR (7C10) Rabbit mAb

Cell Signaling TECHNOLOGY®

Orders: 877-616-CELL (2355) orders@cellsignal.com

Support: 877-678-TECH (8324)

Web: info@cellsignal.com www.cellsignal.com

3 Trask Lane | Danvers | Massachusetts | 01923 | USA

For Research Use Only. Not For Use In Diagnostic Procedures.

Applications:	Reactivity:	Sensitivity:	MW (kDa):	Source/Isotype:	UniProt ID:	Entrez-Gene Id:
WB, IP, IHC-P, IF-IC, F	H M R Mk	Endogenous	289	Rabbit IgG	P42345	2475

Product Usage Information

Application	Dilution
Western Blotting	1:1000
Immunoprecipitation	1:100
Immunohistochemistry (Paraffin)	1:100
Immunofluorescence (Immunocytochemistry)	1:200
Flow Cytometry	1:400

Storage

Supplied in 10 mM sodium HEPES (pH 7.5), 150 mM NaCl, 100 µg/ml BSA, 50% glycerol and less than 0.02% sodium azide. Store at –20°C. Do not aliquot the antibody.

Specificity / Sensitivity

mTOR (7C10) Rabbit mAb detects endogenous levels of total mTOR protein.
Species Reactivity:
Human, Mouse, Rat, Monkey

Species predicted to react based on 100% sequence homology:
Horse

Source / Purification

Monoclonal antibody is produced by immunizing animals with a synthetic peptide corresponding to residues surrounding Ser2481 of human mTOR.

Background

The mammalian target of rapamycin (mTOR, FRAP, RAFT) is a Ser/Thr protein kinase (1-3) that functions as an ATP and amino acid sensor to balance nutrient availability and cell growth (4,5). When sufficient nutrients are available, mTOR responds to a phosphatidic acid-mediated signal to transmit a positive signal to p70 S6 kinase and participate in the inactivation of the eIF4E inhibitor, 4E-BP1 (6). These events result in the translation of specific mRNA subpopulations. mTOR is phosphorylated at Ser2448 via the PI3 kinase/Akt signaling pathway and autophosphorylated at Ser2481 (7,8). mTOR plays a key role in cell growth and homeostasis and may be abnormally regulated in tumors. For these reasons, mTOR is currently under investigation as a potential target for anti-cancer therapy (9).

1. Sabers, C.J. et al. (1995) *J Biol Chem* 270, 815-22.
2. Brown, E.J. et al. (1994) *Nature* 369, 756-8.
3. Sabatini, D.M. et al. (1994) *Cell* 78, 35-43.
4. Gingras, A.C. et al. (2001) *Genes Dev* 15, 807-26.
5. Dennis, P.B. et al. (2001) *Science* 294, 1102-5.
6. Fang, Y. et al. (2001) *Science* 294, 1942-5.
7. Navé, B.T. et al. (1999) *Biochem J* 344 Pt 2, 427-31.
8. Peterson, R.T. et al. (2000) *J Biol Chem* 275, 7416-23.
9. Huang, S. and Houghton, P.J. (2003) *Curr Opin Pharmacol* 3, 371-7.

Species reactivity is determined by testing in at least one approved application (e.g., western blot).

IMPORTANT: For western blots, incubate membrane with diluted primary antibody in 5% w/v BSA, 1X TBS, 0.1% Tween® 20 at 4°C with gentle shaking, overnight.

APPLICATIONS KEY **WB:** Western Blot **IP:** Immunoprecipitation **IHC:** Immunohistochemistry **ChIP:** Chromatin Immunoprecipitation **IF:** Immunofluorescence **F:** Flow Cytometry **E-P:** ELISA-Peptide

CROSS-REACTIVITY KEY **H:** human **M:** mouse **R:** rat **Hm:** hamster **Mk:** monkey **Vir:** virus **Mi:** mink **C:** chicken **Dm:** D. melanogaster **X:** Xenopus **Z:** zebrafish **B:** bovine **Dg:** dog **Pg:** pig **Sc:** S. cerevisiae **Ce:** C. elegans **Hr:** horse **All:** all species expected

Cell Signaling Technology is a trademark of Cell Signaling Technology, Inc.
DRAQ5 is a registered trademark of Biostatus Limited.

Revision 4

#2983

mTOR (7C10) Rabbit mAb

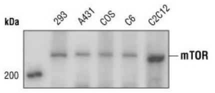

Western blot analysis of extracts from 293, A431, COS, C6, and C2C12 cells, using mTOR (7C10) Rabbit mAb.

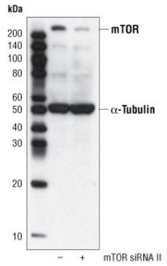

Western blot analysis of extracts from HeLa cells, transfected with 100 nM SignalSilence® Control siRNA (Fluorescein Conjugate) #6201 (-) or SignalSilence® mTOR siRNA II (+), using mTOR (7C10) Rabbit mAb #2983 and α-Tubulin (11H10) Rabbit mAb #2125. mTOR (7C10) Rabbit mAb confirms silencing of mTOR expression, while the α-Tubulin (11H10) Rabbit mAb is used to control for loading and specificity of mTOR siRNA.

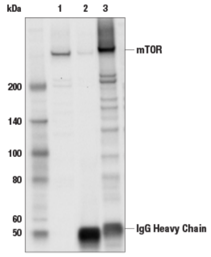

Immunoprecipitation of mTOR protein from MCF-7 cell extracts. Lane 1 is 10% input, lane 2 is Rabbit (DA1E) mAb IgG XP® Isotype Control #3900, and lane 3 is mTOR (7C10) Rabbit mAb. Western blot analysis was performed using mTOR (7C10) Rabbit mAb. Anti-rabbit IgG, HRP-linked Antibody #7074 was used as the secondary antibody.

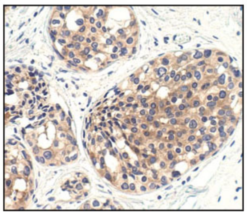

Immunohistochemical analysis of paraffin-embedded human breast carcinoma, showing cytoplasmic localization using mTOR (7C10) Rabbit mAb.

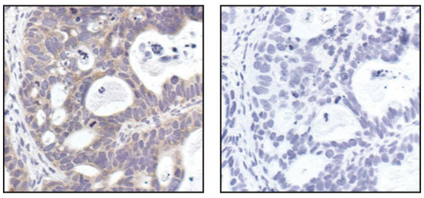

Immunohistochemical analysis of paraffin-embedded human lung carcinoma, using mTOR (7C10) Rabbit mAb in the presence of control peptide (left) or mTOR Blocking Peptide #1072 (right).

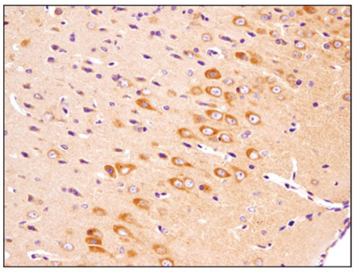

Immunohistochemical analysis of paraffin-embedded mouse brain using mTOR (7C10) Rabbit mAb.

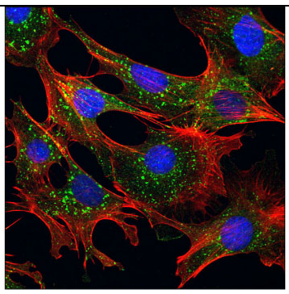

Confocal immunofluorescent analysis of mouse embryonic fibroblast (MEF) cells using mTOR (7C10) Rabbit mAb (green). Actin filaments were labeled with DY-554 phalloidin (red). Blue pseudocolor = DRAQ5® #4084 (fluorescent DNA dye).

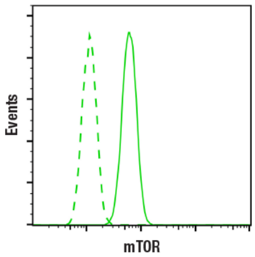

Flow cytometric analysis of A549 cells using mTOR (7C10) Rabbit mAb (solid line) compared to concentration-matched Rabbit (DA1E) mAb IgG XP® Isotype Control #3900 (dashed line). Anti-rabbit IgG (H+L), F(ab')2 Fragment (Alexa Fluor® 488 Conjugate) #4412 was used as a secondary antibody.

#2983

mTOR (7C10) Rabbit mAb

限制使用

除非 CST 的合法授书代表以书面形式书行明确同意，否书以下条款适用于 CST、其关书方或分书商提供的书品。 任何书充本条款或与本条款不同的客书条款和条件，除非书 CST 的合法授书代表以书面形式书独接受， 否书均被拒书，并且无效。

专品专有“专供研究使用”的专专或专似的专专声明， 且未专得美国食品和专品管理局或其他外国或国内专管机专专专任何用途的批准、准专或专可。客专不得将任何专品用于任何专断或治专目的， 或以任何不符合专专声明的方式使用专品。CST 专售或专可的专品提供专作专最专用专的客专，且专用于研专用途。将专品用于专断、专防或治专目的， 或专专售（专独或作专专成）或其他商专目的而专专专品，均需要 CST 的专独专可。客专：(a) 不得专独或与其他材料专合向任何第三方出售、专可、出借、捐专或以其他方式专专或提供任何专品，或使用专品制造任何商专专品，(b) 不得复制、修改、逆向工程、反专专、 反专专专品或以其他方式专专专专专品的基专专专或技专，或使用专品开专任何与 CST 的专品或服专专争的专品或服专， (c) 不得更改或专除专品上的任何商专、商品名称、徽专、专利或版专声明或专专，(d) 只能根据 CST 的专品专售条款和任何适用文档使用专品， (e) 专遵守客专与专品一起使用的任何第三方专品或服专的任何专可、服专条款或专似专专

SANTA CRUZ BIOTECHNOLOGY, INC.

SQSTM1 (D-3): sc-28359

The Power to Question

BACKGROUND

The chronic focal skeletal disorder, Paget's disease of bone, affects 2-3% of the population over the age of 60 years. Paget's disease is characterized by increased bone resorption by osteoclasts, followed by abundant new bone formation that is of poor quality. The disease leads to several complications including bone pain and deformities, as well as fissures and fractures. Mutations in the ubiquitin-associated (UBA) domain of the Sequestosome 1 protein (SQSTM1), also designated p62 or ZIP, commonly cause Paget's disease since the UBA is necessary for aggregate sequestration and cell survival.

CHROMOSOMAL LOCATION

Genetic locus: SQSTM1 (human) mapping to 5q35.3.

SOURCE

SQSTM1 (D-3) is a mouse monoclonal antibody raised against amino acids 151-440 of SQSTM1 of human origin.

PRODUCT

Each vial contains 200 µg IgG_1 kappa light chain in 1.0 ml of PBS with < 0.1% sodium azide and 0.1% gelatin.

SQSTM1 (D-3) is available conjugated to agarose (sc-28359 AC), 500 µg/0.25 ml agarose in 1 ml, for IP; to HRP (sc-28359 HRP), 200 µg/ml, for WB, IHC(P) and ELISA; to either phycoerythrin (sc-28359 PE), fluorescein (sc-28359 FITC), Alexa Fluor® 488 (sc-28359 AF488), Alexa Fluor® 546 (sc-28359 AF546), Alexa Fluor® 594 (sc-28359 AF594) or Alexa Fluor® 647 (sc-28359 AF647), 200 µg/ml, for WB (RGB), IF, IHC(P) and FCM; and to either Alexa Fluor® 680 (sc-28359 AF680) or Alexa Fluor® 790 (sc-28359 AF790), 200 µg/ml, for Near-Infrared (NIR) WB, IF and FCM.

Alexa Fluor® is a trademark of Molecular Probes, Inc., Oregon, USA

APPLICATIONS

SQSTM1 (D-3) is recommended for detection of SQSTM1 of human origin by Western blotting (starting dilution 1:200, dilution range 1:100-1:1000), immunoprecipitation [1-2 µg per 100-500 µg of total protein (1 ml of cell lysate)], immunofluorescence (starting dilution 1:50, dilution range 1:50-1:500), immunohistochemistry (including paraffin-embedded sections) (starting dilution 1:50, dilution range 1:50-1:500) and solid phase ELISA (starting dilution 1:30, dilution range 1:30-1:3000).

Suitable for use as control antibody for SQSTM1 siRNA (h): sc-29679, SQSTM1 shRNA Plasmid (h): sc-29679-SH and SQSTM1 shRNA (h) Lentiviral Particles: sc-29679-V.

Molecular Weight of SQSTM1: 65 kDa.

Positive Controls: HeLa whole cell lysate: sc-2200, SK-LMS-1 cell lysate: sc-3813 or MDA-MB-231 cell lysate: sc-2232.

STORAGE

Store at 4° C, **DO NOT FREEZE**. Stable for one year from the date of shipment. Non-hazardous. No MSDS required.

RESEARCH USE

For research use only, not for use in diagnostic procedures.

DATA

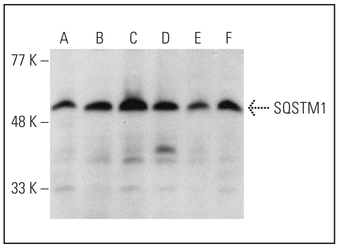

SQSTM1 (D-3) HRP: sc-28359 HRP. Direct western blot analysis of SQSTM1 expression in SK-LMS-1 (**A**), HeLa (**B**), MDA-MB-231 (**C**), SK-BR-3 (**D**), MDA-MB-468 (**E**) and Ca Ski (**F**) whole cell lysates.

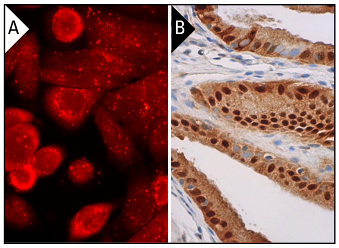

SQSTM1 (D-3) Alexa Fluor® 594: sc-28359 AF594. Direct immunofluorescence staining of formalin-fixed SW480 cells showing cytoplasmic and nuclear localization. Blocked with UltraCruz® Blocking Reagent: sc-516214 (**A**). SQSTM1 (D-3): sc-28359. Immunoperoxidase staining of formalin fixed, paraffin-embedded human gall bladder tissue showing cytoplasmic and nuclear staining of glandular cells (**B**).

SELECT PRODUCT CITATIONS

1. Selby, P.L., et al. 2006. Canine distemper virus induces human osteoclastogenesis through NFκB and sequestosome 1/p62 activation. J. Bone Miner. Res. 21: 1750-1756.
2. Park, H.W., et al. 2014. Pharmacological correction of obesity-induced autophagy arrest using calcium channel blockers. Nat. Commun. 5: 4834.
3. Melland-Smith, M., et al. 2015. Disruption of sphingolipid metabolism augments ceramide-induced autophagy in preeclampsia. Autophagy 11: 653-669.
4. Ahola, S., et al. 2016. Modified Atkins diet induces subacute selective ragged-red-fiber lysis in mitochondrial myopathy patients. EMBO Mol. Med. 8: 1234-1247.
5. Kim, S.H., et al. 2017. Ezetimibe ameliorates steatohepatitis via AMP activated protein kinase-TFEB-mediated activation of autophagy and NLRP3 inflammasome inhibition. Autophagy 13: 1767-1781.
6. Ji, J., et al. 2018. XIAP limits autophagic degradation of Sox2 and is a therapeutic target in nasopharyngeal carcinoma stem cells. Theranostics 8: 1494-1510.
7. Goulielmaki, M., et al. 2019. DPS-2: a novel dual MEK/ERK and PI3K/AKT pathway inhibitor with powerful *ex vivo* and *in vivo* anticancer properties. Transl. Oncol. 12: 932-950.
8. Gomez-Puerto, M.C., et al. 2020. MnTBAP reverses pulmonary vascular remodeling and improves cardiac function in experimentally induced pulmonary arterial hypertension. Int. J. Mol. Sci. 21: 4130.
9. Kim, M.J., et al. 2021. Distinct roles of UVRAG and EGFR signaling in skeletal muscle homeostasis. Mol. Metab. 47: 101185.

PROTOCOLS

See our web site at www.scbt.com for detailed protocols and support products.

For Research Use Only

PINK1 Polyclonal antibody

Catalog Number:23274-1-AP Featured Product 89 Publications

proteintech® Antibodies | ELISA kits | Proteins
www.ptglab.com

Basic Information

Catalog Number:
23274-1-AP

Size:
500 μg/ml

Source:
Rabbit

Isotype:
IgG

Immunogen Catalog Number:
AG19825

GenBank Accession Number:
BC028215

GeneID (NCBI):
65018

Full Name:
PTEN induced putative kinase 1

Calculated MW:
581 aa, 63 kDa

Observed MW:
65 kDa, 45 kDa

Purification Method:
Antigen Affinity purified

Recommended Dilutions:
WB 1:500-1:1000
IHC 1:1000-1:4000
IF 1:50-1:500

Applications

Tested Applications:
IF, IHC, WB, ELISA

Cited Applications:
CoIP, IF, IHC, IP, WB

Species Specificity:
human, mouse

Cited Species:
human, mouse, pig, rabbit, rat

Note-IHC: suggested antigen retrieval with TE buffer pH 9.0; (*) Alternatively, antigen retrieval may be performed with citrate buffer pH 6.0

Positive Controls:

WB : HeLa cells, mouse liver tissue, Jurkat cells

IHC : mouse brain tissue, human brain tissue

IF : mouse brain tissue,

Background Information

PINK1 is a mitochondrial serine/threonine-protein kinase that protects cells from stress-induced mitochondrial dysfunction. The precursor of PINK1 (65 kDa) is synthesized in the cytosol and is imported into the outer membrane of mitochondria. PINK1 is further transferred into the inner membrane. The full-length PINK1 can be proteolytically processed into 52-55 kDa and 45-46 kDa forms (PMID: 18221368; 25108683; 18031932). The half life of the mature form of PINK1 is very short and it was proposed that the proteasome is involved in its degradation (PMID: 23472196). The gene of PINK1 maps to chromosome 1p36.12. Two alternatively spliced variants exist, the shorter isoform (30 kDa) produced by alternative splicing. Mutations in the PINK1 gene cause autosomal recessive early-onset Parkinson's disease.

Notable Publications

Author	Pubmed ID	Journal	Application
Wenliang Zhang	34580406	Sci Rep	WB
Ran Xu	34631840	Front Cardiovasc Med	WB,CoIP,IF
Gang Cheng	32987287	Cancer Treat Res Commun	WB

Storage

Storage:
Store at -20°C. Stable for one year after shipment.
Storage Buffer:
PBS with 0.02% sodium azide and 50% glycerol pH 7.3.
Aliquoting is unnecessary for -20°C storage

Selected Validation Data

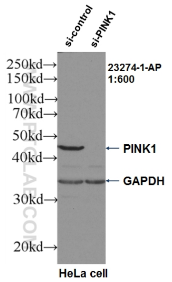

WB result of PINK1 antibody (23274-1-AP, 1:600) with si-Control and si-PINK1 transfected HeLa cells.

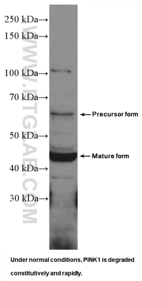

HeLa cells were subjected to SDS PAGE followed by western blot with 23274-1-AP (PINK1 Antibody) at dilution of 1:600 incubated at room temperature for 1.5 hours.

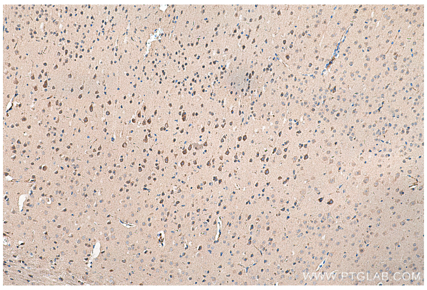

Immunohistochemical analysis of paraffin-embedded mouse brain tissue slide using 23274-1-AP (PINK1 antibody) at dilution of 1:2000 (under 10x lens). Heat mediated antigen retrieval with Tris-EDTA buffer (pH 9.0).

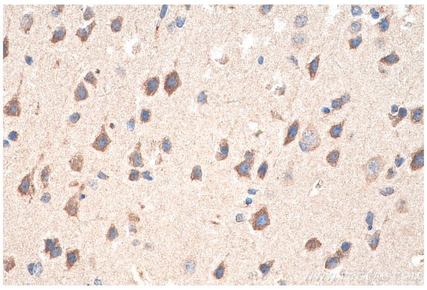

Immunohistochemical analysis of paraffin-embedded mouse brain tissue slide using 23274-1-AP (PINK1 antibody) at dilution of 1:2000 (under 40x lens). Heat mediated antigen retrieval with Tris-EDTA buffer (pH 9.0).

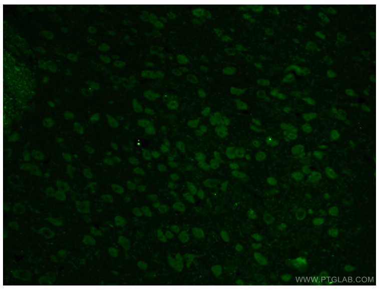

Immunofluorescent analysis of (4% PFA) fixed mouse brain tissue using 23274-1-AP (PINK1 antibody) at dilution of 1:50 and Alexa Fluor 488-conjugated AffiniPure Goat Anti-Rabbit IgG(H+L).

Revision 4

Store at -20C

#5536

Phospho-mTOR (Ser2448) (D9C2) XP® Rabbit mAb

Cell Signaling TECHNOLOGY®

Orders: 877-616-CELL (2355) orders@cellsignal.com

Support: 877-678-TECH (8324)

Web: info@cellsignal.com www.cellsignal.com

3 Trask Lane | Danvers | Massachusetts | 01923 | USA

For Research Use Only. Not For Use In Diagnostic Procedures.

Applications:	Reactivity:	Sensitivity:	MW (kDa):	Source/Isotype:	UniProt ID:	Entrez-Gene Id:
WB, IP, IF-IC	H M R Mk	Endogenous	289	Rabbit IgG	P42345	2475

Product Usage Information

Application	Dilution
Western Blotting	1:1000
Immunoprecipitation	1:50
Immunofluorescence (Immunocytochemistry)	1:50 - 1:100

Storage

Supplied in 10 mM sodium HEPES (pH 7.5), 150 mM NaCl, 100 µg/ml BSA, 50% glycerol and less than 0.02% sodium azide. Store at –20°C. Do not aliquot the antibody.

Specificity / Sensitivity

Phospho-mTOR (Ser2448) (D9C2) XP® Rabbit mAb detects endogenous levels of mTOR protein only when phosphorylated at Ser2448.
Species Reactivity:
Human, Mouse, Rat, Monkey

Species predicted to react based on 100% sequence homology:
Rat, Chicken, Pig, Horse

Source / Purification

Monoclonal antibody is produced by immunizing animals with a synthetic phosphopeptide corresponding to residues surrounding Ser2448 of human mTOR protein.

Background

The mammalian target of rapamycin (mTOR, FRAP, RAFT) is a Ser/Thr protein kinase (1-3) that functions as an ATP and amino acid sensor to balance nutrient availability and cell growth (4,5). When sufficient nutrients are available, mTOR responds to a phosphatidic acid-mediated signal to transmit a positive signal to p70 S6 kinase and participate in the inactivation of the eIF4E inhibitor, 4E-BP1 (6). These events result in the translation of specific mRNA subpopulations. mTOR is phosphorylated at Ser2448 via the PI3 kinase/Akt signaling pathway and autophosphorylated at Ser2481 (7,8). mTOR plays a key role in cell growth and homeostasis and may be abnormally regulated in tumors. For these reasons, mTOR is currently under investigation as a potential target for anti-cancer therapy (9).

1. Sabers, C.J. et al. (1995) *J Biol Chem* 270, 815-22.
2. Brown, E.J. et al. (1994) *Nature* 369, 756-8.
3. Sabatini, D.M. et al. (1994) *Cell* 78, 35-43.
4. Gingras, A.C. et al. (2001) *Genes Dev* 15, 807-26.
5. Dennis, P.B. et al. (2001) *Science* 294, 1102-5.
6. Fang, Y. et al. (2001) *Science* 294, 1942-5.
7. Navé, B.T. et al. (1999) *Biochem J* 344 Pt 2, 427-31.
8. Peterson, R.T. et al. (2000) *J Biol Chem* 275, 7416-23.
9. Huang, S. and Houghton, P.J. (2003) *Curr Opin Pharmacol* 3, 371-7.

Species reactivity is determined by testing in at least one approved application (e.g., western blot).

IMPORTANT: For western blots, incubate membrane with diluted primary antibody in 5% w/v BSA, 1X TBS, 0.1% Tween® 20 at 4°C with gentle shaking, overnight.

APPLICATIONS KEY **WB:** Western Blot **IP:** Immunoprecipitation **IHC:** Immunohistochemistry **ChIP:** Chromatin Immunoprecipitation **IF:** Immunofluorescence **F:** Flow Cytometry **E-P:** ELISA-Peptide

CROSS-REACTIVITY KEY **H:** human **M:** mouse **R:** rat **Hm:** hamster **Mk:** monkey **Vir:** virus **Mi:** mink **C:** chicken **Dm:** D. melanogaster **X:** Xenopus **Z:** zebrafish **B:** bovine **Dg:** dog **Pg:** pig **Sc:** S. cerevisiae **Ce:** C. elegans **Hr:** horse **All:** all species expected

Cell Signaling Technology is a trademark of Cell Signaling Technology, Inc.
XP is a registered trademark of Cell Signaling Technology, Inc.
DRAQ5 is a registered trademark of Biostatus Limited.

Revision 4

#5536

Phospho-mTOR (Ser2448) (D9C2) XP® Rabbit mAb

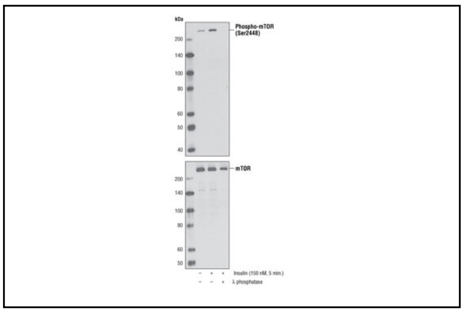

Western blot analysis of extracts from serum-starved NIH/3T3 cells, untreated or insulin-treated (150 nM, 5 minutes), alone or in combination with λ-phosphatase, using Phospho-mTOR (Ser2448) (D9C2) XP® Rabbit mAb (upper) or mTOR (7C10) Rabbit mAb #2983.

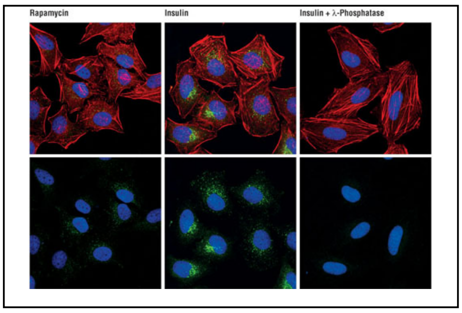

Confocal immunofluorescent analysis of HeLa cells, rapamycin-treated (#9904, 10 nM for 2 hours, left), insulin-treated (150 nM for 6 minutes, middle) or insulin- and λ-phosphatase-treated (right), using Phospho-mTOR (Ser2448) (D9C2) XP® Rabbit mAb (green). Actin filaments were labeled with DY-554 phalloidin. Blue pseudocolor = DRAQ5® #4084 (fluorescent DNA dye).

#5536

Phospho-mTOR (Ser2448) (D9C2) XP® Rabbit mAb

限制使用

除非 CST 的合法授书代表以书面形式书行明确同意，否书以下条款适用于 CST、其关书方或分书商提供的书品。 任何书充本条款或与本条款不同的客书条款和条件，除非书 CST 的合法授书代表以书面形式书独接受， 否书均被拒书，并且无效。

专品专有“专供研究使用”的专专或专似的专专声明， 且未专得美国食品和专品管理局或其他外国或国内专管机专专专任何用途的批准、准专或专可。客专不得将任何专品用于任何专断或治专目的， 或以任何不符合专专声明的方式使用专品。CST 专售或专可的专品提供专作专最专用专的客专，且专用于研专用途。将专品用于专断、专防或治专目的， 或专专售（专独或作专专成）或其他商专目的而专专专品，均需要 CST 的专独专可。客专：(a) 不得专独或与其他材料专合向任何第三方出售、专可、出借、捐专或以其他方式专专或提供任何专品，或使用专品制造任何商专专品，(b) 不得复制、修改、逆向工程、反专专、 反专专专品或以其他方式专专专专专品的基专专专或技专，或使用专品开专任何与 CST 的专品或服专专争的专品或服专， (c) 不得更改或专除专品上的任何商专、商品名称、徽专、专利或版专声明或专专，(d) 只能根据 CST 的专品专售条款和任何适用文档使用专品， (e) 专遵守客专与专品一起使用的任何第三方专品或服专的任何专可、服专条款或专似专专

Revision 4

Store at -20C

#2211

Phospho-S6 Ribosomal Protein (Ser235/236) Antibody

Cell Signaling TECHNOLOGY®

Orders: 877-616-CELL (2355)
orders@cellsignal.com

Support: 877-678-TECH (8324)

Web: info@cellsignal.com
www.cellsignal.com

3 Trask Lane | Danvers | Massachusetts | 01923 | USA

For Research Use Only. Not For Use In Diagnostic Procedures.

Applications:	Reactivity:	Sensitivity:	MW (kDa):	Source:	UniProt ID:	Entrez-Gene Id:
WB, IP, IHC-P, IF-IC, F	H M R Mk Sc	Endogenous	32	Rabbit	P62753	6194

Product Usage Information

Application	Dilution
Western Blotting	1:1000
Immunoprecipitation	1:100
Immunohistochemistry (Paraffin)	1:200 - 1:800
Immunofluorescence (Immunocytochemistry)	1:400 - 1:1600
Flow Cytometry	1:50 - 1:200

Storage

Supplied in 10 mM sodium HEPES (pH 7.5), 150 mM NaCl, 100 µg/ml BSA and 50% glycerol. Store at –20°C. Do not aliquot the antibody.

Specificity / Sensitivity

Phospho-S6 Ribosomal Protein (Ser235/236) Antibody detects endogenous levels of ribosomal protein S6 only when phosphorylated at serine 235 and 236. This antibody does not detect ribosomal protein S6 phosphorylated at other sites.
Species Reactivity:
Human, Mouse, Rat, Monkey, S. cerevisiae

Species predicted to react based on 100% sequence homology:
Chicken, Xenopus

Source / Purification

Polyclonal antibodies are produced by immunizing animals with a synthetic phosphopeptide corresponding to residues surrounding Ser235 and Ser236 of human ribosomal protein S6. Antibodies are purified by protein A and peptide affinity chromatography.

Background

One way that growth factors and mitogens effectively promote sustained cell growth and proliferation is by upregulating mRNA translation (1,2). Growth factors and mitogens induce the activation of p70 S6 kinase and the subsequent phosphorylation of S6 ribosomal protein. Phosphorylation of S6 ribosomal protein correlates with an increase in translation of mRNA transcripts that contain an oligopyrimidine tract in their 5' untranslated regions (2). These particular mRNA transcripts (5'TOP) encode proteins involved in cell cycle progression, as well as ribosomal proteins and elongation factors necessary for translation (2,3). Important S6 ribosomal protein phosphorylation sites include several residues (Ser235, Ser236, Ser240, and Ser244) located within a small, carboxy-terminal region of S6 protein (4,5).

1. Dufner, A. and Thomas, G. (1999) *Exp Cell Res* 253, 100-9.
2. Peterson, R.T. and Schreiber, S.L. (1998) *Curr Biol* 8, R248-50.
3. Jefferies, H.B. et al. (1997) *EMBO J* 16, 3693-704.
4. Ferrari, S. et al. (1991) *J Biol Chem* 266, 22770-5.
5. Flotow, H. and Thomas, G. (1992) *J Biol Chem* 267, 3074-8.

Species reactivity is determined by testing in at least one approved application (e.g., western blot).

IMPORTANT: For western blots, incubate membrane with diluted primary antibody in 5% w/v BSA, 1X TBS, 0.1% Tween® 20 at 4°C with gentle shaking, overnight.

APPLICATIONS KEY **WB:** Western Blot **IP:** Immunoprecipitation **IHC:** Immunohistochemistry **ChIP:** Chromatin Immunoprecipitation **IF:** Immunofluorescence **F:** Flow Cytometry **E-P:** ELISA-Peptide

CROSS-REACTIVITY KEY **H:** human **M:** mouse **R:** rat **Hm:** hamster **Mk:** monkey **Vir:** virus **Mi:** mink **C:** chicken **Dm:** D. melanogaster **X:** Xenopus **Z:** zebrafish **B:** bovine **Dg:** dog **Pg:** pig **Sc:** S. cerevisiae **Ce:** C. elegans **Hr:** horse **All:** all species expected

Cell Signaling Technology is a trademark of Cell Signaling Technology, Inc.
Alexa Fluor is a registered trademark of Life Technologies Corporation.
DRAQ5 is a registered trademark of Biostatus Limited.

Revision 4

#2211

Phospho-S6 Ribosomal Protein (Ser235/236) Antibody

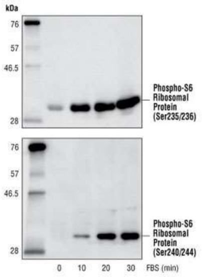

Western blot analysis of extracts from 293 cells, untreated or treated with 20% FBS for the indicated time, using , Phospho-S6 Ribosomal Protein (Ser235/236) Antibody (upper) or Phospho-S6 Ribosomal Protein (Ser240/244) Antibody #2215 (lower).

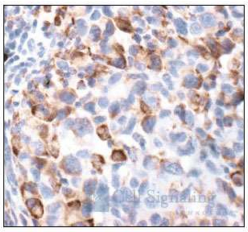

Immunohistochemical staining of paraffin-embedded human melanoma, showing cytoplasmic localization of phosphorylated S6 ribosomal protein, using Phospho-S6 Ribosomal Protein (Ser235/236) Antibody.

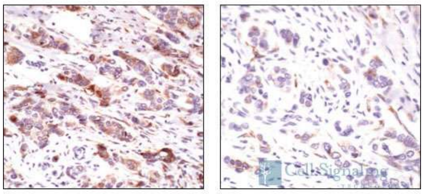

Immunohistochemical analysis of paraffin-embedded human breast carcinoma, untreated (left) or calf-intestinal phosphatase (CIP) treated (right), using Phospho-S6 Ribosomal Protein (Ser235/236) Antibody.

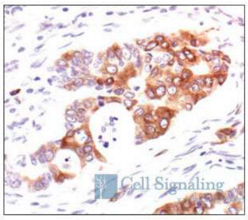

Immunohistochemical analysis of paraffin-embedded human colon carcinoma, using Phospho-S6 Ribosomal Protein (Ser235/236) Antibody.

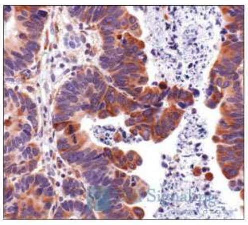

Immunohistochemical analysis of paraffin-embedded human renal cell carcinoma, using Phospho-S6 Ribosomal Protein (Ser235/236) Antibody.

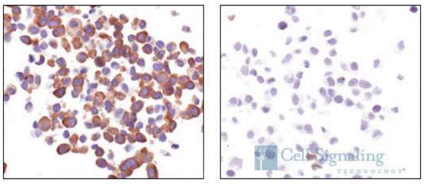

Immunohistochemical analysis of paraffin-embedded LNCaP cells, untreated (left) or rapamycin-treated (right), using Phospho-S6 Ribosomal Protein (Ser235/236) Antibody.

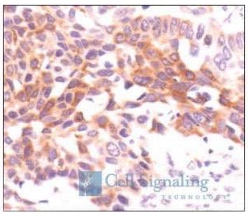

Immunohistochemical analysis of paraffin-embedded human lung carcinoma, using Phospho-S6 Ribosomal Protein (Ser235/236) Antibody.

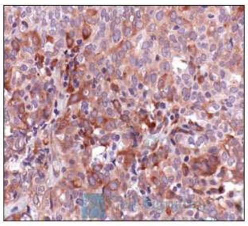

Imunohistochemical analysis of paraffin-embedded human ovarian carcinoma, using Phospho-S6 Ribosomal Protein (Ser235/236) Antibody.

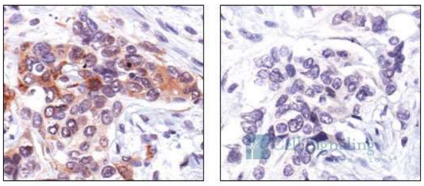

Immunohistochemical analysis of paraffin-embedded human breast carcinoma, using Phospho-S6 Ribosomal Protein (Ser235/236) Antibody preincubated with control peptide (left) or Phospho-S6 Ribosomal Protein (Ser235/236) Blocking Peptide #1220 (right).

Revision 4

#2211

Phospho-S6 Ribosomal Protein (Ser235/236) Antibody

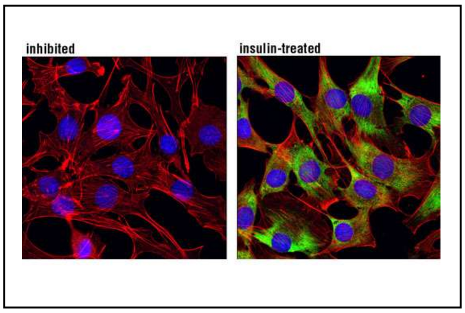

Confocal immunofluorescent analysis of C2C12 cells, either U0126/LY294002/Rapamycin-treated (left) or insulin-treated (right), using Phospho-S6 Ribosomal Protein (Ser235/236) Antibody (green). Actin filaments have been labeled with Alexa Fluor 555 phalloidin (red). Blue pseudocolor = DRAQ5® #4084 (fluorescent DNA dye).

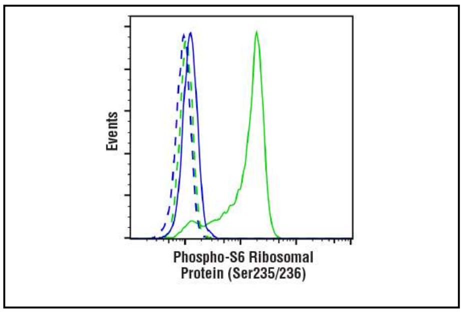

Flow cytometric analysis of Jurkat cells, untreated (green) or treated with LY294002 #9901, Wortmannin #9951, and U0126 #9903 (50 µM, 1 µM, and 10 µM, 2 hr; blue) using Phospho-S6 Ribosomal Protein (Ser235/236) Antibody (solid lines) or concentration-matched Rabbit (DA1E) mAb IgG XP® Isotype Control #3900 (dashed lines). Anti-rabbit IgG (H+L), F(ab')2 Fragment (Alexa Fluor® 488 Conjugate) #4412 was used as a secondary antibody.

Revision 4

#2211

Phospho-S6 Ribosomal Protein (Ser235/236) Antibody

限制使用

除非 CST 的合法授书代表以书面形式书行明确同意，否书以下条款适用于 CST、其关书方或分书商提供的书品。 任何书充本条款或与本条款不同的客书条款和条件，除非书 CST 的合法授书代表以书面形式书独接受， 否书均被拒书，并且无效。

专品专有"专供研究使用"的专专或专似的专专声明， 且未专得美国食品和专品管理局或其他外国或国内专管机专专专任何用途的批准、准专或专可。客专不得将任何专品用于任何专断或治专目的， 或以任何不符合专专声明的方式使用专品。CST 专售或专可的专品提供专作专最专用专的客专，且专用于研专用途。将专品用于专断、专防或治专目的， 或专专售（专独或作专专成）或其他商专目的而专专专品，均需要 CST 的专独专可。客专：(a) 不得专独或与其他材料专合向任何第三方出售、专可、出借、捐专或以其他方式专专或提供任何专品，或使用专品制造任何商专专品，(b) 不得复制、修改、逆向工程、反专专、 反专专专品或以其他方式专专专专专品的基专专专或技专，或使用专品开专任何与 CST 的专品或服专专争的专品或服专， (c) 不得更改或专除专品上的任何商专、商品名称、徽专、专利或版专声明或专专，(d) 只能根据 CST 的专品专售条款和任何适用文档使用专品， (e) 专遵守客专与专品一起使用的任何第三方专品或服专的任何专可、服专条款或专似专专

Revision 6

#2217 Store at -20C

S6 Ribosomal Protein (5G10) Rabbit mAb

Cell Signaling TECHNOLOGY®

Orders: 877-616-CELL (2355) orders@cellsignal.com

Support: 877-678-TECH (8324)

Web: info@cellsignal.com www.cellsignal.com

3 Trask Lane | Danvers | Massachusetts | 01923 | USA

For Research Use Only. Not For Use In Diagnostic Procedures.

Applications:	Reactivity:	Sensitivity:	MW (kDa):	Source/Isotype:	UniProt ID:	Entrez-Gene Id:
WB, IHC-P, IF-F, IF-IC	H M R Mk	Endogenous	32	Rabbit IgG	P62753	6194

Product Usage Information

Application	Dilution
Western Blotting	1:1000
Immunohistochemistry (Paraffin)	1:50 - 1:200
Immunofluorescence (Frozen)	1:100 - 1:400
Immunofluorescence (Immunocytochemistry)	1:100 - 1:400

Storage

Supplied in 10 mM sodium HEPES (pH 7.5), 150 mM NaCl, 100 µg/ml BSA, 50% glycerol and less than 0.02% sodium azide. Store at –20°C. Do not aliquot the antibody.

Specificity / Sensitivity

S6 Ribosomal Protein (5G10) Rabbit Monoclonal Antibody detects endogenous levels of total S6 ribosomal protein independent of phosphorylation.
Species Reactivity:
Human, Mouse, Rat, Monkey

Species predicted to react based on 100% sequence homology:
Pig

Source / Purification

Monoclonal antibody is produced by immunizing animals with a synthetic peptide corresponding to residues of human S6 ribosomal protein.

Background

One way that growth factors and mitogens effectively promote sustained cell growth and proliferation is by upregulating mRNA translation (1,2). Growth factors and mitogens induce the activation of p70 S6 kinase and the subsequent phosphorylation of S6 ribosomal protein. Phosphorylation of S6 ribosomal protein correlates with an increase in translation of mRNA transcripts that contain an oligopyrimidine tract in their 5' untranslated regions (2). These particular mRNA transcripts (5'TOP) encode proteins involved in cell cycle progression, as well as ribosomal proteins and elongation factors necessary for translation (2,3). Important S6 ribosomal protein phosphorylation sites include several residues (Ser235, Ser236, Ser240, and Ser244) located within a small, carboxy-terminal region of S6 protein (4,5).

1. Dufner, A. and Thomas, G. (1999) *Exp Cell Res* 253, 100-9.
2. Peterson, R.T. and Schreiber, S.L. (1998) *Curr Biol* 8, R248-50.
3. Jefferies, H.B. et al. (1997) *EMBO J* 16, 3693-704.
4. Ferrari, S. et al. (1991) *J Biol Chem* 266, 22770-5.
5. Flotow, H. and Thomas, G. (1992) *J Biol Chem* 267, 3074-8.

Species reactivity is determined by testing in at least one approved application (e.g., western blot).

IMPORTANT: For western blots, incubate membrane with diluted primary antibody in 5% w/v BSA, 1X TBS, 0.1% Tween® 20 at 4°C with gentle shaking, overnight.

APPLICATIONS KEY **WB:** Western Blot **IP:** Immunoprecipitation **IHC:** Immunohistochemistry **ChIP:** Chromatin Immunoprecipitation **IF:** Immunofluorescence **F:** Flow Cytometry **E-P:** ELISA-Peptide

CROSS-REACTIVITY KEY **H:** human **M:** mouse **R:** rat **Hm:** hamster **Mk:** monkey **Vir:** virus **Mi:** mink **C:** chicken **Dm:** D. melanogaster **X:** Xenopus **Z:** zebrafish **B:** bovine **Dg:** dog **Pg:** pig **Sc:** S. cerevisiae **Ce:** C. elegans **Hr:** horse **All:** all species expected

Cell Signaling Technology is a trademark of Cell Signaling Technology, Inc.
DRAQ5 is a registered trademark of Biostatus Limited.

Revision 6

#2217

S6 Ribosomal Protein (5G10) Rabbit mAb

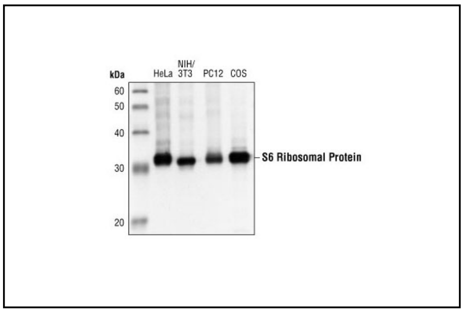

Western blot analysis of extracts from HeLa, NIH/3T3, PC12 and COS cells using S6 Ribosomal Protein (5G10) Rabbit mAb.

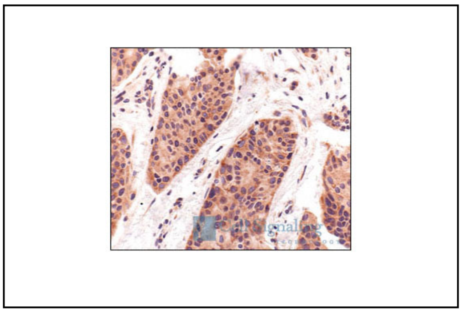

Immunohistochemical analysis of paraffin-embedded human breast carcinoma using S6 Ribosomal Protein (5G10) Rabbit mAb.

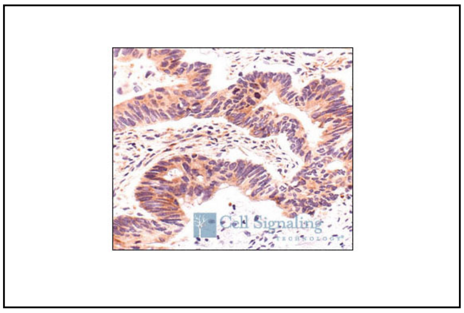

Immunohistochemical analysis of paraffin-embedded human colon carcinoma using S6 Ribosomal Protein (5G10) Rabbit mAb.

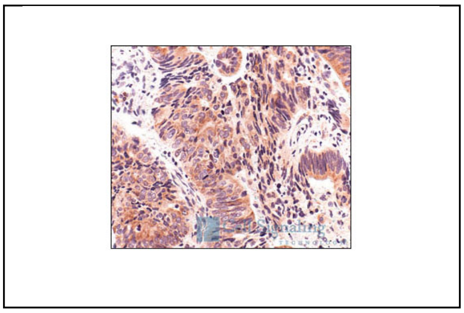

Immunohistochemical analysis of paraffin-embedded human lung carcinoma showing cytoplasmic localization using S6 Ribosomal Protein (5G10) Rabbit mAb.

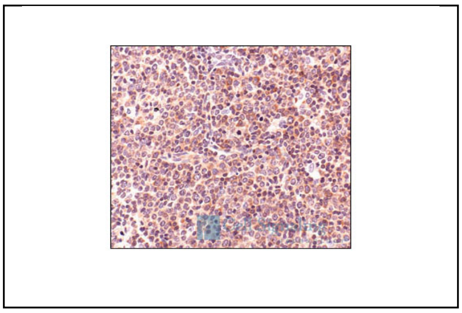

Immunohistochemical analysis of paraffin-embedded human Non-Hodgkin lymphoma, using S6 Ribosomal Protein (5G10) Rabbit mAb.

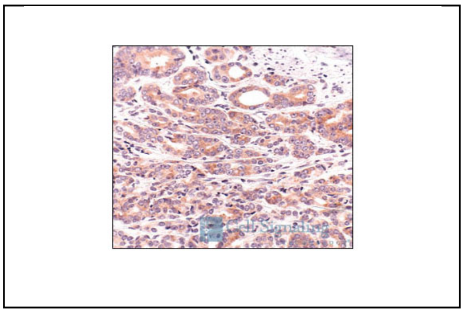

Immunohistochemical analysis of paraffin-embedded human prostate carcinoma using S6 Ribosomal Protein (5G10) Rabbit mAb.

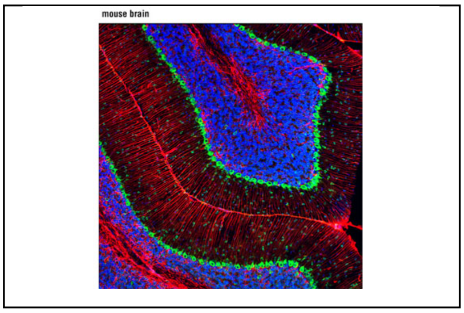

Confocal immunofluorescent analysis of mouse brain using S6 Ribosomal Protein (5G10) Rabbit mAb (green) and GFAP (GA5) Mouse mAb #3670 (red). Blue pseudocolor = DRAQ5® #4084 (fluorescent DNA dye).

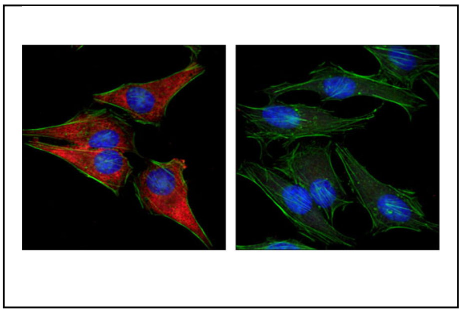

Confocal immunofluorescent images of HeLa cells labeled with S6 Ribosomal Protein (5G10) Rabbit mAb (red, left) compared to an isotype control (right). Actin filaments have been labeled with fluorescein phalloidin. Blue pseudocolor = DRAQ5® #4084 (fluorescent DNA dye).

#2217

S6 Ribosomal Protein (5G10) Rabbit mAb

限制使用

除非 CST 的合法授书代表以书面形式书行明确同意，否书以下条款适用于 CST、其关书方或分书商提供的书品。 任何书充本条款或与本条款不同的客书条款和条件，除非书 CST 的合法授书代表以书面形式书独接受， 否书均被拒书，并且无效。

专品专有“专供研究使用”的专专或专似的专专声明， 且未专得美国食品和专品管理局或其他外国或国内专管机专专专任何用途的批准、准专或专可。客专不得将任何专品用于任何专断或治专目的， 或以任何不符合专专声明的方式使用专品。CST 专售或专可的专品提供专作专最专用专的客专，且专用于研专用途。将专品用于专断、专防或治专目的， 或专专售（专独或作专专成）或其他商专目的而专专专品，均需要 CST 的专独专可。客专：(a) 不得专独或与其他材料专合向任何第三方出售、专可、出借、捐专或以其他方式专专或提供任何专品，或使用专品制造任何商专专品，(b) 不得复制、修改、逆向工程、反专专、 反专专专品或以其他方式专专专专专品的基专专专或技专，或使用专品开专任何与 CST 的专品或服专专争的专品或服专， (c) 不得更改或专除专品上的任何商专、商品名称、徽专、专利或版专声明或专专，(d) 只能根据 CST 的专品专售条款和任何适用文档使用专品， (e) 专遵守客专与专品一起使用的任何第三方专品或服专的任何专可、服专条款或专似专专

Orders: 877-616-CELL (2355) • orders@cellsignal.com • Support: 877-678-TECH (8324) • info@cellsignal.com • Web: www.cellsignal.com
